# Cyclic Thiosulfonates for Thiol-Mediated Uptake: Cascade Exchangers, Transporters, Inhibitors

**DOI:** 10.1021/jacsau.1c00573

**Published:** 2022-03-22

**Authors:** Takehiro Kato, Bumhee Lim, Yangyang Cheng, Anh-Tuan Pham, John Maynard, Dimitri Moreau, Amalia I. Poblador-Bahamonde, Naomi Sakai, Stefan Matile

**Affiliations:** Department of Organic Chemistry, University of Geneva, 1211 Geneva, Switzerland

**Keywords:** dynamic covalent chemistry, cyclic thiosulfonates, exchange cascades, proticity, cellular uptake, lentivector entry

## Abstract

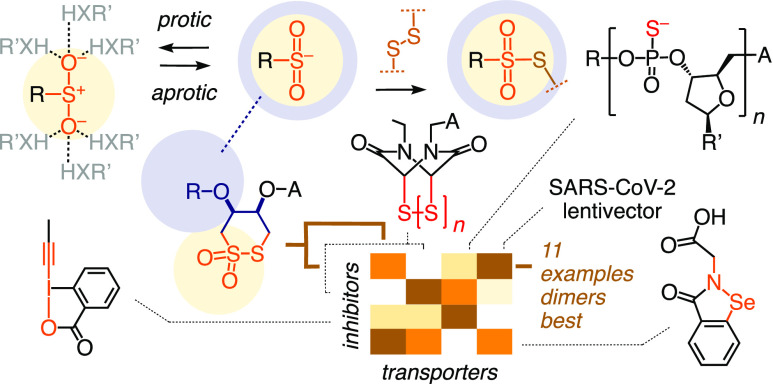

Thiol-mediated uptake is emerging as a powerful method to penetrate cells. Cyclic oligochalcogenides (COCs) have been identified as privileged scaffolds to enable and inhibit thiol-mediated uptake because they can act as dynamic covalent cascade exchangers, i.e., every exchange produces a new, covalently tethered exchanger. In this study, our focus is on the essentially unexplored COCs of higher oxidation levels. Quantitative characterization of the underlying dynamic covalent exchange cascades reveals that the initial ring opening of cyclic thiosulfonates (CTOs) proceeds at a high speed even at a low pH. The released sulfinates exchange with disulfides in aprotic but much less in protic environments. Hydrophobic domains were thus introduced to direct CTOs into hydrophobic pockets to enhance their reactivity. Equipped with such directing groups, fluorescently labeled CTOs entered the cytosol of living cells more efficiently than the popular asparagusic acid. Added as competitive agents, CTOs inhibit the uptake of various COC transporters and SARS-CoV-2 lentivectors. Orthogonal trends found with different transporters support the existence of multiple cellular partners to account for the diverse expressions of thiol-mediated uptake. Dominant self-inhibition and high activity of dimers imply selective and synergistic exchange in hydrophobic pockets as distinguishing characteristics of thiol-mediated uptake with CTOs. The best CTO dimers with hydrophobic directing groups inhibit the cellular entry of SARS-CoV-2 lentivectors with an IC_50_ significantly lower than the previous best CTO, below the 10 μM threshold and better than ebselen. Taken together, these results identify CTOs as an intriguing motif for use in cytosolic delivery, as inhibitors of lentivector entry, and for the evolution of dynamic covalent networks in the broadest sense, with reactivity-based selectivity of cascade exchange emerging as a distinguishing characteristic that deserves further attention.

## Introduction

The classical “sulfur redox switch” refers to the oxidation into sulfoxides and sulfones and their respective reduction back to sulfides.^1−6^ This redox switch has emerged as a valuable tool to build functional supramolecular systems, particularly concerning on/off switching of peptide secondary structures,^1−3^ amphiphilicity,^1,2^ π-acidity,^5^ optical properties,^4,5^ chirality,^1,5^ macrodipoles,^1,5,6^ membrane permeability,^2,6^ voltage-gated ion channels,^6^ and so on. The complementary oxidation of disulfides did not attract attention in a similar context.^7^

**Figure 1:**
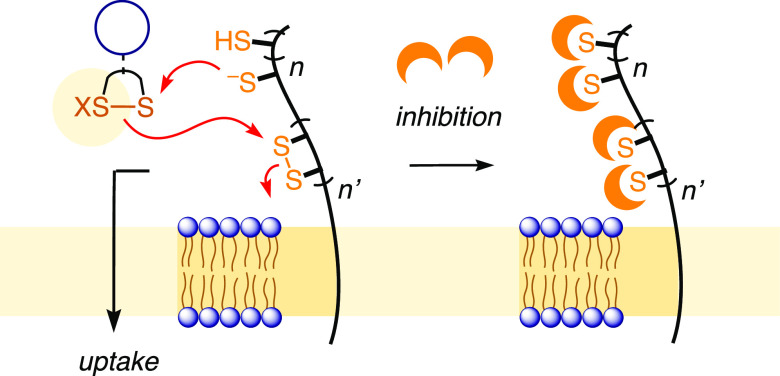
Thiol-mediated uptake, which occurs and gets inhibited by dynamic covalent sulfur exchange with cellular partners, is explored here using cyclic disulfides, cyclic thiosulfinates (X = O), as well as cyclic thiosulfonates (CTO, X = O_2_).

Unlike most sulfides,^8−11^ disulfides are dynamic by nature, and their exchange is ubiquitous in chemistry and biology.^12−37^ Cyclic oligochalcogenides (COCs) are of particular interest because they provide access to dynamic covalent exchange cascades.^12,38−46^ In the best explored cyclic disulfides, exchange with thiols produces a new, covalently tethered thiol that can continue to exchange (Figure 1).

**Figure 2:**
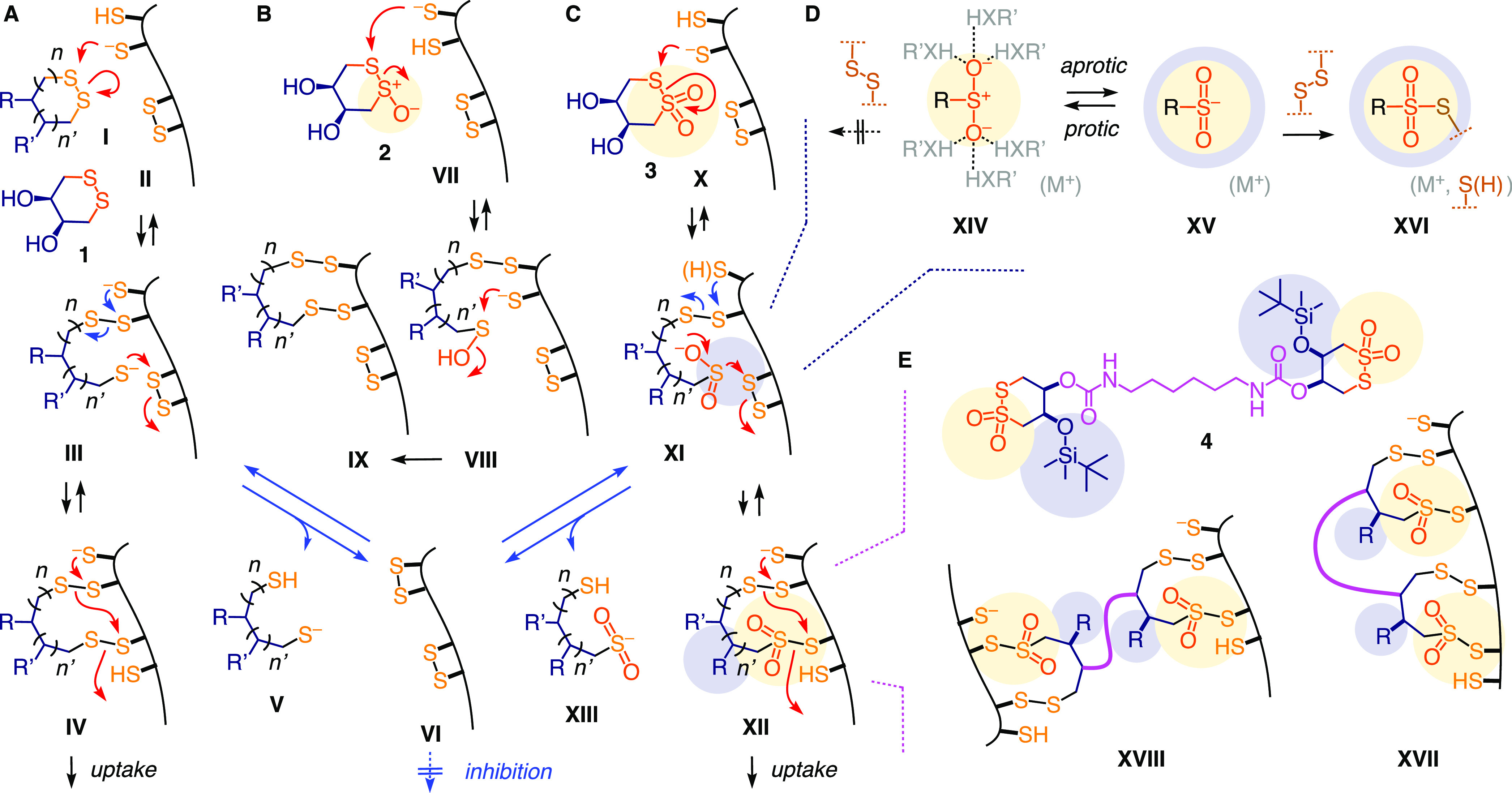
Conceivable dynamic covalent exchange cascades of (A) cyclic disulfides, (B) thiosulfinates, and (C) thiosulfonates. (D) The concept of reactivity-based selectivity and directing groups: Activation of dynamic covalent sulfinate chemistry in aprotic environments. (E) Possible exchange cascades of thiosulfonate dimers with cellular thiols and disulfides during uptake.

Analogous to sulfides, the oxidation of cyclic disulfides affords cyclic thiosulfinates^12,46−48^ and cyclic thiosulfonates (CTOs).^7,12,48−60^ Cyclic thiosulfinates have been shown to react rapidly and selectively with proximal thiols to result in their cross-linking through disulfide bonds (Figure 2).^46^ Pioneering studies by Castellano and co-workers support that CTOs can exchange with one thiol and one disulfide to afford a new disulfide, a new thiosulfonate, and a new thiol.^49^ Such exchange cascades on the sulfur-rich growth factor receptors and helicase zinc fingers have been implied to account for antitumor and antiviral activity of selected CTOs.^49,50^

Dynamic covalent exchange cascades with cellular thiols and disulfides are the hallmark of thiol-mediated uptake.^12,30,37,51,61^ This approach, defined by inhibition with thiol-reactive agents, is increasingly recognized as an attractive, general, and practical method to deliver substrates of free choice into cells. Reconsidering early insights into HIV,^61^ the inhibition of thiol-mediated uptake received renewed attention with the outbreak of the SARS-CoV-2 pandemic.^51^ Besides direct translocation across the plasma membrane,^12−15^ thiol-mediated uptake can also occur in combination with endocytosis,^52,62^ fusion,^61^ or mixed mechanisms. Thiol-mediated direct translocation into the cytosol has so far been explored with COCs covering disulfides of varied ring tension,^38−41^ diselenides,^42^ and benzopolysulfanes.^43^ Emerging as a general concept beyond COCs, we propose to refer to molecules with this reactivity as “cascade exchangers”, or CAXs. Their definition will be further generalized to dynamic covalent exchangers that, upon exchange with thiols (or disulfides), produce a new (or offer another) covalently tethered exchanger that can continue to exchange. This form also covers, for instance, oligonucleotide phosphorothioates^52^ or all acyclic oligo- and polymers, including the increasingly popular cell-penetrating poly(disulfide)s.^12−16,36^

COCs on higher oxidation levels have not been considered for thiol-mediated uptake. However, recent screenings revealed CTOs as good inhibitors of both thiol-mediated uptake and the cellular entry of SARS-CoV-2 lentiviral vectors.^51^ Herein, we examine CTOs as potentially distinct new CAX. Exchange cascades with CTOs are characterized by fast ring opening and selective continuation in aprotic surroundings. This reactivity-based selectivity is shown to translate into efficient cytosolic delivery that can be inhibited with distinct selectivity. CTOs also inhibit the cellular entry of lentivectors, the best below the 10 μM threshold.

## Results and Discussion

### Concepts

Ring-opening dynamic covalent exchange with “exofacial” thiol/ates on the cell surface initiates thiol-mediated cytosolic delivery of cyclic disulfides **I** (Figure 2A, **II**).^12^ The released but covalently tethered thiol/ate can then continue to exchange with accessible disulfides, either nearby or after target conformational changes (**III**), to result in two disulfides bridging the opened CAX and the cellular target(s) (**IV**). Since the first exofacial thiol often originates from a disulfide, a nearby thiol remains to assist the continuation of the exchange cascade through locally and temporarily misfolded protein targets to cross the membrane into the cytosol eventually. This process presumably involves also membrane disorganization, that is, transient micellar pores.^12^ Alternatively, the nearby thiol could exchange with the newly formed disulfide to release reduced dithiol CAXs (**V**) while blocking two essential exofacial thiols as disulfide (**VI**), thereby inhibiting thiol-mediated uptake.

To launch exchange cascades with disulfide COCs, the activation of ring opening by ring tension as in 1,2-dithiolanes has proven powerful.^38^ There is no evidence for significant thiol-mediated uptake with 1,2-dithianes, presumably because the equilibrium is shifted to the ring-closed side, a characteristic best known from dithiothreitol as a universal disulfide reducing agent. Oxidation of the diastereomeric dithioerythritol leads to cyclic disulfide **1** followed by the cyclic thiosulfinate **2** and particularly CTO **3**, the central motif of this study. Exchange of cyclic thiosulfenates such as **2** with thiols is fast (**VII**) and releases covalently tethered sulfinate, which reacts with a nearby thiol (**VIII**) to yield another disulfide (**IX**, Figure 2B).^46^ Since the second step is irreversible, the overall equilibrium shifts to the product side.^46^ The resulting, doubly disulfide bridged product **IX** lost two exofacial thiols, suggesting that cyclic thiosulfinates should be better inhibitors than transporters, although initiation of an exchange cascade by another thiol/ate is of course conceivable.

With CTOs like **3**, the propensity of ring opening should be even higher (**X**, Figure 2C). The released but tethered sulfinate (p*K*_a_ = 2.7) in intermediate **XI** is not only a good leaving group but also a weak nucleophile, possibly to be considered as a proticity-dependent thiolate mimic capable of exchanging with nearby disulfides (Figure 2D, *vide infra*).^48,49,63−65^ Different from intermediate **IV**, the resulting doubly bridged intermediate **XII** contains one thiosulfonate bridge. In exchange cascades triggered by nearby thiol/ates, participation of this preserved thiosulfonate should make a difference in both reactivity and selectivity. Analogous to **III**, intermediate **XI** can also undergo disulfide exchange with a nearby thiol to release the reduced CAX **XIII** and the fully oxidized target **VI**. Masking of essential exofacial thiols as disulfides (**IV**, **VI**, or **IX**) or thiosulfonate (**XII**) should have a detrimental effect on the thiol-mediated uptake of reporters and viruses.

Three strategies were considered to increase the inhibitory and uptake activity of CTOs further. (i) The addition of activating substituents on the cycle to increase reactivity is conceptually trivial and mechanistically intriguing, as detailed in the cascade analysis part of this study. (ii) Hydrogen-bonding to the oxygens is expected to deactivate the sulfinate obtained by ring-opening exchange as a nucleophile (**XIV**, Figure 2D).^66^ In proteins, sulfinates are produced by the oxidation of cysteine thiols, but deactivated through hydrogen bonding, thus rarely exchanging with disulfides to form thiosulfonate bridges.^67^ We therefore decided to surround CTOs by hydrophobic groups, which should direct CTOs into aprotic pockets of proteins or membranes to prevent such inactivation (e.g., Figure 2C, **XI**, **XII**, blue circles, 2D, **XV**). Since thiolates are less hydrogen-bonded, they will gain nucleophilicity only moderately by their destabilization in a less polar environment^68^ and rather be inactivated by protonation due to increased p*K*_a_. Thus, the equilibrium of sulfinate-disulfide exchange should shift toward the side of the thiosulfonate (**XVI**) and thiol products in aprotic environments.^64,65^ This concept of reactivity-based selectivity from directing groups could be expected to add unique characteristics to CTO exchange cascades. (iii) CTO dimers such as **4** were attractive considering that thiol-mediated uptake is thought to operate with dynamic covalent exchange cascades. Dimers with appropriate tether length could thus engage in multivalent exchanges with two targets within the same (**XVII**) or different proteins (**XVIII**, Figure 2E) to strengthen their dynamic covalent binding.^69,70^

### Synthesis

The synthesis of all inhibitors and transporters explored in this study was relatively straightforward (Schemes S1–S29). The CTO dimer **4**, for instance, was accessible from dithioerythritol (DTE) **5** in four steps (Scheme 1). Like the oxidation of sulfides into sulfoxides and sulfones, disulfides can be oxidized into thiosulfinates and thiosulfonates with *m*CPBA or H_2_O_2_. This oxidation introduced asymmetry. With stereochemistry less relevant at this early stage, CTO **3** was used as a racemic mixture. Monosilylation gave **6** with high site-selectivity. The second alcohol was reacted first with *p*-nitrophenyl (PNP) chloroformate, and the stable intermediate **7** was then reacted with diamine **8**. Dimer **4** was obtained as a mixture of three stereoisomers, one pair of enantiomers, and the *meso* compound.

**Scheme 1:**
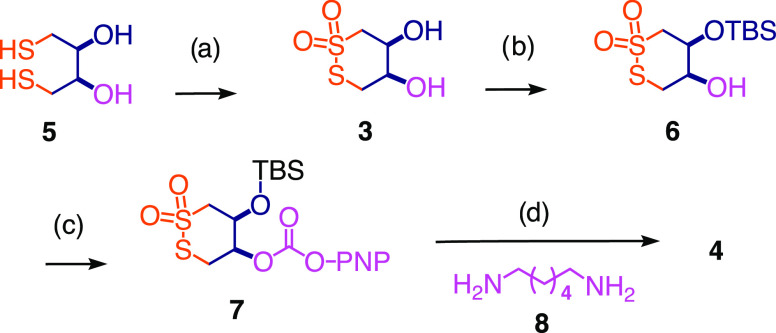
Synthesis of Cyclic Thiosulfonate Dimers H_2_O_2_, AcOH, rt, 20 h, 41%. TBSOTf, 2,6-lutidine, THF, −78 °C to rt, 3.5 h, 87%. PNP chloroformate, pyridine, CH_2_Cl_2_, rt, 3 h, 86%. H_2_N(CH_2_)_6_NH_2_, *i*Pr_2_NEt, DMF, rt, 2 h, 89%.

### Cascade Exchange

One objective of this study was to identify properties, including less obvious ones, that could be of use to elucidate thiol-mediated uptake. In this spirit, chalcogen bonding^71−76^ of anions to sulfur was considered as an unorthodox approach to possibly parametrize the electrophilicity of cascade exchangers. Related to the antibonding σ* orbital, ideal chalcogen bonds extend linearly from the covalent S–S bond.

**Figure 3:**
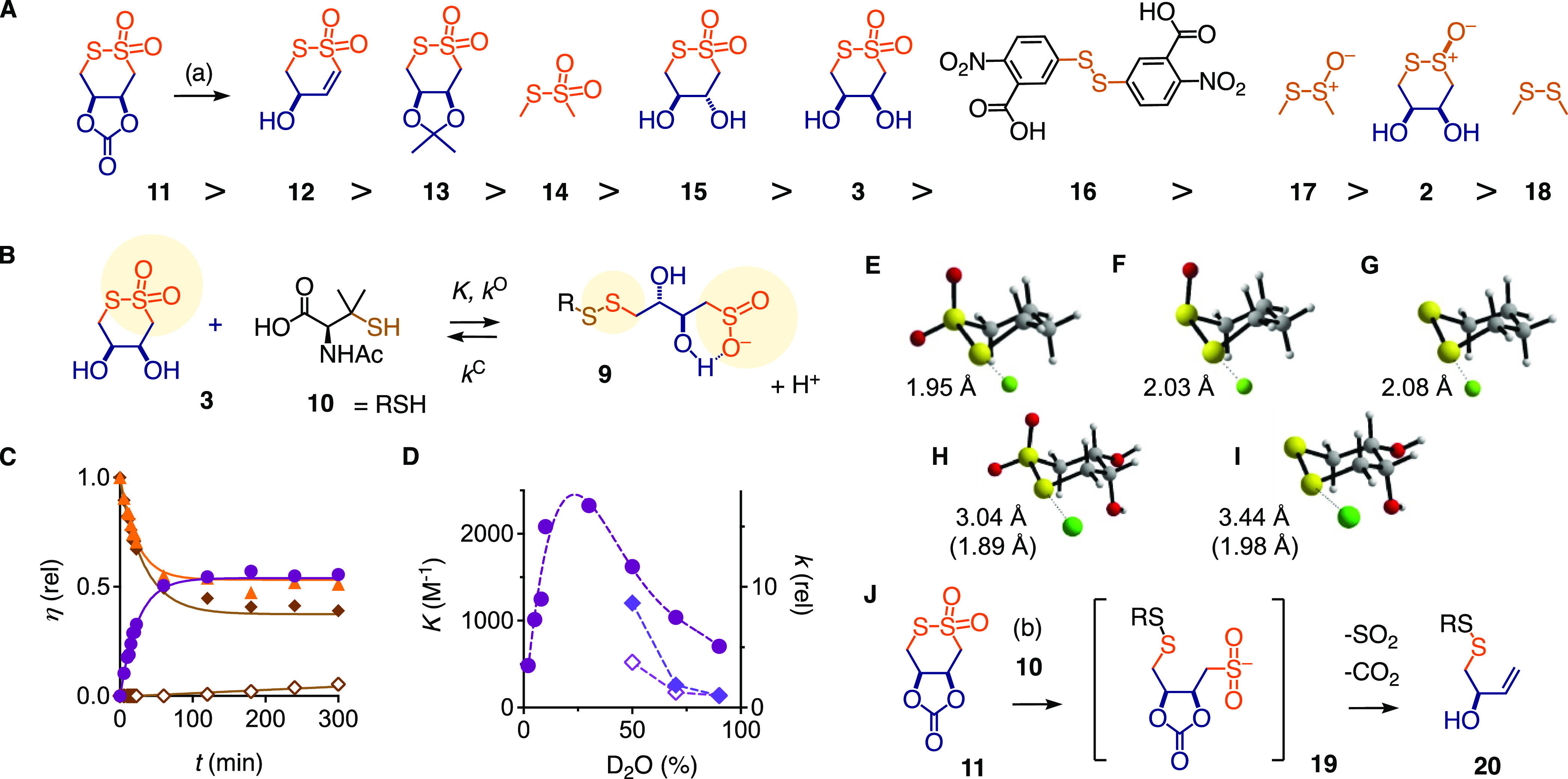
(A) Comparison of ring-opening rate constants (*k*^O^) for the exchange reactions of **2**, **3**, and **11**–**18** with **10** (Table 1); (a) deuterated buffer/DMSO-*d*_6_ 95:5, rt, *t*_50_ = <15 min, 84 min, 18 h, and ≫24 h at pD 7.9, 6.7, 5.4, and 4.7, respectively. (B) Dynamic covalent ring opening of CTO **3** and thiol **10** to give product **9**. (C) Original kinetic data for the equilibration of **3** with **10** at pD 2.7 in deuterated phosphate buffer/DMSO-*d*_6_ 9:1. Gold triangles: **3**; purple circles: **9**; filled diamonds: **10**; empty diamonds: homo-disulfide of **10**. (D) Dependence of *K* (filled circles) and rate constants (relative, *k* at 90% D_2_O as 1, *k*^O^ filled diamonds, *k*^C^ empty diamonds) for **3** with **10** at pD 2.7 (estimate) on solvent polarity. (E–G) Fluoride complexes of 1,2-dithiane 1,1-dioxide (E), 1,2-dithiane 1-oxide (F), and 1,2-dithiane (G), calculated with M06-2X/6-311++G** (green: F^–^, yellow: S, red: O, gray: C, white: H). (H, I) Chloride complexes of **3** (H) and **1** (I, green: Cl^–^, in parentheses: bond length for F^–^). (J) Fragmentation of **11** with **10**; (b) **10**, DMSO-*d*_6_, rt.

Various hydrogen-bonding patterns with axial and equatorial oxygen substituents and their binding to the anion added too many variables for computational modeling with the vicinal alcohols in **1**–**3** for straightforward predictions. Moreover, hydroxyl groups are absent in the most active dimer **4** and the cyclic carbonate (below). Computational simulations were thus initiated with the substituent-free 1,2-dithiane 1,1-dioxide (Figure 3E). For fluoride as chalcogen-bond acceptor, the M06-2X/6-311++G** minimized^76^ structure placed the anion at 1.95 Å from the sulfur, which is shorter than the sum of their Van der Waals (VdW) radii (3.27 Å) and longer than covalent S–F bonds (∼1.6 Å, Figure 3E). By formal reduction to 1,2-dithiane 1-oxide and 1,2-dithiane, the fluoride–sulfur distances increased to 2.03 and 2.08 Å (Figure 3F,G). The 1,2-dithiane 1-oxide with the oxygen in axial position was confirmed^51,77^ to be more stable (–2.65 kcal mol^–1^), and its chalcogen bond marginally shorter than that in the isomer with equatorial oxygen (2.04 Å, Figure S59a). Chalcogen-bond length thus correlated well with ring-opening rates (see below). Chalcogen-bond angles were >171° for all cases, close to perfection without much room for improvement.

Calculations with chloride in place of fluoride did not afford stable complexes for 1,2-dithiane, its thiosulfinate and thiosulfonate. For CTO **3**, modeled in a conformation without contributions from hydrogen bonding, a stable chloride complex could be computed with a chalcogen-bond angle of 172° and a bond length of 3.04 Å (VdW radii = 3.55 Å, S···F^–^: 1.89 Å, Figures 3H and S59b). The chloride complex of disulfide **1** was stable as well, featuring a chalcogen bond length of 3.44 Å and an angle of 161°, that is 19° deviated from linearity (S···F^–^: 1.98 Å, Figures 3I and S59c). These results were in qualitative agreement with those of F^–^ binding to the 1,2-dithiane redox series, thus further calculations with thiosulfinate **2** were considered unnecessary. Taken together, the consistent trends obtained suggested that computed length of chalcogen bonds could serve as valuable contributors to parametrize the exchange cascades accounting for thiol-mediated uptake.

The dynamic covalent opening of CTO **3** with thiols yields product **9** with a disulfide and a sulfinate (Figure 3B). Consistent with short chalcogen bonds found *in silico*, the opening of CTO **3** was instantaneous on an NMR timescale with most thiols. To decelerate ring opening, bulky thiols were considered in combination with lowered pH to deactivate the nucleophile. With *N*-Ac-d-penicillamine **10**, the opening of 5 mM CTO **3** in phosphate buffer/DMSO-*d*_6_ 9:1 was still immediate at pD 6.7 but became detectable in ^1^H NMR spectroscopy kinetics under more acidic conditions. The ring-opening rate constants *k*^O^ were estimated by fitting the initial velocities to second-order kinetics. Only lower bound rates were approximated from the earliest determined substrate conversions when reactions were still too fast despite the unfavorable conditions. At pD 5.4, dynamic covalent ring opening of CTO **3** with equimolar tertiary thiol **10** occurred with *k*^O^ > 0.49 M^–1^ s^–1^ and equilibrated at *K* = 6.0 × 10^3^ M^–1^ (Table 1, entry 1, Figures S4 and S5). At pD 2.7, further deceleration to *k*^O^ = 0.10 M^–1^ s^–1^ shifted the equilibrium to *K* = 500 M^–1^ (Table 1, entry 2, Figures S6 and S7). Original kinetics for equimolar exchangers revealed how these data translate to about 50% conversion, yielding at equilibrium about equal amounts of closed CTO **3** and opened sulfinate **9** as a mixture of diastereomers (Figure 3C, circles and triangles). At equilibrium, the concentration of thiol **10** further decreased slowly due to oxidation into the homodimer (Figure 3C, diamonds).

**Table 1:** Cascade Exchange Analysis^a^

	C^b^	pD^c^	D_2_O (%)^d^	*c*^e^ (mM)	*k*^O^ f (M^–1^ s^–1^)	*k*_rel_^O^ g	*K*^h^ (10^3^ M^–1^)
1	**3**	5.4	90	5	>0.49	1	6.0
2	**3**	2.7	90	5	0.10^i^	1	0.50^j^
3	**3**	2.7^k^	90	5	0.15		0.71
4	**3**	2.7^k^	70	5	0.28		1.0
5	**3**	2.7^k^	50	5	>1.3		1.8
6	**3**	2.7^k^	30	5	>2.8		2.3
7	**3**	2.7^k^	10	5	>2.1		2.1
8	**3**	2.7^k^	8	5	>1.4		1.3
9	**3**	2.7^k^	5	5	>1.3		0.94
10	**3**	2.7^k^	2	5	>1.4		0.73
11	**15**	2.7	90	5	>0.56	>5.6	2.0
12	**11**	2.7	90	2	>12	>120	>170
13	**12**	2.7	90	2	>6.1	>61	21
14	**13**	2.7	90	2	2.3	23	17
15	**14**	2.7	90	2	0.52	5.2	
16	**16**	5.4	90	5	0.11	<0.22	
17	**17**	5.4	85^l^	5^m^	0.004	<0.008	
18	**2**	5.4	85^l^	5^m^	0.001	<0.002	
19	**18**	5.4	90	5	NR		

a From ^1^H NMR kinetics with equimolar **10** at rt (Figure 3).

b Cascade exchangers and acyclic controls (Figure 3).

c pD of the same concentration and composition of Na^+^ phosphate solution in D_2_O.

d Fraction of D_2_O in a mixture with DMSO-*d*_6_ containing Na^+^ phosphate (180 mM, unless stated), pD as indicated.

e Substrate concentration.

f Rate constant of ring opening (or equivalent for acyclic controls).

g Rate constant of ring opening relative to **3** at pD 2.7 or 5.4.

h Equilibrium constant for ring opening and closing.

i Mean of independent experiments (SEM = 30%; SEM of technical replicates: 3%).

j Mean of independent experiments (SEM = 10%; SEM of technical replicates: 0.4%).

k Estimated pD. Na^+^ phosphate (20 mM in solvent mixture).

l Na^+^ phosphate (170 mM in solvent mixture).

m 2 equiv of **10** were used.

Cascade exchangers and controls **2**, **11**–**18** were tested under similar conditions (Figures 3A and S8–S27). As expected, the opening of cyclic thiosulfinate **2** was >500× slower (Table 1, entry 18). Among acyclic controls, thiosulfonate **14** exchanged faster than thiosulfinate **17** and disulfide **18**, which did not exchange under these unfavorable conditions (entries 15, 17, 19). The activated disulfide **16**, i.e., Ellman’s reagent DTNB, exchanged faster than thiosulfinates **17** and **2** but >5× slower than the lead CTO **3** (entry 16). The dithiothreitol (DTT) diastereomer **15** opened 5× as fast as **3** and equilibrated more on the opened side, perhaps because the intramolecular hydrogen bonding in the open form is more favorable (entry 11).

The strained cyclic carbonate **11** was considered to accelerate CTO opening and further stabilize the opened form (entry 12). However, at pD 7.9, carbonate **11** decarboxylated within 15 min to CTO alkene **12**. This degradation decelerated with decreasing pD to afford stable carbonate **11** for at least 1 day at pD 4.7 (Figure 3A). Ring opening of **11** was too fast even at pD 2.7, it proceeded to near completion with a >100× higher rate than **3**. Decarboxylated alkene **12** reacted also very fast, the apparently lower rate is the consequence of more favorable ring closure back to **12** due to preorganization by the *cis* alkene (entry 13). The alternative Michael addition of thiol **10** to alkene **12** was not observed (Figure S12).

With thiol **10** in DMSO-*d*_6_ without aqueous phosphate solution, carbonate **11** further fragmented through **19** into **20**, releasing SO_2_ and CO_2_ (Figures 3J and S44, Scheme S30). The cyclic acetal **13** was thus considered as a more stable analog of carbonate **11** (Figure 3A). This gain in stability came at the cost of fast ring opening, with rates for **13** still about 4× those of the acyclic control **14** (entry 14).

As explained above as the basis of the envisioned directing groups and the reactivity-mediated selectivity model (Figure 2D), varied proticities as found in biological microenvironments can affect thiolate protonation, sulfinate activation by H-bond removal, and so on. The ring-opening equilibria of CTO **3** depended indeed strongly on solvent proticity (Figures 3D and S28–S43, Table 1, entries 3–10). From the 90% deuterated phosphate buffer in DMSO-*d*_6_ used above to compare opening kinetics, decreasing D_2_O content at constant phosphate concentration caused a shift of equilibrium to the product side (Figure 3D, circles). This shift occurred because *k*^O^ increased more than *k*^C^ (= *k*^O^/*K*) (Figure 3D, diamonds). However, further decreasing D_2_O content from 25 to ≤10% shifted the equilibrium strongly to the closed form. The resulting bell-shaped dependence of the opening equilibrium of CTO **3** on protic solvents thus revealed strong acceleration of ring closure at sufficiently low water content due to more significant activation of sulfinate than thiolate exchangers (Figure 2D, **XV**, **XVI**).

**Figure 4:**
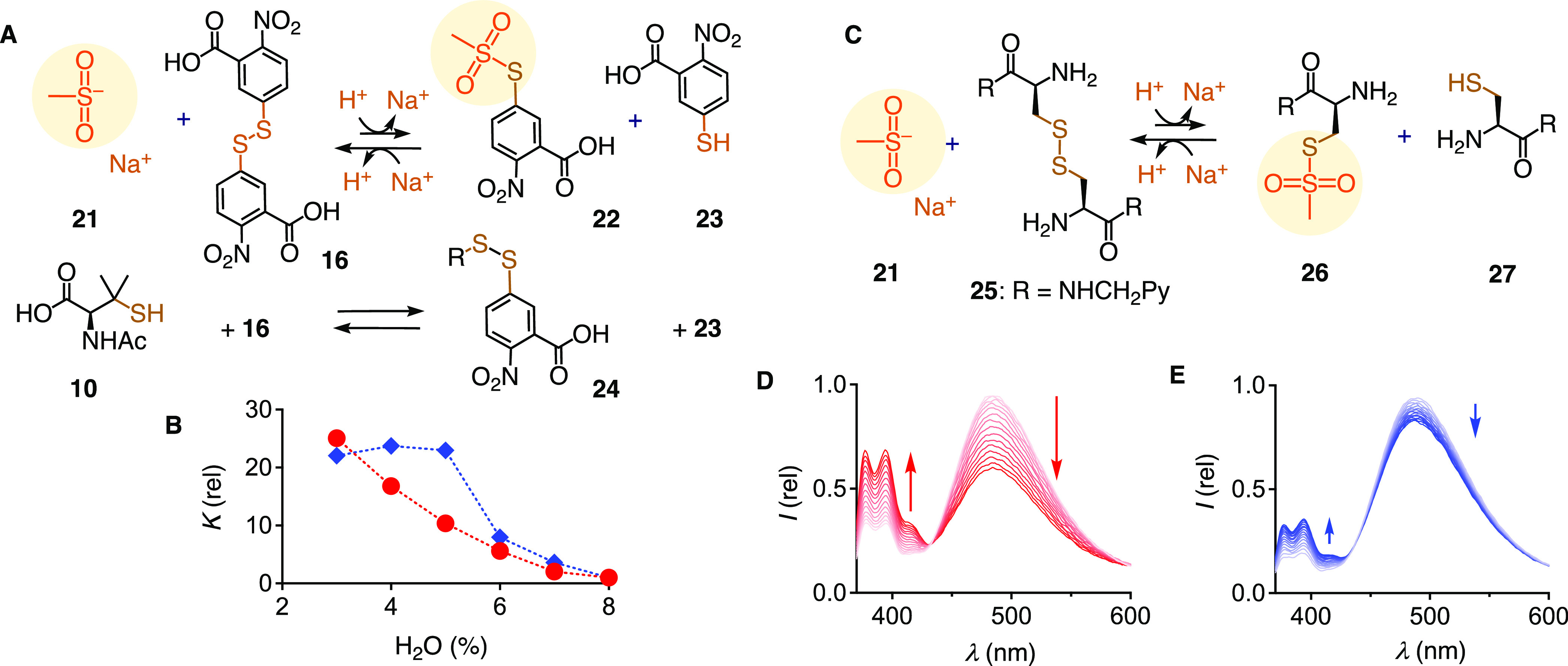
(A) Model experiments for exchange of sodium sulfinate **21** and thiol **10** with disulfide **16**. (B) Dependence of *K* for the exchange of **21** and **16** (red circles) compared to thiol **10** and **16** (blue diamonds) on the water content in DMF, normalized against *K* at 8% H_2_O (PBS, pH = 7.4). (C) Exchange of sulfinate **21** with cystine **25**; Py = pyrene. (D) Normalized emission spectra (λ_ex_ = 344 nm) measured every 6 min after the addition of **21** to **25** in DMF/PBS 95:5, pH 7.4. (E) Same in DMF/PBS 50:50.

This proticity-dependent reactivity of sulfinates was thus in support of the concept of directing groups for reactivity-based selectivity (Figure 2D). However, possible alternative interpretations of results with cascade exchanger **3** called for model studies with the minimalist sulfinate exchanger **21** (Figure 4A). The formation of thiosulfonate **22** by dynamic covalent exchange with DTNB **16** was detectable by the appearance of the absorption of conjugate thiolate base of **23** (Figure S47). Exchange became detectable only at <10% water content in DMF, buffered at pH = 7.4. Decreasing water content from 8% caused a sharp shift of equilibrium to the thiosulfonate product **22** (Figure 4B, circles). Exchange with thiol **10** shifted with decreasing water content first to the side of disulfide product **24** (Figure 4B, diamonds, S48). However, at <5% water, conditions where exchange with the anionic sulfinate **21** really turns on, exchange with the increasingly neutral thiol/ate **10** stopped accelerating. While the initially increasing exchange rate with **10** at decreasing water content was consistent with thiolate activation by its destabilization in reduced polarity,^68^ the plateau at low proticity presumably originated from the onset of thiolate protonation (Figure 4B, diamonds), which obviously does not occur as easily with the less basic sulfinate **21** (Figure 4B, circles). Overlay of the dependence of exchange equilibria with sulfinate **21** and thiol/ate **10** on solvent proticity (Figure 4B) qualitatively reproduced the bell-shaped dependence obtained with CTO **3** (Figure 3D). This good correlation was important because it supported that the findings made at pD 2.7 (Figure 3D) are valid also at pH 7.4, and because it supported that the activation of the sulfinate exchanger with decreasing proticity (Figure 2D) indeed accounts for the emergence of ring closure of opened **9** into CTO **3** (Figure 3D).

The ability of a minimalist, cascade-decoupled sulfinate exchanger like **21** to exchange with disulfides of cystine in cellular partners in thiol-mediated uptake was probed with the pyrene labeled model **25** (Figure 4C). Decrease of the excimer band in the emission spectrum of dimer **25** confirmed that sulfinate **21** exchanges with disulfides of cystine in cellular partners, affording the two pyrene monomers **26** and **27** (Figure 4D,C). The velocity of this exchange decreased with increasing solvent proticity (Figure 4E). These results thus identified CTO **3** as operational CAX for cellular disulfides, and demonstrated that dynamic covalent exchange cascades with CTOs like **3** are selective for low-proticity environments needed to activate sulfinate exchangers. This reactivity-based selectivity is absent in cyclic disulfides and, thus, a unique characteristic of CTOs (Figure 2D).

Taken together, the main findings of this section toward parametrizing characteristics of CAXs and thus thiol-mediated uptake are as follows: (1) CTOs open faster than cyclic disulfides and thiosulfinates. (2) Chalcogen bonding to chloride or fluoride anions reflects these trends by bond shortening with increasing oxidation levels. (3) Low-proticity environment causes strong activation of sulfinates compared to thiols, affecting the exchange equilibria. We hope that these trends and their functional significance will encourage comprehensive studies on this important topic.

### Transporters

**Figure 5:**
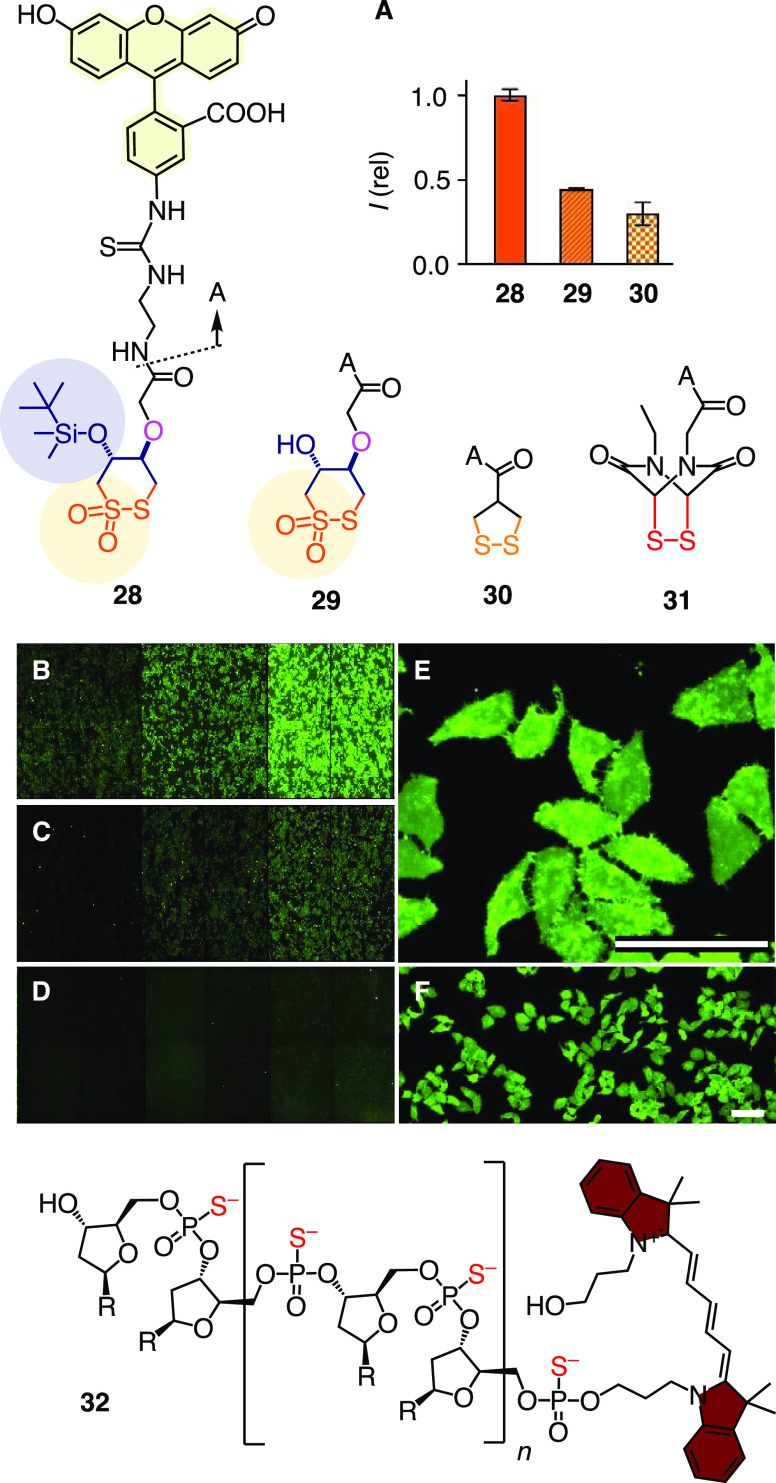
New (**28**, **29**) and old (**30**–**32**) transporters for thiol-mediated cytosolic delivery used in this study, with (A) fluorescence intensity of HK cells in SDCM images after incubation with 20 μM **28** (B), **29** (C), and **30** (D), original multiwell plates for automated transporter imaging with HK cells incubated with **28** (B), **29** (C), and **30** (D, 10, 20, 50 μM, left to right), and (E) large and (F) small magnifications of (B). Scale bars: 100 μm. Compounds **28** and **29** are regioisomeric mixtures.

To assess the cellular uptake of CTOs, the fluorescently labeled transporters **28** and **29** were prepared (Figure 5). These two lead structures were selected based on extensive screenings of cytosolic delivery and lentivector uptake inhibitors (*vide infra*). The synthesis of new transporters **28** and **29** is described in the Supporting Information (Scheme S29).

Cellular uptake was measured by automated high-content high-throughput (HCHT) imaging.^51^ This method provides access to biologically meaningful and statistically relevant data from thousands of cells within a very short time. For the uptake monitoring, HeLa Kyoto (HK) cells in multiwell plates were incubated with the fluorescent transporters **28**–**30** for 1 h, washed, and imaged by spinning disk confocal microscopy (SDCM) to quantify the fluorescent transporters that have entered the cells. Both CTOs transporters **28** and **29** were more active than the AspA **30** standard (Figure 5A–D). The better activity of CTOs **28** compared to **29** was consistent with the concept of hydrophobic directing groups, expected to guide CTOs into an aprotic environment within membranes or proteins to activate the sulfinate exchanger and thus turn on dynamic covalent exchange cascades with cellular partners (Figure 2D). The passive diffusion of transporter **28** across the membrane is conceivable but unlikely because this process can be inhibited by other CAXs (see the next section).

Consistent with the importance of dynamic covalent exchange cascades, the disulfide analog of CTO **29**, fluorescently labeled DTT/DTE **1**, has been shown previously to be less active than AspA **30** as a transporter.^38^ Intermediate thiosulfinate derivatives of **2** were not judged promising as transporters due to their much slower exchange (Figure 3) and weaker inhibition compared to CTOs (below), together with mechanistic considerations (Figure 2B).

One advantage of HCHT imaging of cellular uptake compared to flow cytometry^38,42,43^ is that it simultaneously gives information on toxicity and intracellular localization. Relative viability can be extracted by comparing the number of cells stained with Hoechst 33342 (HOE) and dead cells stained with propidium iodide (PI). Information on the intracellular localization of reporters can be quantified through segmentation of cellular compartments stained with specific markers.^52^ For CTO **28**, microscopy images revealed a major diffused staining all over the cell body, which confirmed a dominant cytosolic delivery (Figure 5E,F). The minor punctate structures could be associated with negligible endolysosomal accumulation.

Cytosolic delivery is a hallmark of thiol-mediated uptake, which accounts for its usefulness in practice.^12^ However, images of similarly even distribution in cells have been reported so far only for diselenolanes.^42^ In the absence of targeting units, AspA **30** affords more punctate features in agreement with increasing contributions from endocytosis, mostly, but not only, mediated by the transferrin receptor.^41^ The same is observed for OPS **32**, known to exchange with many different membrane proteins.^52^ Benzopolysulfanes^43^ and particularly ETP **31**(39) accumulate more in the nucleus. Although interesting, these intrinsic intracellular distributions are of minor importance because attached targeting groups can dictate the destination of transporters once in the cytosol.^78^

### Inhibitors of Cytosolic Delivery

**Figure 6:**
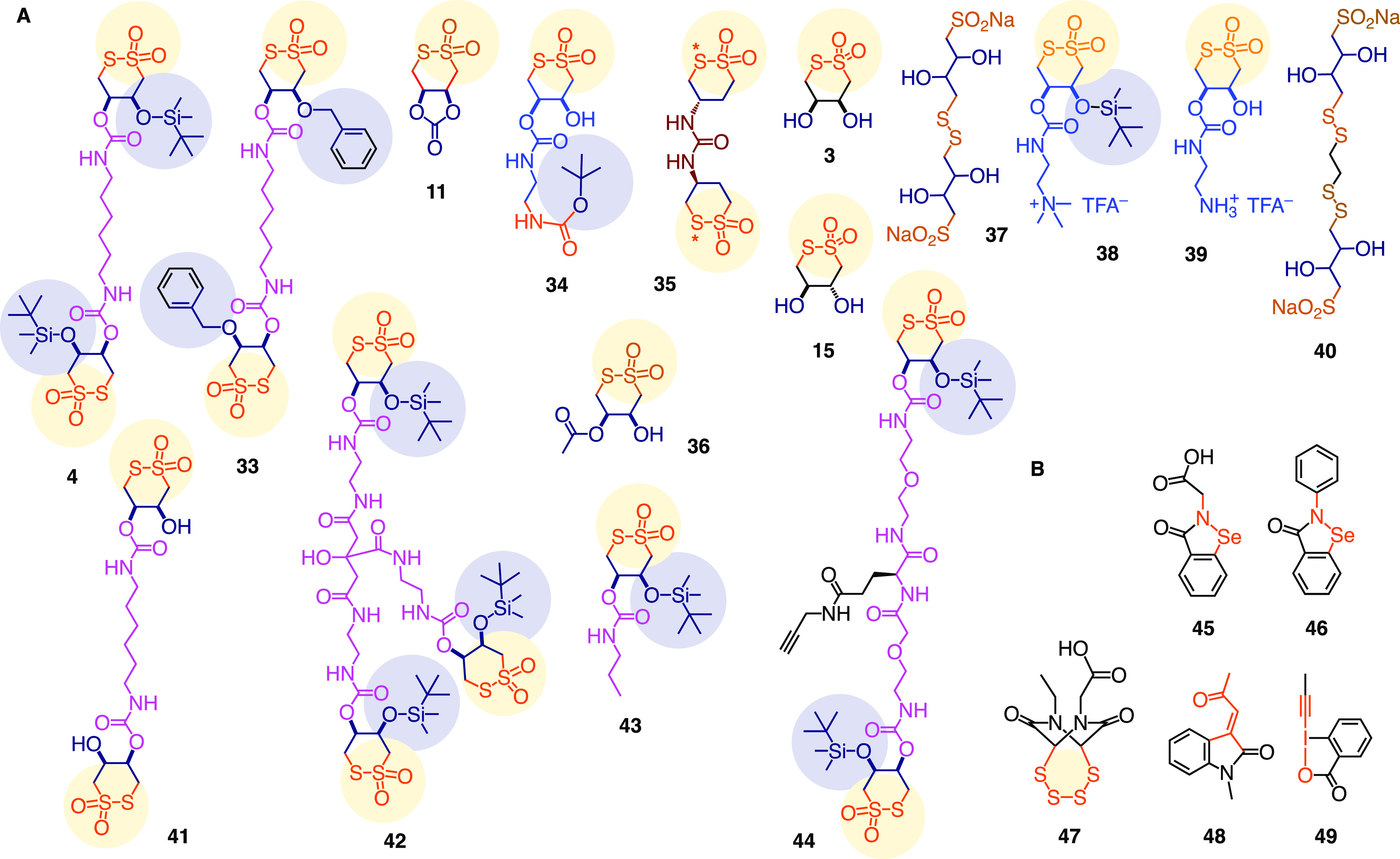
(A) Cascade exchanging inhibitor candidates **3–4**, **11**, **15**, and **33–44** and (B) controls **45–49** (Tables 1–3). *Alternative position of SO_2_.

Thiol-mediated uptake is proven by its inhibition with thiol-reactive agents, including “self-inhibition” by the same cascade exchangers (Figures 1 and 2A–C). The inhibition of CTO **28** uptake was thus explored in comparison to known transporters, that is, the fluorescently labeled ETP **31**(39) and OPS **32**(52) (Figures 5 and S49–S58; Tables 2 and S1–S11). Pertinent inhibitor candidates were selected from dynamic covalent exchangers centered around CTOs (**2**–**4**, **11**–**18**) and the lentivector entry inhibitor candidates (**33**–**44**, Figure 6A), complemented by thiol-reactive controls beyond CTOs (**45**–**49**, Figure 6B, Table 2). Many more exchangers were examined, i.e., **50**–**78**, but they were either poorly active, highly toxic, or unable to reveal new, significant trends beyond the ones described below. Their structures, synthesis, and inhibitory activities are thus duly reported in the Supporting Information.

**Table 2:** Inhibition of Cytosolic Delivery^a^

	I^b^	T^c^	cond^d^	MIC^e^ (μM)	IC_50_^f^ (μM)	RV_50_^g^ (μM)
1^h^	**2**	**31**	P	800	>5000	4400
2	**2**	**28**	P	>50	>100	>100
3^h^	**3**	**31**	P	200	>1000	1300
4	**3**	**28**	P	5	60	>1000
5^i^	**3**	**32**	P	250	550	
6	**3**	**31**	C	3	35	>1000
7	**15**	**31**	C	1.5	22	>500
8	**11**	**31**	P	1.5	9	>20
9	**11**	**31**	C	<5	>50	>50
10	**11**	**28**	C	<1	>50	>50
11	**12**	**31**	C	<1	7	>50
12	**12**	**28**	C	5	60	100
13	**4**	**28**	C	0.3	≈15	>5
14	**33**	**28**	C	1	>5	>5
15	**34**	**31**	P	3	>100	>100
16	**34**	**31**	C	16	>100	>100
17	**34**	**28**	C	1	26	>50
18	**35**	**31**	C	9	51	>100
19	**35**	**28**	C	4	25	>50
20	**45**	**31**	P	<1	10	>20
21	**45**	**31**	C	2	8	>20
22^i^	**45**	**32**	P	>100	>100	
23	**45**	**28**	P	1	8	>20
24	**36**	**31**	C	2	27	>50
25	**37**	**31**	C	7	≈60	>50
26	**38**	**31**	C	23	47	>50
27	**39**	**31**	C	14	≈60	>50
28	**40**	**31**	C	8	50	>50
29	**46**	**31**	C	<2	49	>100
30^h^	**47**	**31**	P	≤0.1	1.3	35
31	**47**	**28**	P	>10	>10	>10
32^i^	**47**	**32**	P	2	50	
33^j^	**48**	**31**	P	<1	4	>20
34^j^	**49**	**31**	P	4	40	60
35^i^	**49**	**32**	P	0.3	10	
36	**49**	**28**	P	9	53	>50
37	**41**	**28**	C	1	8	>50
38	**42**	**28**	P	2	5	22
39	**43**	**28**	P	4	>50	43
40	**44**	**28**	P			4

a From HCHT imaging of HK cells incubated first with

b inhibitors I at varied concentrations for 1 h and then with

c transporters T at the constant concentration (**28**, 5 μM; **31**, 10 μM; **32**, 0.5 or 1 μM) for 30 min, reporting fluorescence intensity of T inside intact, PI negative cells.

d Conditions: P = pre-incubation: I removed before addition of T. C = co-incubation: I not removed before addition of T.

e Concentration needed to reach 15% inhibition.

f Concentration needed to reach 50% inhibition. SEM ≤ 30%.

g Concentration needed to lower relative viability (RV) by 50%. RV: number of PI negative cells divided by HOE-positive cells. SEM ≤ 30%.

h Data from ref (51).

i Data from ref (52).

j Data from ref (79).

**Figure 7:**
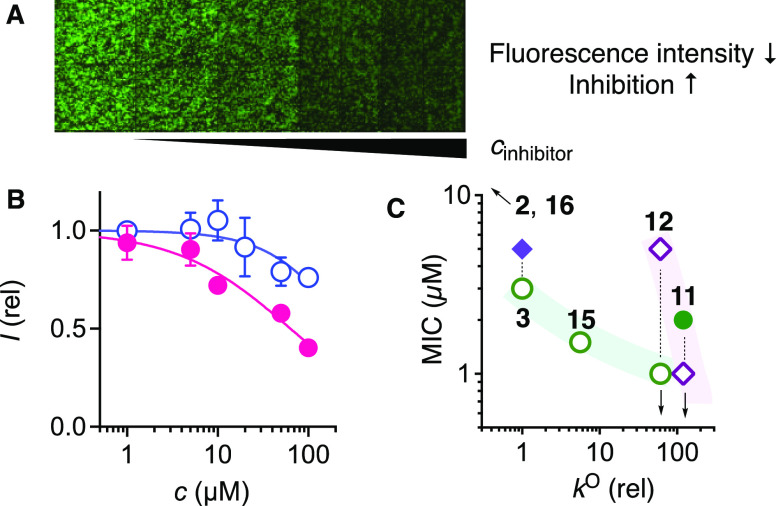
(A) Multiwell plate for automated inhibitor screening with HK cells incubated first with inhibitor **3** (0, 10, 20, 50, 100 μM, left to right) and then with CTO transporter **28** (constant concentration). (B) Dose–response curve from automated screening for uptake inhibition of CTO **28** by cyclic thiosulfinate **2** (empty circles) and CTO **3** (filled circles). (C) Comparison of the relative ring-opening rates (*k*^O^) and MICs of transporters (green circles: **31**; purple diamonds: **28**) under co-incubation (empty symbols) or pre-incubation conditions (filled symbols). Downward arrows indicate the MICs to be lower.

Uptake inhibition was measured by the automated HCHT imaging used already to elaborate on cytosolic delivery. For inhibitor screening, HK cells in multiwell plates were incubated with increasing concentrations of inhibitors, followed by fluorescent transporters **28**, **31**, and **32** at a constant concentration. After the renewal of extracellular medium and addition of HOE and PI, the dose response of transporter fluorescence was recorded and analyzed only of PI negative, live cells to yield MIC at ∼15% inhibition and, if accessible, IC_50_ at 50% inhibition (Figure 7A,B, Table 2). If inhibitors are removed before the addition of transporters **28**, **31**, and **32**, the method is referred to as “pre-incubation”. This is of interest to minimize exchange between the transporter and the inhibitor but is often problematic because dynamic covalent exchange between the inhibitor and cellular target can also be reversed and thus lost. The complementary “co-incubation” operates without inhibitor removal before the addition of transporters **28**, **31**, and **32**. In this case, apparent inhibition might arise from the inhibitor exchanging with transporter instead of cellular thiols. However, this is less likely to occur without ring opening by a thiol (Figure 2A–C), and ring-opened transporters remain dynamic to preserve partial and possibly regain full activity, depending on conditions (Figure 3B–D). Nevertheless, such contributions cannot be fully excluded. Ring-opening polymerization^12^ of transporters and inhibitors are not likely to take place under these conditions because compared to known polymerization conditions of cyclic disulfides, concentrations are too low and ring back-closure too favorable. Presumably irrelevant for uptake inhibition, we however like to point out that controlled ring-opening polymerization of CTOs, ETPs, and other new CAXs should be of general interest in the future, particularly for dynamer materials science applications.^12,21−23^

Within the monomeric redox series, the inhibitory activities related well to the ring-opening rates (Figure 7C). Increasing inhibition of ETP **31** uptake from both S–O stereoisomers of cyclic thiosulfinate **2** to the cyclic thiosulfonate **3** was previously reported^51^ and in agreement with 1000× faster and chemically distinct exchange cascades (Table 2, entries 1, 3; Figure 2B,C). The same trend was found for the new CTO transporter **28**: CTO **3** inhibited uptake clearly better than the less reactive cyclic thiosulfinate **2** (Figure 7B, Table 2, entries 2, 4). Under pre-incubation conditions, CTO **3** inhibited the uptake much more efficiently of CTO **28** than of ETP **31** and OPS **32** (entries 3–5). Against ETP **31**, a much lower MIC was obtained under co-incubation conditions (entries 3, 6). The difference between pre- and co-incubation suggested that the dynamic covalent inhibition of the cellular partners in the uptake of ETP **31** is easily reversed during the exchange of the media applied to remove unreacted and rapidly releasable CTO **3**. Consistent with this interpretation, the faster DTT-derived *trans* diastereomer **15** was slightly more active under co-incubation conditions (entry 7; Figure 7C).

Acceleration of dynamic covalent exchange with the cyclic carbonate **11** further enhanced inhibition of both ETP **31** and CTO **28** (entries 8–10). With the decarboxylation product **12**, inhibition of ETP **31** exceeded CTO **28**, and the high activity was conserved (entries 11, 12). For the inhibition of **31** under co-incubation conditions, increasing opening rates perfectly correlated with increasing inhibition for **12** > **15** > **3** (Figure 7C, empty circles). The same correlation was observed for the inhibition of CTO **28** by **11** > **12** under co-incubation conditions (Figure 7C, empty diamonds).

Monomeric and dimeric CTOs **4** and **33**–**44** reliably inhibited the uptake of ETP **31** and CTO **28** with significant variations. With increasing hydrophobicity and bolaamphiphilicity, detection of the activity at higher concentrations could become problematic due to inhibitor self-assembly and precipitation. This was also true for the most important dimers **4** and **33**, which were inactive at >10 μM. However, focused re-evaluation at low concentrations under co-incubation conditions revealed dimers **4** and **33** among the best inhibitors of the cytosolic delivery with transporter **28** (entries 13, 14), which was consistent with their ability to inhibit the entry of SARS-CoV-2 (SC2) lentivector best (*vide infra*).

**Figure 8:**
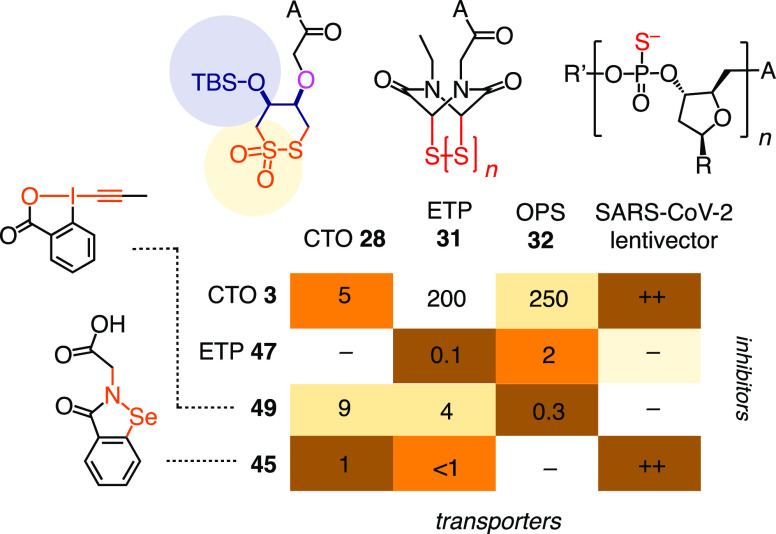
Minimalist heatmap for transporters **28**, **31**, **32**, and SC2 and inhibitors **3**, **45**, **47**, and **49** (with MICs in μM and qualitative inhibition of SC2 uptake, from Tables 2 and 3). −: inactive, R, R′, A: Figure 5. For transporter inhibition, data used are for pre-incubation conditions.

Ebselen **46**, a reported SC2 antiviral,^80−82^ efficiently inhibited the cytosolic delivery of ETP **31** (entry 29). The better soluble, more amphiphilic ebselen analog **45** was even more powerful (entries 20, 21). The amphiphilic ebselen **45** also inhibited the cytosolic delivery of CTO **28** but not OPS **32** (entries 22, 23, Figure 8). OPS **32** was best inhibited by the irreversible hypervalent iodine reagent **49** (entry 35, Figure 8).^52,79^ Like CTOs “self-inhibiting” CTO **28**, the cytosolic delivery of ETP **31** was best self-inhibited with ETPs, with the expanded tetrasulfide **47** being most interesting (entry 30). The resulting heatmap showed strong contrasts, powerful self-inhibition, and different top inhibitors for all transporters (Figure 8). The orthogonal selectivities for inhibiting CTO, OPS, and ETP transporters supported the emerging concept of thiol-mediated uptake as a complex dynamic covalent network involving different pathways with multiple cellular partners. Compared to lentivector uptake inhibition (*vide infra*), CTO **28** correlated best, whereas OPS **32** was perfectly orthogonal (Figure 8). Inhibition of CTO **28** could thus possibly become best to predict lentivector uptake inhibition.

Taken together, the inhibition of CTO **28** uptake by a wide variety of CAX implies that it enters the cytosol through thiol-mediated uptake, while passive diffusion can be excluded at this point. CTOs with faster exchange kinetics give better MICs. With other factors such as self-assembly and precipitation contributing to results and producing unconventional dose response, inactivity of inhibitor candidates should not be overinterpreted. It could only mean that the right conditions to observe inhibition have not been found. This is particularly true for hydrophobic, amphiphilic, and bolaamphiphilic inhibitors. What matters for interpretation are positive results for successful inhibition.

### Inhibitors of Lentivector Uptake

**Figure 9:**
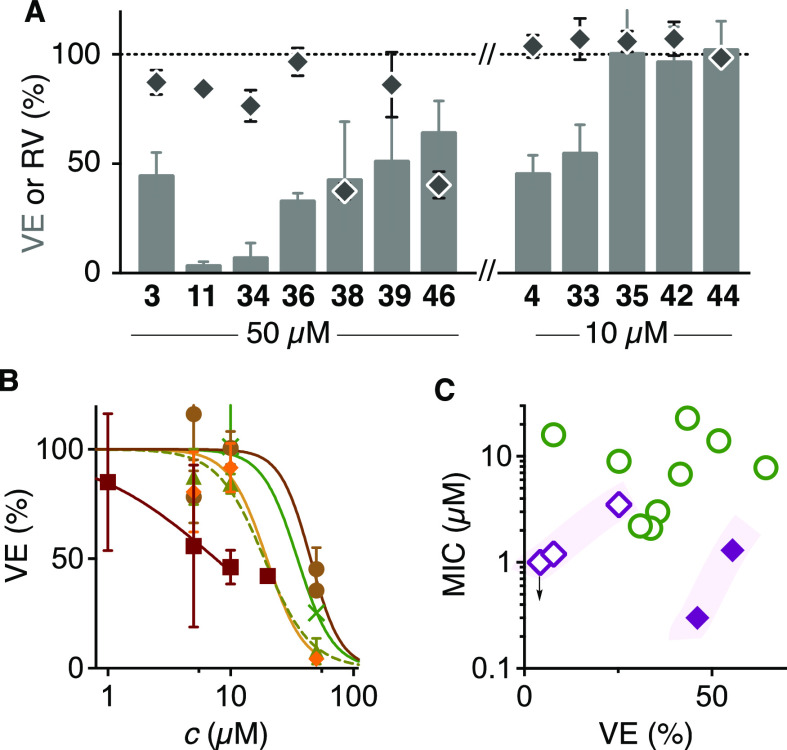
(A) Normalized VE (gray columns) and RV (filled diamonds) upon treatment with monomers at 50 μM and dimers at 10 μM. (B) Normalized dose–response curves of VE inhibition by **3** (brown circles), **4** (maroon squares), **11** (orange diamonds), **34** (olive triangles), and **35** (green crosses). (C) Comparison of VE (filled symbols: 10 μM inhibitor; empty symbols: 50 μM inhibitor) and the MICs of transporter entry under co-incubation conditions (green circles: ETP **31**; purple diamonds: CTO **28**).

To elucidate the potential of CTOs to inhibit viral entry, they were incubated with A549 human lung alveolar basal epithelial cells overexpressing ACE2 and TMPRSS2^83^ for 1 h. Then, incubation was continued for 6 h in the presence of lentivirus expressing the SC2 spike protein with D614G mutation and coding for a luciferase reporter (Tables 3 and S12). Earlier, we reported the ≈60% inhibition of luciferase expression, thus SC2 lentivector entry (VE), by 50 μM CTO **3** (Table 3, entry 8).^51^ At 10 μM, however, CTO **3** was inactive (Figure 9A,B, circles). Consistent with much higher reactivity (Figure 3A), carbonate **11** was more active at 50 and 10 μM (entry 3). Hill analysis of these dose responses (Figure 9B, circles vs diamonds) indicated that **11** is about twice as active as **3**, and the activities are cooperative (Hill coefficient *n* ≈ 3), i.e., multiple exchanges are needed to inhibit the vector entry. Although meaningful, these implications are based on a few data points and thus should not be overinterpreted. Carbonate **11** was not further pursued because high reactivity coincided with poor stability (Figure 3A,J), and **34** showed similarly high activity without stability issues (entry 4, Figure 9B, triangles). Compared to **34**, acylated and cationic CTOs **36**, **38**, and **39** were less active, and **38** also suffered from the onset of toxicity, possibly due to its amphiphilicity (entries 7, 10, 11, Figure 9A). These results agreed with the concept of aproticity-enhanced reactivity (Figures 2D and 3D).

**Table 3:** Inhibition of SC2 Lentivector Entry^a^

	I^b^	*c* (μM)^c^	VE (%)^d^	RV (%)^e^
1	**4**	10	46 ± 8	103
2	**33**	10	56 ± 12	107
3	**11**	50	4 ± 1	84
4	**34**	50	8 ± 6	76
5	**35**	50	25 ± 3	96^f^
6	**45**	50	31 ± 3	81
7	**36**	50	34 ± 3	97
8	**3**	50	36 ± 3	81
9	**37**	50	42 ± 32	97^f^
10	**38**	50	43 ± 26	37
11	**39**	50	52 ± 31	86^f^
12	**40**	50	64 ± 25	96^f^
13	**46**	50	65 ± 13	40^f^
14	**47**	50	83 ± 12	70
15	**41**	5	>100^f^	100
16	**48**	5	>100	94
17	**49**	5	>100	92
18	**16**	5	>100	89
19	**42**	10	97 ± 15	107
20	**43**	10	99 ± 12	87
21	**44**	10	103 ± 12	98

a From normalized luminescence intensity of A549 human lung alveolar basal epithelium cells overexpressing ACE2 and TMPRSS2 after incubation with

b inhibitor candidates at

c concentrations *c* (1 h) and lentivirus with D614G SC2 spike protein (6 h), followed by 3 days for luciferase expression, reported as

d vector entry (VE) in % (*n* = 3, ±SD).

e RV: Relative cell viability. SD ≤ 10%.

f SD > 10%.

As anticipated from the cooperativity discussed above, multivalent inhibitors were proven more powerful (Figure 2E). CTO **4** inhibited SC2 lentivector entry with an IC_50_ ∼10 μM (entry 1, Figure 9B, squares). Less cooperative dose response implied that **4** occupies multiple binding sites of target proteins through both CTOs to elicit its inhibitory activity. Higher concentrations beyond 10 μM did not lead to increased inhibition because of the onset of self-assembly of the bolaamphiphile **4** in this assay, perhaps followed by precipitation and/or ring-opening polymerization. This high concentration effect was as previously observed in the screening of thiol-mediated uptake inhibitors, also with the popular Ellman reagent **16**.^51,79^

The de-silylated dimer **41** was inactive at 5 μM (entry 15), i.e., around the IC_50_ of dimer **4** (entry 1, Figure 9A). This result echoed the poorer activity of transporter **29** compared to **28** (Figure 7). Both comparisons supported the concept of hydrophobic directing groups to exchange with cellular thiols in hydrophobic pockets and shield the released sulfinate from inactivation by hydrogen bonding (Figures 2D, 3D, and 4B). Consistent with this interpretation, replacement of the TBS groups with benzyls in **33** was tolerated with negligible losses in activity at 10 μM (entry 2, Figure 9A). However, further structural modifications were not accepted, supporting the involvement of quite specific molecular recognition. This included, disappointingly, trimer **42**, dimer **44** with a central alkyne for further derivatization, and dimer **35** with a shortened spacer (entries 5, 19, 21, Figure 9A). The steep dose response of the urea dimer **35** with high activity at 50 μM and inactivity at 10 μM suggested that its short linker hinders the divalent binding to cellular targets (Figure 9B, crosses). The inactivity of monomer **43** further supported that both CTOs in dimers **4** and **33** are necessary and that simple partitioning effects are not decisive for activity (entry 20, Figure 2E).

Among other types of inhibitors tested, ring-opened CTOs **37** and **40** showed activities similar to the original **3**, suggesting that these linear compounds are in equilibrium with CTOs, which are the active form (entries 9, 12). Ebselen **46**, an SC2 antiviral candidate,^80−82^ was at 50 μM more toxic than active as an inhibitor of SC2 lentivector entry (entry 13, Figure 9A). The more hydrophilic, anionic ebselen analogue **45** was more active and nontoxic at 50 μM (entry 6). The hypervalent iodine reagent **49** is an irreversible covalent thiol-reactive agent, and was the most active inhibitor of cytosolic delivery of OPS **32** but less powerful against ETP **31**(79) and particularly CTO **28** (Figure 8). The covalent reagent **49** did not inhibit SC2 entry at 5 μM, i.e., around the IC_50_ of dimers **4** and **33** (entry 17). It even slightly enhanced SC2 entry, which is not an unusual phenomenon associated with membrane disturbing activity at the onset of cytotoxicity. The inactivity of the irreversible covalent thiol-reactive agent **49** could further support the importance of exchange cascades to inhibit SC2 lentivector entry and the existence of orthogonal exchange network coding for thiol-mediated uptake (Figure 8).

Similar trends were found for super-cinnamaldehyde **48** (Figure 6). Introduced to react with thiols in the pain receptor TRPA1,^84^ super-cinnamaldehyde **48** was identified as one of the most efficient inhibitors of the cytosolic delivery with ETP **31** (Table 2, entry 33).^52,79^ For SC2 vector entry, weak activation rather than inhibition was found at 5 μM (Table 3, entry 16). Also poorly active was ETP-S_4_**47**, the other most potent inhibitor of ETP **31** and also OPS **32** entry (Figure 8, Table 3, entry 14). Ellman’s reagent **16**, finally, has been confirmed previously as a very poor inhibitor of cytosolic delivery by thiol-mediated uptake with all tested transporters^51^ but early on proposed as an inhibitor of thiol-mediated entry of HIV.^61^ For SC2 entry, the result was as for controls **48** and **49**, that is weak activation rather than inhibition at 5 μM (entry 18). Thus overall, inhibitory activities against vector entry correlated well with that against CTO transporter **28**, but poorly against ETP transporter **31** (Figures 8 and 9C). Taken together, the inactive controls **16**, **47**–**49** all supported the potential of CTOs, particularly dimers, to inhibit SC2 lentivector entry as well as the importance of exchange cascades and hydrophobic directing groups for this activity.

Many active compounds in thiol-mediated uptake as transporters or inhibitors are also known to act on other sites. Like paxlovid,^85^ ebselen is a cysteine protease inhibitor^82^ while CTOs have been associated with EGFR and zinc fingers.^49,86^ Preliminary results indicated that dimer **4** at 10 μM inhibits trypsin by >80% but neither cathepsin L nor B; at 50 μM, **3** inhibits trypsin and cathepsin B but not L, **12**, **34**, and **35** inhibit only cathepsin B (not shown).

Ultimately, multitarget activity^80^ to a certain extent will be common to all dynamic covalent inhibitor candidates tested. However, the objective of this study was not to identify alternative targets but to elaborate on the correlation of the inhibition of SC2 lentivector entry on the one hand and thiol-mediated cytosolic delivery on the other. A high number of compounds capable of doing both has been identified. The future will tell if this is a coincidence or more. Thiol-mediated uptake has so far received little attention with regard to SC2 entry. However, possibly thiol-mediated processes in SC fusion have been reported,^87,88^ and many membrane proteins have been associated with both processes, most notably the TfR and Scarb1.^12,41,89,90^ The identification of top activities for CTO dimers argues against enzyme inhibition and in favor of dynamic covalent exchange cascades with more than one partner as mode of action. A heatmap with different inhibitors for different transporters (Figure 8) supports this emerging view of thiol-mediated uptake as a complex network of general significance for cellular entry and indicates that CTO transporters and SC2 might operate with the same cellular partners.

## Conclusions

The objective of this study was to shift attention in thiol-mediated uptake from disulfides to higher oxidation levels and introduce cyclic thiosulfonates (CTOs) as well as to use the new cascade exchangers (CAXs) to outline the unique chemical space accounting for thiol-mediated uptake. The new CTOs are confirmed to act as transporters for cytosolic delivery, inhibitors of the cytosolic delivery by thiol-mediated uptake, including self-inhibition, and inhibitors of SC2 lentivector entry. The most active transporters **28** are CTOs equipped with hydrophobic directing groups. The most active inhibitors **4** and **33** are CTO dimers with the same hydrophobic directing groups, followed by CTO monomer **11** with enhanced reactivity.

In the light of these results, the most important lesson learned is that CTO opening is fast and the resulting tethered sulfinates continue to exchange with disulfides, preferably in aprotic environments. This reactivity-based selectivity of CTOs called for the concept of hydrophobic directing groups to bring the CTOs to the reactive disulfides. This concept is unique among the CAXs known so far and should be of interest also in the materials sciences.

The second lesson learned is that with the development of CTOs as new CAXs, the number of dual inhibitors for cytosolic delivery of thiol-mediated uptake and SC2 entry increases significantly. The top activity of dimers with directing groups, i.e., **4** and **33**, supports the involvement of dynamic covalent exchange cascades.

The third lesson learned concerns the comparison of different transporters with different inhibitors. The introduced minimalist heatmap shows different inhibitors as best with different transporters. This finding supports the emerging view of thiol-mediated uptake as a general complex network encoding for more than one cellular partner and pathway to bring matter into cells. A better understanding of this dynamic covalent circuitry accounting for thiol-mediated uptake in the broadest sense emerges as the most important and most demanding future challenge. The introduction of both more CAXs and engineered cells for pattern generation will likely be crucial for progress in this direction.

Why is thiol-mediated uptake so useful in practice but so poorly understood? Reasons might include the complexity and the dynamic nature of the systems at work, and the large underexplored chemical space covered. With CTOs as examples for newly emerging CAXs, we aimed here to create awareness for this unique chemical space and outline as many possible intertopical connectors as possible. Realized trends correlate computed chalcogen bond length and ring-opening rates with uptake inhibition efficiency (Figures 3A,E–I and 7C). As another example, the concept of proticity control of CTOs is consistently reflected in cascade exchange equilibria, cytosolic delivery, and the inhibition of lentivector entry (Figures 2D, 3D, 4B, 5A–C, and 9). Considering the intertopical correlations between complex systems involved, the three top inhibitors **4**, **33**, and **11** of lentivector uptake are overall remarkably well rationalized by exchange velocity, directing groups, and divalency. Nevertheless, not all intertopical correlations match that well, as expected for the diverse parameters contributing to biological function. It is thus understood that, despite all consistency, most intertopical correlations found could ultimately turn out to be just coincidences. In any case, the generally meaningful trends identified by these interconnections support central concepts, enable new ones (e.g., reactivity-mediated selectivity), and provide transporters **28** and inhibitors **4** and **33** with outstanding activity. Their likely relevance will, hopefully, stimulate in-depth studies on the individual topics involved and help direct future efforts to ultimately crack thiol-mediated uptake.

## Supporting Information Available

Detailed experimental procedures, material and methods, compound synthesis and characterization, original ^1^H and ^13^C NMR spectra, time-dependent ^1^H NMR and absorption spectra and kinetics curves for cascade exchange, procedures for cell culture and HCHT imaging, dose–response curves and data for the cytosolic delivery of **28** and **31** and its inhibition, procedures for SC2 lentivector entry inhibition, and computational data (PDF)
