# Supplementary material for: Cyclic Thiosulfonates for Thiol-Mediated Uptake: Cascade Exchangers, Transporters, Inhibitors
Source: JACS Au. 2022 Mar 22;2(4):839–52. doi: 10.1021/jacsau.1c00573 (PMC9088311; doi:10.1021/jacsau.1c00573)
Supplement: Supplementary file 1 — au1c00573_si_001.pdf [file au1c00573_si_001.pdf]

Supporting Information

Cyclic Thiosulfonates for Thiol-Mediated Uptake: Cascade Exchangers, Transporters, Inhibitors

Takehiro Kato, Bumhee Lim, Yangyang Cheng, Anh-Tuan Pham, John Maynard, Dimitri Moreau, Amalia I. Poblador-Bahamonde, Naomi Sakai, and Stefan Matile*

Department of Organic Chemistry, University of Geneva, 1211 Geneva, Switzerland

*stefan.matile@unige.ch

Table of Contents

1. Materials and Methods S4

2. Synthesis of Inhibitors and Transporters S7

  2.1. Synthesis of Mono-Thiosulfonates S8

    2.1.1. Synthesis of DTE-Thiosulfonate Derivatives S8

    2.1.2. Synthesis of Amino-Substituted Cyclic Thiosulfonates S22

    2.1.3. Synthesis of Non-Substituted Cyclic Thiosulfonates S27

  2.2. Synthesis of Bis-Thiosulfonates S38

    2.2.1. Synthesis of DTE-Thiosulfonate Derivatives S38

    2.2.2. Synthesis of Amino-Substituted Cyclic Thiosulfonates S50

  2.3. Synthesis of Tris-Thiosulfonates S51

  2.4. Synthesis of Disulfinates and Other Inhibitor Candidates S53

  2.5. Synthesis of Transporters S56

3. Dynamic Covalent Cascade Exchange Kinetics S61

  3.1. ^{1}H NMR Spectroscopy S61

    3.1.1. Dynamic Covalent Ring Opening with Thiols S63

    3.1.2. Dependence of Ring Opening on Proticity S77

    3.1.3. Desulfinative Ring Opening of Cyclic Carbonate **11** S86

  3.2. UV-Vis Spectroscopy S87

    3.2.1. Determination of Extinction Coefficients S87

    3.2.2. Exchange of Sulfinates with Disulfides S89

    3.2.3. Exchange of Thiols with Disulfides S90

  3.3. Fluorescence Spectroscopy S91

4. High-Content High-Throughput (HCHT) Inhibitor Screening S92

4.1. Cell Culture S92

4.2. Cellular Uptake Experiments S92

4.3. HCHT Inhibitor Screening S93

4.4. Data Analysis for HCHT Screening S94

4.5. Results of HCHT Inhibitor Screening S96

5. Inhibition of Lentiviral Vector Entry S117

6. Computational Studies S119

7. Supporting References S120

8. Computational Data S125

9. NMR Spectra S134

1. Materials and Methods

As in ref. S1. Briefly, reagents for synthesis and commercially available final compounds were purchased from Sigma-Aldrich, Brunschwig, Alfa Aesar, Merck, TCI, Acros, and Iris Biotech. Phosphate buffered saline (PBS, pH = 7.4), DMEM (GlutaMAX, 4.5 g/L D-glucose, with phenol red) medium, FluoroBrite DMEM (high D-Glucose) medium, Penicillin-Streptomycin, Fetal Bovine Serum, TrypLE Express Enzyme and V96-MicroWell platen were obtained from Thermo Fisher Scientific. μ-Plate 96-Well Black were obtained from Ibidi. Hoechst 33342 (HOE, 10 mg/mL solution in water) and propidium iodide (PI, 1.0 mg/mL solution in water) were obtained from Invitrogen by Thermo Fisher Scientific.

Column chromatography was carried out on silica gel (SiliaFlash® P60, SILICYCLE, 230 – 400 mesh). Analytical thin layer chromatography (TLC) was performed on silica gel 60 F_{254} (Merck). Reverse phase flash chromatography (column: SNAP Ultra C18) and hydrophilic interaction chromatography (column: BGB Scorpius Diol column) were performed on Biotage Isolera™ Four (eluents: CH_3CN and H_2O with 0.1% TFA). Preparative HPLC was performed using JASCO LC-2000 Plus system equipped with quaternary pump (JASCO PU-2089) and UV/Vis detector (JASCO UV-2077 Plus). pH values were measured with a Consort C832 multi-parameter analyzer equipped with a VWR glass membrane pH electrode calibrated with Titrisol solution from Merck at pH 4.00, 7.00 and 10.01. pD values were obtained by the following correction; pD = pH + 0.41. UV-Vis spectra were recorded on a JASCO V-650 spectrophotometer equipped with a stirrer and a temperature controller (25 ºC). Fluorescence measurements were performed using a FluoroMax-4 spectrofluorometer (Horiba Scientific) equipped with a stirrer and a temperature controller. All fluorescence spectra were background subtracted and corrected using correction factors supplied by the manufacturer. Melting points (Mp) were measured on a Melting Point M-565 (BUCHI). IR spectra were recorded on a Perkin Elmer Spectrum 100 FT-IR spectrometer (ATR, Golden Gate, unless stated) and are reported

as wavenumbers v in cm^{-1} with band intensities indicated as br (broad), s (strong), m (medium), w (weak). ^{1}H NMR spectra were recorded (as indicated) either on a Bruker 300, 400 or 500 MHz spectrometer and are reported as chemical shifts (δ) in ppm relative to the internal $CHCl_3$ signal at 7.26 ppm, the internal CHD_2OD signal at 3.30 ppm or the internal $D_3CSOCHD_2$ signal at 2.50 ppm as the standard. Spin multiplicities are reported as a singlet (s), doublet (d), triplet (t) and quartet (q) with coupling constants (J) given in Hz, or multiplet (m). Broad peaks and apparent couplings are marked as br and app, respectively. ^{13}C NMR spectra were recorded (as indicated) either on a Bruker 75, 101 or 126 MHz spectrometer and are reported as chemical shifts (δ) in ppm relative to the internal $CDCl_3$ signal at 77.16 ppm, the internal CD_3OD signal at 49.00 ppm or the internal D_3CSOCD_3 signal at 39.52 ppm as the standard. ^{1}H and ^{13}C resonances were assigned with the aid of additional information from 1D and 2D NMR spectra (H,H-COSY, H,H-TOCSY, DEPT 135, HSQC and HMBC). ESI-MS analyses for the characterization of new compounds were performed on an ESI API 150EX. APCI-MS was measured on Biotage IsoleraTM Dalton 2000 system with APCI detector. MALDI MS analyses for the characterization of new compounds were performed with DCTB as a matrix using Bruker MALDI Autoflex Speed TOF/TOF. HR ESI-MS analyses for the characterization of new compounds were performed on Xevo G2-S Tof (Waters). HPLC-MS analyses were recorded using a Thermo Scientific Accela HPLC equipped with a Thermo C18 (5 cm x 2.1 mm, 1.9 µm particles) Hypersil gold column coupled with an LCQ Fleet three-dimensional ion trap mass spectrometer (ESI, Thermo Scientific) with a linear elution gradient from 95% H_2O / 5% CH_3CN + 0.1% TFA to 10% H_2O / 90% CH_3CN + 0.1% TFA in 4.0 min at a flow rate of 0.75 mL/min. Retention times (R_t) are reported in minutes. All mass data are reported as mass-per-charge ratio m/z ([assignment]). Fluorescence cellular imaging was performed using an IXM-C automated microscope from ImageXpress equipped with a Lumencor Aura III with 5 independently selectable solid-state light sources, bandpass filters and 5 objectives (4x to 60x).

Sample preparation and washing on μ-Plate 96-Well Black was performed using a Plate washer Biotek EL406®. In the structures of chemical compounds, bold bond and hashed bold bond were used to indicate relative stereochemistries, whereas wedged bond and hashed wedged bond were used to indicate absolute stereochemistries.

Abbreviations. Ac: Acetyl; ACE2: Angiotensin-converting enzyme 2; APCI: Atmospheric-pressure chemical ionization; aq.: Aqueous; Bn: Benzyl; Boc: *tert*-Butoxycarbonyl; Bu: Butyl; DCTB: {(2*E*)-2-Methyl-3-[4-(2-methyl-2-propanyl)phenyl]-2-propen-1-ylidene}malononitrile; DIAD: Diisopropyl azodicarboxylate; DMAP: 4-(Dimethylamino)pyridine; DMEM: Dulbecco's modified eagle medium; DMF: *N*,*N*-Dimethylformamide; DMSO: Dimethyl sulfoxide; DTE: 1,4-Dithioerythritol; DTNB: 5,5'-Dithiobis(2-nitrobenzoic acid); DTT: 1,4-Dithio-DL-threitol; EDCI: 1-Ethyl-3-(3-dimethylaminopropyl)carbodiimide; ESI: Electrospray ionization; FBS: Fetal bovine serum; FITC: Fluorescein isothiocyanate; HATU: 1-[Bis(dimethylamino)methylene]-1*H*-1,2,3-triazolo[4,5-*b*]pyridinium 3-oxide hexafluorophosphate); HCHT: High-content high-throughput; HEK cells: Human embryonic kidney cells; HOE: Hoechst 33342; *i*-: *iso*-; IC_{50}: Half maximal inhibitory concentration; MALDI: Matrix-assisted laser disorption/ionization; *m*-CPBA: 3-Chloroperoxybenzoic acid; MICs: Minimum inhibitory concentrations; Mp: Melting point; *n*-: *normal*-; NHS: *N*-Hydroxysuccinimide; *p*-: *para*-; PBS: Phosphate buffer saline; PI: Propidium iodide; Pr: Propyl; quant.: Quantitative yield; R_f: Retardation factor; rt: Room temperature; RV: Relative viability; sat.: Saturated; SC2: SARS-CoV-2; SDCM: Spinning disk confocal microscopy; SD: Standard deviation; SE: Standard error; SEM: Standard error of mean; *t*-: *tert*-; Tf: Trifluoromethanesulfonyl; TFA: Trifluoroacetic acid/trifluoroacetate; THF: Tetrahydrofuran; TMPRSS2: Transmembrane serine protease 2; TNB: 2-Nitro-5-thiobenzoic acid; Ts: 4-Toluenesulfonyl.

2. Synthesis of Inhibitors and Transporters

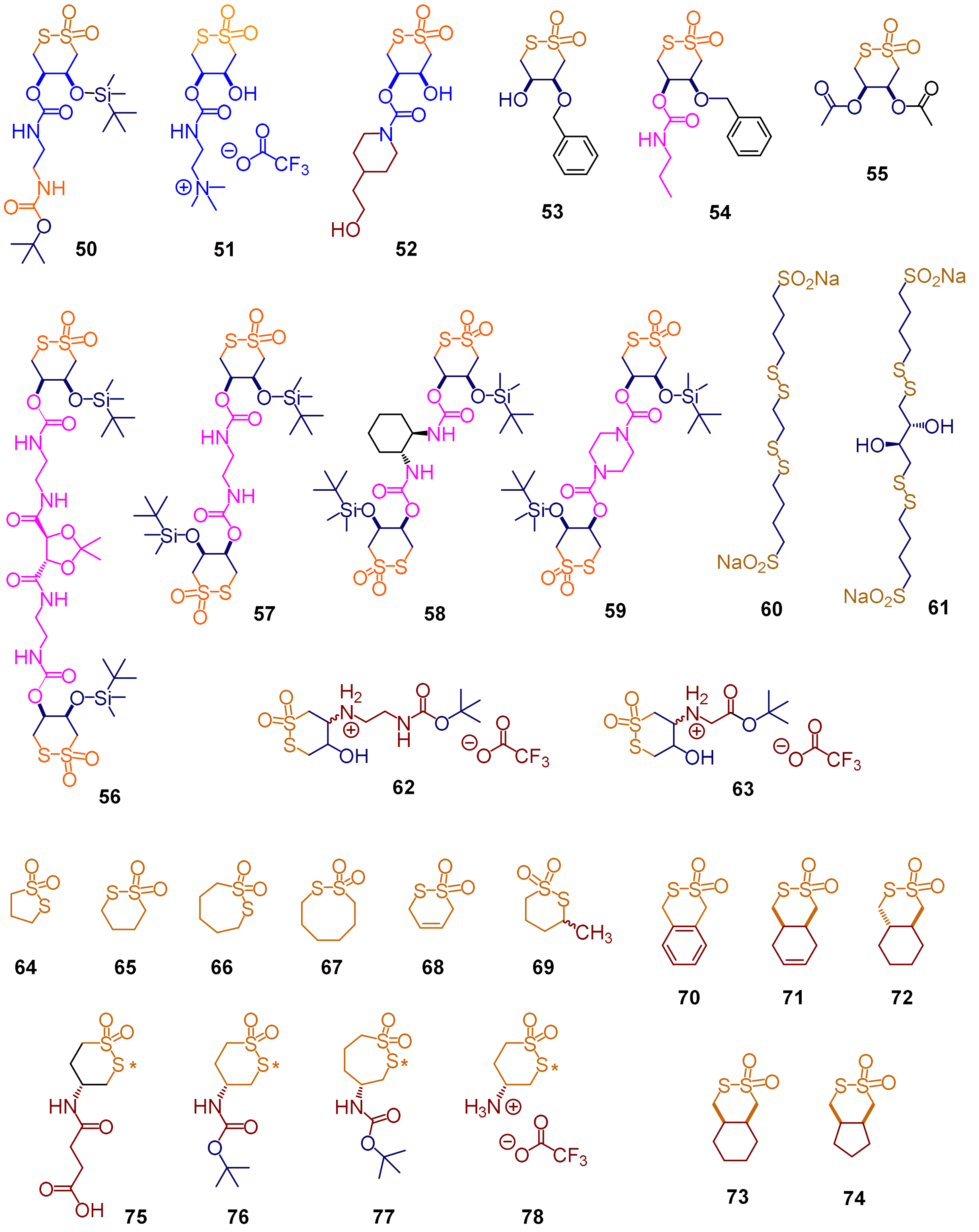

Figure S1. Structure of synthetic inhibitor and antiviral candidates. *Alternative position of SO_2.

2.1. Synthesis of Mono-Thiosulfonates

2.1.1. Synthesis of DTE-Thiosulfonate Derivatives

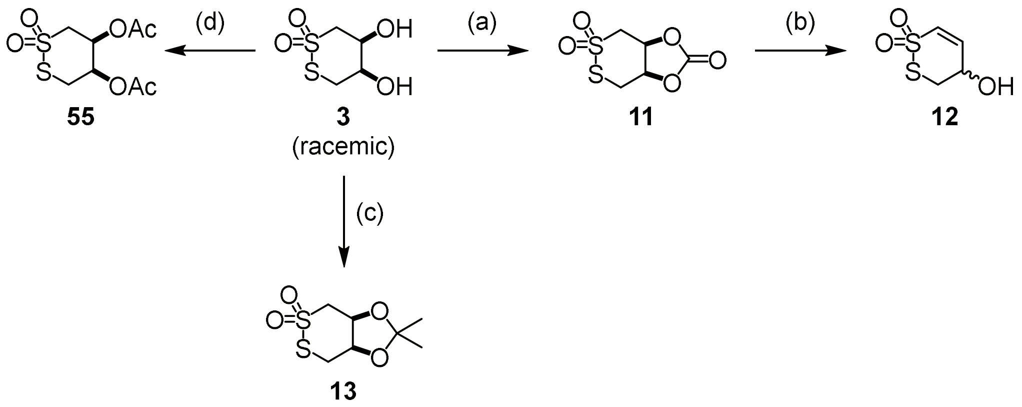

Scheme S1. (a) Triphosgene, pyridine, MeCN, rt, 3 h, 76%; (b) *t*-BuOK, DMF, 0 °C, 30 min, 66%; (c) 2-methoxypropene, H_2SO_4, acetone, rt, 40 min, 94%; (d) Ac_2O, pyridine, rt, 2.5 h, 52%.

Compound 3 (racemic) was synthesized and purified according to procedures described in ref. S2 and S3.

Compound 11. To a solution of **3** (37.6 mg, 0.204 mmol) and pyridine (97 µL, 1.2 mmol) in MeCN (3.0 mL) at -45 °C (MeCN/dry ice bath), a solution of triphosgene (31.3 mg, 0.105 mmol) in MeCN (1.0 mL) was added. The cooling bath was removed and the mixture was stirred at room temperature for 3 h. Silica gel (0.5 g) was added to the mixture, and the volatiles were removed under vacuum. The crude product absorbed on the silica gel was purified by flash column chromatography (SiO_2, 1.5 g, CH_2Cl_2 to CH_2Cl_2/MeOH 10:1, R_f (CH_2Cl_2/MeOH 10:1): 0.50). The fractions containing the desired product were concentrated, and the residue was triturated in CH_2Cl_2/MeOH 10:1 (x3) and pentane to afford **11** (32.8 mg, 76%) as a colorless solid. Mp: 137 – 138 °C; IR (neat): 2986 (w), 2937 (w), 1902 (w), 1802 (s), 1784 (s), 1414 (w), 1365 (m), 1301 (s), 1272 (m), 1251 (m), 1185 (m), 1155 (m), 1125 (s), 1090 (s), 1046 (s), 897 (w), 815 (w), 759 (m), 737(m), 676 (m), 659 (m); ^{1}H NMR (400 MHz, DMSO-d_6): 5.42 (ddd, $^3J_{HH}$ = 7.6, 5.7, 4.2 Hz, 1H), 5.25 (ddd, $^3J_{HH}$ = 7.6, 4.8, 3.2 Hz, 1H), 4.35 (dd, $^2J_{HH}$ = 14.8, $^3J_{HH}$

= 5.7 Hz, 1H), 4.26 (dd, $^2J_{HH}$ = 14.8, $^3J_{HH}$ = 4.2 Hz, 1H), 3.90 (dd, $^2J_{HH}$ = 15.7, $^3J_{HH}$ = 4.8 Hz, 1H), 3.84 (dd, $^2J_{HH}$ = 15.7, $^3J_{HH}$ = 3.2 Hz, 1H); ^{13}C NMR (101 MHz, DMSO-d_6): 153.0 (C), 74.8 (CH), 70.0 (CH), 56.0 (CH_2), 34.2 (CH_2); MS (APCI): 233 ($[M+Na]^+$).

Compound 12. To a solution of **11** (21.7 mg, 0.103 mmol) in DMF (1.0 mL) at 0 °C, *t*-BuOK (14.7 mg, 0.131 mmol) was added. After 30 min, AcOH (8.6 µL, 0.150 mmol) was added and the resulting solution was concentrated. The residue was purified by flash column chromatography (SiO_2, 1 g, CH_2Cl_2/MeOH 100:1 to 50:1, R_f (CH_2Cl_2/MeOH 10:1): 0.38) twice and PTLC (CH_2Cl_2/MeOH 15:1, developed twice) to afford **12** (11.4 mg, 66%) as a colorless solid. Mp: 53 – 54 °C; IR (neat): 3487 (s), 3043 (m), 1410 (m), 1302 (s), 1247 (m), 1186 (m), 1160 (m), 1123 (s), 1063 (s), 1021 (s), 875 (w), 846 (m), 819 (m), 727 (s), 707 (s), 651 (m); ^{1}H NMR (400 MHz, $CDCl_3$): 6.67 (dd, $^3J_{HH}$ = 11.0, $^4J_{HH}$ = 1.7 Hz, 1H), 6.33 (ddt, $^3J_{HH}$ = 11.0, 3.8, $^4J_{HH}$ = 1.3 Hz, 1H), 4.51 – 4.47 (m, 1H), 3.84 (dd, $^2J_{HH}$ = 14.9, $^3J_{HH}$ = 3.5 Hz, 1H), 3.70 (ddd, $^2J_{HH}$ = 14.9, $^3J_{HH}$ = 6.2, $^4J_{HH}$ = 1.3 Hz, 1H); ^{13}C NMR (101 MHz, CD_3OD): 140.9 (CH), 136.2 (CH), 63.1 (CH), 38.6 (CH_2); MS (ESI): 184 ($[M+NH_4]^+$).

Compound 13. To a solution of **3** (20.0 mg, 0.109 mmol) in acetone (5.0 mL) at room temperature, 2-methoxypropene (96 µL, 1.0 mmol) and H_2SO_4 (5.4 µL, 0.10 mmol) were added successively. After the mixture was stirred for 40 min, $Ca(OH)_2$ (39.1 mg, 0.527 mmol) was added. The mixture was stirred for further 20 min, filtered, and concentrated. The residue was purified by flash column chromatography (SiO_2, 1 g, CH_2Cl_2 to CH_2Cl_2/acetone 10:1, R_f (CH_2Cl_2/acetone 5:1): 0.47) to afford **13** (22.8 mg, 94%) as a colorless solid. Mp: 105 – 106 °C; IR (neat): 2993 (w), 2945 (w), 1373 (m), 1312 (s), 1217 (s), 1149 (m), 1128 (s), 1102 (m), 1067 (s), 1038 (s), 931 (m), 882 (m), 850 (m), 807 (m), 665 (m); ^{1}H NMR (400 MHz, $CDCl_3$): 4.66 (ddd, $^3J_{HH}$ = 8.7, 6.1, 4.6 Hz, 1H), 4.40 (ddd, $^3J_{HH}$ = 4.6, 4.6, 3.2 Hz, 1H), 3.75 (dd, $^2J_{HH}$ = 15.3, $^3J_{HH}$ = 3.2 Hz, 1H), 3.69 – 3.61 (m, 2H), 3.51 (dd, $^2J_{HH}$ = 13.7, $^3J_{HH}$ = 8.7 Hz, 1H), 1.55

(s, 3H), 1.42 (s, 3H); ^{13}C NMR (101 MHz, $CDCl_3$): 110.3 (C), 74.6 (CH), 68.3 (CH), 59.6 (CH_2), 34.2 (CH_2), 28.4 (CH_3), 26.4 (CH_3); MS (ESI): 471 ([2M+Na]$^+$), 247 ([M+Na]$^+$).

Compound 55. Compound **3** (12.3 mg, 66.8 μmol) was dissolved in a mixture of pyridine (0.54 mL) and Ac_2O (0.54 mL) at room temperature. After the mixture was stirred for 2.5 h, toluene was added, and the solution was concentrated and azeotropically dried with toluene. The residue was purified by flash column chromatography (SiO_2, 1 g, pentane/EtOAc 5:1 to 1:1, R_f (pentane/EtOAc 1:1): 0.44) to afford **55** (9.4 mg, 52%) as a colorless solid. Mp: 140 – 141 °C; IR (neat): 3006 (w), 2941 (w), 1742 (s), 1370 (m), 1321 (s), 1223 (s), 1200 (s), 1160 (m), 1126 (s), 1037 (s), 960 (s), 780 (m), 714 (m); ^{1}H NMR (400 MHz, $CDCl_3$): 5.51 – 5.43 (m, 2H), 3.81 (dd, $^2J_{HH}$ = 13.0, $^3J_{HH}$ = 10.9 Hz, 1H), 3.59 (dd, $^2J_{HH}$ = 15.3, $^3J_{HH}$ = 1.7 Hz, 1H), 3.56 – 3.46 (m, 2H), 2.17 (s, 3H), 2.08 (s, 3H); ^{13}C NMR (101 MHz, $CDCl_3$): 169.9 (C), 169.2 (C), 69.5 (CH), 64.4 (CH), 59.0 (CH_2), 34.8 (CH_2), 21.0 (CH_3), 20.8 (CH_3); MS (ESI): 827 ([3M+Na]$^+$), 559 ([2M+Na]$^+$), 291 ([M+Na]$^+$).

Compound 15 (racemic) was synthesized and purified according to procedures described in ref. S2 and S3.

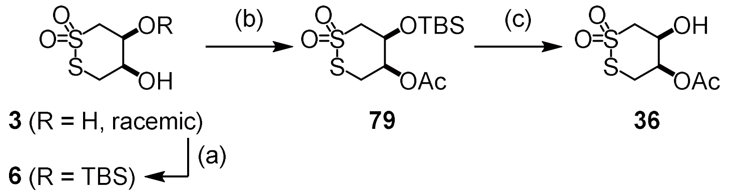

Scheme S2. (a) TBSOTf, 2,6-lutidine, THF, -78 °C to rt, 3.5 h, 87%; (b) Ac_2O, pyridine, CH_2Cl_2, rt, 1.5 h and 19 h (two cycles), 90%; (c) TBAF, AcOH, THF, 0 °C, 25 min, 68%.

Compound 6. To a solution of **3** (74.1 mg, 0.403 mmol) and 2,6-lutidine (0.10 mL, 0.88 mmol) in THF (4.0 mL) at -78 °C (acetone/dry ice bath) under Ar atmosphere, TBSOTf (0.10 mL, 0.44 mmol) was added dropwise. The dry ice was removed to gradually warm up the reaction mixture to room temperature. After the mixture was stirred for 3.5 h, the mixture was

concentrated. The residue was purified by flash column chromatography (SiO_2, 5 g, pentane/EtOAc 5:1 to 1:1, R_f (pentane/EtOAc 3:1): 0.29) to afford **6** (104 mg, 87%) as a colorless solid. Mp: 113 – 114 °C; IR (neat): 3262 (w, br), 2932 (w), 1684 (s), 1529 (w), 1477 (w), 1412 (w), 1362 (w), 1313 (m), 1256 (m), 1200 (m), 1118 (s), 944 (w), 917 (w), 839 (s), 782 (s), 718 (m), 673 (w); ^{1}H NMR (400 MHz, $CDCl_3$): 4.28 (ddd, $^3J_{HH}$ = 10.8, 4.1, 2.7 Hz, 1H), 4.11 (ddd, $^3J_{HH}$ = 4.5, 2.7, 1.6 Hz, 1H), 3.65 (dd, $^2J_{HH}$ = 13.0, $^3J_{HH}$ =10.8 Hz, 1H), 3.52 (dd, $^2J_{HH}$ = 14.9, $^3J_{HH}$ = 1.6 Hz, 1H), 3.39 (dd, $^2J_{HH}$ = 14.9, $^3J_{HH}$ = 4.5 Hz, 1H), 3.25 (dd, $^2J_{HH}$ = 13.0, $^3J_{HH}$ = 4.1 Hz, 1H), 0.91 (s, 9H), 0.15 (s, 6H); ^{13}C NMR (101 MHz, $CDCl_3$): 71.5 (CH), 65.2 (CH), 60.5 (CH_2), 36.2 (CH_2), 25.7 (CH_3), 18.1 (C), -4.5 (CH_3), -4.7 (CH_3); MS (ESI): 316 ($[M+NH_4]^+$).

Compound 79. To a solution of **6** (29.7 mg, 0.995 mmol) in CH_2Cl_2 (1.0 mL) at room temperature, pyridine (0.16 mL, 2.0 mmol) and Ac_2O (95 μL, 1.0 mmol) were added successively. After the mixture was stirred for 1.5 h, toluene was added and the mixture was concentrated. The residue was dissolved in pyridine (0.50 mL) at room temperature and Ac_2O (0.50 mL) was added to the solution. After the mixture was stirred for 19 h, toluene was added and the mixture was concentrated. The residue was purified by flash column chromatography (SiO_2, 1 g, pentane/EtOAc 10:1 to 3:1, R_f (pentane/EtOAc 7:1): 0.27) to afford **79** (30.6 mg, 90%) as a colorless solid. Mp: 85 – 86 °C; IR (neat): 2931 (m), 2856 (w), 1746 (m), 1374 (w), 1316 (m), 1255 (m), 1229 (s), 1202 (m), 1138 (s), 1102 (s), 1047 (m), 951 (m), 893 (w), 837 (s), 780 (s), 747 (m), 674 (w); ^{1}H NMR (400 MHz, $CDCl_3$): 5.38 – 5.29 (m, 1H), 4.35 (ddd, $^3J_{HH}$ = 10.7, 3.8, 2.8 Hz, 1H), 3.70 (dd, $^2J_{HH}$ = 13.2, $^3J_{HH}$ = 10.7 Hz, 1H), 3.50 (dd, $^2J_{HH}$ = 15.2, $^3J_{HH}$ = 1.7 Hz, 1H), 3.43 (dd, $^2J_{HH}$ = 15.2, $^3J_{HH}$ = 4.9 Hz, 1H), 3.36 (dd, $^2J_{HH}$ = 13.2, $^3J_{HH}$ = 3.8 Hz, 1H), 2.15 (s, 3H), 0.87 (s, 9H), 0.12 (s, 3H), 0.11 (s, 3H); ^{13}C NMR (101 MHz, $CDCl_3$): 170.2 (C), 69.9 (CH), 66.7 (CH), 62.1 (CH_2), 34.7 (CH_2), 25.6 (CH_3), 20.9 (CH_3), 18.0 (C), -4.73 (CH_3), -4.81 (CH_3); MS (ESI): 703 ($[2M+Na]^+$), 363 ($[M+Na]^+$), 341 ($[M+H]^+$).

Compound 36. To a solution of **79** (6.9 mg, 20 µmol) and AcOH (3.4 µL, 60 µmol) in THF (0.40 mL) at 0 °C, TBAF (1.0 M in THF, 30 µL, 30 µmol) was added. After the mixture was stirred for 25 min, sat. NH_4Cl aq. was added. The resulting mixture was extracted with EtOAc (x3), and the combined organic layers were dried over Na_2SO_4, filtered, and concentrated. The residue was purified by flash column chromatography (SiO_2, 1 g, pentane/EtOAc 7:1 to 1:1, R_f (pentane/EtOAc 1:2): 0.20) to afford **36** (3.1 mg, 68%) as a colorless solid. Mp: 126 – 127 °C; IR (neat): 3514 (m), 2982 (w), 1731 (s), 1368 (m), 1305 (m), 1228 (s), 1196 (m), 1135 (s), 1039 (s), 993 (m), 952 (m), 905 (m), 779 (m), 681 (w), 627 (w); ^{1}H NMR (400 MHz, $CDCl_3$): 5.33 (ddd, $^3J_{HH}$ = 6.4, 2.7, 1.9 Hz, 1H), 4.50 – 4.41 (br m, 1H), 3.72 – 3.58 (m, 3H), 3.45 (br d, $^2J_{HH}$ = 14.9 Hz, 1H), 2.82 (br s, 1H), 2.19 (s, 3H); ^{13}C NMR (101 MHz, $CDCl_3$): 170.6 (C), 69.2 (CH), 67.9 (CH), 61.9 (CH_2), 33.9 (CH_2), 21.1 (CH_3); MS (ESI): 244 ($[M+NH_4]^+$).

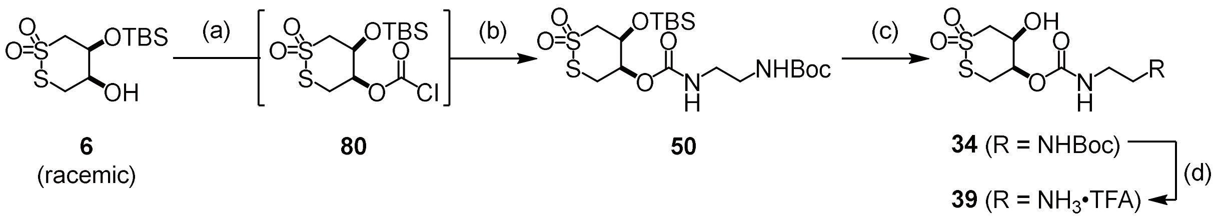

Scheme S3. (a) Triphosgene, pyridine, CH_2Cl_2, rt, 2.5 h; (b) *N*-Boc-ethylenediamine, MeCN, rt, 1 h, 85%; (c) TBAF, AcOH, THF, 0 °C, 25 min, 80%. (d) CH_2Cl_2, TFA, rt, 25 min, 90%.

Compound 50. To a solution of **6** (50.4 mg, 0.169 mmol) and pyridine (81 µL, 1.0 mmol) in CH_2Cl_2 (1.7 mL) at 0 ºC (ice bath), triphosgene (45.9 mg, 0.155 mmol) was added. The cooling bath was removed, and the reaction mixture was stirred for 2.5 h. The mixture was concentrated to give crude chloroformate **80**, which was dissolved in MeCN (1.7 mL). *N*-Boc-ethylenediamine (53 µL, 0.34 mmol) was added to the solution at room temperature. The mixture was stirred for 1 h and concentrated. The residue was purified by flash column chromatography (SiO_2, 0.5 g, pentane/EtOAc 10:1 to 1.5:1, R_f (pentane/EtOAc 3:1): 0.41) to

afford **50** (69.8 mg, 85%) as a colorless solid. Mp: 58 – 59 °C (decomp.); IR (neat): 3354 (br, w), 2933 (w), 1699 (s), 1516 (m), 1464 (w), 1366 (w), 1319 (m), 1252 (s), 1202 (w), 1141 (s), 1111 (s), 916 (w), 839 (s), 780 (s), 750 (w), 672 (w); ^{1}H NMR (400 MHz, $CDCl_3$): 5.38 (br s, 1H), 5.24 (m, 1H), 4.78 (br s, 1H), 4.33 (ddd, $^{3}J_{HH}$ = 10.8, 3.4, 3.4 Hz, 1H), 3.64 (dd, $^{2}J_{HH}$ = 12.7, $^{3}J_{HH}$ = 10.8 Hz, 1H), 3.54 – 3.43 (m, 2H), 3.40 – 3.18 (m, 5H), 1.44 (s, 9H), 0.87 (s, 9H), 0.12 (s, 6H); ^{13}C NMR (101 MHz, $CDCl_3$): 156.6 (C), 155.6 (C), 80.0 (C), 70.1 (CH), 67.2 (CH), 62.1 (CH_2), 42.1 (CH_2), 40.4 (CH_2), 35.3 (CH_2), 28.5 (CH_3), 25.7 (CH_3), 18.0 (C), -4.76 (CH_3), -4.79 (CH_3); MS (ESI): 485 ($[M+H]^+$).

Compound 34. To a solution of **50** (20.0 mg, 41.3 μmol) and AcOH (14 μL, 0.25 mmol) in THF (2.1 mL) at 0 °C, TBAF (1.0 M in THF, 0.12 mL, 0.12 mmol) was added. After the mixture was stirred for 25 min, sat. NH_4Cl aq. was added. The resulting mixture was extracted with EtOAc (x3), and the combined organic layers were dried over Na_2SO_4, filtered, and concentrated. The residue was purified by flash column chromatography twice (SiO_2, 1 g, CH_2Cl_2 to CH_2Cl_2/MeOH 40:1; SiO_2, 0.5 g, CH_2Cl_2 to CH_2Cl_2/MeOH 40:1, R_f (CH_2Cl_2/MeOH 10:1): 0.35) to afford **34** (12.3 mg, 80%) as a colorless solid. Mp: 52 – 53 °C; IR (neat): 3358 (br, m), 2978 (w), 2931 (w), 1690 (s), 1517 (s), 1458 (w), 1395 (w), 1366 (m), 1313 (m), 1246 (s), 1125 (s), 1059 (s), 982 (m), 911 (m), 859 (w), 784 (m), 732 (m), 643 (w); ^{1}H NMR (500 MHz, $CDCl_3$, 50 °C): 5.50 (br s, 1H), 5.31 (br s, 1H), 4.82 (br s, 1H), 4.41 (ddd, $^{3}J_{HH}$ = 10.2, 4.4, 2.8 Hz, 1H), 3.82 – 3.04 (m, 8H), 1.45 (s, 9H); ^{13}C NMR (126 MHz, $CDCl_3$, 50 °C): 157.0 (C), 156.0 (C), 80.4 (C), 69.7 (CH), 68.2 (CH), 62.0 (CH_2), 42.6 (CH_2), 40.4 (CH_2), 35.0 (CH_2), 28.6 (CH_3); HRMS (ESI, +ve) calcd for $C_{12}H_{22}N_2O_7S_2$ ($[M+Na]^+$): 393.0761, found: 393.0762.

Compound 39. To a solution of **34** (5.7 mg, 15 μmol) in CH_2Cl_2 (0.24 mL) at room temperature, TFA (60 μL) was added. The mixture was stirred for 25 min and concentrated. The residue was triturated in Et_2O (x2) and in pentane to afford **39** (5.3 mg, 90%) as a colorless solid. Mp: 60 – 61 °C; IR (neat): 3060 (br, m), 1670 (s), 1526 (m), 1413 (w), 1310 (m), 1258

(m), 1182 (s), 1121 (s), 1058 (s), 958 (m), 913 (m), 837 (m), 797 (m), 722 (m); ^{1}H NMR (400 MHz, CD_3OD) 5.30 – 5.27 (m, 1H), 4.35 (ddd, $^{3}J_{HH}$ = 10.9, 3.7, 3.7 Hz, 1H), 3.71 – 3.35 (m, 6H), 3.15 – 3.00 (m, 2H); ^{13}C NMR (101 MHz, CD_3OD): 158.2 (C), 69.9 (CH), 68.7 (CH), 62.3 (CH_2), 41.1 (CH_2), 39.5 (CH_2), 36.1 (CH_2); MS (ESI): 271 ([M-TFA]$^+$).

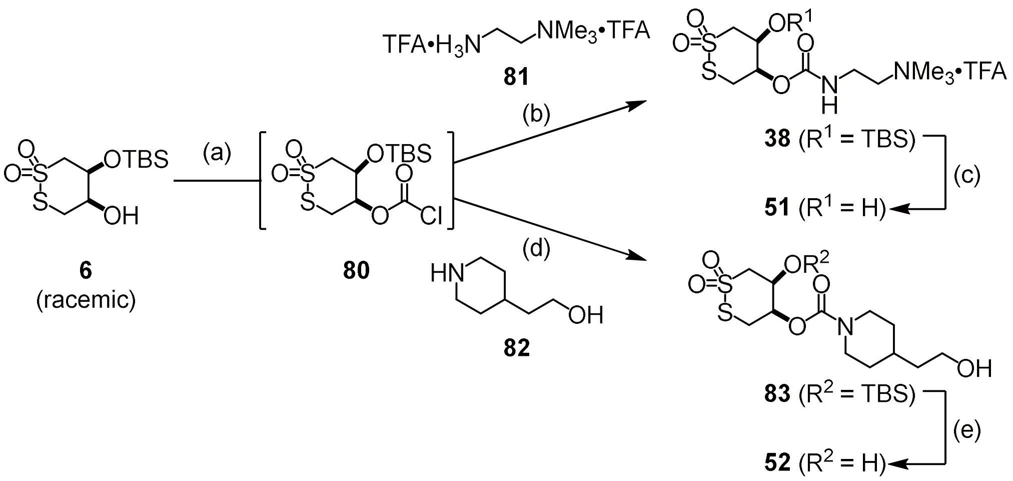

Scheme S4. (a) Triphosgene, pyridine, CH_2Cl_2, rt, 2 h; (b) **81**, Et_3N, DMF/MeCN, rt, 40 min, 31%; (c) TFA, CH_2Cl_2, MeOH, rt, 150 h, 91%; (d) **82**, CH_3CN, rt, 1.5 h, 55%; (e) TBAF, AcOH, THF, 0 °C to rt, 2.5 h, 57%.

Compound 81 was synthesized and purified according to procedures described in ref. S4.

Compound 38. To a solution of **6** (39.2 mg, 0.131 mmol) and pyridine (65 μL, 0.80 mmol) in CH_2Cl_2 (1.3 mL) at 0 °C, triphosgene (42.2 mg, 0.142 mmol) was added. The mixture was warmed up to room temperature, stirred for 2 h, and concentrated to give crude chloroformate **80**, which was dissolved in MeCN (1.1 mL). Compound **81** (57.5 mg, 0.174 mmol, azeotropically dried with toluene) and Et_3N (49 μL,0.35 mmol) were dissolved in DMF (0.29 mL). The stock solution (0.27 mL) was added dropwise to the solution of crude **80** at room temperature. The mixture was stirred for 40 min and concentrated. The residue was purified by reverse phase flash column chromatography (SNAP Ultra C18 12 g, Eluent: H_2O + 0.1% TFA/CH_3CN + 0.1% TFA gradient from 100:0 to 60:40). Fractions containing the desired product were lyophilized to afford **38** (22.5 mg, 31%) as a slightly yellow solid. Mp: 60 – 61

°C; IR (neat): 3262 (br w), 2932 (w), 1719 (m), 1685 (s), 1529 (w), 1477 (w), 1412 (w), 1362 (w), 1313 (m), 1256 (s), 1200 (s), 1169 (m), 1118 (s), 944 (w), 917 (w), 839 (s), 782 (s), 751 (m), 718 (m), 673 (w); ^{1}H NMR (400 MHz, CD_3OD): 5.27 (m, 1H), 4.43 (ddd, $^3J_{HH}$ = 10.6, 3.8, 2.6 Hz, 1H), 3.70 – 3.45 (m, 8H), 3.19 (s, 9H), 0.90 (s, 9H), 0.16 (s, 3H), 0.15 (s, 3H); ^{13}C NMR (101 MHz, CD_3OD): 156.2 (C), 69.9 (CH), 67.8 (CH), 64.7 (t, $^1J_{CN}$ = 2.8 Hz, CH_2), 61.5 (CH_2), 52.7 (t, $^1J_{CN}$ = 3.6 Hz, CH_3), 34.9 (CH_2), 34.3 (CH_2), 24.7 (CH_3), 17.4 (C), -6.29 (CH_3), -6.35 (CH_3); MS (ESI): 427 ($[M-TFA]^+$).

Compound 51. To a solution of **38** (5.5 mg, 10 μmol) in CH_2Cl_2 (0.10 mL) at room temperature, TFA (0.10 mL) was added. The solution was stirred for 30 h, and MeOH (20 μL) was added. The resulting mixture was stirred at room temperature for additional 5 days and concentrated. The residue was purified by reverse phase flash column chromatography (SNAP Ultra C18 12 g, H_2O + 0.1% TFA/CH_3CN + 0.1% TFA gradient from 100:0 to 60:40). Fractions containing the desired product were lyophilized to afford **51** (4.0 mg, 91%) as a sticky colorless amorphous solid. IR (neat): 3262 (br, w), 1715 (m), 1673 (s), 1528 (w), 1480 (w), 1415 (w), 1310 (m), 1259 (m), 1197 (s), 1173 (s), 1118 (s), 1067 (s), 967 (w), 947 (w), 914 (w), 830 (w), 799 (w), 718 (w), 641(w); ^{1}H NMR (400 MHz, CD_3OD): 5.35 – 5.23 (m, 1H), 4.41 – 4.26 (m, 1H), 3.73 – 3.54 (m, 6H), 3.53 – 3.47 (m, 2H), 3.19 (s, 9H); ^{13}C NMR (101 MHz, CD_3OD): 157.6 (C), 69.9 (CH), 69.0 (CH), 66.1 (t, $^1J_{CN}$ = 3.0 Hz, CH_2), 62.3 (CH_2), 54.1 (t, $^1J_{CN}$ = 4.0 Hz, CH_3), 36.4 (CH_2), 36.1 (CH_2); MS (ESI): 313 ($[M-TFA]^+$).

Compound 83. To a solution of **6** (155 mg, 0.519 mmol) and pyridine (0.25 mL, 3.1 mmol) in CH_2Cl_2 (8.0 mL) at 0 °C (ice bath), triphosgene (154 mg, 0.515 mmol) was added. The cooling bath was removed, and the reaction mixture was stirred for 2 h. The mixture was concentrated to give crude chloroformate **80**, which was dissolved in MeCN (6.0 mL). A solution of **82** (80.5 mg, 0.623 mmol) in MeCN (2.0 mL) was added to the solution of crude **80** at room temperature. The mixture was stirred for 1.5 h and concentrated. The residue was

purified by flash column chromatography (SiO_2, 5 g, pentane/EtOAc 2:1 then CH_2Cl_2/MeOH 20:1, R_f (pentane/EtOAc 2:1): 0.08) to afford **83** (130 mg, 55%) as a light-yellow solid. Mp: 131 – 132 °C; IR (neat): 3289 (br w), 2928 (m), 2856 (m), 1702 (s), 1470 (m), 1434 (m), 1361 (w), 1311 (m), 1245 (m), 1199 (m), 1112 (s), 1078 (s), 975 (m), 916 (m), 900 (m), 837 (s), 782 (s), 752 (s), 673 (w); ^1H NMR (400 MHz, $CDCl_3$): 5.25 (br m, 1H), 4.34 (ddd, $^3J_{HH}$ = 10.8, 4.0, 2.8 Hz, 1H), 4.25 – 4.05 (m, 2H), 3.72 (t, $^3J_{HH}$ = 6.5 Hz, 2H), 3.60 (dd, $^2J_{HH}$ = 13.0, $^3J_{HH}$ = 10.8 Hz, 1H), 3.54 – 3.45 (m, 2H), 3.36 (dd, $^2J_{HH}$ = 13.0, $^3J_{HH}$ = 4.0 Hz, 1H), 2.94 – 2.67 (m, 2H), 1.81 – 1.60 (m, 3H), 1.58 – 1.51 (overlapped with H_2O peak, 2H), 1.33 – 1.06 (m, 2H), 0.87 (s, 9H), 0.11 (s, 6H); ^{13}C NMR (101 MHz, $CDCl_3$): 154.0 (C), 70.1 (CH), 67.6 (CH), 62.3 (CH_2), 60.3 (CH_2), 44.6 (CH_2), 39.4 (CH_2), 35.5 (CH_2), 32.6 (CH), 32.4 (CH_2), 25.7 (CH_3), 18.0 (C), -4.70 (CH_3), -4.79 (CH_3); MS (ESI): 454 ($[M+H]^+$).

Compound 52. To a solution of **83** (95 mg, 0.21 mmol) and AcOH (72 µL, 1.3 mmol) in THF (5.0 mL) at 0 °C, TBAF (1.0 M in THF, 0.63 mL, 0.63 mmol) was added. After the mixture was stirred at 0 °C for 30 min and at room temperature for 2 h, sat. NH_4Cl aq. was added. The resulting mixture was extracted with EtOAc (x3), and the combined organic layers were dried over Na_2SO_4, filtered, and concentrated. The residue was purified by flash column chromatography (SiO_2, 5 g, CH_2Cl_2/MeOH 20:1, R_f (CH_2Cl_2/MeOH 20:1): 0.46) to afford **52** (42 mg, 57%) as a colorless solid. Mp: 130 – 131 °C; IR (neat): 3288 (br w), 2929 (w), 2860 (w), 1673 (s), 1439 (m), 1316 (m), 1273 (w), 1250 (m), 1196 (m), 1131 (m), 1061 (m), 914 (m), 784 (w), 763 (w), 644 (w); ^1H NMR (400 MHz, CD_3OD): 5.25 (ddd, $^3J_{HH}$ = 4.5, 2.6, 1.6 Hz, 1H), 4.32 (ddd, $^3J_{HH}$ = 9.8, 5.2, 2.6 Hz, 1H), 4.24 – 4.05 (m, 2H), 3.70 – 3.48 (m, 6H), 3.02 – 2.76 (m, 2H), 1.81 – 1.70 (m, 2H), 1.72 – 1.61 (m, 1H), 1.49 (td, $^3J_{HH}$ = 6.7, 6.7 Hz, 2H), 1.28 – 1.06 (m, 2H); ^{13}C NMR (101 MHz, CD_3OD): 154.9 (C), 69.2 (CH), 68.5 (CH), 61.8 (CH_2), 59.4 (CH_2), 44.7 (CH_2), 39.3 (CH_2), 35.3 (CH_2), 32.8 (CH), 32.2 (CH_2); MS (ESI): 340 ($[M+H]^+$).

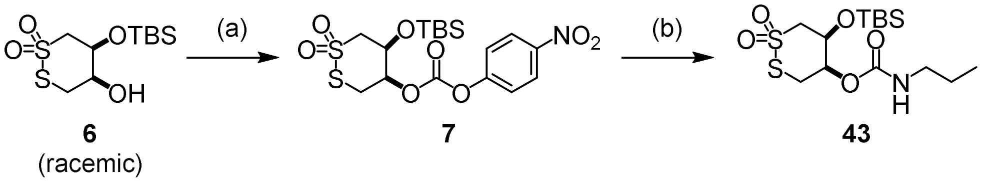

Scheme S5. (a) *p*-Nitrophenyl chloroformate, pyridine, CH_2Cl_2, rt, 3 h, 86%; (b) *n*-$PrNH_2$, CH_2Cl_2, rt, 80 min, 80%.

Compound 7. To a solution of **6** (22.1 mg, 74.1 μmol) and *p*-nitrophenyl chloroformate (74.6 mg, 0.370 mmol) in dry CH_2Cl_2 (0.74 mL) at 0 °C, pyridine (0.30 mL, 3.7 mmol) was added dropwise. The mixture was warmed to room temperature and stirred for 3 h. The solution was concentrated, and the residue was purified by flash column chromatography (SiO_2, CH_2Cl_2, R_f (CH_2Cl_2): 0.50) to afford **7** (29.5 mg, 86%) as a colorless solid. Mp: 146 – 147 ˚C; IR (neat): 2930 (w), 2858 (w), 1767 (s), 1529 (s), 1349 (m), 1309 (m), 1253 (s), 1194 (s), 1137 (s), 1108 (s), 853 (s); ^{1}H NMR (300 MHz, $CDCl_3$): 8.30 (d, $^3J_{HH}$ = 9.1 Hz, 2H), 7.38 (d, $^3J_{HH}$ = 9.1 Hz, 2H), 5.37 – 5.31 (m, 1H), 4.45 (ddd, $^3J_{HH}$ = 10.8, 3.9, 2.7 Hz, 1H), 3.75 (dd, $^2J_{HH}$ = 13.2, $^3J_{HH}$ = 10.8 Hz, 1H), 3.64 (dd, $^2J_{HH}$ = 15.4, $^3J_{HH}$ = 1.1 Hz, 1H), 3.55 (dd, $^2J_{HH}$ = 15.4, $^3J_{HH}$ = 4.8 Hz, 1H), 3.41 (dd, $^2J_{HH}$ = 13.2, $^3J_{HH}$ = 3.9 Hz, 1H), 0.89 (s, 9H), 0.15 (s, 3H), 0.14 (s, 3H); ^{13}C NMR (75 MHz, $CDCl_3$): 155.3 (C), 152.1 (C), 145.8 (C), 125.6 (CH), 121.8 (CH), 71.6 (CH), 70.0 (CH), 61.9 (CH_2), 34.5 (CH_2), 25.6 (CH_3), 18.0 (C), -4.76 (CH_3), -4.83 (CH_3); MS (MALDI): 486 ($[M+Na]^+$)

Compound 43. To a solution of **7** (24.5 mg, 52.8 μmol) in CH_2Cl_2 (0.53 mL) at room temperature, *n*-$PrNH_2$ (13 μL, 0.16 mmol) was added. The mixture was stirred for 80 min and concentrated. The residue was purified by flash column chromatography (SiO_2, 1 g, CH_2Cl_2, R_f (CH_2Cl_2/acetone 50:1): 0.33) and PTLC (developed with CH_2Cl_2/acetone 50:1) to afford **43** (16.2 mg, 80%) as a colorless solid. Mp: 140 – 141 °C; IR (neat): 3261 (w), 2932 (m), 2859 (w), 1726 (m), 1694 (s), 1556 (m), 1470 (w), 1315 (s), 1259 (s), 1206 (m), 1133 (s), 1112 (s), 1051 (m), 979 (w), 921 (w), 838 (s), 783 (s), 748 (s), 673 (m); ^{1}H NMR (400 MHz, $CDCl_3$):

5.23 (ddd, $^3J_{HH}$ = 3.1, 3.1, 2.8 Hz, 1H), 4.88 (br s, 1H), 4.33 (ddd, $^3J_{HH}$ = 10.7, 3.8, 2.8 Hz, 1H), 3.65 (dd, $^2J_{HH}$ = 13.0, $^3J_{HH}$ = 10.7 Hz, 1H), 3.55 – 3.41 (m, 2H), 3.34 (dd, $^2J_{HH}$ = 13.0, $^3J_{HH}$ = 3.8 Hz, 1H), 3.25 – 3.02 (m, 2H), 1.62 – 1.49 (m, 2H), 0.93 (t, $^3J_{HH}$ = 7.4 Hz, 3H), 0.87 (s, 9H), 0.12 (s, 6H); ^{13}C NMR (101 MHz, $CDCl_3$): 155.3 (C), 70.1 (CH), 67.0 (CH), 62.1 (CH_2), 43.0 (CH_2), 35.4 (CH_2), 25.7 (CH_3), 23.2 (CH_2), 18.0 (C), 11.3 (CH_3), -4.76 (CH_3), -4.80 (CH_3); MS (ESI): 406 ([M+Na]$^+$).

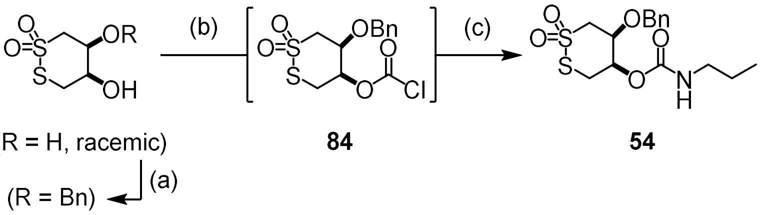

Scheme S6. (a) BnBr, KI, NaH, DMF, -45 °C to rt, 25 h, 16%; (b) triphosgene, pyridine, CH_2Cl_2, rt, 2.5 h; (c) *n*-$PrNH_2$, CH_2Cl_2, rt, 1 h, 15%.

Compound 53. To a solution of **3** (190 mg, 1.03 mmol), KI (40.2 mg, 0.242 mmol) and BnBr (0.59 mL, 5.0 mmol) in DMF (5.0 mL) at -45 °C (MeCN/dry ice bath), NaH (60% with mineral oil, 42.3 mg, 1.06 mmol) was added. The cooling bath was removed and the mixture was stirred at room temperature for 25 h. Sat. NH_4Cl aq. was added, and the mixture was extracted with CH_2Cl_2 (x3). The combined organic layers were dried over Na_2SO_4, filtered, and concentrated. The residue was purified by flash column chromatography (SiO_2, 5 g, CH_2Cl_2 to CH_2Cl_2/MeOH 10:1, R_f (CH_2Cl_2/MeOH 15:1): 0.29) and reverse phase flash column chromatography (SNAP Ultra C18 12 g, H_2O + 0.1% TFA/CH_3CN + 0.1% TFA gradient from 90:10 to 40:60) to give a mixture of regioisomers. The mixture was triturated in $CHCl_3$ (x3) to afford **53** (42.9 mg, 16%, single regioisomer) as a colorless solid. Mp: 158 – 159 °C; IR (neat): 3424 (m), 2929 (w), 1401 (w), 1361 (m), 1304 (s), 1234 (m), 1200 (m), 1129 (s), 1095 (m), 1062 (s), 997 (m), 926 (m), 841 (w), 779 (m), 749 (m), 711 (m), 699 (m), 648 (m); ^{1}H NMR (400 MHz, $CDCl_3$): 7.43 – 7.35 (m, 3H), 7.35 – 7.30 (m, 2H), 4.69 (d, $^2J_{HH}$ = 11.6 Hz, 1H), 4.62 (d, $^2J_{HH}$ = 11.6 Hz, 1H), 4.39 – 4.26 (m, 1H), 4.07 (ddd, $^3J_{HH}$ = 10.9, 3.9, 2.6 Hz, 1H), 3.71

(dd, $^2J_{HH}$ = 13.0, $^3J_{HH}$ = 10.9 Hz, 1H), 3.49 (dd, $^2J_{HH}$ = 14.9, $^3J_{HH}$ = 1.7 Hz, 1H), 3.46 (dd, $^2J_{HH}$ = 13.0, $^3J_{HH}$ = 3.9 Hz, 1H), 3.38 (dd, $^2J_{HH}$ = 14.9, $^3J_{HH}$ = 4.8 Hz, 1H), 2.57 (br s, 1H); ^{13}C NMR (101 MHz, $CDCl_3$): 136.6 (C), 129.0 (CH), 128.8 (CH), 128.1 (CH), 77.3 (CH), 72.0 (CH_2), 63.3 (CH), 58.2 (CH_2), 36.6 (CH_2); MS (ESI): 292 ($[M+NH_4]^+$).

Compound 54. To a solution of **53** (16.3 mg, 59.4 μmol) and pyridine (29 μL, 0.36 mmol) in CH_2Cl_2 (0.59 mL) at 0 °C, triphosgene (17.5 mg, 59.0 μmol) was added. The mixture was warmed up to room temperature, stirred for 2.5 h and concentrated to give crude chloroformate **84**. The residue was dissolved in CH_2Cl_2 (1.1 mL), and *n*-$PrNH_2$ (14.6 μL, 0.18 mmol) was added at room temperature. After the mixture was stirred for 1 h, MeOH was added and the resulting solution was concentrated. The residue was purified by flash column chromatography (SiO_2, 1 g, CH_2Cl_2 to CH_2Cl_2/MeOH 100:1, R_f (CH_2Cl_2/MeOH 50:1): 0.28) twice and reverse phase flash column chromatography (SNAP Ultra C18 12 g, Eluent: H_2O + 0.1% TFA/CH_3CN + 0.1% TFA gradient from 80:20 to 15:85) to afford **54** (3.3 mg, 15%) as a colorless oil. IR (neat): 3399 (br w), 2963 (w), 2932 (w), 1711 (s), 1523 (m), 1456 (w), 1405 (w), 1315 (s), 1234 (s), 1201 (m), 1126 (s), 1074 (s), 971 (m), 920 (m), 785 (m), 738 (m), 698 (m), 646 (w), 618 (w); ^{1}H NMR (400 MHz, $CDCl_3$): 7.41 – 7.29 (m, 5H), 5.56 – 5.45 (m, 1H), 4.91 (br s, 1H), 4.71 (d, $^2J_{HH}$ = 11.5 Hz, 1H), 4.58 (d, $^2J_{HH}$ = 11.5 Hz, 1H), 4.10 (ddd, $^3J_{HH}$ = 11.2, 3.8, 2.6 Hz, 1H), 3.69 (dd, $^2J_{HH}$ = 13.0, $^3J_{HH}$ = 11.2 Hz, 1H), 3.57 – 3.41 (m, 3H), 3.18 (td, $^3J_{HH}$ = 7.0, 6.6 Hz, 2H), 1.55 (qt, $^3J_{HH}$ = 7.4, 7.0 Hz, 2H), 0.93 (t, $^3J_{HH}$ = 7.4 Hz, 3H); ^{13}C NMR (101 MHz, $CDCl_3$): 155.2 (C), 136.7 (C), 128.8 (CH), 128.6 (CH), 128.2 (CH), 75.7 (CH), 72.0 (CH_2), 64.0 (CH), 59.8 (CH_2), 43.1 (CH_2), 35.7 (CH_2), 23.2 (CH_2), 11.4 (CH_3); MS (ESI): 377 ($[M+NH_4]^+$).

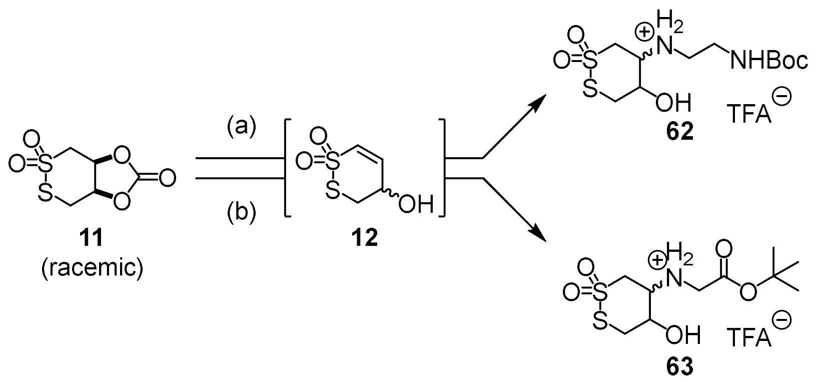

Scheme S7. (a) *N*-Boc-Ethylenediamine, MeCN, rt, 1 h, 67%, dr 1:1; (b) TBAOH, H-Gly-O*t*-Bu·HCl, DMF, rt, 16 h, 76%, dr 55:45.

Compounds 62. To a suspension of **11** (21.2 mg, 0.101 mmol) in MeCN (0.20 mL) at room temperature, *N*-Boc-ethylenediamine (17 µL, 0.107 mmol) was added. After the mixture was stirred for 30 min, MeCN (0.50 mL) was added. The mixture was stirred for further 30 min and concentrated. The residue was purified by flash column chromatography (SiO_2, 2 g, CH_2Cl_2/MeOH 50:1 to 15:1, R_f (CH_2Cl_2/MeOH 10:1): 0.29) and reverse phase flash column chromatography (SNAP Ultra C18 12 g, Eluent: H_2O + 0.1% TFA/CH_3CN + 0.1% TFA gradient from 100:0 to 50:50) to afford **62** (30.0 mg, 67%, a 1:1 mixture of two diastereomers) as a colorless solid. IR (neat): 3364 (w), 2985 (w), 2942 (w), 1665 (s), 1520 (m), 1445 (w), 1369 (w), 1313 (m), 1283 (w), 1252 (w), 1169 (m), 1130 (s), 1071 (m), 929 (w), 838 (w), 799 (m), 784 (m), 722 (m); ^{1}H NMR (400 MHz, CD_3OD) **62a**: 4.19 (ddd, $^3J_{HH}$ = 8.9, 8.4, 6.0 Hz, 1H), 4.10 – 4.04 (m, 1H), 3.95 (dd, $^2J_{HH}$ = 12.6, $^3J_{HH}$ = 12.2 Hz, 1H), 3.90 – 3.83 (m, 1H), 3.42 – 3.37 (m, 4H), 3.29 – 3.15 (m, 2H), 1.45 (s, 9H); **62b**: 4.51 – 4.46 (br m, 1H), 4.08 – 4.03 (m, 1H), 3.90 – 3.84 (m, 1H), 3.78 (dd, $^2J_{HH}$ = 12.8, $^3J_{HH}$ = 3.9 Hz, 1H), 3.66 (dd, $^2J_{HH}$ = 15.2, $^3J_{HH}$ = 1.4 Hz, 1H), 3.49 (dd, $^2J_{HH}$ = 15.2, $^3J_{HH}$ = 5.0 Hz, 1H), 3.42 – 3.37 (m, 2H), 3.29 – 3.15 (m, 2H), 1.45 (s, 9H). ^{13}C NMR (101 MHz, CD_3OD) **62a**: 162.7 (q, $^2J_{CF}$ = 35.7 Hz, TFA anion), 159.36/159.08 (C), 118.0 (q, $^1J_{CF}$ = 292 Hz, TFA anion), 81.24/81.16 (C), 69.0 (CH), 60.3 (CH), 60.2 (CH_2), 46.6 (CH_2), 38.2/38.0 (CH_2), 37.0 (CH_2), 28.6 (CH_3); **62b**: 162.7 (q, $^2J_{CF}$ = 35.7

Hz, TFA anion), 159.36/159.08 (C), 118.0 (q, $^{1}J_{CF}$ = 292 Hz, TFA anion), 81.24/81.16 (C), 60.5 (CH), 60.0 (CH), 55.9 (CH_2), 46.6 (CH_2), 39.7 (CH_2), 38.2/38.0 (CH_2), 28.6 (CH_3); MS (ESI): 327 ($[M-TFA]^+$).

Compounds 63. To a solution of H-Gly-O*t*-Bu·HCl (19.5 mg, 0.116 mmol) in DMF (0.50 mL) at room temperature, TBAOH (1.0 M in MeOH, 0.11 mL, 0.11 mmol) and **11** (20.6 mg, 98.0 µmol) were added successively. The mixture was stirred for 16 h and concentrated. The residue was purified by flash column chromatography (SiO_2, 1 g, CH_2Cl_2 to CH_2Cl_2/MeOH 50:1, R_f (CH_2Cl_2/MeOH 15:1): 0.27) and reverse phase flash column chromatography (SNAP Ultra C18 12 g, Eluent: H_2O + 0.1% TFA/CH_3CN + 0.1% TFA gradient from 100:0 to 60:40) to afford **63** (30.8 mg, 76%, a 55:45 mixture of two diastereomers) as a colorless solid. IR (neat):3254 (w), 2985 (w), 2936 (w), 1740 (m), 1662 (s), 1595 (w), 1480 (w), 1426 (w), 1373 (w), 1321 (m), 1302 (m), 1255 (m), 1198 (m), 1177 (m), 1130 (s), 1082 (w), 1064 (w), 1046 (w), 953 (w), 924 (w), 890 (w), 835 (w), 804 (w), 749 (w), 721 (w), 688 (w); ^{1}H NMR (400 MHz, CD_3OD) **63a** (major): 4.15 – 4.06 (m, 1H), 4.02 (dd, $^{2}J_{HH}$ = 12.6, $^{3}J_{HH}$ = 2.9 Hz, 1H), 3.91 – 3.81 (m, 2H), 3.85 – 3.76 (m, 1H), 3.75 – 3.68 (m, 1H), 3.38 (d, $^{3}J_{HH}$ = 7.3 Hz, 2H), 1.53/1.52 (s, 9H); **63b** (minor): 4.47 (br s, 1H), 3.98 – 3.89 (m, 3H), 3.88 – 3.73 (m, 2H), 3.64 (dd, $^{2}J_{HH}$ = 15.2, $^{3}J_{HH}$ = 0.9 Hz, 1H), 3.47 (dd, $^{2}J_{HH}$ = 15.2, $^{3}J_{HH}$ = 4.9 Hz, 1H), 1.53/1.52 (s, 9H); ^{13}C NMR (101 MHz, CD_3OD) **63a** (major): 168.1 (C), 85.3/85.0 (C), 69.9 (CH), 61.10 (CH_2), 60.6 (CH), 47.3 (CH_2), 37.1 (CH_2), 28.2 (CH_3); **63b** (minor): 167.4 (C), 85.3/85.0 (C), 61.05 (CH), 60.1 (CH), 56.4 (CH_2), 46.8 (CH_2), 39.8 (CH_2), 28.2 (CH_3); MS (ESI): 298 ($[M-TFA]^+$).

2.1.2. Synthesis of Amino-Substituted Cyclic Thiosulfonates

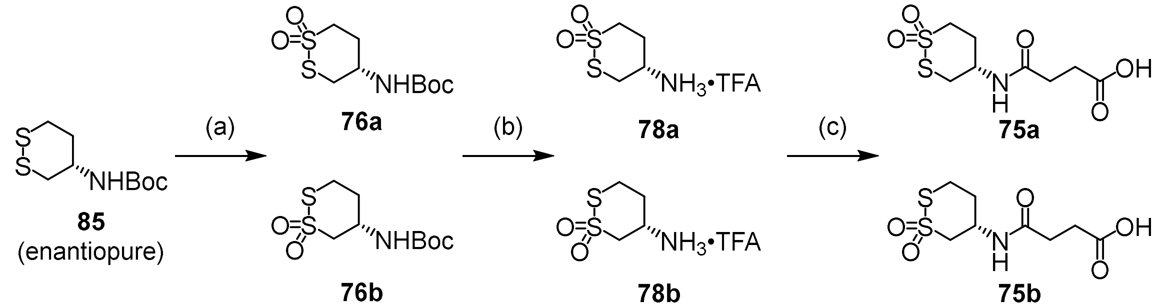

Scheme S8. (a) *m*-CPBA, CH_2Cl_2, rt, 22 h, 66%, **76a**:**76b** = 6:1; (b) HCl, MeOH, rt, 12 h, then anion exchange, 81%, **78a**:**78b** = 9:1; (c) succinic anhydride, Et_3N, CH_2Cl_2, rt, 12 h, 59%, **75a**:**75b** = 7:1.

Compound 85 was synthesized and purified according to procedures described in ref. S5.

Compounds 76. To a solution of **85** (141 mg, 0.599 mmol) in CH_2Cl_2 (6.0 mL) at room temperature, *m*-CPBA (77% with H_2O, 340 mg, 1.52 mmol) was added. After 22 h, the mixture was diluted with CH_2Cl_2, washed with sat. $NaHCO_3$ aq. (x3), dried over Na_2SO_4, filtered, and concentrated to afford **76** (105 mg, 66%, **76a**:**76b** = 6:1) as a colorless solid. IR (neat): 3344 (w), 2981 (w), 2941 (w), 1681 (s), 1519 (s), 1461 (w), 1415 (w), 1392 (w), 1367 (w), 1305 (s), 1258 (m), 1226 (w), 1167 (m), 1128 (s), 1054 (m), 1013 (m), 894 (w), 876 (w), 856 (w), 804 (w), 782 (w), 746 (w), 715 (m), 631 (w); ^{1}H NMR (500 MHz, DMSO-d_6) **76a** (major): 7.28 (d, $^3J_{HH}$ = 7.4 Hz, 1H), 3.94 – 3.84 (m, 1H), 3.77 – 3.64 (m, 2H), 3.36 (dd, $^2J_{HH}$ = 13.8, $^3J_{HH}$ = 3.1 Hz, 1H), 3.27 (dd, $^2J_{HH}$ = 13.8, $^3J_{HH}$ = 8.8 Hz, 1H), 2.27 – 2.18 (m, 2H), 1.39 (s, 9H); **76b** (minor, some peaks were deduced from HSQC and HMBC): 7.25 (d, $^3J_{HH}$ = 7.4 Hz, 1H), 4.02 – 3.95 (m, 1H), 3.71 (1H), 3.45 (ddd, $^2J_{HH}$ = 14.3, $^3J_{HH}$ = 4.6, 3.2 Hz, 1H), 3.25 – 3.15 (m, 2H), 2.24 (1H), 1.75 – 1.65 (m, 1H), 1.39 (s, 9H); ^{13}C NMR (126 MHz, DMSO-d_6) **76a** (major): 154.8 (C), 78.4 (C), 58.0 (CH_2), 45.2 (CH), 38.0 (CH_2), 30.9 (CH_2), 28.2 (CH_3); **76b** (minor): 154.3 (C), 78.6 (C), 63.4 (CH_2), 48.4 (CH), 31.62 (CH_2), 31.58 (CH_2), 28.1 (CH_3); MS (ESI): 285 ($[M+NH_4]^+$).

Compounds 78. Compounds **76** (33 mg, 0.12 mmol, **76a**:**76b** = 6:1) were dissolved in a solution of HCl (1.25 M in MeOH, 3.0 mL) at room temperature. The reaction mixture was stirred for 12 h and concentrated. The residue was purified by preparative HPLC (flow rate: 4 mL/min, detection wavelength: 210 nm, eluent: H_2O + 0.1% TFA/ MeCN + 0.1% TFA gradient from 100:0 to 99:1 (the first 7 min), then 99:1 to 5:95 in 25 min). Fractions containing the desired products were lyophilized to afford **78** (28 mg, 81%, **78a**:**78b** = 9:1, anion exchange took place during purification) as a colorless, very hygroscopic solid. IR (neat): 2934 (br w), 1779 (w), 1666 (m), 1531 (w), 1432 (w), 1381 (w), 1325 (m), 1303 (m), 1165 (s), 1128 (s), 1033 (w), 997 (w), 895 (w), 839 (w), 796 (m), 720 (m), 705 (m), 632 (w); ^{1}H NMR (400 MHz, DMSO-d_6) **78a** (major): 8.53 (s, 3H), 3.86 – 3.77 (m, 2H), 3.76 – 3.66 (m, 1H), 3.61 – 3.47 (overlapped with H_2O peak, 2H), 2.50 – 2.42 (m, 1H), 2.42 – 2.32 (m, 1H); **78b** (minor): 8.53 (s, 3H), 3.92 (dd, $^{2}J_{HH}$ = 12.8, $^{3}J_{HH}$ = 3.0 Hz, 1H), 3.81 – 3.73 (m, 1H), 3.66 – 3.56 (overlapped with H_2O peak, 1H), 3.62 – 3.54 (overlapped with H_2O peak, 1H), 3.28 (dd, $^{2}J_{HH}$ = 12.8, $^{3}J_{HH}$ = 12.8 Hz, 1H), 2.49 – 2.44 (m, 1H), 2.06 – 1.85 (m, 1H); ^{13}C NMR (126 MHz, DMSO-d_6) **78a** (major): 158.4 (q, $^{2}J_{CF}$ = 32.6 Hz, TFA anion), 116.7 (q, $^{1}J_{CF}$ = 297 Hz, TFA anion), 57.1 (CH_2), 45.3 (CH), 35.9 (CH_2), 29.3 (CH_2); **78b** (minor): 158.4 (q, $^{2}J_{CF}$ = 32.6 Hz, TFA anion), 116.7 (q, $^{1}J_{CF}$ = 297 Hz, TFA anion), 60.8 (CH_2), 48.3 (CH), 31.3 (CH_2), 29.1 (CH_2); HPLC-MS: R_t = 0.33 min, 168 ($[M+H]^+$).

Compounds 75. To a solution of **78** (39 mg, 0.24 mmol, **78a**:**78b** = 9:1) in CH_2Cl_2 (2.0 mL) at room temperature, Et_3N (99 μL, 0.71 mmol) and succinic anhydride (28.3 mg, 0.283 mmol) were added successively. The reaction mixture was stirred for 12 h and concentrated. The residue was purified by preparative HPLC (flow rate: 4 mL/min, detection wavelength: 210 nm, eluent: CH_3CN + 0.1% TFA/ H_2O + 0.1% TFA gradient from 0% CH_3CN + 0.1% TFA to 1% (the first 7 min), then 1% to 95% in 20 min). Fractions containing the desired products were lyophilized to afford **75** (37 mg, 59%, **75a**:**75b** = 7:1) as a very hygroscopic colorless

solid. IR (neat): 3282 (m), 2929 (br w), 2550 (br w), 1701 (s), 1671 (w), 1623 (s), 1556 (s), 1436 (w), 1408 (w), 1376 (w), 1320 (m), 1297 (s), 1252 (m), 1229 (m), 1196 (w), 1172 (m), 1122 (s), 1023 (w), 997 (w), 929 (m), 895 (m), 837 (m), 806 (w), 762 (w), 739 (m), 716 (m), 648 (w); ^{1}H NMR (500 MHz, DMSO-d_6) **75a (**major): 12.08 (br s, 1H), 8.19 (d, $^3J_{HH}$ = 7.4 Hz, 1H), 4.22 – 4.11 (m, 1H), 3.80 – 3.63 (m, 2H), 3.40 (d, $^2J_{HH}$ = 13.8 Hz, 1H), 3.27 (dd, $^2J_{HH}$ = 13.8, $^3J_{HH}$ = 6.8 Hz, 1H), 2.47 – 2.41 (m, 2H), 2.42 – 2.28 (m, 2H), 2.31 – 2.16 (m, 2H); **75b (**minor): 12.08 (br s, 1H), 8.24 – 8.15 (br m, 1H), 4.35 – 4.23 (m, 1H), 3.70 – 3.63 (m, 1H), 3.48 (ddd, $^2J_{HH}$ = 14.5, $^3J_{HH}$ = 4.4, 3.4 Hz, 1H), 3.33 – 3.26 (m, 1H), 3.27 – 3.19 (m, 1H), 2.47 – 2.41 (m, 2H), 2.42 – 2.28 (m, 2H), 2.26 – 2.19 (m, 1H), 1.81 – 1.68 (m, 1H); ^{13}C NMR (126 MHz, DMSO-d_6) **75a (**major): 173.8 (C), 170.9 (C), 57.5 (CH_2), 43.1 (CH), 37.9 (CH_2), 30.6 (CH_2), 30.0 (CH_2), 29.03 (CH_2); **75b (**minor): 173.7 (C), 170.3 (C), 63.0 (CH_2), 46.9 (CH), 31.7 (CH_2), 31.1 (CH_2), 30.0 (CH_2), 28.97 (CH_2); MS (ESI): 290 ([M+Na]$^+$).

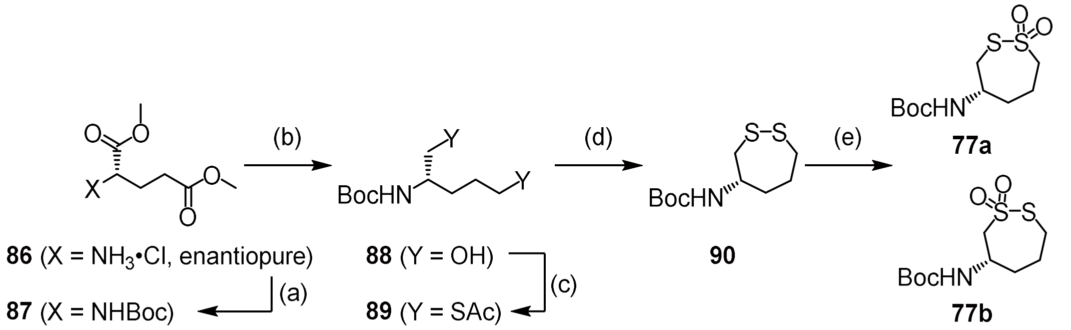

Scheme S9. (a) Boc_2O, $NaHCO_3$, CH_2Cl_2/H_2O, 60 °C, 4 h, 96%; (b) $LiAlH_4$, dry THF, rt, 4 h, 36%; (c) PPh_3, DIAD, AcSH, 0 °C to rt, 17 h, 57%; (d) KOH, air, MeOH, rt, 12 h, 15%; (e) *m*-CPBA, rt, 12 h, 17%, **77a**:**77b** = 10:1.

Compound 87. To a mixture of CH_2Cl_2 (150 mL) and H_2O (100 mL) at room temperature, $NaHCO_3$ (4.2 g, 50 mmol), **86** (8.0 g, 38 mmol) and Boc_2O (9.8 g, 45 mmol) were added successively. The reaction mixture was heated to 60 °C and stirred for 4 h. The reaction mixture was cooled to room temperature, and the organic layer was separated, dried over Na_2SO_4, filtered, and concentrated. The residue was purified by flash column chromatography (SiO_2,

CH_2Cl_2/MeOH 40:1, R_f (CH_2Cl_2/MeOH 25:1): 0.34) to afford **87** (10 g, 96%) as a colorless oil. Spectroscopic data were identical to those reported in ref. S6.

Compound 88. To a suspension of $LiAlH_4$ (2.5 g, 66 mmol) in dry THF (200 mL) under N_2 at 0 °C, a solution of **87** (6.0 g, 22 mmol) in dry THF (100 mL) was added dropwise. The reaction mixture was warmed to room temperature and stirred for 3 h. H_2O (1.0 mL) was added dropwise to the reaction mixture at 0 °C, followed by 15% NaOH aq. (1.0 mL) and H_2O (2.0 mL). The resulting mixture was filtered, and the solid was washed several times with CH_2Cl_2. The filtrates were combined, dried over Na_2SO_4, filtered, and concentrated. The residue was purified by flash column chromatography (SiO_2, EtOAc, R_f (EtOAc): 0.27) to afford **88** (1.7 g, 36%) as a colorless oil. Spectroscopic data were identical to those reported in ref. S7.

Compound 89. To a solution of PPh_3 (2.0 g, 7.6 mmol) in dry THF (30 mL) under N_2 at 0 °C, DIAD (1.5 mL, 7.6 mmol) was added dropwise. The reaction mixture was stirred at 0 °C for 20 min. Then a solution of **88** (0.70 g, 3.2 mmol) in dry THF (12 mL) and AcSH (0.55 mL, 7.6 mmol) were added successively. The reaction mixture was stirred at 0 °C for 1 h, then at room temperature for 16 h. The mixture was concentrated, and the residue was purified by flash column chromatography (SiO_2, pentane/EtOAc 4:1, R_f (pentane/EtOAc 4:1): 0.32) to afford **89** (606 mg, 57%) as a colorless oil. $[\alpha]_D^{20}$ -17.0 (*c* 1.00, $CHCl_3$); IR (neat): 3360 (w), 2984 (w), 2937 (w), 1685 (s), 1519 (s), 1454 (w), 1395 (w), 1371 (w), 1353 (w), 1298 (w), 1281 (w), 1245 (m), 1167 (m), 1130 (s), 1101 (m), 1051 (m), 1026 (w), 954 (m), 872 (w), 761 (w), 744 (w), 716 (w), 658 (w), 620 (s); ^1H NMR (400 MHz, $CDCl_3$): 4.48 (d, $^3J_{HH}$ = 7.8 Hz, 1H), 3.80 – 3.54 (br m, 1H), 3.07 (dd, $^2J_{HH}$ = 13.8, $^3J_{HH}$ = 4.6 Hz, 1H), 2.97 (dd, $^2J_{HH}$ = 13.8, $^3J_{HH}$ =7.2 Hz, 1H), 2.91 – 2.83 (m, 2H), 2.34 (s, 3H), 2.32 (s, 3H), 1.71 – 1.51 (m, 4H), 1.42 (s, 9H); ^{13}C NMR (101 MHz, $CDCl_3$): 195.9 (C), 195.7 (C), 155.6 (C), 79.6 (C), 50.3 (CH), 34.0 (CH_2), 33.5 (CH_2), 30.8 (CH_3), 30.7 (CH_3), 28.9 (CH_2), 28.5 (CH_3), 26.3 (CH_2); MS (ESI): 236 ($[M-Boc+2H]^+$), 194 ($[M-Boc-Ac+3H]^+$).

Compound 90. To a solution of **89** (504 mg, 1.50 mmol) in MeOH (50 mL) at room temperature, KOH (340 mg, 6.07 mmol) was added. The reaction mixture was stirred for 12 h with a stream of air bubbling through the solution. The mixture was concentrated, and the residue was purified by flash column chromatography (SiO_2, pentane/EtOAc 30:1 to 3:1, R_f (pentane/EtOAc 9:1): 0.51) to afford **90** (55 mg, 15%) as a colorless solid. Mp: 110 – 111 °C; $[\alpha]_D^{20}$ -37.4 (*c* 1.00, $CHCl_3$); IR (neat): 3362 (m), 2981 (w), 2935 (w), 2907 (w), 1676 (s), 1512 (s), 1443 (w), 1402 (w), 1388 (w), 1365 (w), 1344 (w), 1303 (m), 1278 (m), 1231 (m), 1160 (s), 1045 (m), 999 (m), 929 (w), 863 (w), 825 (w), 780 (w), 750 (w), 622 (w); ^{1}H NMR (400 MHz, DMSO-d_6): 7.01 (d, $^3J_{HH}$ = 7.7 Hz, 1H), 3.76 – 3.59 (m, 1H), 3.06 (dd, $^2J_{HH}$ = 13.6, $^3J_{HH}$ = 4.7 Hz, 1H), 2.92 (dt, $^2J_{HH}$ = 12.5, $^3J_{HH}$ = 4.6 Hz, 1H), 2.66 (ddd, $^2J_{HH}$ = 12.5, $^3J_{HH}$ = 10.3, 4.7 Hz, 1H), 2.60 (dd, $^2J_{HH}$ = 13.6, $^3J_{HH}$ = 9.9 Hz, 1H), 2.21 – 2.10 (m, 1H), 1.93 – 1.71 (m, 3H), 1.37 (s, 9H); ^{13}C NMR (101 MHz, DMSO-d_6): 154.6 (C), 77.8 (C), 53.0 (CH), 44.1 (CH_2), 36.0 (CH_2), 32.8 (CH_2), 28.2 (CH_3), 24.4 (CH_2); MS (ESI): 133 ([M-NHBoc]$^+$).

Compounds 77. To a solution of **90** (149 mg, 0.597 mmol) in CH_2Cl_2 (6.0 mL) at room temperature, *m*-CPBA (368 mg, 1.49 mmol) was added. The reaction mixture was stirred for 12 h, diluted with CH_2Cl_2 and washed with sat. $NaHCO_3$ aq. (x3). The organic layer was dried over Na_2SO_4, filtered, and concentrated. The residue was purified by flash column chromatography (SiO_2, CH_2Cl_2/MeOH 40:1, R_f (CH_2Cl_2/MeOH 40:1) **77a**: 0.31, **77b**: 0.42) to afford **77** (29 mg, 17%, **77a**:**77b** = 10:1) as a colorless solid. IR (neat): 3364 (w), 2977 (w), 2934 (w), 1688 (s), 1509 (m), 1451 (w), 1392 (w), 1366 (w), 1316 (s), 1248 (m), 1163 (s), 1120 (s), 1050 (m), 1025 (m), 1008 (m), 951 (w), 866 (w), 848 (w), 825 (w), 781 (w), 699 (w); ^{1}H NMR (500 MHz, DMSO-d_6) **77a** (major): 7.24 (d, $^3J_{HH}$ = 7.0 Hz, 1H), 4.00 – 3.89 (m, 1H), 3.62 – 3.50 (m, 2H), 3.45 (dd, $^2J_{HH}$ = 15.7, $^3J_{HH}$ = 5.3 Hz, 1H), 3.19 (dd, $^2J_{HH}$ = 15.7, $^3J_{HH}$ = 6.6 Hz, 1H), 2.11 – 2.04 (m, 1H), 2.04 – 1.95 (m, 1H), 1.87 – 1.79 (m, 1H), 1.79 – 1.70 (m, 1H), 1.37 (s, 9H); **77b** (minor): 7.12 (d, $^3J_{HH}$ = 7.1 Hz, 1H), 3.85 – 3.77 (m, 2H), 3.60 – 3.54 (m,

1H), 3.28 (t, $^3J_{HH}$ = 6.3 Hz, 2H), 2.14 – 2.03 (m, 2H), 2.01 – 1.93 (m, 1H), 1.92 – 1.85 (m, 1H), 1.38 (s, 9H); ^{13}C NMR (126 MHz, DMSO-d_6) **77a** (major): 154.7 (C), 78.1 (C), 65.1 (CH_2), 51.2 (CH), 37.7 (CH_2), 30.8 (CH_2), 28.22 (CH_3), 18.2 (CH_2); **77b** (minor):154.5 (C), 78.5 (C), 68.3 (CH_2), 46.7 (CH), 34.6 (CH_2), 33.0 (CH_2), 28.18 (CH_3), 27.1 (CH_2); MS (ESI): 299 ([M+NH_4]$^+$).

2.1.3. Synthesis of Non-Substituted Cyclic Thiosulfonates

General procedure A (for the introduction of SAc groups). To a solution of di-halo or di-tosylate substrate (1.0 equiv.) in DMF (0.10 M) at room temperature, 18-crown-6-ether (1.0 equiv.) and KSAc (4.0 equiv.) were added successively. The reaction mixture was stirred under N_2 at room temperature for 12 h and then concentrated. H_2O and CH_2Cl_2 were added to the residue, and the organic layer was separated, washed with H_2O and brine, dried over Na_2SO_4, filtered, and concentrated. The residue was purified by flash column chromatography (SiO_2) to afford the desired bis-thioacetate.

General procedure B (for the deprotection of SAc groups). Bis-thioacetate substrate was dissolved in a solution of HCl (1.25 M in MeOH) at room temperature to form a 0.30 M solution. The reaction mixture was stirred under N_2 for 4 h and concentrated. The residue was purified by flash column chromatography (SiO_2) to afford the desired dithiol.

General procedure C (for the formation of cyclic thiosulfonates). To a solution of dithiol (1.0 equiv.) in AcOH at 0 °C, 30% H_2O_2 aq. (3.5 equiv.) was added dropwise. The reaction mixture was warmed up to room temperature, stirred for 12 h, and concentrated. The residue was purified by flash column chromatography (SiO_2) to afford the desired thiosulfonate.

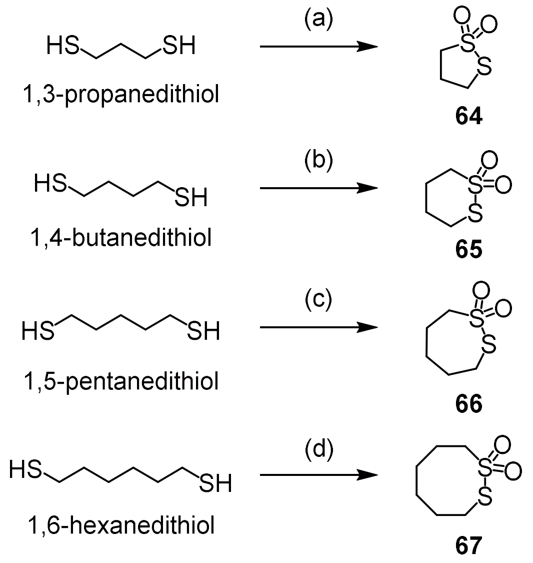

Scheme S10. (a) H_2O_2, 0 ºC to rt, 12 h, 39%; (b) H_2O_2, 0 ºC to rt, 12 h, 20%; (c) H_2O_2, 0 ºC to rt, 12 h, 4%; (d) H_2O_2, 0 ºC to rt, 12 h, 1%.

Compound 64 (537 mg, 39%, colorless oil) was synthesized from 1,3-propanedithiol (1.0 mL, 10 mmol) in AcOH (22 mL) following general procedure C. R_f (CH_2Cl_2): 0.24; IR (neat): 3000 (w), 2943 (w), 2857 (w), 1436 (w), 1409 (w), 1298 (s), 1270 (m), 1210 (w), 1151 (m), 1118 (s), 1028 (w), 994 (w), 909 (w), 873 (w), 844 (w), 733 (w), 699 (m), 660 (w); ^{1}H NMR (400 MHz, $CDCl_3$): 3.72 (t, $^3J_{HH}$ = 6.7 Hz, 2H), 3.42 (t, $^3J_{HH}$ = 6.7 Hz, 2H), 2.61 (tt, $^3J_{HH}$ = 6.7, 6.7 Hz, 2H); ^{13}C NMR (101 MHz, $CDCl_3$): 58.4 (CH_2), 36.9 (CH_2), 25.1 (CH_2); MS (ESI): 156 ($[M+NH_4]^+$).

Compound 65 (45 mg, 20%, colorless solid) was synthesized from 1,4-butanedithiol (184 mg, 1.51 mmol) in AcOH (1.5 mL) following general procedure C. R_f (CH_2Cl_2): 0.49. Spectroscopic data were identical to those reported in ref. S8.

Compound 66 (17 mg, 4%, colorless oil) was synthesized from 1,5-pentanedithiol (0.30 mL, 2.3 mmol) in AcOH (220 mL) following general procedure C. R_f (CH_2Cl_2): 0.53; IR (neat): 2932 (m), 2859 (w), 1448 (m), 1402 (w), 1315 (s), 1291 (s), 1224 (w), 1208 (w), 1173 (w), 1115 (s), 1042 (m), 960 (w), 929 (w), 832 (m), 784 (w), 736 (w), 695 (s), 656 (w); ^{1}H NMR (400 MHz, $CDCl_3$): 3.52 – 3.44 (m, 2H), 3.29 (t, $^3J_{HH}$ = 6.4 Hz, 2H), 2.12 – 1.93 (m, 6H); ^{13}C

NMR (101 MHz, $CDCl_3$): 65.8 (CH_2), 34.3 (CH_2), 30.4 (CH_2), 24.7 (CH_2), 22.5 (CH_2); MS (ESI): 184 ($[M+NH_4]^+$).

Compound 67 (3 mg, 1%, colorless oil) was synthesized from 1,6-hexanedithiol (300 µL, 1.96 mmol) in AcOH (196 ml) following general procedure C. R_f (CH_2Cl_2): 0.50; IR (neat): 2935 (m), 2859 (w), 1458 (w), 1409 (w), 1365 (w), 1309 (s), 1254 (w), 1229 (w), 1121 (s), 1058 (m), 959 (w), 762 (m), 726 (m), 692 (w), 621 (w); ^1H NMR (500 MHz, $CDCl_3$): 3.60 – 3.54 (m, 2H), 3.41 – 3.35 (m, 2H), 2.17 – 2.09 (m, 2H), 1.93 – 1.82 (m, 4H), 1.82 – 1.74 (m, 2H); ^{13}C NMR (126 MHz, $CDCl_3$): 64.2 (CH_2), 36.3 (CH_2), 27.8 (CH_2), 24.5 (CH_2), 23.4 (CH_2), 22.8 (CH_2); MS (ESI): 198 ($[M+NH_4]^+$).

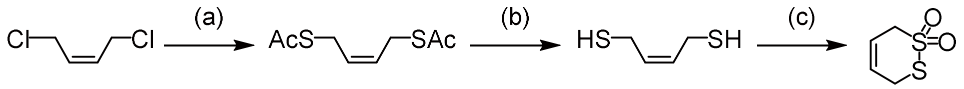

91 92 93 68

Scheme S11. (a) KSAc, 18-crown-6-ether, DMF, rt, under N_2, 12 h, 58%; (b) HCl, MeOH, rt, under N_2, 4 h, 61%; (c) H_2O_2, 0 °C to rt, 12 h, 5%.

Compound 92 (1.7 g, 58%, colorless oil) was synthesized from **91** (1.5 mL, 14 mmol) following general procedure A. R_f (CH_2Cl_2): 0.44. Spectroscopic data were identical to those reported in ref. S9.

Compound 93 (180 mg, 61%, smelly colorless oil) was synthesized from **92** (500 mg, 2.45 mmol) following general procedure B. Spectroscopic data were identical to those reported in ref. S9.

Compound 68 (16 mg, 5%, pale yellow oil) was synthesized from **93** (252 mg, 2.10 mmol) in AcOH (4.5 mL) following general procedure C. R_f (CH_2Cl_2): 0.33; IR (neat): 2940 (w), 2905 (w), 1660 (w), 1413 (w), 1392 (w), 1295 (s), 1253 (m), 1201 (m), 1147 (m), 1115 (s), 1004 (w), 971 (w), 905 (w), 873 (w), 830 (m), 735 (s), 644 (s); ^1H NMR (400 MHz, $CDCl_3$): 6.12 – 6.03 (m, 1H), 5.84 – 5.76 (m, 1H), 3.99 – 3.91 (m, 4H); ^{13}C NMR (101 MHz, $CDCl_3$): 124.0 (CH), 123.1 (CH), 57.2 (CH_2), 34.6 (CH_2).

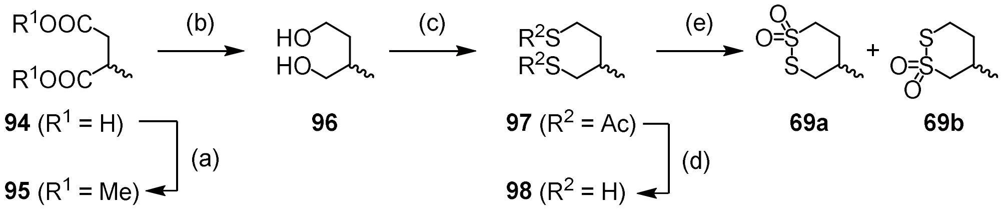

Scheme S12. (a) $SOCl_2$, MeOH, 0 °C to rt, under N_2, 12 h, 97%; (b) $LiAlH_4$, dry THF, 0 °C to rt, 3 h, 82%; (c) PPh_3, DIAD, AcSH, dry THF, 0 °C to rt, under N_2, 17 h, 43%; (d) HCl, MeOH, rt, under N_2, 4 h, 59%; (e) H_2O_2, 0 °C to rt, 12 h, 46%, **69a**:**69b** = 2:1.

Compound 95. To a solution of **94** (5.0 g, 38 mmol) in MeOH (60 mL) under N_2 at 0 °C, $SOCl_2$ (8.3 mL, 114 mmol) was added dropwise. The reaction mixture was warmed up to room temperature, stirred for 12 h, and concentrated. The residue was dissolved in CH_2Cl_2, washed with sat. $NaHCO_3$ aq. and brine, dried over Na_2SO_4, filtered, and concentrated to afford **95** (5.9 g, 97%) as a colorless oil. Spectroscopic data were identical to those reported in ref. S10.

Compound 96. To a suspension of $LiAlH_4$ (2.1 g, 55 mmol) in dry THF (150 mL) under N_2 at 0 °C, a solution of **95** (3.0 g, 19 mmol) in dry THF (50 mL) was added dropwise. The reaction mixture was stirred at 0 °C for 1 h, then at room temperature for additional 2 h. H_2O (2.2 mL) was added dropwise to the reaction mixture at 0 °C, followed by 15% NaOH aq. (2.2 mL) and H_2O (6.6 mL). The resulting mixture was stirred at room temperature for 1 h and filtered. The solid was washed several times with CH_2Cl_2. The filtrates were combined, dried over Na_2SO_4, filtered, and concentrated. The residue was purified by flash column chromatography (SiO_2, EtOAc, R_f (EtOAc): 0.45) to afford **96** (1.6 g, 82%) as a colorless oil. Spectroscopic data were identical to those reported in ref. S11.

Compound 97. To a solution of PPh_3 (6.0 g, 23 mmol) in dry THF (70 mL) under N_2 at 0 °C, DIAD (3.6 mL, 23 mmol) was added dropwise. The reaction mixture was stirred at 0 °C for 20 min. Then a solution of **96** (1.0 g, 9.6 mmol) in dry THF (29 mL) and AcSH (1.6 mL, 23 mmol) were added successively. The reaction mixture was stirred at 0 °C for 1 h, then at room

temperature for 16 h. The mixture was concentrated, and the residue was purified by flash column chromatography (SiO_2, CH_2Cl_2, R_f (CH_2Cl_2): 0.47) to afford **97** (913 mg, 43%) as a colorless oil. IR (neat): 2963 (w), 2928 (w), 1691 (s), 1425 (w), 1378 (w), 1354 (w), 1135 (m), 1108 (m), 958 (m), 627 (m); ^{1}H NMR (400 MHz, $CDCl_3$): 2.99 – 2.74 (m, 4H), 2.33 (s, 3H), 2.32 (s, 3H), 1.82 – 1.70 (m, 1H), 1.70 – 1.58 (m, 1H), 1.51 – 1.40 (m, 1H), 0.97 (d, $^3J_{HH}$ = 6.7 Hz, 3H); ^{13}C NMR (101 MHz, $CDCl_3$): 195.9 (C), 195.8 (C), 35.63 (CH_2), 35.56 (CH_2), 32.9 (CH), 30.80 (CH_3), 30.76 (CH_3), 26.9 (CH_2), 18.9 (CH_3); MS (ESI): 103 ([M-SAc-Ac+H]$^+$).

Compound 98 (332 mg, 59%, smelly colorless oil) was synthesized from **97** (913 mg, 4.14 mmol) following general procedure B. R_f (pentane/CH_2Cl_2 9:1): 0.27; IR (neat): 2958 (s), 2924 (s), 2872 (w), 2551 (w), 1457 (m), 1432 (m), 1377 (m), 1305 (w), 1283 (m), 1256 (w), 1162 (w), 1089 (w), 930 (w), 885 (w), 828 (w), 707 (m), 669 (w); ^{1}H NMR (400 MHz, $CDCl_3$): 2.65 – 2.48 (m, 3H), 2.43 (ddd, $^2J_{HH}$ = 13.3, $^3J_{HH}$ = 8.2, 6.4 Hz, 1H), 1.86 – 1.71 (m, 2H), 1.57 – 1.46 (m, 1H), 1.34 (t, $^3J_{HH}$ = 7.7 Hz, 1H), 1.26 (t, $^3J_{HH}$ = 8.2 Hz, 1H), 0.98 (d, $^3J_{HH}$ = 6.6 Hz, 3H); ^{13}C NMR (101 MHz, $CDCl_3$): 39.6 (CH_2), 34.9 (CH), 31.3 (CH_2), 22.5 (CH_2), 18.3 (CH_3); MS (ESI): 69 ([M-2SH-H]$^+$), 103 ([M-SH]$^+$).

Compounds 69 (112 mg, 46%, colorless oil, **69a**:**69b** = 2:1) were synthesized from **98** (200 mg, 1.47 mmol) in AcOH (2.5 mL) following general procedure C. R_f (CH_2Cl_2): 0.44; IR (neat): 2964 (w), 2928 (w), 2878 (w), 1457 (w). 1418 (w), 1401 (w), 1381 (w), 1313 (s), 1292 (s), 1243 (w), 1202 (w), 1126 (s), 1067 (w), 1046 (w), 945 (w), 890 (m), 849 (w), 778 (m), 734 (w), 713 (m), 674 (w), 650 (w); ^{1}H NMR (500 MHz, $CDCl_3$) **69a** (major): 3.46 – 3.38 (m, 1H), 3.35 (ddd, $^2J_{HH}$ = 13.6, $^3J_{HH}$ = 6.0, 3.1 Hz, 1H), 3.30 – 3.24 (m, 1H), 3.13 – 3.08 (m, 1H), 2.32 – 2.23 (m, 1H), 2.22 – 2.13 (m, 2H), 1.13 (d, $^3J_{HH}$ = 6.4 Hz, 3H); **69b** (minor): 3.53 – 3.44 (m, 1H), 3.32 – 3.27 (m, 1H), 3.17 – 3.11 (m, 1H), 3.11 – 3.03 (m, 1H), 2.57 – 2.45 (m, 1H), 2.22 – 2.15 (m, 1H), 1.62 – 1.51 (m, 1H), 1.11 (d, $^3J_{HH}$ = 6.8 Hz, 3H); ^{13}C NMR (126 MHz, $CDCl_3$)

69a (major): 58.7 (CH_2), 41.2 (CH_2), 33.73/33.70 (CH_2), 30.8 (CH), 20.3 (CH_3); **69b** (minor): 65.9 (CH_2), 34.3 (CH_2), 33.73/33.70 (CH_2), 33.4 (CH), 21.9 (CH_3); MS (ESI): 184 ($[M+NH_4]^+$).

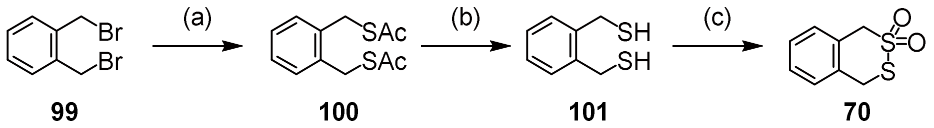

Scheme S13. (a) KSAc, 18-crown-6-ether, DMF, rt, under N_2, 12 h, 89%; (b) HCl, MeOH, rt, under N_2, 4 h, 82%; (c) H_2O_2, 0 °C to rt, 12 h, 31%.

Compound 100 (428 mg, 89%, red brown oil) was synthesized from **99** (500 mg, 1.89 mmol) following general procedure A. R_f (CH_2Cl_2): 0.47. Spectroscopic data were consistent with those reported in ref. S12.

Compound 101 (235 mg, 82%, smelly brown oil) was synthesized from **100** (428 mg, 1.68 mmol) following general procedure B. R_f (pentane/CH_2Cl_2 1:1): 0.62. Spectroscopic data were consistent with those reported in ref. S12.

Compound 70 (86.0 mg, 31%, colorless solid) was synthesized from **101** (235 mg, 1.38 mmol) following general procedure C. R_f (pentane/CH_2Cl_2 4:1): 0.35. Spectroscopic data were consistent with those reported in ref. S13.

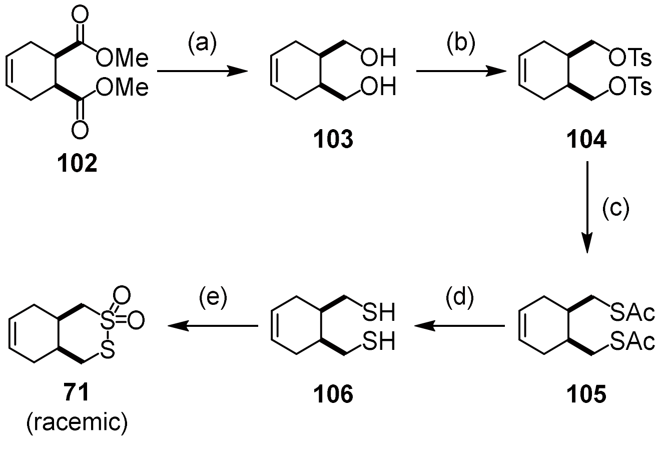

Scheme S14. (a) $LiAlH_4$, dry THF, 0 °C to reflux, 1.5 h, 81%; (b) TsCl, pyridine, 0 °C to rt, 12 h, 100%; (c) KSAc, 18-crown-6-ether, DMF, rt, under N_2, 12 h, 45%; (d) HCl, MeOH, rt, under N_2, 4 h, 76%; (e) H_2O_2, 0 °C to rt, 12 h, 7%.

Compound 102 was synthesized and purified according to procedures described in ref. S14.

Compound 103 was synthesized and purified according to procedures described in ref. S15.

Compound 104 was synthesized and purified according to procedures described in ref. S16.

Compound 105 (582 mg, 45%, colorless oil) was synthesized from **104** (1.3 g, 5.03 mmol) following general procedure A. R_f (CH_2Cl_2): 0.46; IR (neat): 3026 (w), 2906 (w), 2840 (w), 1690 (s), 1426 (w), 1354 (w), 1135 (m), 1108 (m), 959 (m), 663 (w), 628 (m); ^{1}H NMR (400 MHz, $CDCl_3$): 5.63 – 5.56 (m, 2H), 3.00 (dd, $^2J_{HH}$ = 13.5, $^3J_{HH}$ = 4.8 Hz, 2H), 2.81 (dd, $^2J_{HH}$ = 13.5, $^3J_{HH}$ = 8.8 Hz, 2H), 2.33 (s, 6H), 2.22 – 2.10 (m, 2H), 2.01 – 1.86 (m, 4H); ^{13}C NMR (101 MHz, $CDCl_3$): 195.9 (C), 125.3 (CH), 36.8 (CH), 30.8 (CH_3), 30.0 (CH_2), 28.8 (CH_2); MS (ESI): 141 ($[M\text{-}SAc\text{-}Ac+H]^+$), 107 ($[M\text{-}2SAc\text{-}H]^+$), 175 ($[M\text{-}2Ac+3H]^+$).

Compound 106 (299 mg, 76%, smelly colorless oil) was synthesized from **105** (582 mg, 2.25 mmol) following general procedure B. R_f (pentane/CH_2Cl_2 9:1): 0.34; IR (neat): 3339 (br w), 2931 (s), 2856 (m), 2538 (w), 1742 (br w), 1445 (m), 1343 (w), 1316 (w), 1217 (w), 1119 (w), 1019 (m), 713 (w); ^{1}H NMR (400 MHz, $CDCl_3$): 5.65 – 5.58 (m, 2H), 2.54 (ddd, $^2J_{HH}$ = 12.8, $^3J_{HH}$ = 7.6, 4.9 Hz, 2H), 2.42 (ddd, $^2J_{HH}$ = 12.8, $^3J_{HH}$ = 8.4, 8.4 Hz, 2H), 2.27 – 2.12 (m, 2H), 2.09 – 1.95 (m, 4H), 1.33 (dd, $^3J_{HH}$ = 8.4, 7.6 Hz, 2H); ^{13}C NMR (101 MHz, $CDCl_3$): 125.4 (CH), 39.9 (CH), 28.3 (CH_2), 25.2 (CH_2). MS (ESI): 141 ($[M\text{-}SAc\text{-}Ac+H]^+$), 107 ($[M\text{-}2SAc\text{-}H]^+$).

Compound 71 (25 mg, 7%, colorless oil) was synthesized from **106** (299 mg, 1.72 mmol) in AcOH (4.0 mL) following general procedure C. R_f (CH_2Cl_2): 0.49; IR (neat): 3033 (w), 2973 (w), 2916 (w), 2881 (w), 2846 (w), 1655 (w), 1443 (w), 1417 (w), 1397 (w), 1374 (w), 1325 (w), 1303 (s), 1245 (w), 1222 (m), 1197 (w), 1180 (w), 1163 (w), 1120 (s), 1052 (w), 1027 (w),

985 (w), 955 (w), 935 (w), 898 (w), 863 (w), 817 (w), 767 (w), 744 (w), 671 (m), 653 (m); ^{1}H NMR (400 MHz, $CDCl_3$): 5.78 – 5.71 (m, 1H), 5.65 – 5.59 (m, 1H), 3.76 (dd, $^{2}J_{HH}$ = 14.3, $^{3}J_{HH}$ = 2.5 Hz, 1H), 3.46 (dd, $^{2}J_{HH}$ = 13.5, $^{3}J_{HH}$ = 13.0 Hz, 1H), 3.05 (dd, $^{2}J_{HH}$ = 14.3, $^{3}J_{HH}$ = 3.8 Hz, 1H), 3.00 (dd, $^{2}J_{HH}$ = 13.5, $^{3}J_{HH}$ = 3.0 Hz, 1H), 2.88 – 2.81 (m, 1H), 2.74 – 2.63 (m, 1H), 2.60 – 2.50 (m, 1H), 2.44 – 2.36 (m, 1H), 2.05 – 1.95 (m, 1H), 1.90 – 1.81 (m, 1H); ^{13}C NMR (101 MHz, $CDCl_3$): 125.3 (CH), 123.4 (CH), 60.2 (CH_2), 40.3 (CH_2), 34.8 (CH), 31.7 (CH_2), 30.1 (CH), 23.7 (CH_2); MS (ESI): 222 ($[M+NH_4]^+$).

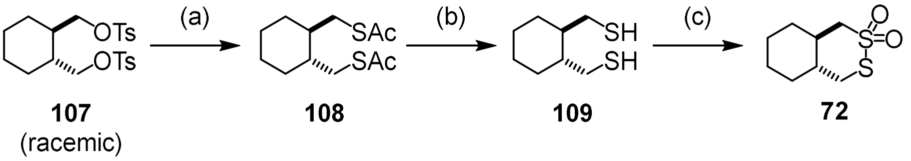

Scheme S15. (a) KSAc, 18-crown-6-ether, DMF, rt, under N_2, 12 h, 72%; (b) HCl, MeOH, rt, under N_2, 4 h, 54%; (c) H_2O_2, 0 °C to rt, 12 h, 34%.

Compound 107 was synthesized and purified according to procedures described in ref. S17.

Compound 108 (496 mg, 72%, yellow oil) was synthesized from **107** (1.2 g, 2.7 mmol) following general procedure A. R_f (pentane/CH_2Cl_2 1:3): 0.31; IR (neat): 2925 (m), 2854 (w), 1691 (s), 1446 (w), 1424 (w), 1354 (w), 1135 (m), 1108 (w), 954 (w), 628 (w); ^{1}H NMR (400 MHz, $CDCl_3$): 3.24 (dd, $^{2}J_{HH}$ = 13.6, $^{3}J_{HH}$ = 2.9 Hz, 2H), 2.83 – 2.74 (m, 2H), 2.34 (s, 6H), 1.86 – 1.76 (m, 2H), 1.73 – 1.61 (m, 2H), 1.46 – 1.35 (m, 2H), 1.27 – 1.12 (m, 2H), 1.09 – 0.95 (m, 2H); ^{13}C NMR (101 MHz, $CDCl_3$): 195.8 (C), 41.2 (CH), 33.2 (CH_2), 31.5 (CH_2), 30.8 (CH_3), 25.8 (CH_2); MS (ESI): 143 ($[M-SAc-Ac+H]^+$).

Compound 109 (180 mg, 54%, colorless oil) was synthesized from **108** (496 mg, 1.90 mmol) following general procedure B. R_f (pentane/CH_2Cl_2 9:1): 0.26. Spectroscopic data were identical to those reported in ref. S18.

Compound 72 (71 mg, 34%, colorless solid) was synthesized from **109** (180 mg, 1.02 mmol) following general procedure C. R_f (pentane/CH_2Cl_2 1:4): 0.53; Mp: 80 – 81 °C; IR (neat): 2954 (w), 2921 (m), 2854 (w), 1445 (w), 1398 (w), 1332 (w), 1302 (m), 1286 (m), 1236 (w), 1178 (w), 1110 (s), 1074 (m), 953 (w), 929 (w), 887 (w), 837 (w), 814 (w), 775 (m), 711 (w); ^{1}H NMR (400 MHz, $CDCl_3$): 3.30 (dd, $^2J_{HH}$ = 14.3, $^3J_{HH}$ = 11.5 Hz, 1H), 3.24 – 3.16 (m, 2H), 2.88 (dd, $^2J_{HH}$ = 14.3, $^3J_{HH}$ = 2.9 Hz, 1H), 2.17 – 2.05 (m, 1H), 1.89 – 1.74 (m, 3H), 1.69 – 1.54 (m, 2H), 1.42 – 1.04 (m, 4H); ^{13}C NMR (101 MHz, $CDCl_3$): 64.7 (CH_2), 42.4 (CH), 42.0 (CH), 40.1 (CH_2), 33.5 (CH_2), 32.1 (CH_2), 26.2 (CH_2), 25.0 (CH_2); MS (ESI): 207 ([M+H]$^+$).

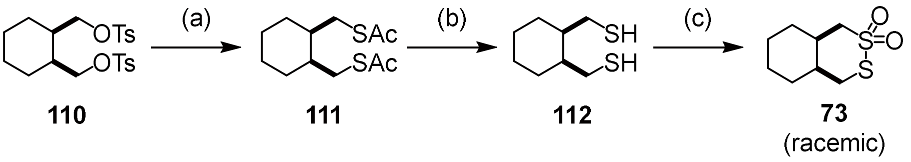

Scheme S16. (a) KSAc, 18-crown-6-ether, DMF, rt, under N_2, 12 h, 80%; (b) HCl, MeOH, rt, under N_2, 4 h, 91%; (c) H_2O_2, 0 °C to rt, 12 h, 30%.

Compound 110 was synthesized and purified according to procedures described in ref. S19.

Compound 111 (484 mg, 80%, yellow oil) was synthesized from **110** (1.05 g, 2.32 mmol) following general procedure A. R_f (pentane/CH_2Cl_2 1:3): 0.36; IR (neat): 2926 (m), 2857 (w), 1690 (s), 1450 (w), 1419 (w), 1354 (w), 1255 (w), 1135 (m), 1109 (m), 957 (m), 746 (w), 666 (w), 630 (m); ^{1}H NMR (400 MHz, $CDCl_3$): 2.93 (dd, $^2J_{HH}$ = 13.5, $^3J_{HH}$ = 5.7 Hz, 2H), 2.87 (dd, $^2J_{HH}$ = 13.5, $^3J_{HH}$ = 8.9 Hz, 2H), 2.33 (s, 6H), 1.87 – 1.78 (m, 2H), 1.59 – 1.41 (m, 6H), 1.39 – 1.27 (m, 2H); ^{13}C NMR (101 MHz, $CDCl_3$): 195.9 (C), 39.3 (CH), 30.8 (CH_3), 29.8 (CH_2), 28.1 (CH_2), 23.2 (CH_2); MS (ESI): 143 ([M-SAc-Ac+H]$^+$).

Compound 112 (299 mg, 91%, colorless oil) was synthesized from **111** (484 mg, 1.86 mmol) following general procedure B. R_f (pentane/CH_2Cl_2 9:1): 0.47. Spectroscopic data were identical to those reported in ref. S18.

Compound 73 (106 mg, 30%, colorless solid) was synthesized from **112** (299 mg, 1.70 mmol) in AcOH (2 mL) following general procedure C. R_f (pentane/CH_2Cl_2 1:4): 0.44; Mp: 94 – 95 °C; IR (neat): 2983 (w), 2930 (s), 2860 (m), 1451 (w), 1415 (w), 1397 (w), 1371 (w), 1346 (w), 1302 (s), 1247 (m), 1167 (w), 1125 (s), 1051 (m), 978 (w), 953 (w), 926 (w), 907 (w), 888 (w), 817 (m), 778 (m), 660 (w), 620 (w); ^{1}H NMR (400 MHz, $CDCl_3$): 3.66 (br s, 1H), 3.62 (dd, $^2J_{HH}$ = 13.0, $^3J_{HH}$ = 13.0 Hz, 1H), 3.21 – 2.85 (m, 2H), 2.72 (br d, $^3J_{HH}$ = 13.0 Hz, 1H), 2.15 – 2.00 (m, 1H), 1.99 – 1.78 (m, 2H), 1.77 – 1.66 (m, 1H), 1.62 – 1.50 (m, 2H), 1.49 – 1.13 (m, 3H); ^{13}C NMR (101 MHz, $CDCl_3$): 59.2 (CH_2), 41.3 (CH_2), 37.0 (CH), 34.1 (CH), 32.0 (CH_2), 25.4 (CH_2), 24.4 (CH_2), 20.6 (CH_2); MS (ESI): 430 ([2M+NH_4]$^+$), 224 ([M+NH_4]$^+$).

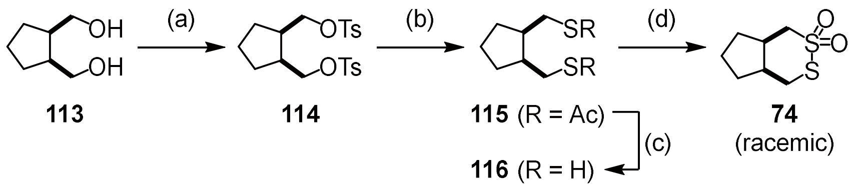

Scheme S17. (a) TsCl, pyridine, $CHCl_3$, rt, 0 °C to rt, 12 h, 66%; (b) KSAc, 18-crown-6-ether, DMF, rt, under N_2, 12 h, 72%; (c) HCl, MeOH, rt, under N_2, 4 h, 54%; (d) H_2O_2, 0 °C to rt, 12 h, 34%.

Compound 113 was synthesized and purified according to procedures described in ref. S20.

Compound 114. Tosyl chloride (1.4 g, 7.4 mmol) was dissolved in pyridine (3.3 mL) at 0 °C. A solution of **113** (241 mg, 1.85 mmol) in $CHCl_3$ (1.0 mL) was added, and the resulting mixture was warmed up to room temperature and stirred for 2 h. The mixture was poured onto ice, and 1 M HCl aq. was added until the pH was confirmed to be lower than 5. The mixture was extracted with CH_2Cl_2, and the organic layer was dried over Na_2SO_4, filtered, and concentrated. The residue was purified by flash column chromatography (SiO_2, pentane/CH_2Cl_2 1:1 to CH_2Cl_2, R_f (CH_2Cl_2): 0.33) to afford **114** (533 mg, 66%) as a colorless solid. Mp: 137 – 138 °C; IR (neat): 2961 (w), 1598 (w), 1494 (w), 1455 (w), 1360 (s), 1308 (w), 1293 (w), 1175

(s), 1121 (w), 1097 (w), 1020 (w), 950 (s), 815 (m), 786 (w), 666 (m); ^{1}H NMR (500 MHz, $CDCl_3$): 7.76 (d, $^3J_{HH}$ = 8.2 Hz, 4H), 7.35 (d, $^3J_{HH}$ = 8.2 Hz, 4H), 3.96 (dd, $^2J_{HH}$ = 9.8, $^3J_{HH}$ = 6.6 Hz, 2H), 3.90 (dd, $^2J_{HH}$ = 9.8, $^3J_{HH}$ = 6.6 Hz, 2H), 2.46 (s, 6H), 2.35 – 2.24 (m, 2H), 1.81 – 1.69 (m, 2H), 1.69 – 1.58 (m, 1H), 1.58 – 1.44 (m, 1H), 1.40 – 1.26 (m, 2H); ^{13}C NMR (126 MHz, $CDCl_3$): 145.1 (C), 133.0 (C), 130.1 (CH), 128.0 (CH), 70.6 (CH_2), 40.5 (CH), 28.4 (CH_2), 23.1 (CH_2), 21.8 (CH_3); MS (ESI): 267 ($[M-OTs]^+$), 456 ($[M+NH_4]^+$).

Compound 115 (154 mg, 51%, yellow oil) was synthesized from **114** (533 mg, 1.22 mmol) following general procedure A. R_f (pentane/CH_2Cl_2 1:3): 0.36; IR (neat): 2954 (w), 2872 (w), 1689 (s), 1424 (w), 1354 (w), 1134 (m), 1109 (m), 957 (m), 747 (w), 628 (m); ^{1}H NMR (400 MHz, $CDCl_3$): 2.98 (dd, $^2J_{HH}$ = 13.2, $^3J_{HH}$ = 5.7 Hz, 2H), 2.79 (dd, $^2J_{HH}$ = 13.2, $^3J_{HH}$ = 9.1 Hz, 2H), 2.33 (s, 6H), 2.18 – 2.06 (m, 2H), 1.83 – 1.64 (m, 3H), 1.64 – 1.50 (m, 1H), 1.49 – 1.36 (m, 2H); ^{13}C NMR (101 MHz, $CDCl_3$): 195.9 (C), 42.6 (CH), 30.8 (CH_3), 30.3 (CH_2), 29.9 (CH_2), 22.3 (CH_2); MS (ESI): 95 ($[M-2SAc-H]^+$), 129 ($[M-SAc-Ac+H]^+$).

Compound 116 (39 mg, 38%, colorless oil) was synthesized from **115** (154 mg, 0.625 mmol) following general procedure B. R_f (pentane/CH_2Cl_2 1:1): 0.61; IR (neat): 2952 (s), 2869 (m), 2552 (w), 1453 (w), 1307 (w), 1274 (w), 1247 (w), 720 (w); ^{1}H NMR (400 MHz, $CDCl_3$): 2.61 (ddd, $^2J_{HH}$ = 12.9, $^3J_{HH}$ = 7.5, 5.7 Hz, 2H), 2.36 (ddd, $^2J_{HH}$ = 12.9, $^3J_{HH}$ = 9.0, 7.5 Hz, 2H), 2.17 – 2.06 (m, 2H), 1.82 (m, 2H), 1.74 – 1.54 (m, 2H), 1.54 – 1.43 (m, 2H), 1.34 (dd, $^3J_{HH}$ = 7.5, 7.5 Hz, 2H); ^{13}C NMR (101 MHz, $CDCl_3$): 46.2 (CH), 30.0 (CH_2), 25.0 (CH_2), 22.3 (CH_2); MS (ESI): 95 ($[M-2SAc-H]^+$).

Compound 74 (25.2 mg, 56%, colorless oil) was synthesized from **116** (39 mg, 0.24 mmol) in AcOH (0.30 mL) following general procedure C. R_f (pentane/CH_2Cl_2 1:4): 0.33; IR (neat): 2946 (w), 2876 (w), 1460 (w), 1421 (w), 1400 (w), 1362 (w), 1305 (s), 1264 (m), 1215 (w), 1172 (w), 1135 (m), 1113 (s), 1035 (w), 1014 (w), 985 (w), 962 (w), 927 (w), 887 (w), 844 (w), 794 (m), 671 (w), 614 (w); ^{1}H NMR (400 MHz, $CDCl_3$): 3.80 (dd, $^2J_{HH}$ = 14.6, $^3J_{HH}$ = 3.3 Hz,

1H), 3.22 (dd, ${}^{2}J_{HH}$ = 14.6, ${}^{3}J_{HH}$ = 4.4 Hz, 1H), 3.19 – 3.11 (m, 2H), 2.88 – 2.78 (m, 1H), 2.35 – 2.26 (m, 1H), 2.00 – 1.86 (m, 2H), 1.86 – 1.70 (m, 3H), 1.63 – 1.53 (m, 1H); ^{13}C NMR (101 MHz, $CDCl_3$): 58.9 (CH_2), 41.5 (CH), 36.9 (CH_2), 36.2 (CH), 31.0 (CH_2), 25.6 (CH_2), 21.5 (CH_2); MS (ESI): 215 ([M+Na]$^+$).

2.2. Synthesis of Bis-Thiosulfonates

2.2.1. Synthesis of DTE-Thiosulfonate Derivatives

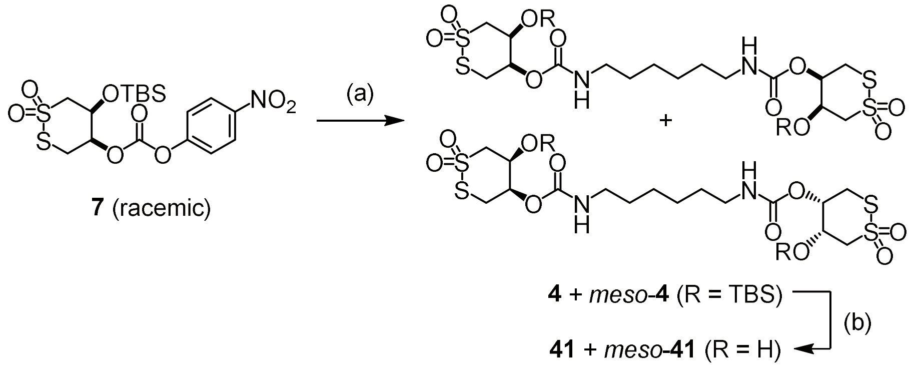

Scheme S18. (a) $H_2N(CH_2)_6NH_2$ (**8**), *i*-Pr_2NEt, DMF, rt, 2 h, 89%; (b) CH_2Cl_2, TFA, MeOH, rt, 125 h, 80%.

Compounds 4 and *meso*-4. Hexamethylenediamine (**8**, 38.7 mg, 0.333 mmol, azeotropically dried with toluene) and *i*-Pr_2NEt (0.21 mL, 1.21 mmol) were dissolved in DMF (3.0 mL). The stock solution (1.0 mL) was added dropwise to a solution of **7** (92.4 mg, 0.199 mmol) in DMF (1.0 mL) at room temperature. After the mixture was stirred for 2 h, CH_2Cl_2 and sat. Na_2CO_3 aq. were added. The resulting mixture was extracted with CH_2Cl_2 (x3) and the combined organic layers were washed with sat. Na_2CO_3 aq. (x3), H_2O and brine, dried over Na_2SO_4, filtered, and concentrated. The residue was purified by flash column chromatography (SiO_2, 1 g, CH_2Cl_2/acetone 100:1 to 20:1, R_f (CH_2Cl_2/acetone 20:1): 0.23) to afford a mixture of **4** and ***meso*-4** (68.0 mg, 89%, ratio not determined) as a colorless solid. IR (neat): 3245 (w, br), 2931 (m), 2858 (w), 1728 (w), 1695 (m), 1560 (m), 1469 (w), 1314 (m), 1258 (m), 1203 (m), 1114 (s), 981 (w), 914 (w), 837 (s), 781 (s), 749 (m), 670 (m); ^{1}H NMR (400 MHz, $CDCl_3$):

5.27 – 5.19 (m, 2H), 4.97 – 4.84 (br m, 2H), 4.33 (ddd, $^3J_{HH}$ = 10.8, 3.6, 3.4 Hz, 2H), 3.65 (dd, $^2J_{HH}$ = 13.0, $^3J_{HH}$ = 10.8 Hz, 2H), 3.54 – 3.45 (m, 4H), 3.33 (dd, $^2J_{HH}$ = 13.0, $^3J_{HH}$ = 3.6 Hz, 2H), 3.24 – 3.12 (m, 4H), 1.58 – 1.48 (m, 4H), 1.40 – 1.30 (m, 4H), 0.88 (s, 18H), 0.12 (s, 12H); ^{13}C NMR (101 MHz, $CDCl_3$): 155.3 (C), 70.1 (CH), 67.0 (CH), 62.1 (CH_2), 41.1 (CH_2), 35.4 (CH_2), 29.9 (CH_2), 26.3 (CH_2), 25.7 (CH_3), 18.0 (C), -4.75 (CH_3), -4.78 (CH_3); HRMS (ESI, +ve) calcd for $C_{28}H_{56}N_2O_{10}S_4Si_2$ ([M+H]$^+$): 765.2430, found: 765.2412.

Compounds 41 and ***meso*-41**. To a solution of a mixture of **4** and ***meso*-4** (7.7 mg, 10 μmol) in CH_2Cl_2 (0.63 mL) and MeOH (63 μL) at room temperature, TFA (0.31 mL) was added. The mixture was stirred for 5 days, concentrated, and azeotropically dried with MeOH. The residue was purified by flash column chromatography (SiO_2, 1 g, CH_2Cl_2 to CH_2Cl_2/MeOH 10:1, R_f (CH_2Cl_2/MeOH 10:1): 0.25) to afford a mixture of **41** and ***meso*-41** (4.3 mg, 80%, ratio not determined) as a colorless solid. IR (neat): 3384 (br, m), 2932 (m), 1699 (s), 1527 (m), 1409 (w), 1310 (s), 1241 (m), 1196 (w), 1122 (s), 1059 (s), 914 (w), 786 (m), 641 (w); ^{1}H NMR (400 MHz, CD_3OD): 5.27 – 5.17 (m, 2H), 4.37 – 4.25 (m, 2H), 3.67 (dd, $^2J_{HH}$ = 13.0, $^3J_{HH}$ = 11.0 Hz, 2H), 3.63 – 3.47 (m, 6H), 3.12 (t, $^3J_{HH}$ = 7.0 Hz, 4H), 1.64 – 1.46 (m, 4H), 1.43 – 1.31 (m, 4H); ^{13}C NMR (101 MHz, CD_3OD): 157.7 (C), 70.1 (CH), 68.6 (CH), 62.4 (CH_2), 41.7 (CH_2), 36.2 (CH_2), 30.7 (CH_2), 27.3 (CH_2); HRMS (ESI, +ve) calcd for $C_{16}H_{28}N_2O_{10}S_4$ ([M+H]$^+$): 537.0700, found: 537.0688.

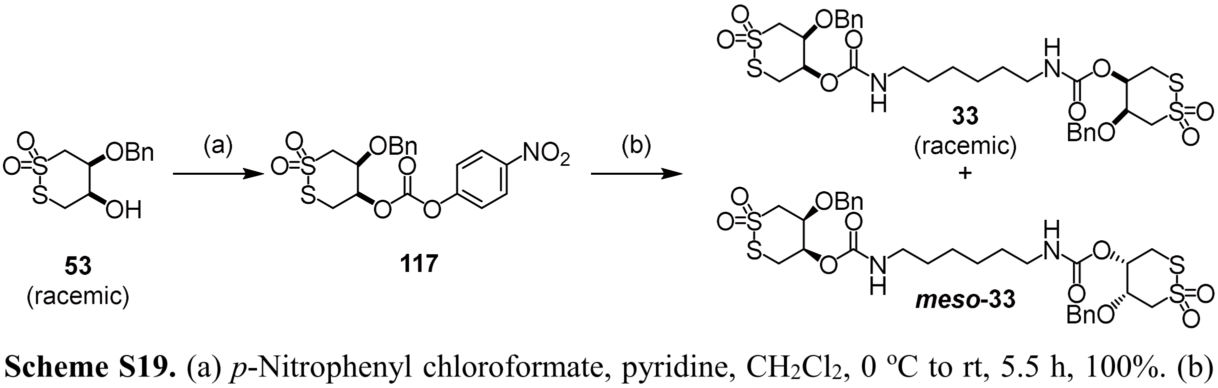

Scheme S19. (a) *p*-Nitrophenyl chloroformate, pyridine, CH_2Cl_2, 0 °C to rt, 5.5 h, 100%. (b) $H_2N(CH_2)_6NH_2$ (**8**), Et_3N, MeCN, rt, 2.5 h, 64%.

Compound 117. To a solution of **53** (45.2 mg, 0.165 mmol) in CH_2Cl_2 (1.7 mL) at 0 °C (ice bath), *p*-nitrophenylchloroformate (33.4 mg, 0.166 mmol) and pyridine (27 µL, 0.33 mmol) were added successively. The cooling bath was removed, and the mixture was stirred at room temperature for 3.5 h. Pyridine (0.11 mL, 1.32 mmol) and *p*-nitrophenylchloroformate (140 mg, 0.694 mmol) were added. The mixture was stirred at room temperature for further 2 h and passed through a pad of silica gel (5 g, CH_2Cl_2), and the filtrate was concentrated. The residue was purified by PTLC (CH_2Cl_2/acetone 50:1, R_f (CH_2Cl_2/acetone 50:1): 0.38) to afford **117** (72.2 mg, 100%) as a colorless foam. Mp: 140 – 141 °C (decomp.); IR (neat): 2934 (w), 1765 (s), 1617 (w), 1594 (m), 1523 (s), 1493 (m), 1456 (w), 1404 (w), 1348 (m), 1318 (m), 1224 (s), 1193 (s), 1132 (s), 1074 (m), 1010 (m), 914 (m), 859 (m), 733 (m), 697 (m), 647 (m); ^{1}H NMR (400 MHz, $CDCl_3$): 8.30 – 8.23 (m, 2H), 7.45 – 7.28 (m, 7H), 5.56 – 5.52 (m, 1H), 4.71 (d, $^{2}J_{HH}$ = 11.4 Hz, 1H), 4.67 (d, $^{2}J_{HH}$ = 11.4 Hz, 1H), 4.23 (ddd, $^{3}J_{HH}$ = 11.2, 3.9, 2.6 Hz, 1H), 3.80 (dd, $^{2}J_{HH}$ = 13.2, $^{3}J_{HH}$ = 11.2 Hz, 1H), 3.67 – 3.54 (m, 3H); ^{13}C NMR (101 MHz, $CDCl_3$): 155.3 (C), 152.0 (C), 145.8 (C), 136.4 (C), 129.0 (CH), 128.8 (CH), 128.2 (CH), 125.5 (CH), 121.9 (CH), 75.7 (CH), 72.4 (CH_2), 69.0 (CH), 59.6 (CH_2), 34.7 (CH_2).

Compounds 33 and ***meso*-33**. Hexamethylenediamine (**8**, 28.8 mg, 0.248 mmol, azeotropically dried with toluene) and Et_3N (115 µL, 0.83 mmol) were dissolved in MeCN (13.5 mL). The stock solution was added portionwise (6x0.20 mL) over 1.5 h to a solution of **117** (16.1 mg, 36.6 µmol) in MeCN (0.63 mL) at room temperature, and the mixture was stirred for additional 1 h. The mixture was concentrated, and the residue was purified by flash column chromatography (SiO_2, 0.5 g, CH_2Cl_2/MeOH 100:1 to 20:1, R_f (CH_2Cl_2/MeOH 30:1): 0.33) and PTLC (CH_2Cl_2/MeOH 30:1; CH_2Cl_2/acetone 10:1) to afford a mixture of **33** and ***meso*-33** (8.4 mg, 64%, ratio not determined) as a colorless solid. IR (neat): 3406 (w), 2927 (m), 1709 (s), 1521 (m), 1455 (w), 1404 (w), 1314 (s), 1237 (s), 1200 (m), 1125 (s), 1073 (s), 915 (m), 785 (m), 739 (m), 699 (m), 645 (w); ^{1}H NMR (400 MHz, $CDCl_3$): 7.51 – 7.29 (m, 10H), 5.56 – 5.44

(m, 2H), 4.94 (br t, $^3J_{HH}$ = 5.6 Hz, 2H), 4.71 (d, $^2J_{HH}$ = 11.5 Hz, 2H), 4.57 (d, $^2J_{HH}$ = 11.5 Hz, 2H), 4.10 (ddd, $^3J_{HH}$ = 11.2, 3.8, 2.6 Hz, 2H), 3.69 (dd, $^2J_{HH}$ = 12.1, $^3J_{HH}$ = 11.2 Hz, 2H), 3.60 – 3.34 (m, 6H), 3.19 (td, $^3J_{HH}$ = 6.8, 5.6 Hz, 4H), 1.56 – 1.43 (m, 4H), 1.39 – 1.29 (m, 4H). ^{13}C NMR (101 MHz, $CDCl_3$): 155.2 (C), 136.7 (C), 128.9 (CH), 128.6 (CH), 128.1 (CH), 75.8 (CH), 72.0 (CH_2), 64.1 (CH), 59.8 (CH_2), 41.1 (CH_2), 35.7 (CH_2), 29.7 (CH_2), 26.3 (CH_2). MS (ESI): 739 ($[M+Na]^+$).

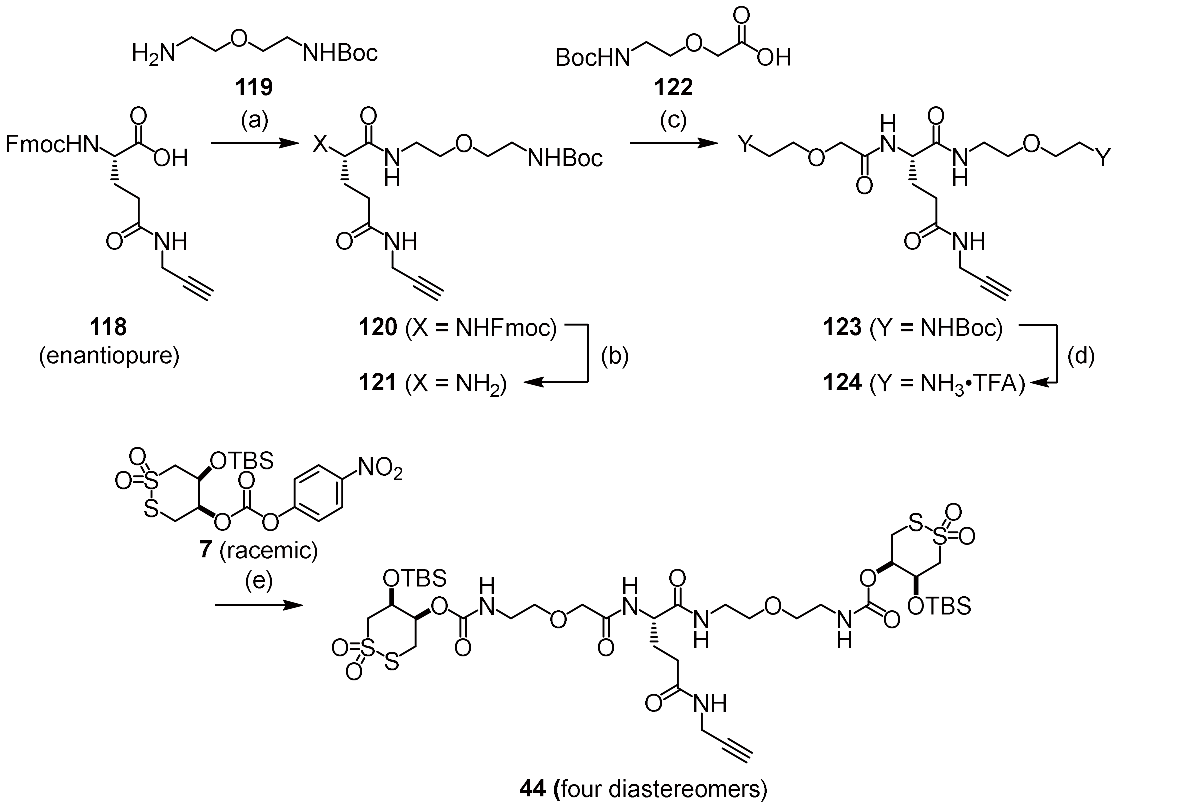

Scheme S20. (a) **119**, HATU, *i*-Pr_2NEt, DMF, rt, 1 h, 81%; (b) Me_2NH, THF, rt, 2 h, quant.; (c) **122**, HATU, *i*-Pr_2NEt, DMF, rt, 1 h, 77%; (d) TFA, CH_2Cl_2, 0 °C to rt, 1 h, 92% e) **7**, *i*-Pr_2NEt, DMF, rt, 2 h, 83%, dr 1:1:1:1.

Compound 118 was synthesized and purified according to procedures described in ref. S21.

Compound 119 was synthesized and purified according to procedures described in ref. S22.

Compound 120. To a solution of **118** (490 mg, 1.20 mmol) and HATU (456 mg, 1.20 mmol) in DMF (3.0 mL) at room temperature, *i*-Pr_2NEt (0.21 mL, 1.2 mmol) was added. After the resulting solution was stirred for 30 s, a solution of **119** (296 mg, 1.45 mmol) in DMF (3.0 mL) was added at room temperature. The reaction mixture was stirred for 1 h, diluted with EtOAc, and washed with sat. $NaHCO_3$ aq. (x3), 10% citric acid aq. (x3), H_2O, 5% LiCl aq. (x2), and brine. The organic layer was dried over Na_2SO_4, filtered, and concentrated. The residue was dissolved in a minimal amount of CH_2Cl_2, and the product was precipitated with pentane (x3). The solid was further purified by flash column chromatography (SiO_2, CH_2Cl_2 to CH_2Cl_2/MeOH 9:1, R_f (CH_2Cl_2/MeOH 20:1): 0.30) to afford **120** (575 mg, 81%) as a colorless solid. Mp: 144 – 145 °C; $[\alpha]_D^{20}$ +0.35 (c 0.50, MeOH); IR (neat) : 3296 (m), 2935 (w), 1687 (s), 1643 (s), 1529 (s), 1275 (s), 1248 (s), 1125 (s), 737 (m), 667 (s); ^1H NMR (400 MHz, $CDCl_3/CD_3OD$ 1:1): 7.71 (d, $^3J_{HH}$ = 7.5 Hz, 2H), 7.55 (d, $^3J_{HH}$ = 7.8 Hz, 2H), 7.35 (app t, $^3J_{HH}$ = 7.3 Hz, 2H), 7.30 – 7.22 (app t, $^3J_{HH}$ = 7.4 Hz, 2H), 4.40 – 4.27 (m, 2H), 4.15 (t, $^3J_{HH}$ = 6.9 Hz, 1H), 4.09 (dd, $^3J_{HH}$ = 8.2, 5.3 Hz, 1H), 4.01 – 3.88 (m, 2H), 3.49 – 3.40 (m, 4H), 3.39 – 3.31 (m, 2H), 3.20 (t, $^3J_{HH}$ = 5.1 Hz, 2H), 2.31 – 2.12 (m, 3H), 2.08 – 1.95 (m, 1H), 1.92 – 1.78 (m, 1H), 1.37 (s, 9H); ^{13}C NMR (101 MHz, $CDCl_3/CD_3OD$ 1:1): 172.9 (C), 172.0 (C), 156.7 (C), 156.5 (C), 143.8/143.7 (C), 141.34/141.31 (C), 127.8 (CH), 127.1 (CH), 125.1 (CH), 120.0 (CH), 79.5 (C), 79.4 (C), 71.4 (CH), 70.1 (CH_2), 69.3 (CH_2), 67.0 (CH_2), 54.1 (CH), 47.2 (CH), 40.3 (CH_2), 39.2 (CH_2), 32.0 (CH_2), 29.1 (2xCH_2), 28.4 (CH_3); MS (ESI): 615 ($[M+Na]^+$).

Compound 121. A solution of **120** (410 mg, 0.692 mmol) in a 2.0 M solution of Me_2NH in THF (5.1 mL) was stirred at room temperature for 2 h. The mixture was concentrated, and the residue was dissolved in a minimal amount of CH_2Cl_2 and then precipitated with pentane (x3) to afford **121** (257 mg, quant.) as a pale yellow oil, which was used for the next reaction without further purification. $[\alpha]_D^{20}$ +5.1 (c 0.50, MeOH); IR (neat): 3292 (m), 2975 (w), 2930 (w), 2872 (w), 1644 (s), 1525 (s), 1250 (s), 1167 (s), 1118 (s), 862 (w), 651 (s); ^1H NMR (400

MHz, $CDCl_3$): 7.64 (br s, 1H), 6.70 (br s, 1H), 5.10 (br s, 1H), 4.04 (dd, $^3J_{HH}$ = 5.3, $^4J_{HH}$ = 2.5 Hz, 2H), 3.60 – 3.40 (m, 7H), 3.35 – 3.32 (m, 2H), 2.37 (t, $^3J_{HH}$ = 6.7 Hz, 2H), 2.22 (t, $^4J_{HH}$ = 2.5 Hz, 1H), 2.10 – 1.84 (m, 2H), 1.45 (s, 9H). ^{13}C NMR (101 MHz, $CDCl_3$): 174.9 (C), 172.7 (C), 156.2 (C), 79.8 (C), 79.5 (C), 71.6 (CH), 70.2 (CH_2), 69.6 (CH_2), 54.2 (CH), 40.5 (CH_2), 38.9 (CH_2), 32.7 (CH_2), 31.3 (CH_2), 29.2 (CH_2), 28.6 (CH_3); MS (ESI): 393 ($[M+Na]^+$).

Compound 122 was synthesized and purified according to procedures described in ref. S23.

Compound 123. To a solution of **122** (178 mg, 0.812 mmol) and HATU (308 mg, 0.811 mmol) in DMF (1.7 mL) at room temperature, *i*-Pr_2NEt (0.14 mL, 0.81 mmol) was added. After the resulting solution was stirred for 30 s, a solution of **121** (249 mg, 0.672 mmol) in DMF (1.7 mL) was added at room temperature. The reaction mixture was stirred for 1 h, diluted with EtOAc, and washed with sat. $NaHCO_3$ aq. (x3), 10% citric acid aq. (x3), H_2O, 5% LiCl aq. (x2), and brine. The organic layer was dried over Na_2SO_4, filtered, and concentrated. The residue was dissolved in a minimal amount of CH_2Cl_2, and the product was precipitated with pentane (x3). The solid was further purified by flash column chromatography (SiO_2, CH_2Cl_2 to CH_2Cl_2/MeOH 9:1, R_f (CH_2Cl_2/MeOH 20:1): 0.20) to afford **123** (295 mg, 77%) as a colorless solid. Mp: 51 – 52 °C; $[\alpha]_D^{20}$ -1.8 (c 1.00, MeOH); IR (neat): 3304 (m), 2976 (w), 1650 (s), 1525 (s), 1248 (s), 1166 (s), 1118 (s), 864 (w), 644 (m); ^{1}H NMR (400 MHz, $CDCl_3$): 8.17 (d, $^3J_{HH}$ = 6.8 Hz, 1H), 7.17 (t, $^3J_{HH}$ = 5.5 Hz, 1H), 6.73 (br s, 1H), 5.79 (br s, 1H), 5.43 (br s, 1H), 4.45 – 4.37 (m, 1H), 4.06 (dd, $^3J_{HH}$ = 5.3, $^4J_{HH}$ = 2.6 Hz, 2H), 3.99 (s, 2H), 3.59 – 3.53 (m, 2H), 3.53 – 3.45 (m, 4H), 3.45 – 3.33 (m, 4H), 3.28 (br s, 2H), 2.57 – 2.45 (m, 1H), 2.44 – 2.33 (m, 1H), 2.23 (t, $^4J_{HH}$ = 2.6 Hz, 1H), 2.15 – 2.07 (m, 2H), 1.43 (s, 9H), 1.42 (s, 9H); ^{13}C NMR (101 MHz, $CDCl_3$): 173.2 (C), 171.1 (C), 170.6 (C), 156.4 (C), 156.3 (C), 79.5 (3xC), 71.9 (CH), 71.3 (CH_2), 70.5 (CH_2), 70.3 (CH_2), 69.3 (CH_2), 52.7 (CH), 40.6 (2xCH_2), 39.4 (CH_2), 32.4 (CH_2), 29.6 (CH_2), 28.57 (CH_3), 28.56 (CH_3), 27.8 (CH_2); MS (ESI): 595 ($[M+Na]^+$).

Compound 124. To a solution of **123** (274 mg, 0.479 mmol) in CH_2Cl_2 (2.4 mL) at 0 ˚C, TFA (2.4 mL) was added. The reaction mixture was allowed to warm to room temperature, and stirred for 1 h. The reaction mixture was concentrated, and the residue was dissolved in a minimal amount of CH_2Cl_2 and precipitated with Et_2O (x3) to afford **124** (266 mg, 93%) as an orange oil, which was used for the next reaction without further purification. $[\alpha]_D^{20}$ +3.2 (c 0.50, MeOH); IR (neat): 3268 (m), 3059 (m), 2926 (m), 1643 (s), 1530 (s), 1177 (s), 1123 (s), 835 (m), 799 (m), 721 (s); ^{1}H NMR (400 MHz, CD_3OD): 4.33 (dd, $^3J_{HH}$ = 8.3, 5.0 Hz, 1H), 4.11 (d, $^2J_{HH}$ = 14.9 Hz, 1H) , 4.07 (d, $^2J_{HH}$ = 14.9 Hz, 1H), 3.98 (dd, $^2J_{HH}$ = 17.6, $^4J_{HH}$ = 2.6 Hz, 1H), 3.92 (dd, $^2J_{HH}$ = 17.6, $^4J_{HH}$ = 2.6 Hz, 1H), 3.82 – 3.76 (m, 2H), 3.71 – 3.65 (m, 2H), 3.58 (t, $^3J_{HH}$ = 5.4 Hz, 2H), 3.50 – 3.36 (m, 2H), 3.24 (t, $^3J_{HH}$ = 5.0 Hz, 2H), 3.19 – 3.06 (m, 2H), 2.62 (t, $^4J_{HH}$ = 2.6 Hz, 1H), 2.47 – 2.27 (m, 2H), 2.16 – 1.93 (m, 2H); ^{13}C NMR (101 MHz, CD_3OD): 175.0 (C), 174.0 (C), 172.1 (C), 162.5 (q, $^2J_{CF}$ = 36.0 Hz, TFA anion), 118.0 (q, $^1J_{CF}$ = 292 Hz, TFA anion), 80.5 (C), 72.4 (CH), 70.9 (CH_2), 70.6 (CH_2), 68.2 (CH_2), 67.6 (CH_2), 54.6 (CH), 40.6 (2xCH_2), 40.1 (CH_2), 32.6 (CH_2), 29.7 (CH_2), 28.2 (CH_2); MS (ESI): 372 ($[M-2TFA-H]^+$).

Compounds 44. To a solution of **124** (43.5 mg, 72.6 µmol) and **7** (69.1 mg, 0.149 mmol) in DMF (0.73 µL) at room temperature, *i*-Pr_2NEt (54 µL, 0.305 mmol) was added. The reaction was stirred for 2 h, diluted with EtOAc, and washed with sat. $NaHCO_3$ aq. (x3), 5% LiCl aq. (x2), and brine. The organic layer was dried over Na_2SO_4, filtered, and concentrated. The residue was purified by flash column chromatography (SiO_2, CH_2Cl_2 to CH_2Cl_2/MeOH 9:1, R_f (CH_2Cl_2/MeOH 9:1): 0.40) to afford **44** (61.2 mg, 83%, a 1:1:1:1 mixture of four diastereomers) as a colorless foam. IR (neat): 3302 (m), 2953 (m), 2859 (m), 1719 (s), 1660 (s), 1522 (s), 1318 (s), 1255 (s), 1111 (s), 839 (s), 733 (s); ^{1}H NMR (400 MHz, $CDCl_3$): 8.29 – 7.91 (m, 1H), 7.40 – 7.06 (m, 1H), 6.78 – 6.44 (m, 2H), 6.42 – 6.13 (m, 1H), 5.38 – 5.11 (m, 2H), 4.53 – 4.24 (m, 3H), 4.17 – 3.87 (m, 4H), 3.76 – 3.23 (m, 20H), 2.65 – 2.48 (m, 1H), 2.45 – 2.32 (m, 1H), 2.29 – 2.20 (m, 1H), 2.19 – 2.01 (m, 2H), 0.93 – 0.83 (m, 18H), 0.16 – 0.04 (m, 12H); ^{13}C NMR

(101 MHz, $CDCl_3$): 173.6/173.5/173.4 (C), 171.14/171.07 (C), 170.7/170.6/170.4/170.3 (C), 155.7 (2xC), 79.51/79.45/79.39/79.37 (C), 72.2/72.1 (CH), 70.9/70.73/70.71 (CH_2), 70.4 (CH_2), 70.23/70.20 (2xCH), 70.0/69.9 (CH_2), 69.2 (CH_2), 66.9/66.8/66.7 (2xCH), 61.9 (2xCH_2), 52.9/52.8 (CH), 41.14/41/09 (CH_2), 39.33/39.25 (2xCH_2), 35.50/35.45/35.41 (2xCH_2), 32.6/32.49/32.46 (CH_2), 29.6 (CH_2), 27.8/27.7/27.5/27.4 (CH_2), 25.6 (2xCH_3), 18.0 (2xC), -4.72 – -4.88 (4xCH_3); MS (ESI): 1042 ($[M+Na]^+$).

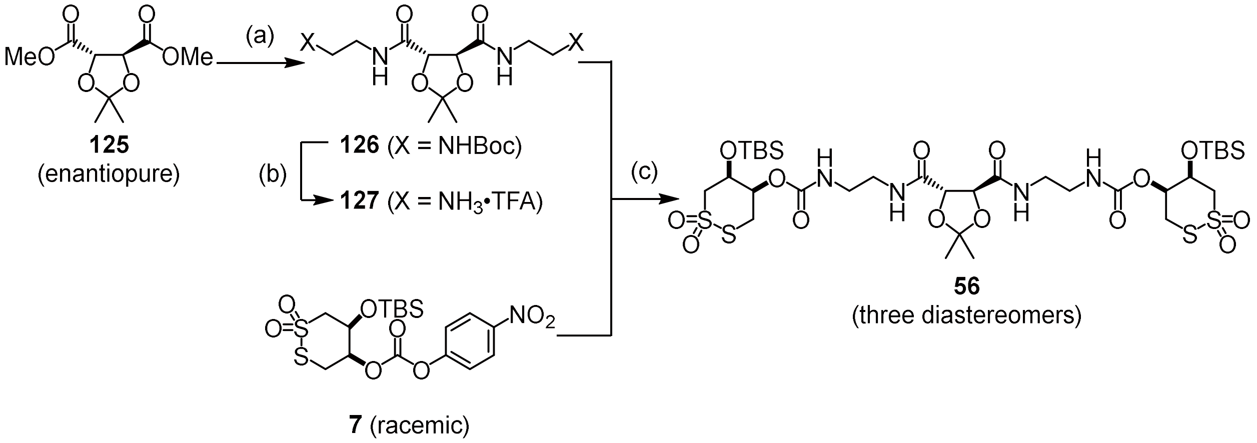

Scheme S21. (a) *N*-Boc-Ethylenediamine, neat, rt, 22 h, 62%; (b) TFA, CH_2Cl_2, rt, 1.5 h; (c) *i*-Pr_2NEt, DMF, rt, 3 h, 48%.

Compound 126. Compound **125** (0.37 mL, 2.0 mmol) and *N*-Boc-ethylenediamine (0.63 mL, 4.00 mmol) were mixed at room temperature and stirred for 22 h to form a solid, which was purified by flash column chromatography (10 g, CH_2Cl_2 to CH_2Cl_2/MeOH 10:1, R_f (CH_2Cl_2/MeOH 10:1): 0.25) to afford **126** (585 mg, 62%) as a colorless solid. Mp: 58 – 59 °C (decomp.); $[\alpha]_D^{20}$ + 3.01 (c 1.00, $CHCl_3$); IR (neat): 3336 (m), 2986 (w), 2941 (w), 1665 (s), 1521 (s), 1366 (m), 1454 (w), 1366 (m), 1248 (s), 1163 (s), 1119 (m), 1083 (m), 993 (m), 861 (m), 782 (w), 731 (w), 669 (w); ^{1}H NMR (400 MHz, $CDCl_3$): 7.34 (br s, 2H), 4.95 (br s, 2H), 4.50 (s, 2H), 3.50 – 3.21 (m, 8H), 1.49 (s, 6H), 1.43 (s, 18H); ^{13}C NMR (101 MHz, $CDCl_3$): 170.4 (C), 156.6 (C), 112.6 (C), 79.8 (C), 77.6 (CH), 40.4 (CH_2), 40.2 (CH_2), 28.5 (CH_3), 26.3 (CH_3); MS (ESI): 475 ($[M+H]^+$).

Compounds 56. To a solution of **126** (21.0 mg, 44.2 μmol) in CH_2Cl_2 (0.22 mL) at room temperature, TFA (0.22 mL) was added. The mixture was stirred for 1.5 h and concentrated to give crude ammonium **127**, which was dissolved in DMF (0.44 mL). *i*-Pr_2NEt (46 μL, 0.26 mmol) and **7** (43.1 mg, 93.0 μmol) were added to the solution, and the resulting mixture was stirred for 3 h. The mixture was diluted with a 5:1 mixture of CH_2Cl_2 and EtOH, washed with sat. Na_2CO_3 aq. (x3), dried over Na_2SO_4, filtered, and concentrated. The residue was purified by flash column chromatography (SiO_2, 1 g, CH_2Cl_2 to CH_2Cl_2/MeOH 50:1, R_f (CH_2Cl_2/MeOH 10:1): 0.43). Fractions containing the desired products were concentrated, and the residue was dissolved in a 5:1 mixture of CH_2Cl_2 and EtOH, washed with sat. Na_2CO_3 aq. (x2), H_2O and brine, dried over Na_2SO_4, filtered, and concentrated. The residue was triturated in Et_2O (x2) and pentane to afford **56** (19.7 mg, 48%, a mixture of three diastereomers, ratio not determined) as a colorless solid. IR (neat): 3357 (br w), 2934 (w), 2859 (w), 1717 (m), 1682 (m), 1524 (m), 1468 (w), 1361 (w), 1317 (m), 1253 (s), 1205 (m), 1111 (s), 1082 (s), 986 (w), 916 (w), 839 (s), 780 (s), 750 (m), 672 (w); ^{1}H NMR (400 MHz, $CDCl_3$): 7.24 – 7.01 (br m, 2H), 5.55 (br s, 2H), 5.22 (s, 2H), 4.53 – 4.43 (m, 2H), 4.39 – 4.28 (m, 2H), 3.76 – 3.61 (m, 2H), 3.59 – 3.21 (m, 14H), 1.50 (s, 6H), 0.87 (s, 18H), 0.12 (s, 12H); ^{13}C NMR (101 MHz, $CDCl_3$): 170.5 – 170.3 (C), 155.9 – 155.7 (C), 113.0 – 112.8 (C), 77.4 (CH), 70.1 (CH), 67.5 – 67.2 (CH), 62.0 (CH_2), 41.1/40.9 (CH_2), 39.8/39.5 (CH_2), 35.3 (CH_2), 26.3 (CH_3), 25.7 (CH_3), 18.1 (C), -4.75 (CH_3), -4.78 (CH_3); MS (ESI): 945 ($[M+Na]^+$).

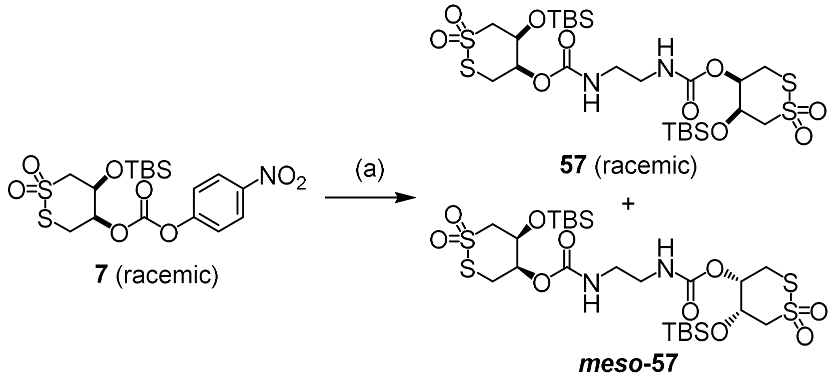

Scheme S22. (a) $H_2N(CH_2)_2NH_2$, *i*-Pr_2NEt, DMF, rt, 1 h, 53%.

Compounds 57 and ***meso*-57**. Ethylenediamine (12 μL, 0.18 mmol) and *i*-Pr_2NEt (0.11 mL, 0.63 mmol) were dissolved in DMF (2.3 mL). The stock solution (0.26 mL) was added dropwise to a solution of **7** (48.3 mg, 0.104 mmol) in DMF (0.26 mL) at room temperature. After the mixture was stirred for 1 h, CH_2Cl_2 and sat. Na_2CO_3 aq. were added. The resulting mixture was extracted with CH_2Cl_2, and the combined organic layers were washed with sat. Na_2CO_3 aq. (x3), H_2O and brine, dried over Na_2SO_4, filtered, and concentrated. The residue was purified by flash column chromatography (SiO_2, 1 g, CH_2Cl_2 to CH_2Cl_2/acetone 30:1, R_f (CH_2Cl_2/acetone 30:1): 0.41) to afford a mixture of **57** and ***meso*-57** (19.7 mg, 53%, ratio not determined) as a colorless solid. IR (neat): 3288 (w), 2932 (w), 2859 (w), 1713 (s), 1518 (w), 1469 (w), 1401 (m), 1353 (w), 1316 (m), 1255 (m), 1238 (m), 1201 (m), 1124 (s), 1059 (m), 1006 (w), 980 (m), 914 (w), 839 (s), 779 (s), 750 (m), 672 (w); ^{1}H NMR (400 MHz, $CDCl_3$): 5.30 – 5.16 (m, 4H), 4.34 (ddd, $^3J_{HH}$ = 10.9, 3.3, 3.3 Hz, 2H), 3.63 (dd, $^2J_{HH}$ = 12.8, $^3J_{HH}$ = 10.9 Hz, 2H), 3.55 – 3.40 (m, 4H), 3.41 – 3.27 (m, 6H), 0.879/0.876 (s, 18H), 0.13 (s, 6H). ^{13}C NMR (101 MHz, $CDCl_3$): 155.7 (C), 70.0 (CH), 67.4 (CH), 62.1 (CH_2), 41.4 (CH_2), 35.3 (CH_2), 25.7 (CH_3), 18.1 (C), -4.73 (CH_3), -4.78 (CH_3); HRMS (ESI, +ve) calcd for $C_{24}H_{48}N_2O_{10}S_4Si_2$ ($[M+Na]^+$): 709.1803, found: 709.1794.

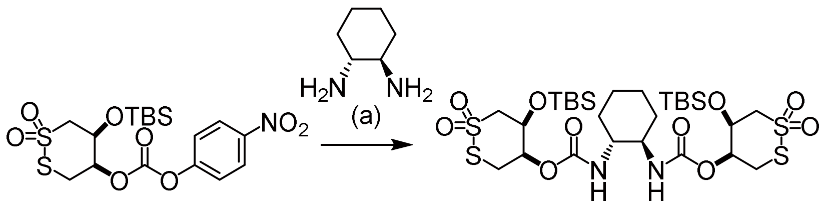

7 (racemic)

58
(three diastereomers)

Scheme S23. (a) (1*R*,2*R*)-1,2-Diaminocyclohexane, *i*-Pr_2NEt, DMF, 5 h, 57%.

Compounds 58. To a solution of (1*R*,2*R*)-1,2-diaminocyclohexane (7.07 mg, 61.9 μmol) and *i*-Pr_2NEt (43 μL, 0.25 mmol) in DMF (1.2 mL) at room temperature, **7** (61.3 mg, 0.132 nnol) was added. After the mixture was stirred for 5 h, sat. Na_2CO_3 aq. was added and the mixture was extracted with CH_2Cl_2 (x3). The combined organic layers were washed with sat. Na_2CO_3 aq. (x2), H_2O and brine, dried over Na_2SO_4, filtered, and concentrated. The residue was purified by flash column chromatography (SiO_2, 1 g, pentane/CH_2Cl_2 1:1 to CH_2Cl_2 to CH_2Cl_2/acetone 20:1, R_f (CH_2Cl_2/acetone 20:1): 0.36). Fractions containing the desired products were concentrated, and the residue was triturated in a 1:2 mixture of Et_2O and pentane (x3) to afford **58** (27.0 mg, 57%, a mixture of three diastereomers, ratio not determined) as a colorless solid. IR (neat): 3384 (br w), 2931 (m), 2858 (w), 1714 (m), 1519 (w), 1467 (w), 1360 (w), 1317 (m), 1255 (m), 1232 (m), 1201 (m), 1139 (m), 1112 (s), 1082 (m), 1007 (w), 916 (w), 838 (s), 779 (s), 750 (m), 672 (m); ^{1}H NMR (400 MHz, $CDCl_3$): 5.37 – 4.81 (m, 4H), 4.39 – 4.27 (m, 2H), 3.72 – 3.15 (m, 10H), 2.21 – 1.92 (m, 2H), 1.76 (br s, 2H), 1.46 – 1.11 (m, 4H), 0.89 – 0.85 (m, 18H), 0.12 (s, 12H); ^{13}C NMR (101 MHz, $CDCl_3$): 155.7/155.4 (C), 70.0 (CH), 67.3 (CH), 62.4 – 62.0 (CH_2), 55.8/55.1 (CH), 35.3 (CH_2), 32.7 (CH_2), 25.8/25.7/25.6 (CH_3), 24.7 (CH_2), 18.0 (C), -4.52/-4.73/-4.79 (2xCH_3); MS (ESI): 1543 ($[2M+NH_4]^+$), 780 ($[M+NH_4]^+$).

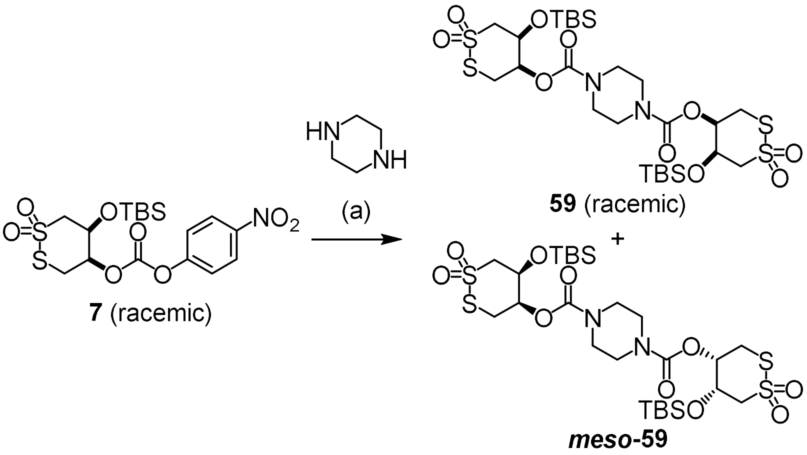

Scheme S24. (a) Piperazine, *i*-Pr_2NEt, DMF, 2.5 h, 88%.

Compounds 59 and ***meso*-59**. To a solution of piperazine (7.02 mg, 81.5 µmol) and *i*-Pr_2NEt (57 µL, 0.33 mmol) in DMF (1.6 mL) at room temperature, **7** (77.6 mg, 0.167 mmol) was added. After the mixture was stirred for 2.5 h, sat. Na_2CO_3 aq. was added and the mixture was extracted with CH_2Cl_2 (x3). The combined organic layers were washed with sat. Na_2CO_3 aq. (x2), H_2O and brine, dried over Na_2SO_4, filtered, and concentrated. The residue was triturated in MeOH (x2) and dried by lyophilization to afford a mixture of **59** and ***meso*-59** (52.6 mg, 88%, ratio not determined) as a colorless solid. IR (neat): 2930 (w), 2858 (w), 1690 (m), 1458 (m), 1429 (m), 1351 (w), 1314 (m), 1283 (w), 1254 (w), 1231 (s), 1201 (m), 1142 (m), 1108 (s), 1083 (s), 998 (m), 962 (w), 916 (m), 907 (m), 858 (w), 837 (s), 796 (m), 779 (s), 746 (s), 698 (w), 672 (w); ^{1}H NMR (400 MHz, $CDCl_3$): 5.30 – 5.22 (m, 2H), 4.37 (ddd, $^3J_{HH}$ = 10.8, 3.8, 2.8 Hz, 2H), 3.71 – 3.42 (m, 14H), 3.42 – 3.32 (m, 2H), 0.869/0.866 (s, 18H), 0.14 – 0.11 (m, 6H); ^{13}C NMR (101 MHz, $CDCl_3$): 154.0 (C), 70.0 (CH), 68.0 (CH), 62.3 (CH_2), 43.9/43.7 (CH_2), 35.4 (CH_2), 25.7 (CH_3), 18.0 (C), -4.69/-4.71/-4.80 (2xCH_3); MS (ESI): 1492 ($[2M+Na]^+$), 757 ($[M+Na]^+$).

2.2.2. Synthesis of Amino-Substituted Cyclic Thiosulfonates

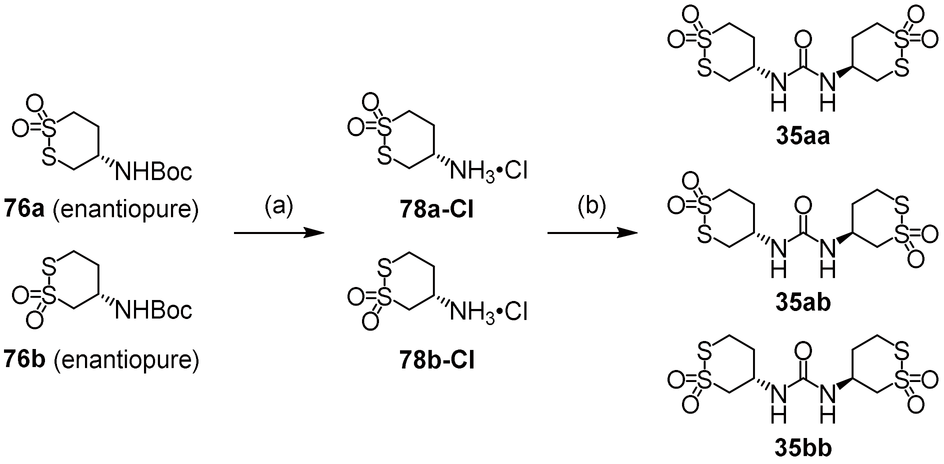

Scheme S25. (a) HCl, 1,4-dioxane, rt, 63 h; (b) triphosgene, Et_3N, MeCN, rt, 2 h, 54% (2 steps), **35aa**:**35ab**:**35bb** = 100:20:~0.5.

Compounds 35. Compounds **76** (49.4 mg, 0.185 mmol, **76a**:**76b** = 6:1) were dissolved in a solution of HCl (4.0 M in 1,4-dioxane, 6.2 mL) at room temperature and the solution was stirred for 63 h. The mixture was concentrated and azeotropically dried with MeOH (x3) to give crude ammonium **78-Cl** (a mixture of **78a-Cl** and **78b-Cl**, ratio not determined), which was used for the next reaction without further purification. A stock solution of triphosgene (20.8 mg, 70.1 μmol) in MeCN (1.3 mL) was prepared. To a solution of the crude **78-Cl** and Et_3N (0.10 mL, 0.74 mmol) in MeCN (1.0 mL) at room temperature, the stock solution of triphosgene was added portionwise (6x0.14 mL, 6x7.5 μmol) over 1 h. After the mixture was stirred for additional 1 h, MeOH was added to the mixture, and the resulting solution was concentrated. The residue was purified by reverse phase flash column chromatography (SNAP Ultra C18 12 g, Eluent: H_2O + 0.1% TFA/CH_3CN + 0.1% TFA gradient from 100:0 to 60:40). Fractions containing the desired products were lyophilized to afford **35** (18.0 mg, 54% over 2 steps, **35aa**:**35ab**:**35bb** = 100:20:~0.5) as a colorless solid. IR (neat): 3381 (w), 3387 (br w), 2924 (w), 1668 (m), 1628 (m), 1550 (s), 1407 (w), 1309 (s), 1291 (s), 1256 (m), 1226 (w), 1201 (w), 1124 (s), 1067 (m), 1047 (m), 1022 (m), 1003 (m), 910 (w), 892 (w), 825 (w), 800 (w), 764 (w), 714 (m), 627 (w); ^{1}H NMR (500 MHz, DMSO-d_6) **35aa**: 6.53 (d, $^{3}J_{HH}$ = 8.0 Hz, 2H), 4.11

– 4.01 (m, 2H), 3.75 – 3.65 (m, 2H), 3.59 (ddd, $^2J_{HH}$ = 14.0, $^3J_{HH}$ = 8.4, 3.7 Hz, 2H), 3.51 – 3.41 (m, 2H), 3.31 (dd, $^2J_{HH}$ = 14.0, $^3J_{HH}$ = 7.7 Hz, 2H), 2.33 – 2.16 (m, 4H); **35ab** (some peaks are deduced from ^{1}H integration and structural similarity with **35aa**): 6.70 (d, $^3J_{HH}$ = 7.2 Hz, 1H), 6.34 (d, $^3J_{HH}$ = 8.8 Hz, 1H), 4.24 – 4.15 (m, 1H), 4.11 – 4.01 (m, 1H), 3.74 – 3.65 (m, 2H), 3.64 – 3.53 (m, 1H), 3.50 – 3.41 (m, 3H), 3.35 – 3.27 (m, 1H), 3.22 (ddd, $^2J_{HH}$ = 14.0, $^3J_{HH}$ = 11.6, 2.2 Hz, 1H), 2.33 – 2.16 (m, 3H), 1.84 – 1.74 (m, 1H); **35bb** (all peaks are overlapped with **35aa** or/and **35ab** and deduced from structural similarity with **35ab**): 6.34 (d, $^3J_{HH}$ = 8.8 Hz, 2H), 4.24 – 4.15 (m, 2H), 3.73 – 3.67 (m, 2H), 3.49 – 3.41 (m, 4H), 3.25 – 3.18 (m, 2H), 2.24 – 2.16 (m, 2H), 1.84 – 1.74 (m, 2H); ^{13}C NMR (126 MHz, DMSO-d_6) **35aa**: 156.1 (C), 57.4 (br, CH_2), 43.2 (br, CH), 37.2 (br, CH_2), 29.54 (CH_2); **35ab**: 155.7 (C), 63.4 (CH_2), 57.4 (CH_2), 47.6 (CH), 43.2 (CH), 37.2 (CH_2), 31.52 (CH_2), 31.4 (CH_2), 31.2 (CH_2); **35bb** (two peaks are overlapped or not detected): 63.5 (CH_2), 47.7 (CH), 31.47 (CH_2); MS (ESI): 378 ($[M+NH_4]^+$).

2.3. Synthesis of Tris-Thiosulfonates

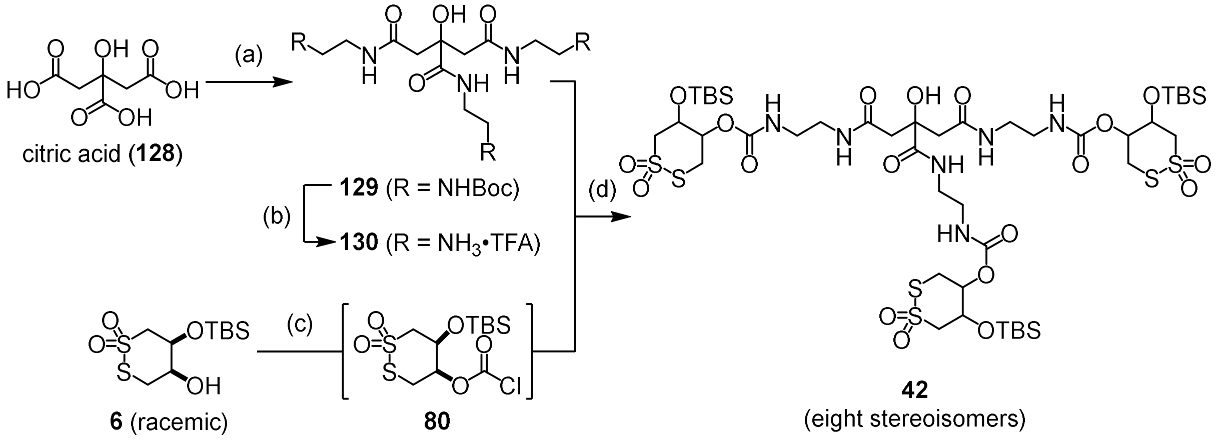

Scheme S26. (a) *N*-Boc-Ethylenediamine, EDCI·HCl, DMAP, DMF, rt, 74 h, 76% (b) TFA, CH_2Cl_2, rt, 22.5 h; (c) triphosgene, pyridine, CH_2Cl_2, rt, 4 h; (d) Et_3N, MeCN/DMF, rt, 1.5 h, 7.1% (2 steps).

Compound 129. To a solution of citric acid (**128**, 384 mg, 2.00 mmol) in DMF (10 mL) at room temperature, *N*-Boc-ethylenediamine (1.1 mL, 7.2 mmol), DMAP (885 mg, 7.24 mmL) and EDCI·HCl (1.40 g, 7.30 mmol) were added successively. The mixture was stirred for 74 h and concentrated. The residue was dissolved in CH_2Cl_2, and the solution was washed with 10% citric acid aq. and brine, dried over Na_2SO_4, filtered, and concentrated. The residue was purified by recrystallization (CH_2Cl_2/MeOH/Et_2O) to afford **129** (946 mg, 76%) as a colorless solid. Mp: 168 – 169 °C; IR (neat): 3360 (m), 2981 (w), 2940 (w), 1688 (s), 1646 (m), 1527 (s), 1446 (m), 1390 (w), 1367 (w), 1322 (w), 1278 (s), 1247 (s), 1172 (s), 979 (m), 869 (w), 782 (w), 717 (w), 634 (w); ^{1}H NMR (400 MHz, DMSO-d_6): 8.03 – 7.95 (br m, 2H), 7.91 – 7.85 (br m, 1H), 6.79 – 6.70 (br m, 3H), 6.19 (br s, 1H), 3.14 – 2.89 (m, 12H), 2.52 (overlapped with DMSO, 2H), 2.41 (d, $^2J_{HH}$ = 14.3 Hz, 1H), 1.37 (s, 27H); ^{13}C NMR (101 MHz, DMSO-d_6): 173.8 (C), 170.1 (C), 155.64 (C), 155.55 (C), 77.7 (C), 77.6 (C), 74.7 (C), 43.0 (CH_2), 39.4 (CH_2), 39.3 (CH_2), 38.9 (CH_2), 38.6 (CH_2), 28.2 (2xCH_3); MS (ESI): 619 ([M+H]$^+$).

Compounds 42. To a suspension of **129** (63.9 mg, 0.103 mmol) in CH_2Cl_2 (0.50 mL) at room temperature, TFA (0.50 mL) was added. After the mixture was stirred for 22.5 h, Et_2O was added to form a precipitate. The supernatant was removed, and the solid was triturated in Et_2O (x2) and dried by lyophilization to give crude ammonium **130**. The residue and Et_3N (84 μL, 0.60 mmol) were dissolved in DMF (0.45 mL) and used for the next reaction. To a solution of **6** (134 mg, 0.450 mmol) and pyridine (0.22 mL, 2.7 mmol) in CH_2Cl_2 (4.5 mL) at 0 °C, triphosgene (136 mg, 0.458 mmol) was added. The mixture was warmed up to room temperature, stirred for 4 h, and concentrated to give crude chloroformate **80**. The residue was dissolved in MeCN (1.1 mL), and the solution of **130** and Et_3N was added in five portions over 1.5 h at room temperature. The mixture was stirred for additional 15 min. MeOH was added to the mixture and the resulting solution was concentrated. The residue was purified by flash column chromatography (SiO_2, 10 g, CH_2Cl_2/MeOH 100:1 to 15:1; SiO_2, 2 g, CH_2Cl_2/MeOH

100:1 to 20:1, twice; SNAP Ultra C18 12 g, Eluent: H_2O + 0.1% TFA/CH_3CN + 0.1% TFA gradient from 60:40 to 5:95; SiO_2, 1 g, CH_2Cl_2 to CH_2Cl_2/MeOH 20:1, R_f (CH_2Cl_2/MeOH 10:1): 0.06) to afford **42** (9.5 mg, 7.1%, a mixture of eight stereoisomers, ratio not determined) as a colorless solid. IR (neat): 3362 (br w), 2931 (m), 2859 (m), 2490 (w), 1709 (m), 1654 (m), 1424 (m), 1358 (w), 1317 (m), 1253 (m), 1204 (w), 1133 (s), 1110 (s), 979 (m), 915 (m), 839 (s), 780 (s), 750 (m), 671 (m); ^1H NMR (400 MHz, CD_3OD): 5.34 – 5.22 (m, 3H), 4.50 – 4.35 (m, 3H), 3.73 – 3.63 (m, 3H), 3.63 – 3.53 (m, 6H), 3.53 – 3.43 (m, 3H), 3.43 – 3.04 (m, 12H), 2.76 – 2.62 (m, 2H), 2.60 – 2.48 (m, 2H), 0.90 (s, 27H), 0.19 – 0.11 (m, 18H); ^{13}C NMR (101 MHz, CD_3OD): 176.6 (C), 172.84/172.80/172.74/172.70 (C), 157.9/157.8/157.7 (2xC), 76.4/76.3 (C), 71.4/71.3 (2xCH), 69.0 – 68.5 (2xCH), 63.2 – 62.8 (2xCH_2), 44.7 – 44.2 (CH_2), 41.3 – 41.1 (2xCH_2), 40.6 – 40.2 (2xCH_2), 36.1 – 35.8 (2xCH_2), 26.2 – 26.0 (2xCH_3), 18.9 – 18.7 (2xC), -4.71 – -4.96 (4xCH_3); MS (ESI): 1291 ($[M+H]^+$).

2.4. Synthesis of Disulfinates and Inhibitor Controls

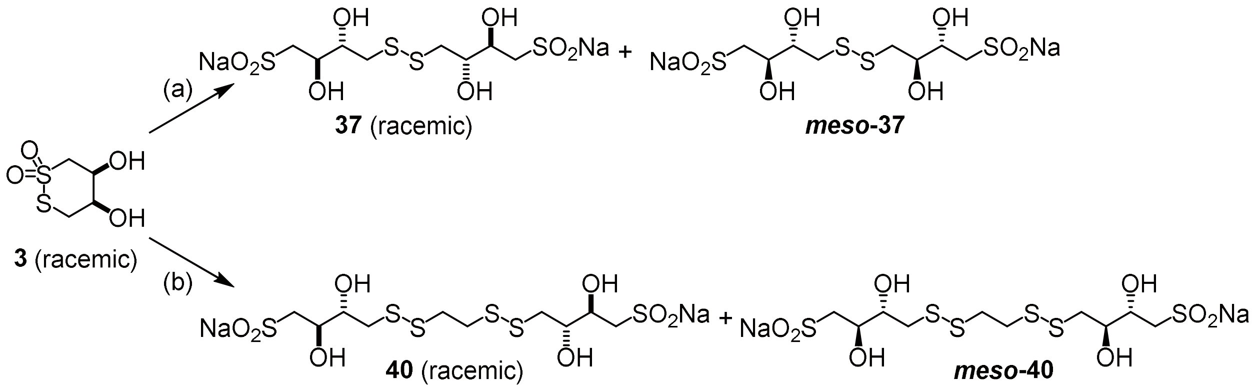

Scheme S27. (a) Dithioerythritol, NaOMe, MeOH, 0 °C, 10 min, 25%; (b) $HS(CH_2)_2SH$, NaOMe, MeOH, 0 °C, 10 min, 9.5%.

Compounds 37 and ***meso*-37**. To a solution of **3** (91.1 mg, 0.495 mmol) and dithioerythritol (26.1 mg, 0.169 mmol) in dry MeOH (1.3 mL) at 0 °C, a solution of NaOMe (freshly prepared from Na (8.1 mg, 0.35 mmol) and MeOH (0.33 mL)) was added. After the mixture was stirred for 10 min, dry Et_2O was added until no additional precipitate was formed.

The solid was collected and dissolved in a minimal amount of MeOH. Et_2O was carefully added until the solution became turbid. The precipitate was removed by centrifugation and the supernatant was transferred to another flask. Et_2O was added to the solution until precipitation was complete. The solid was collected, dissolved in MeOH, and precipitated with Et_2O. The solid was collected, triturated in Et_2O, and dried by lyophilization to afford a mixture of **37** and ***meso*-37** (26.4 mg, 25%, ratio not determined) as a colorless solid. The procedure was taken from ref. S2. IR (neat): 3258 (br m), 1649 (w), 1401 (w), 992 (s), 950 (s), 764 (w); ^{1}H NMR (400 MHz, CD_3OD): 4.09 – 3.98 (m, 2H), 3.79 – 3.70 (m, 2H), 3.104/3.096 (dd, $^2J_{HH}$ = 13.6, $^3J_{HH}$ = 3.1 Hz, 2H), 2.78/2.74 (dd, $^2J_{HH}$ = 13.6, $^3J_{HH}$ = 8.8 Hz, 2H), 2.54 (dd, $^2J_{HH}$ = 13.2, $^3J_{HH}$ = 2.9 Hz, 2H), 2.45 (dd, $^2J_{HH}$ = 13.2, $^3J_{HH}$ = 9.2 Hz, 2H); ^{13}C NMR (101 MHz, CD_3OD): 74.34/74.27 (CH), 71.0 (CH), 64.75/64.69 (CH_2), 44.0/43.6 (CH_2); MS (ESI): 184 ($[M-2Na]^{2-}$).

Compounds 40 and *meso*-40. To a solution of **3** (93.6 mg, 0.508 mmol) and 1,2-ethanedithiol (14 µL, 0.17 mmol) in dry MeOH (1.3 mL) at 0 °C, a solution of NaOMe (freshly prepared from Na (8.1 mg, 0.35 mmol) and MeOH (0.33 mL)) was added. After the mixture was stirred for 10 min, dry Et_2O was added until no additional precipitate was formed. The solid was collected and dissolved in a minimal amount of MeOH. Et_2O was carefully added until the solution became turbid. The precipitate was removed by centrifugation and the supernatant was transferred to another flask. Et_2O was added to the solution until precipitation was complete. The solid was collected, dissolved in MeOH, and precipitated with Et_2O. The solid was collected and dried by lyophilization to afford a mixture of **40** and ***meso*-40** (8.0 mg, 9.5%, ratio not determined) as a colorless solid. The procedure was taken from ref. S2. IR (neat): 3268 (br m), 1649 (m), 1413 (m), 994 (s), 936 (s), 857 (w), 730 (w); ^{1}H NMR (400 MHz, CD_3OD): 4.07 – 3.99 (m, 2H), 3.77 – 3.68 (m, 2H), 3.09 (dd, $^2J_{HH}$ = 13.7, $^3J_{HH}$ = 2.9 Hz, 2H), 3.06 (s, 4H), 2.77 (dd, $^2J_{HH}$ = 13.7, $^3J_{HH}$ = 8.9 Hz, 2H), 2.54 (dd, $^2J_{HH}$ = 13.1, $^3J_{HH}$ = 2.4 Hz, 2H), 2.45 (dd,

$^{2}J_{HH}$ = 13.1, $^{3}J_{HH}$ = 9.0 Hz, 2H); ^{13}C NMR (101 MHz, CD_3OD): 74.3 (CH), 71.0 (CH), 64.8 (CH_2), 44.2 (CH_2), 38.4 (CH_2); MS (ESI): 265 ($[M+H+Na]^{2+}$).

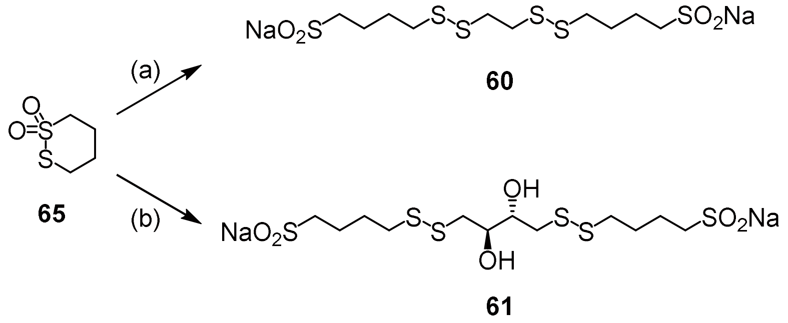

Scheme S28. (a) 1,2-Ethanedithiol, Na, MeOH, 0 °C to rt, under N_2, 42%; (a) dithioerythritol, Na, MeOH, 0 °C to rt, under N_2, 69%.

Compound 60 was synthesized and purified according to procedures described in ref. S24.

Compound 61 was synthesized and purified according to procedures described in ref. S25.

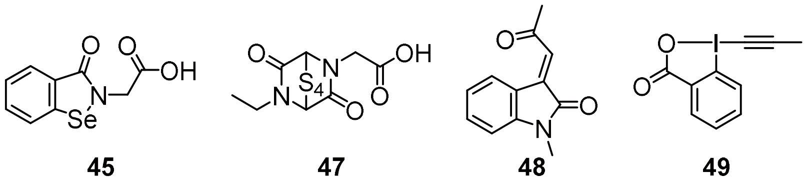

Figure S2. Structure of inhibitor and antiviral controls.

Compound 45 was synthesized and purified according to procedures described in ref. S26.

Compound 47 was synthesized and purified according to procedures described in ref. S27.

Compound 48 was synthesized and purified according to procedures described in ref. S28.

Compound 49 was synthesized and purified according to procedures described in ref. S29.

2.5. Synthesis of Transporters

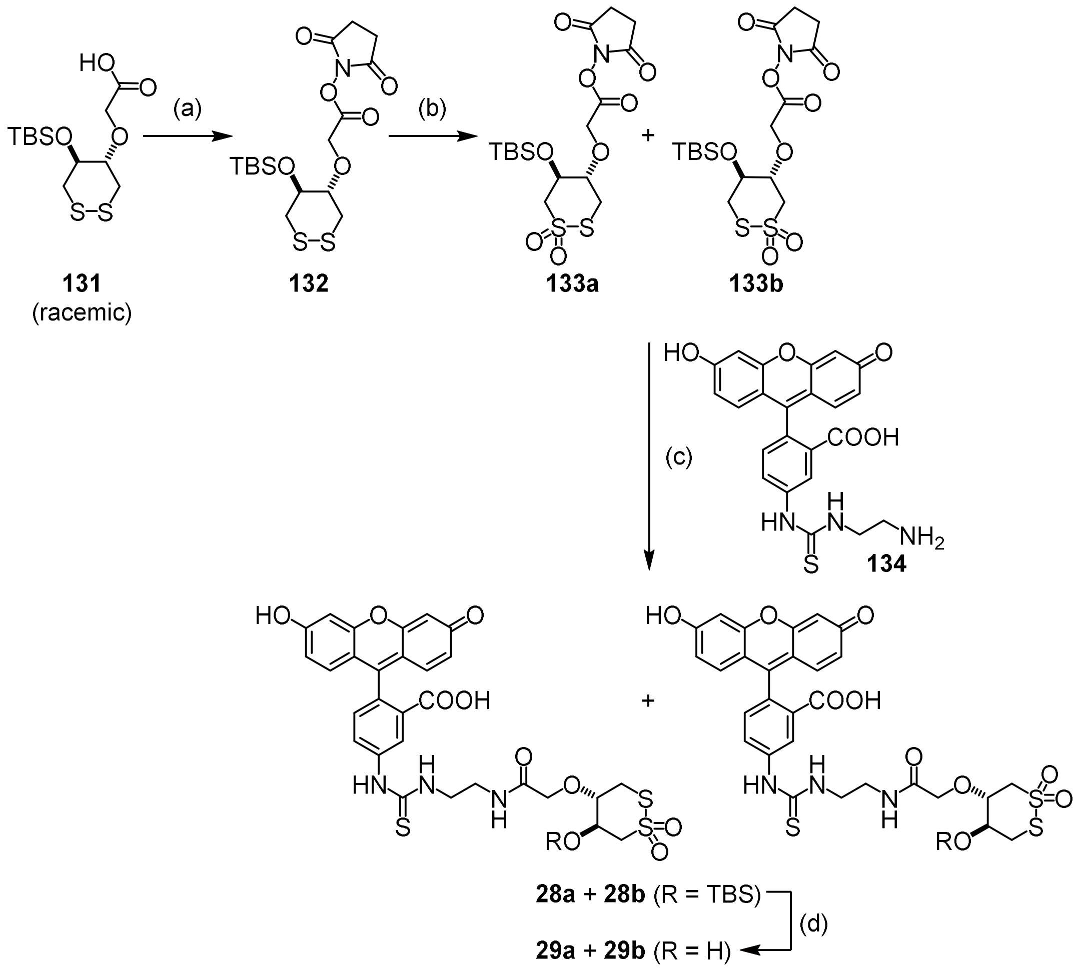

Scheme S29. (a) NHS, EDCI·HCl, CH_2Cl_2, rt, 12 h, 29%; (b) *m*-CPBA, rt, 2 d, 47%; (c) **134**, DMF, rt, 4 h, 31%, **28a**:**28b** = 3:2; (d) HCl, MeOH, rt, 1 h, 33%, **29a**:**29b** = 3:2.

Compound 131 (racemic) was synthesized and purified according to procedures described in ref. S30.

Compound 132. To a solution of NHS (68 mg, 0.59 mmol) in CH_2Cl_2 (6 mL) at room temperature, **131** (191 mg, 0.589 mmol) and EDCI·HCl (151 mg, 0.764 mmol) were added successively. The reaction mixture was stirred for 12 h, diluted with CH_2Cl_2, and washed with H_2O. The organic layer was dried over Na_2SO_4, filtered, and concentrated. The residue was purified by flash column chromatography (SiO_2, CH_2Cl_2, R_f (CH_2Cl_2): 0.35) to afford **132** (71 mg, 29%) as a colorless solid. Mp: 83 – 84 °C; IR (neat): 2949 (w), 2930 (w), 2857 (w), 1825

(w), 1785 (w), 1737 (s), 1469 (w), 1426 (w), 1381 (w), 1358 (w), 1253 (w), 1203 (m), 1148 (m), 1065 (s), 1000 (w), 953 (w), 884 (m), 852 (m), 838 (m), 777 (m), 751 (w), 731 (w), 669 (w), 644 (m), 615 (w); ^{1}H NMR (400 MHz, $CDCl_3$): 4.64 (d, $^2J_{HH}$ = 17.3 Hz, 1H), 4.57 (d, $^2J_{HH}$ = 17.3 Hz, 1H), 3.77 – 3.68 (m, 1H), 3.38 (ddd, $^3J_{HH}$ = 10.3, 8.2, 3.6 Hz, 1H), 3.32 (dd, $^3J_{HH}$ = 13.5, 3.6 Hz, 1H), 3.03 – 2.88 (m, 3H), 2.84 (s, 4H), 0.90 (s, 9H), 0.10 (s, 3H), 0.09 (s, 3H); ^{13}C NMR (101 MHz, $CDCl_3$): 168.8 (C), 166.5 (C), 84.5 (CH), 75.6 (CH), 67.5 (CH_2), 42.2 (CH_2), 39.3 (CH_2), 25.9 (CH_3), 25.7 (CH_2), 18.0 (C), -4.56 (CH_3), -4.66 (CH_3).

Compounds 133. To a solution of **132** (631 mg, 1.50 mmol) in CH_2Cl_2 (16 mL) at room temperature, *m*-CPBA (861 mg, 3.75 mmol) was added. After the reaction mixture was stirred for 2 days, the reaction mixture was diluted with CH_2Cl_2, and washed with sat. $NaHCO_3$ aq. (x3). The organic layer was dried over Na_2SO_4 and concentrated to afford crude thiosulfonates **133** (321 mg, 47%, **133a**:**133b** = 2.7:1) as a colorless solid, which was used for the next reaction without further purification. ^{1}H NMR (500 MHz, $CDCl_3$) **133a** (major): 4.69 (d, $^2J_{HH}$ = 17.2 Hz, 1H), 4.61 (d, $^2J_{HH}$ = 17.2 Hz, 1H), 4.26 (ddd, $^3J_{HH}$ = 9.7, 8.2, 4.4 Hz, 1H), 3.66 (ddd, $^3J_{HH}$ = 10.5, 8.2, 3.5 Hz, 1H), 3.60 – 3.49 (m, 3H), 3.35 (dd, $^2J_{HH}$ = 14.6, $^3J_{HH}$ = 10.4 Hz, 1H), 2.87 (s, 4H), 0.91 (s, 9H), 0.14 (s, 3H), 0.14 (s, 3H); **133b** (minor): 4.69 – 4.59 (m, 2H), 4.06 – 3.89 (m, 3H), 3.58 – 3.51 (m, 1H), 3.31 – 3.25 (m, 1H), 3.17 (br d, $^2J_{HH}$ = 12.9 Hz, 1H), 2.87 (s, 4H), 0.91 (s, 9H), 0.14 (s, 6H); ^{13}C NMR (126 MHz, $CDCl_3$) **133a** (major): 168.74 (C), 166.3 (C), 81.9 (CH), 71.9 (CH), 67.6 (CH_2), 65.7 (CH_2), 32.9 (CH_2), 25.75 (CH_3), 25.71 (CH_2), 17.9 (C), -4.64 (CH_3), -4.82 (CH_3); **133b** (minor): 168.67 (C), 166.0 (C), 80.7 (CH), 71.9 (CH), 67.4 (CH_2), 62.5 (CH_2), 35.8 (CH_2), 25.78 (CH_3), 25.71 (CH_2), 18.0 (C), -4.59 (CH_3), -4.71 (CH_3); MS (ESI): 471 ($[M+NH_4]^+$)

Compound 134 was synthesized and purified according to procedures described in ref. S27.

Compounds 28. To a solution of **133** (56.5 mg, 0.125 mmol, **133a**:**133b** = 2.7:1) in DMF (1.5 mL) at room temperature, **134** (56 mg, 0.13 mmol) was added. The reaction mixture was stirred for 4 h and concentrated. The residue was purified by reverse phase flash column chromatography (SNAP Ultra C18 30 g, eluent: CH_3CN + 0.1% TFA/ H_2O + 0.1% TFA gradient from 10:90 to 90:10). Fractions containing the desired products were lyophilized to afford **28** (30 mg, 31%, **28a**:**28b** = 3:2) as a yellow solid. IR (neat): 3291 (br w), 2932 (w), 2858 (w), 2614 (br w), 1721 (w), 1635 (m), 1595 (s), 1540 (s), 1456 (m), 1384 (m), 1313 (s), 1255 (s), 1202 (s), 1114 (s), 995 (w), 918 (w), 889 (w), 838 (m), 780 (m), 719 (w), 669 (w); ^{1}H NMR (500 MHz, CD_3OD) **28a** (major): 8.13 (s, 1H), 7.78 – 7.69 (m, 1H), 7.14 – 7.08 (m, 1H), 6.89 (br s, 2H), 6.78 (br s, 2H), 6.64 (br s, 2H), 4.43 – 4.38 (m, 1H), 4.23 – 4.13 (m, 2H), 3.99 – 3.78 (m, 2H), 3.77 – 3.70 (m, 1H), 3.73 – 3.67 (m, 1H), 3.71 – 3.67 (m, 1H), 3.58 – 3.49 (m, 2H), 3.52 – 3.44 (m, 1H), 3.39 (dd, $^2J_{HH}$ = 14.6, $^3J_{HH}$ = 8.4 Hz, 1H), 0.92 (s, 9H), 0.15 (s, 3H), 0.14 (s, 3H); **28b** (minor): 8.13 (s, 1H), 7.78 – 7.69 (m, 1H), 7.14 – 7.08 (m, 1H), 6.89 (br s, 2H), 6.78 (br s, 2H), 6.64 (br s, 2H), 4.24 – 4.15 (m, 3H), 3.99 – 3.93 (m, 1H), 3.99 – 3.78 (m, 2H), 3.89 – 3.83 (m, 1H), 3.78 – 3.71 (m, 1H), 3.68 – 3.61 (m, 1H), 3.58 – 3.49 (m, 2H), 3.19 (dd, $^2J_{HH}$ = 14.2, $^2J_{HH}$ = 7.3 Hz, 1H), 0.93 (s, 9H), 0.16 (s, 3H), 0.15 (s, 3H); ^{13}C NMR (126 MHz, CD_3OD) **28a** (major): 183.5 (C), 172.6/172.5 (C), 169.8 (C), 165.5 (C), 160.9 (C), 160.6 (C), 156.6 (C), 142.6 (C), 131.9 (CH), 130.6/130.5 (CH), 127.9 (CH), 122.7 (CH), 118.2 (C), 116.4 (CH), 113.9 (C), 103.4 (CH), 79.0 (CH), 72.5 (CH), 70.4 (CH_2), 64.8 (CH_2), 44.7 (CH_2), 40.5 (CH_2), 33.2 (CH_2), 26.18 (CH_3), 18.78 (C), -4.58 (CH_3), -4.79 (CH_3); **28b** (minor): 183.5 (C), 172.6/172.5 (C), 169.8 (C), 165.5 (C), 160.9 (C), 160.6 (C), 156.6 (C), 142.6 (C), 131.9 (CH), 130.6/130.5 (CH), 127.9 (CH), 122.7 (CH), 118.2 (C), 116.4 (CH), 113.9 (C), 103.4 (CH), 81.2 (CH), 70.6 (CH_2), 70.4 (CH), 60.3 (CH_2), 44.8 (CH_2), 40.2 (CH_2), 37.5 (CH_2), 26.22 (CH_3), 18.84 (C), -4.58 (CH_3), -4.72 (CH_3); HPLC-MS: R_t = 2.74, 788 ($[M+H]^+$).

Compounds 29. Thiosulfonates **28** (13 mg, 17 μmol) were dissolved in a solution of HCl (1.25 M in MeOH, 2.0 mL) at room temperature. The reaction mixture was stirred for 1 h and concentrated. The residue was purified by reverse phase flash column chromatography (SNAP Ultra C18 12 g, eluent: CH_3CN + 0.1% TFA/ H_2O + 0.1% TFA gradient from 10:90 to 90:10). Fractions containing the desired products were lyophilized to afford **29** (3.7 mg, 33%, **29a**:**29b** = 3:2) as a yellow solid. IR (neat): 3256 (br), 2925 (br), 1639 (m), 1601 (m), 1541 (m), 1451 (w), 1312 (m), 1176 (s), 1117 (s), 916 (w), 836 (w), 797 (w), 719 (w); ^{1}H NMR (500 MHz, CD_3OD) **29a** (major): 8.30 – 8.22 (br m, 1H), 7.88 (br s, 1H), 7.26 (d, $^{2}J_{HH}$ = 7.1 Hz, 1H), 7.02 (br s, 2H), 6.91 (s, 2H), 6.78 (br s, 2H), 4.27 – 4.15 (m, 3H), 3.98 – 3.76 (m, 2H), 3.79 – 3.72 (m, 1H), 3.78 – 3.71 (m, 1H), 3.64 – 3.56 (m, 1H), 3.63 – 3.45 (m, 2H), 3.57 – 3.51 (m, 1H), 3.29 – 3.20 (m, 1H); **29b** (minor): 8.30 – 8.22 (br m, 1H), 7.88 (br s, 1H), 7.26 (d, $^{2}J_{HH}$ = 7.1 Hz, 1H), 7.02 (br s, 2H), 6.91 (s, 2H), 6.78 (br s, 2H), 4.27 – 4.18 (m, 2H), 4.03 – 3.97 (m, 1H), 4.00 – 3.96 (m, 1H), 3.99 – 3.75 (m, 2H), 3.90 – 3.85 (m, 1H), 3.67 – 3.59 (m, 1H), 3.63 – 3.45 (m, 2H), 3.43 – 3.37 (m, 1H), 3.27 – 3.20 (m, 1H); ^{13}C NMR (126 MHz, CD_3OD) **29a** (major): 183.4 (C), 173.2/173.0 (C), 170.0 (C), 165.4 (C), 160.9 (C), 160.6 (C), 156.4 (C), 142.5 (C), 131.8 (CH), 130.8/130.3 (CH), 127.8 (CH), 122.5 (CH), 118.2 (C), 116.1 (CH), 113.7 (C), 103.4 (CH), 82.0 (CH), 71.1 (CH), 70.2/70.0 (CH_2), 65.9 (CH_2), 45.0 (CH_2), 39.8 (CH_2), 33.2 (CH_2); **29b** (minor): 183.4 (C), 173.2/173.0 (C), 170.0 (C), 165.4 (C), 160.9 (C), 160.6 (C), 156.4 (C), 142.5 (C), 131.8 (CH), 130.8/130.3 (CH), 127.8 (CH), 122.5 (CH), 118.2 (C), 116.1 (CH), 113.7 (C), 103.4 (CH), 81.4 (CH), 71.1 (CH), 70.2/70.0 (CH_2), 62.4 (CH_2), 45.0 (CH_2), 39.8 (CH_2), 36.3 (CH_2); HPLC-MS: R_t = 1.97, 674 (100, $[M+H]^+$).

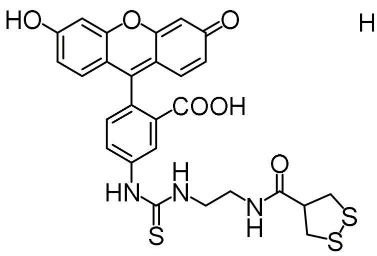

30

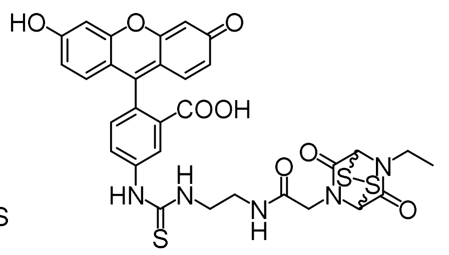

31

Figure S3. Structure of previously reported transporters.

Compound 30 was synthesized and purified according to procedures described in ref. S31.

Compound 31 (racemic) was synthesized and purified according to procedures described in ref. S32.

3. Dynamic Covalent Cascade Exchange Kinetics

3.1. ¹H NMR Spectroscopy

The general equation of reactions of thiosulfonates and disulfides (**A**) with a thiol (**B**) is shown as:

$$\mathrm{A + B} \underset{k_{-1}}{\overset{k_1}{\rightleftharpoons}} \mathrm{C} \quad or \quad \mathrm{A + B} \underset{k_{-1}}{\overset{k_1}{\rightleftharpoons}} \mathrm{C + D} \tag{S1}$$

The change in the concentration of compound **A** is described as:

$$\frac{d[\mathrm{A}]}{dt} = -k_1[\mathrm{A}][\mathrm{B}] + k_{-1}[\mathrm{C}] \quad or \quad \frac{d[\mathrm{A}]}{dt} = -k_1[\mathrm{A}][\mathrm{B}] + k_{-1}[\mathrm{C}][\mathrm{D}] \tag{S2}$$

When the initial concentrations of **A** and **B** are the same ($[A]_0 = [B]_0$), those two species keep the same ratio ([A] = [B]) throughout the reaction. Besides, in the initial stage of the reaction, the concentration of the product **C** (and **D**) is ignorable ($[C] = [D] \approx 0$). Thus, (S2) is approximated as:

$$\frac{\mathrm{d}[\mathrm{A}]}{\mathrm{dt}} = -k_1[\mathrm{A}]^2 \tag{S3}$$

$$-\frac{d[\mathrm{A}]}{[\mathrm{A}]^2} = k_1 dt \tag{S4}$$

$$\frac{1}{[\mathrm{A}]} - \frac{1}{[\mathrm{A}]_0} = k_1 t \tag{S5}$$

$$\frac{1}{[\mathrm{A}]} = k_1 t + \frac{1}{[\mathrm{A}]_0} \tag{S6}$$

The same operation can be performed for the concentration of **B**:

$$\frac{1}{[\mathrm{B}]} = k_1 t + \frac{1}{[\mathrm{B}]_0} \tag{S7}$$

The general equation of reactions of thiosulfinates (**E**) with a thiol (**B**) is shown as:

$$\mathrm{E + B} \xrightarrow{k_1} \mathrm{F} \quad \text{and} \quad \mathrm{F + B} \xrightarrow{k_2} \mathrm{G} \tag{S8}$$

The first reaction is irreversible, thus the change in the concentration of **E** is described as:

$$\frac{d[\mathrm{E}]}{dt} = -k_1[\mathrm{E}][\mathrm{B}] \tag{S9}$$

The initial product of the reaction (**F**, sulfenate) is shown to be highly reactive ($k_2 >> k_1$, see Figure S22), thus steady-state approximation can be applied to the concentration of **F**:

$$\frac{d[\mathrm{F}]}{dt} = k_1[\mathrm{E}][\mathrm{B}] - k_2[\mathrm{F}][\mathrm{B}] \approx 0 \tag{S10}$$

$$[\mathrm{F}] \approx \frac{k_1}{k_2}[\mathrm{E}] \tag{S11}$$

Hence, the change in the concentration of compound **B** is described and approximated as:

$$\frac{d[\mathrm{B}]}{dt} = -k_1[\mathrm{E}][\mathrm{B}] - k_2[\mathrm{F}][\mathrm{B}] \approx -2k_1[\mathrm{E}][\mathrm{B}] = 2\frac{d[\mathrm{E}]}{dt} \tag{S12}$$

Thus, when the initial concentrations of **E** and **B** are in 1:2 ratio ($2[\mathrm{E}]_0 = [\mathrm{B}]_0$), those two species keep the same ratio ($2[\mathrm{E}] = [\mathrm{B}]$) throughout the reaction, and (S9) and (S12) are respectively transformed as:

$$\frac{d[\mathrm{E}]}{dt} \approx -k_1[\mathrm{E}][\mathrm{B}] = -2k_1[\mathrm{E}]^2 \tag{S13}$$

$$\frac{1}{[\mathrm{E}]} = 2k_1 t + \frac{1}{[\mathrm{E}]_0} \tag{S14}$$

$$\frac{d[\mathrm{B}]}{dt} \approx -2k_1[\mathrm{E}][\mathrm{B}] = -k_1[\mathrm{B}]^2 \tag{S15}$$

$$\frac{1}{[\mathrm{B}]} = k_1 t + \frac{1}{[\mathrm{B}]_0} \tag{S16}$$

Equilibrium constants for reactions with two reactants A and B and a product C are calculated as:

$$K = \frac{[\mathrm{C}]_{\mathrm{eq}}}{[\mathrm{A}]_{\mathrm{eq}}[\mathrm{B}]_{\mathrm{eq}}} \quad \text{(S17)}$$

3.1.1. Dynamic Covalent Ring Opening with Thiols

General procedure A (at 5 mM): To a solution of deuterated sodium phosphate (200 mM in D_2O, pD 2.7 or 5.4, 180 mM final concentration), a stock solution of substrate (0.10 M in DMSO-d_6, 5.0 mM final concentration) was added and stirred. Then a stock solution of **10** (0.10 M in DMSO-d_6, 5.0 mM final concentration) was added and vigorously stirred at room temperature. The solution was transferred immediately to an NMR tube and NMR data were recorded repeatedly. The concentration of the substrate or thiol at each time plot was calculated by comparing the integrals of the peaks indicated with arrows in the figures, and plotted. The data points were fitted to obtain k_1 with the Equation (S6) or (S7) using Graphpad Prism.

General procedure B (at 2 mM): To a solution of deuterated sodium phosphate (200 mM in D_2O, pD 2.7 or 5.4, 180 mM final concentration), DMSO-d_6 (10% DMSO-d_6 in the reaction mixture) and a stock solution of substrate (0.10 M in DMSO-d_6, 2.0 mM final concentration) were added and stirred. Then a stock solution of **10** (0.10 M in DMSO-d_6, 2.0 mM final concentration) was added and vigorously stirred at room temperature. The solution was transferred immediately to an NMR tube and NMR data were recorded repeatedly. The concentration of the substrate or thiol at each time plot was calculated by comparing the integrals of the peaks indicated with arrows in the figures, and plotted. The data points were fitted to obtain k_1 with the Equation (S6) or (S7) using Graphpad Prism.

General procedure C (for thiosulfinates): To a solution of deuterated sodium phosphate (200 mM in D_2O, pD 5.4, 170 mM final concentration), a stock solution of substrate (0.10 M in DMSO-d_6, 5.0 mM final concentration) was added and stirred. Then a stock solution of **10** (0.10 M in DMSO-d_6, 10 mM final concentration) was added and vigorously stirred at room temperature. The solution was transferred immediately to an NMR tube and NMR data were recorded repeatedly. The concentration of the substrate or thiol at each time plot was calculated by comparing the integrals of the peaks indicated with arrows in the figures, and plotted. The data points were fitted to obtain k_1 with the Equation (S14) or (S16) using Graphpad Prism.

Compound 2 was synthesized and purified according to procedures described in ref. S33.

Compound 10 was synthesized and purified according to procedures described in ref. S34.

Exchange of 3 with thiol 10 at pD 5.4 (Table 1, entry 1). Following the procedure A, 1.0 mL of reaction mixture was prepared. The reaction was monitored by 300 MHz NMR over 1 h. The products **9** (1:1 mixture of diastereomers) were identified by ^{1}H NMR (300 MHz): 4.45/4.41 (s, 1H), 4.02 – 3.91 (s, 1H), 3.88 – 3.78 (m, 1H), 3.08 – 2.94 (m, 1H), 2.83 – 2.72 (m, 1H), 2.67 – 2.56 (m, 1H), 2.46 – 2.36 (m, 1H), 2.06 (s, 3H), 1.47/1.45 (s, 3H), 1.40/1.37 (s, 3H).

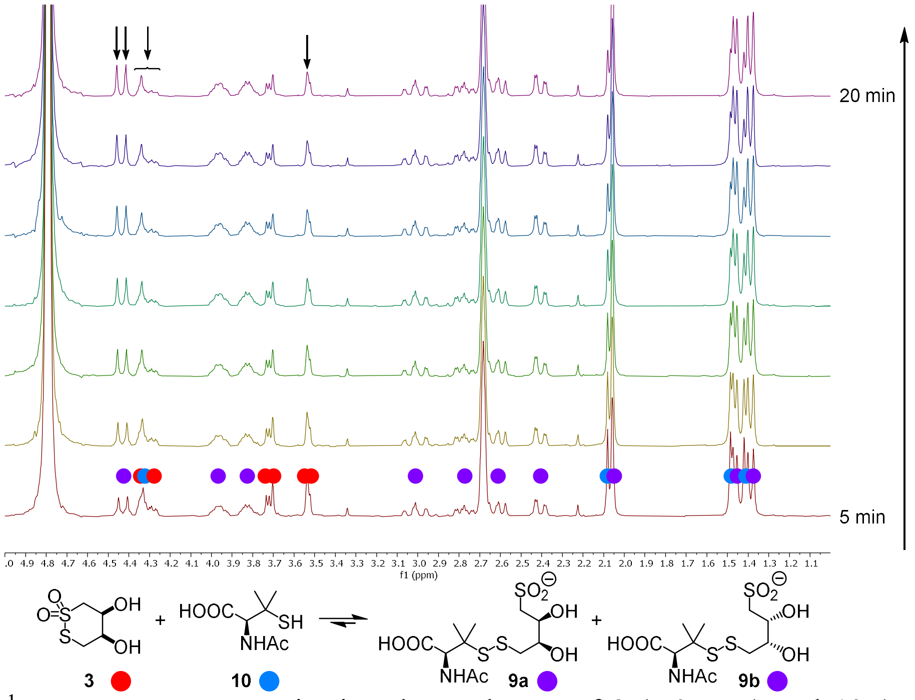

Figure S4. ^{1}H NMR spectra monitoring the exchange of **3** (5.0 mM) and **10** (5.0 mM) in a deuterated sodium phosphate solution (180 mM, pD 5.4) in D_2O/DMSO-d_6 9:1.

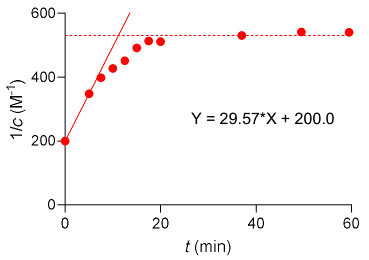

Figure S5. Inverse of concentration c of **3** as a function of exchange time t with **10** with a dotted line indicating the equilibrium. The first two points (t = 0, 5 min) were used for the estimation of the rate constant with Equation (S6), $k_1 > 0.49$ M^{-1} s^{-1}. Equilibrium constant K was calculated based on the data at t = 37.5 min following Equation (S17), $K = 6.0 \times 10^3$ M^{-1} (Table 1, entry 1).

Exchange of 3 with thiol 10 at pD 2.7 (Table 1, entry 2). Following the procedure A, 1.0 mL of reaction mixture was prepared. The reaction was monitored by 300 MHz NMR over 7 h. The products **9** (1:1 mixture of diastereomers) were identified by ^{1}H NMR (300 MHz): 4.56/4.52 (s, 1H), 4.01 – 3.91 (m, 1H), 3.88 – 3.78 (m, 1H), 3.09 – 2.95 (m, 1H), 2.86 – 2.61 (m, 2H), 2.57 – 2.48 (m, 1H), 2.05 (s, 3H), 1.47/1.45 (s, 3H), 1.41/1.39 (s, 3H).

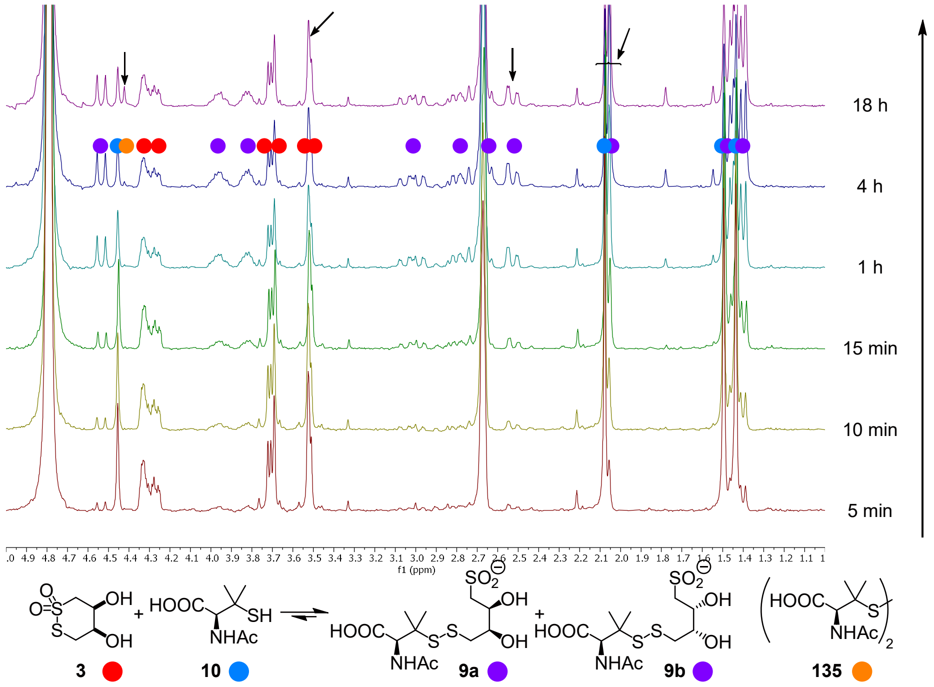

Figure S6. ^{1}H NMR spectra monitoring the exchange of **3** (5.0 mM) and **10** (5.0 mM) in a deuterated sodium phosphate solution (180 mM, pD 2.7) in D_2O/DMSO-d_6 9:1.

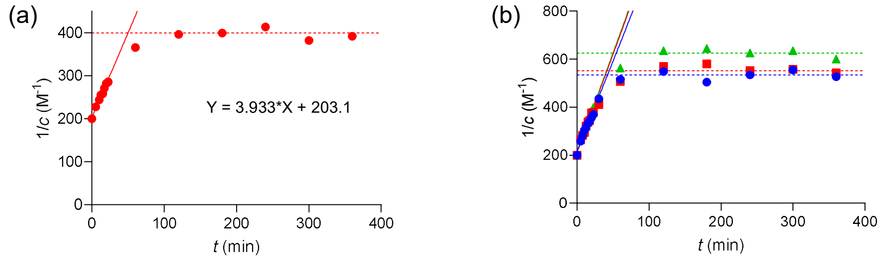

Figure S7. (a) Inverse of concentration c of **3** as a function of exchange time t with **10** with a dotted line indicating the equilibrium. The first seven points (t = 0 – 20 min) before the onset of equilibration (Figure 3C) were used for the linear fitting to Equation (S6), $k_1 = 0.066 \pm 0.003$ M^{-1} s^{-1} (best fit ± SE). Equilibrium constant K was calculated based on the data at t = 360 min following Equation (S17), $K = 450$ M^{-1}; (b) Results of technical triplicates, $k_1 = 0.133 \pm 0.004$ M^{-1} s^{-1}, $K = 547 \pm 2$ M^{-1} (mean ± SEM) at t = 240 min (Table 1, entry 2).

Exchange of 15 with thiol 10 at pD 2.7 (Table 1, entry 11). Following the procedure A, 1.0 mL of reaction mixture was prepared. The reaction was monitored by 300 MHz NMR over 12 h. The products **136** (1:1 mixture of diastereomers) were identified by ^{1}H NMR (300 MHz): 4.53/4.50 (s, 1H), 4.04 – 3.94 (m, 1H), 3.85 – 3.77 (br m, 1H), 3.04 – 2.71 (m, 3H), 2.43 (dd, $^{2}J_{HH}$ = 13.0, $^{3}J_{HH}$ = 3.5 Hz, 1H), 2.06 (s, 3H), 1.46 (s, 3H), 1.41/1.39 (s, 3H).

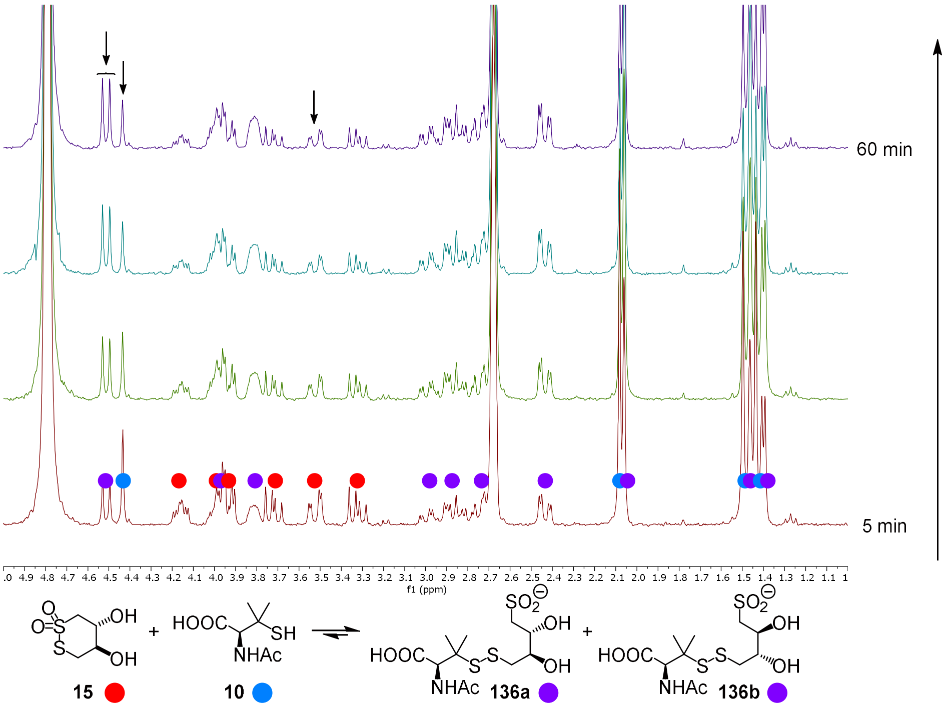

Figure S8. ^{1}H NMR spectra monitoring the exchange of **15** (5.0 mM) and **10** (5.0 mM) in a deuterated sodium phosphate solution (180 mM, pD 2.7) in D_2O/DMSO-d_6 9:1.

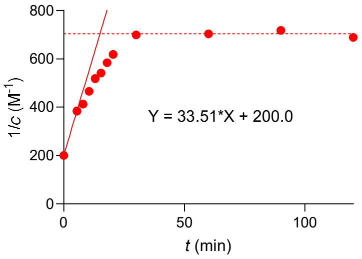

Figure S9. Inverse of concentration c of **15** as a function of exchange time t with **10** with a dotted line indicating the equilibrium. The first two points (t = 0, 5 min) were used for the estimation of the rate constant with Equation (S6), $k_1 > 0.56$ M^{-1} s^{-1}. Equilibrium constant K was calculated based on the data at t = 60 min following Equation (S17), $K = 2.0 \times 10^{3}$ M^{-1} (Table 1, entry 11).

Exchange of 11 with thiol 10 at pD 2.7 (Table 1, entry 12). Following the procedure B, 2.0 mL of reaction mixture was prepared. The reaction was monitored by 400 MHz NMR over 1 h. The products **19** (1:1 mixture of diastereomers) were identified by ^{1}H NMR (300 MHz): 5.28 – 5.15 (m, 2H), 4.54 (s, 1H), 3.27 – 3.06 (m, 2H), 3.02 – 2.89 (m, 1H), 2.57 – 2.48 (m, 1H), 2.06 (s, 3H), 1.47/1.45 (s, 3H), 1.42/1.40 (s, 3H).

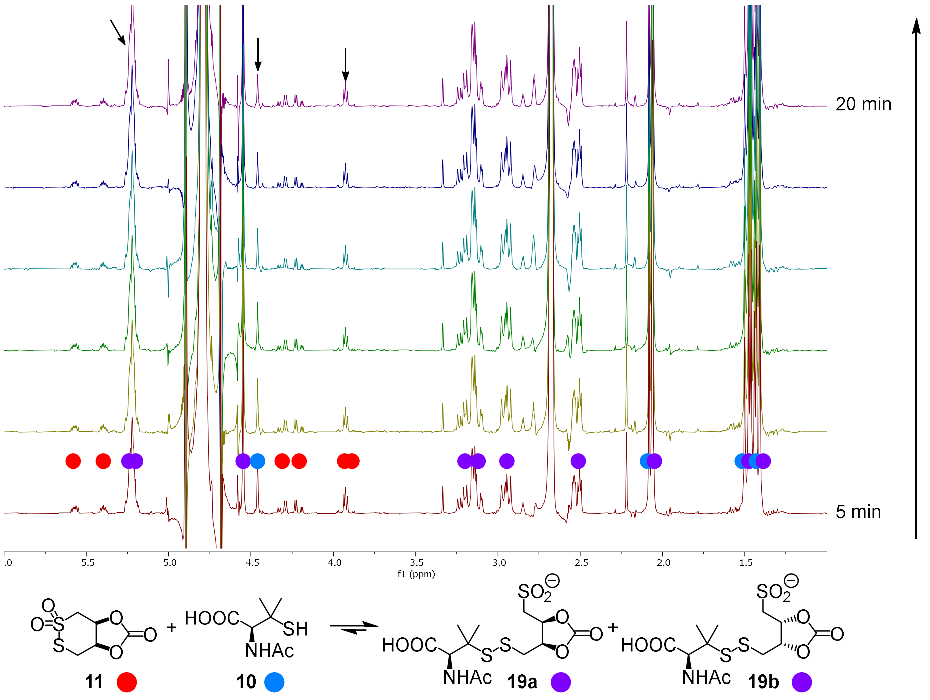

Figure S10. ^{1}H NMR spectra monitoring the exchange of **11** (2.0 mM) and **10** (2.0 mM) in a deuterated sodium phosphate solution (180 mM, pD 2.7) in D_2O/DMSO-d_6 9:1.

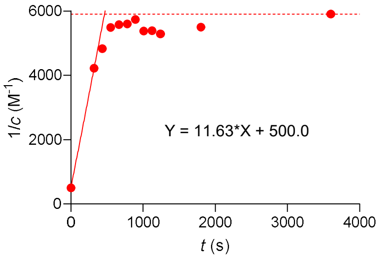

Figure S11. Inverse of concentration c of **11** as a function of exchange time t with **10** with a dotted line indicating the equilibrium. The first two points (t = 0, 320 s) were used for the estimation of the rate constant with Equation (S6), $k_1 > 12$ M^{-1} s^{-1}. Equilibrium constant K was calculated based on the data at t = 3600 s following Equation (S17), $K > 1.7 \times 10^5$ M^{-1} (Table 1, entry 12).

Exchange of 12 with thiol 10 at pD 2.7 (Table 1, entry 13). Following the procedure B, 2.0 mL of reaction mixture was prepared. The reaction was monitored by 400 MHz NMR over 30 min. The products **137** (1:1 mixture of diastereomers) were identified by ^{1}H NMR (300 MHz): 6.07 (d, $^2J_{HH}$ = 10.3 Hz, 1H), 5.92 – 5.83 (m, 1H), 5.14 – 5.04 (m, 1H), 4.54 (s, 1H), 2.98 – 2.80 (m, 2H), 2.06/2.05 (s, 3H), 1.47/1.46 (s, 3H), 1.42/1.41 (s, 3H).

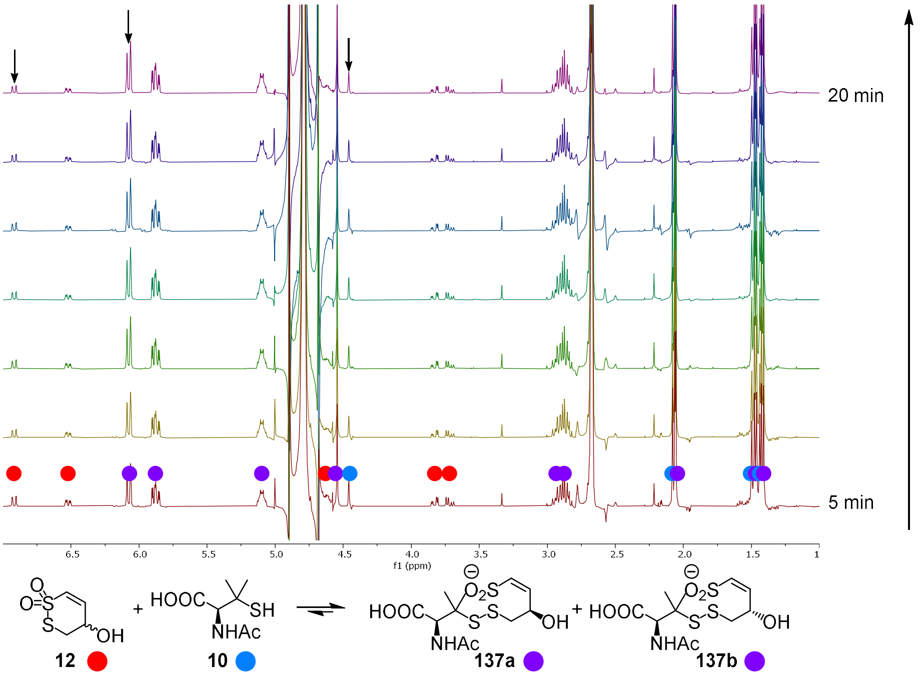

Figure S12. ^{1}H NMR spectra monitoring the exchange of **12** (2.0 mM) and **10** (2.0 mM) in a deuterated sodium phosphate solution (180 mM, pD 2.7) in D_2O/DMSO-d_6 9:1.

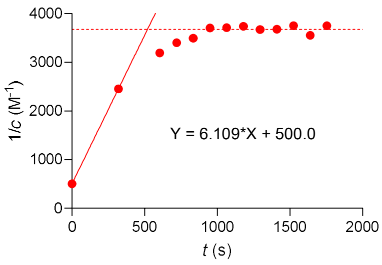

Figure S13. Inverse of concentration c of **12** as a function of exchange time t with **10** with a dotted line indicating the equilibrium. The first two points (t = 0, 300 s) were used for the estimation of the rate constant with Equation (S6), $k_1 > 6.1$ M^{-1} s^{-1}. Equilibrium constant K was calculated based on the data at t = 1300 s following Equation (S17), $K = 2.1 \times 10^4$ M^{-1} (Table 1, entry 13).

Exchange of 13 with thiol 10 at pD 2.7 (Table 1, entry 14). Following the procedure B, 1.0 mL of reaction mixture was prepared. The reaction was monitored by 400 MHz NMR over 3.5 h. The products **138** (1:1 mixture of diastereomers) were identified by ^{1}H NMR (400 MHz): 4.57 – 4.48 (m, 3H), 3.10 – 2.79 (m, 2H), 2.76 – 2.69 (m, 1H), 2.38 – 2.29 (m, 1H), 2.07/2.06 (s, 3H), 1.51 (s, 3H), 1.47/1.46 (s, 3H), 1.44/1.42 (s, 3H), 1.40 (s, 3H).

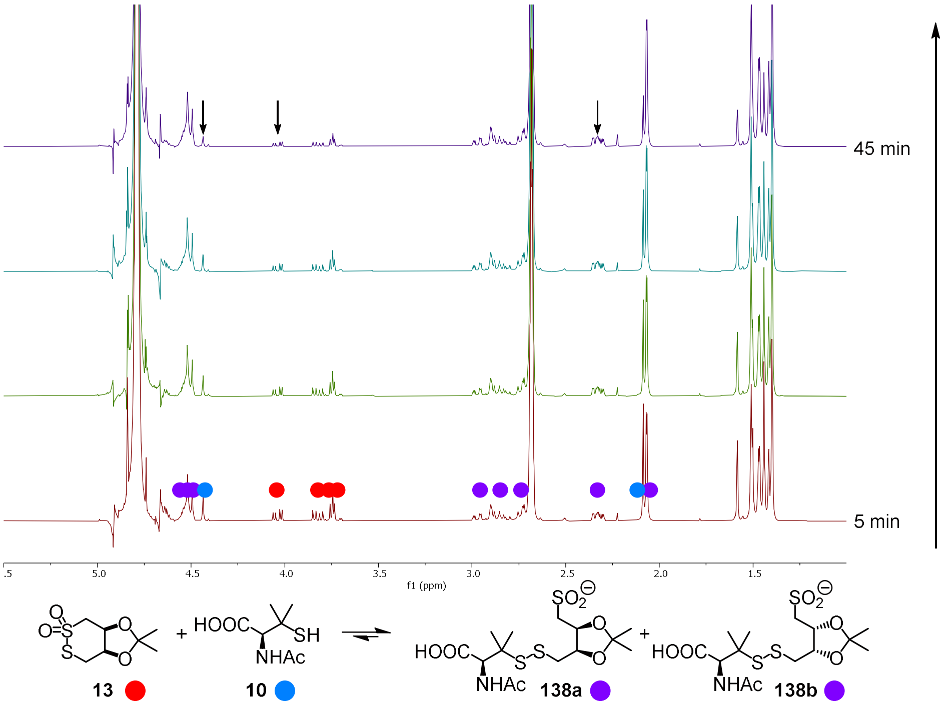

Figure S14. ^{1}H NMR spectra monitoring the exchange of **13** (2.0 mM) and **10** (2.0 mM) in a deuterated sodium phosphate solution (180 mM, pD 2.7) in D_2O/DMSO-d_6 9:1.

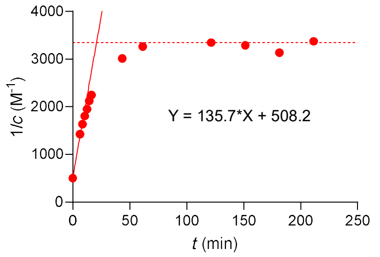

Figure S15. Inverse of concentration c of **13** as a function of exchange time t with **10** with a dotted line indicating the equilibrium. The first three points (t = 0, 6.5, 8.5 min) before the onset of equilibration (Figure 3C) were used for the linear fitting to Equation (S6), k_1 = 2.3 ± 0.1 M^{-1} s^{-1} (best fit ± SE). Equilibrium constant K was calculated based on the data at t = 120 min following Equation (S17), $K = 1.7 \times 10^4$ M^{-1} (Table 1, entry 14).

Exchange of 14 with thiol 10 at pD 2.7 (Table 1, entry 15). Following the procedure B, 1.0 mL of reaction mixture was prepared. The reaction was monitored by 400 MHz NMR over 75 min. The product **139** was identified by ^{1}H NMR (400 MHz): 4.51 (s, 1H), 2.43 (s, 3H), 2.06 (s, 3H), 1.47 (s, 3H), 1.40 (s, 3H). The peak at 2.36 ppm corresponds to $MeSO_2^-$.

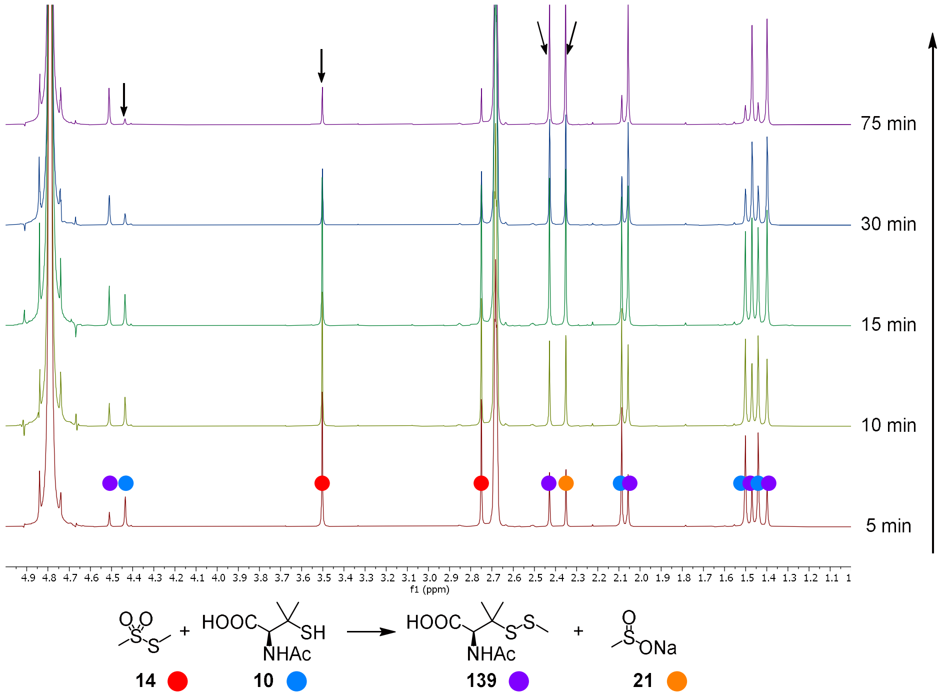

Figure S16. ^{1}H NMR spectra monitoring the exchange of **14** (2.0 mM) and **10** (2.0 mM) in a deuterated sodium phosphate solution (180 mM, pD 2.7) in D_2O/DMSO-d_6 9:1.

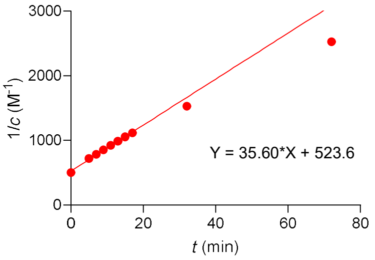

Figure S17. Inverse of concentration c of **10** as a function of exchange time t with **14**. The first eight points (t = 0 – 17 min) were used for the linear fitting to Equation (S7), k_1 = 0.52 ± 0.02 M^{-1} s^{-1} (best fit ± SE, Table 1, entry 15).

Exchange of 16 with thiol 10 at pD 5.4 (Table 1, entry 16). Following the procedure A, 0.50 mL of reaction mixture was prepared. The reaction was monitored by 300 MHz NMR over 9 h. The product **24** was identified by ^{1}H NMR (300 MHz): 8.10 – 8.01 (m, 1H), 7.80 – 7.68 (m, 2H), 4.40 (s, 1H), 1.97 (s, 3H), 1.44 (s, 3H), 1.38 (s, 3H).

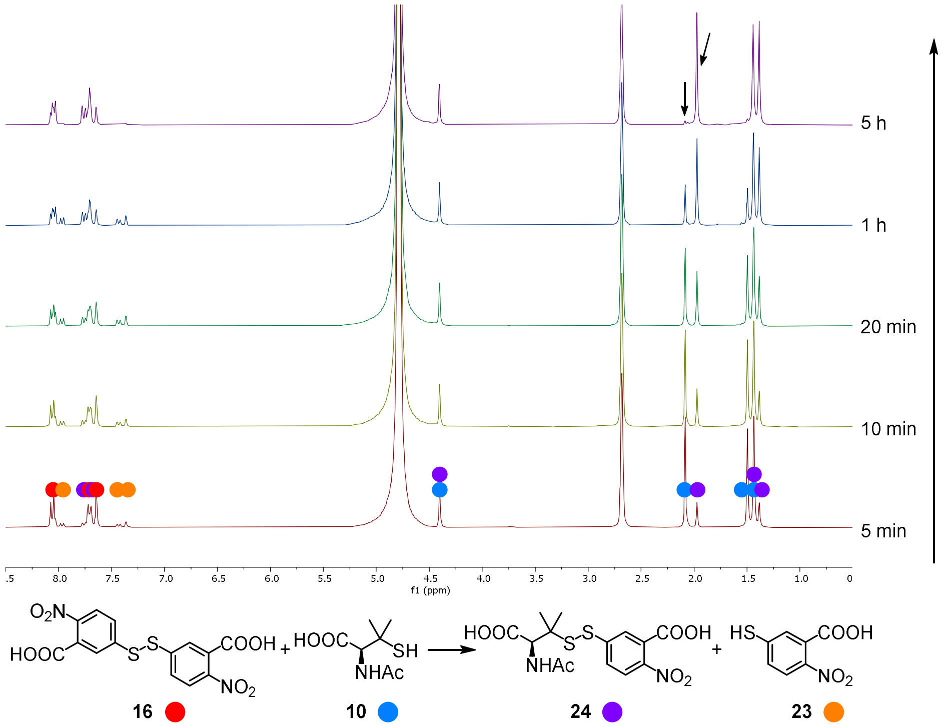

Figure S18. ^{1}H NMR spectra monitoring the exchange of **16** (5.0 mM) and **10** (5.0 mM) in a deuterated sodium phosphate solution (180 mM, pD 5.4) in D_2O/DMSO-d_6 9:1.

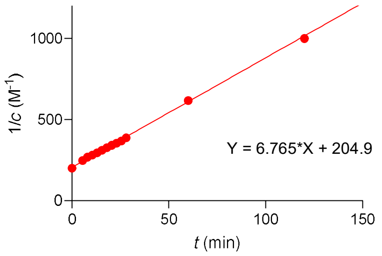

Figure S19. Inverse of concentration c of **10** as a function of exchange time t with **16**. The first twelve points (t = 0 – 60 min) were used for the linear fitting to Equation (S7), k_1 = 0.113 ± 0.002 M^{-1} s^{-1} (best fit ± SE, Table 1, entry 16).

Exchange of 17 with thiol 10 at pD 5.4 (Table 1, entry 17). Following the procedure C, 0.60 mL of reaction mixture was prepared. The reaction was monitored by 300 MHz NMR over 60 h. The product **139** was identified by ^{1}H NMR (400 MHz): 4.44 (s, 1H), 2.43 (s, 3H), 2.06 (s, 3H), 1.48 (s, 3H), 1.39 (s, 3H). No peak from MeSOH was observed.

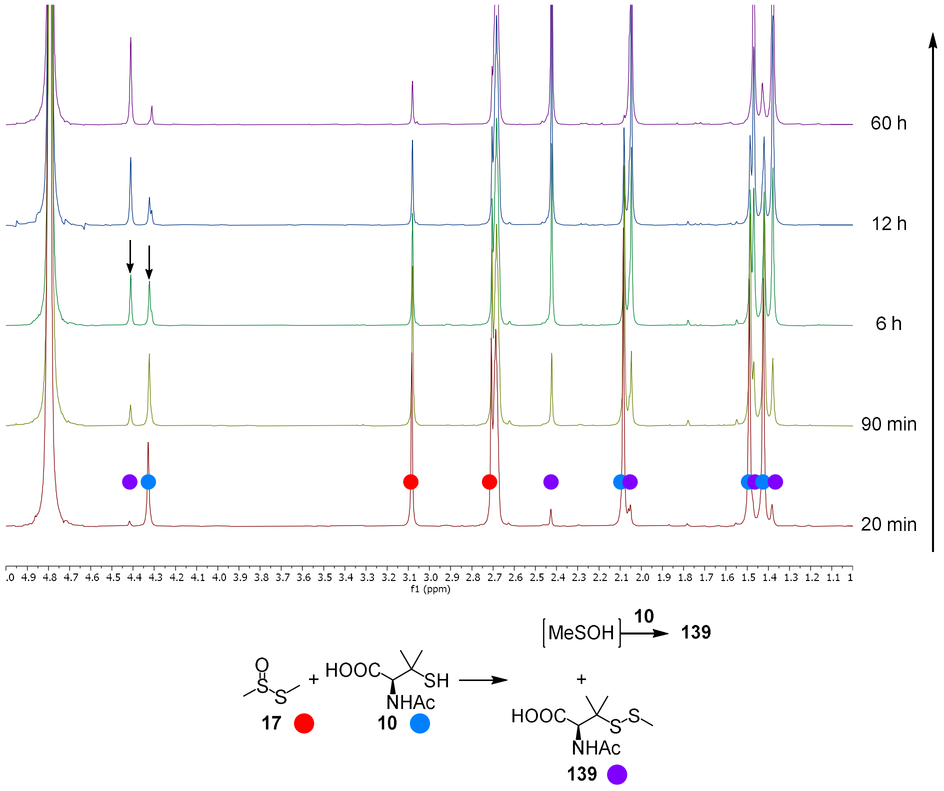

Figure S20. ^{1}H NMR spectra monitoring the exchange of **17** (5.0 mM) and **10** (10 mM) in a deuterated sodium phosphate solution (170 mM, pD 5.4) in D_2O/DMSO-d_6 85:15.

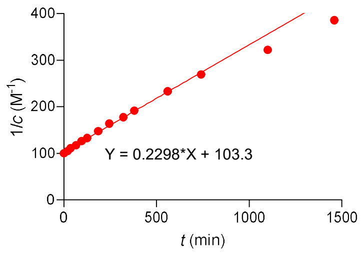

Figure S21. Inverse of concentration c of **10** as a function of exchange time t with **17**. The first twelve points (t = 0 – 740 min) were used for the linear fitting to Equation (S16), k_1 = (4.15 ± 0.05) × 10^{-3} M^{-1} s^{-1} (best fit ± SE, Table 1, entry 17).

Exchange of 2 with thiol 10 at pD 6.7. Following the procedure C, 1.0 mL of reaction mixture was prepared with pD 6.7 buffer instead of pD 5.4 buffer. The reaction was monitored by 300 MHz NMR over 7 h. The product **141** was identified by ^{1}H NMR (400 MHz): 4.37 (s, 1H), 4.32 (s, 1H), 3.90 – 3.81 (m, 2H), 3.08 – 2.94 (m, 2H), 2.88 – 2.74 (m, 2H), 2.05 (s, 3H), 2.05 (s, 3H), 1.47 (s, 3H), 1.45 (s, 3H), 1.38 (s, 3H), 1.35 (s, 3H). Use of 1 equiv. of thiol **10** led to a ~1:1 mixture of **2** and **141** after 36 h under otherwise same conditions, which indicates the fast capture of intermediate **140** by thiol **10**.

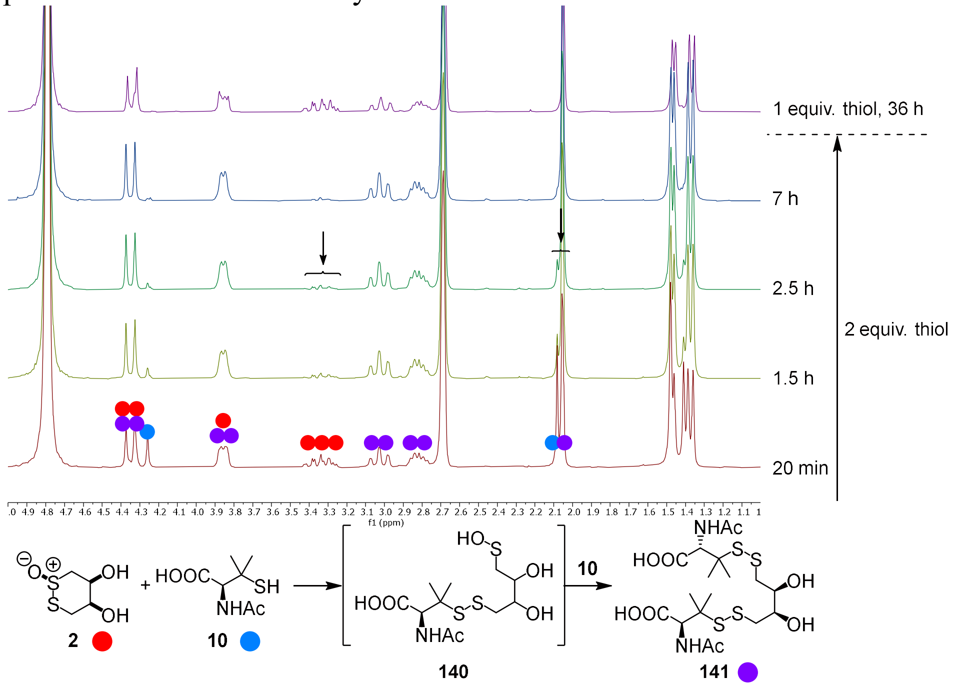

Figure S22. ^{1}H NMR spectra monitoring the exchange of **2** (5.0 mM) and **10** (5.0 mM or 10 mM) in a deuterated sodium phosphate solution (170 mM, pD 6.7) in D_2O/DMSO-d_6 85:15.

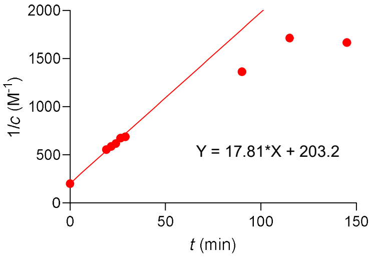

Figure S23. Inverse of concentration c of **2** as a function of exchange time t with **10** (10 mM). The first five points (t = 0 – 26.5 min) were used for the linear fitting to Equation (S14), k_1 = 0.148 ± 0.004 M^{-1} s^{-1} (best fit ± SE).

Exchange of 2 with thiol 10 at pD 5.4 (Table 1, entry 18). Following the procedure C, 1.0 mL of reaction mixture was prepared. The reaction was monitored by 300 MHz NMR over 60 h. Gradual formation of **1** was also observed and a proposed mechanism is also shown below. The products **141** and **1** were identified by ^{1}H NMR (400 MHz); **141**: 4.44 (s, 1H), 4.40 (s, 1H), 3.88 – 3.83 (m, 2H), 3.09 – 2.97 (m, 2H), 2.88 – 2.78 (m, 2H), 2.06 (s, 6H), 1.48 (s, 3H), 1.46 (s, 3H), 1.40 (s, 3H), 1.38 (s, 3H); **1**: 4.06 – 3.91 (br s, 2H), 3.16 – 3.05 (m, 4H).

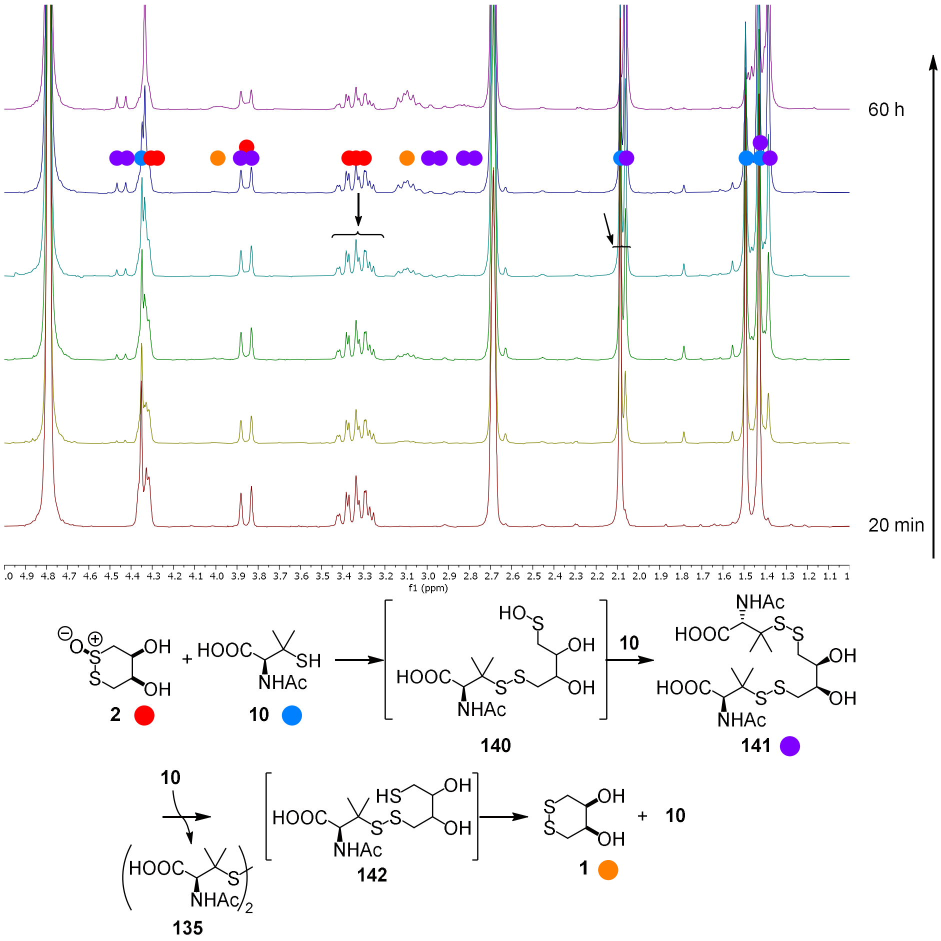

Figure S24. ^{1}H NMR spectra monitoring the exchange of **2** (5.0 mM) and **10** (10 mM) in a deuterated sodium phosphate solution (170 mM, pD 5.4) in D_2O/DMSO-d_6 85:15 and a proposed mechanism for the formation of **1**.

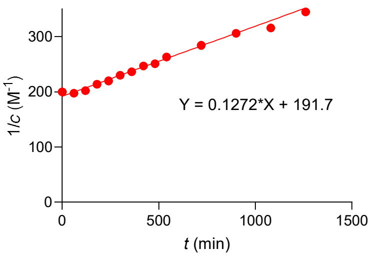

Figure S25. Inverse of concentration c of **2** as a function of exchange time t with **10**. The first twelve points (t = 0 – 900 min) were used for the linear fitting to Equation (S14), $k_1 = (1.06 \pm 0.03) \times 10^{-3}$ M^{-1} s^{-1} (best fit ± SE, Table 1, entry 18).

Exchange of 18 with thiol 10 at pD 5.4 (Table 1, entry 19). Following the procedure A, 0.60 mL of reaction mixture was prepared. The reaction was monitored by 300 MHz NMR over 60 h. The expected product **139** was not observed. Only slow oxidation of **10** to homo-disulfide **135** was observed; **135**: ^{1}H NMR (300 MHz): 4.28 (s, 2H), 2.05 (s, 6H), 1.42 (s, 6H), 1.37 (s, 6H).

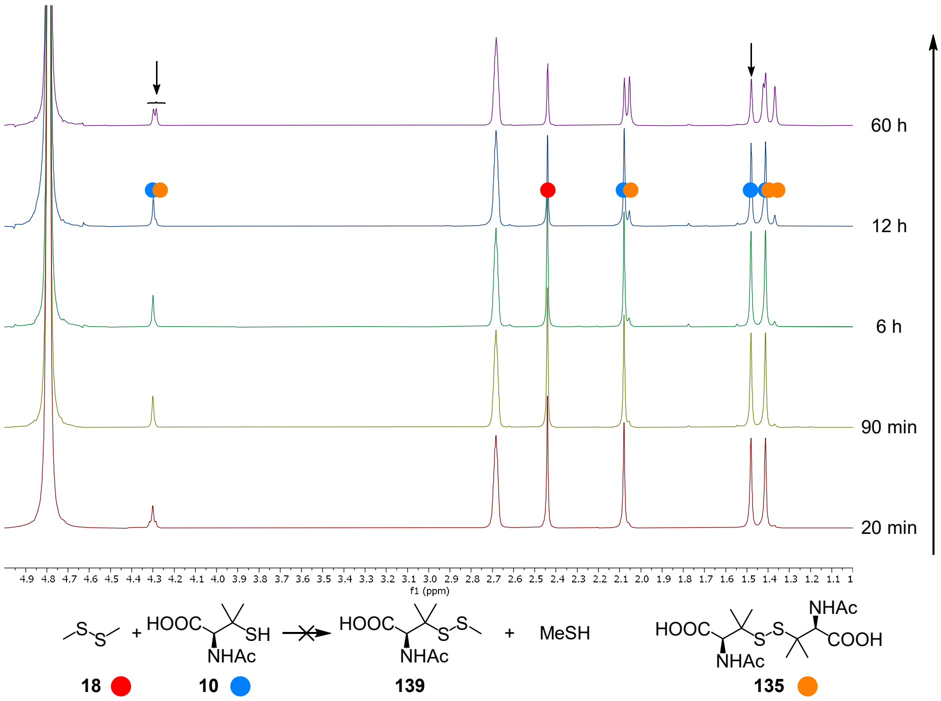

Figure S26. ^{1}H NMR spectra monitoring the exchange of **18** (5 mM) and **10** (5 mM) in a deuterated sodium phosphate solution (180 mM, pD 5.4) in D_2O/DMSO-d_6 9:1.

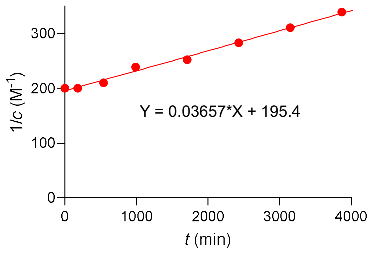

Figure S27. Inverse of concentration c of **10** as a function of time t. The first eight points (t = 0 – 3870 min) were used for the linear fitting to Equation (S7) leading to the rate constant of oxidation of **10** into **135**, $k_1 = (6.1 \pm 0.2) \times 10^{-4}\ M^{-1}\ s^{-1}$ (best fit ± SE).

3.1.2. Dependence of Ring Opening on Proticity

A mixture of deuterated phosphoric acid and NaOD (30% in D_2O), which gave pD 2.7 in D_2O, was used as buffer in all solvent mixtures. To a solution of deuterated sodium phosphate (20 mM final concentration) in a mixture of D_2O and DMSO-d_6 (540 µL, final D_2O content: 90, 70, 50, 30, 10, 8, 5 or 2%), a stock solution of **3** (0.10 M in DMSO-d_6, 5.0 mM final concentration) was added and stirred. Then a stock solution of **10** (0.10 M in DMSO-d_6, 5.0 mM final concentration) was added and vigorously stirred at room temperature. The solution was transferred immediately to an NMR tube and ^1H NMR data were recorded repeatedly.

Separatedly, solutions of **10** (5.0 mM) in a solution of deuterated sodium phosphate (20 mM final concentration) in a mixture of D_2O and DMSO-d_6 (540 µL, final D_2O content: 90, 70, 50, 30, 10, 8, 5 or 2%) were prepared as a control and oxidation of **10** into disulfide **135** was traced on ^1H NMR.

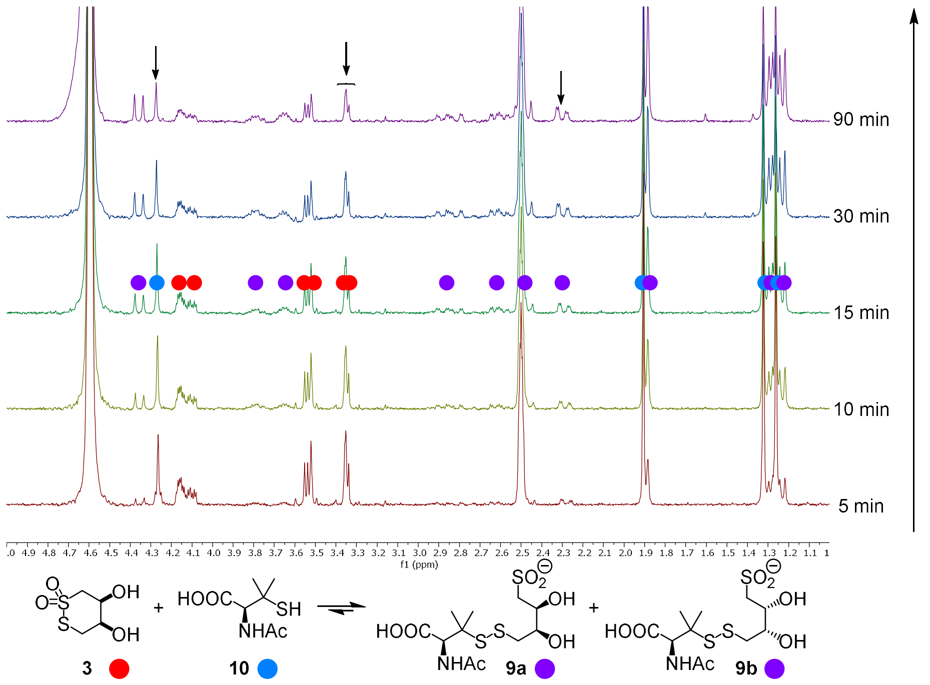

Figure S28. ^{1}H NMR spectra monitoring the exchange of **3** (5.0 mM) and **10** (5.0 mM) in a deuterated sodium phosphate solution (20 mM) in D_2O/DMSO-d_6 9:1 (Table 1, entry 3).

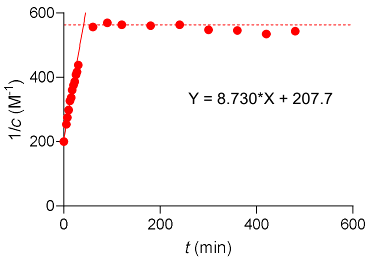

Figure S29. Inverse of concentration c of **3** as a function of exchange time t with **10** with a dotted line indicating the equilibrium. The first eight points (t = 0 – 20 min) before the onset of equilibration (Figure 3C) were used for the linear fitting to Equation (S6), k_1 = 0.15 ± 0.01 M^{-1} s^{-1} (best fit ± SE). Equilibrium constant K was calculated based on the data at t = 120 min following Equation (S17), $K = 7.1 \times 10^2$ M^{-1}. Up to 10% of **10** was converted to **135** within 120 min in the control experiment (Table 1, entry 3).

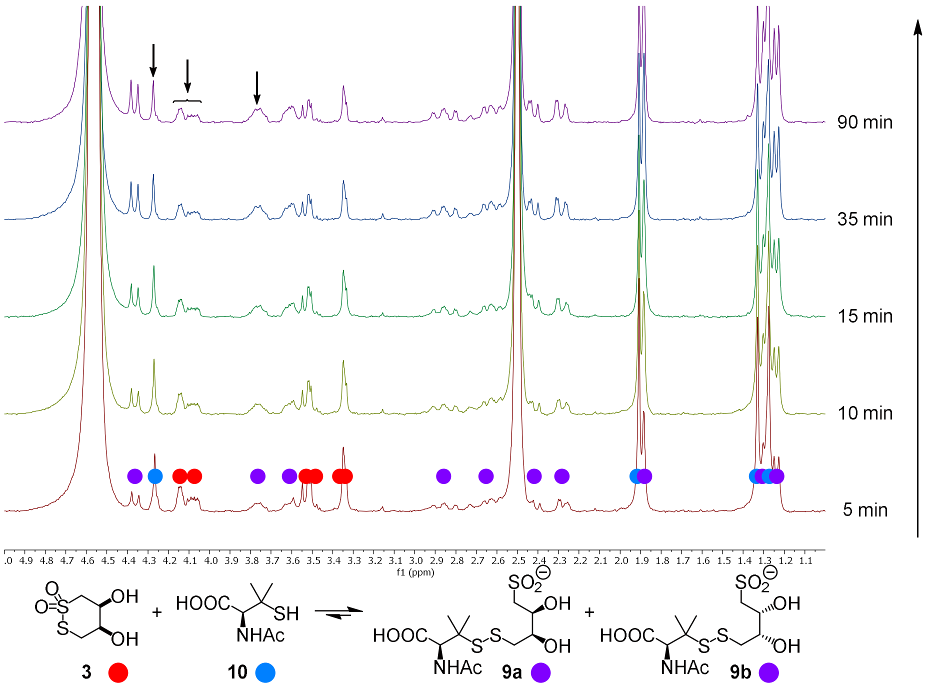

Figure S30. ^{1}H NMR spectra monitoring the exchange of **3** (5.0 mM) and **10** (5.0 mM) in a deuterated sodium phosphate solution (20 mM) in D_2O/DMSO-d_6 7:3 (Table 1, entry 4).

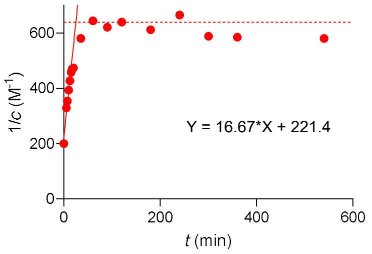

Figure S31. Inverse of concentration *c* of **3** as a function of exchange time *t* with **10** with a dotted line indicating the equilibrium. The first six points (t = 0 – 15 min) before the onset of equilibration (Figure 3C) were used for the linear fitting to Equation (S6), k_1 = 0.28 ± 0.03 M^{-1} s^{-1} (best fit ± SE). Equilibrium constant K was calculated based on the data at t = 60 min following Equation (S17), $K = 1.0 \times 10^3$ M^{-1}. Up to 10% of **10** was converted to **135** within 120 min in the control experiment (Table 1, entry 4).

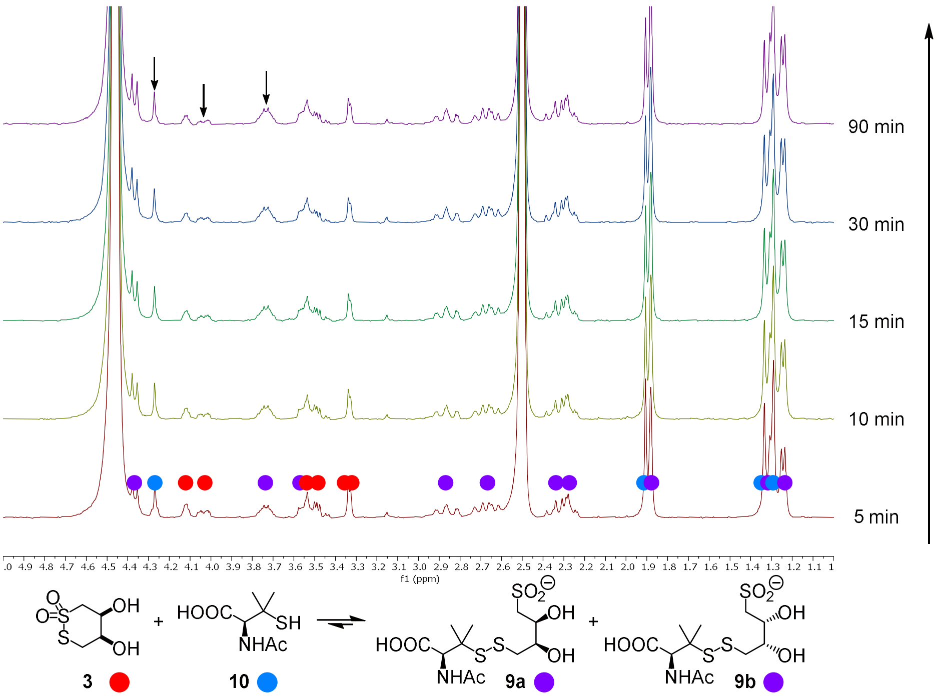

Figure S32. ^{1}H NMR spectra monitoring the exchange of **3** (5.0 mM) and **10** (5.0 mM) in a deuterated sodium phosphate solution (20 mM) in D_2O/DMSO-d_6 1:1 (Table 1, entry 5).

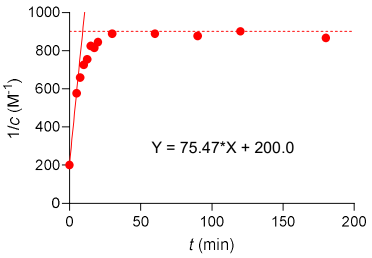

Figure S33. Inverse of concentration *c* of **3** as a function of exchange time *t* with **10** with a dotted line indicating the equilibrium. The first two points (t = 0, 5 min) were used for the estimation of the rate constant with Equation (S6), $k_1 > 1.3$ M^{-1} s^{-1}. Equilibrium constant K was calculated based on the data at t = 120 min following Equation (S17), $K = 1.8 \times 10^3$ M^{-1}. Up to 8% of **10** was converted to **135** within 120 min in the control experiment (Table 1, entry 5).

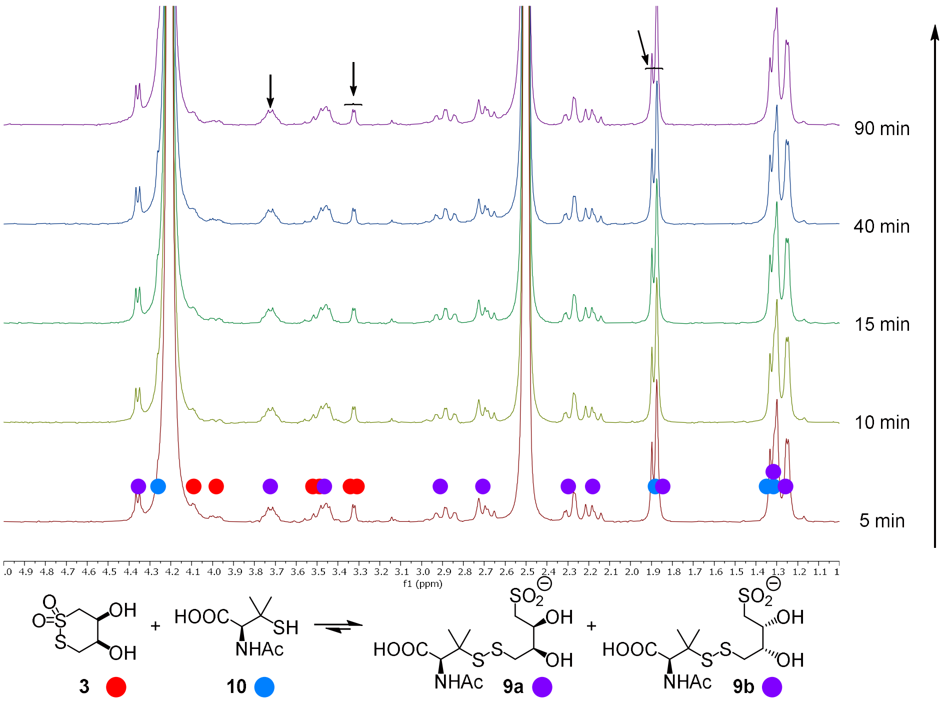

Figure S34. ^{1}H NMR spectra monitoring the exchange of **3** (5.0 mM) and **10** (5.0 mM) in a deuterated sodium phosphate solution (20 mM) in D_2O/DMSO-d_6 3:7 (Table 1, entry 6).

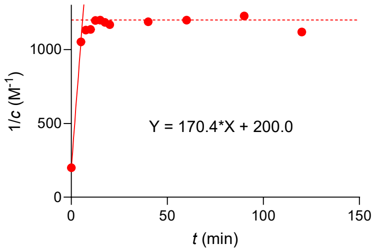

Figure S35. Inverse of concentration c of **3** as a function of exchange time t with **10** with a dotted line indicating the equilibrium. The first two points (t = 0, 5 min) were used for the estimation of the rate constant with Equation (S6), $k_1 > 2.8$ M^{-1} s^{-1}. Equilibrium constant K was calculated based on the data at t = 60 min following Equation (S17), $K = 2.3 \times 10^{3}$ M^{-1}. Up to 6% of **10** was converted to **135** within 120 min in the control experiment (Table 1, entry 6).

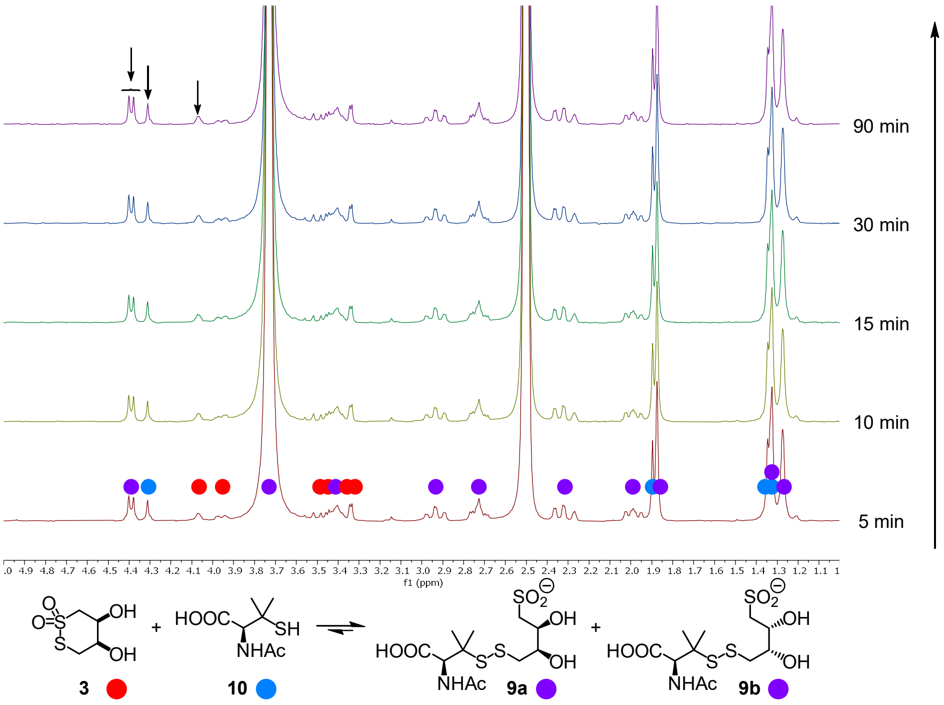

Figure S36. ^{1}H NMR spectra monitoring the exchange of **3** (5.0 mM) and **10** (5.0 mM) in a deuterated sodium phosphate solution (20 mM) in D_2O/DMSO-d_6 1:9 (Table 1, entry 7).

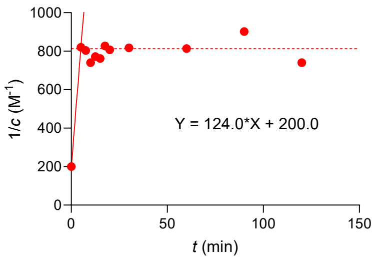

Figure S37. Inverse of concentration *c* of **3** as a function of exchange time *t* with **10** with a dotted line indicating the equilibrium. The first two points (t = 0, 5 min) were used for the estimation of the rate constant with Equation (S6), $k_1 > 2.1$ M^{-1} s^{-1}. Equilibrium constant K was calculated based on the data at t = 60 min following Equation (S17), $K = 2.1 \times 10^3$ M^{-1}. Up to 7% of **10** was converted to **135** within 120 min in the control experiment (Table 1, entry 7).

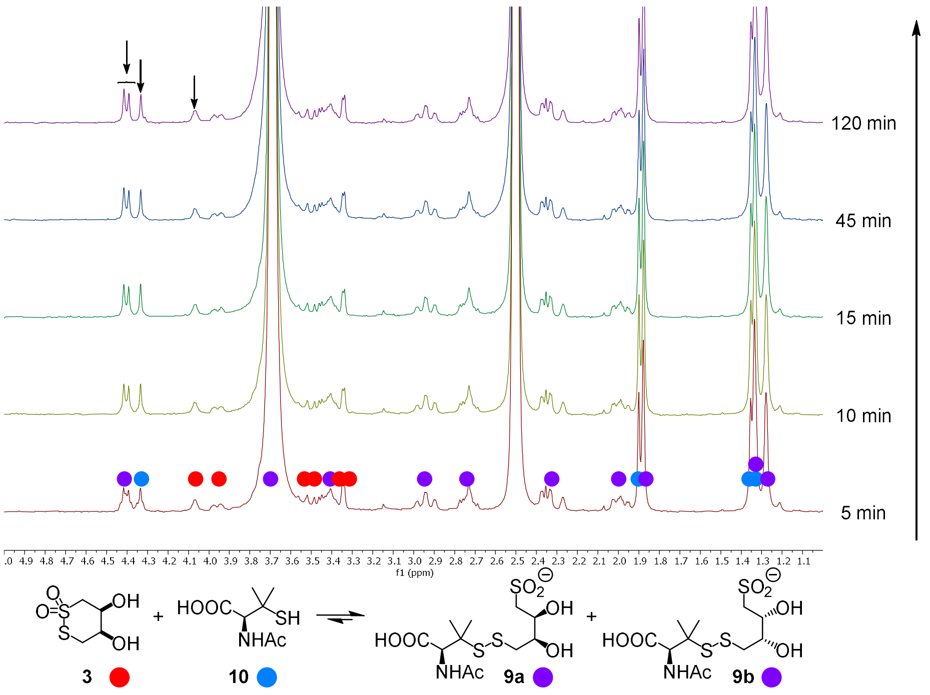

Figure S38. ^{1}H NMR spectra monitoring the exchange of **3** (5 mM) and **10** (5 mM) in a deuterated sodium phosphate solution (20 mM) in D_2O/DMSO-d_6 8:92 (Table 1, entry 8).

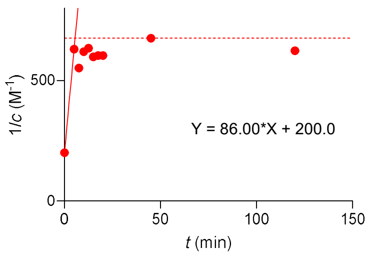

Figure S39. Inverse of concentration c of **3** as a function of exchange time t with **10** with a dotted line indicating the equilibrium. The first two points (t = 0, 5 min) were used for the estimation of the rate constant with Equation (S6), $k_1 > 1.4$ M^{-1} s^{-1}. Equilibrium constant K was calculated based on the data at t = 45 min following Equation (S17), $K = 1.3 \times 10^3$ M^{-1}. No oxidation of **10** was observed within 360 min in the control experiment (Table 1, entry 8).

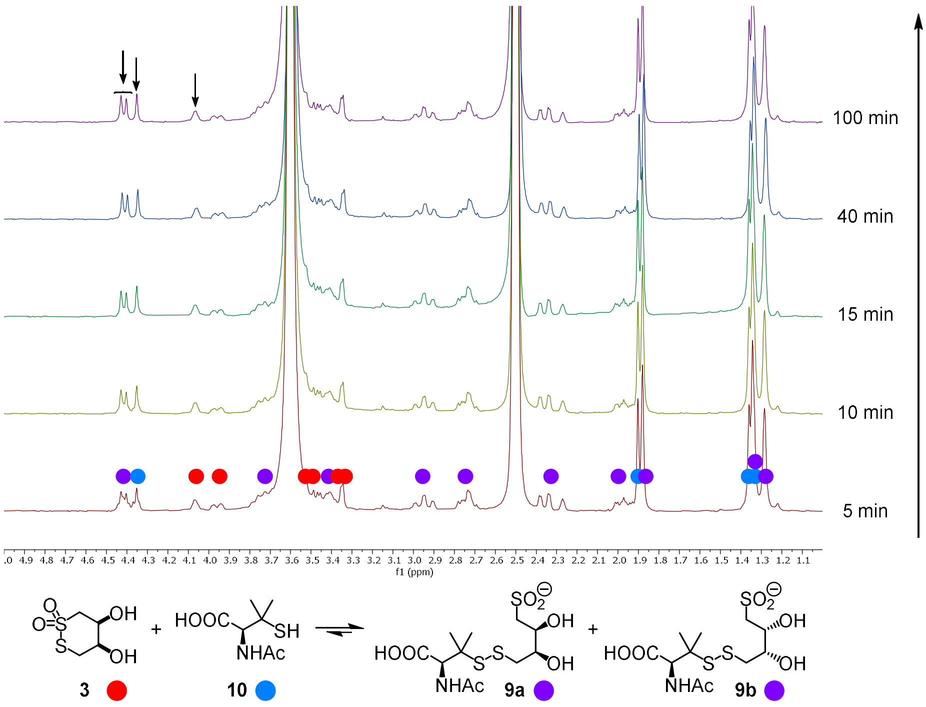

Figure S40. ^{1}H NMR spectra monitoring the exchange of **3** (5.0 mM) and **10** (5.0 mM) in a deuterated sodium phosphate solution (20 mM) in D_2O/DMSO-d_6 5:95 (Table 1, entry 9).

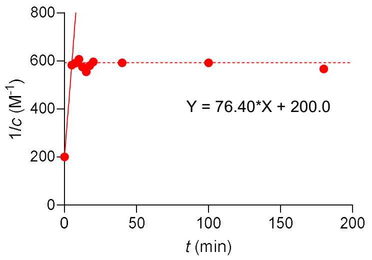

Figure S41. Inverse of concentration c of **3** as a function of exchange time t with **10** with a dotted line indicating the equilibrium. The first two points (t = 0, 5 min) were used for the estimation of the rate constant with Equation (S6), $k_1 > 1.3$ M^{-1} s^{-1}. Equilibrium constant K was calculated based on the data at t = 40 min following Equation (S17), $K = 9.4 \times 10^2$ M^{-1}. No oxidation of **10** was observed within 360 min in the control experiment (Table 1, entry 9).

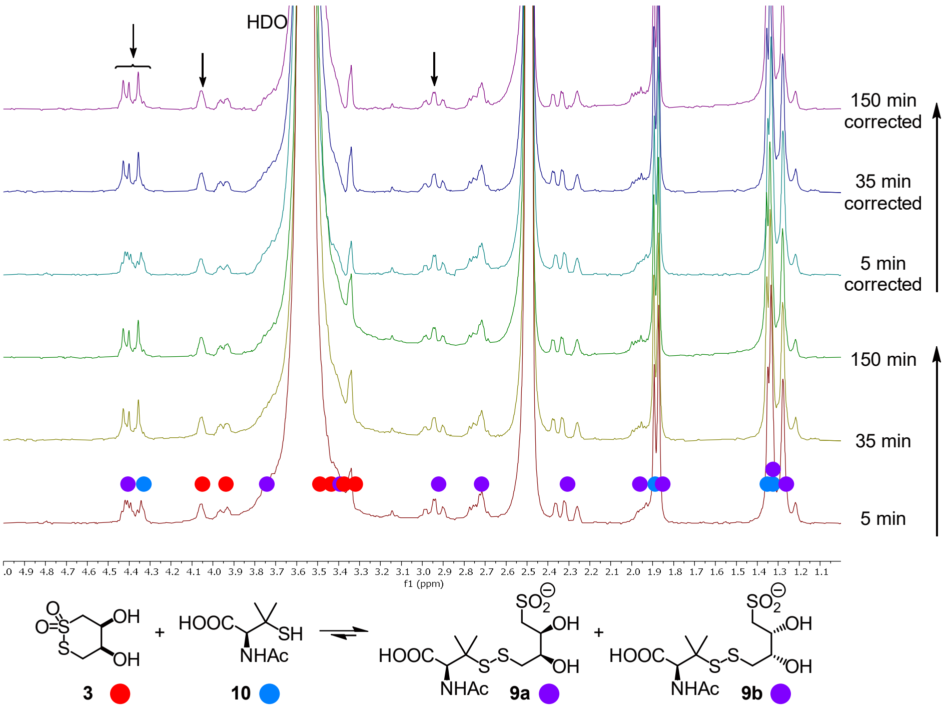

Figure S42. ^{1}H NMR spectra monitoring the exchange of **3** (5.0 mM) and **10** (5.0 mM) in a deuterated sodium phosphate solution (20 mM) in D_2O/DMSO-d_6 2:98 (Table 1, entry 10) before (bottom) and after (top) manual baseline correction. Integration was performed after manual baseline corrections, and the values may have inaccuracy due to the big peak of HDO.

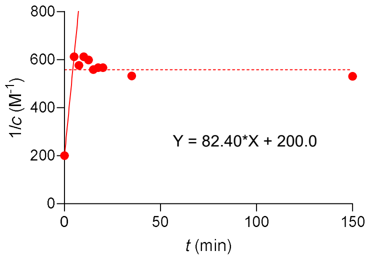

Figure S43. Inverse of concentration *c* of **3** as a function of exchange time *t* with **10** with a dotted line indicating the equilibrium. The first two points (t = 0, 5 min) were used for the estimation of the rate constant with Equation (S6), $k_1 > 1.4\ M^{-1}\ s^{-1}$. Equilibrium constant K was calculated based on the data at t = 15 min following Equation (S17), $K = 7.3 \times 10^{2}\ M^{-1}$. The data were plotted after manual baseline correction of the spectra. No oxidation of **10** was observed within 360 min in the control experiment (Table 1, entry 10).

3.1.3. Desulfinative Ring Opening of Cyclic Carbonate 11

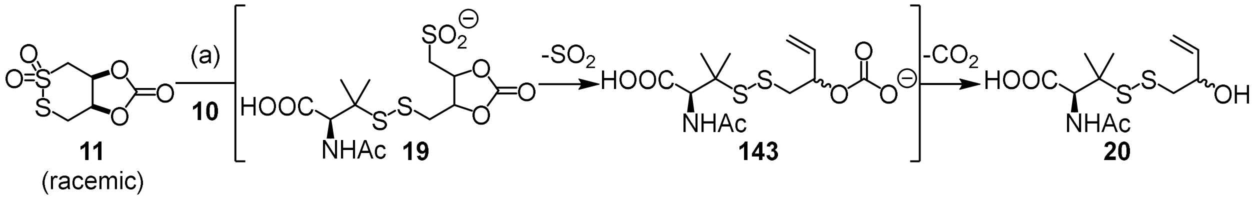

Scheme S30. (a) *N*-Ac-D-penicillamine (**10**), DMSO-d_6, rt.

Compounds 20. To DMSO-d_6 (540 µL), a stock solution of **11** (0.10 M, 30 µL, 3.0 µmol) in DMSO-d_6 and a stock solution of **10** (0.10 M, 30 µL, 3.0 µmol) in DMSO-d_6 were successively added. The mixture was vigorously stirred at room temperature. The solution was transferred immediately to an NMR tube and NMR data were recorded repeatedly. The reaction initially gave exchange products **19**, then **19** were converted into **20** spontaneously. The products **20** were identified by ^{1}H NMR (400 MHz): 8.12 (d. $^3J_{HH}$ = 8.9 Hz, 1H), 5.82/5.81 (ddd, $^3J_{HH}$ = 17.2, 10.3, 5.7 Hz, 1H), 5.21 (ddd, $^2J_{HH}$ = 1.8, $^3J_{HH}$ = 17.2, $^4J_{HH}$ = 2.3 Hz, 1H), 5.06 (ddd, $^2J_{HH}$ = 1.8, $^3J_{HH}$ = 10.3, $^4J_{HH}$ = 1.8 Hz, 1H), 4.46/4.44 (d, $^3J_{HH}$ = 8.9 Hz, 1H), 4.16 – 4.09 (m, 1H), 2.82 – 2.74 (m, 2H), 1.88/1.87 (s, 3H), 1.35/1.34 (s, 3H), 1.28 (s, 3H).

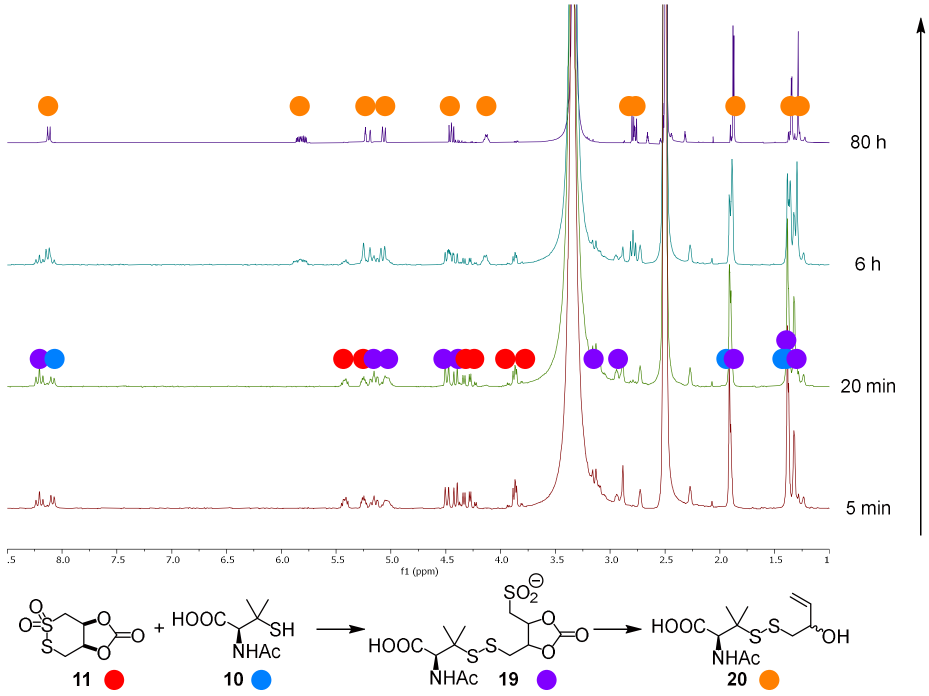

Figure S44. ^{1}H NMR spectra monitoring the exchange of **11** and **10** in DMSO-d_6.

3.2. UV-Vis Spectroscopy

3.2.1. Determination of Extinction Coefficients

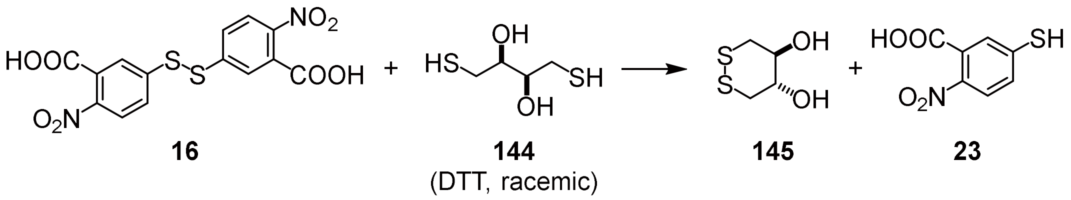

Scheme S31. Reduction of DTNB **16** with DTT **144** to form TNB **23**.

Determination of the isosbestic point for different DMF/H_2O ratios. To a mixture of DMF and H_2O (1.66 mL, final water content: 2 – 10%) in a glass cuvette (*l* = 1 cm), PBS buffer (x1, 40 µL) and a freshly-prepared stock solution of DTT (**144**, 2.0 mM in DMF, 200 µL, 200 µM final concentration) were added and a blank measurement was performed. Then a stock solution of DTNB (**16**, 100 µM in DMF, 100 µL, 5.0 µM final concentration) was added and vigorously stirred to give 10 µM solution of TNB (**23**). Absorption spectra were collected until no further change was observed. The blank spectrum was subtracted from the original spectra to give those reported in Figure S45. Similar solvent polarity dependence of λ_{max} was reported previously.[S35]

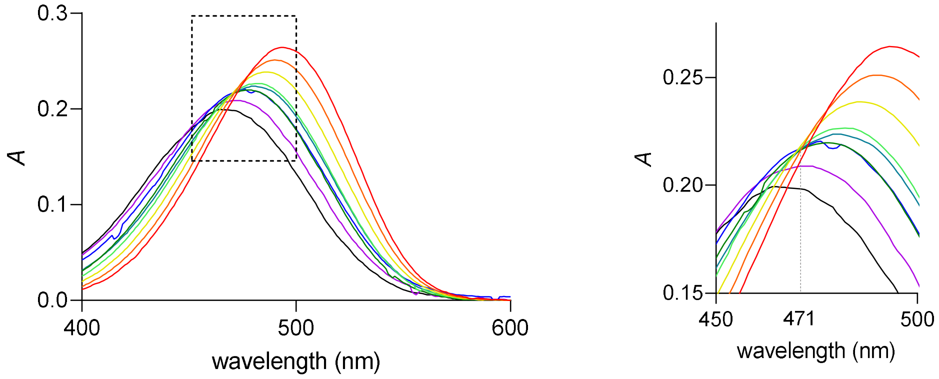

Figure S45. Absorption spectra of **23** generated from the reaction of **16** and **144** in PBS-buffered DMF with 2 – 10% H_2O; red: 2%; orange: 3%; yellow: 4%; light green: 5%; green: 6%; turquoise: 7%; blue: 8%; purple: 9%; black: 10%. Isosbestic point = 471 nm (3 – 8% H_2O).

Determination of the extinction coefficient at the isosbestic point. To a mixture of DMF (1.88 mL) and H_2O (60 µL, final water content: 5%) in a glass cuvette (1 cm), PBS buffer (x1, 40 µL) and a freshly-prepared stock solution of DTT (**144**, 20 mM in DMF, 20 µL, 200 µM final concentration) were added and a blank measurement was performed. Then a stock solution of DTNB (**16**, 1.0 µM in DMF, 1.0 µL) was added and vigorously stirred stirred to give 1.0 µM solution of TNB (**23**). Absorption spectra were collected until no further change was observed. The same operation was repeated to get spectra of up to 10 µM TNB solution. The blank spectrum was subtracted from the original spectra, and absorbance at 471 nm was plotted as a function of concentration of **23** to give the standard curve in Figure S46. The molar extinction coefficient was calculated to be $\varepsilon_{471} = 2.48 \times 10^4$ cm^{-1} M^{-1} (PBS-buffered 5% H_2O in DMF).

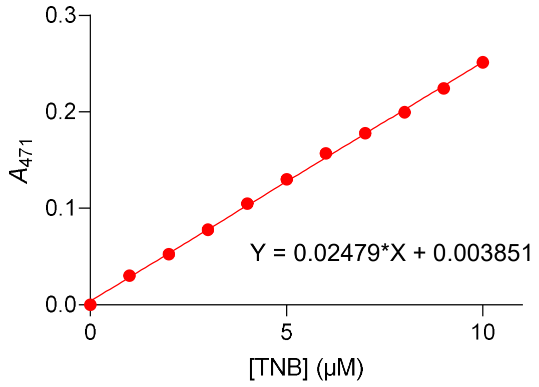

Figure S46. TNB concentration standard curve to give molar extinction coefficient of TNB (**23**) as the slope, $R^2 = 0.9991$.

3.2.2. Exchange of Sulfinates with Disulfides

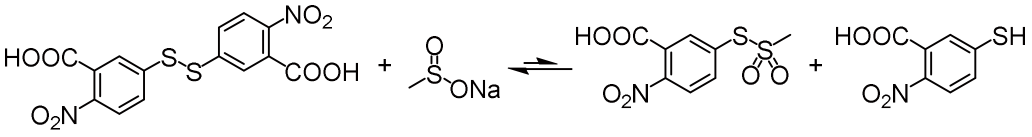

Scheme S32. Cleavage of DTNB **16** with $MeSO_2Na$ **21** to form TNB **23**.

To a mixture of DMF and H_2O (1.94 mL, final water content: 3 – 8%) in a glass cuvette (1 cm), PBS buffer (x1, 40 μL) and a stock solution of DTNB (**16**, 10 mM in DMF, 5.0 μL, 25 μM final concentration) were successively added and a blank measurement was performed. Then a freshly-prepared solution of $MeSO_2Na$ (**21**, 5.0 mM in DMF, 10 μL, 25 μM final concentration) was added and the mixture was vigorously stirred for 10 s. Absorption spectra were collected every 30 s until no further change was observed. The blank spectrum was subtracted from the original spectra, and absorbance at 471 nm was plotted as a function of time to give those reported in Figure S47a. Equilibrium constants were calculated based on the absorbance at 490 s (Figure S47b, Figure 7B).

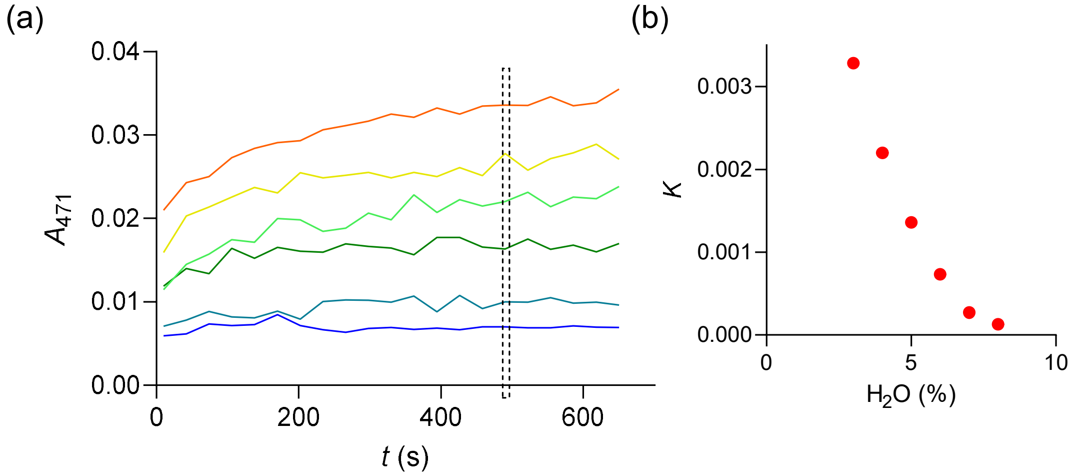

Figure S47. (a) Absorbance at 471 nm as a function of exchange time *t* and (b) equilibrium constant *K* in the reaction of **16** and **21**; orange: 3%; yellow: 4%; light green: 5%; green: 6%; turquoise: 7%; blue: 8%.

3.2.3. Exchange of Thiols with Disulfides

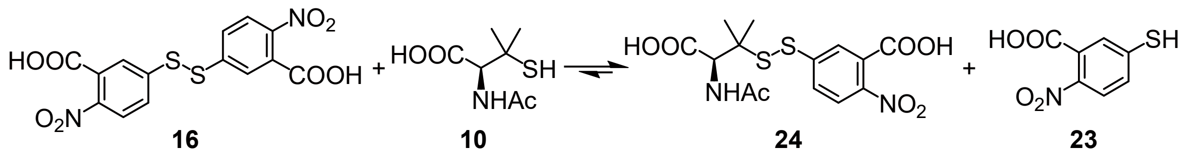

Scheme S33. Cleavage of DTNB **16** with *N*-Ac-D-penicillamine **10** to form TNB **23**.

To a mixture of DMF and H_2O (1.94 mL, final water content: 3 – 8%) in a glass cuvette (1 cm), PBS buffer (x1, 40 μL) and a stock solution of DTNB (**16**, 1.0 mM in DMF, 10 μL, 5.0 μM final concentration) were successively added and a blank measurement was performed. Then a freshly-prepared solution of *N*-Ac-D-penicillamine (**10**, 1.0 mM in DMF, 10 μL, 5.0 μM final concentration) was added and the mixture was vigorously stirred for 10 s. Absorption spectra were collected every 120 s until no further change was observed. The blank spectrum was subtracted from the original spectra, and absorbance at 471 nm was plotted as a function of time to give those reported in Figure S48a. Equilibrium constants were calculated based on the stabilized absorbance (Figure S48b, Figure 7B).

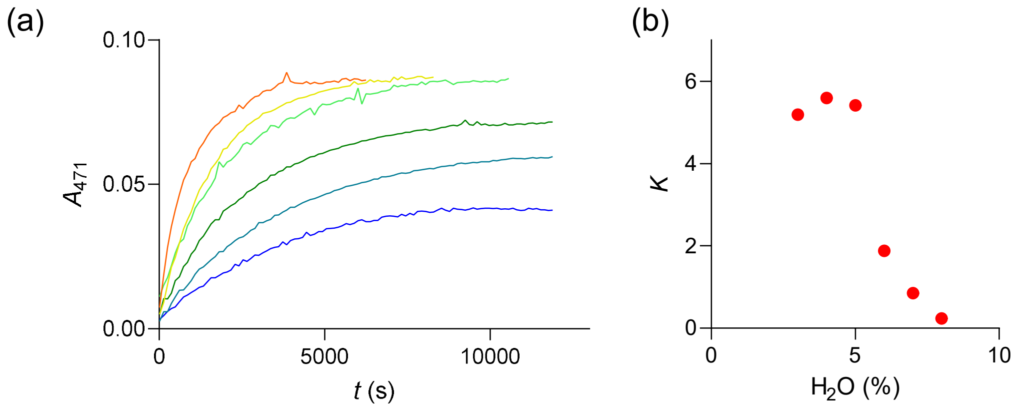

Figure S48. (a) Absorbance at 471 nm as a function of exchange time *t* and (b) equilibrium constant *K* in the reaction of **16** and **10**; orange: 3%; yellow: 4%; light green: 5%; green: 6%; turquoise: 7%; blue: 8%.

3.3. Fluorescence Spectroscopy

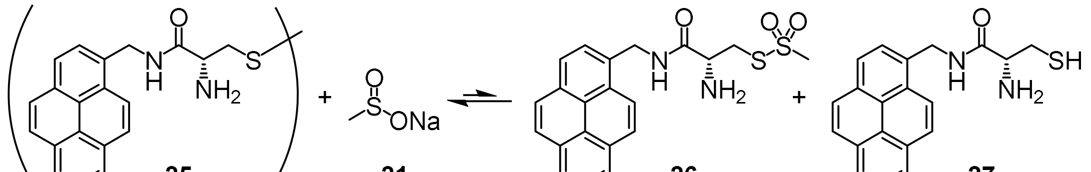

Scheme S34. Cleavage of bis-pyrene **25** with $MeSO_2Na$ **21** to form mono-pyrenes **26** and **27**.

To a mixture of DMF and H_2O (1.86 mL, final water content: 5 or 50%) in a quartz cuvette (1 cm), PBS buffer (x1, 40 μL) and a stock solution of $(PyCys)_2$[S36] (**25**, 0.20 mM in DMSO, 0.5 μL, 50 nM final concentration) was added and an emission spectrum (λ_{ex} = 344 nm) was collected. Then a freshly-prepared stock solution of $MeSO_2Na$ (**21**, 10 mM in DMF, 100 μL, 500 μM final concentration) was added, and emission spectra were collected over 90 min.

4. High-Content High-Throughput (HCHT) Inhibitor Screening

4.1. Cell Culture

As in ref S1. Namely, Human cervical cancer-derived HeLa Kyoto cells were cultured in DMEM (GlutaMAX, 4.5 g/L D-glucose, with phenol red) medium containing 10% fetal bovine serum (FBS) and 1% Penicillin/Streptomycin (PS). The cells were grown on a 25 cm^3 tissue culture flask (TPD Corporation) under 5% CO_2 humidified atmosphere at 37 °C. Cells were detached by treatment with 1.5 mL of TrypLE Express at 37 °C for 5 min, followed by the addition of 6 mL of DMEM (GlutaMAX, 4.5 g/L D-glucose, with phenol red) medium at 37 °C. The cells were resuspended in DMEM (GlutaMAX, 4.5 g/L D-glucose, with phenol red) medium and plated according to the concentration needed.

4.2. Cellular Uptake Experiments

HeLa Kyoto cells were seeded at 8 $\times 10^4$ cells/mL in FluoroBrite DMEM + 10% FBS on µ-Plate 96-well Black ibiTreat sterile and incubated at 37 °C with 5% CO_2 for 24 h. After removing the medium, the cells were washed with PBS (3 × 3 mL/well), fresh FluoroBrite DMEM medium (4 × 150 µL/well) was added into the wells using a plate washer (Biotek EL406®), keeping a final volume of 135 µL/well. Then, the various concentrations of transporters **28**, **29** and **30** (10× final concentration in PBS) were added (15 µL/well) to give final concentrations of 10, 20 and 50 µM. After 30 min of incubation at 37 °C with 5% CO_2, the cells were washed with the method described above and kept in clean FluoroBrite DMEM. For live cell imaging, samples were kept at 37 °C with 5% CO_2 during the microscope imaging. The distribution of fluorescence stained cells was analyzed without fixing using an IXM-C automated microscope. 4 images per well were recorded using 10× objective lens, and fluorescent images were acquired with three channels, blue with 377/50 nm excitation filter and 477/60 nm emission filter, green for FITC transporter with 475/34 nm excitation filter and

536/40 nm emission filter, and red with 531/40 nm excitation filter and 593/40 nm emission filter.

4.3. HCHT Inhibitor Screening

Pre-Incubation Method. As in ref. S1. HeLa Kyoto cells were seeded at 8 $\times 10^4$ cells/mL in FluoroBrite DMEM + 10% FBS on μ-Plate 96-well Black ibiTreat sterile and incubated at 37 °C with 5% CO_2 for 24 h. Then, the medium was removed and cells were washed with PBS (3 × 3 mL/well), fresh FluoroBrite DMEM medium (4 × 150 μL/well) was added into the cells using a plate washer (Biotek EL406®), keeping a final volume of 135 μL/well. Different concentrations of the inhibitors were prepared by serial dilution with 10% final concentration DMSO (10× in PBS). The solution of compounds **4** and **33** was prepared final DMSO concentration of 10% in each dilution (10× in PBS). These inhibitor solutions (10× final concentration), transporter **28** (10× in DMEM), transporters **31** and **32** (10× in PBS) a solution of HOE (100 μg/mL) and PI (10 μg/mL) in PBS were prepared freshly in a 96-well V-bottom plate before adding to the cells. The inhibitor solutions from the V-bottom plate were added to the cells (15 μL/well) in a final volume of 150 μL/well using an electronic multichannel pipette and cells were incubated for 1 h at 37 °C with 5% CO_2. After this incubation, cells were washed again with PBS and DMEM using the plate washer (in the way mentioned above), and transporter **28**, **31** or **32** from the V-bottom plate was added (15 μL/well) to the cells in a final volume of 150 μL/well. A final concentration of each transporter **28**, **31** and **32** was 5 μM, 10 μM and 0.5 μM, respectively, except for the control wells, where only PBS was added (15 μL/well). Cells treated with transporter **28** or **31** were incubated for 30 min, and cells treated with **32** were incubated for 2 h at 37 °C with 5% CO_2. After that, the cells were washed with PBS and DMEM using the plate washer, and the solution of Hoechst 33342 and PI from the V-bottom plate was added (15 μL/well) to the cells in a final volume of 150 μL/well. After 15 min of incubation at 37 °C with 5% CO_2, the cells were washed and kept in clean FluoroBrite

DMEM. For live cell imaging, samples were kept at 37 °C with 5% CO_2 during the microscope imaging. The distribution of fluorescence stained cells was analyzed without fixing using an IXM-C automated microscope. 4 images per well were recorded using 10× objective lens, and fluorescent images were acquired with three channels, blue for Hoechst 33342 with 377/50 nm excitation filter and 477/60 nm emission filter, green for FITC transporter with 475/34 nm excitation filter and 536/40 nm emission filter, and red for PI 531/40 nm excitation filter and 593/40 nm emission filter.

Co-Incubation Method. The overall process is the same as a pre-incubation method, except for the washing process of cells after incubation with inhibitors. After incubation with inhibitors for 1 h, the transporter was added immediately to the cells without washing the plates.

4.4. Data Analysis for HCHT Screening

SDCM images resulting from the cellular uptake experiment and inhibitor screening using transporters were automatically analyzed and quantified by the described protocol in ref. S1 and S36. Relative fluorescent intensity values per cell for each condition in the presence of inhibitors were normalized to the same parameter for the condition with only the transporter (no inhibitor, $I_{rel} = 1$) or for the condition with no transporter (HOE and PI only, $I_{rel} = 0$). Duplicates were performed in each condition and averaged. The resulting dependence of the relative fluorescent intensity values (I_{rel}) to the concentration of inhibitors ($c_{inhibitor}$) was plotted and fitted with Equation (S18) to retrieve the half maximal inhibitory concentration (IC_{50}) and the Hill coefficient (n). MIC values were estimated from the fit curve as the concentration at which 15% of uptake was inhibited.

$$I_{\mathrm{rel}} = \frac{1}{\left(1 + \left(\frac{\mathrm{IC}_{50}}{c_{\mathrm{inhibitor}}}\right)^{-n}\right)} \quad \text{(S18)}$$

Relative cell viability (RV) for each condition in the presence of inhibitors was calculated as the count of Hoechst 33342 stained cells minus the count of PI stained cells divided by the count of Hoechst 33342 stained cells only for each set of experiments. The resulting dependence of the relative cell viability (RV_{rel}) to the concentration of inhibitors ($c_{\mathrm{inhibitor}}$) was plotted and fitted with Equation (S19) to retrieve the concentration causing 50% cell growth inhibition (RV_{50}) value and the Hill coefficient (n).

$$RV_{\mathrm{rel}} = \frac{1}{\left(1 + \left(\frac{\mathrm{RV}_{50}}{c_{\mathrm{inhibitor}}}\right)^{-n}\right)} \tag{S19}$$

Note: Transporters **28**, **31** and **32** were not toxic for the condition performed in this study, the count value of living cells with the addition of transporters, Hoechst 33342 and PI is always similar to that of living cells with the only addition of Hoechst 33342 and PI.

4.5. Results of HCHT Inhibitor Screening

Table S1. Screening of inhibitor candidates in Figure S1 for inhibition of cellular uptake.

Entry	I[a]	T[b]	cond[c]	MIC[d] (μM)	IC_{50}[e] (μM)	RV_{50}[f] (μM)
1	**6**	**31**	C	<1	5 ± 1	15 ± 1
2	**13**	**31**	C	>10	>10	29 ± 6
3	**50**	**31**	C	5.6	20 ± 3	≈20
4	**51**	**31**	C	11	>50	>50
5	**52**	**31**	C	7.6	26 ± 4	>50
6	**53**	**28**	P	2.1	>10	>10
7	**54**	**28**	P	3.5	>10	>10
8	**55**	**31**	C	5.7	40 ± 10	38 ± 1
9	**56**	**28**	P	2.9	>5	8 ± 1
10	**57**	**28**	P	1.6	10 ± 4	>20
11	**58**	**28**	P	>20	>20	>20
12	**59**	**28**	P	16	>20	>20
13	**60**	**31**	C	12	50 ± 10	>50
14	**61**	**31**	C	-	>50	>50
15	**62**	**31**	C	2.8	21 ± 3	>50
16	**63**	**31**	C	3.2	13 ± 1	>50
17	**64**	**31**	C	1.3	30 ± 20	26 ± 3
18	**65**	**31**	C	41	>50	>50
19	**66**	**31**	C	11	19 ± 1	23 ± 2

Table S1 (continued). Screening of inhibitor candidates in Figure S1 for inhibition of cellular uptake.

Entry	I[a]	T[b]	cond[c]	MIC[d] (μM)	IC_{50}[e] (μM)	RV_{50}[f] (μM)
20	**67**	**31**	C	1.1	>50	>50
21	**68**	**31**	C	7.1	43 ± 9	48 ± 3
22	**69**	**31**	C	32	70 ± 10	>50
23	**70**	**31**	C	13	>50	25 ± 0.5
24	**71**	**31**	C	1.5	12 ± 2	43 ± 5
25	**72**	**31**	C	3.7	8 ± 2	19 ± 1
26	**73**	**31**	C	3.9	9 ± 1	48 ± 5
27	**74**	**31**	C	3.7	15 ± 1	>50
28	**75**	**31**	C	17	>50	>50
29	**76**	**31**	C	2.1	>20	≈40
30	**77**	**31**	C	11	≈20	21 ± 3
31	**78**	**31**	C	9.5	40 ± 5	>50

[a]Inhibitors at varied concentrations. [b]Transporters at constant concentration (**28**, 5 μM, **31**, 10 μM). [c]Conditions; P = pre-incubation, C = co-incubation. [d]Concentration needed to reach 15% inhibition. [e]Concentration needed to reach 50% inhibition. [f]Concentration needed to lower relative viability (RV) by 50%.

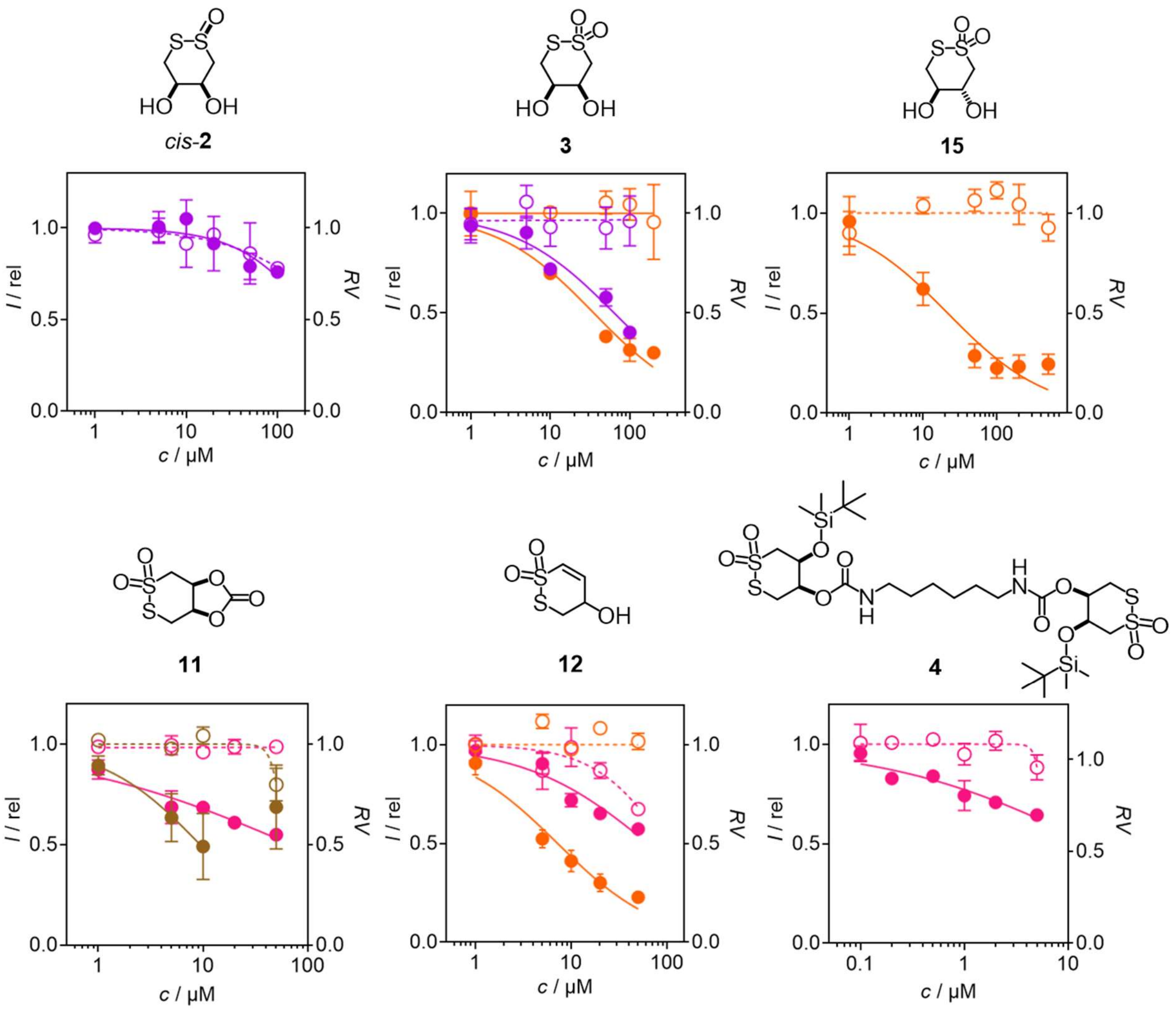

Figure S49. Automatically analyzed HCHT data showing relative fluorescence intensity (filled circles) and relative viability (empty circles) of HeLa Kyoto cells after incubation with each inhibitor for 1 h followed by incubation with the transporter for 30 min; orange circles: co-incubation with **31** (10 µM); brown circles: pre-incubation with **31** (10 µM); pink circles: co-incubation with **28** (5 µM); purple circles: pre-incubation with **28** (5 µM).

Table S2. Inhibition of cellular uptake and cell viability.

Entry[a]	I[b]	T[c]	Cond[d]	MIC[e] (μM)	IC_{50}[f] (μM)	n (IC_{50})[g]	RV_{50}[h] (μM)	n (RV_{50})[i]
2	**2**	**28**	P	>50	>100	-	>100	-
4	**3**	**28**	P	5	60 ± 10	0.7 ± 0.1	>1000	-
6	**3**	**31**	C	3	35 ± 6	0.7 ± 0.1	>1000	-
7	**15**	**31**	C	1.5	22 ± 5	0.6 ± 0.1	>500	-
8	**11**	**31**	P	1.5	9 ± 3	0.9 ± 0.3	>20	-
9	**11**	**31**	C	<5	>50	-	>50	-
10	**11**	**28**	C	<1	70 ± 20	0.4 ± 0.4	>50	-
11	**12**	**31**	C	<1	7.2 ± 0.9	0.8 ± 0.1	>50	-
12	**12**	**28**	C	4.8	60 ± 20	0.7 ± 0.1	≈100	1.1 ± 0.4
13	**4**	**28**	C	0.3	16 ± 7	0.4 ± 0.1	>5	-

[a]Entry in Table 2. [b]Inhibitors. [c]Transporters. [d]Conditions; P = pre-incubation, C = co-incubation. [e]Concentration needed to reach 15% inhibition. [f]Concentration needed to reach 50% inhibition. [g]Hill coefficient for inhibition of cellular uptake. [h]Concentration needed to lower relative viability (RV) by 50%. [i]Hill coefficient for cell viability.

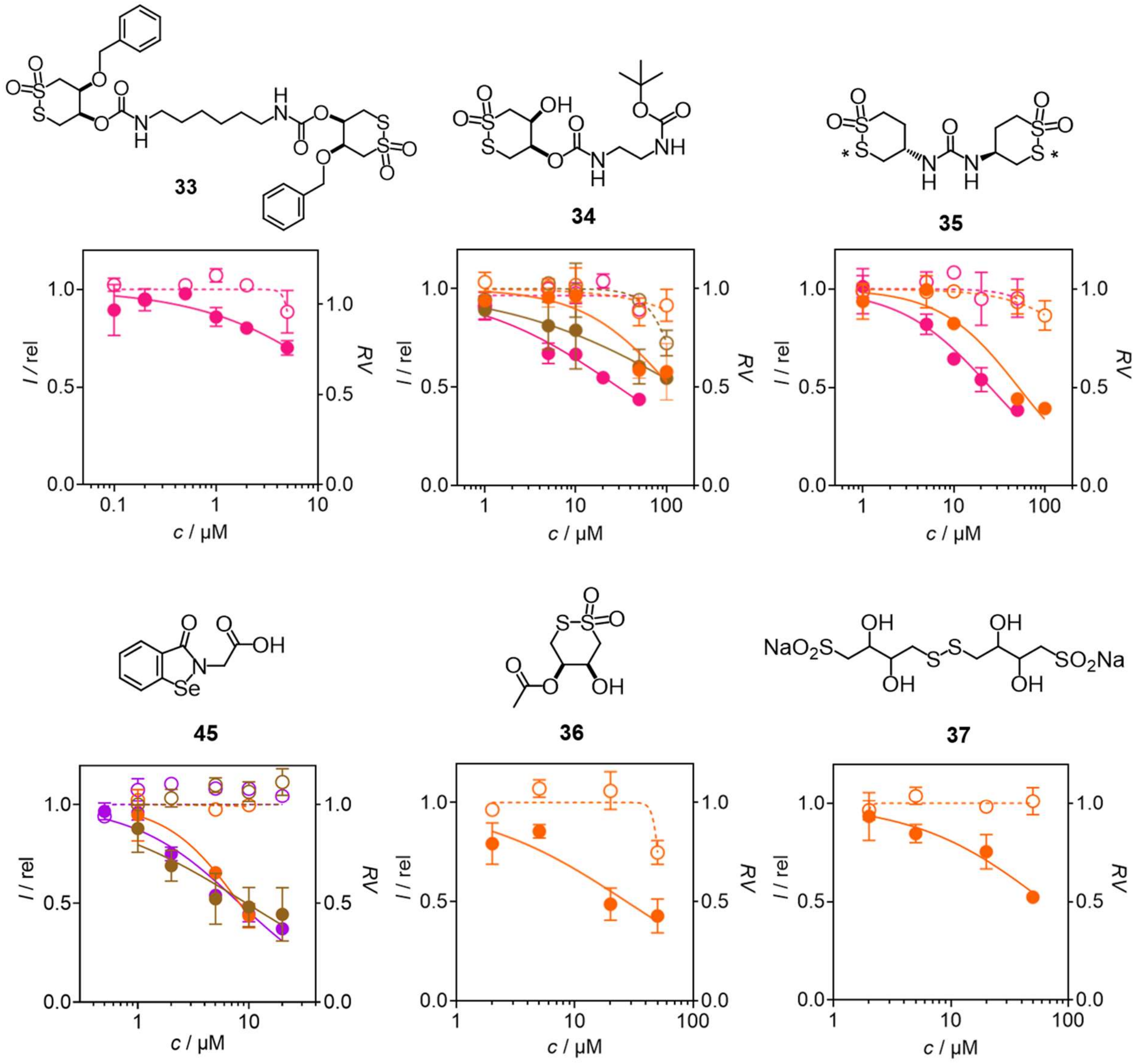

Figure S50. Automatically analyzed HCHT data showing relative fluorescence intensity (filled circles) and relative viability (empty circles) of HeLa Kyoto cells after incubation with each inhibitor for 1 h followed by incubation with the transporter for 30 min; orange circles: co-incubation with **31** (10 µM); brown circles: pre-incubation with **31** (10 µM); pink circles: co-incubation with **28** (5 µM); purple circles: pre-incubation with **28** (5 µM).

Table S3. Inhibition of cellular uptake and cell viability.

Entry[a]	I[b]	T[c]	Cond[d]	MIC[e] (μM)	IC_{50}[f] (μM)	n (IC_{50})[g]	RV_{50}[h] (μM)	n (RV_{50})[i]
14	**33**	**28**	C	1.3	>5	0.6 ± 0.2	>5	-
15	**34**	**31**	P	3.0	>100	0.4 ± 0.1	>100	-
16	**34**	**31**	C	16	>100	0.9 ± 0.2	>100	-
17	**34**	**28**	C	1.2	26 ± 5	0.6 ± 0.1	>50	-
18	**35**	**31**	C	9	51 ± 7	1.0 ± 0.2	>100	-
19	**35**	**28**	C	3.5	25 ± 3	0.9± 0.1	>50	-
20	**45**	**31**	P	<1	10 ± 3	0.6 ± 0.2	>20	-
21	**45**	**31**	C	2.2	8 ± 1	1.3 ± 0.3	>20	-
23	**45**	**28**	P	1.2	8 ± 1	0.9 ± 0.1	>20	-
24	**36**	**31**	C	2.1	27 ± 8	0.7 ± 0.2	>50	-
25	**37**	**31**	C	6.8	60 ± 20	0.8 ± 0.2	>50	-

[a]Entry in Table 2. [b]Inhibitors. [c]Transporters. [d]Conditions; P = pre-incubation, C = co-incubation. [e]Concentration needed to reach 15% inhibition. [f]Concentration needed to reach 50% inhibition. [g]Hill coefficient for inhibition of cellular uptake. [h]Concentration needed to lower relative viability (RV) by 50%. [i]Hill coefficient for cell viability.

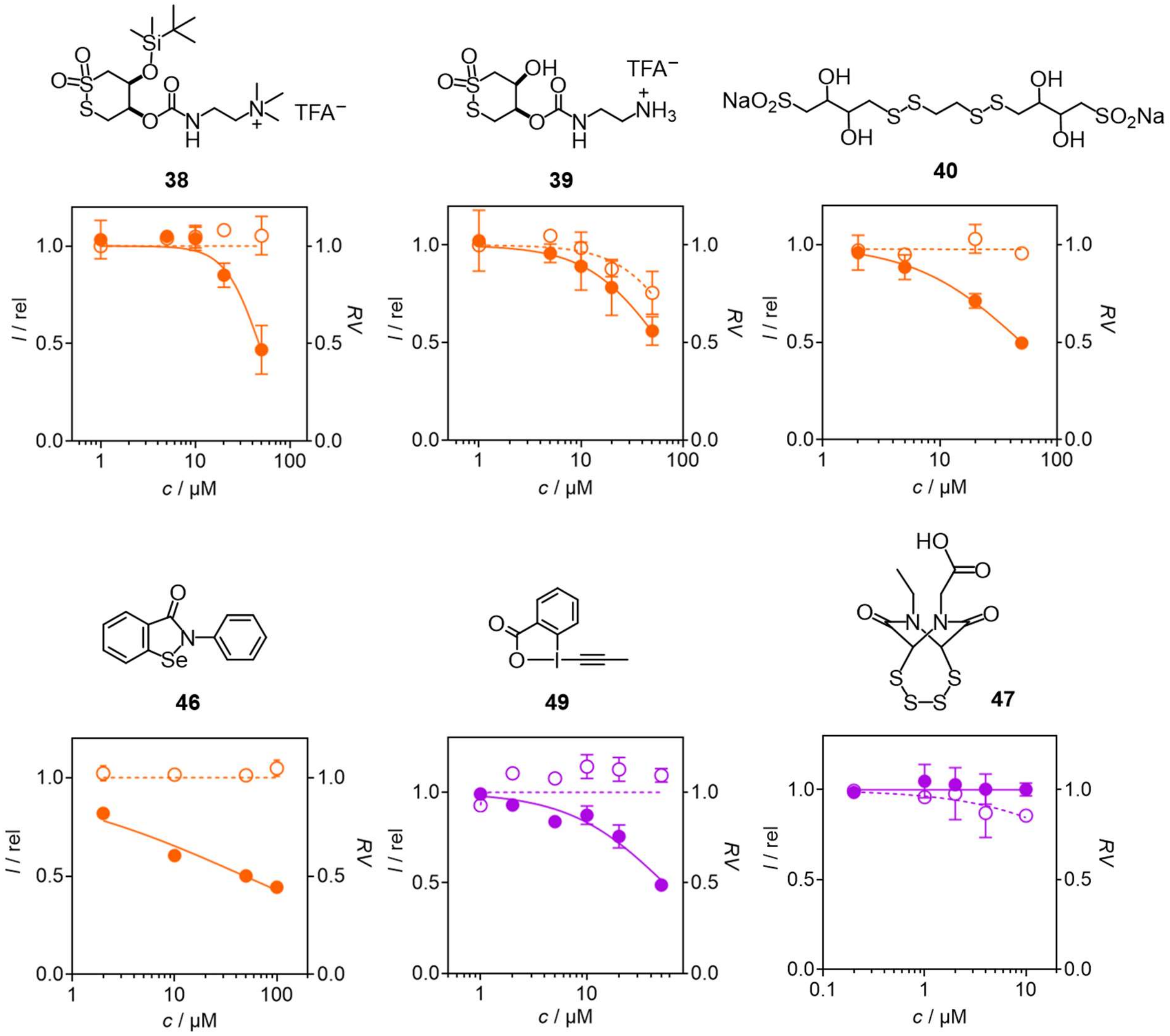

Figure S51. Automatically analyzed HCHT data showing relative fluorescence intensity (filled circles) and relative viability (empty circles) of HeLa Kyoto cells after incubation with each inhibitor for 1 h followed by incubation with the transporter for 30 min; orange circles: co-incubation with **31** (10 µM); purple circles: pre-incubation with **28** (5 µM).

Table S4. Inhibition of cellular uptake and cell viability.

Entry[a]	I[b]	T[c]	Cond[d]	MIC[e] (μM)	IC_{50}[f] (μM)	n (IC_{50})[g]	RV_{50}[h] (μM)	n (RV_{50})[i]
26	**38**	**31**	C	23	47 ± 4	2.4 ± 0.6	>50	-
27	**39**	**31**	C	14	>50	1.2 ± 0.3	>50	-
28	**40**	**31**	C	7.8	50 ± 7	0.9 ± 0.1	>50	-
29	**46**	**31**	C	<2	49 ± 7	1.7 ± 0.0	>100	-
31	**47**	**28**	P	>10	>10	-	>10	-
36	**49**	**28**	P	8.7	53 ± 8	1.0 ± 0.1	>50	-

[a]Entry in Table 2. [b]Inhibitors. [c]Transporters. [d]Conditions; P = pre-incubation, C = co-incubation. [e]Concentration needed to reach 15% inhibition. [f]Concentration needed to reach 50% inhibition. [g]Hill coefficient for inhibition of cellular uptake. [h]Concentration needed to lower relative viability (RV) by 50%. [i]Hill coefficient for cell viability.

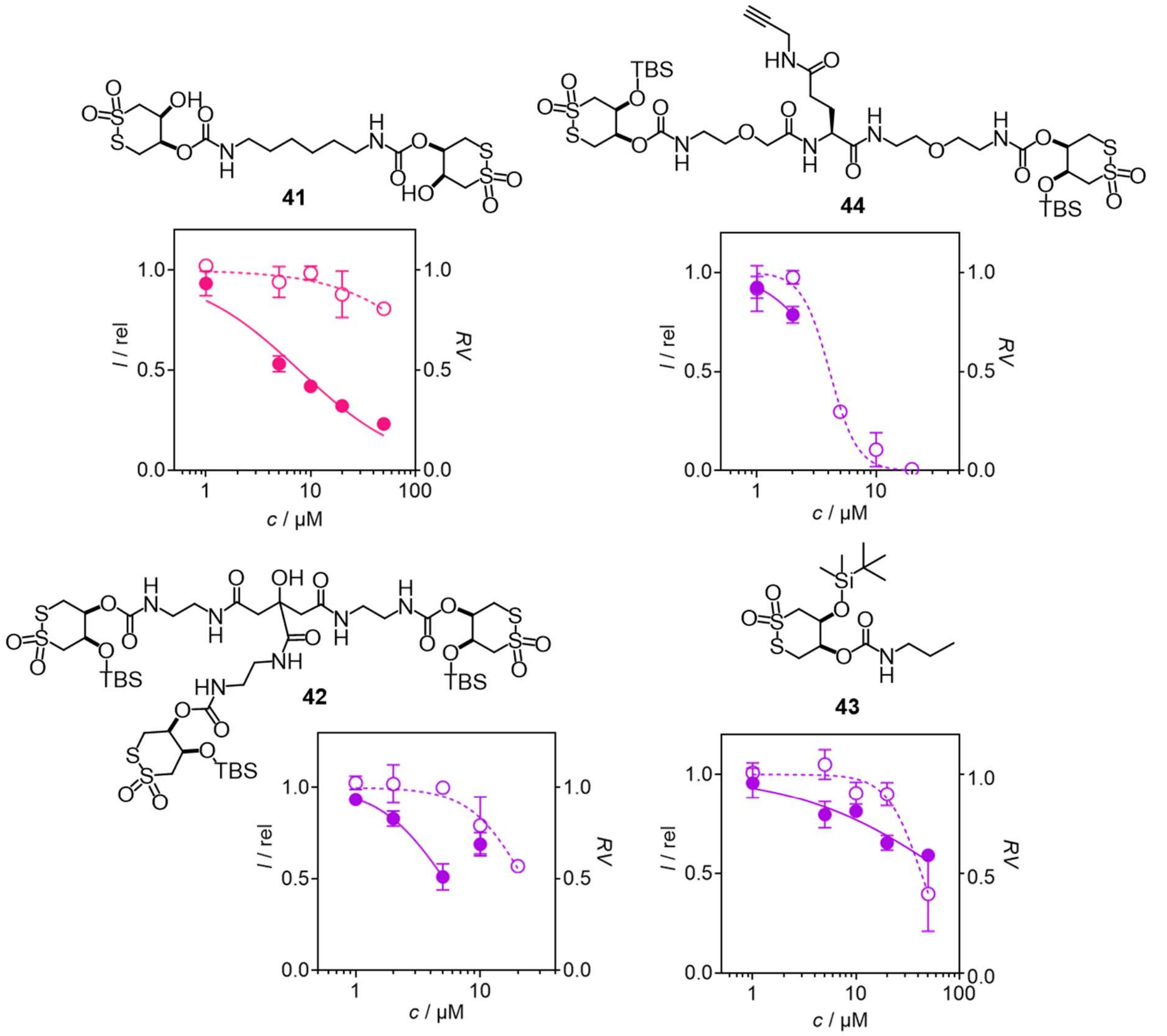

Figure S52. Automatically analyzed HCHT data showing relative fluorescence intensity (filled circles) and relative viability (empty circles) of HeLa Kyoto cells after incubation with each inhibitor for 1 h followed by incubation with the transporter for 30 min; pink circles: co-incubation with **28** (5 µM); purple circles: pre-incubation with **28** (5 µM).

Table S5. Inhibition of cellular uptake and cell viability.

Entry[a]	I[b]	T[c]	Cond[d]	MIC[e] (µM)	IC_{50}[f] (µM)	n (IC_{50})[g]	RV_{50}[h] (µM)	n (RV_{50})[i]
37	**41**	**28**	C	1.0	8 ± 1	0.8 ± 0.1	>50	-
38	**42**	**28**	P	1.8	5.1 ± 0.4	1.6 ± 0.2	22 ± 3	2.0 ± 0.5
39	**43**	**28**	P	4.1	>50	0.6 ± 0.1	43 ± 4	2.6 ± 0.7
40	**44**	**28**	P	-	-	-	4.1 ± 0.3	3.7 ± 0.9

[a]Entry in Table 2. [b]Inhibitors. [c]Transporters. [d]Conditions; P = pre-incubation, C = co-incubation. [e]Concentration needed to reach 15% inhibition. [f]Concentration needed to reach 50% inhibition. [g]Hill coefficient for inhibition of cellular uptake. [h]Concentration needed to lower relative viability (RV) by 50%. [i]Hill coefficient for cell viability.

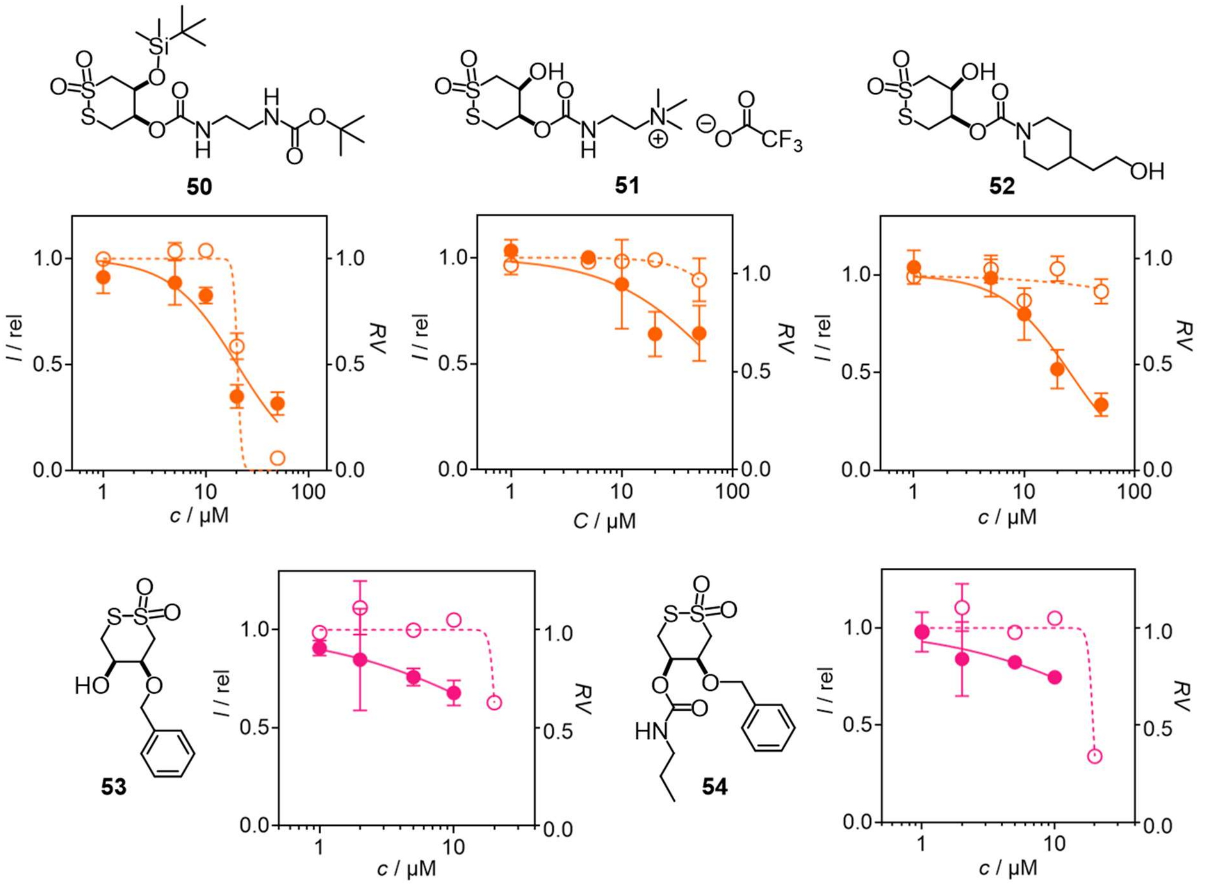

Figure S53. Automatically analyzed HCHT data showing relative fluorescence intensity (filled circles) and relative viability (empty circles) of HeLa Kyoto cells after incubation with each inhibitor for 1 h followed by incubation with the transporter for 30 min; orange circles: co-incubation with **31** (10 µM); pink circles: co-incubation with **28** (5 µM).

Table S6. Inhibition of cellular uptake and cell viability.

Entry[a]	I[b]	T[c]	Cond[d]	MIC[e] (μM)	IC_{50}[f] (μM)	*n* (IC_{50})[g]	RV_{50}[h] (μM)	*n* (RV_{50})[i]
3	**50**	**31**	C	5.6	20 ± 3	1.3 ± 0.3	≈20	-
4	**51**	**31**	C	11	>50	0.9 ± 0.4	>50	-
5	**52**	**31**	C	7.6	26 ± 4	1.4 ± 0.3	>50	-
6	**53**	**28**	P	2.1	>10	-	>10	-
7	**54**	**28**	P	3.5	>10	-	>10	-

[a]Entry in Table S1. [b]Inhibitors. [c]Transporters. [d]Conditions; P = pre-incubation, C = co-incubation. [e]Concentration needed to reach 15% inhibition. [f]Concentration needed to reach 50% inhibition. [g]Hill coefficient for inhibition of cellular uptake. [h]Concentration needed to lower relative viability (RV) by 50%. [i]Hill coefficient for cell viability.

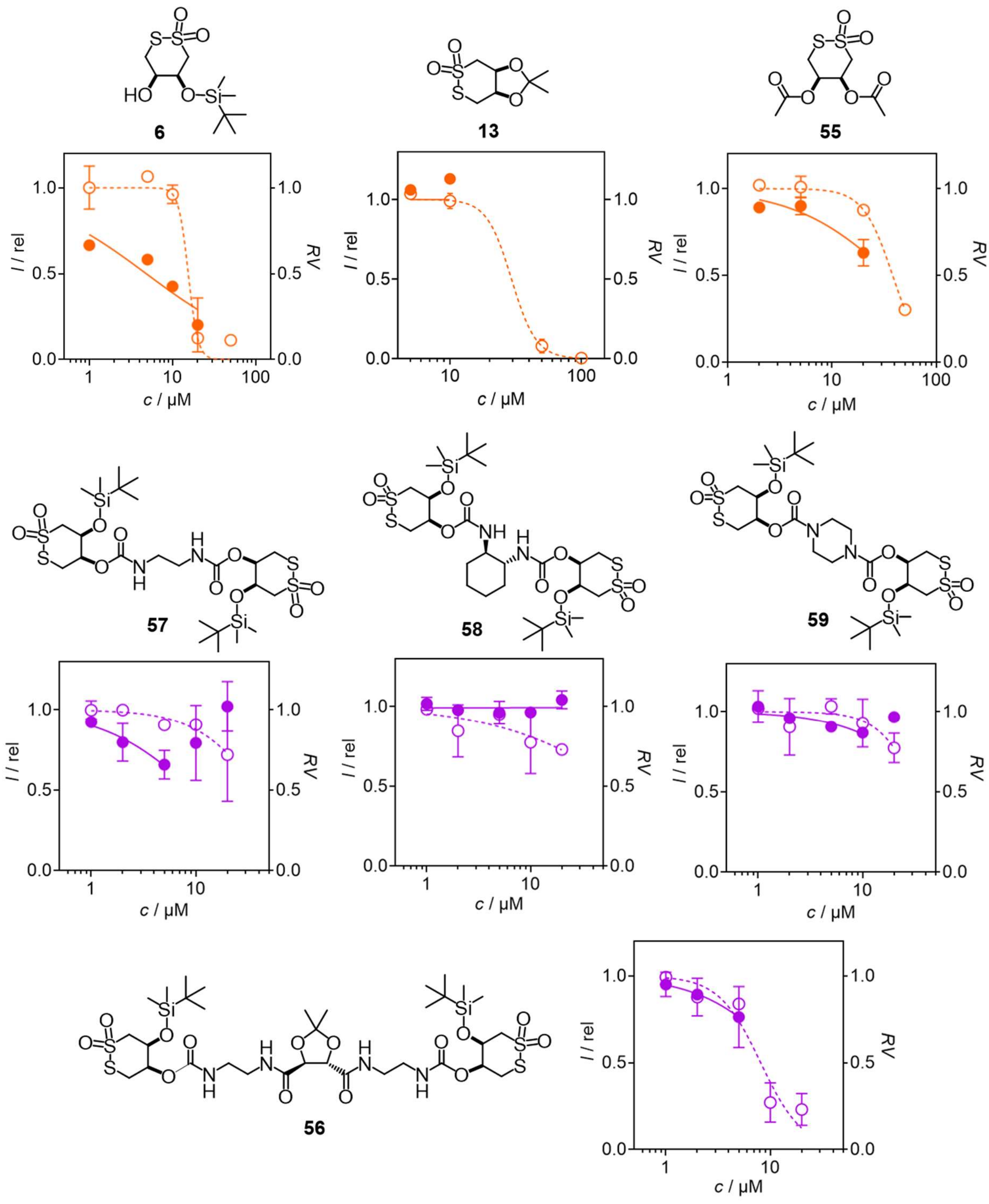

Figure S54. Automatically analyzed HCHT data showing relative fluorescence intensity (filled circles) and relative viability (empty circles) of HeLa Kyoto cells after incubation with each inhibitor for 1 h followed by incubation with the transporter for 30 min; purple circles: pre-incubation with **28** (5 µM), orange circles: co-incubation with **31** (10 µM).

Table S7. Inhibition of cellular uptake and cell viability.

Entry[a]	I[b]	T[c]	Cond[d]	MIC[e] (μM)	IC_{50}[f] (μM)	n (IC_{50})[g]	RV_{50}[h] (μM)	n (RV_{50})[i]
1	**6**	**31**	C	<1	5 ± 1	0.6 ± 0.2	15 ± 1	8 ± 3
2	**13**	**31**	C	>10	>10	-	29 ± 6	
8	**55**	**31**	C	5.7	40 ± 10	0.9 ± 0.2	38 ± 1	3.1 ± 0.3
9	**56**	**28**	P	2.9	>5	-	8 ± 1	2.2 ± 0.5
10	**57**	**28**	P	1.6	10 ± 4	1.0 ± 0.4	>20	-
11	**58**	**28**	P	>20	>20	-	>20	-
12	**59**	**28**	P	16	>20	-	>20	-

[a]Entry in Table S1. [b]Inhibitors. [c]Transporters. [d]Conditions; P = pre-incubation, C = co-incubation. [e]Concentration needed to reach 15% inhibition. [f]Concentration needed to reach 50% inhibition. [g]Hill coefficient for inhibition of cellular uptake. [h]Concentration needed to lower relative viability (RV) by 50%. [i]Hill coefficient for cell viability.

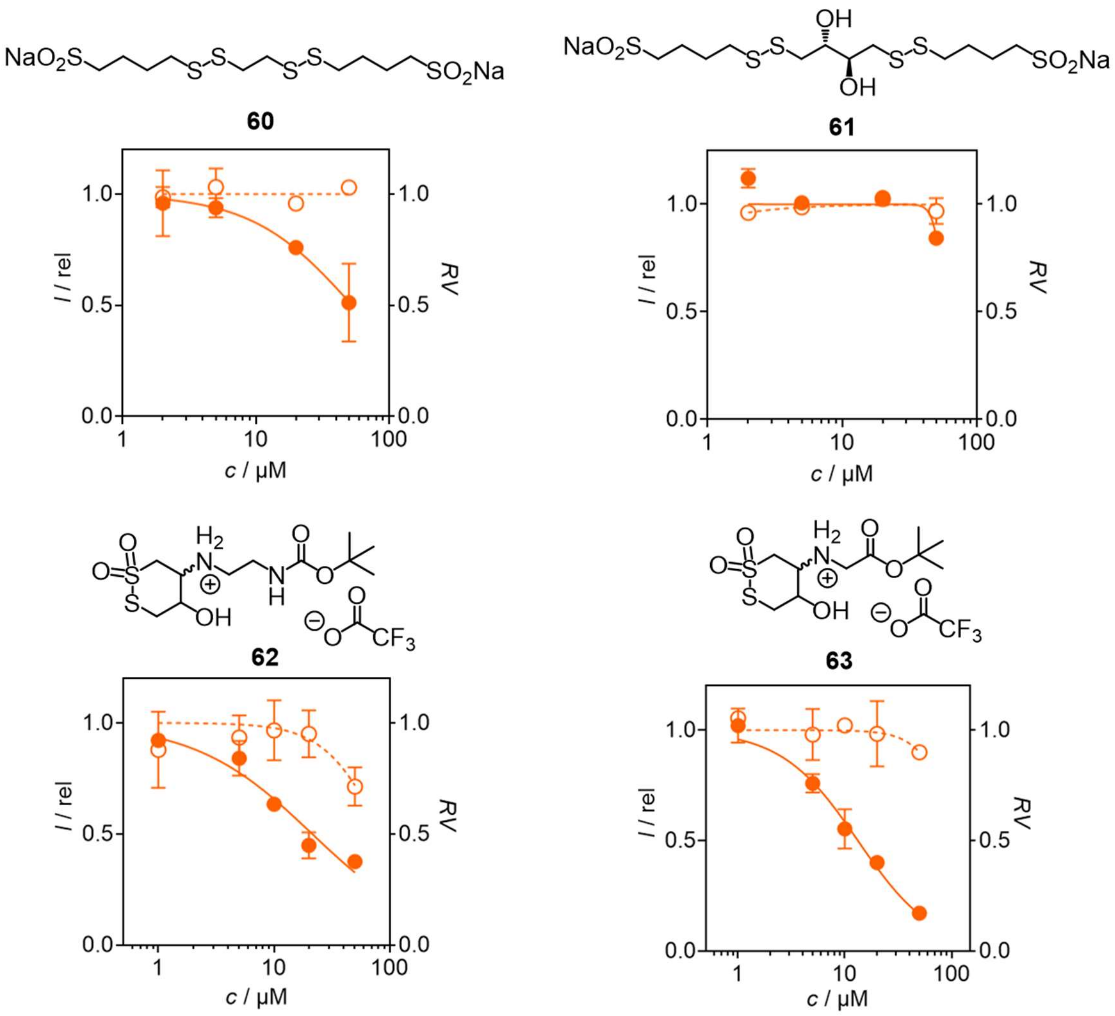

Figure S55. Automatically analyzed HCHT data showing fluorescence intensity (filled circles) and relative viability (empty circles) of HeLa Kyoto cells after incubation with each inhibitor for 1 h followed by co-incubation with transporter **31** (10 µM) for 30 min.

Table S8. Inhibition of cellular uptake and cell viability.

Entry[a]	I[b]	T[c]	Cond[d]	MIC[e] (μM)	IC_{50}[f] (μM)	n (IC_{50})[g]	RV_{50}[h] (μM)	n (RV_{50})[i]
13	**60**	**31**	C	12	50 ± 10	1.1 ± 0.4	>50	-
14	**61**	**31**	C	-	>50	-	>50	-
15	**62**	**31**	C	2.8	21 ± 3	0.9 ± 0.1	>50	-
16	**63**	**31**	C	3.2	13 ± 1	1.2 ± 0.1	>50	-

[a]Entry in Table S1. [b]Inhibitors. [c]Transporters. [d]Conditions; P = pre-incubation, C = co-incubation. [e]Concentration needed to reach 15% inhibition. [f]Concentration needed to reach 50% inhibition. [g]Hill coefficient for inhibition of cellular uptake. [h]Concentration needed to lower relative viability (RV) by 50%. [i]Hill coefficient for cell viability.

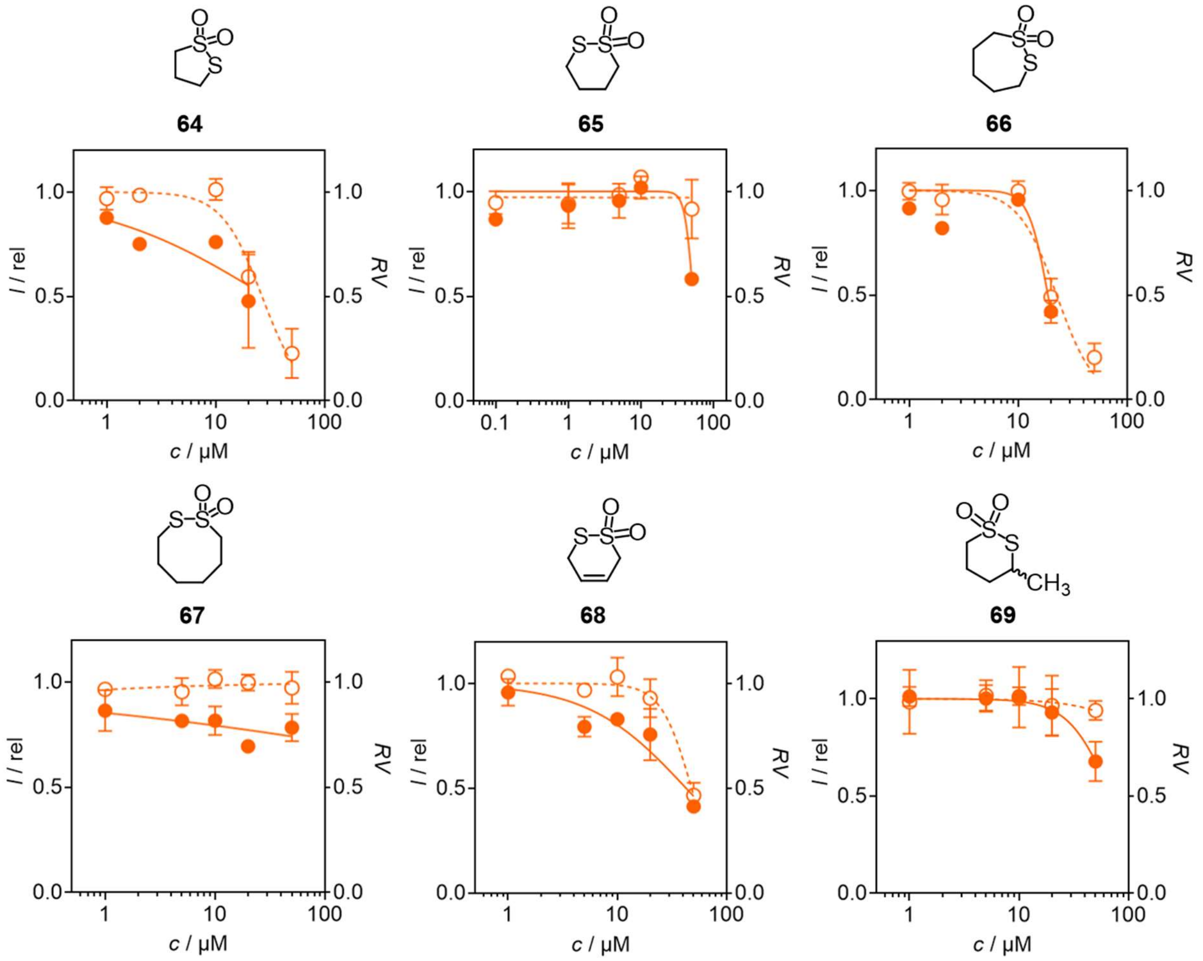

Figure S56. Automatically analyzed HCHT data showing fluorescence intensity (filled circles) and relative viability (empty circles) of HeLa Kyoto cells after incubation with each inhibitor for 1 h followed by co-incubation with transporter **31** (10 μM) for 30 min.

Table S9. Inhibition of cellular uptake and cell viability.

Entry[a]	I[b]	T[c]	Cond[d]	MIC[e] (μM)	IC_{50}[f] (μM)	n (IC_{50})[g]	RV_{50}[h] (μM)	n (RV_{50})[i]
17	**64**	**31**	C	1.3	30 ± 20	0.6 ± 0.2	26 ± 3	2.4 ± 0.5
18	**65**	**31**	C	41	>50	-	>50	-
19	**66**	**31**	C	11	19 ± 1	5 ± 3	23 ± 2	2.5 ± 0.6
20	**67**	**31**	C	1.1	>50	-	>50	-
21	**68**	**31**	C	7.1	43 ± 9	1.0 ± 0.2	48 ± 3	3.1 ± 0.8
22	**69**	**31**	C	32	70 ± 10	2.2 ± 0.9	>50	-

[a]Entry in Table S1. [b]Inhibitors. [c]Transporters. [d]Conditions; P = pre-incubation, C = co-incubation. [e]Concentration needed to reach 15% inhibition. [f]Concentration needed to reach 50% inhibition. [g]Hill coefficient for inhibition of cellular uptake. [h]Concentration needed to lower relative viability (RV) by 50%. [i]Hill coefficient for cell viability.

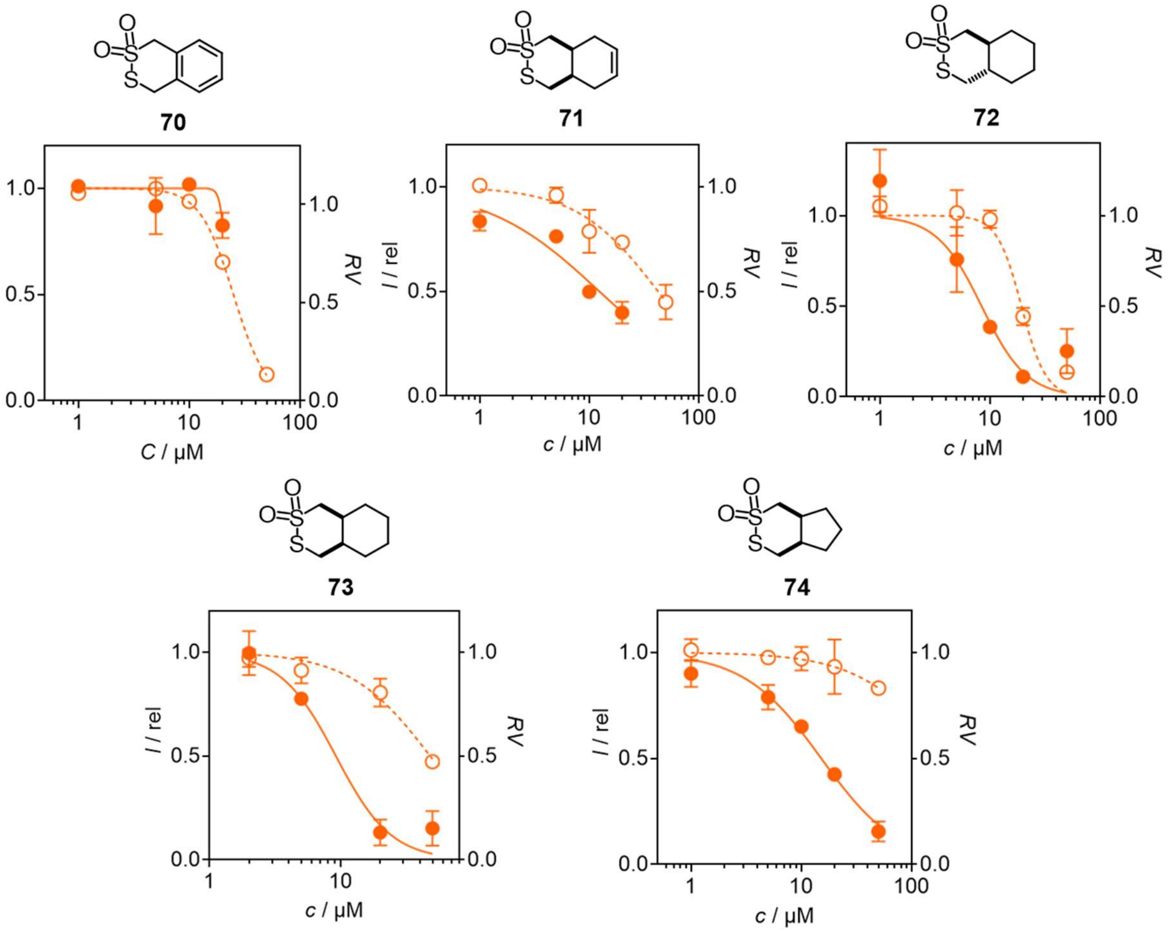

Figure S57. Automatically analyzed HCHT data showing fluorescence intensity (filled circles) and relative viability (empty circles) of HeLa Kyoto cells after incubation with each inhibitor for 1 h followed by co-incubation with transporter **31** (10 µM) for 30 min.

Table S10. Inhibition of cellular uptake and cell viability.

Entry[a]	I[b]	T[c]	Cond[d]	MIC[e] (μM)	IC_{50}[f] (μM)	n (IC_{50})[g]	RV_{50}[h] (μM)	n (RV_{50})[i]
23	**70**	**31**	C	13	>50	-	25 ± 0.5	2.9 ± 0.2
24	**71**	**31**	C	1.5	12 ± 2	0.8 ± 0.2	43 ± 5	1.2 ± 0.2
25	**72**	**31**	C	3.7	8 ± 2	2.1 ± 0.8	19 ± 1	4 ± 1
26	**73**	**31**	C	3.9	9 ± 1	2 ± 1	48 ± 5	1.4 ± 0.2
27	**74**	**31**	C	3.7	15 ± 1	1.2 ± 0.1	>50	-

[a]Entry in Table S1. [b]Inhibitors. [c]Transporters. [d]Conditions; P = pre-incubation, C = co-incubation. [e]Concentration needed to reach 15% inhibition. [f]Concentration needed to reach 50% inhibition. [g]Hill coefficient for inhibition of cellular uptake. [h]Concentration needed to lower relative viability (RV) by 50%. [i]Hill coefficient for cell viability.

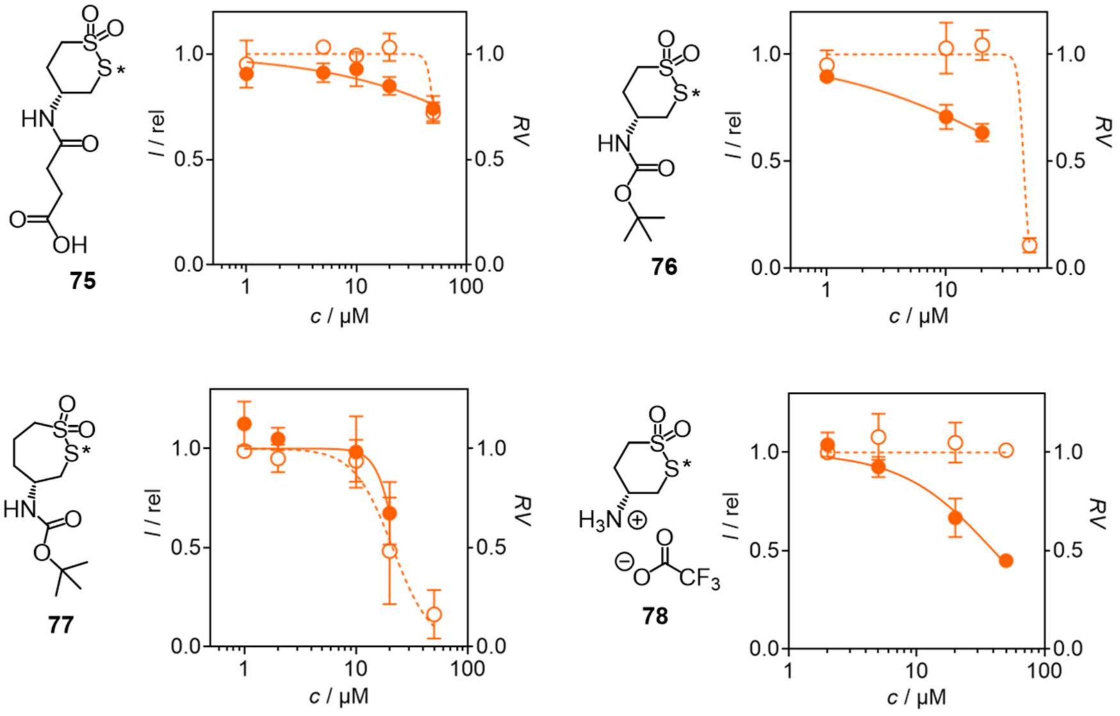

Figure S58. Automatically analyzed HCHT data showing fluorescence intensity (filled circles) and relative viability (empty circles) of HeLa Kyoto cells after incubation with each inhibitor for 1 h followed by co-incubation with transporter **31** (10 µM) for 30 min.

Table S11. Inhibition of cellular uptake and cell viability

Entry[a]	I[b]	T[c]	Cond[d]	MIC[e] (µM)	IC_{50}[f] (µM)	n (IC_{50})[g]	RV_{50}[h] (µM)	n (RV_{50})[i]
28	**75**	**31**	C	17	>50	-	>50	-
29	**76**	**31**	C	2.1	>20	-	≈40	-
30	**77**	**31**	C	11	≈20	-	21 ± 3	2.5 ± 0.8
31	**78**	**31**	C	9.5	40 ± 5	1.2 ± 0.2	>50	-

[a]Entry in Table S1. [b]Inhibitors. [c]Transporters. [d]Conditions; P = pre-incubation, C = co-incubation. [e]Concentration needed to reach 15% inhibition. [f]Concentration needed to reach 50% inhibition. [g]Hill coefficient for inhibition of cellular uptake. [h]Concentration needed to lower relative viability (RV) by 50%. [i]Hill coefficient for cell viability.

5. Inhibition of Lentiviral Vector Entry

As in ref. S1. Inhibition of SC2 lentivirus entry and cell viability measurement were performed as previously described[S1] by NEURIX (64, Avenue de la Roseraie 1205, Geneva, Switzerland). pCG1_SCoV-2 plasmid encoding SARS-CoV-2 S protein was provided by Prof. Dr. Stefan Pöhlmann (Deutsches Primatenzentrum, Leibniz- Institute for Primate research, Göttingen, Germany).

Lentivirus generation. As described in ref. S1. Namely, Standard lentivirus was generated using transient transfection of HEK 293T/17 cells with the CD511B plasmid coding for green fluorescent protein and Gaussia luciferase under the control of cytomegalovirus (CMV) and elongation factor-1 (EF1) promoters, respectively, the psPAX2 plasmid encoding gag/pol, and the pCAG-VSVG envelope plasmid, as described in ref. S37. To generate the SARS-CoV-2 pseudovirus, the pCAG-VSVG plasmid was replaced by the pCG1 plasmid expressing the C-terminally truncated SARS-CoV-2 spike protein harboring the D614G mutation under CMV promoter. Human lung epithelial A549 cells were cultivated in DMEM media supplemented with 10% FBS and 1% non-essential amino acid in a humidified incubator at 37°C with 5% CO_2, all from Thermofisher scientific.

Compound treatment. Stock solutions of inhibitors (10 mM, 50 mM or 500 mM) in DMSO were prepared freshly. Before the cell experiment, these solutions were diluted 1000 times with culture media to give each desired solution with only 0.1% of DMSO (avoid any off-target effect from DMSO). A549 cells were treated with these solutions (desired concentrations (1×) or 10 times diluted (10×)) for 1 h prior to the addition of the lentiviruses. 6 hours later the culture media containing both compounds and lentivirus was discarded and fresh culture media was added. 72 hours later, 10 µL of culture media was sampled and mixed with 50 µL of PBS containing 4 µM of Coelentrazine (Apollo Scientific), and luminescence generated by the gaussia luciferase reporter was measured. In parallel 10 µL of cell counting

kit WST8 (Sigma Aldrich) was added on cells to measure viability by absorbance at 450 nm according to manufacturer instructions.

Table S12. Inhibition of SC2 lentivector entry.[a]

Entry	I[b]	*c* (µM)[c]	VE (%)[d]	RV (%)[e]
1	**6**	5	105 ± 3	105 ± 7
2	**13**	5	80 ± 20	125 ± 5
3	**16**	5	130 ± 20	89 ± 4
4	**17**	5	130 ± 20	87 ± 4
5	**36**	50	34 ± 3	97 ± 6
6	**50**	5	100 ± 40	110 ± 10
7	**51**	5	86 ± 6	116 ± 4
8	**52**	5	93 ± 7	115 ± 9
9	**55**	5	89 ± 7	110 ± 10
10	**60**	5	200 ± 60	95 ± 5
11	**61**	5	250 ± 40	96 ± 4
12	**62**	5	106 ± 7	91 ± 6
13	**64**	5	110 ± 10	100 ± 5
14	**65**	5	130 ± 10	117 ± 3
15	**66**	5	100 ±10	102 ± 1
16	**67**	5	96 ±9	102 ± 7
17	**68**	5	120 ± 20	85 ± 2
18	**69**	5	230± 30	90 ± 3
19	**70**	5	83 ± 8	117 ± 5

Table S12 (continued). Inhibition of SC2 lentivector entry.[a]

Entry	I[b]	*c* (μM)[c]	VE (%)[d]	RV (%)[e]
20	**71**	5	230 ± 40	76 ± 2
21	**72**	5	320 ± 10	91 ± 8
22	**74**	5	240 ± 40	87 ± 1
23	**75**	50	88 ± 9	80 ± 10
24	**76**	5	110 ±20	103 ± 5
25	**77**	5	110 ± 20	99 ± 5
26	**78**	50	99 ±4	46 ± 5

[a]From normalized luminescence intensity of A549 human lung alveolar basal epithelium cells overexpressing ACE2 and TMPRSS2 after incubation with [b]inhibitor candidates at [c]concentrations (1 h) and lentivirus with D614G SC2 spike protein (6 h), followed by 3 days for luciferase expression, reported as [d]vector entry (VE) in % (n = 3, ± SD). [e]RV: Relative cell viability.

6. Computational Studies

Calculations were performed using the Gaussian 09 program,[S38] Structures of **1** and **3** in water were optimized using M06-2X/6-311++G** with the SMD model.[S39] For the most stable conformations obtained, structures of complexes with chloride or fluoride ion were optimized in gas phase to evaluate the intrinsic depth of σ-hole. Structures of complexes of 1,2-dithiane, 1,2-dithiane-1-oxide and 1,2-dithiane-1,1-dioxide with fluoride were also optimized in gas phase. Frequency calculations were performed at the same level to confirm minima (no negative frequencies). Structures were visualized with CYLView.

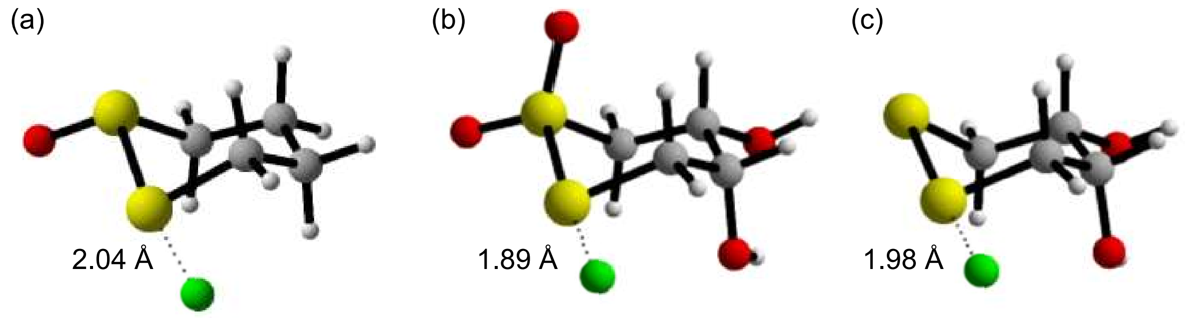

Figure S59. Fluoride complex of (a) 1,2-dithiane-1-*eq*-oxide, (b) **3** and (c) **1** calculated with M06-2X/6-311++G** (green: F^{-}, yellow: S, red: O, grey: C, white: H).

7. Supporting References

(S1) Cheng, Y.; Pham, A.-T.; Kato, T.; Lim, B.; Moreau, D.; López-Andarias, J.; Zong, L.; Sakai, N.; Matile, S. Inhibitors of Thiol-Mediated Uptake. *Chem. Sci.* **2021**, *12*, 626–631.

(S2) Law, B. K.; Castellano R. K., *PCT Int. Appl.*, **2019**, WO 2019241644 Al.

(S3) Rice, W. G.; Schultz, R. R.; Baker, D. C.; Henderson, L. E. *PCT Int. Appl.*, **1998**, WO 9801440 A2.

(S4) Saha, A.; Panda, S.; Paul, S.; Manna, D. Phosphate Bioisostere Containing Amphiphiles: A Novel Class of Squaramide-Based Lipids. *Chem. Commun.* **2016**, *52*, 9438–9441.

(S5) Lukesh, J. C.; Palte, M. J.; Raines, R. T. A Potent, Versatile Disulfide-Reducing Agent from Aspartic Acid. *J. Am. Chem. Soc.* **2012**, *134*, 4057–4059.

(S6) Wang, H.; He, S.; Deng, W.; Zhang, Y.; Li, G.; Sun, J.; Zhao, W.; Guo, Y.; Yin, Z.; Li, D.; Shang, L. Comprehensive Insights into the Catalytic Mechanism of Middle East Respiratory Syndrome 3C-Like Protease and Severe Acute Respiratory Syndrome 3C-Like Protease. *ACS Catal.* **2020**, *10*, 5871–5890.

(S7) Suhartono, M.; Schneider, A. E.; Dürner, G.; Göbel, M. W. Synthetic Aromatic Amino Acids from a Negishi Cross-Coupling Reaction. *Synthesis* **2010**, *2010*, 293–303.

(S8) Ferreira, R. B.; Law, M. E.; Jahn, S. C.; Davis, B. J.; Heldermon, C. D.; Reinhard, M.; Castellano, R. K.; Law, B. K. Novel Agents That Downregulate EGFR, HER2, and HER3 in Parallel. *Oncotarget* **2015**, *6*, 10445–10459.

(S9) Shimizu, H.; Watanabe, H.; Mizuno, M.; Kataoka, T.; Hori, M. Synthesis and Properties of Novel Medium-Sized Heterocyclic Compounds Containing Two Sulfur Atoms in the Ring and Synthetic Approaches to Conjugated Cyclic Disulfonium Ylides. *Heterocycles* **2001**, *54*, 139.

(S10) Hall, M.; Stueckler, C.; Hauer, B.; Stuermer, R.; Friedrich, T.; Breuer, M.; Kroutil, W.; Faber, K. Asymmetric Bioreduction of Activated C=C Bonds Using Zymomonas Mobilis NCR Enoate Reductase and Old Yellow Enzymes OYE 1–3 from Yeasts. *Eur. J. Org. Chem.* **2008**, *2008*, 1511–1516.

(S11) Casas, F.; Trincado, M.; Rodriguez-Lugo, R.; Baneerje, D.; Grützmacher, H. A Diaminopropane Diolefin Ru(0) Complex Catalyzes Hydrogenation and Dehydrogenation Reactions. *ChemCatChem* **2019**, *11*, 5241–5251.

(S12) Johnson, M. R.; Thelin, W. R. *PCT Int. Appl.*, **2016**, WO2016176423 A1.

(S13) Singh, P. K.; Field, L.; Sweetman, B. J. Organic Disulfides and Related Substances. 48. Cyclic Di- and Trisulfides Based on 1,4-Dithiols. *Phosphorus Sulfur Relat. Elem.* **1988**, *39*, 61–71.

(S14) Fuchs, M.; Fürstner, A. Trans-Hydrogenation: Application to a Concise and Scalable Synthesis of Brefeldin A. *Angew. Chem. Int. Ed.* **2015**, *54*, 3978–3982.

(S15) Mier-Vinué, J. de; Lorenzo, J.; Montaña, Á. M.; Moreno, V.; Avilés, F. X. Synthesis, DNA Interaction and Cytotoxicity Studies of *Cis*-{[1,2-Bis(Aminomethyl)Cyclo-hexane]Dihalo}platinum(II) Complexes. *J. Inorg. Biochem.* **2008**, *102*, 973–987.

(S16) Nagasawa, S.; Sasano, Y.; Iwabuchi, Y. Synthesis of 1,3-Cycloalkadienes from Cycloalkenes: Unprecedented Reactivity of Oxoammonium Salts. *Angew. Chem. Int. Ed.* **2016**, *55*, 13189–13194.

(S17) Samuel, C. J. Deazetation of a Bicyclic Azo Compound: Resolution of a Stereochemical Ambiguity and Conformational Analysis of a Biradical. *J. Chem. Soc. Perkin 1* **1989**, 1259–1265.

(S18) Houk, J.; Whitesides, G. M. Structure-Reactivity Relations for Thiol-Disulfide Interchange. *J. Am. Chem. Soc.* **1987**, *109*, 6825–6836.

(S19) Jones, G. B.; Wright, J. M.; Plourde, G. W.; Hynd, G.; Huber, R. S.; Mathews, J. E. A Direct and Stereocontrolled Route to Conjugated Enediynes. *J. Am. Chem. Soc.* **2000**, *122*, 1937–1944.

(S20) Wang, Y.; Hu, X.; Morales-Rivera, C. A.; Li, G.-X.; Huang, X.; He, G.; Liu, P.; Chen, G. Epimerization of Tertiary Carbon Centers via Reversible Radical Cleavage of Unactivated C(sp^3)–H Bonds. *J. Am. Chem. Soc.* **2018**, *140*, 9678–9684.

(S21) Candelon, N.; Hădade, N. D.; Matache, M.; Canet, J.-L.; Cisnetti, F.; Funeriu, D. P.; Nauton, L.; Gautier, A. Luminogenic “Clickable” Lanthanide Complexes for Protein Labeling. *Chem. Commun.* **2013**, *49*, 9206-9208.

(S22) Steinebach, C.; Sosič, I.; Lindner, S.; Bricelj, A.; Kohl, F.; Ng, Y. L. D.; Monschke, M.; Wagner, K. G.; Krönke, J.; Gütschow, M. A MedChem Toolbox for Cereblon-Directed PROTACs. *MedChemComm* **2019**, *10*, 1037–1041.

(S23) Kapras, V.; Slattery, W. T.; Menard, F. A CycloRGDf(Me-V) Analog as Chemical Probe to Study Integrins Function in Living Cells. *ChemRxiv*, **2020**, https://doi.org/10.26434/chemrxiv.11556144.v1.

(S24) Srivastava, P. K.; Field, L. Organic Disulfides and Related Substances. 45. Synthesis and Properties of Some Disulfide Sulfinate Salts Containing No Nitrogen. *J. Chem. Eng. Data* **1986**, *31*, 252–254.

(S25) Macke, J. D.; Field, L. Sulfinic Acids and Related Compounds 20. Synthesis and Properties of Some α, ω-Alkylenebis(dithioalkane-sulfinates). *Phosphorous Sulfur Relat. Elem.* **1988**, *37*, 27–33.

(S26) Jin, W. B.; Xu, C.; Cheng, Q.; Qi, X. L.; Gao, W.; Zheng, Z.; Chan, E. W. C.; Leung, Y.-C.; Chan, T. H.; Wong, K.-Y.; Chen, S.; Chan, K.-F. Investigation of Synergistic Antimicrobial Effects of the Drug Combinations of Meropenem and 1,2-Benzisoselenazol-3(2*H*)-One Derivatives on Carbapenem-Resistant Enterobacteriaceae Producing NDM-1. *Eur. J. Med. Chem.* **2018**, *155*, 285–302.

(S27) Cheng, Y.; Zong, L.; López - Andarias, J.; Bartolami, E.; Okamoto, Y.; Ward, T. R.; Sakai, N.; Matile, S. Cell-Penetrating Dynamic-Covalent Benzopolysulfane Networks. *Angew. Chem. Int. Ed.* **2019**, *58*, 9522–9526.

(S28) Macpherson, L. J.; Dubin, A. E.; Evans, M. J.; Marr, F.; Schultz, P. G.; Cravatt, B. F.; Patapoutian, A. Noxious Compounds Activate TRPA1 Ion Channels through Covalent Modification of Cysteines. *Nature* **2007**, *445*, 541–545.

(S29) Frei, R.; Wodrich, M. D.; Hari, D. P.; Borin, P.-A.; Chauvier, C.; Waser, J. Fast and Highly Chemoselective Alkynylation of Thiols with Hypervalent Iodine Reagents Enabled through a Low Energy Barrier Concerted Mechanism. *J. Am. Chem. Soc.* **2014**, *136*, 16563–16573.

(S30) Calandra, N. A.; Cheng, Y. L.; Kocak, K. A.; Miller, J. S. Total Synthesis of Spiruchostatin A via Chemoselective Macrocyclization Using an Accessible Enantiomerically Pure Latent Thioester. *Org. Lett.* **2009**, *11*, 1971–1974.

(S31) Gasparini, G.; Sargsyan, G.; Bang, E.-K.; Sakai, N.; Matile, S. Ring Tension Applied to Thiol-Mediated Cellular Uptake. *Angew. Chem. Int. Ed.* **2015**, *54*, 7328–7331.

(S32) Zong, L.; Bartolami, E.; Abegg, D.; Adibekian, A.; Sakai, N.; Matile, S. Epidithiodiketopiperazines: Strain-Promoted Thiol-Mediated Cellular Uptake at the Highest Tension. *ACS Cent. Sci.* **2017**, *3*, 449–453.

(S33) Singh, P. K.; Field, L.; Sweetman, B. J. Organic Disulfides and Related Substances. 49. Preparation of Cyclic Thiosulfinates and Reactions with Thiols. *J. Org. Chem.* **1988**, *53*, 2608–2612.

(S34) Cavero Tomas, M. Ph.D. Dissertation, **1999**, University College London.

(S35) Fendler, J. H.; Hinze, W. L. Reactivity Control in Micelles and Surfactant Vesicles. Kinetics and Mechanism of Base-Catalyzed Hydrolysis of 5,5'-Dithiobis(2-Nitrobenzoic Acid) in Water, Hexadecyltrimethylammonium Bromide Micelles, and Dioctadecyldimethylammonium Chloride Surfactant Vesicles. *J. Am. Chem. Soc.* **1981**, *103*, 5439–5447.

(S36) Laurent, Q.; Martinent, R.; Moreau, D.; Winssinger, N.; Sakai, N.; Matile, S. Oligonucleotide Phosphorothioates Enter Cells by Thiol-Mediated Uptake. *Angew. Chem. Int. Ed.* **2021**, *60*, 19102–19106.

(S37) Giry-Laterrière, M.; Verhoeyen, E.; Salmon, P. *Lentiviral Vectors*. In *Viral Vectors for Gene Therapy: Methods and Protocols*; Merten, O.-W., Al-Rubeai, M., Eds.; *Methods in Molecular Biology*; Humana Press: Totowa, NJ, **2011**; 183–209.

(S38) Gaussian 09, Revision D.01, Frisch, M. J.; Trucks, G. W.; Schlegel, H. B.; Scuseria, G. E.; Robb, M. A.; Cheeseman, J. R.; Scalmani, G.; Barone, V.; Mennucci, B.; Petersson, G. A.; Nakatsuji, H.; Caricato, M.; Li, X.; Hratchian, H. P.; Izmaylov, A. F.; Bloino, J.; Zheng, G.; Sonnenberg, J. L.; Hada, M.; Ehara, M.; Toyota, K.; Fukuda, R.; Hasegawa, J.; Ishida, M.; Nakajima, T.; Honda, Y.; Kitao, O.; Nakai, H.; Vreven, T.; Montgomery,

J. A., Jr.; Peralta, J. E.; Ogliaro, F.; Bearpark, M.; Heyd, J. J.; Brothers, E.; Kudin, K. N.; Staroverov, V. N.; Kobayashi, R.; Normand, J.; Raghavachari, K.; Rendell, A.; Burant, J. C.; Iyengar, S. S.; Tomasi, J.; Cossi, M.; Rega, N.; Millam, J. M.; Klene, M.; Knox, J. E.; Cross, J. B.; Bakken, V.; Adamo, C.; Jaramillo, J.; Gomperts, R.; Stratmann, R. E.; Yazyev, O.; Austin, A. J.; Cammi, R.; Pomelli, C.; Ochterski, J. W.; Martin, R. L.; Morokuma, K.; Zakrzewski, V. G.; Voth, G. A.; Salvador, P.; Dannenberg, J. J.; Dapprich, S.; Daniels, A. D.; Farkas, Ö.; Foresman, J. B.; Ortiz, J. V.; Cioslowski, J.; Fox, D. J. Gaussian, Inc., Wallingford CT, **2009**.

(S39) Marenich, A. V.; Cramer, C. J.; Truhlar, D. G. Universal solvation model based on solute electron density and on a continuum model of the solvent defined by the bulk dielectric constant and atomic surface tensions. *J. Phys. Chem. B* **2009**, *113*, 6378-6396.

The original data can be found at: https://doi.org/10.5281/zenodo.6006520

8. Computational Data

1,2-dithiane with F$^-$ (Figure 3G)

Zero-point correction = 0.117157 (Hartree/Particle)

Thermal correction to Energy = 0.125320

Thermal correction to Enthalpy = 0.126264

Thermal correction to Gibbs Free Energy = 0.084085

Sum of electronic and zero-point Energies = -1053.342479

Sum of electronic and thermal Energies = -1053.334317

Sum of electronic and thermal Enthalpies = -1053.333373

Sum of electronic and thermal Free Energies = -1053.375551

C	-2.659466	-0.249946	-0.267433
C	-2.046421	-1.627865	0.004968
C	-0.787139	-1.941172	-0.800096

S	-1.103492	-2.212259	-2.577196
S	-1.637350	-0.036433	-2.861288
C	-3.060199	-0.019520	-1.723581
H	-1.799853	-1.689453	1.073345
H	-1.942112	0.527702	0.020864
F	-0.659441	-4.199400	-2.141037
H	-0.354154	-2.887388	-0.483144
H	-0.056298	-1.134779	-0.696146
H	-3.555127	0.950642	-1.827095
H	-3.760095	-0.797140	-2.045529
H	-3.545385	-0.125316	0.369957
H	-2.785497	-2.410194	-0.198741

1,2-dithiane-1-*ax*-oxide with F$^-$ (Figure 3F)

Zero-point correction = 0.121364 (Hartree/Particle)

Thermal correction to Energy = 0.130400

Thermal correction to Enthalpy = 0.131345

Thermal correction to Gibbs Free Energy = 0.087278

Sum of electronic and zero-point Energies = -1128.521913

Sum of electronic and thermal Energies = -1128.512877

Sum of electronic and thermal Enthalpies = -1128.511933

Sum of electronic and thermal Free Energies = -1128.556000

C	-0.489158	-0.629962	-0.325033
C	-1.051958	0.701131	0.161401
C	-2.200623	1.251360	-0.689527
C	-1.809968	1.491977	-2.145220

S	-1.616435	-0.048008	-3.100043
S	0.313132	-0.466714	-1.967748
H	-1.385265	0.580791	1.198749
H	-3.060009	0.572954	-0.664427
F	-3.433673	0.373743	-3.893482
O	1.203214	0.782764	-1.821520
H	-2.590714	2.020475	-2.685847
H	-0.867246	2.044874	-2.199070
H	0.278047	-0.995158	0.363555
H	-1.265377	-1.389973	-0.440788
H	-0.228603	1.423569	0.163254
H	-2.525363	2.204260	-0.252435

1,2-dithiane-1-*eq*-oxide with F$^-$ (Figure S59a)

Zero-point correction = 0.121049 (Hartree/Particle)

Thermal correction to Energy = 0.130170

Thermal correction to Enthalpy = 0.131115

Thermal correction to Gibbs Free Energy = 0.086774

Sum of electronic and zero-point Energies = -1128.517705

Sum of electronic and thermal Energies = -1128.508583

Sum of electronic and thermal Enthalpies = -1128.507639

Sum of electronic and thermal Free Energies = -1128.551979

C	-0.464385	-0.603802	-0.365122
C	-1.063740	0.698380	0.162499
C	-2.207561	1.253105	-0.690719
C	-1.809535	1.492520	-2.144355

S	-1.618109	-0.047379	-3.105714
S	0.328634	-0.401814	-1.998840
H	-1.418004	0.529879	1.185547
H	-3.060286	0.568026	-0.678692
O	0.826716	-1.802030	-2.363606
H	-2.590215	2.022099	-2.683718
H	-0.880119	2.072007	-2.190561
H	0.316786	-0.982433	0.299388
H	-1.224480	-1.377409	-0.503320
H	-0.269319	1.452677	0.228476
H	-2.542495	2.200956	-0.250582
F	-3.461873	0.413492	-3.854482

1,2-dithiane-1,1-dioxide with F^- (Figure 3E)

Zero-point correction = 0.126336 (Hartree/Particle)

Thermal correction to Energy = 0.136008

Thermal correction to Enthalpy = 0.136953

Thermal correction to Gibbs Free Energy = 0.091446

Sum of electronic and zero-point Energies = -1203.732357

Sum of electronic and thermal Energies = -1203.722684

Sum of electronic and thermal Enthalpies = -1203.721740

Sum of electronic and thermal Free Energies = -1203.767246

C	-2.613946	0.061818	-0.155992
C	-2.047843	-1.316364	0.205408
C	-0.788411	-1.689719	-0.568880
S	-1.119110	-1.769508	-2.353687

S	-1.647661	0.391793	-2.794941
C	-3.042481	0.195921	-1.617955
H	-1.824889	-1.347557	1.277390
O	0.174328	-2.123957	-2.979704
O	-2.225067	-2.756465	-2.478001
H	-1.880587	0.844082	0.066193
F	-2.163873	2.267933	-2.926552
H	-0.418351	-2.683953	-0.305128
H	0.009746	-0.954217	-0.450033
H	-3.609825	1.109040	-1.776598
H	-3.638253	-0.667209	-1.925089
H	-3.489755	0.249292	0.477095
H	-2.806017	-2.081671	0.008749

(4*S*,5*R*)-1,2-dithiane-4,5-diol with F$^-$ (**1F**, Figure S59c)

Zero-point correction = 0.127351 (Hartree/Particle)

Thermal correction to Energy = 0.137457

Thermal correction to Enthalpy = 0.138402

Thermal correction to Gibbs Free Energy = 0.092004

Sum of electronic and zero-point Energies = -1203.775626

Sum of electronic and thermal Energies = -1203.765520

Sum of electronic and thermal Enthalpies = -1203.764575

Sum of electronic and thermal Free Energies = -1203.810973

C	-2.618711	-0.270652	-0.281992
C	-2.016193	-1.627154	0.026943
S	-0.544008	-1.911968	-1.002916

S	0.805814	-0.332313	0.020142
C	-0.363046	0.984151	-0.452755
C	-1.759764	0.920902	0.157553
O	-1.684508	0.987198	1.566457
O	-3.882409	-0.191325	0.409115
H	-2.763492	-2.397028	-0.178458
H	-1.734983	-1.678639	1.082295
H	0.128735	1.892929	-0.108900
H	-0.462843	1.000072	-1.541190
H	-2.287785	1.823987	-0.209529
H	-2.785766	-0.200543	-1.363311
H	-4.299333	0.638245	0.157988
H	-2.489357	0.582618	1.906084
F	1.889520	1.103278	0.842853

(4*S*,5*R*)-1,2-dithiane-4,5-diol with Cl^- (**1Cl**, Figure 3I)

Zero-point correction = 0.126843 (Hartree/Particle)

Thermal correction to Energy = 0.137570

Thermal correction to Enthalpy = 0.138514

Thermal correction to Gibbs Free Energy = 0.088030

Sum of electronic and zero-point Energies = -1564.178051

Sum of electronic and thermal Energies = -1564.167324

Sum of electronic and thermal Enthalpies = -1564.166380

Sum of electronic and thermal Free Energies = -1564.216864

C	-2.660993	-0.265574	-0.313228
C	-2.158433	-1.666684	-0.009631

S	-0.628459	-2.011570	-0.931188
S	0.634546	-0.621740	0.008929
C	-0.345557	0.862211	-0.348793
C	-1.754409	0.847005	0.228687
O	-1.698938	0.779422	1.636190
O	-3.949857	-0.154487	0.308475
H	-2.903818	-2.397231	-0.330927
H	-1.969360	-1.781711	1.058862
H	0.255215	1.667417	0.088168
H	-0.390607	1.002793	-1.431609
H	-2.218650	1.803351	-0.075959
H	-2.758207	-0.149477	-1.399527
H	-4.331902	0.688670	0.047215
H	-2.585843	0.565843	1.943515
Cl	2.597144	2.155521	0.551201

(4*S*,5*R*)-4,5-dihydroxy-1,2-dithiane-1,1-dioxide with F^- (**3F**, Figure S59b)

Zero-point correction = 0.135990 (Hartree/Particle)

Thermal correction to Energy = 0.147774

Thermal correction to Enthalpy = 0.148718

Thermal correction to Gibbs Free Energy = 0.098618

Sum of electronic and zero-point Energies = -1354.163480

Sum of electronic and thermal Energies = -1354.151696

Sum of electronic and thermal Enthalpies = -1354.150752

Sum of electronic and thermal Free Energies = -1354.200852

C	-2.595182	0.002455	-0.108289

C	-2.006361	-1.351427	0.223678
S	-0.473935	-1.584146	-0.733444
S	0.899483	-0.003277	0.228287
C	-0.334359	1.239345	-0.308197
C	-1.722606	1.186654	0.326779
O	-0.021273	-2.956680	-0.414036
O	-0.896482	-1.322891	-2.139287
O	-1.625143	1.240524	1.732029
O	-3.857485	0.094581	0.574513
H	-2.681939	-2.156948	-0.070376
H	-1.735691	-1.437171	1.276776
H	0.143367	2.177540	-0.034533
H	-0.444476	1.184675	-1.394211
H	-2.244776	2.092558	-0.035484
H	-2.747456	0.054930	-1.191428
H	-4.339708	0.837682	0.201372
H	-2.479849	0.958348	2.075214
F	1.888115	1.402713	1.024743

(4*S*,5*R*)-4,5-dihydroxy-1,2-dithiane-1,1-dioxide with Cl$^-$ (3Cl, Figure 3H)

Zero-point correction = 0.136092 (Hartree/Particle)

Thermal correction to Energy = 0.148306

Thermal correction to Enthalpy = 0.149250

Thermal correction to Gibbs Free Energy = 0.096789

Sum of electronic and zero-point Energies = -1714.555460

Sum of electronic and thermal Energies = -1714.543247

Sum of electronic and thermal Enthalpies = -1714.542302

Sum of electronic and thermal Free Energies = -1714.594763

C	-2.637719	0.010754	-0.142756
C	-2.086529	-1.364346	0.188460
S	-0.552089	-1.649803	-0.712443
S	0.750514	-0.228107	0.129650
C	-0.350157	1.174239	-0.279157
C	-1.736659	1.147926	0.350705
O	-0.077080	-2.985378	-0.364557
O	-0.862994	-1.350455	-2.114236
O	-1.631595	1.105302	1.753132
O	-3.906316	0.096252	0.514974
H	-2.771319	-2.149780	-0.136445
H	-1.853963	-1.465730	1.248537
H	0.229854	2.017067	0.104522
H	-0.433978	1.246189	-1.365550
H	-2.221784	2.089958	0.038782
H	-2.763369	0.090514	-1.227714
H	-4.378536	0.851801	0.154529
H	-2.515949	0.937933	2.095487
Cl	2.523911	2.085128	0.998182

9. NMR Spectra

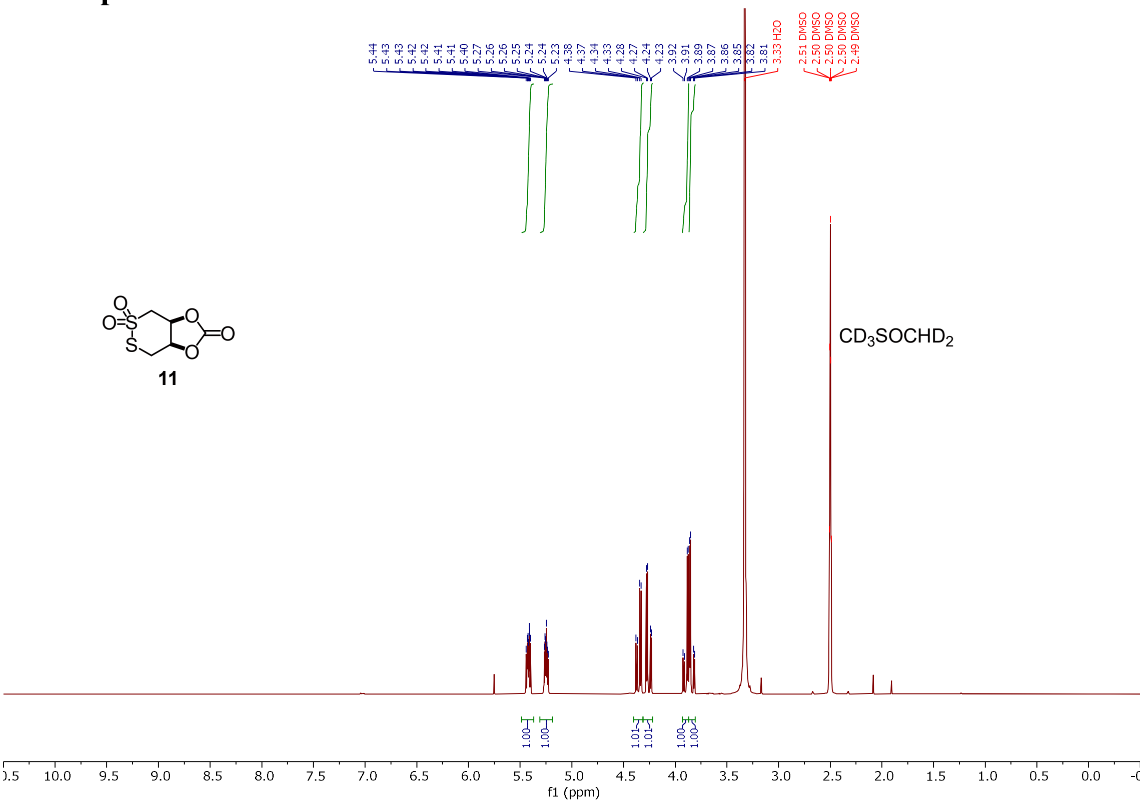

Figure S60. ^{1}H NMR (400 MHz, DMSO-d_6) spectrum of **11**.

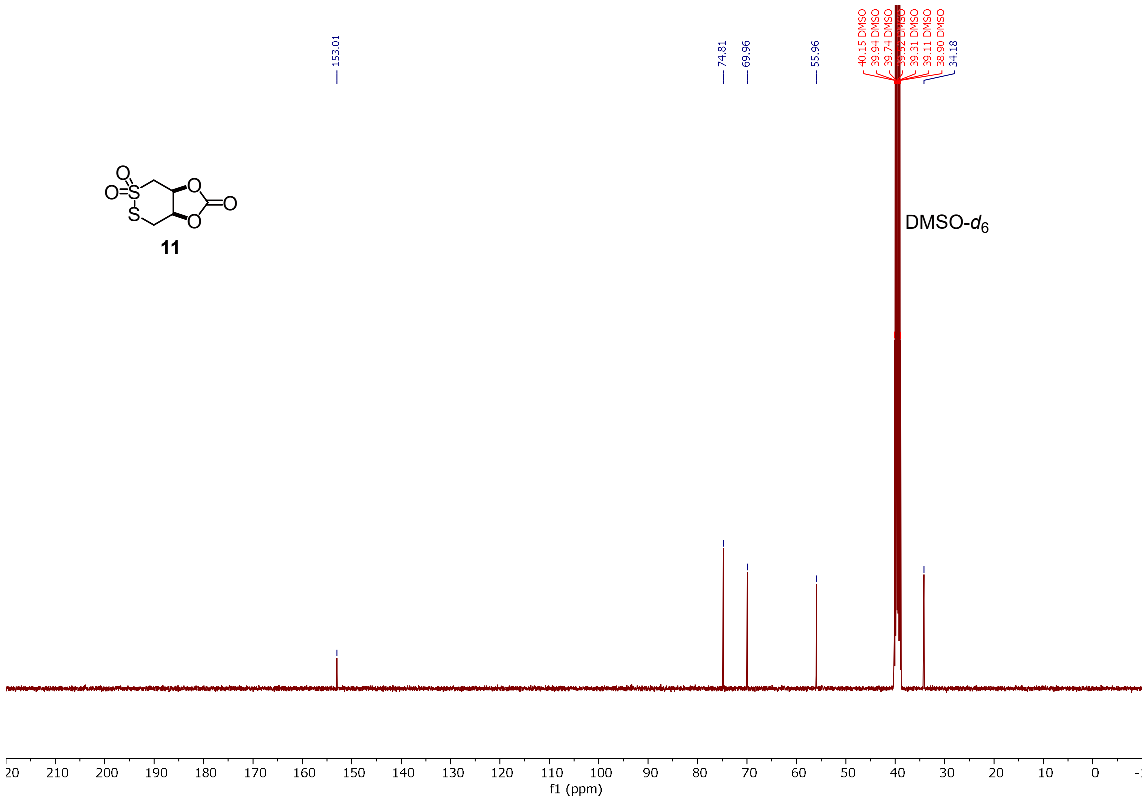

Figure S61. ^{13}C NMR (101 MHz, DMSO-d_6) spectrum of **11**.

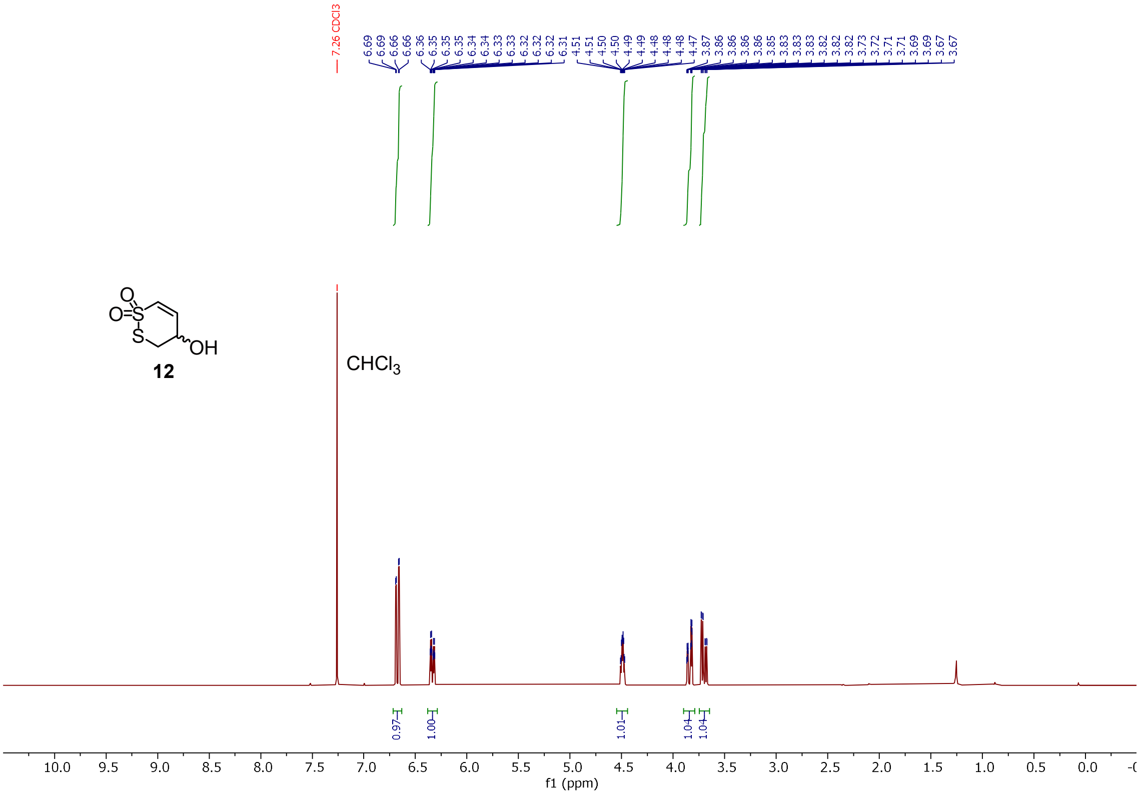

Figure S62. ^{1}H NMR (400 MHz, $CDCl_3$) spectrum of **12**.

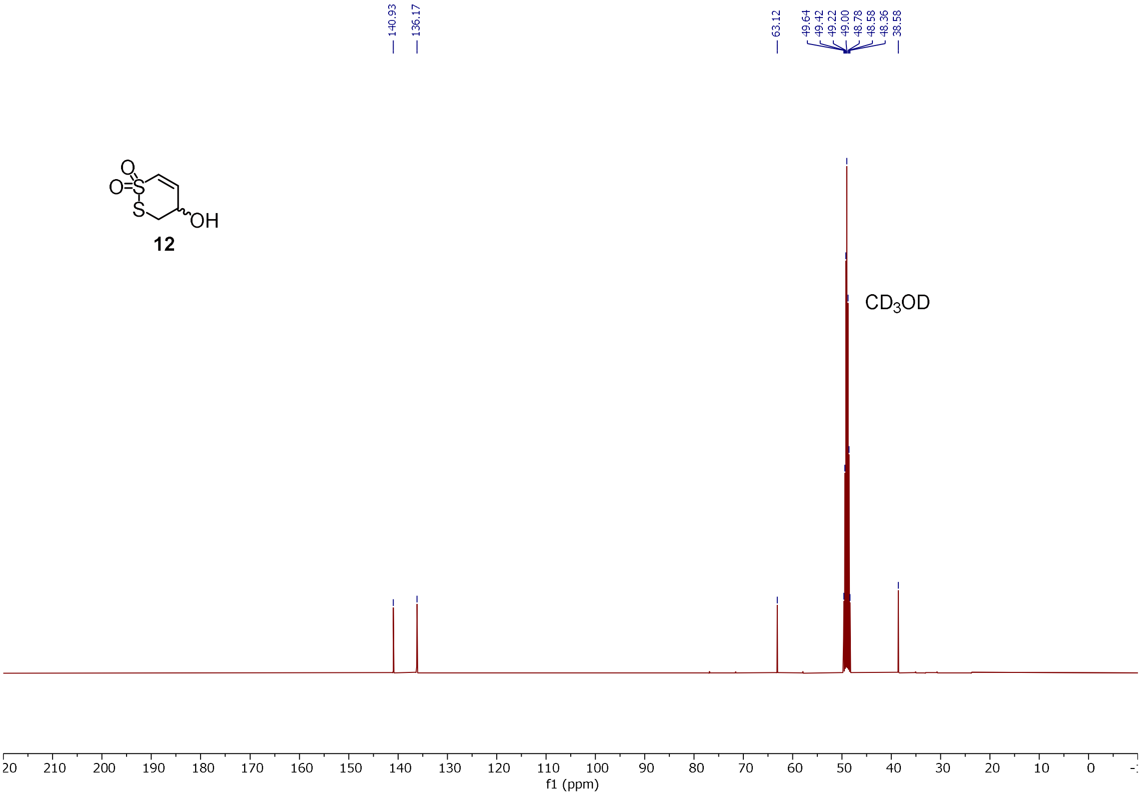

Figure S63. ^{13}C NMR (101 MHz, CD_3OD) spectrum of **12**.

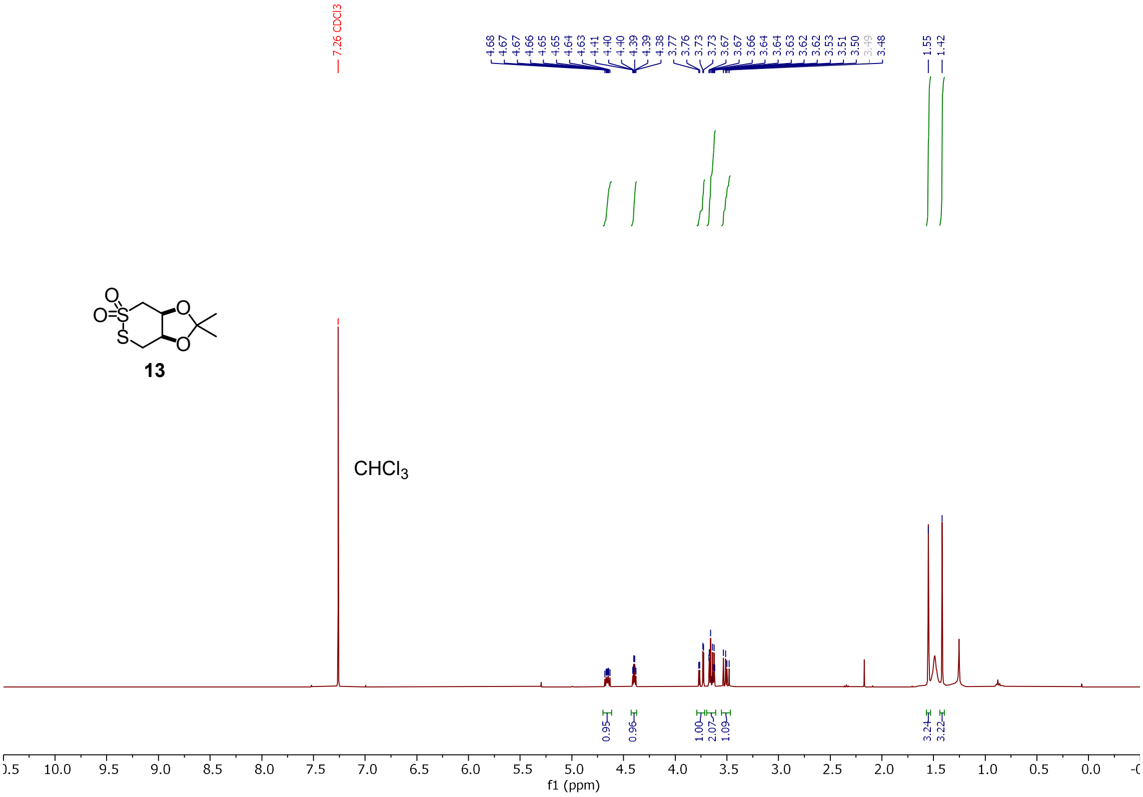

Figure S64. ^{1}H NMR (400 MHz, $CDCl_3$) spectrum of **13**.

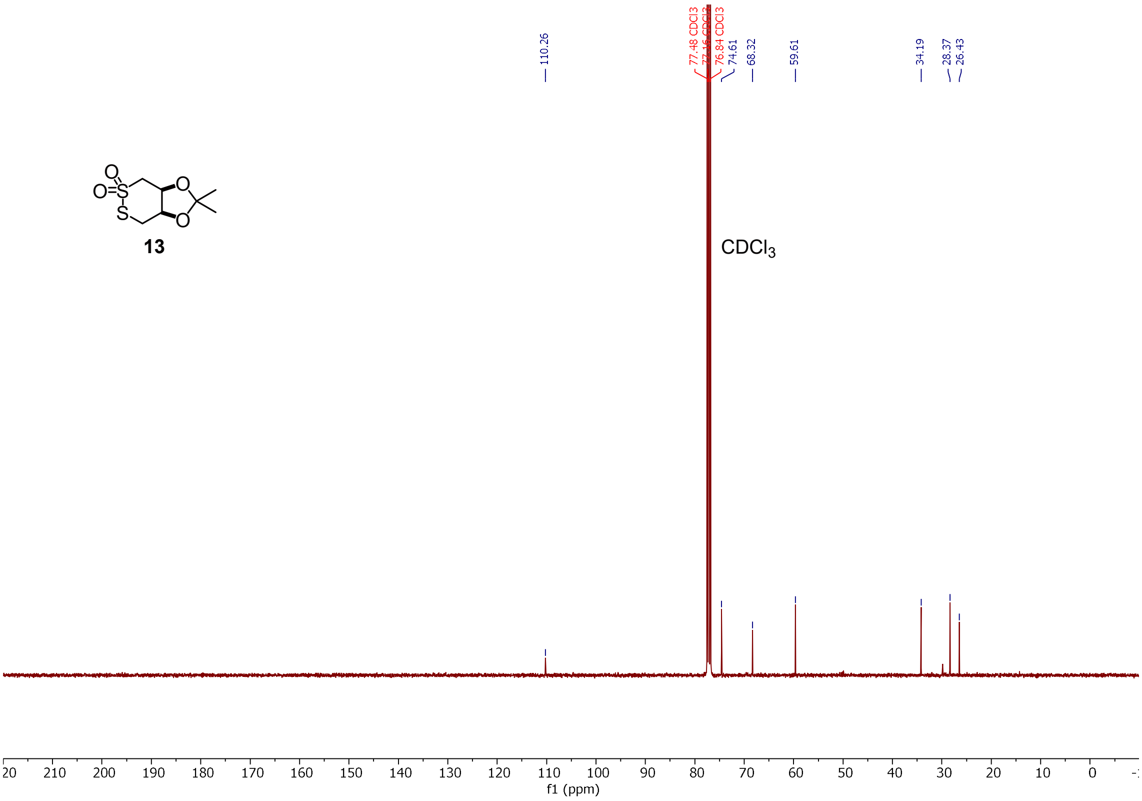

Figure S65. ^{13}C NMR (101 MHz, $CDCl_3$) spectrum of **13**.

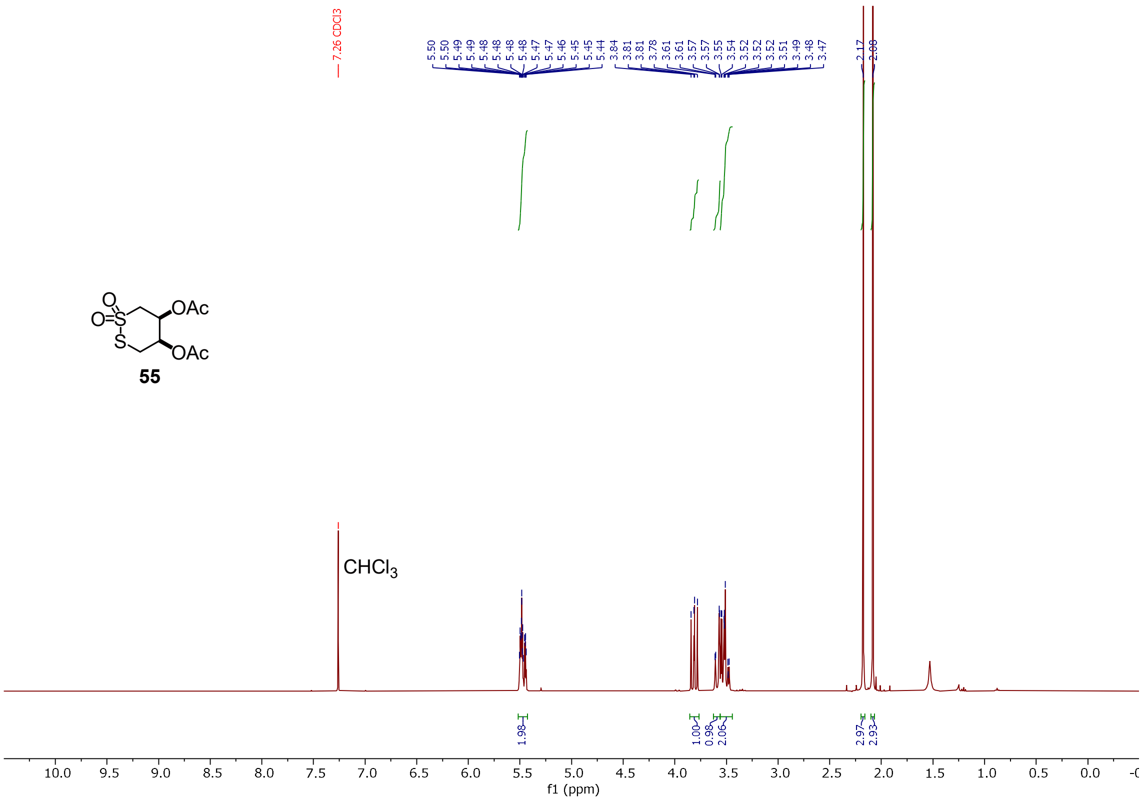

Figure S66. ^{1}H NMR (400 MHz, $CDCl_3$) spectrum of **55**.

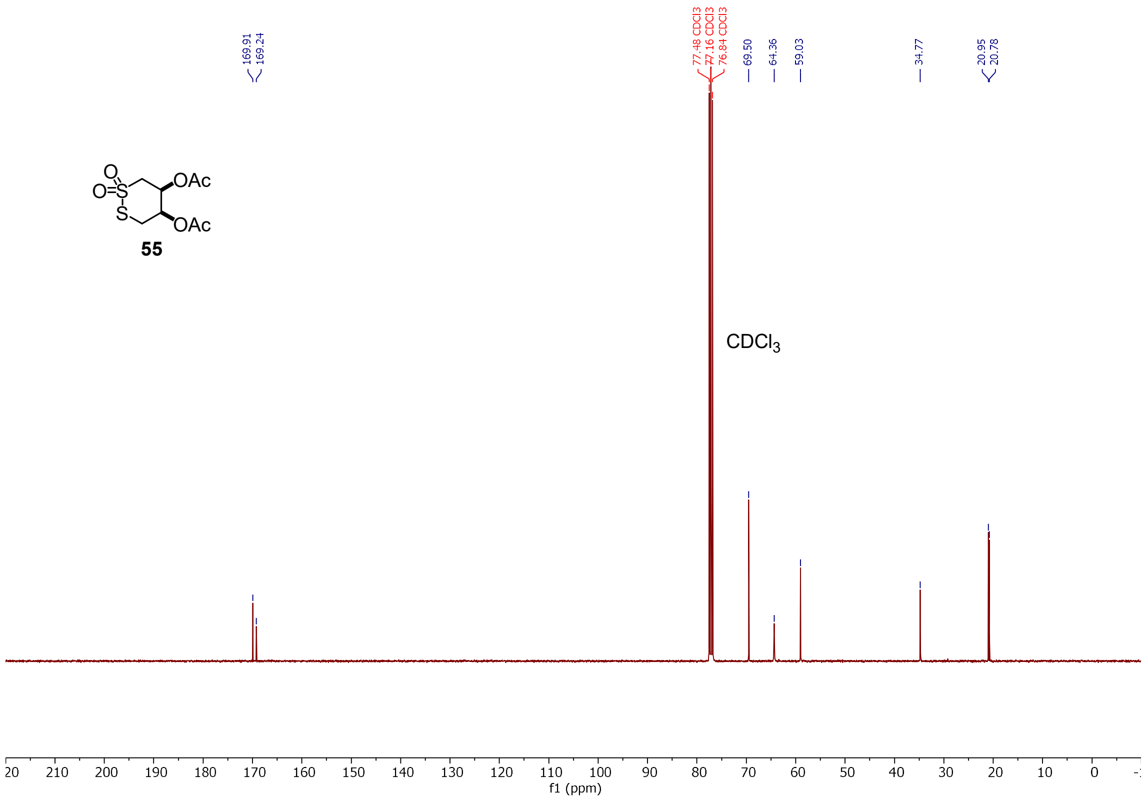

Figure S67. ^{13}C NMR (101 MHz, $CDCl_3$) spectrum of **55**.

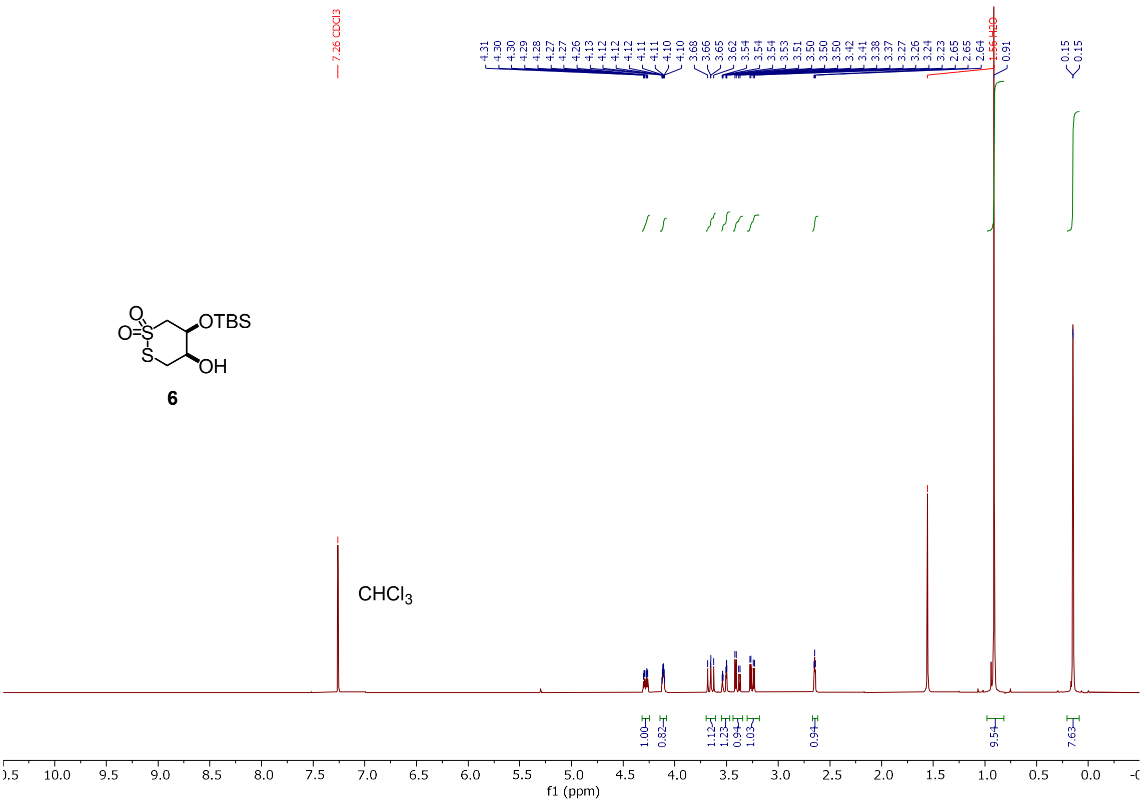

Figure S68. ^{1}H NMR (400 MHz, $CDCl_3$) spectrum of **6**.

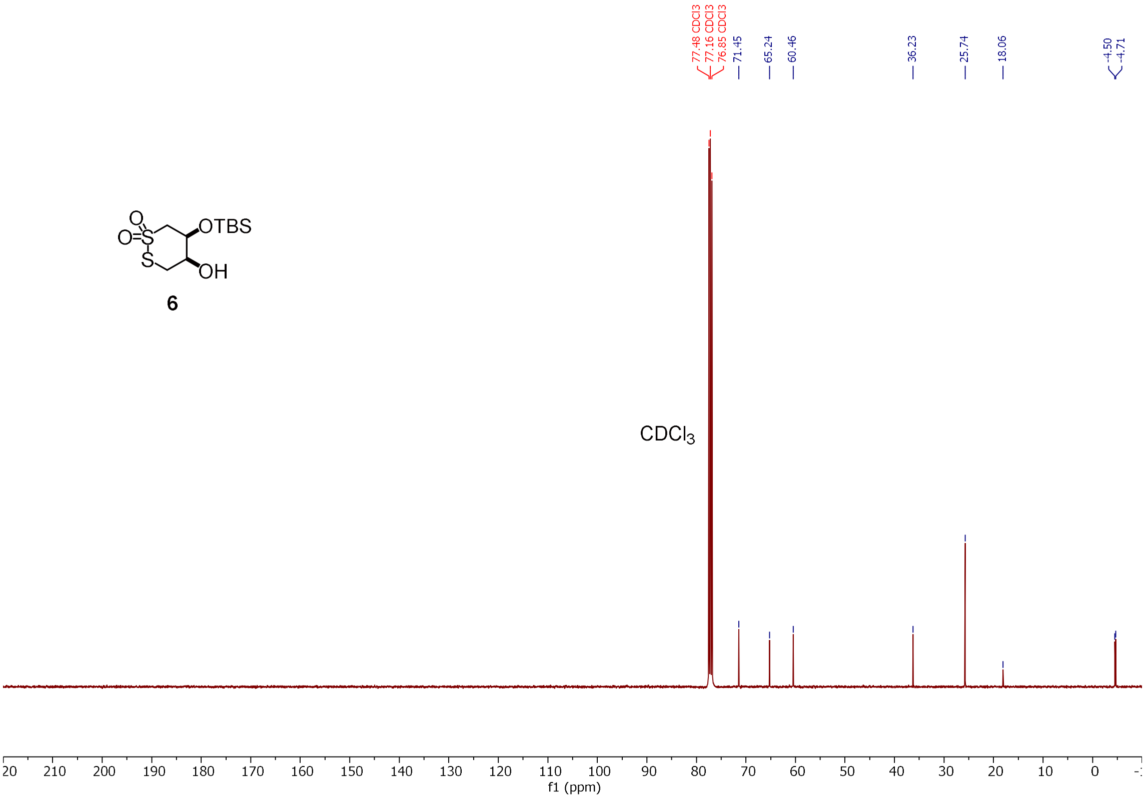

Figure S69. ^{13}C NMR (101 MHz, $CDCl_3$) spectrum of **6**.

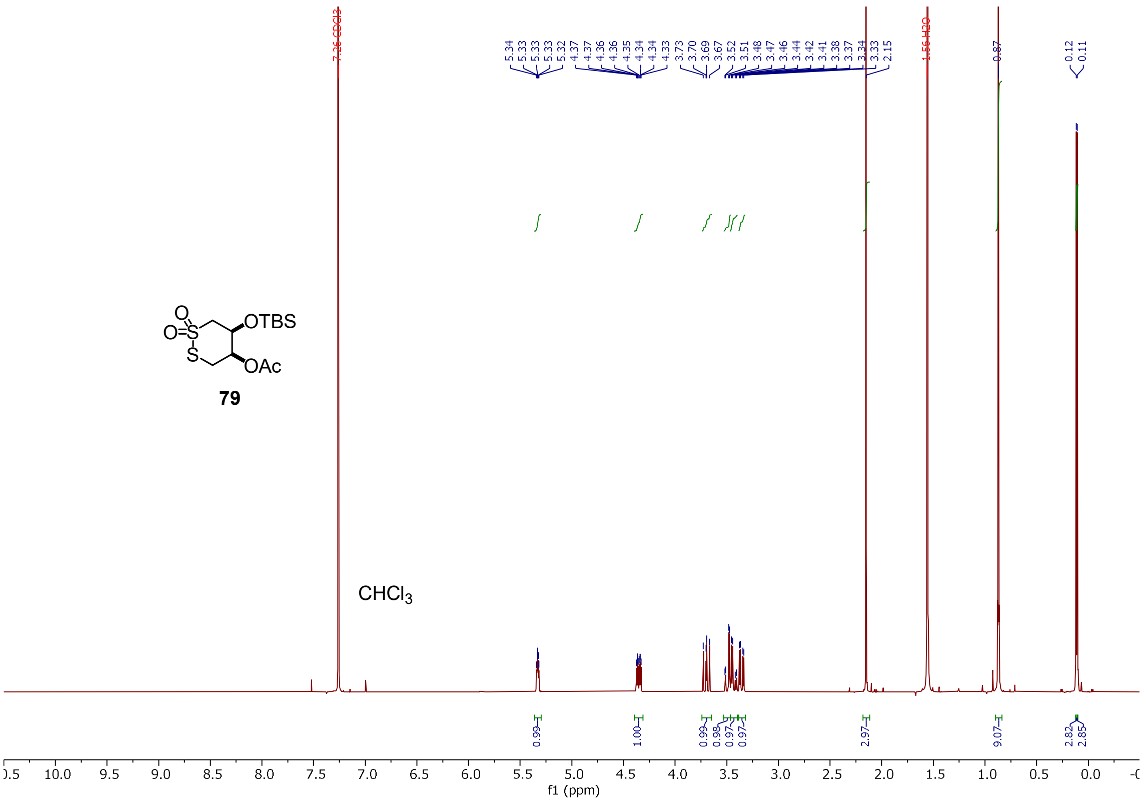

Figure S70. ^{1}H NMR (400 MHz, $CDCl_3$) spectrum of **79**.

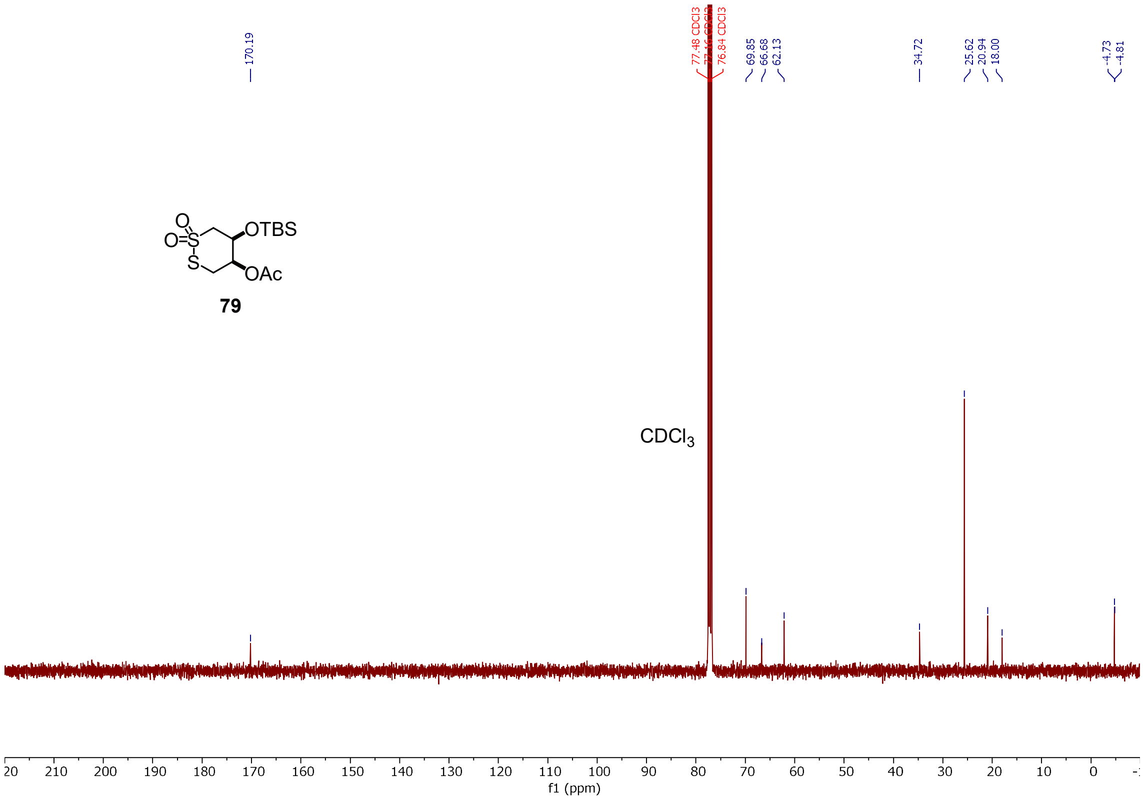

Figure S71. ^{13}C NMR (101 MHz, $CDCl_3$) spectrum of **79**.

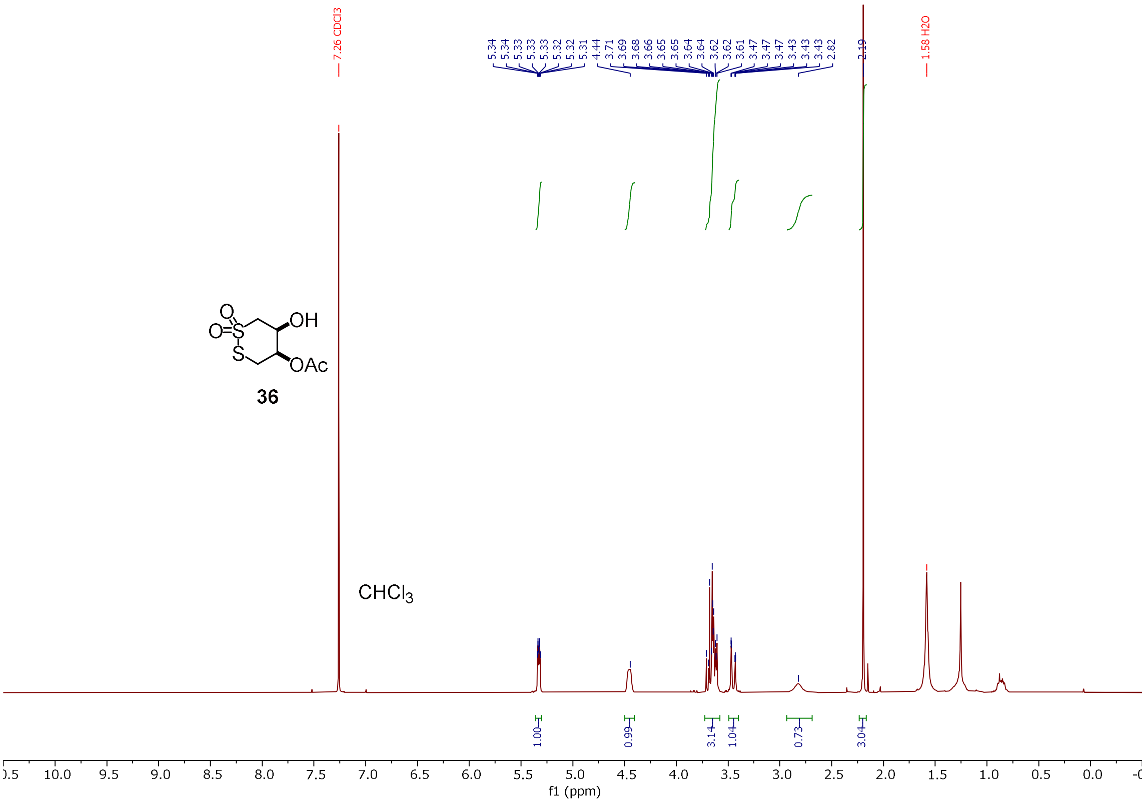

Figure S72. ^{1}H NMR (400 MHz, $CDCl_3$) spectrum of **36**.

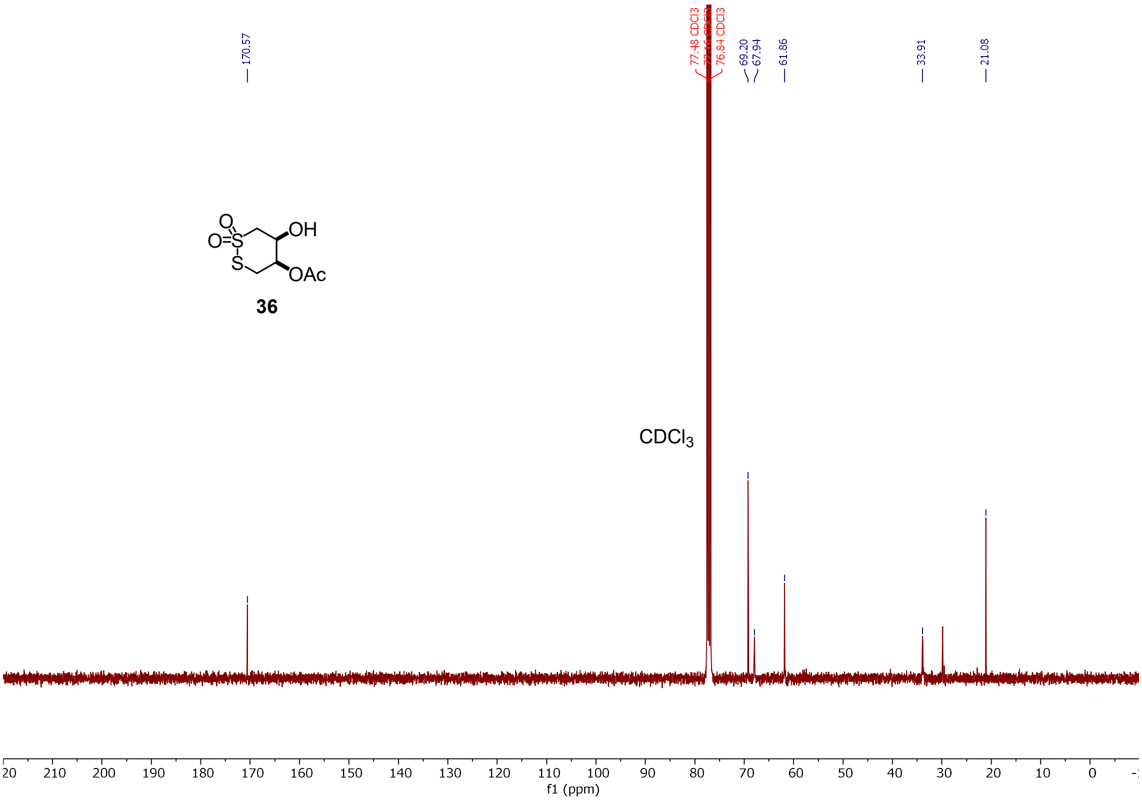

Figure S73. ^{13}C NMR (101 MHz, $CDCl_3$) spectrum of **36**.

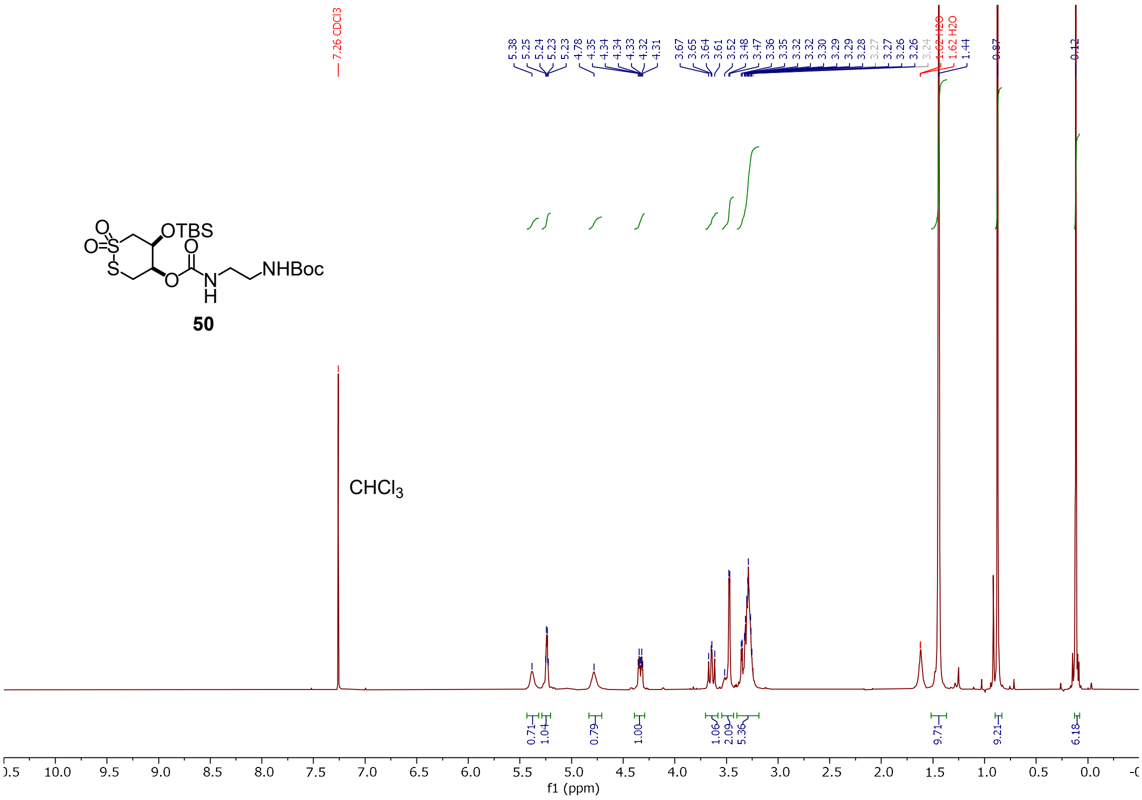

Figure S74. ^{1}H NMR (400 MHz, $CDCl_3$) spectrum of **50**.

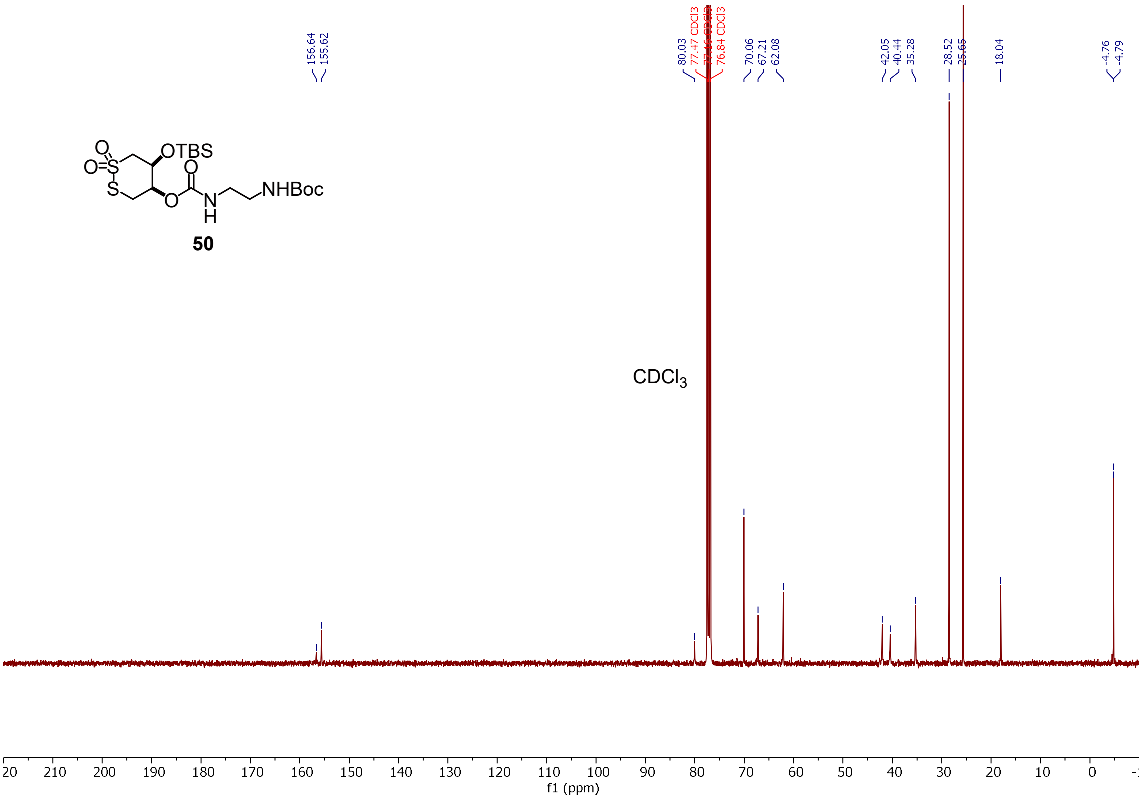

Figure S75. ^{13}C NMR (101 MHz, $CDCl_3$) spectrum of **50**.

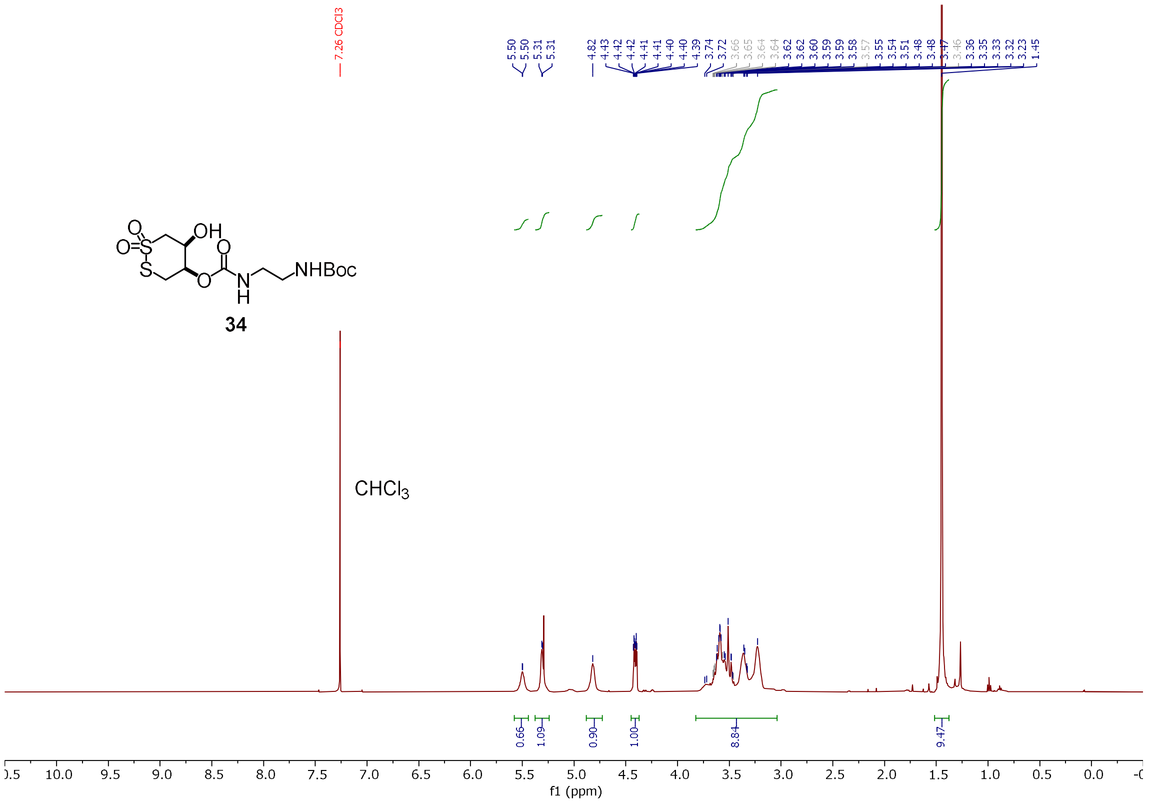

Figure S76. ^{1}H NMR (500 MHz, $CDCl_3$, 50 °C) spectrum of **34**.

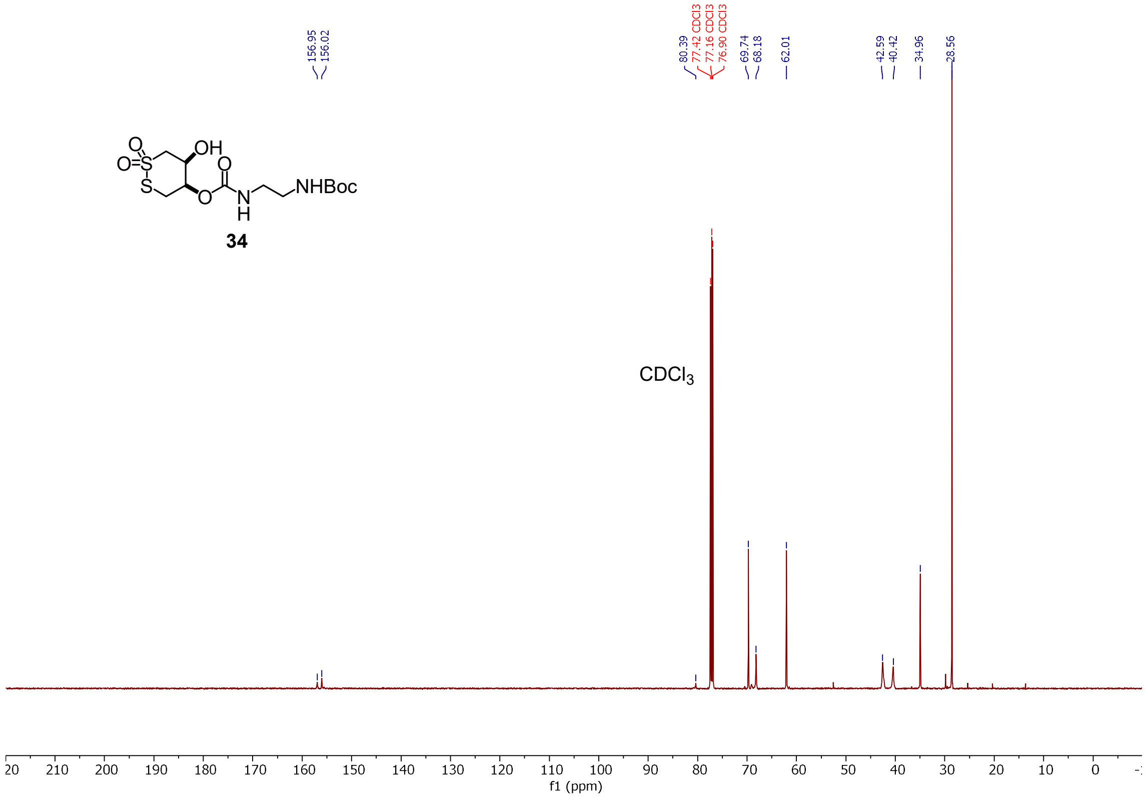

Figure S77. ^{13}C NMR (126 MHz, $CDCl_3$, 50 °C) spectrum of **34**.

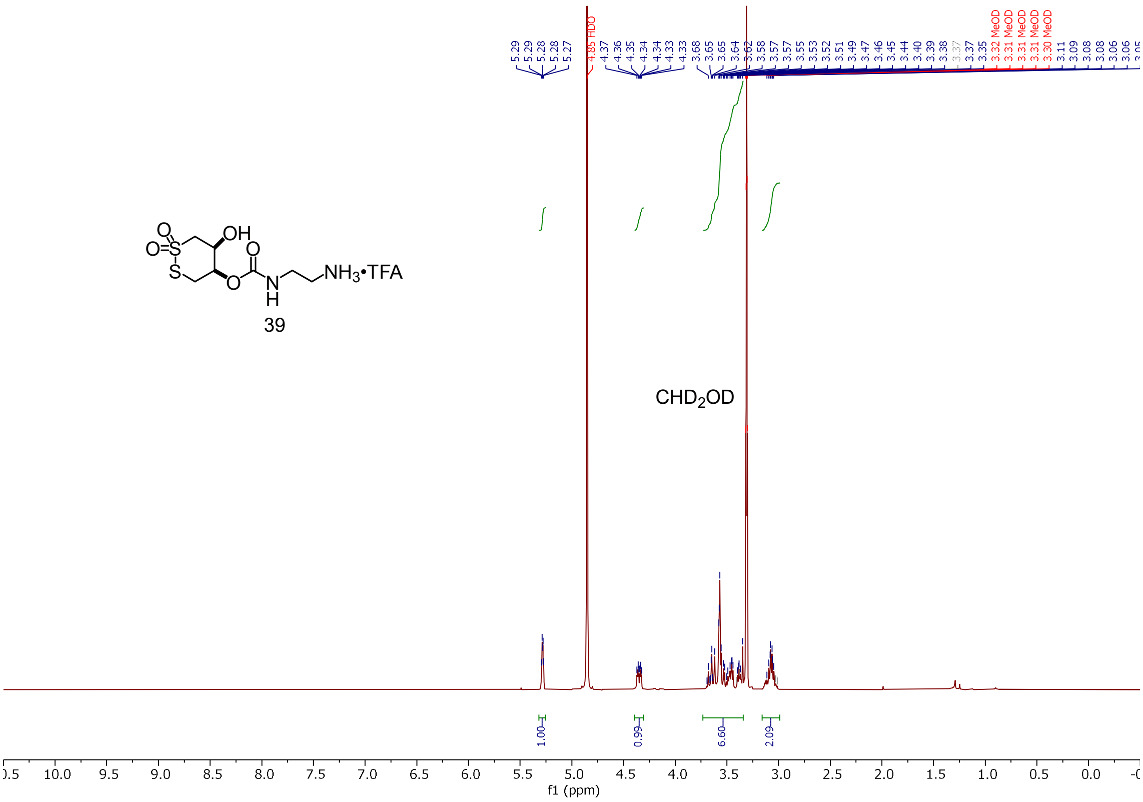

Figure S78. ^{1}H NMR (400 MHz, CD_3OD) spectrum of **39**.

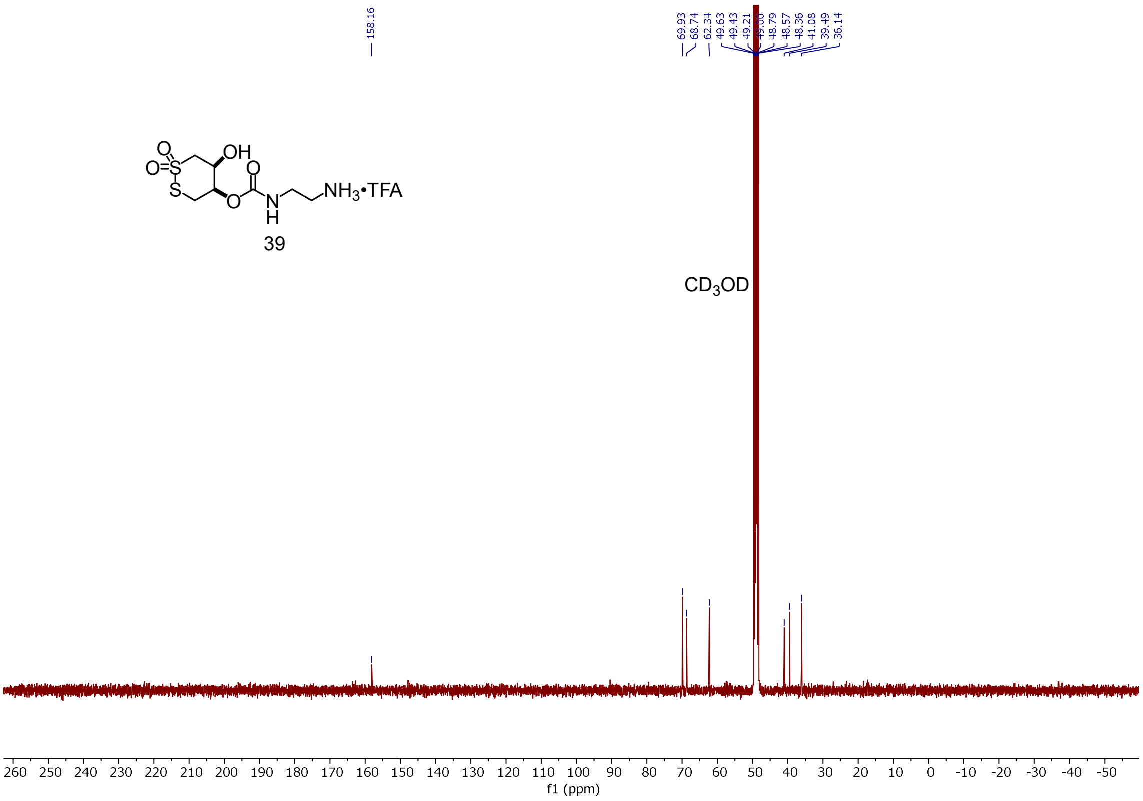

Figure S79. ^{13}C NMR (101 MHz, CD_3OD) spectrum of **39**.

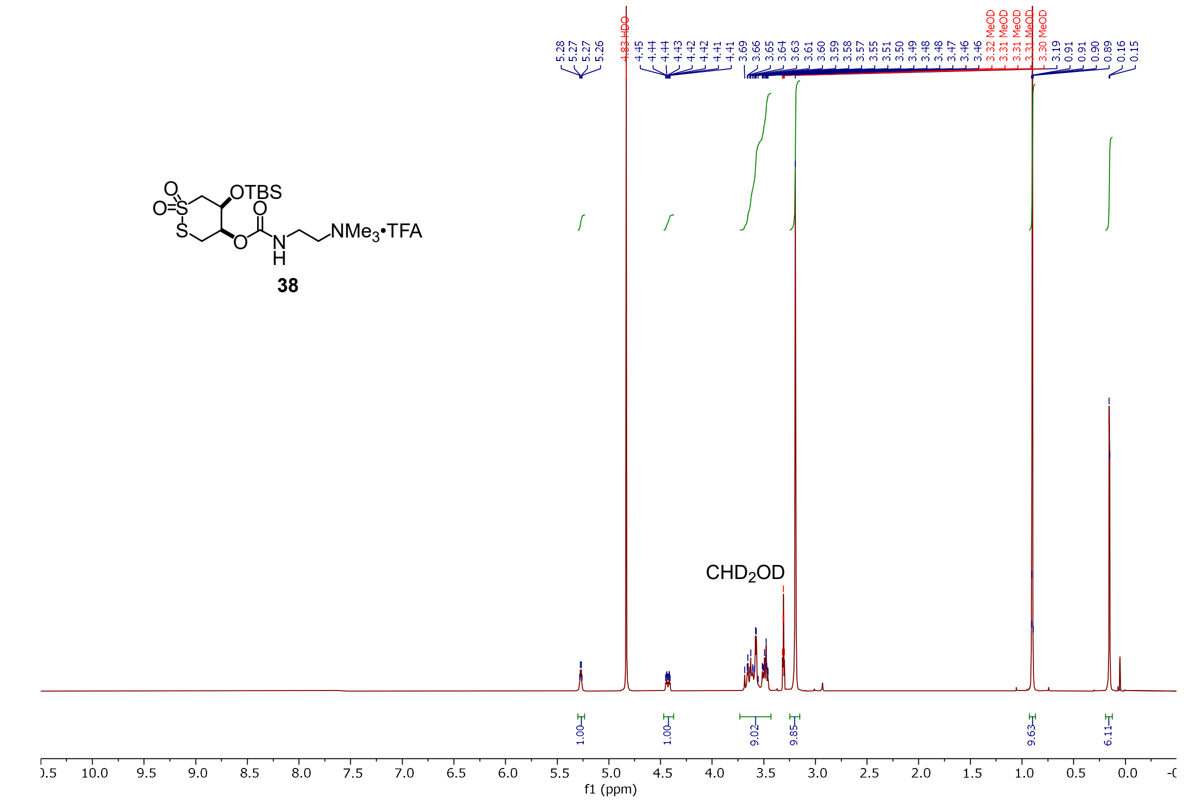

Figure S80. ^{1}H NMR (400 MHz, CD_3OD) spectrum of **38**.

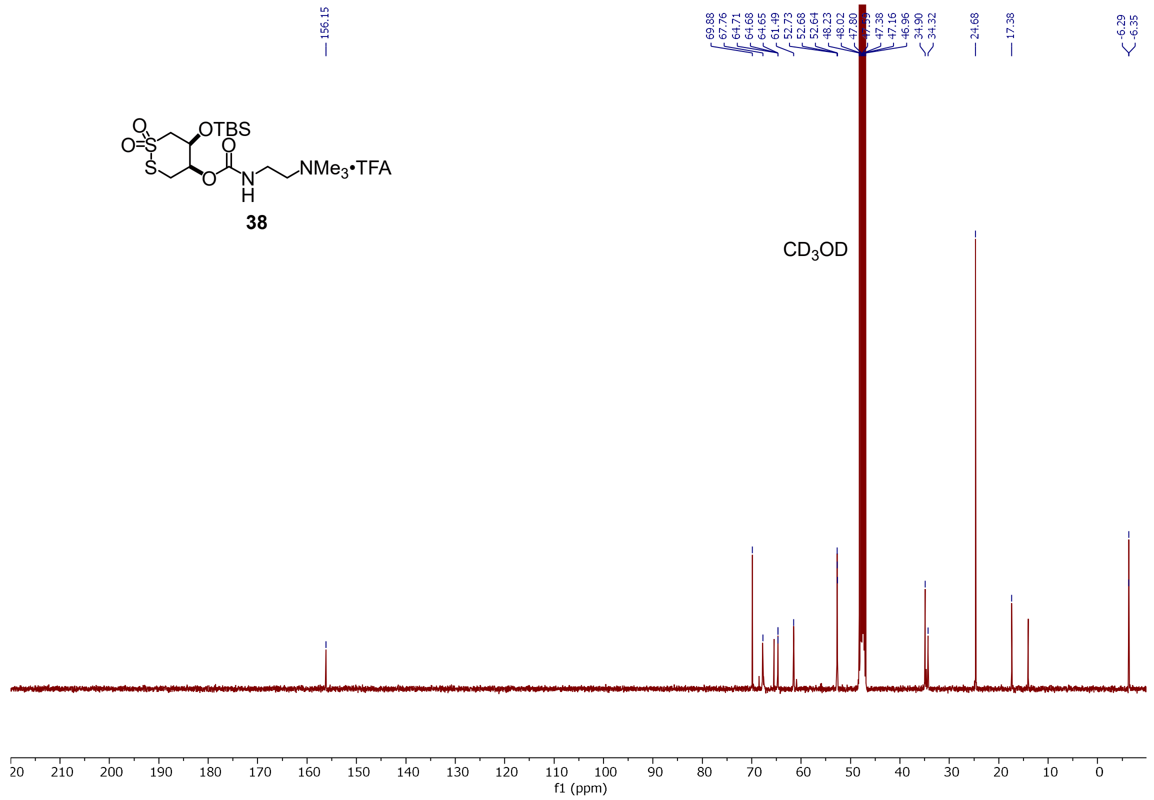

Figure S81. ^{13}C NMR (101 MHz, CD_3OD) spectrum of **38**.

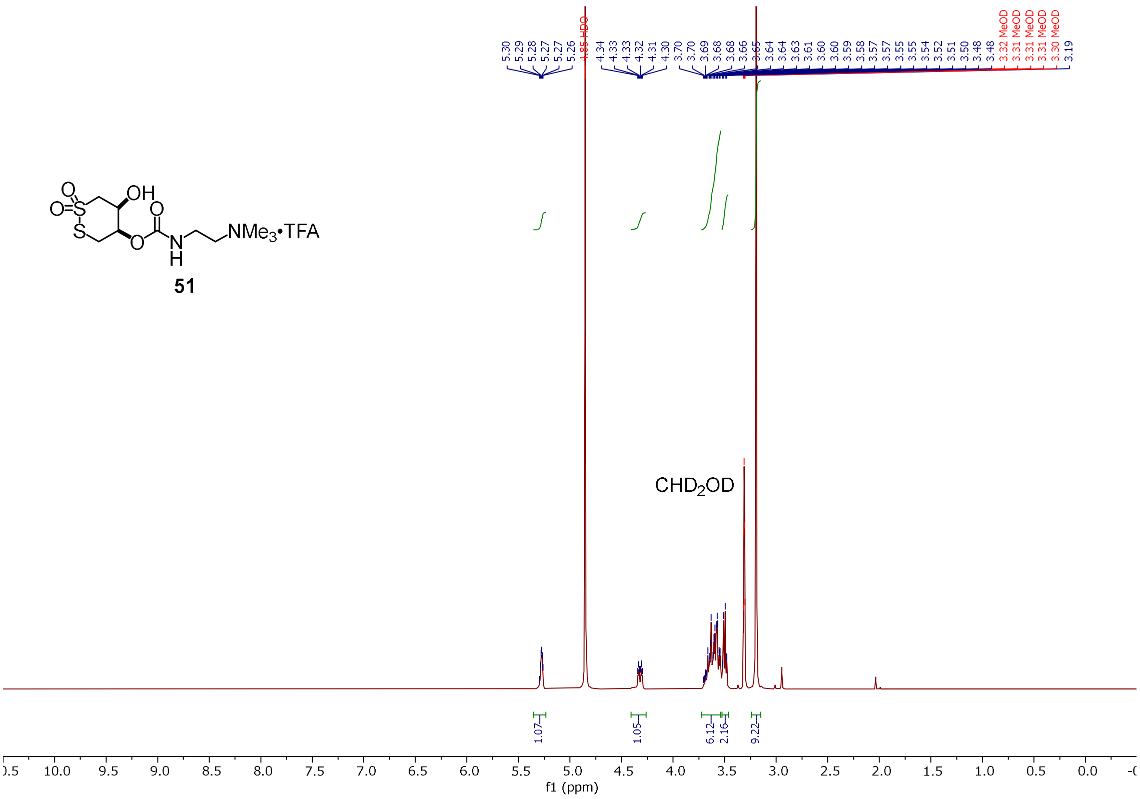

Figure S82. ^{1}H NMR (400 MHz, CD_3OD) spectrum of **51**.

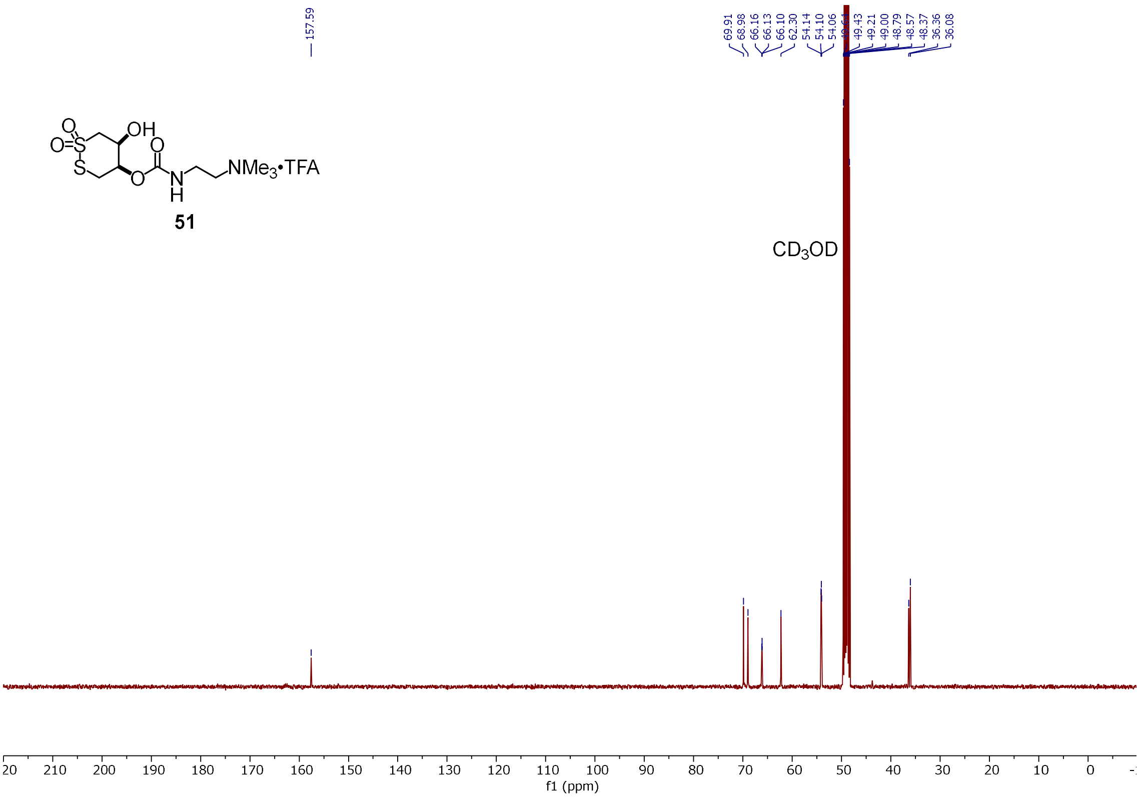

Figure S83. ^{13}C NMR (101 MHz, CD_3OD) spectrum of **51**.

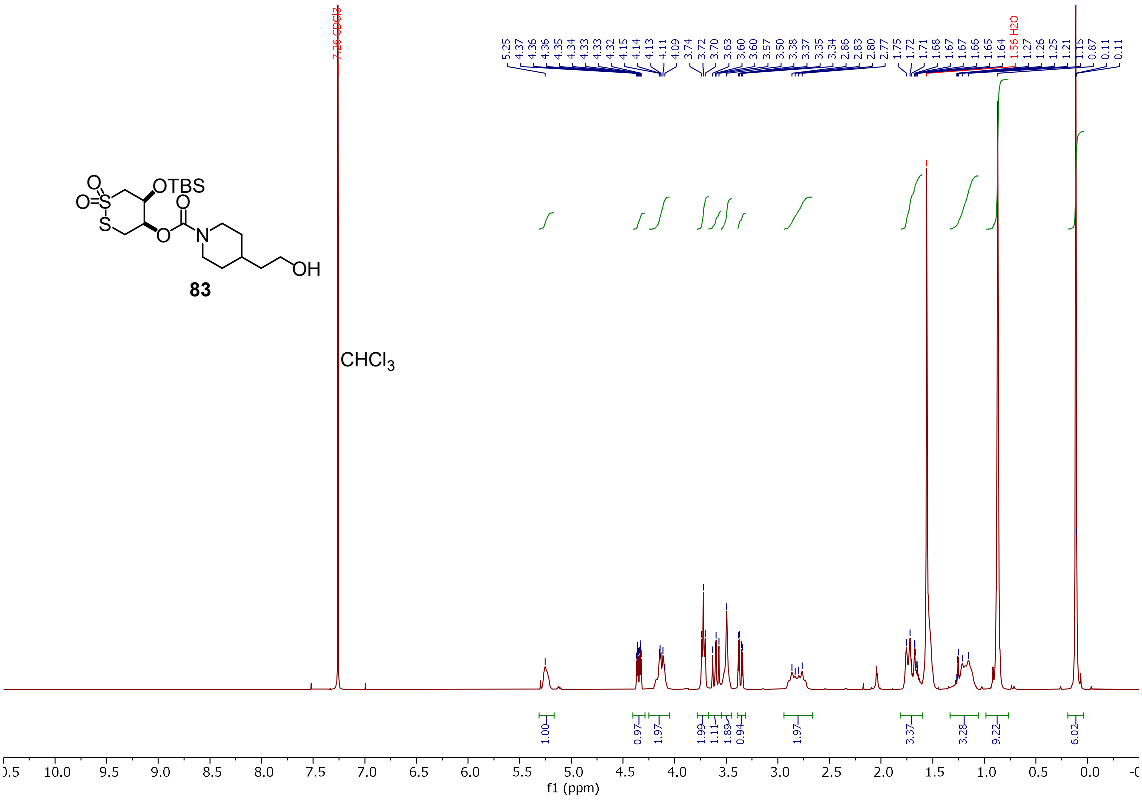

Figure S84. ^{1}H NMR (400 MHz, $CDCl_3$) spectrum of **83**.

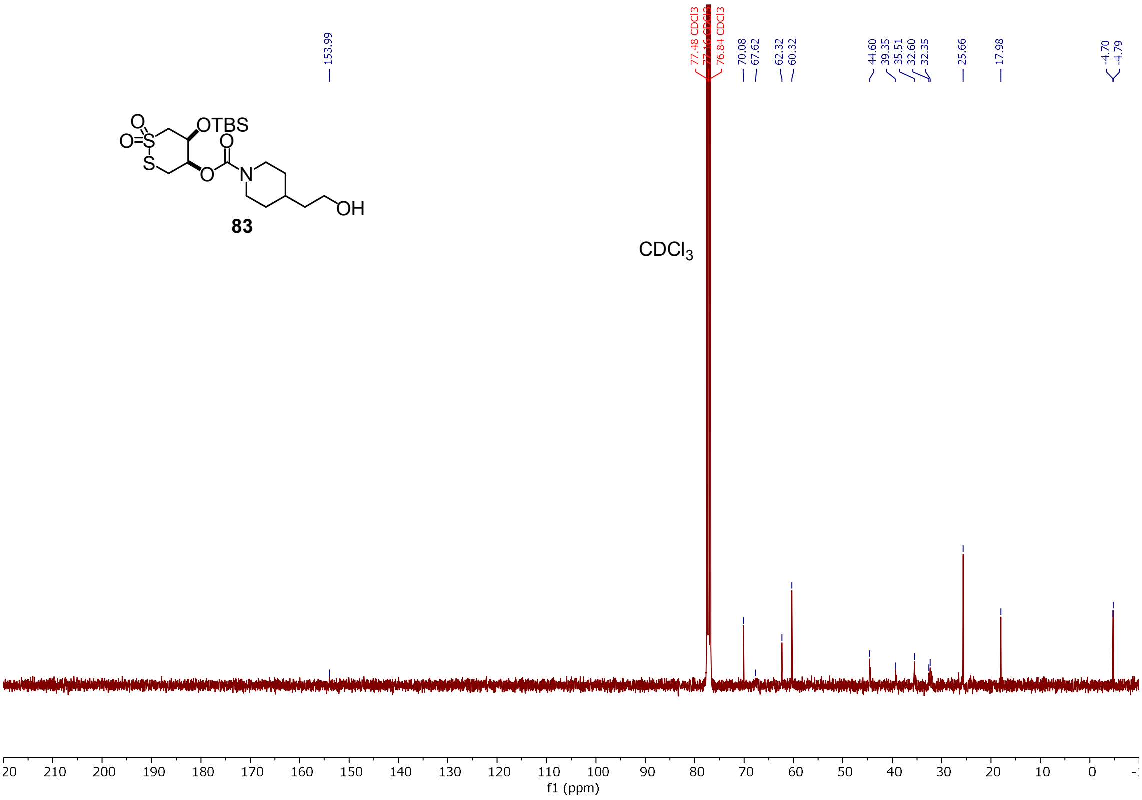

Figure S85. ^{13}C NMR (101 MHz, $CDCl_3$) spectrum of **83**.

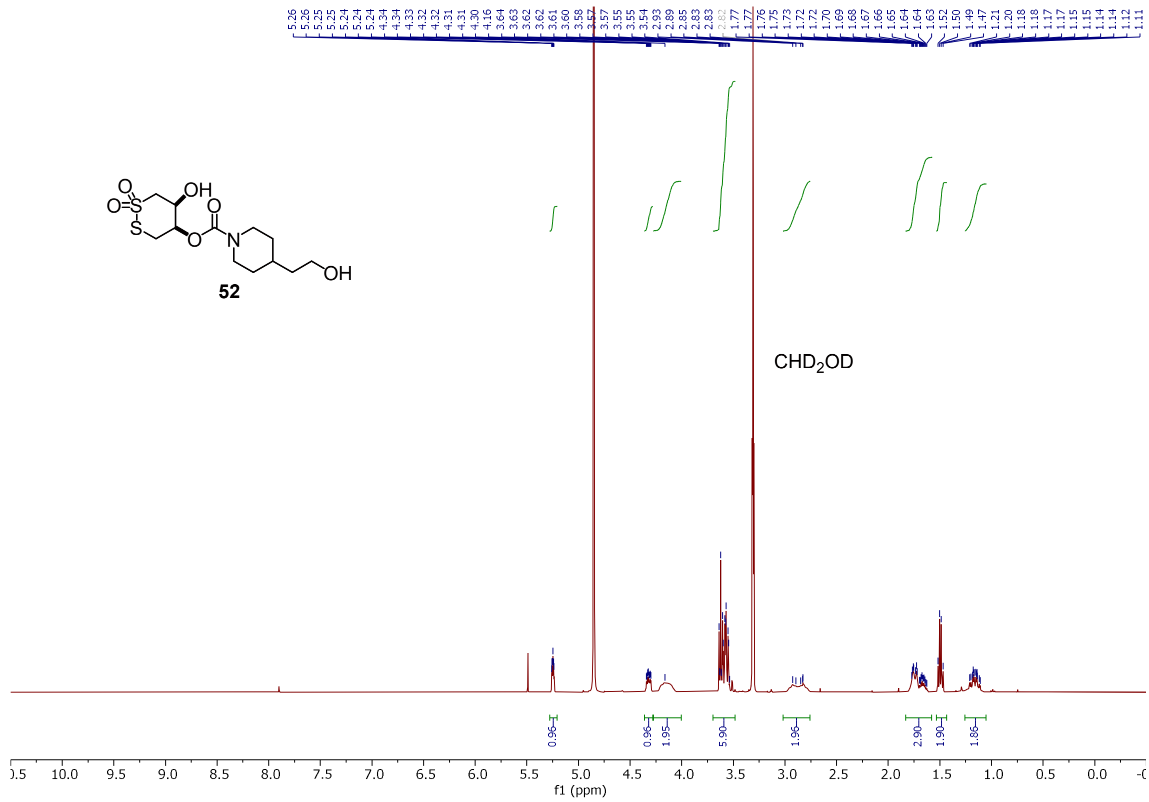

Figure S86. ^{1}H NMR (400 MHz, CD_3OD) spectrum of **52**.

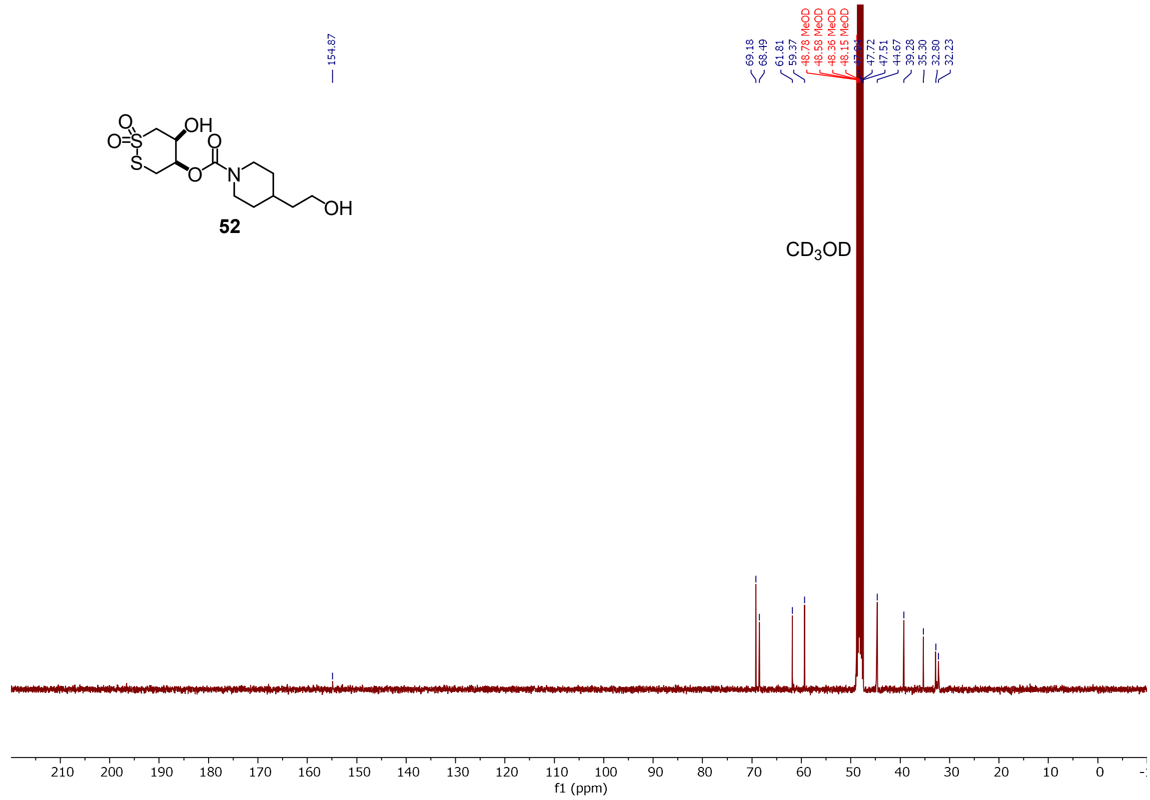

Figure S87. ^{13}C NMR (101 MHz, CD_3OD) spectrum of **52**.

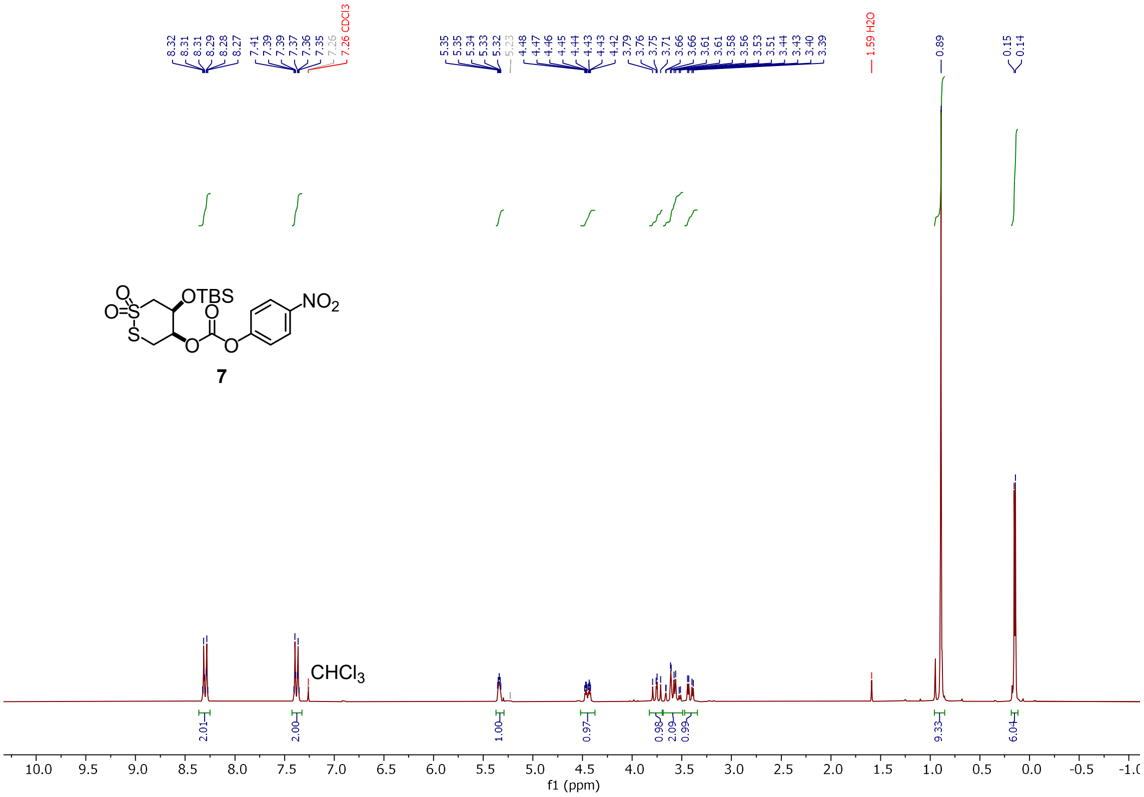

Figure S88. ^{1}H NMR (300 MHz, $CDCl_3$) spectrum of **7**.

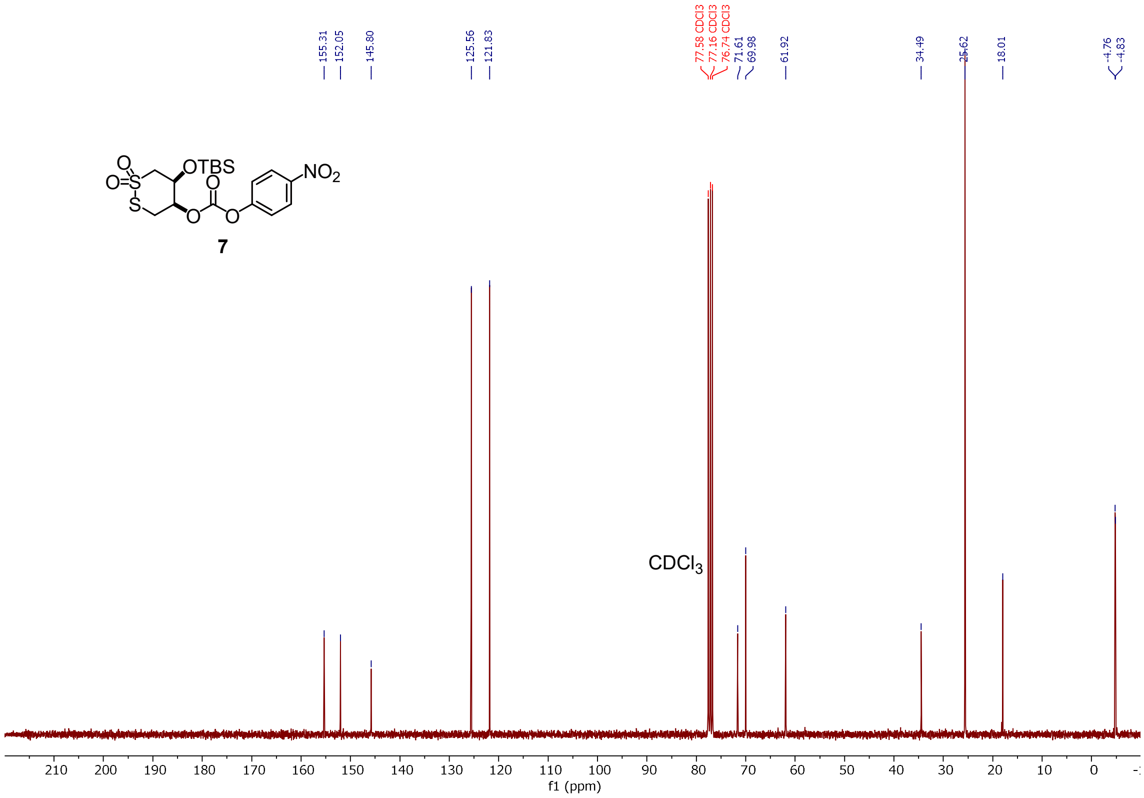

Figure S89. ^{13}C NMR (75 MHz, $CDCl_3$) spectrum of **7**.

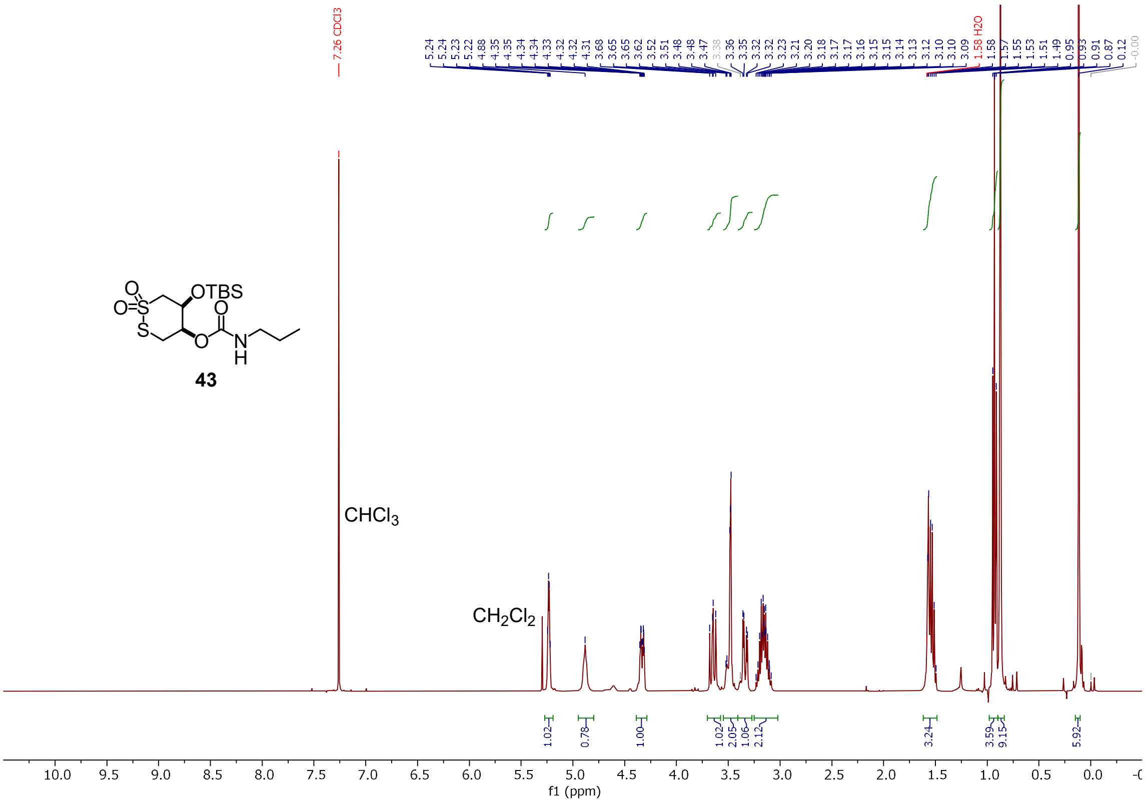

Figure S90. ^{1}H NMR (400 MHz, $CDCl_3$) spectrum of **43**.

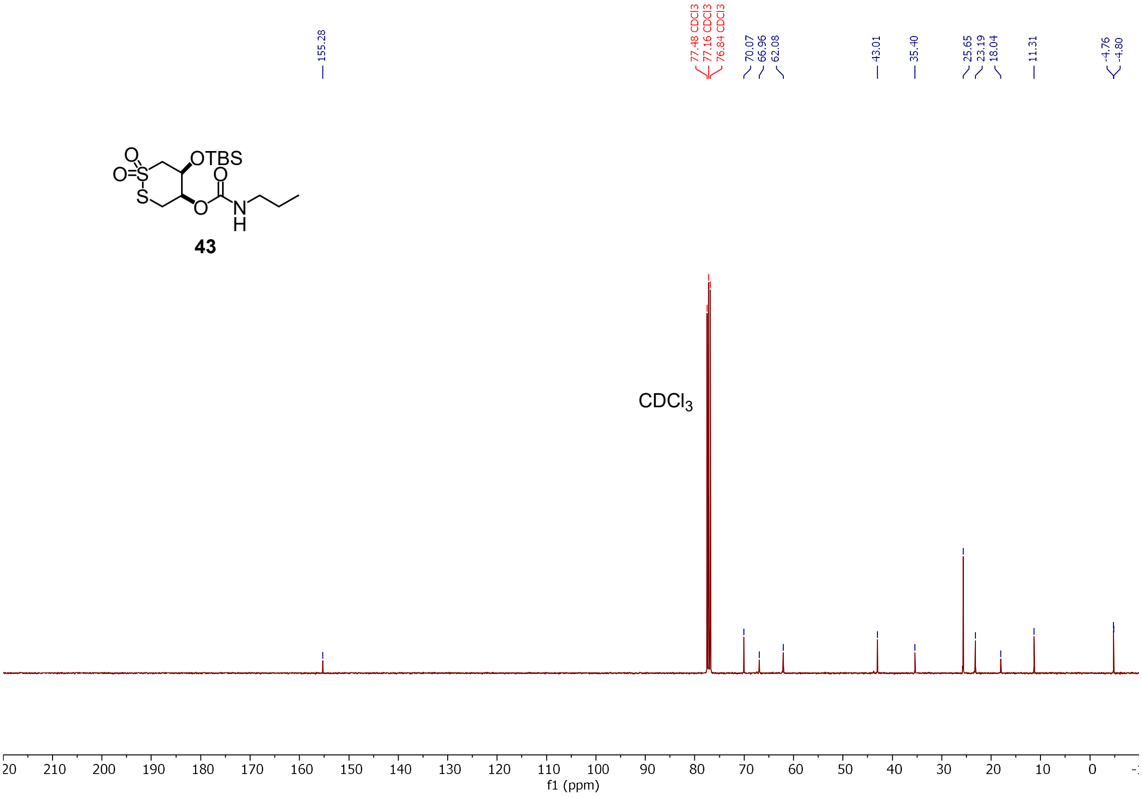

Figure S91. ^{13}C NMR (101 MHz, $CDCl_3$) spectrum of **43**.

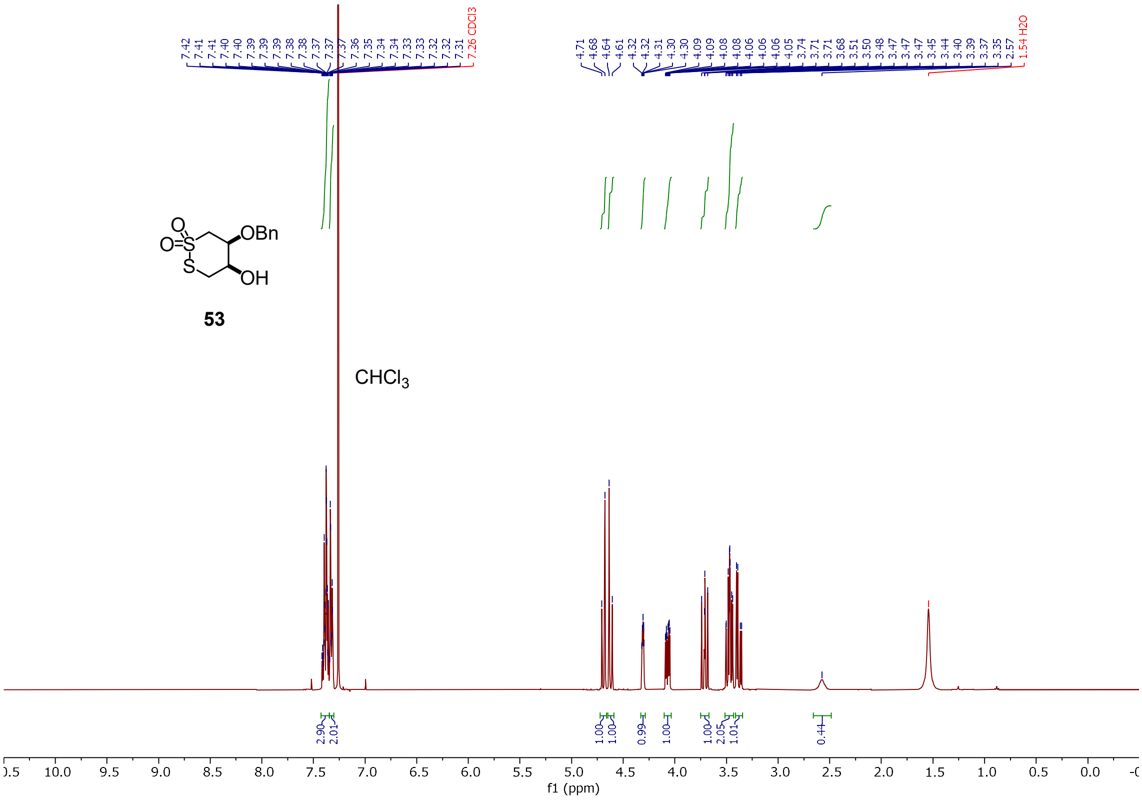

Figure S92. ^{1}H NMR (400 MHz, $CDCl_3$) spectrum of **53**.

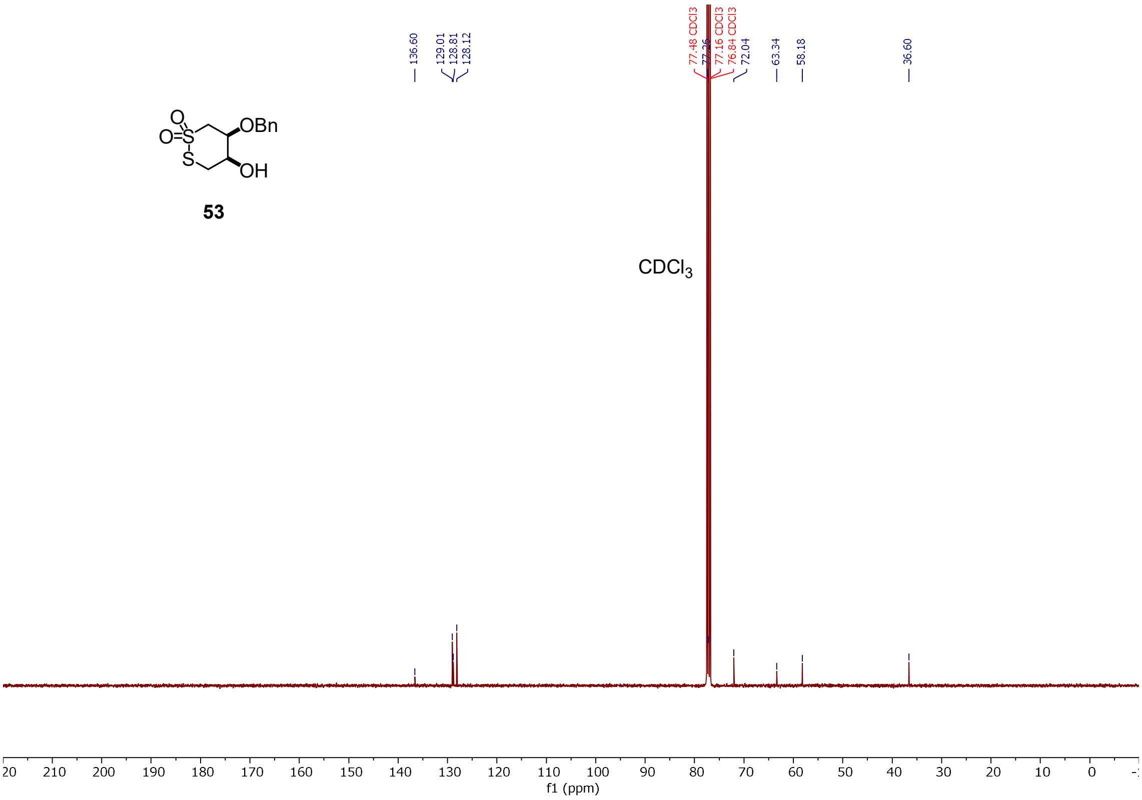

Figure S93. ^{13}C NMR (101 MHz, $CDCl_3$) spectrum of **53**.

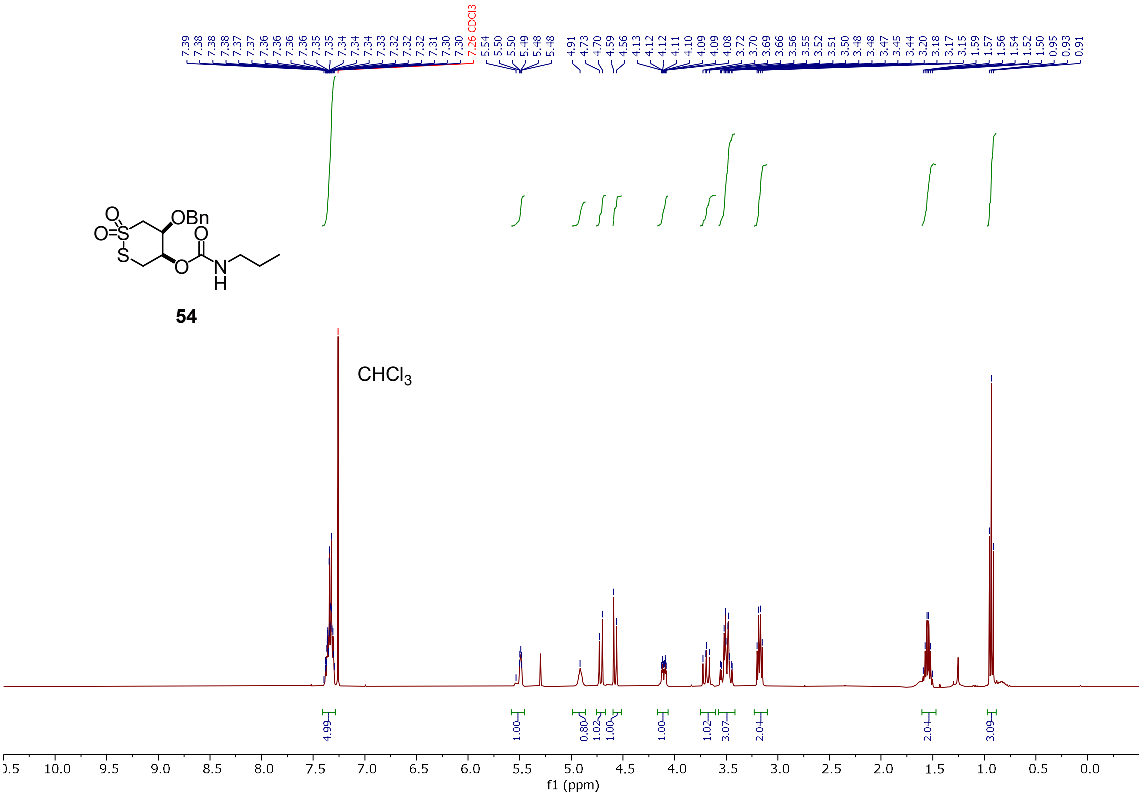

Figure S94. ^1H NMR (400 MHz, $CDCl_3$) spectrum of **54**.

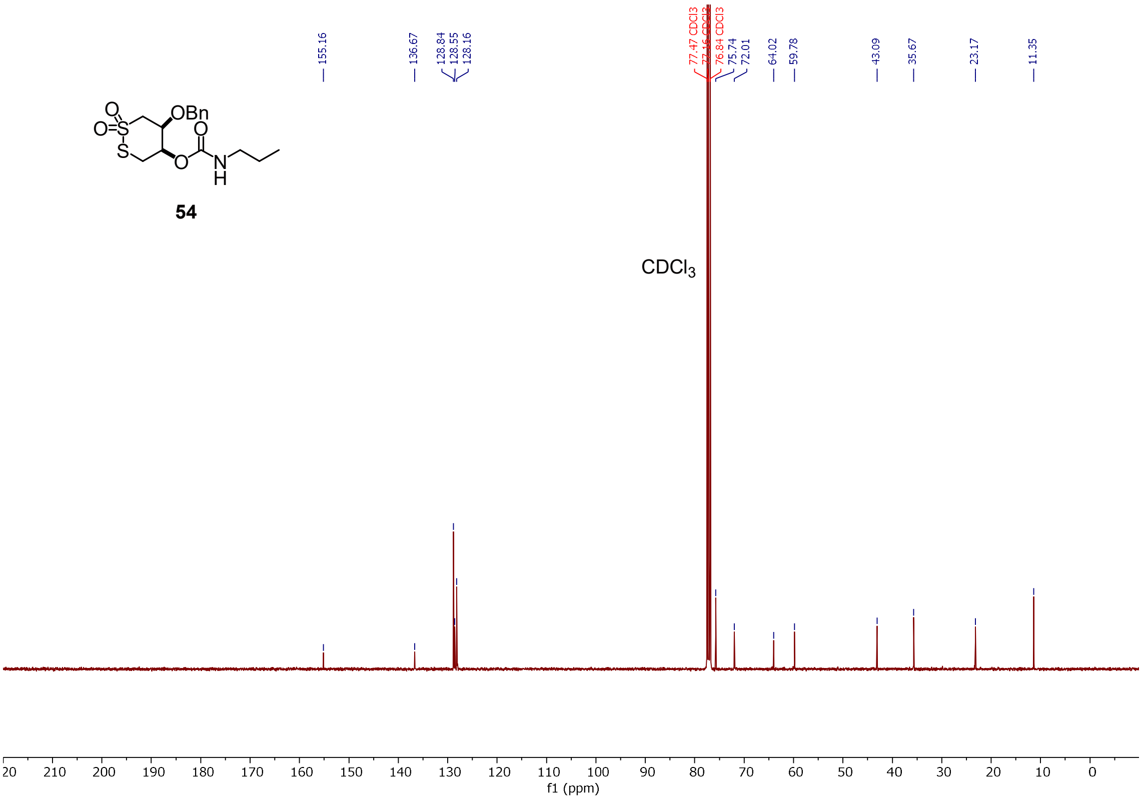

Figure S95. ^{13}C NMR (101 MHz, $CDCl_3$) spectrum of **54**.

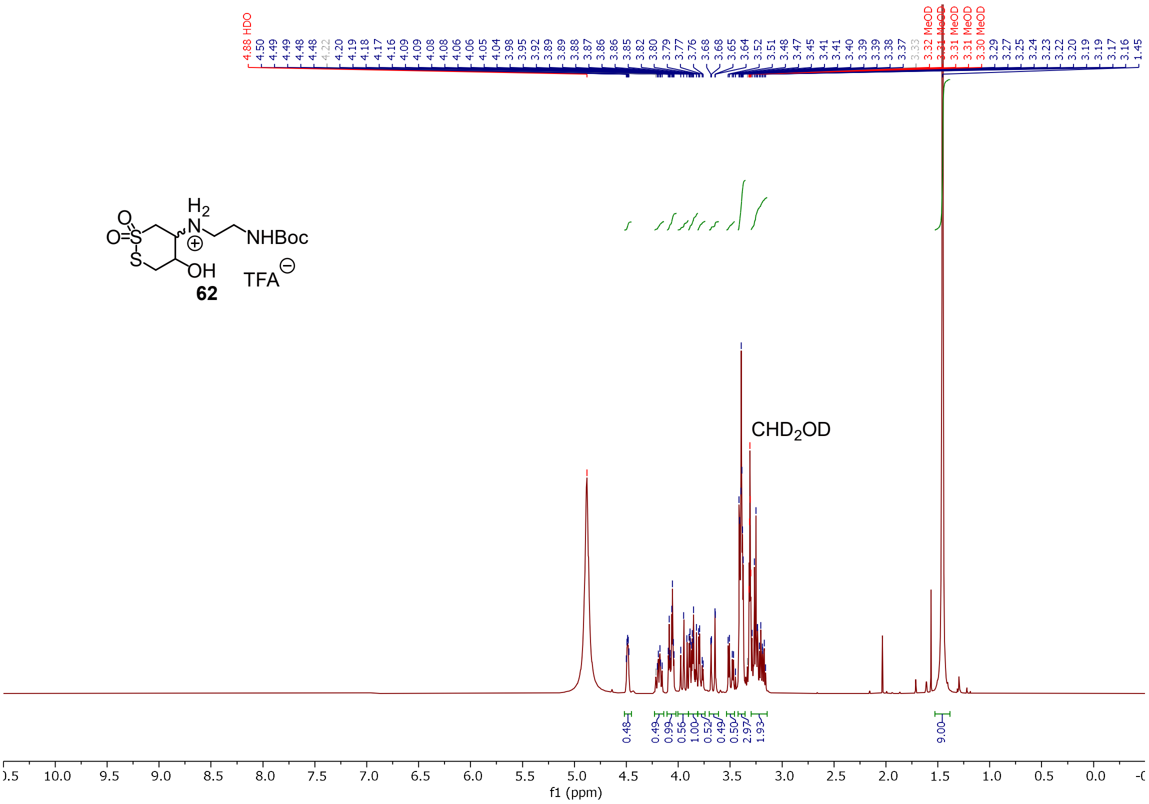

Figure S96. ^{1}H NMR (400 MHz, CD_3OD) spectrum of **62**.

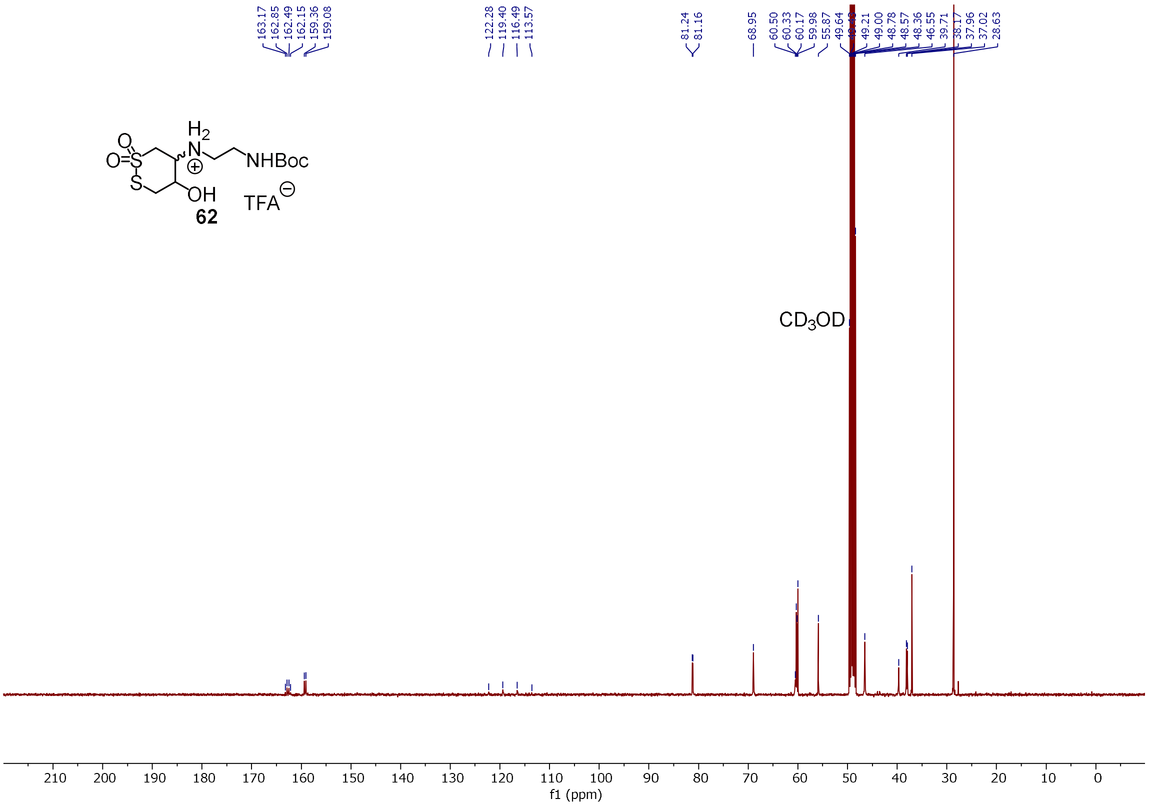

Figure S97. ^{13}C NMR (101 MHz, CD_3OD) spectrum of **62**.

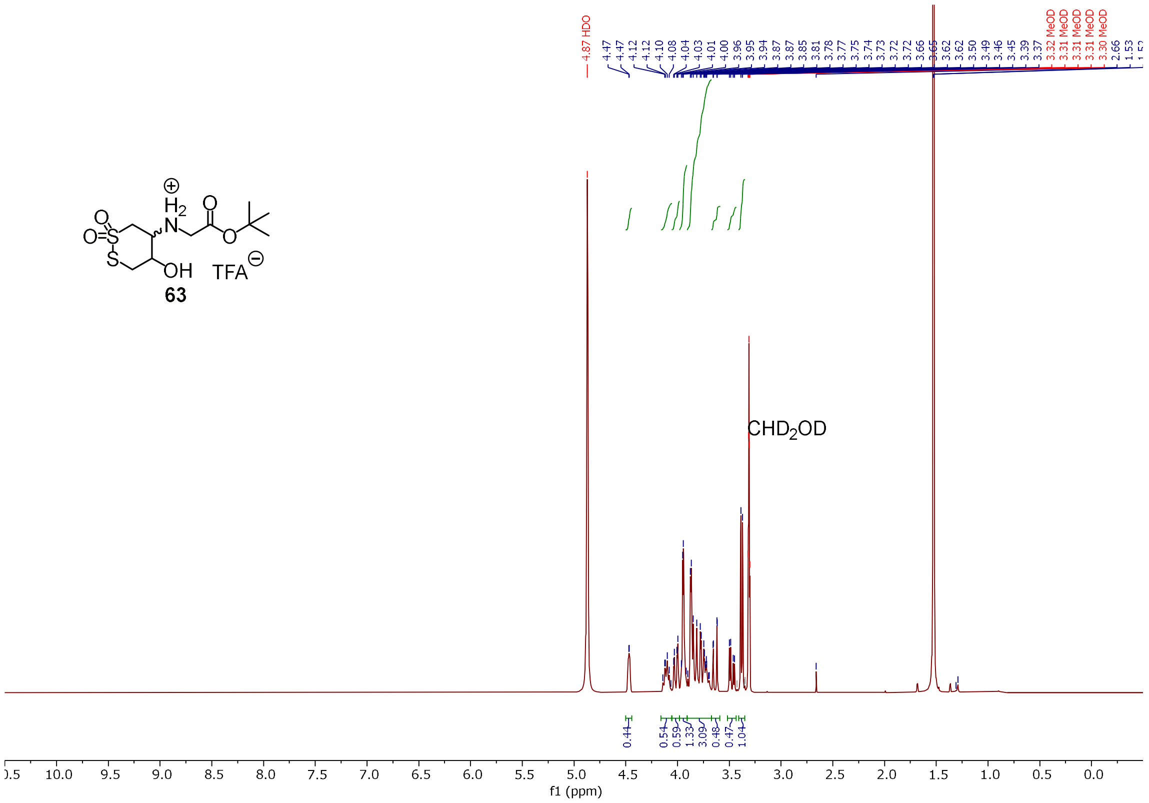

Figure S98. ^{1}H NMR (400 MHz, CD_3OD) spectrum of **63**.

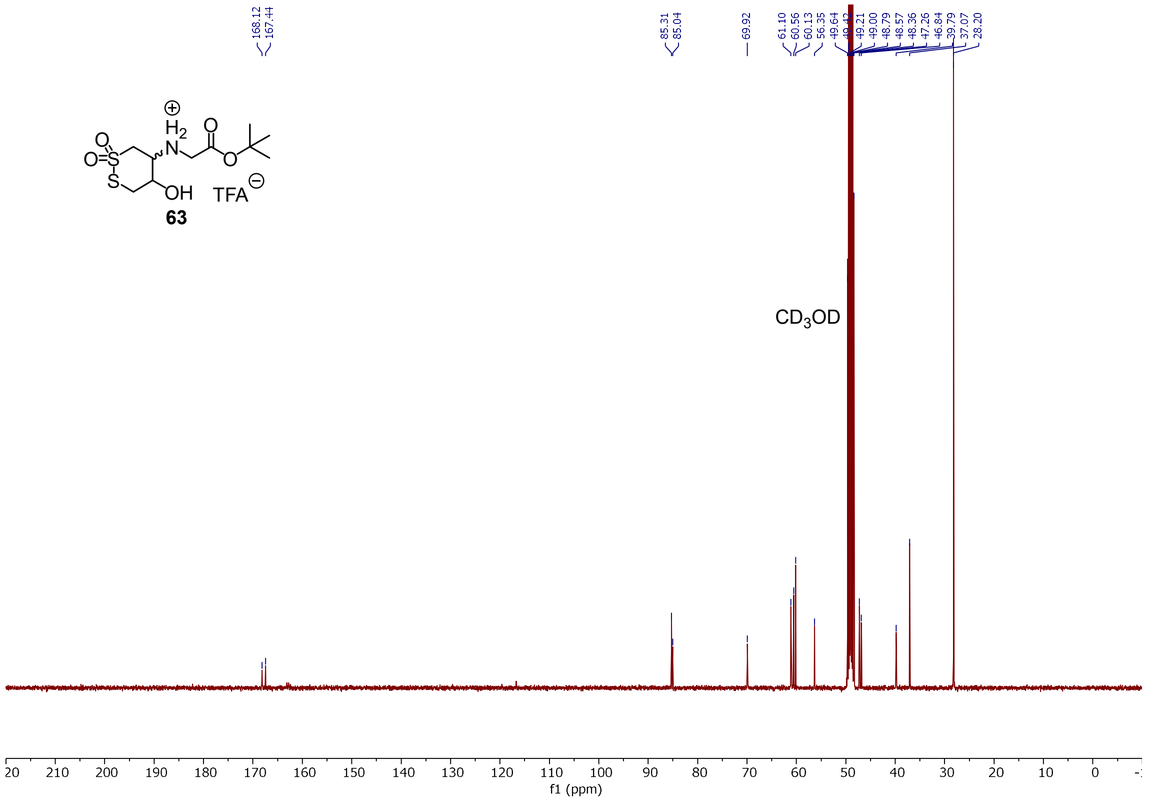

Figure S99. ^{13}C NMR (101 MHz, CD_3OD) spectrum of **63**.

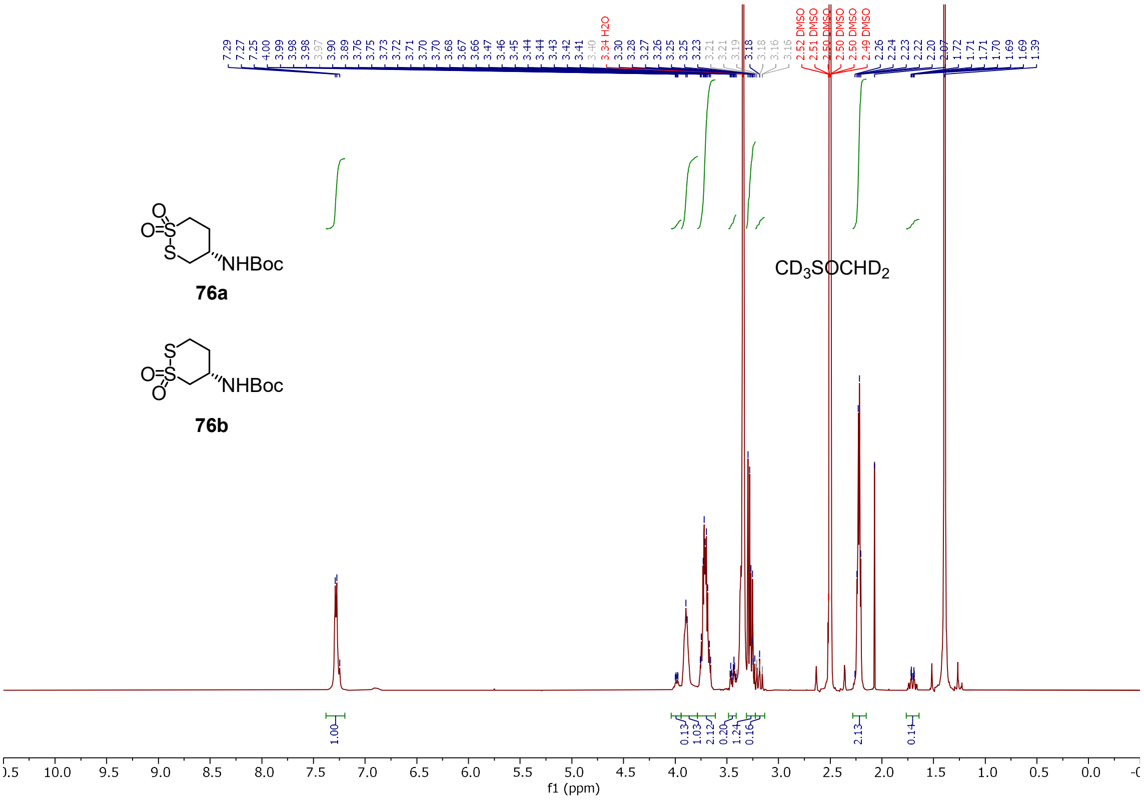

Figure S100. ^{1}H NMR (500 MHz, DMSO-*d*$_6$) spectrum of **76**.

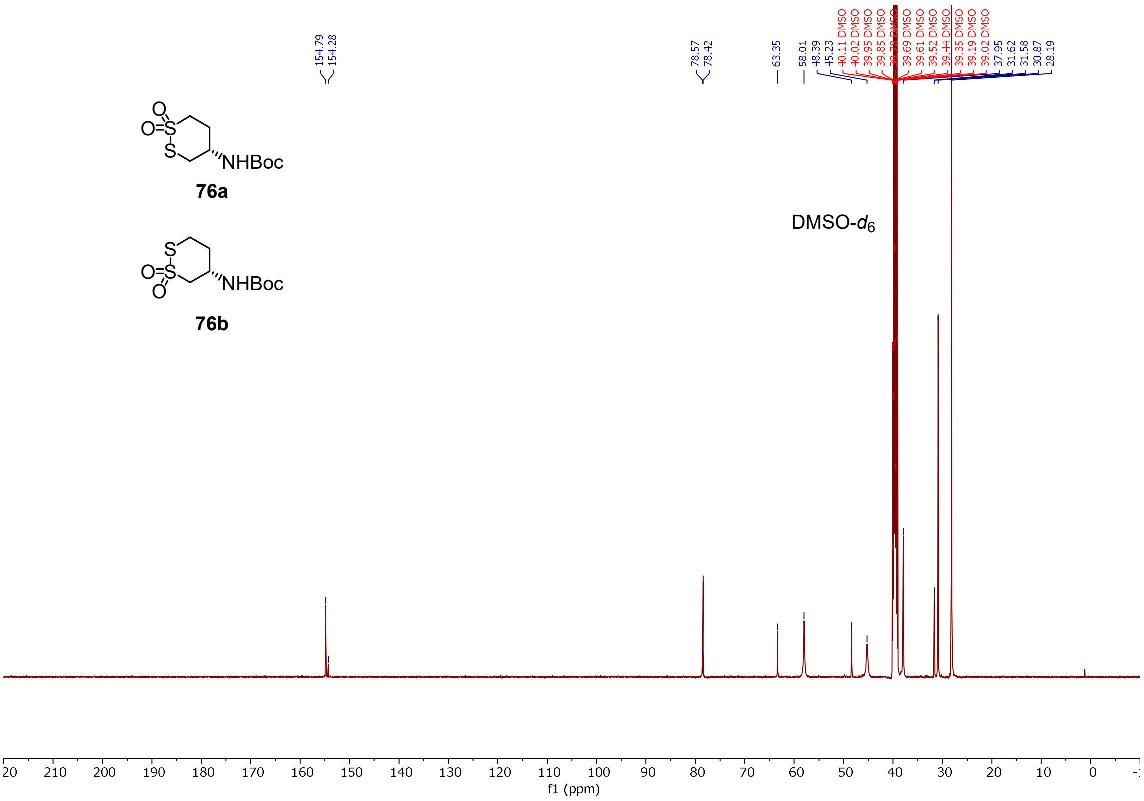

Figure S101. ^{13}C NMR (126 MHz, DMSO-*d*$_6$) spectrum of **76**.

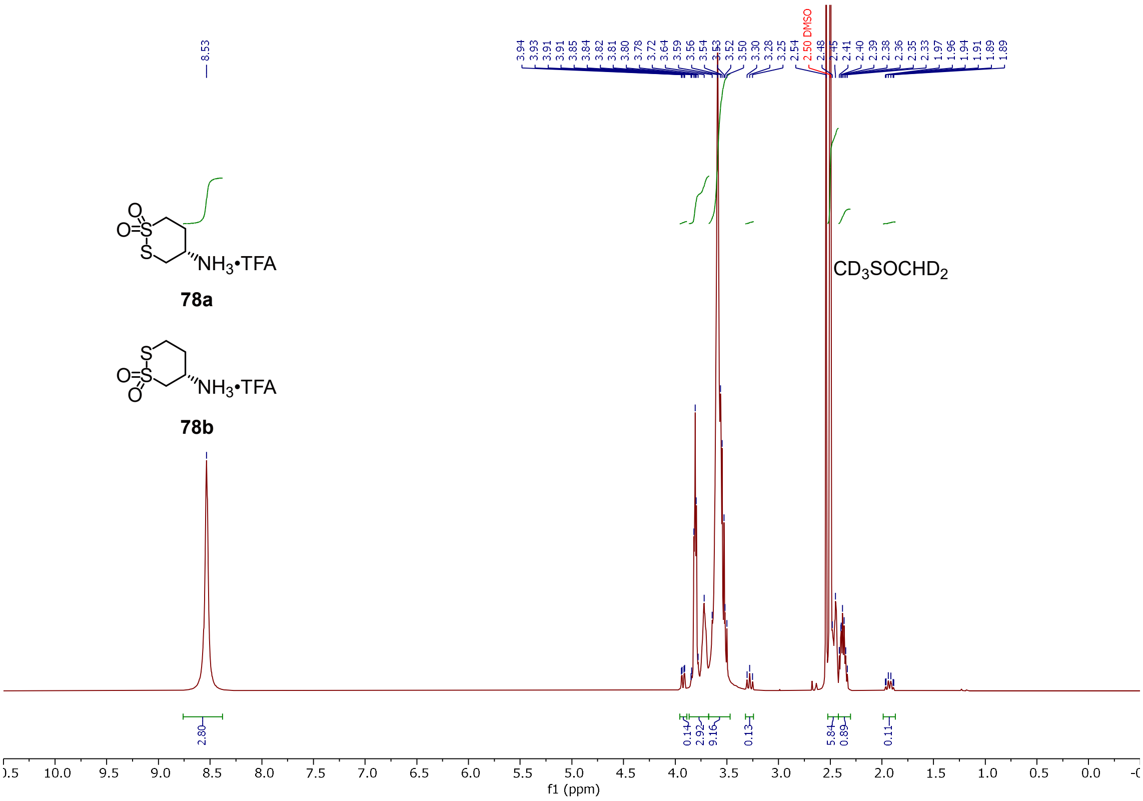

Figure S102. ^{1}H NMR (500 MHz, DMSO-d_6) spectrum of **78**.

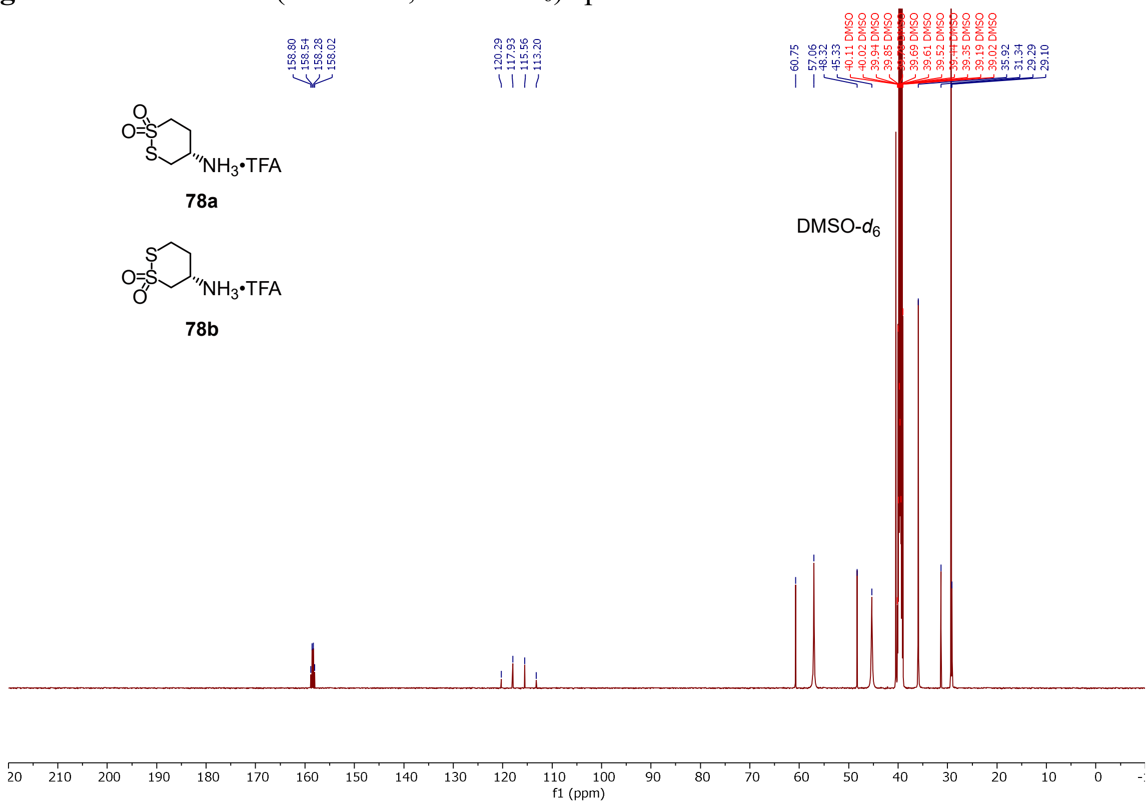

Figure S103. ^{13}C NMR (126 MHz, DMSO-d_6) spectrum of **78**.

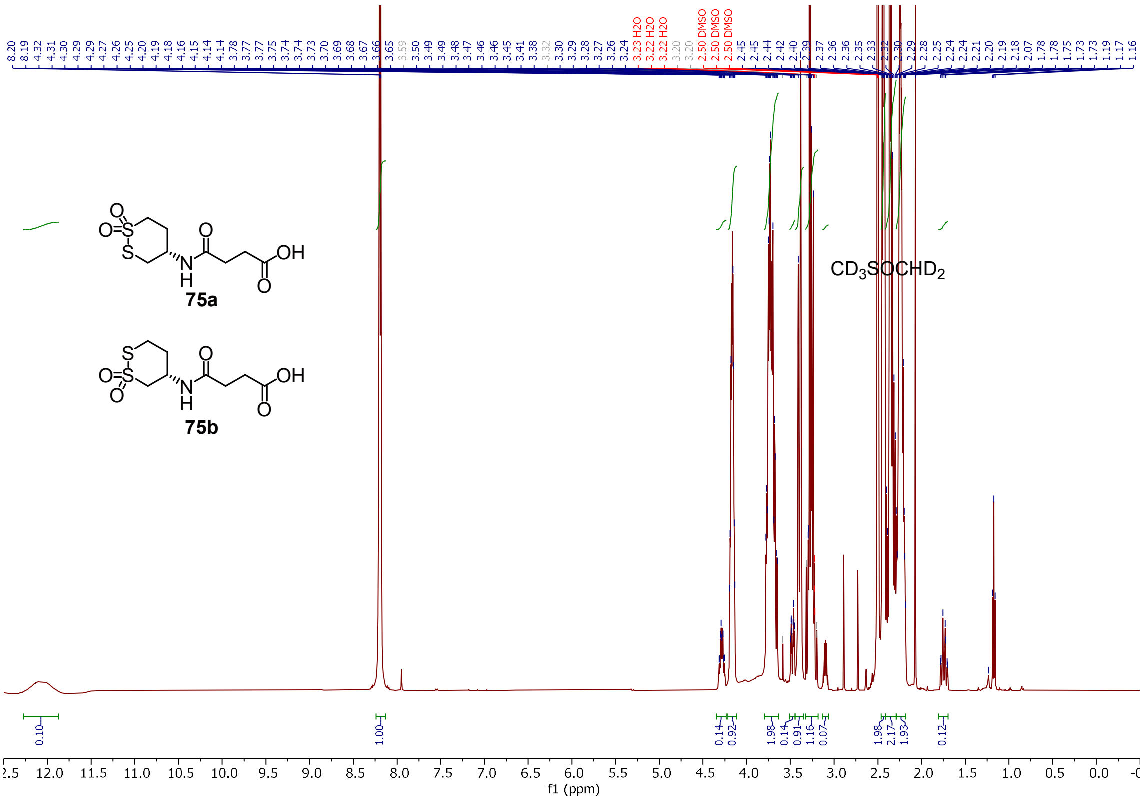

Figure S104. ^{1}H NMR (500 MHz, DMSO-d_6) spectrum of **75**.

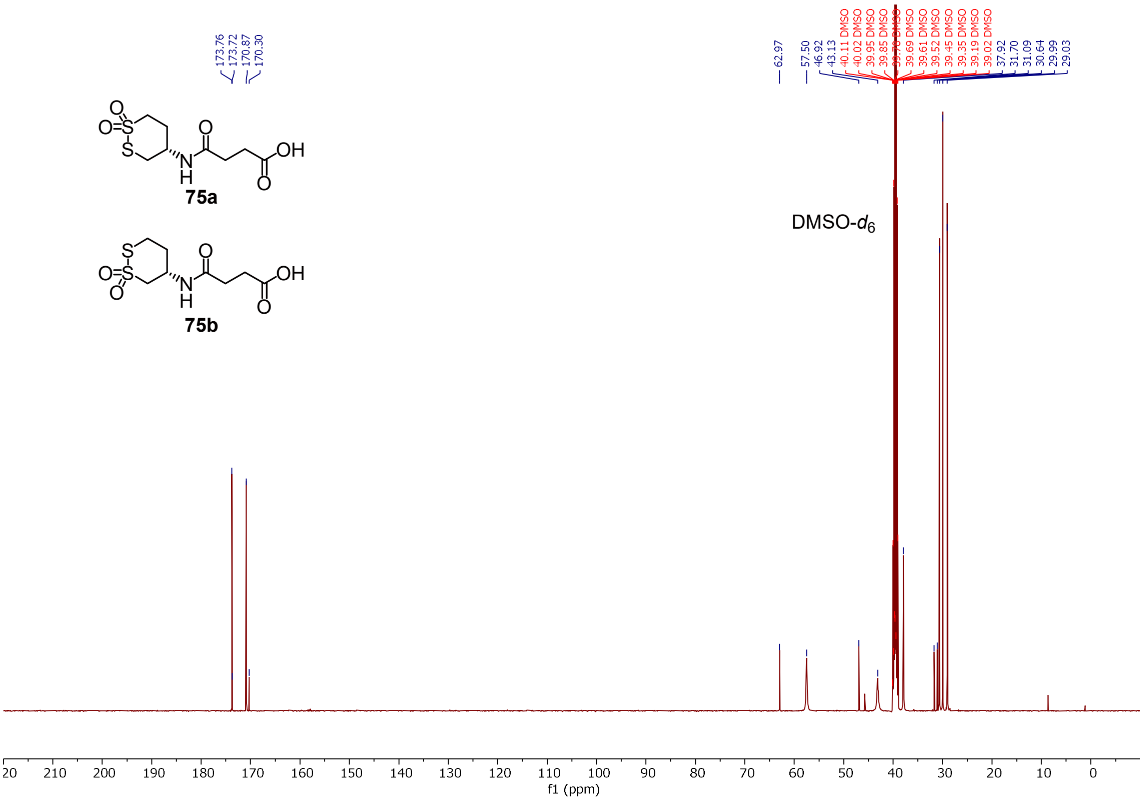

Figure S105. ^{13}C NMR (126 MHz, DMSO-d_6) spectrum of **75**.

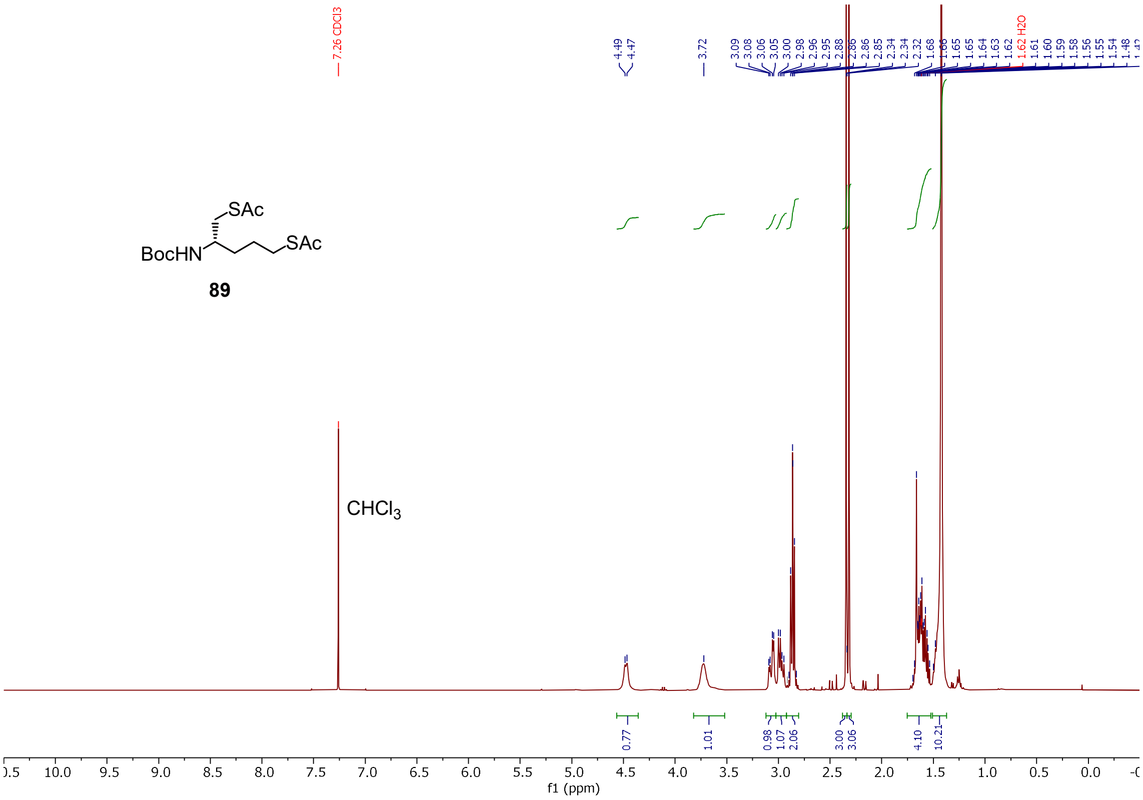

Figure S106. ^{1}H NMR (400 MHz, $CDCl_3$) spectrum of **89**.

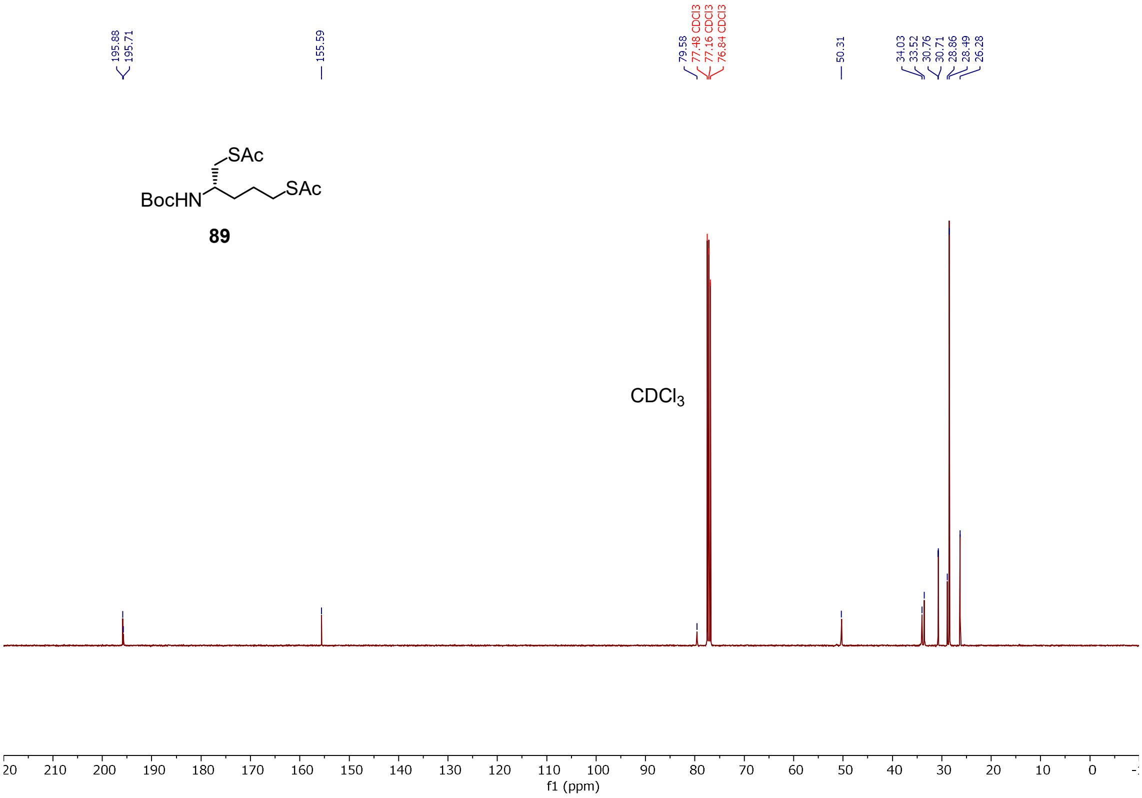

Figure S107. ^{13}C NMR (101 MHz, $CDCl_3$) spectrum of **89**.

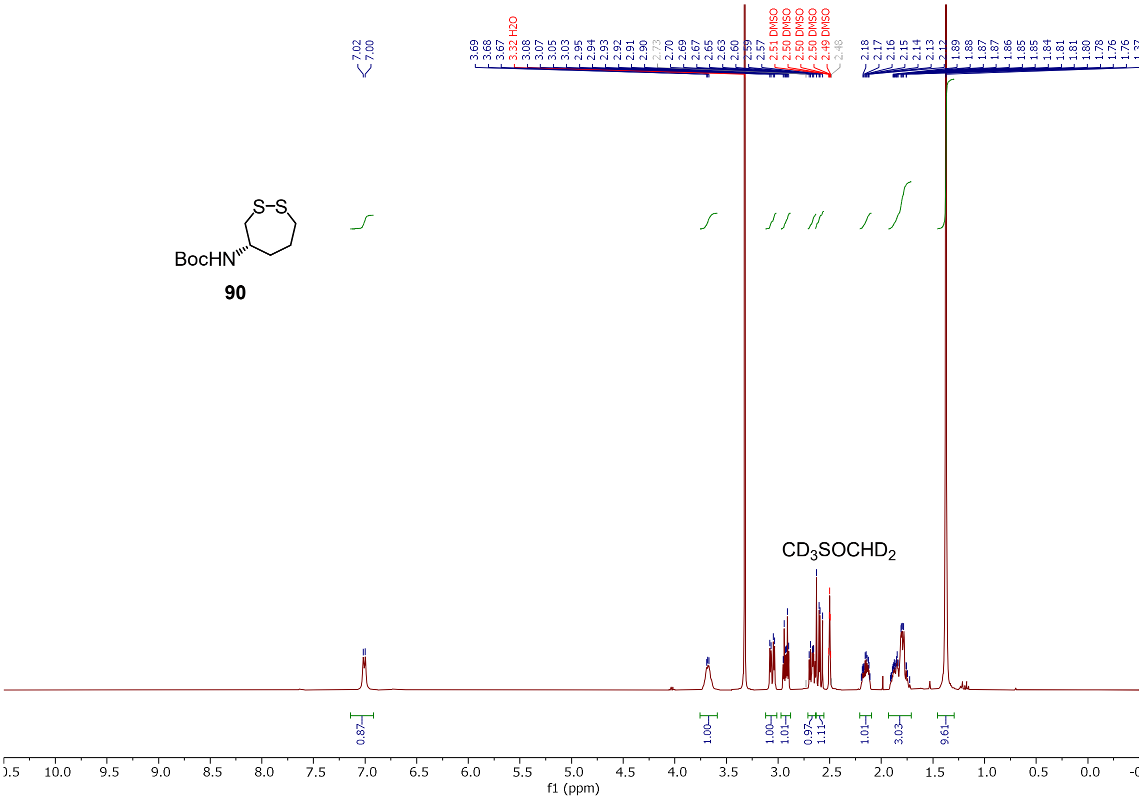

Figure S108. ^{1}H NMR (400 MHz, DMSO-d_6) spectrum of **90**.

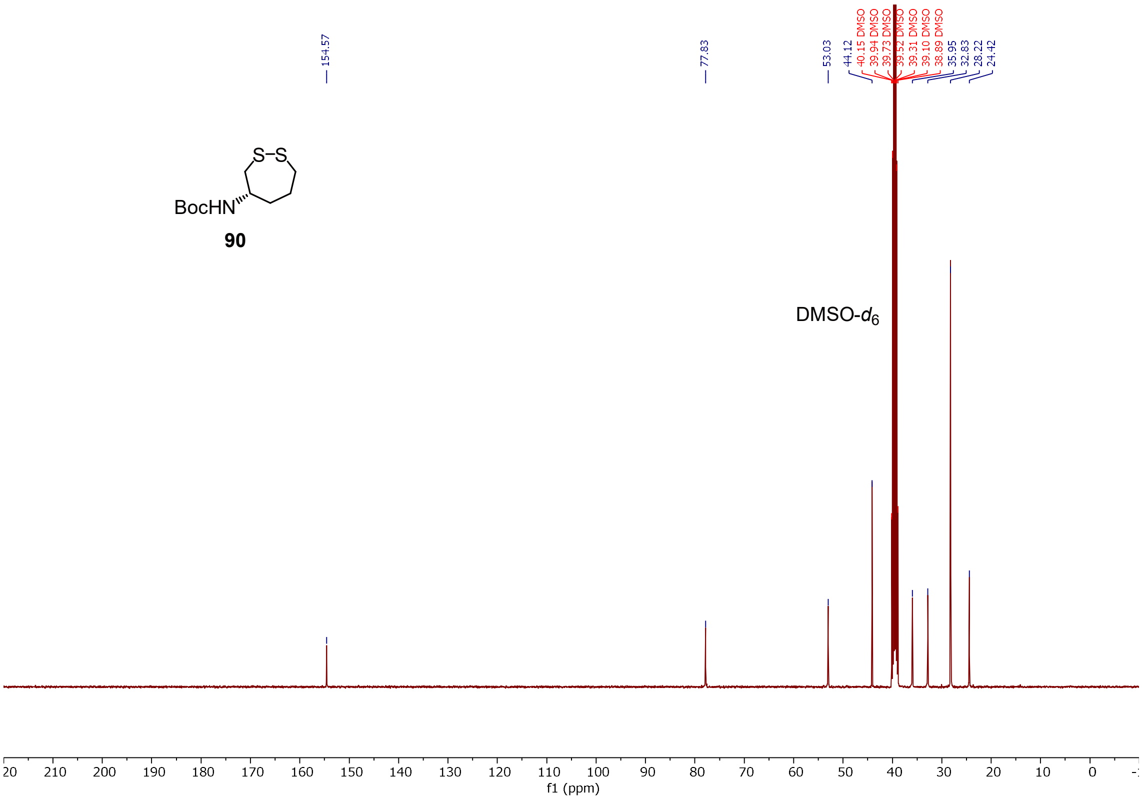

Figure S109. ^{13}C NMR (101 MHz, DMSO-d_6) spectrum of **90**.

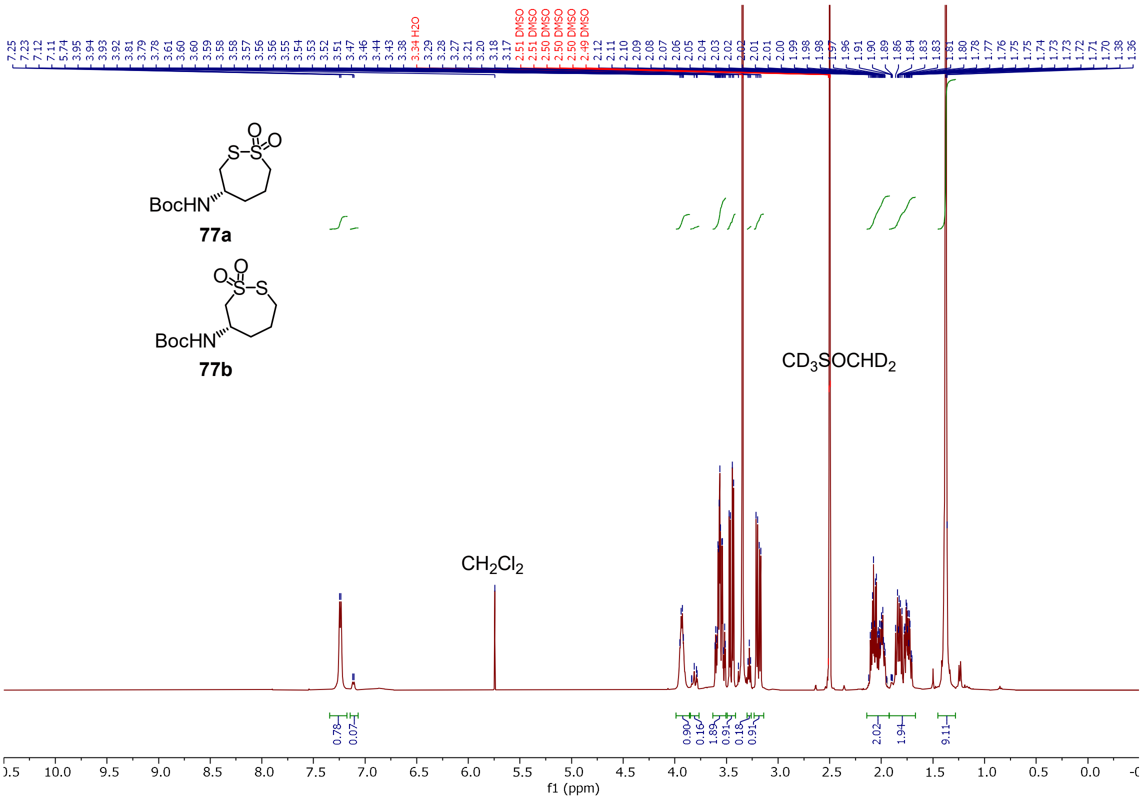

Figure S110. ^{1}H NMR (500 MHz, DMSO-d_6) spectrum of **77**.

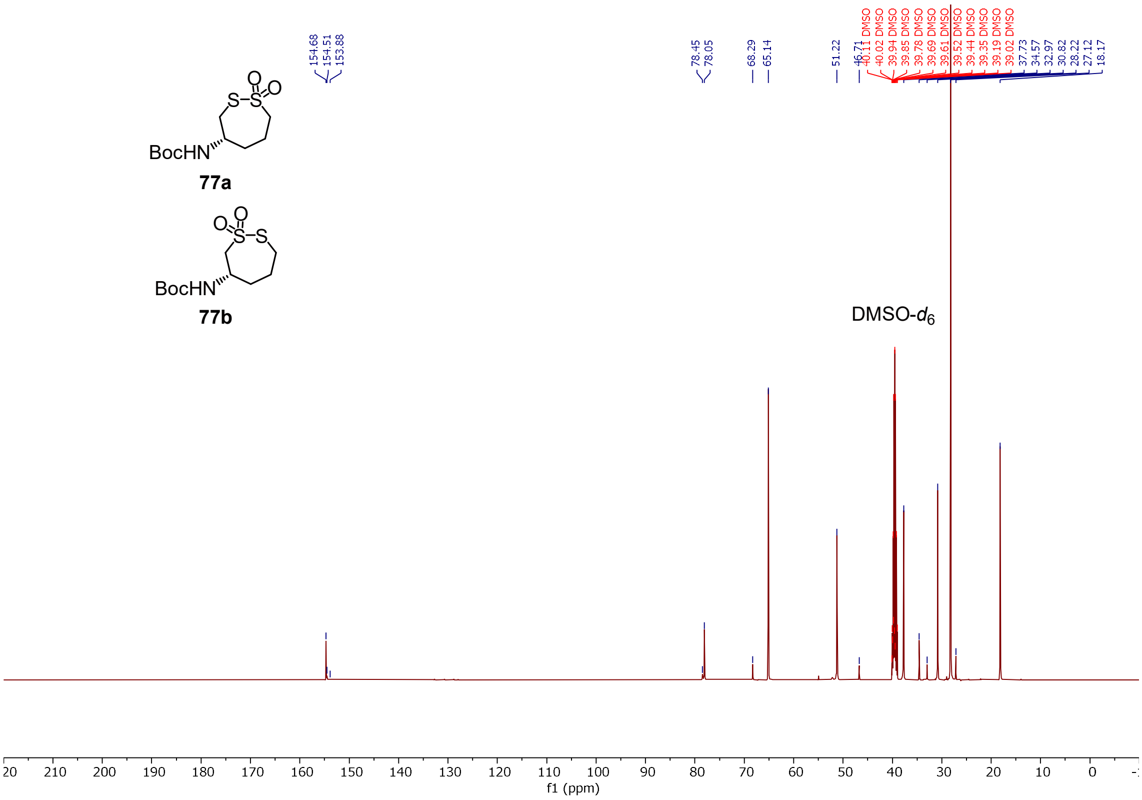

Figure S111. ^{13}C NMR (126 MHz, DMSO-d_6) spectrum of **77**.

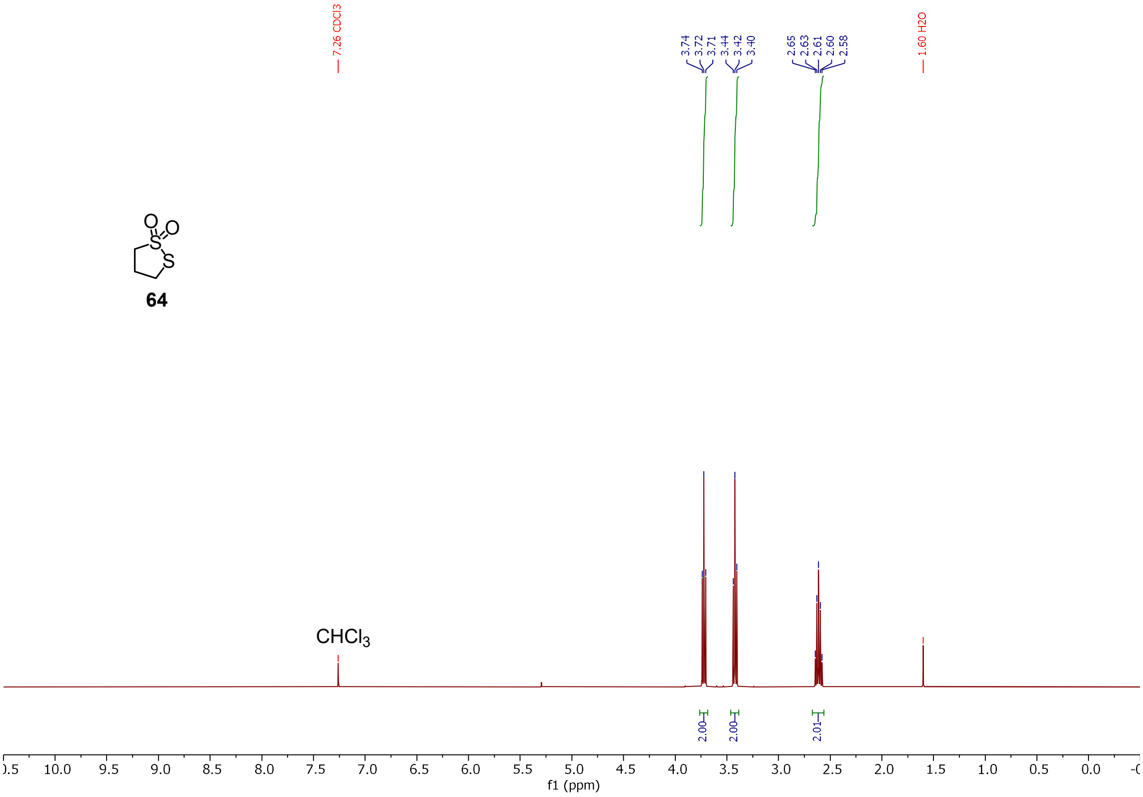

Figure S112. ^{1}H NMR (400 MHz, $CDCl_3$) spectrum of **64**.

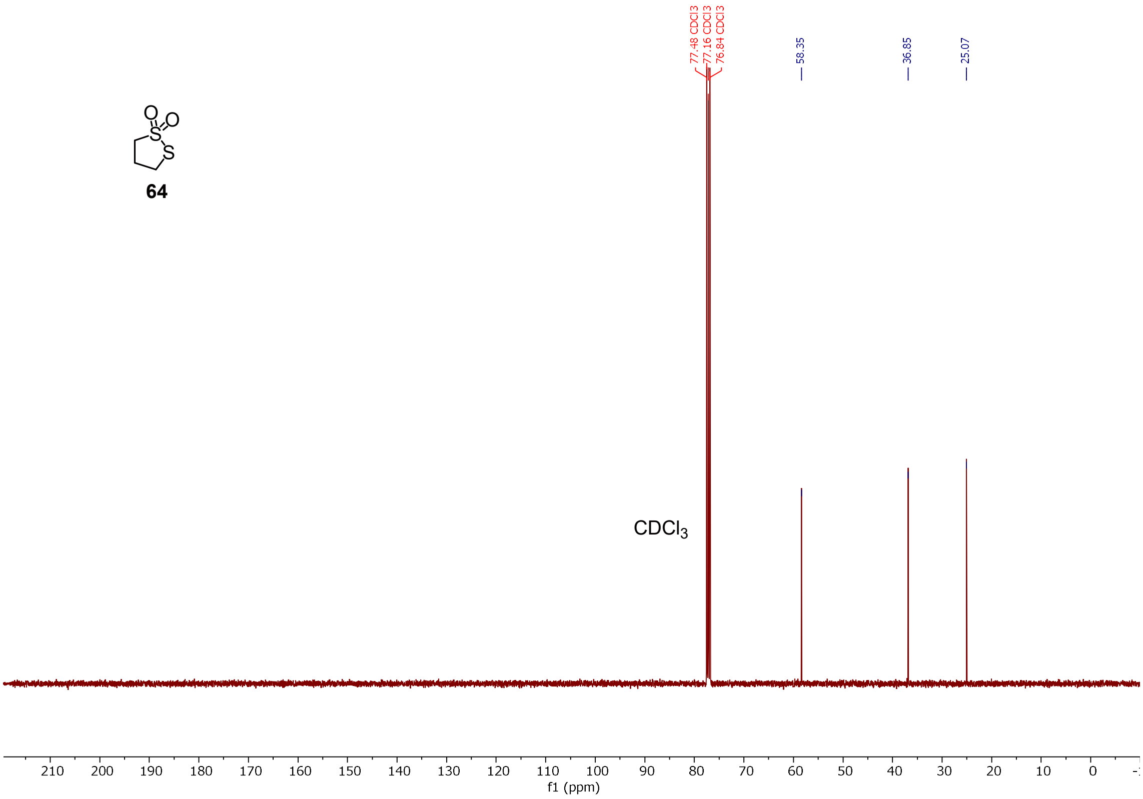

Figure S113. ^{13}C NMR (101 MHz, $CDCl_3$) spectrum of **64**.

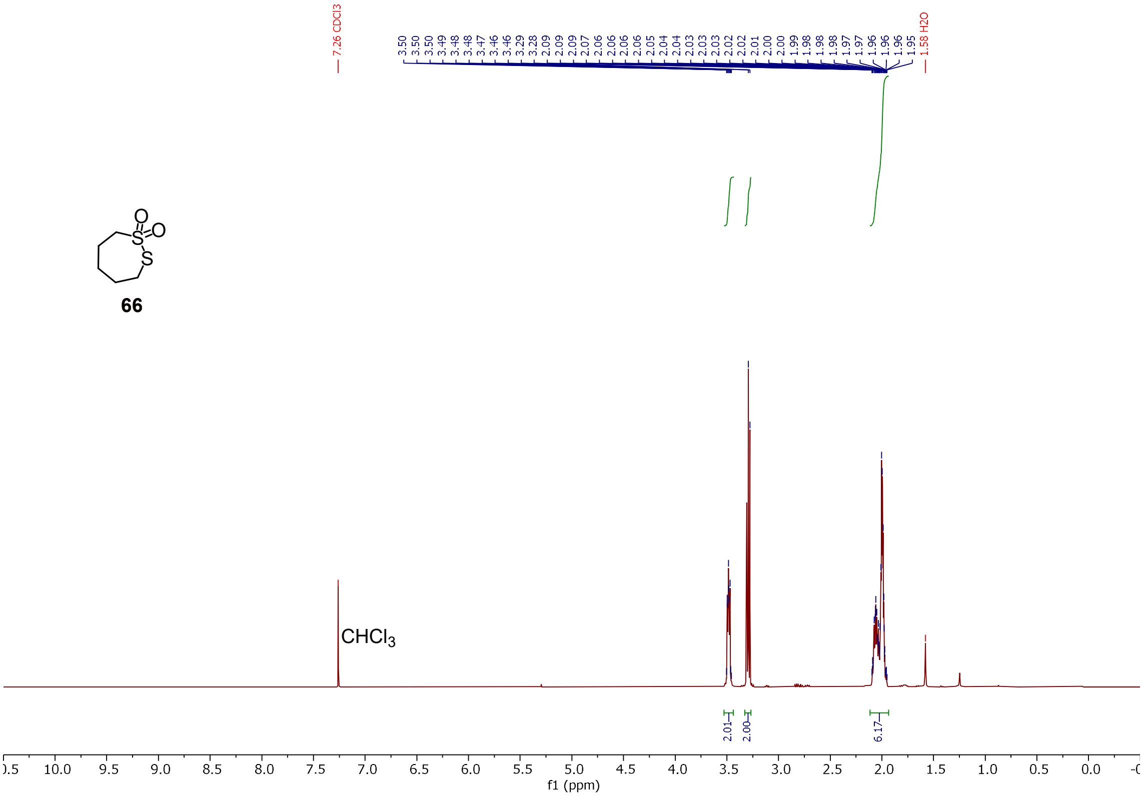

Figure S114. ^{1}H NMR (400 MHz, $CDCl_3$) spectrum of **66**.

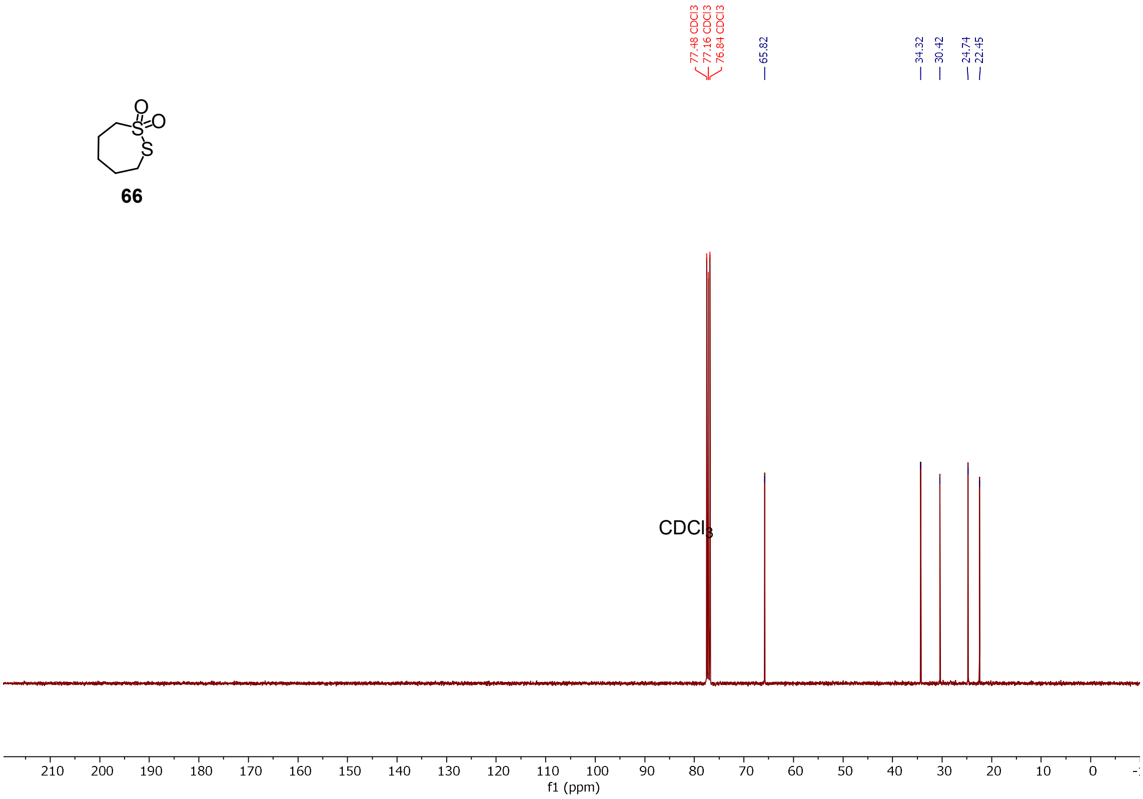

Figure S115. ^{13}C NMR (101 MHz, $CDCl_3$) spectrum of **66**.

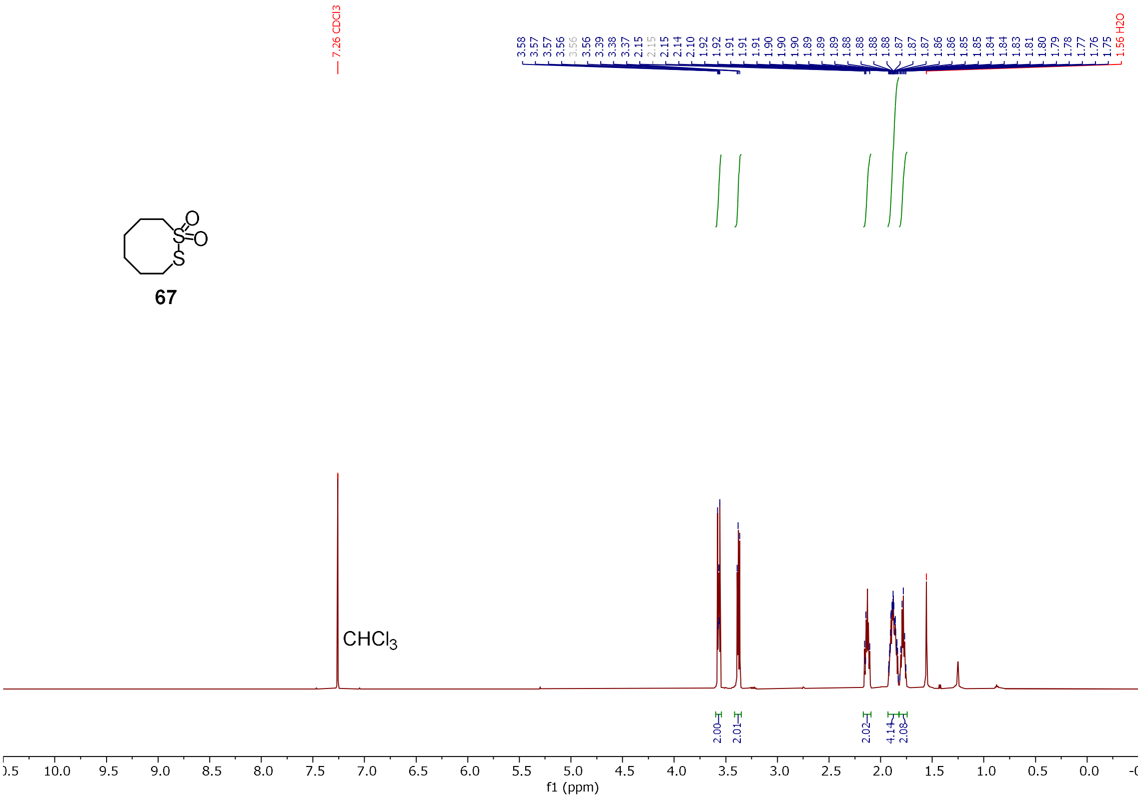

Figure S116. ^{1}H NMR (400 MHz, $CDCl_3$) spectrum of **67**.

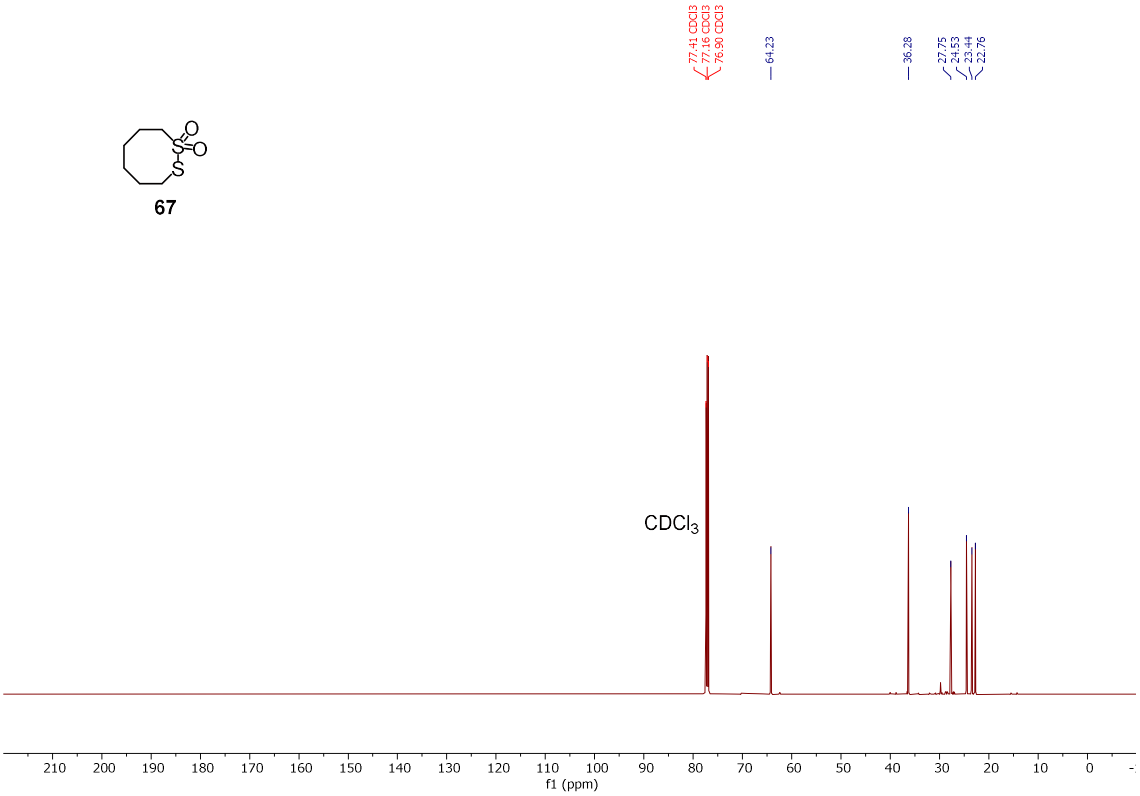

Figure S117. ^{13}C NMR (101 MHz, $CDCl_3$) spectrum of **67**.

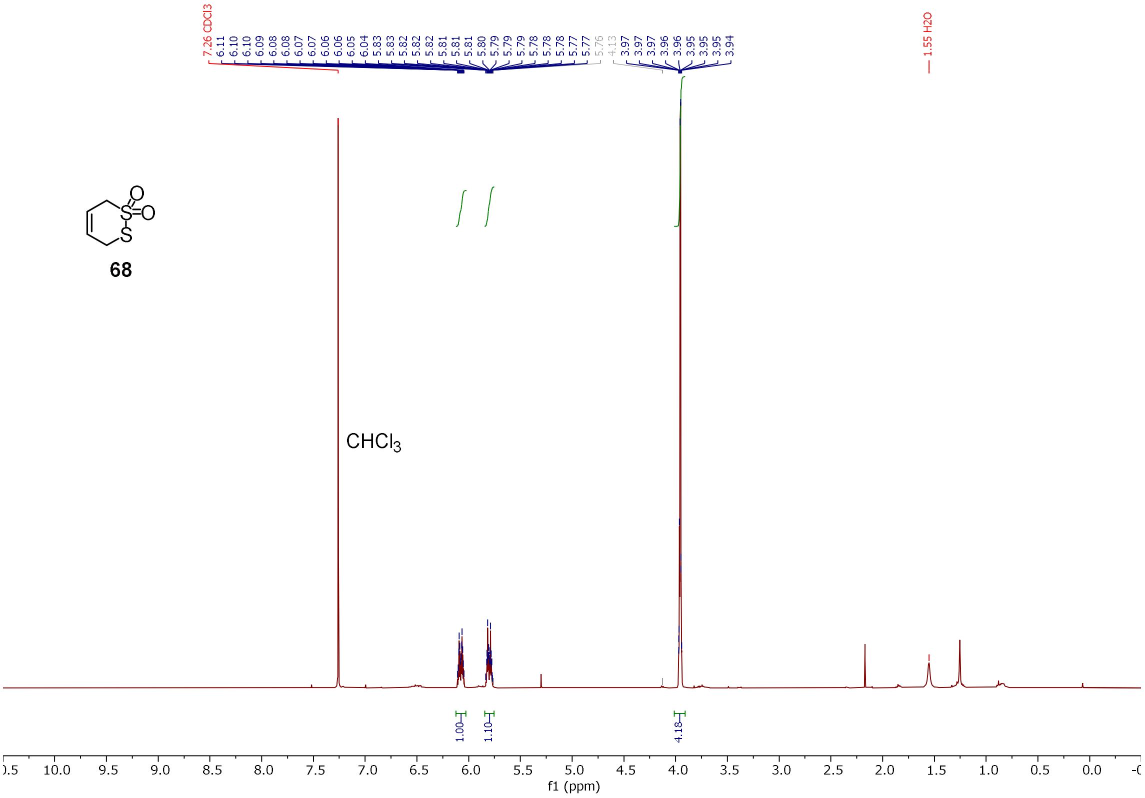

Figure S118. ^{1}H NMR (400 MHz, $CDCl_3$) spectrum of **68**.

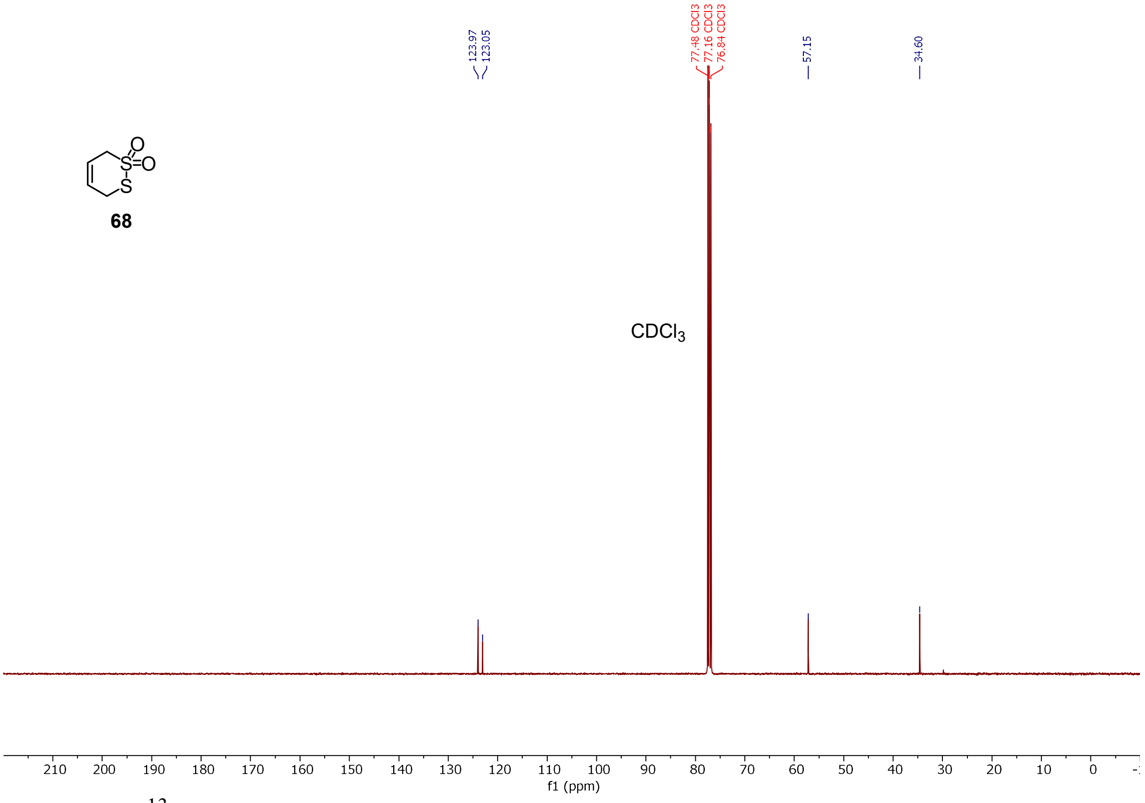

Figure S119. ^{13}C NMR (101 MHz, $CDCl_3$) spectrum of **68**.

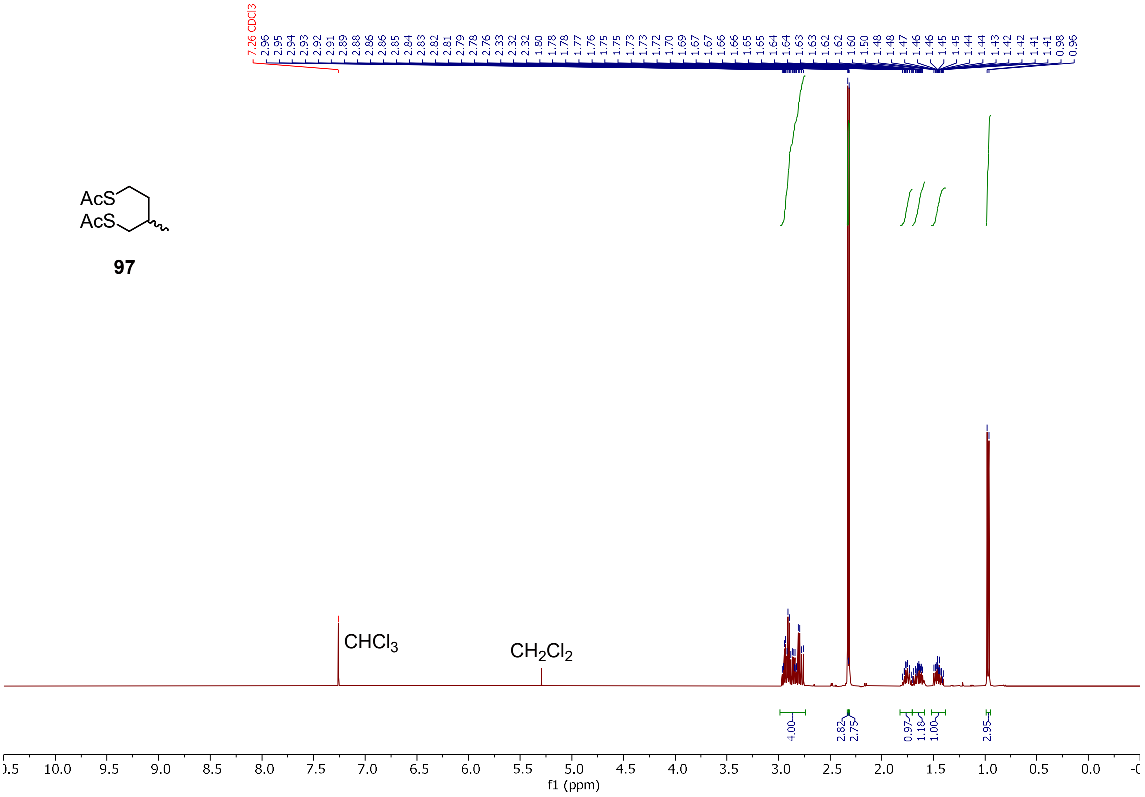

Figure S120. ^{1}H NMR (400 MHz, $CDCl_3$) spectrum of **97**.

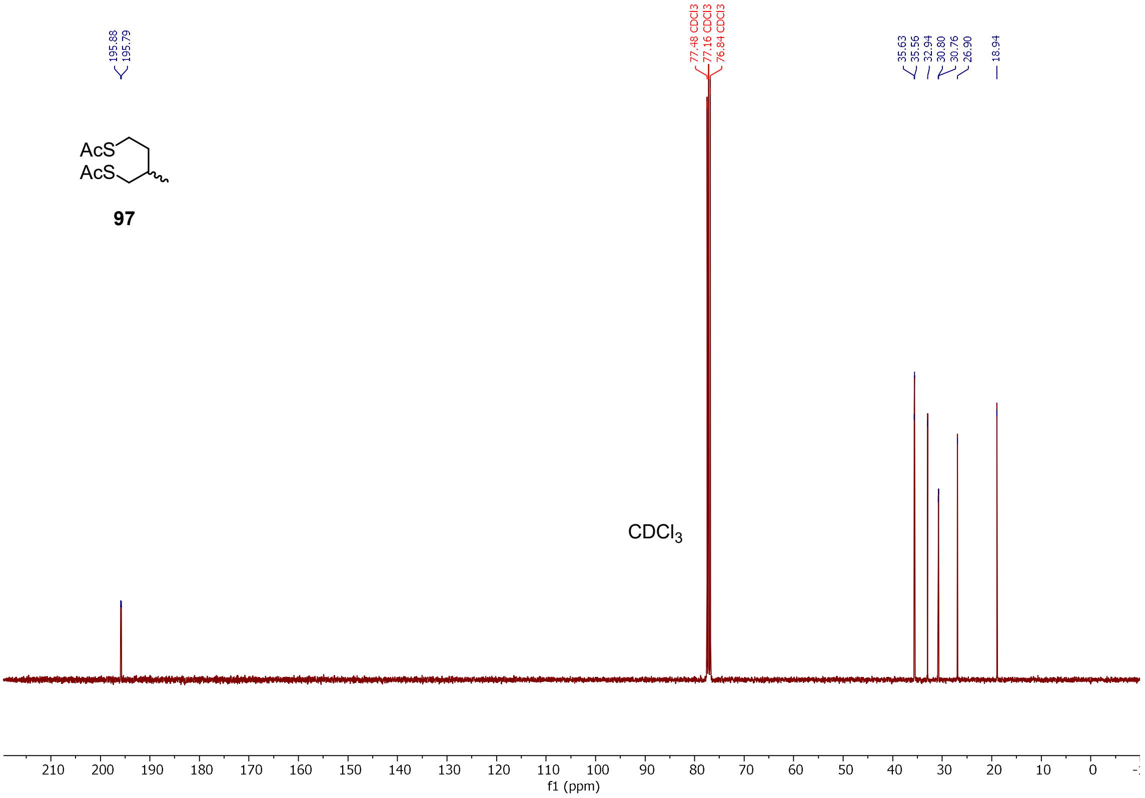

Figure S121. ^{13}C NMR (101 MHz, $CDCl_3$) spectrum of **97**.

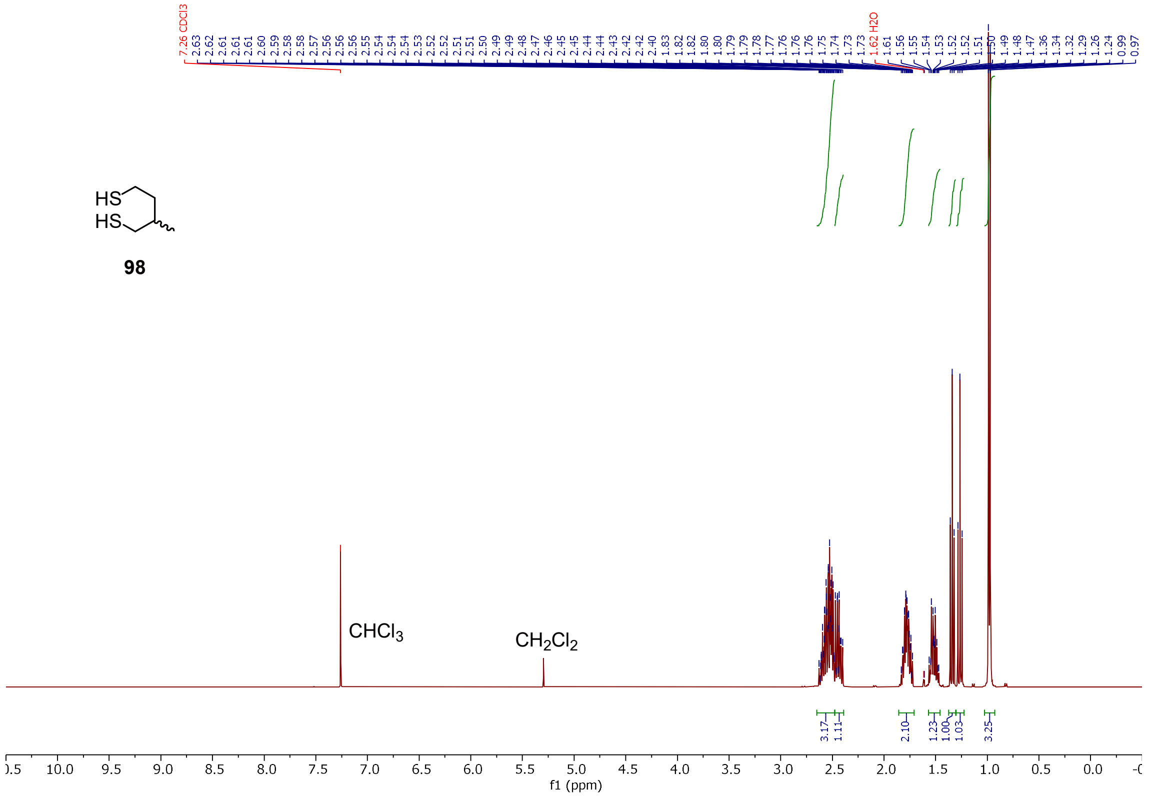

Figure S122. ^{1}H NMR (400 MHz, $CDCl_3$) spectrum of **98**.

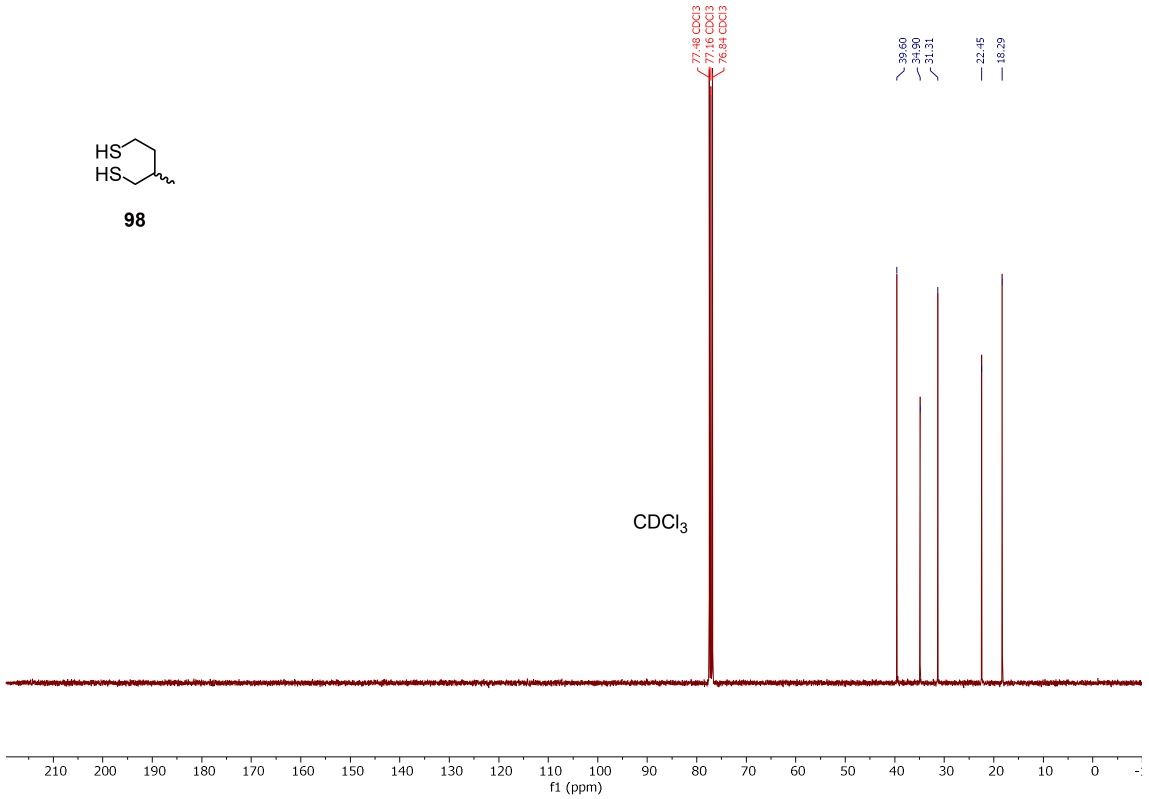

Figure S123. ^{13}C NMR (101 MHz, $CDCl_3$) spectrum of **98**.

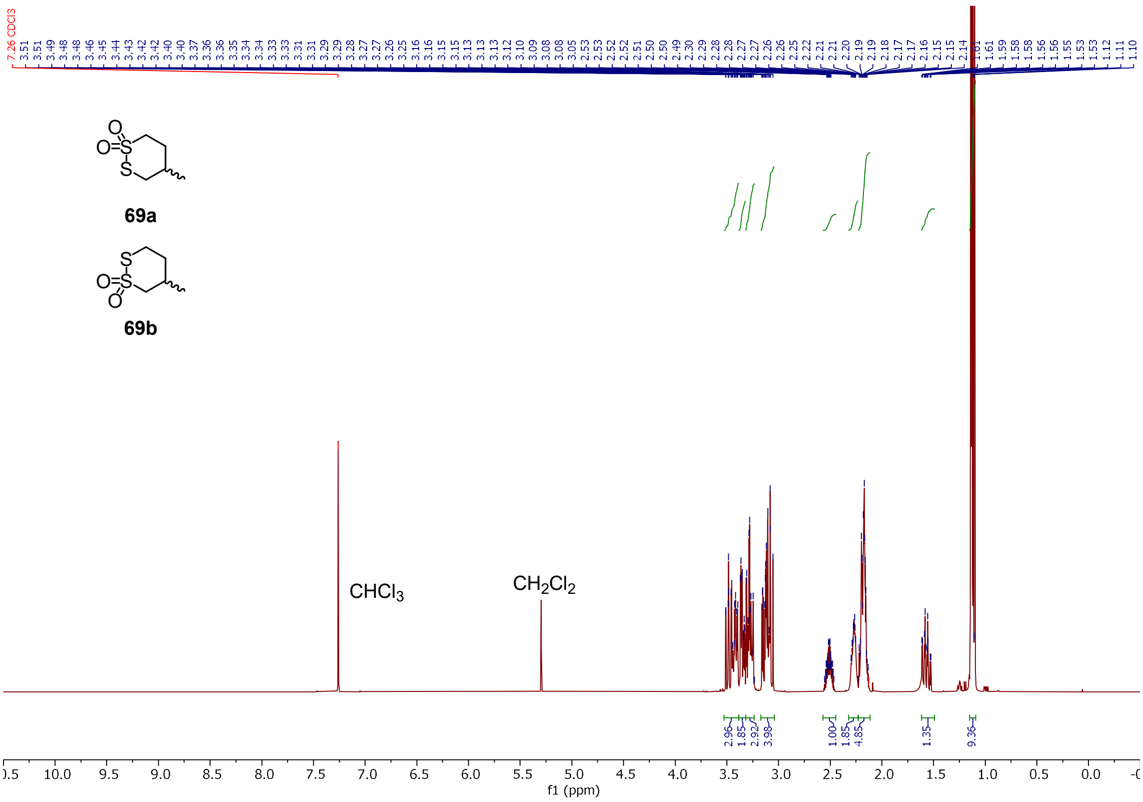

Figure S124. ^{1}H NMR (500 MHz, $CDCl_3$) spectrum of **69**.

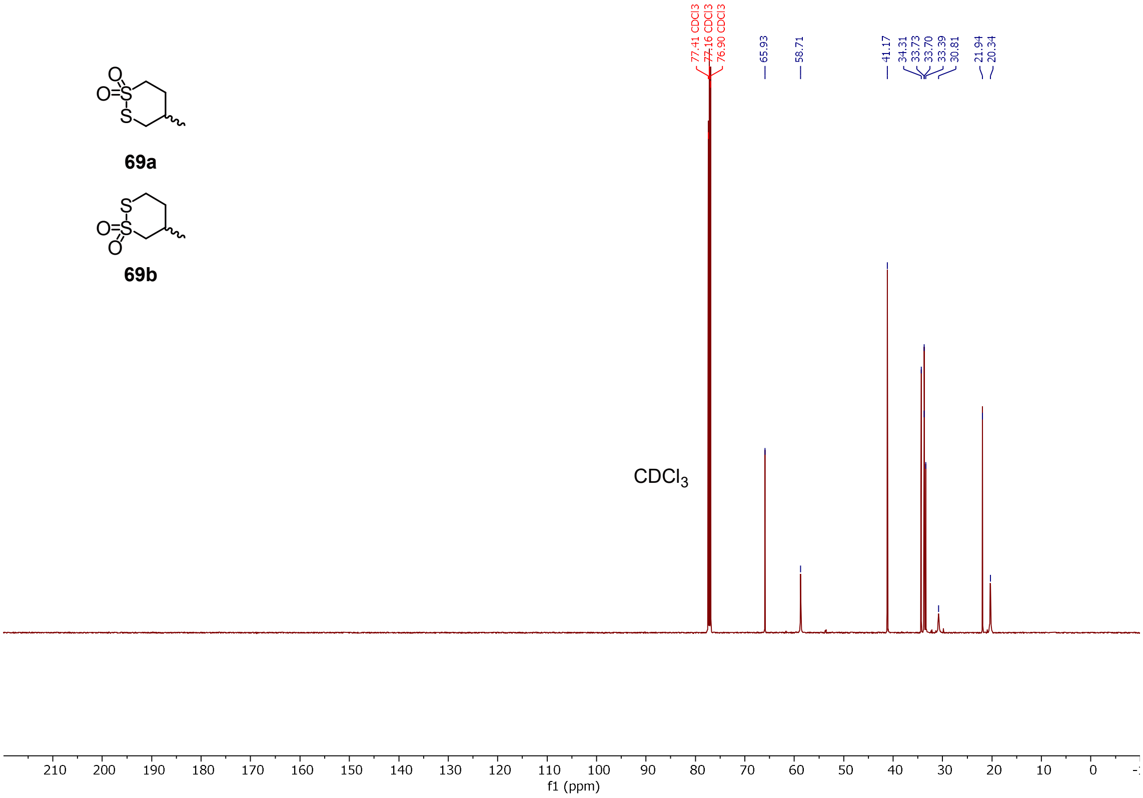

Figure S125. ^{13}C NMR (126 MHz, $CDCl_3$) spectrum of **69**.

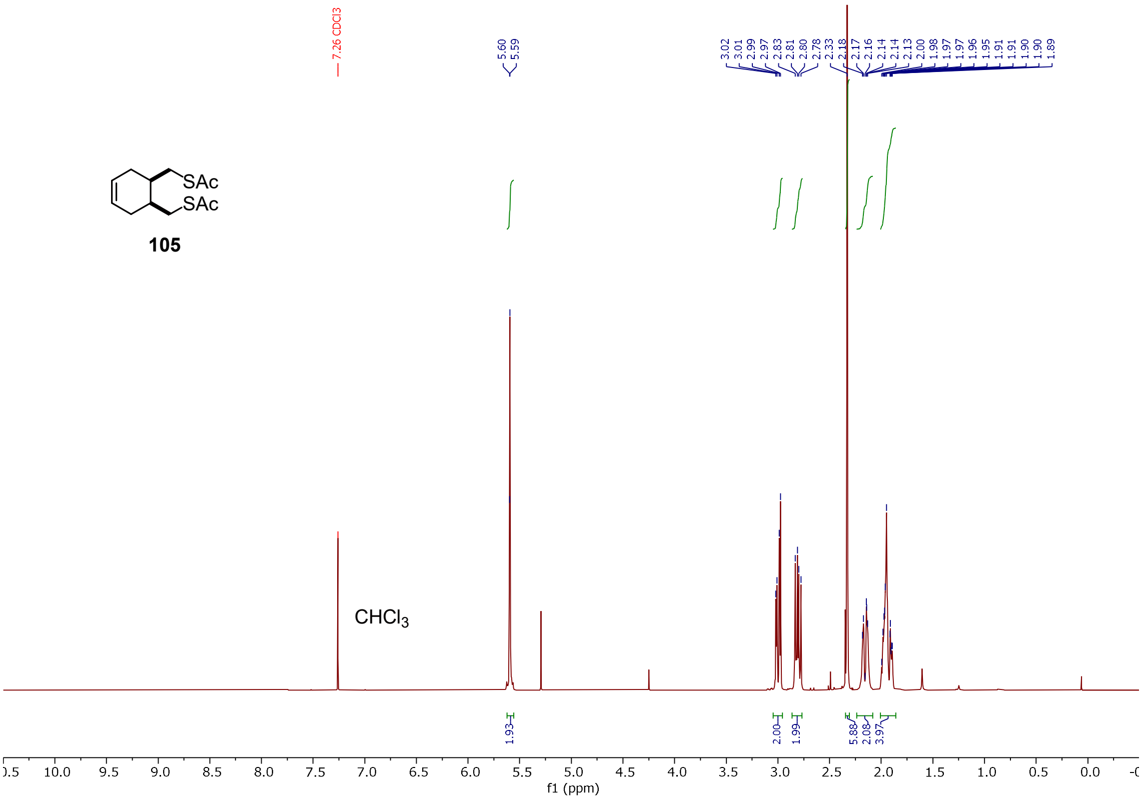

Figure S126. ^{1}H NMR (400 MHz, $CDCl_3$) spectrum of **105**.

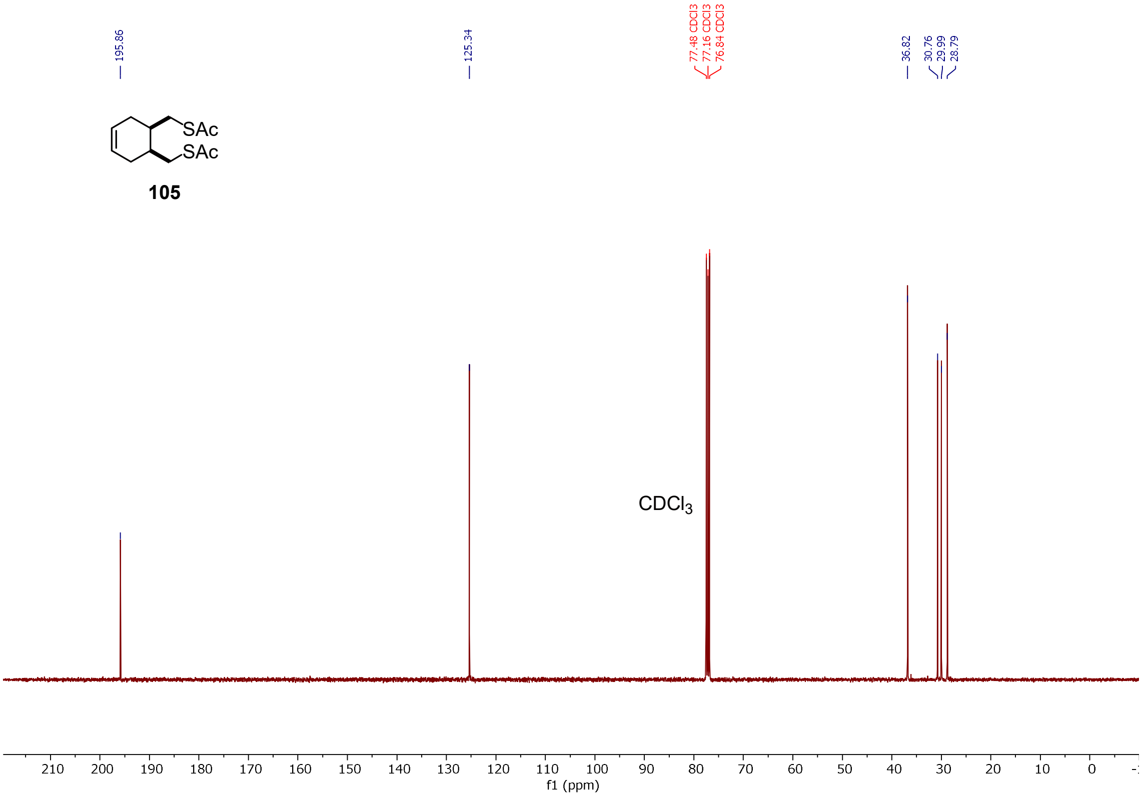

Figure S127. ^{13}C NMR (101 MHz, $CDCl_3$) spectrum of **105**.

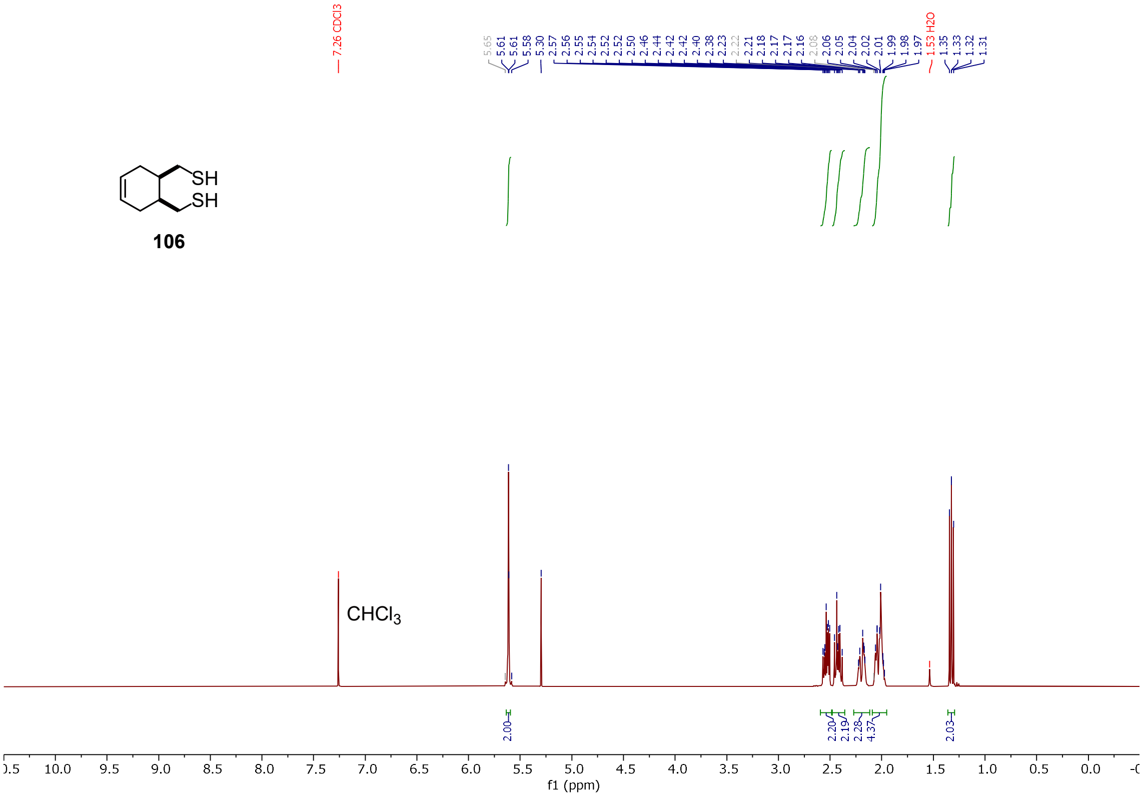

Figure S128. ^{1}H NMR (400 MHz, $CDCl_3$) spectrum of **106**.

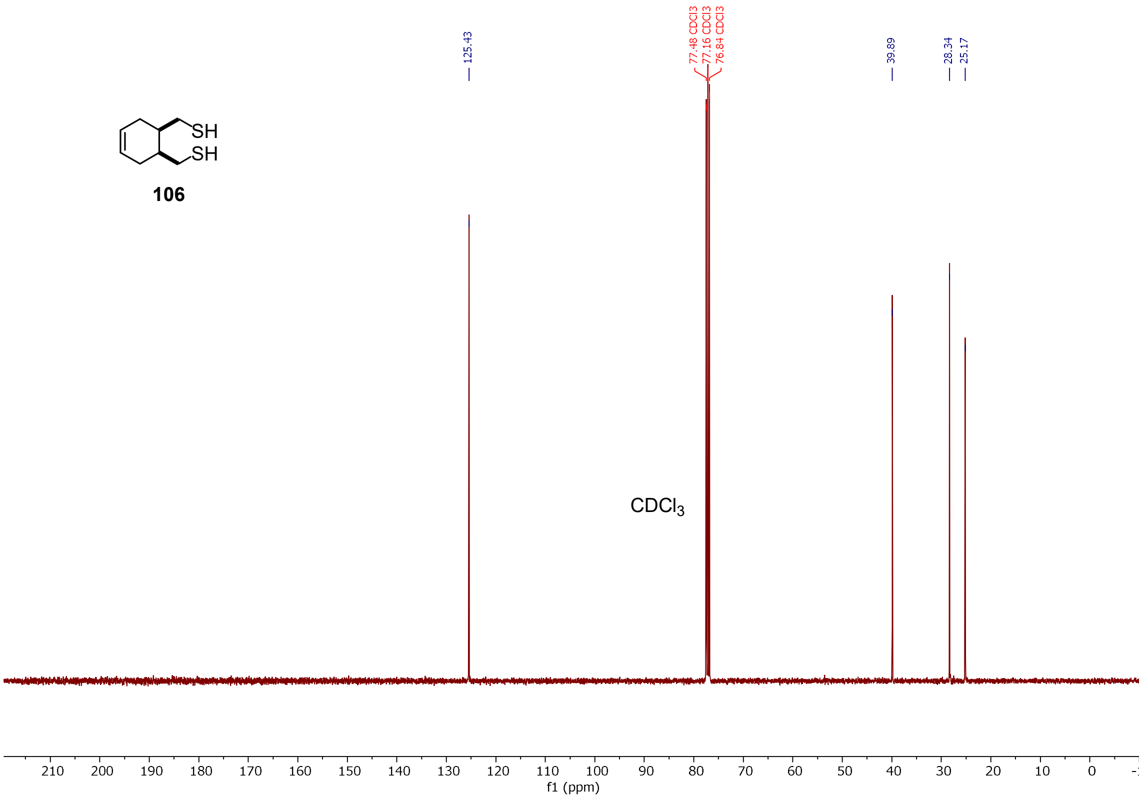

Figure S129. ^{13}C NMR (101 MHz, $CDCl_3$) spectrum of **106**.

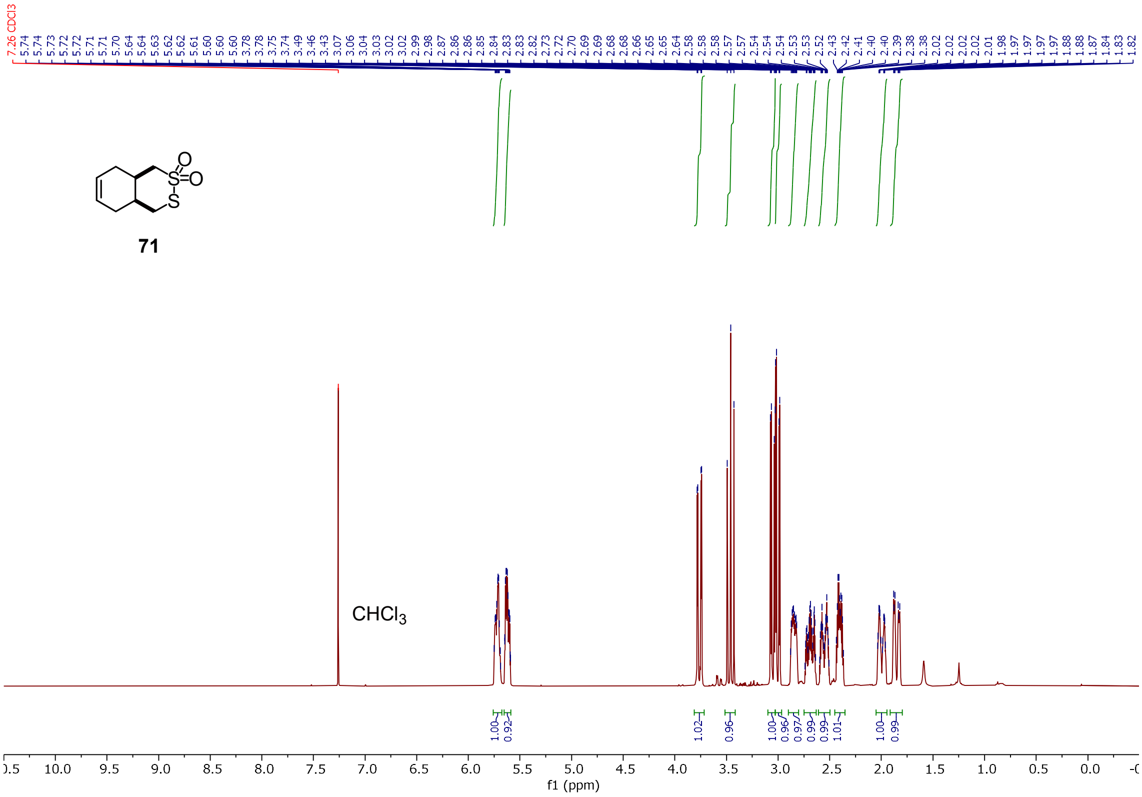

Figure S130. ^{1}H NMR (400 MHz, $CDCl_3$) spectrum of **71**.

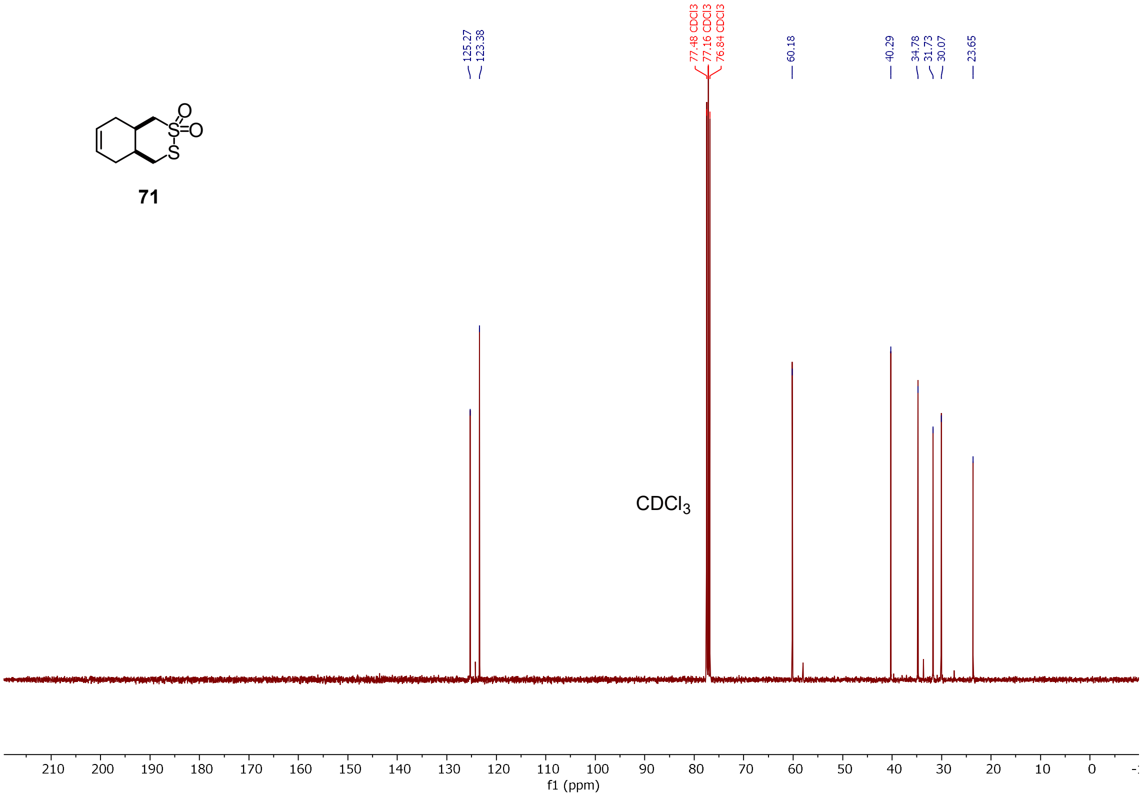

Figure S131. ^{13}C NMR (101 MHz, $CDCl_3$) spectrum of **71**.

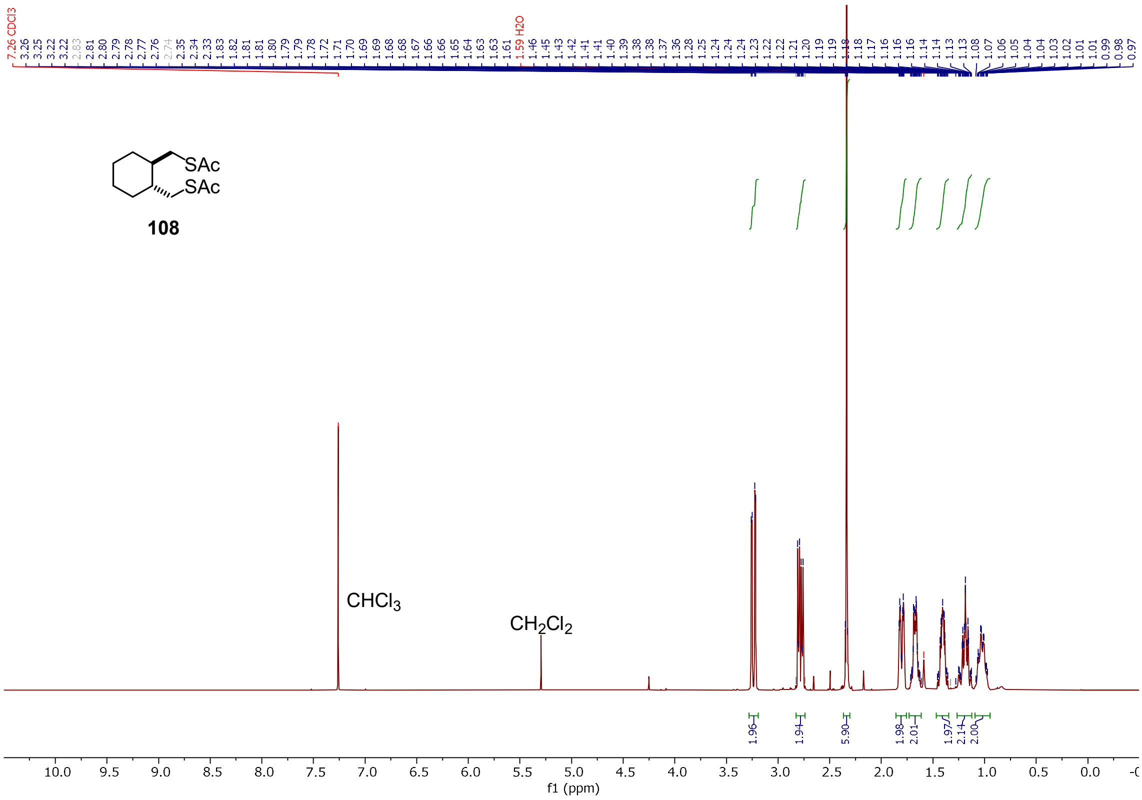

Figure S132. ^{1}H NMR (400 MHz, $CDCl_3$) spectrum of **108**.

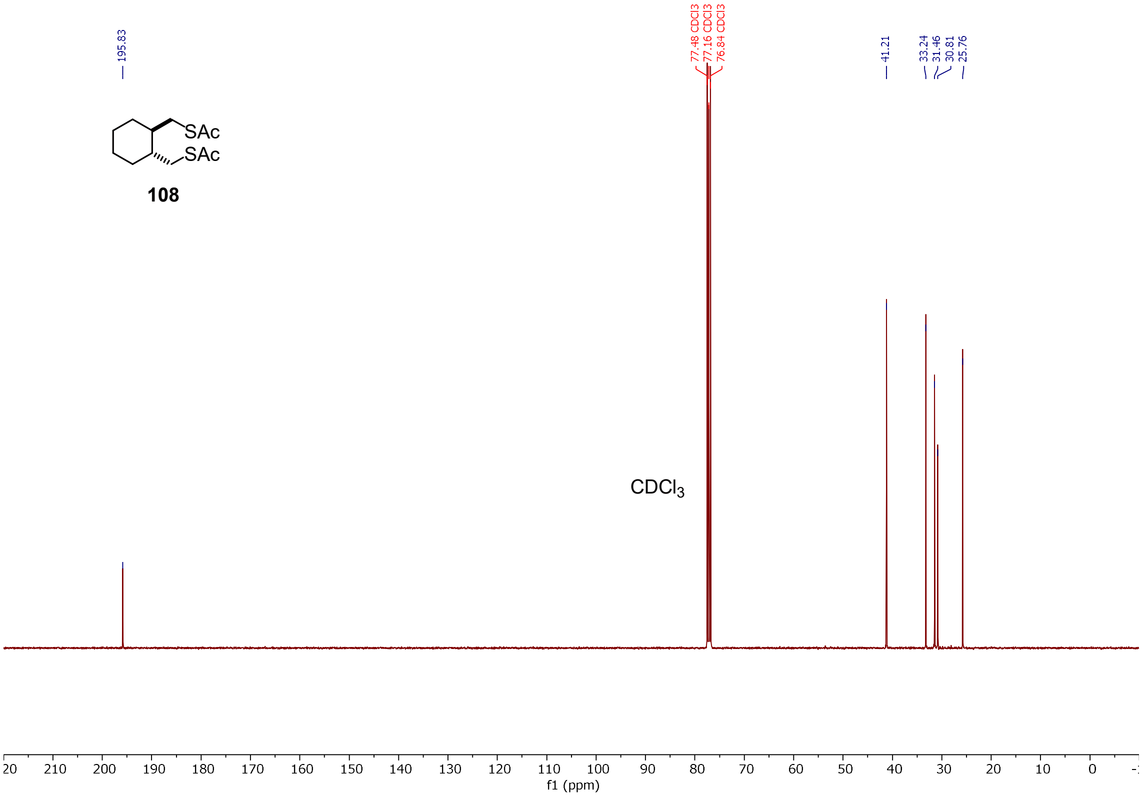

Figure S133. ^{13}C NMR (101 MHz, $CDCl_3$) spectrum of **108**.

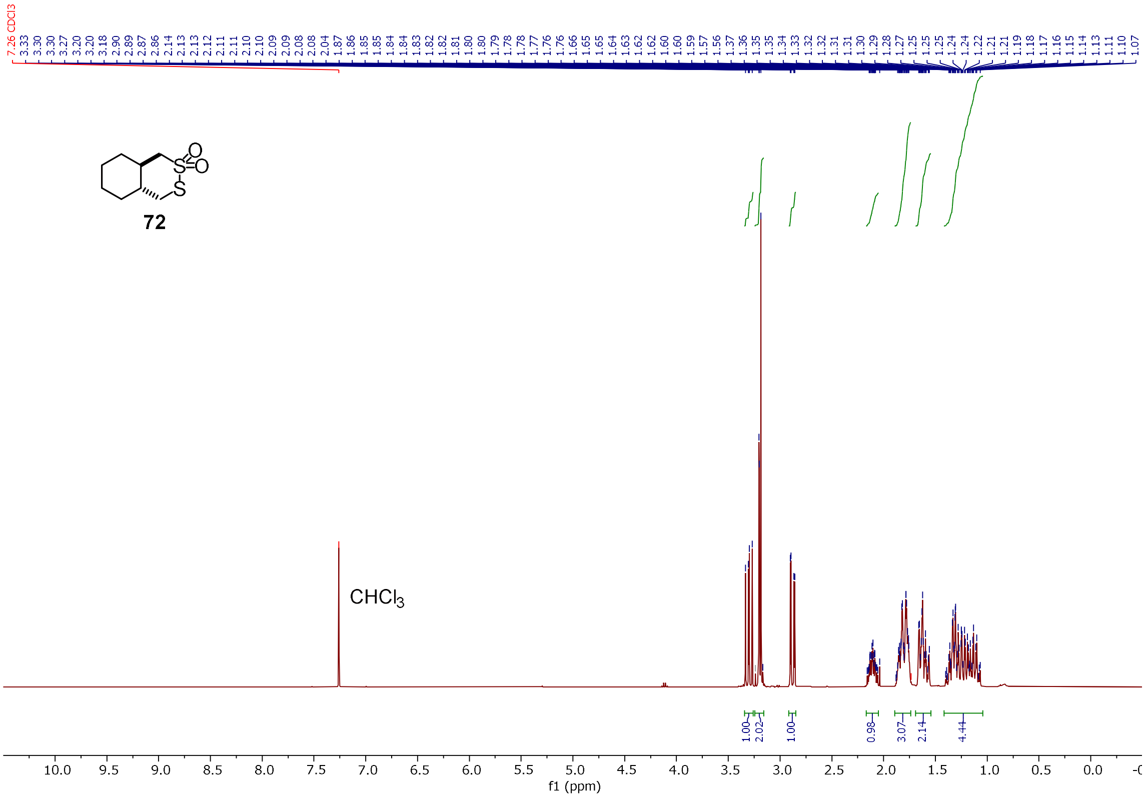

Figure S134. ^{1}H NMR (400 MHz, $CDCl_3$) spectrum of **72**.

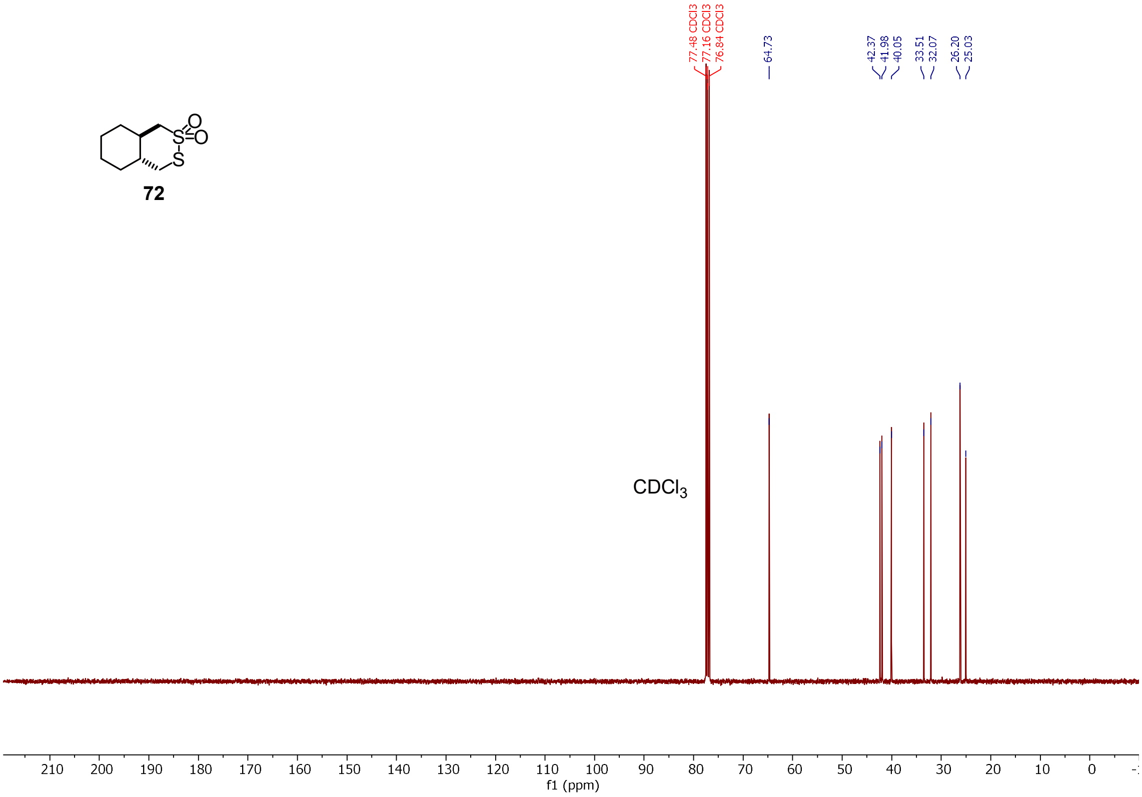

Figure S135. ^{13}C NMR (101 MHz, $CDCl_3$) spectrum of **72**.

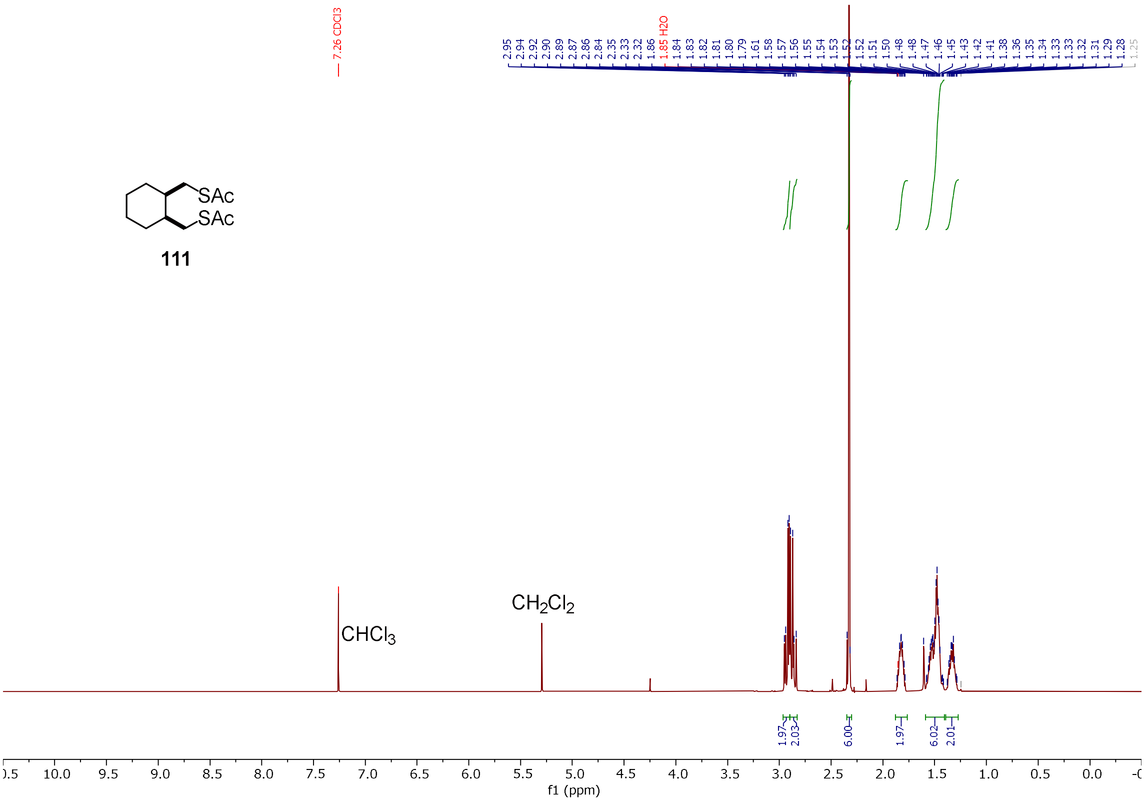

Figure S136. ^{1}H NMR (400 MHz, $CDCl_3$) spectrum of **111**.

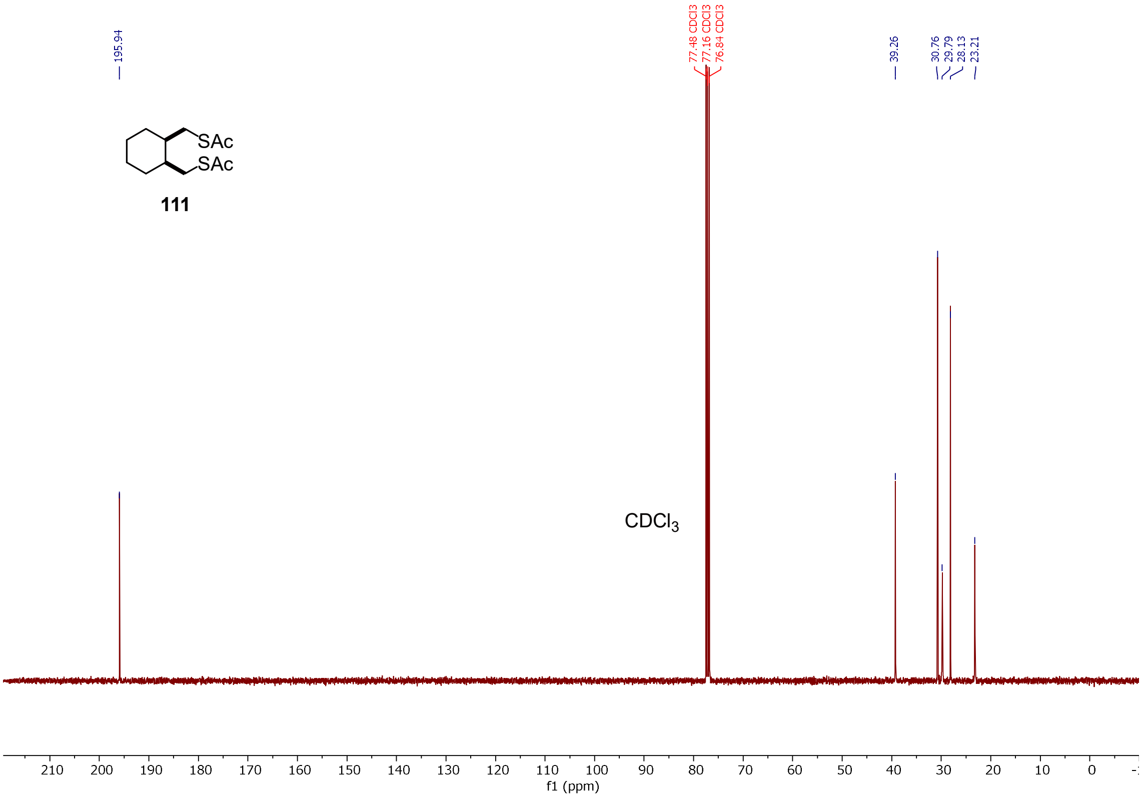

Figure S137. ^{13}C NMR (101 MHz, $CDCl_3$) spectrum of **111**.

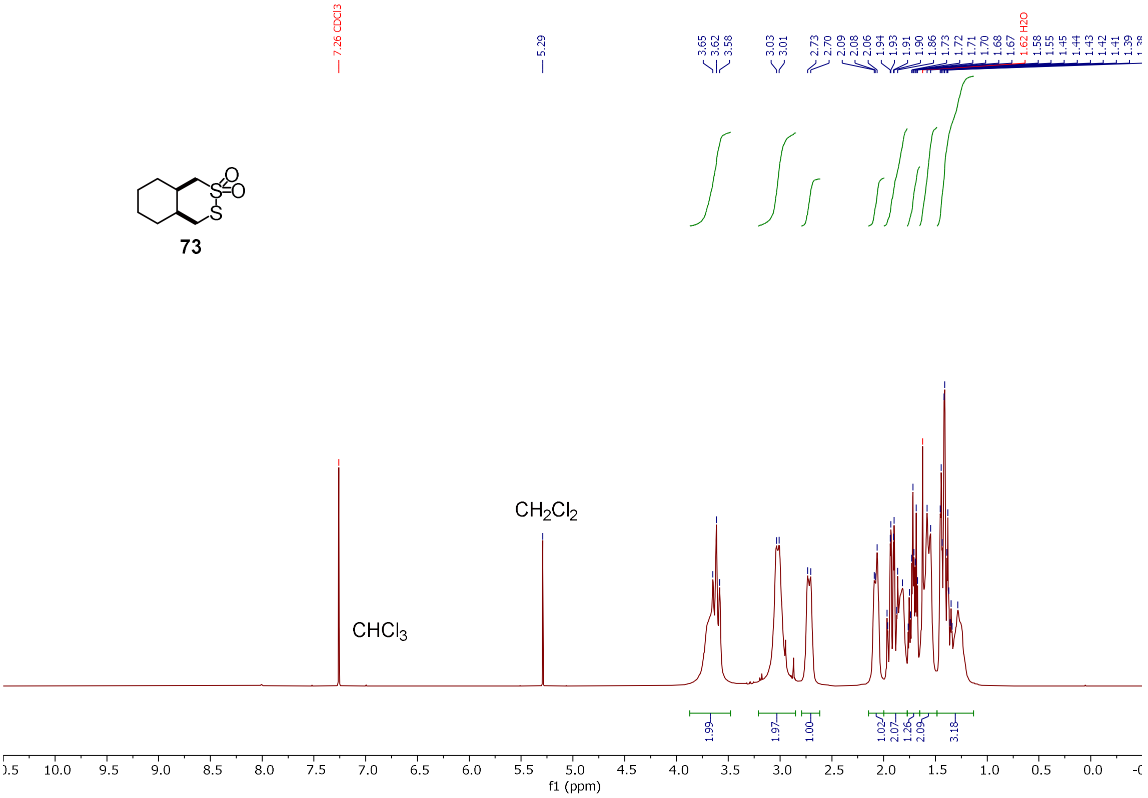

Figure S138. ^{1}H NMR (400 MHz, $CDCl_3$) spectrum of **73**.

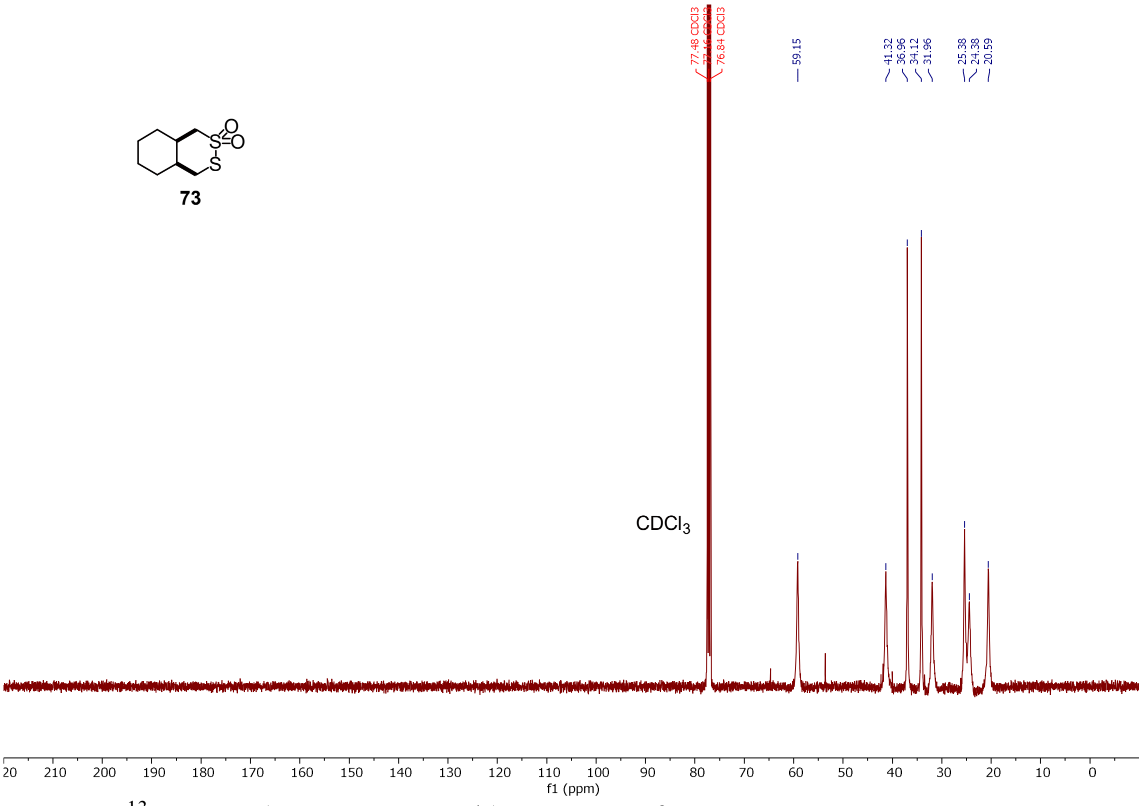

Figure S139. ^{13}C NMR (101 MHz, $CDCl_3$) spectrum of **73**.

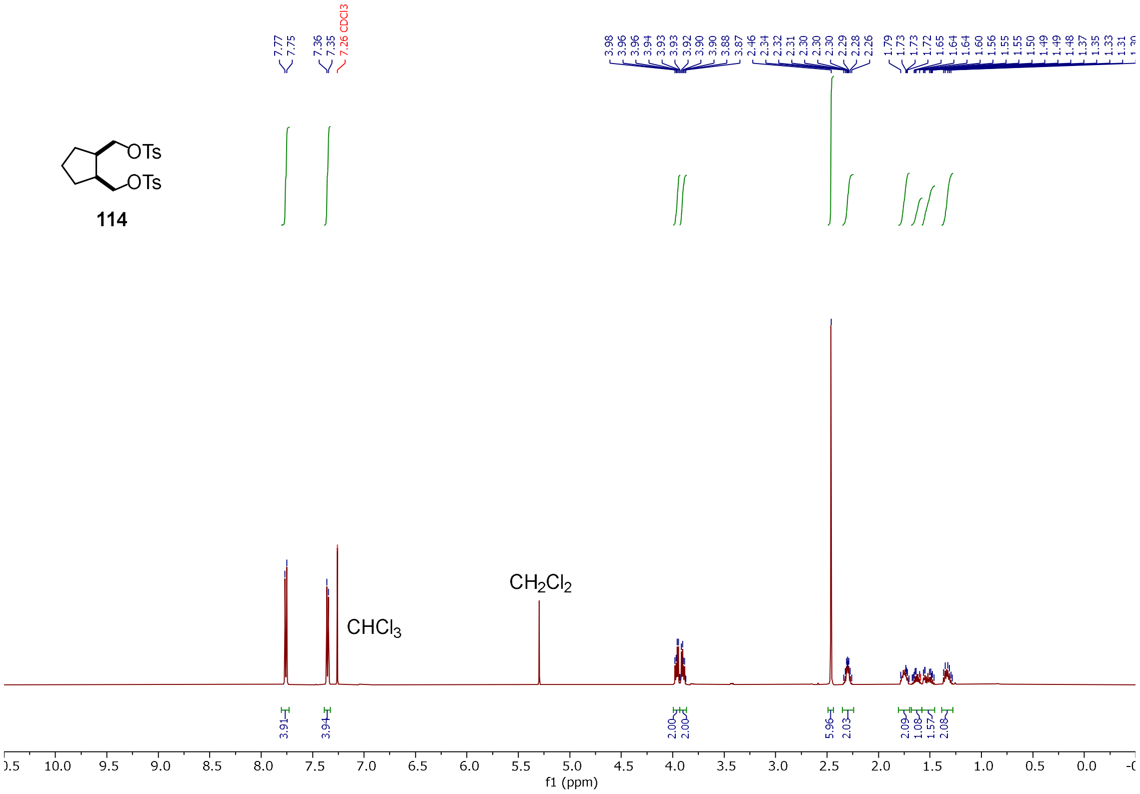

Figure S140. ^{1}H NMR (500 MHz, $CDCl_3$) spectrum of **114**.

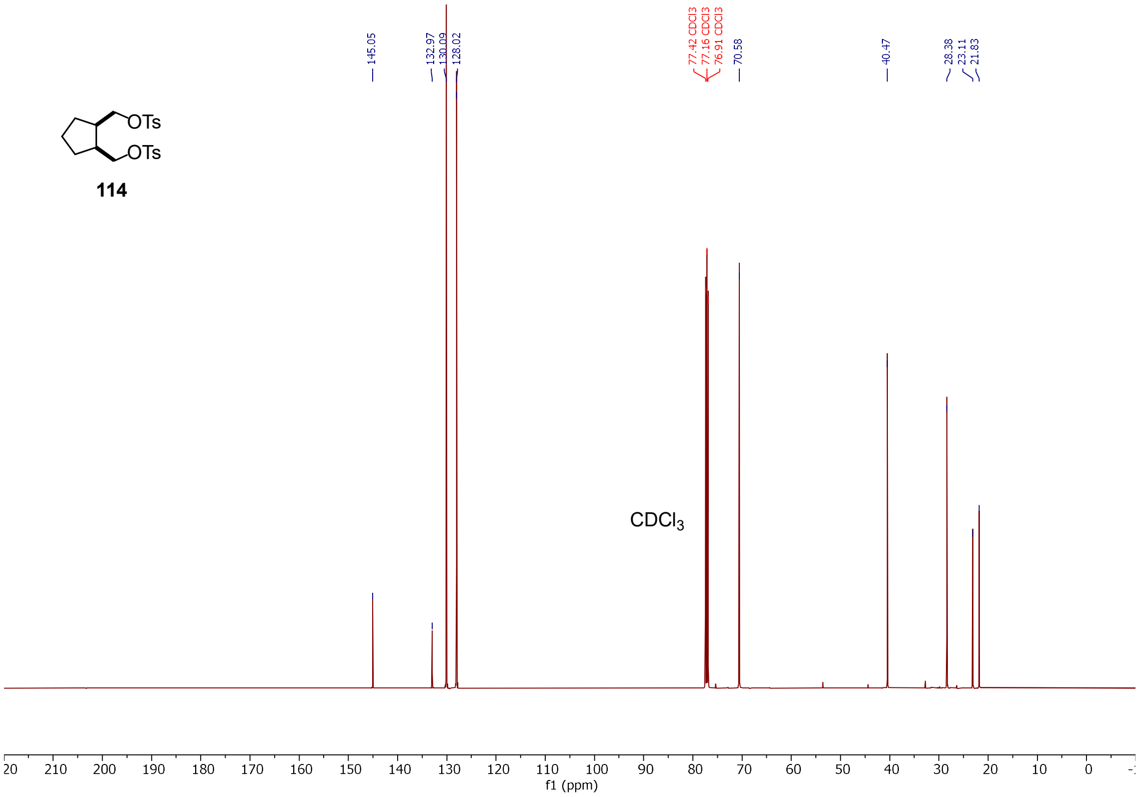

Figure S141. ^{13}C NMR (126 MHz, $CDCl_3$) spectrum of **114**.

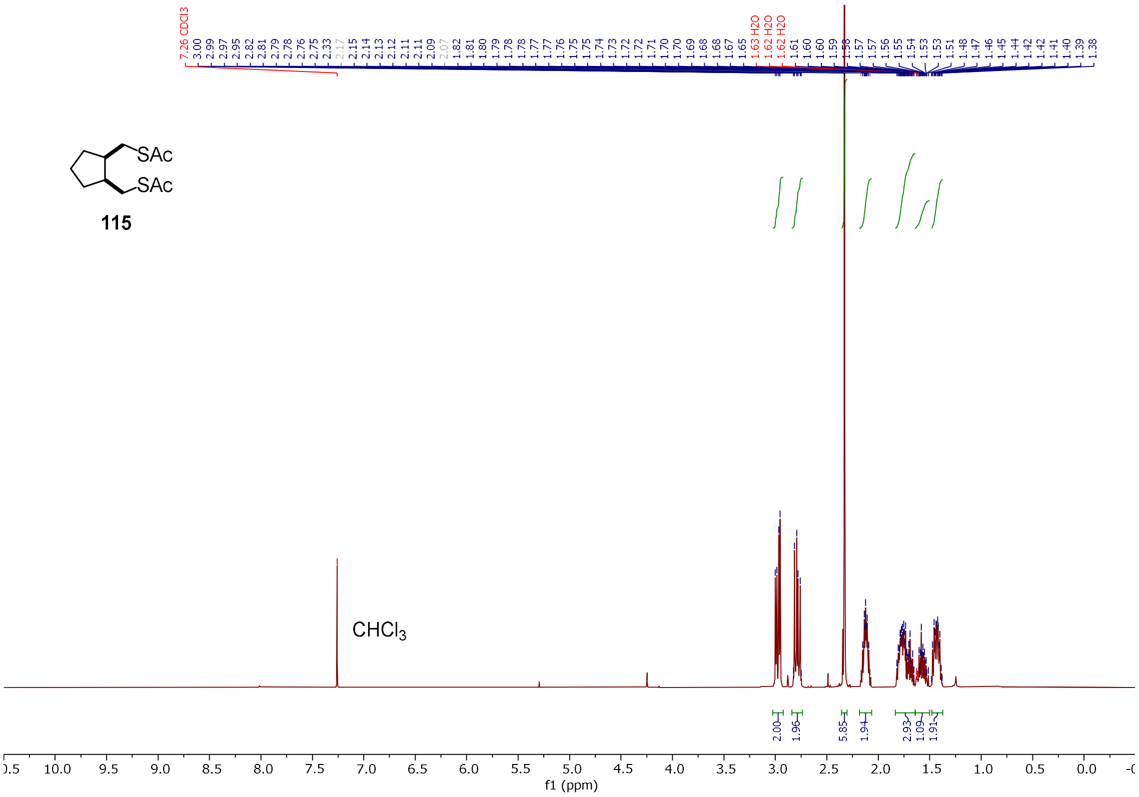

Figure S142. ^{1}H NMR (400 MHz, $CDCl_3$) spectrum of **115**.

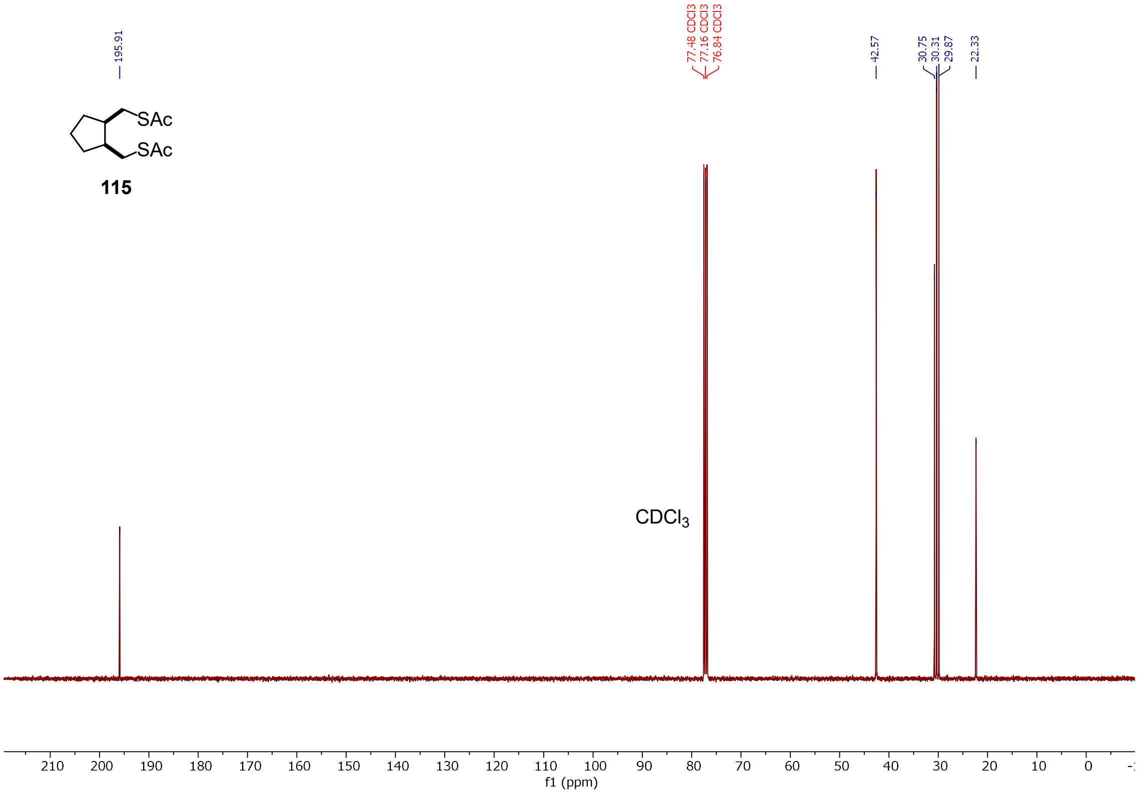

Figure S143. ^{13}C NMR (101 MHz, $CDCl_3$) spectrum of **115**.

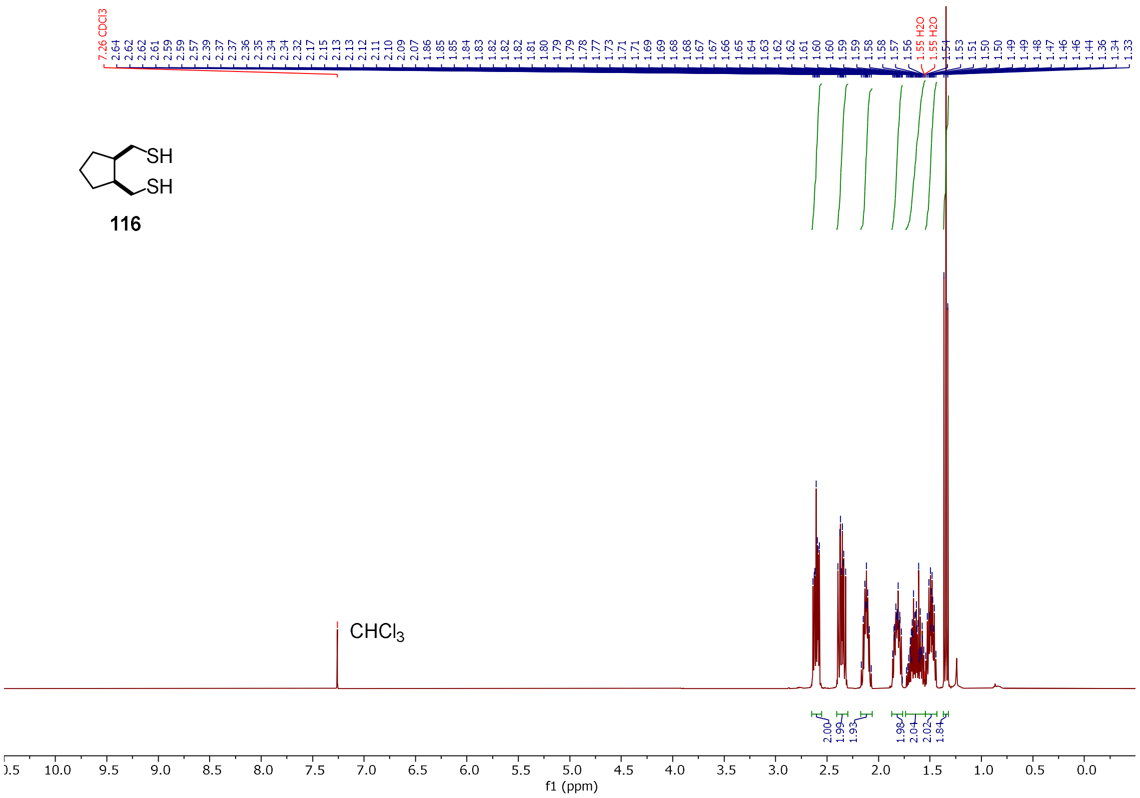

Figure S144. ^{1}H NMR (400 MHz, $CDCl_3$) spectrum of **116**.

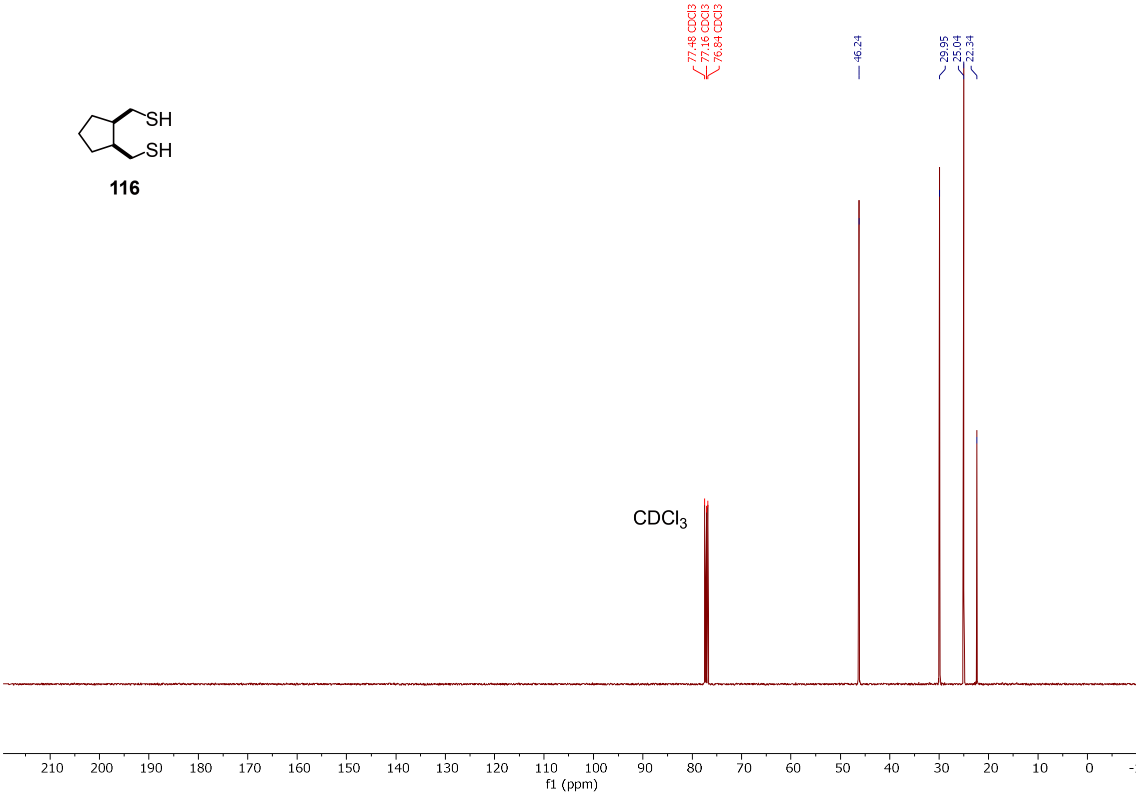

Figure S145. ^{13}C NMR (101 MHz, $CDCl_3$) spectrum of **116**.

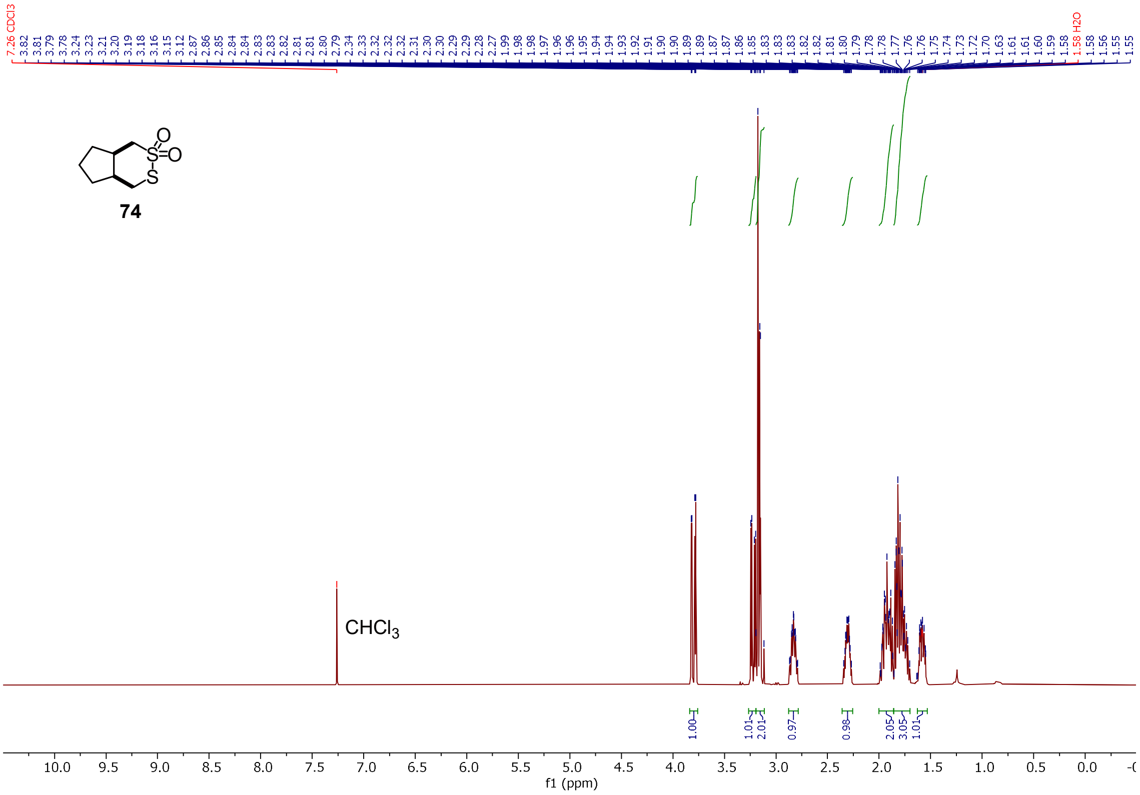

Figure S146. ^{1}H NMR (400 MHz, $CDCl_3$) spectrum of **74**.

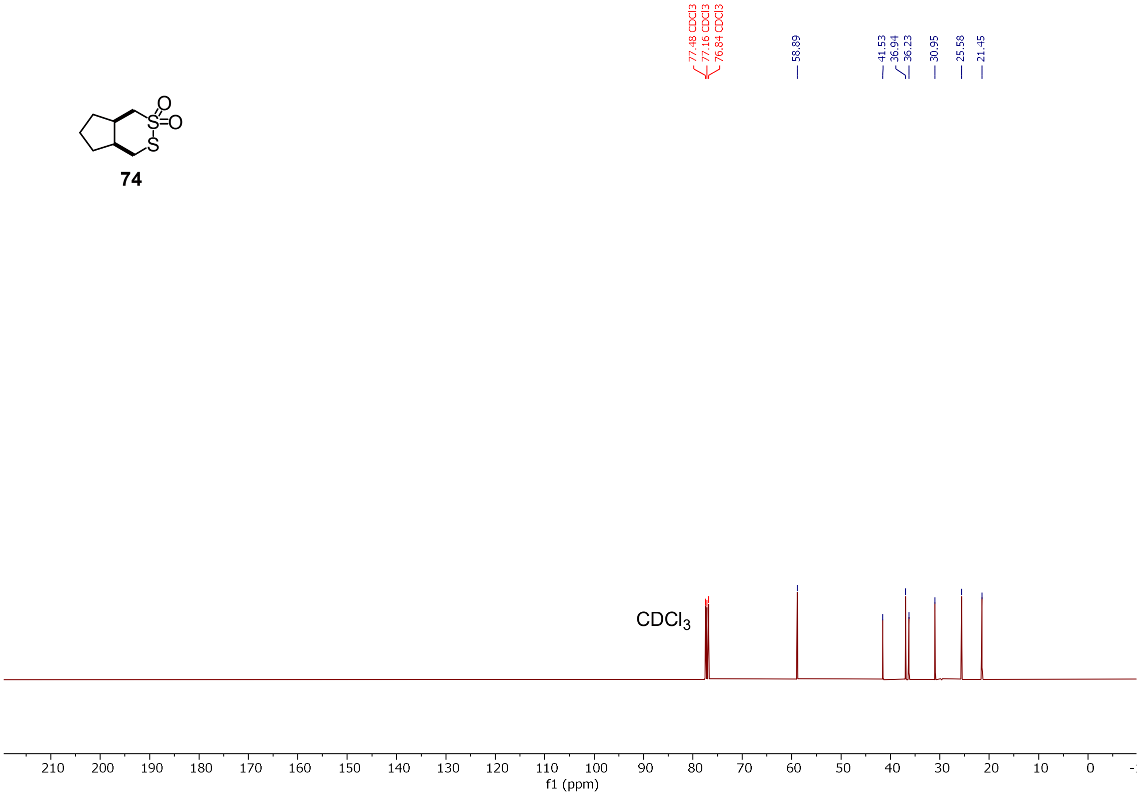

Figure S147. ^{13}C NMR (101 MHz, $CDCl_3$) spectrum of **74**.

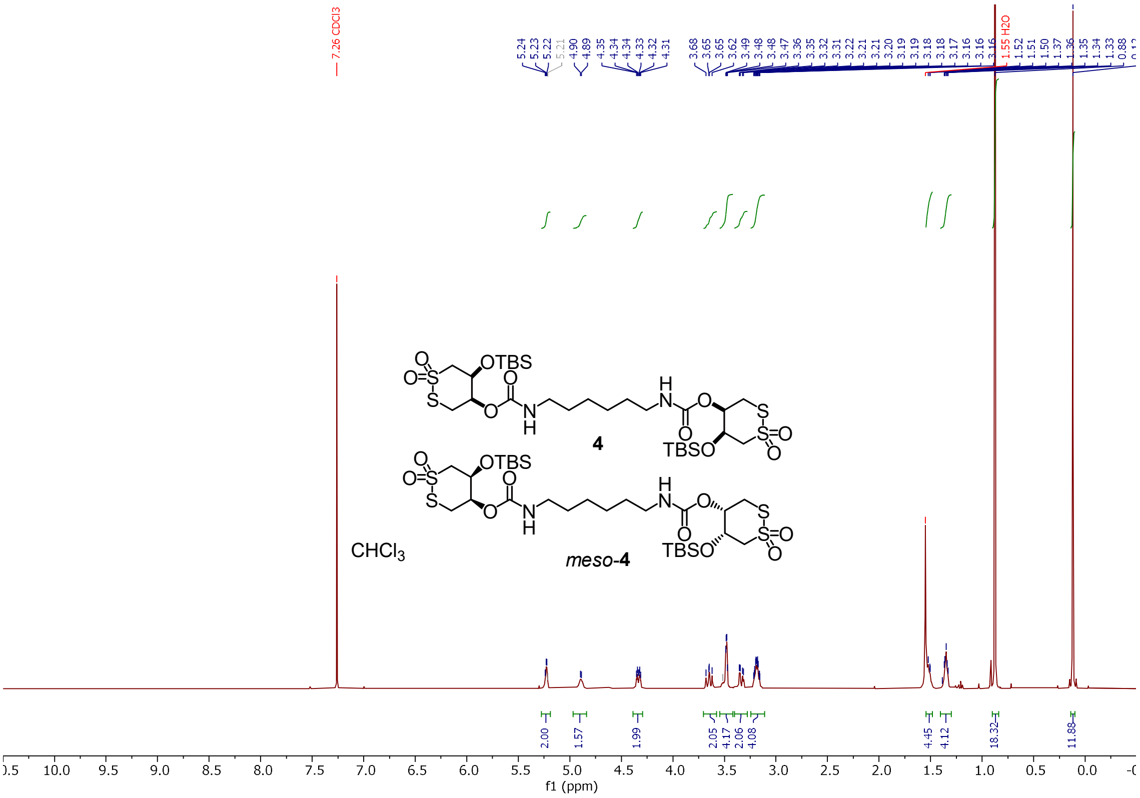

Figure S148. ^{1}H NMR (400 MHz, $CDCl_3$) spectrum of **4** and ***meso*-4**.

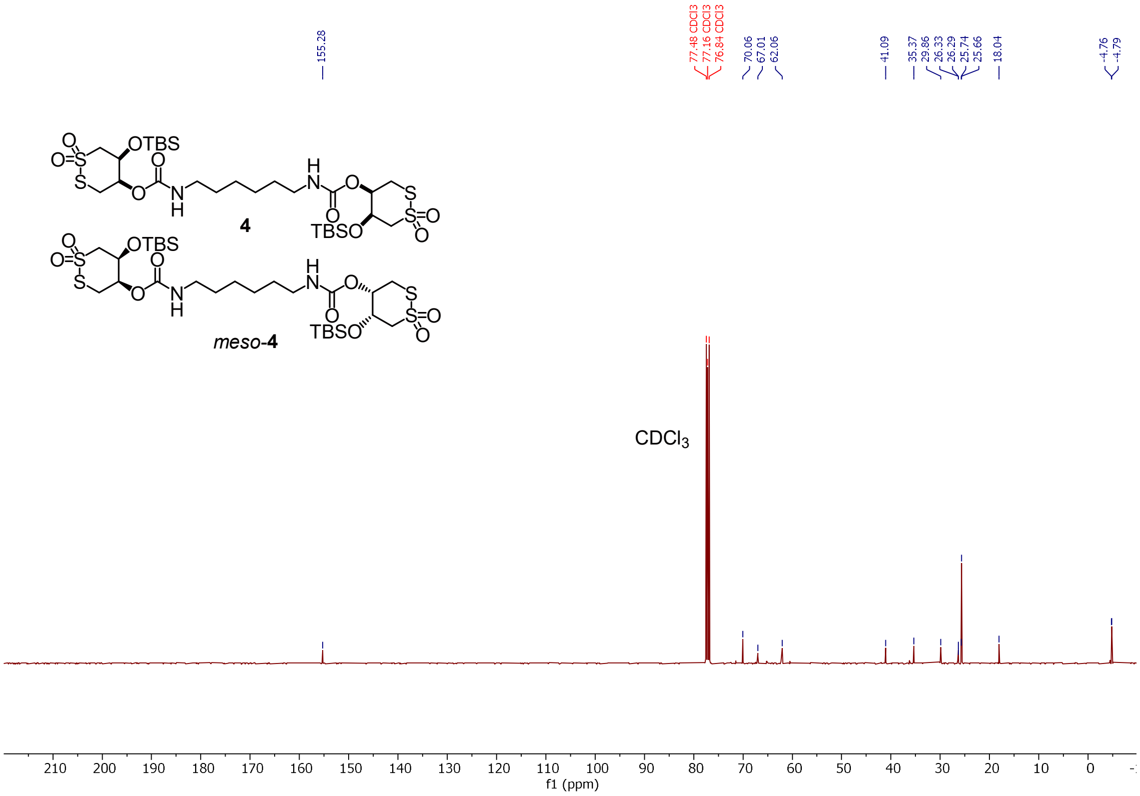

Figure S149. ^{13}C NMR (101 MHz, $CDCl_3$) spectrum of **4** and ***meso*-4**.

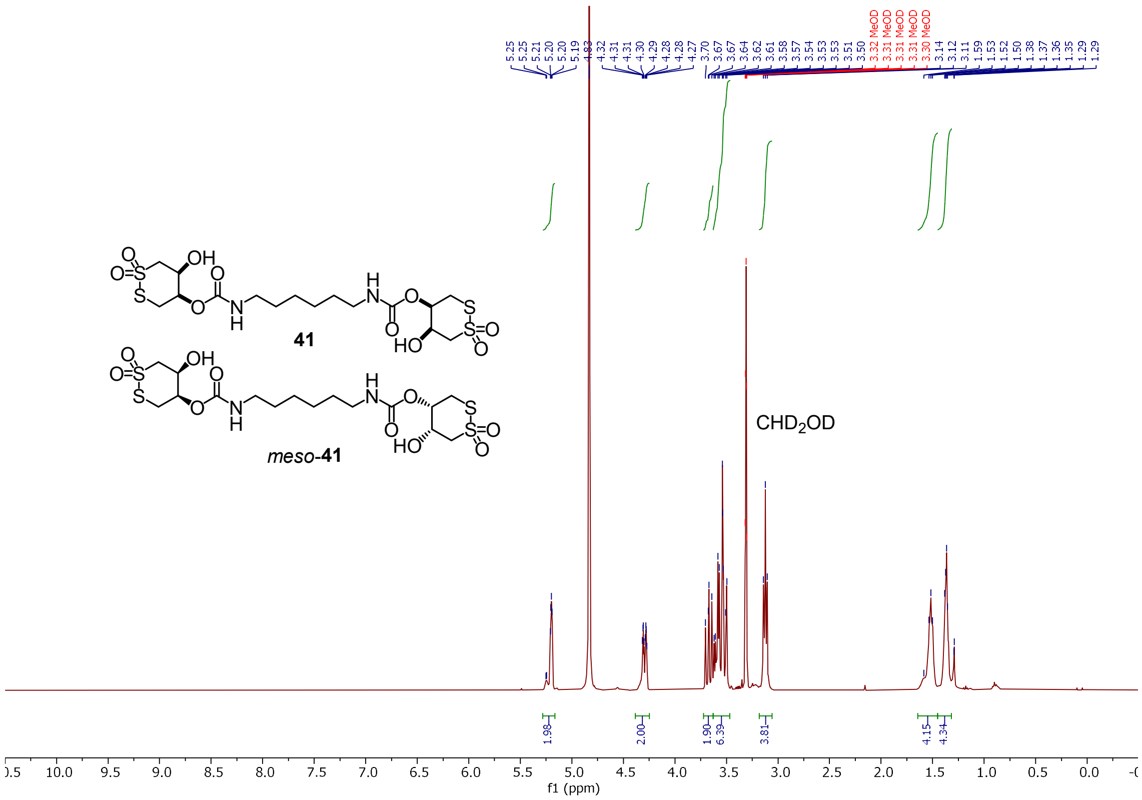

Figure S150. ^{1}H NMR (400 MHz, CD_3OD) spectrum of **41** and ***meso*-41**.

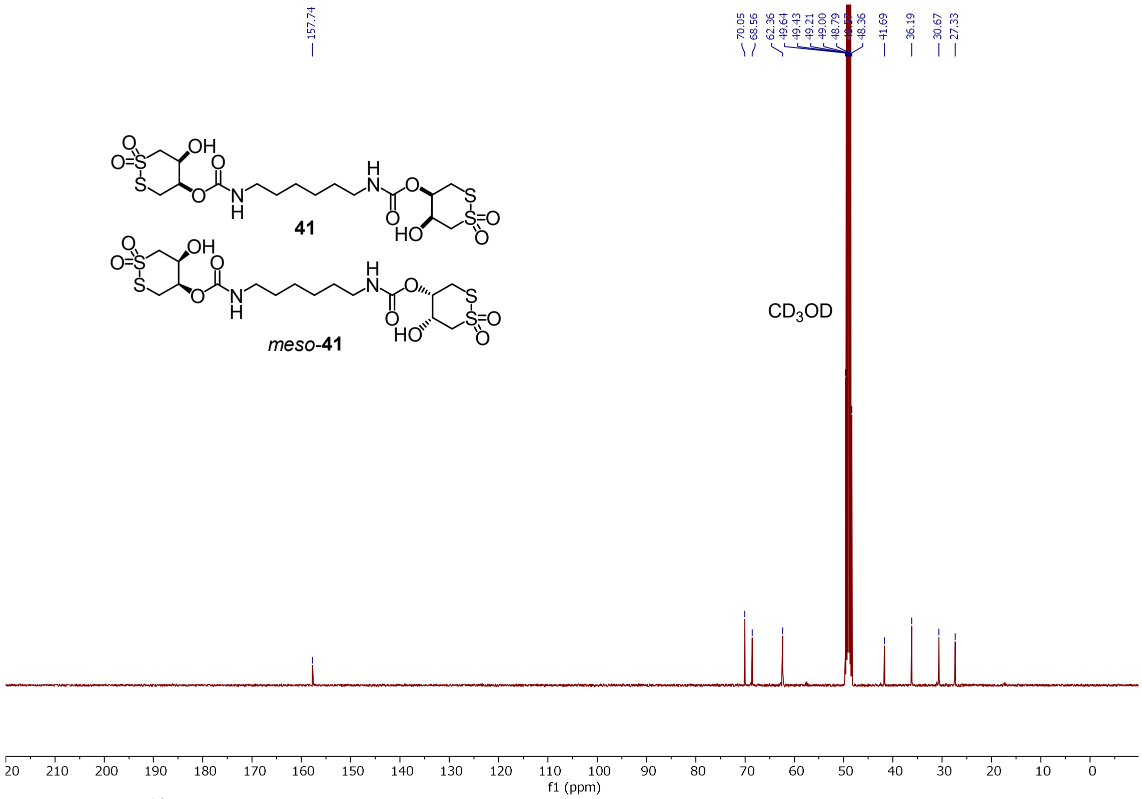

Figure S151. ^{13}C NMR (101 MHz, CD_3OD) spectrum of **41** and ***meso*-41**.

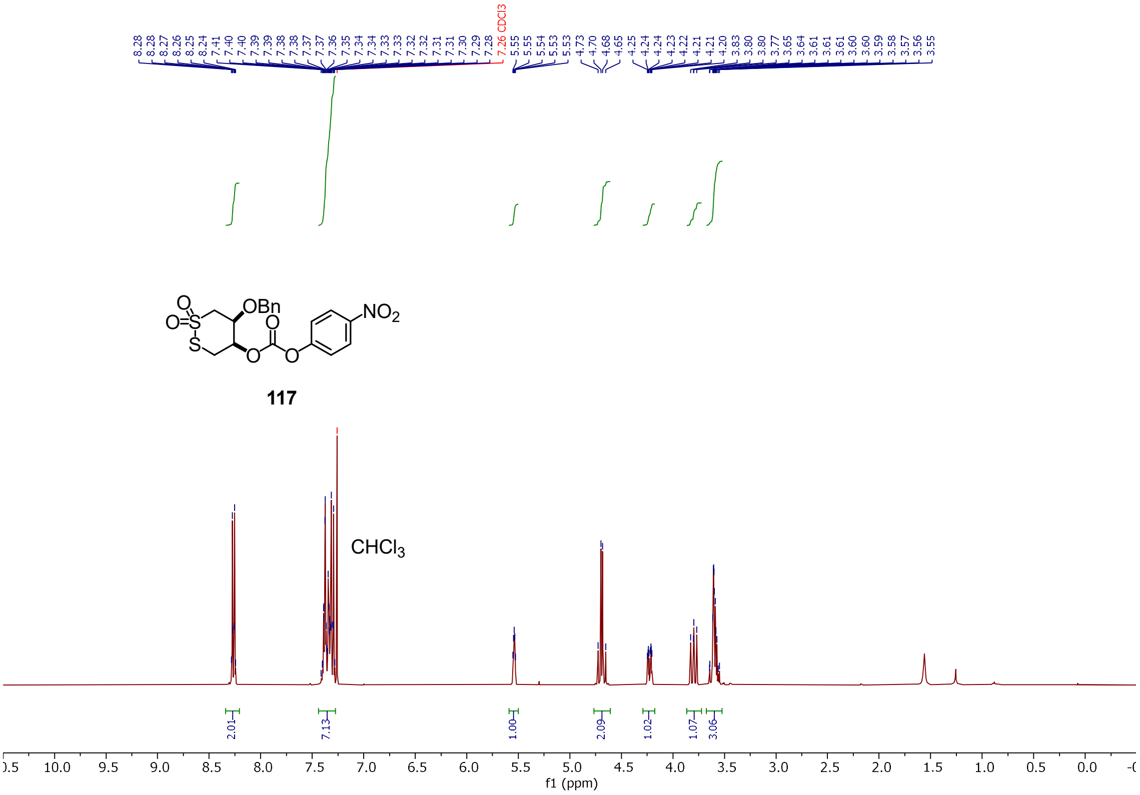

Figure S152. ^{1}H NMR (400 MHz, $CDCl_3$) spectrum of **117**.

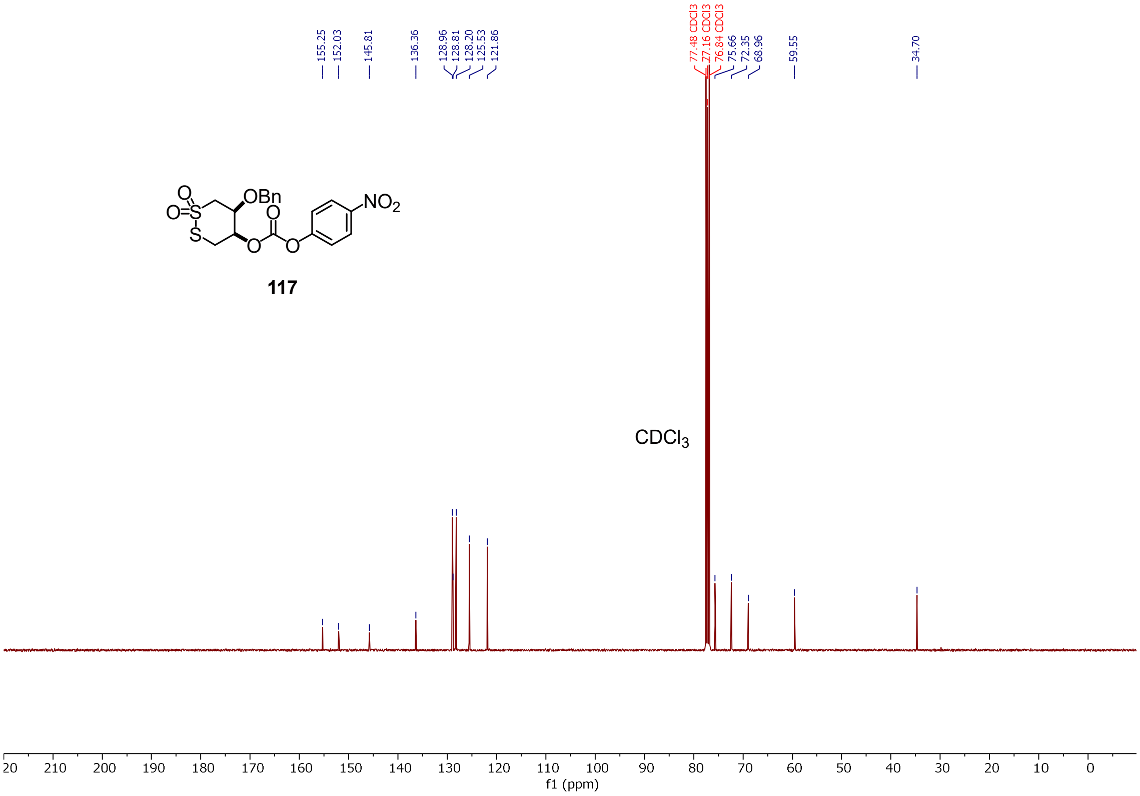

Figure S153. ^{13}C NMR (101 MHz, $CDCl_3$) spectrum of **117**.

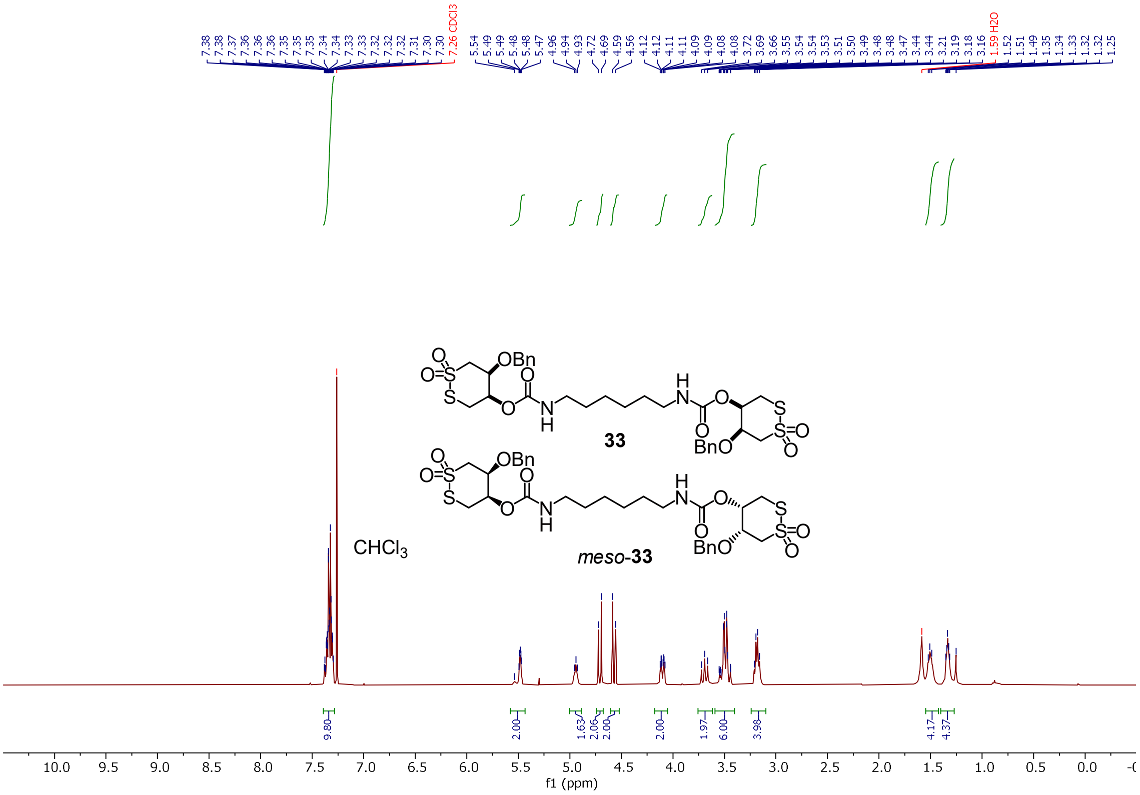

Figure S154. ^{1}H NMR (400 MHz, $CDCl_3$) spectrum of **33** and ***meso*-33**.

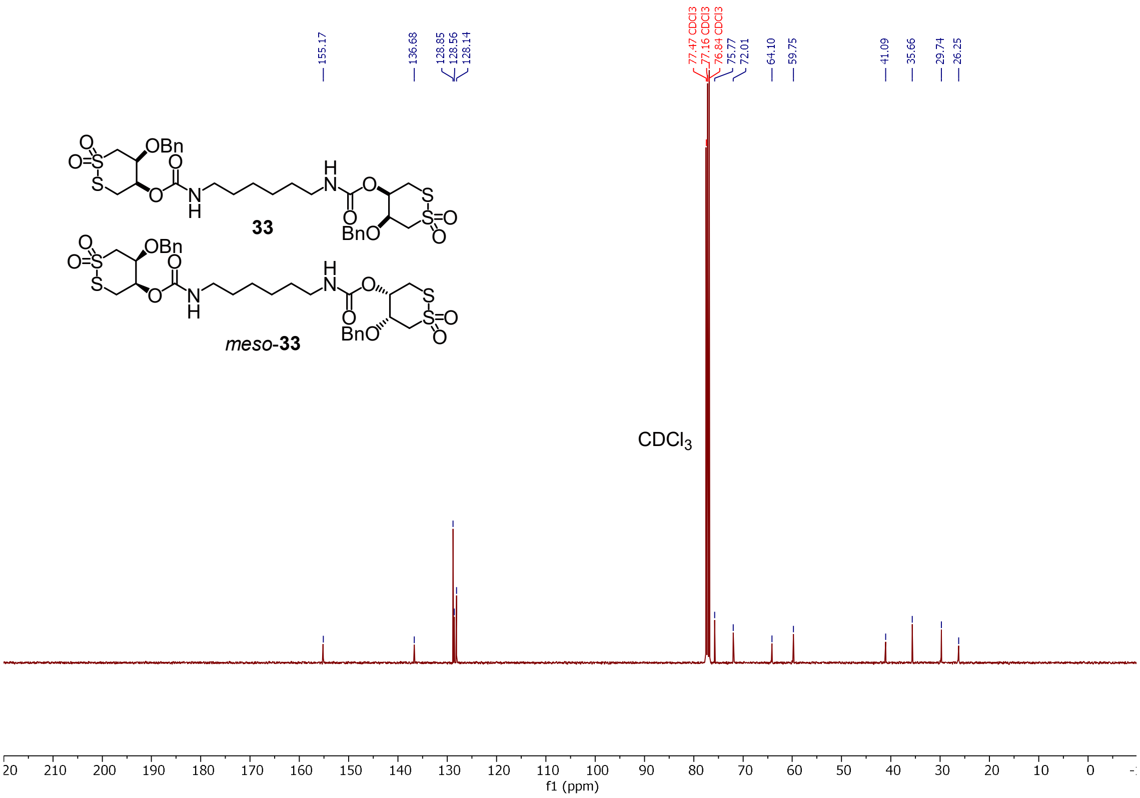

Figure S155. ^{13}C NMR (101 MHz, $CDCl_3$) spectrum of **33** and ***meso*-33**.

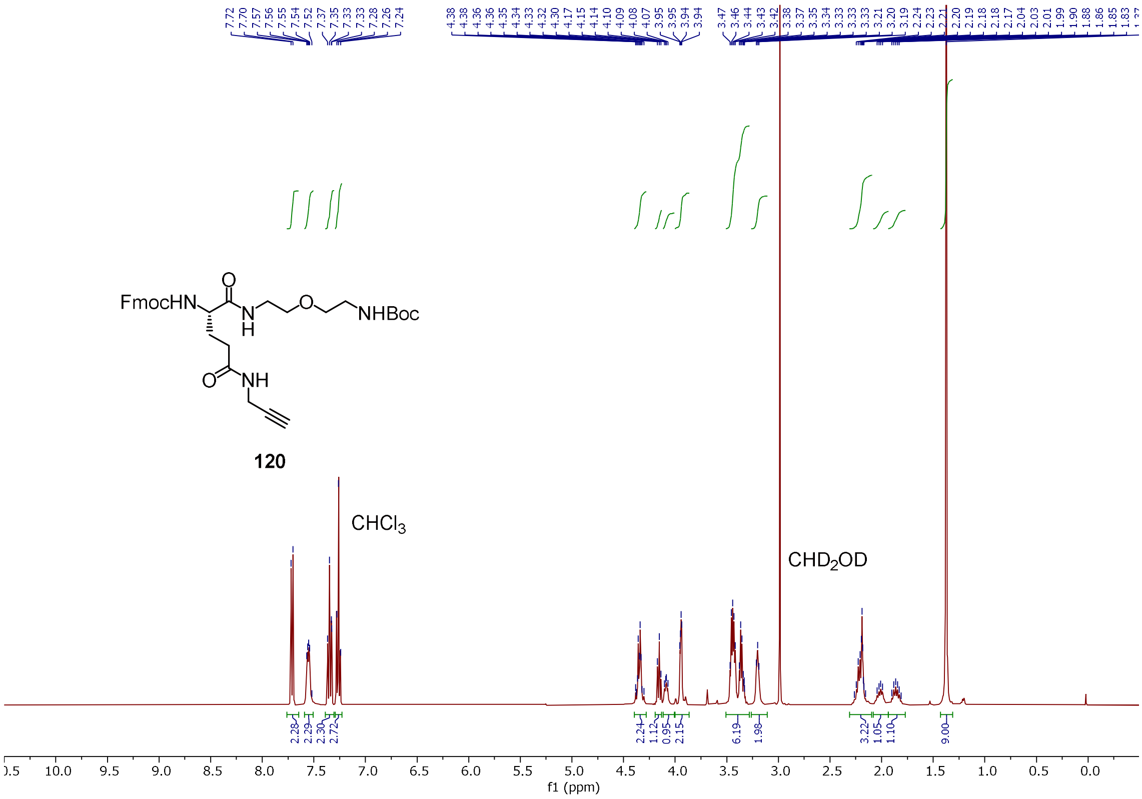

Figure S156. ^{1}H NMR (400 MHz, $CDCl_3/CD_3OD$ 1:1) spectrum of **120**.

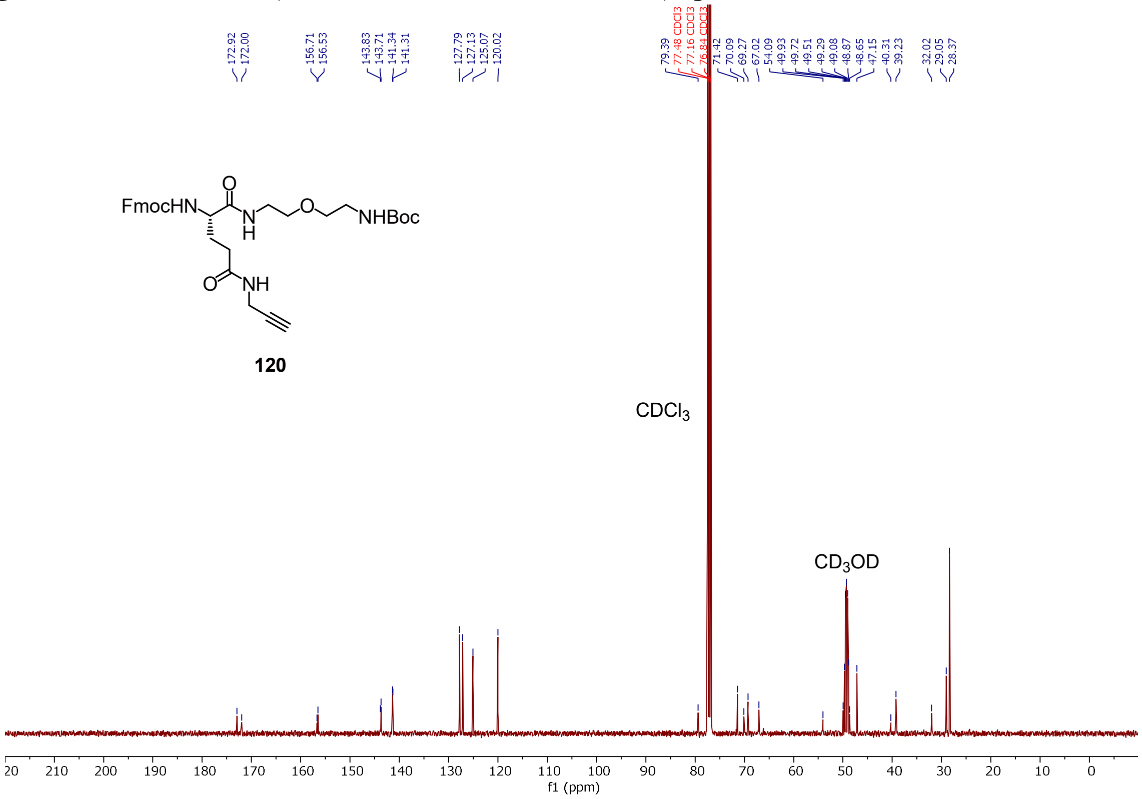

Figure S157. ^{13}C NMR (101 MHz, $CDCl_3/CD_3OD$ 1:1) spectrum of **120**.

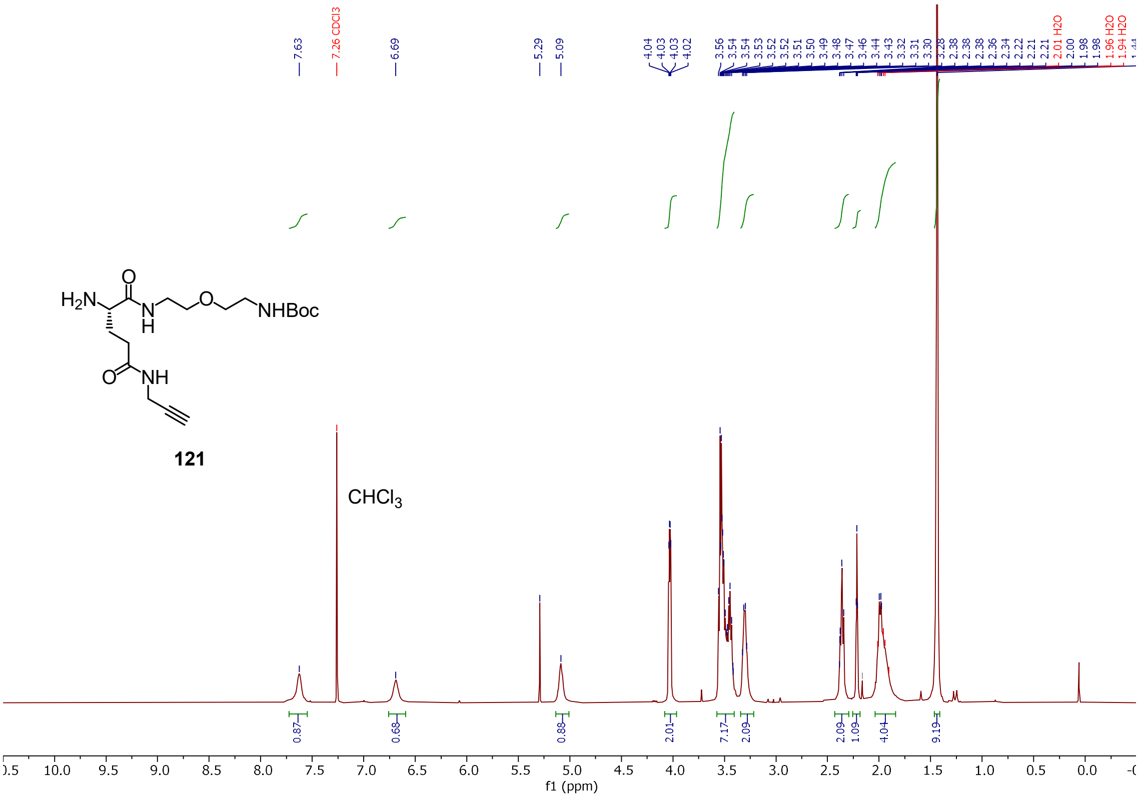

Figure S158. ^{1}H NMR (400 MHz, $CDCl_3$) spectrum of **121**.

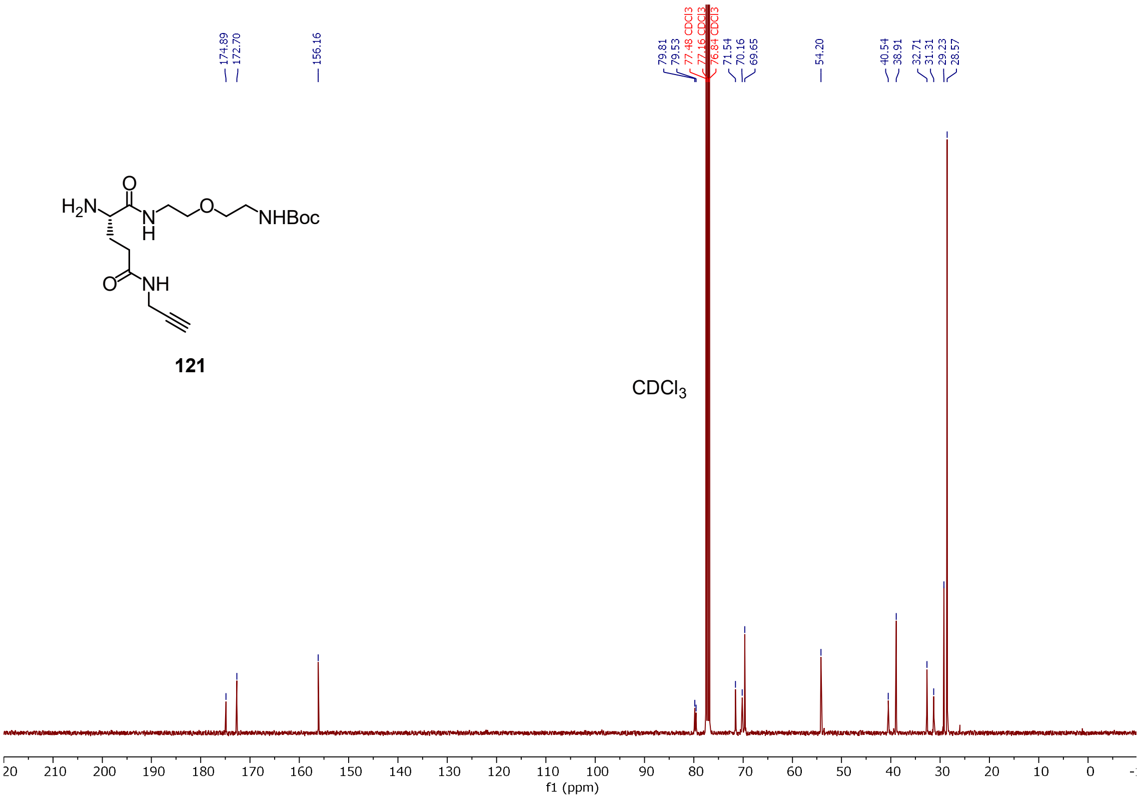

Figure S159. ^{13}C NMR (101 MHz, $CDCl_3$) spectrum of **121**.

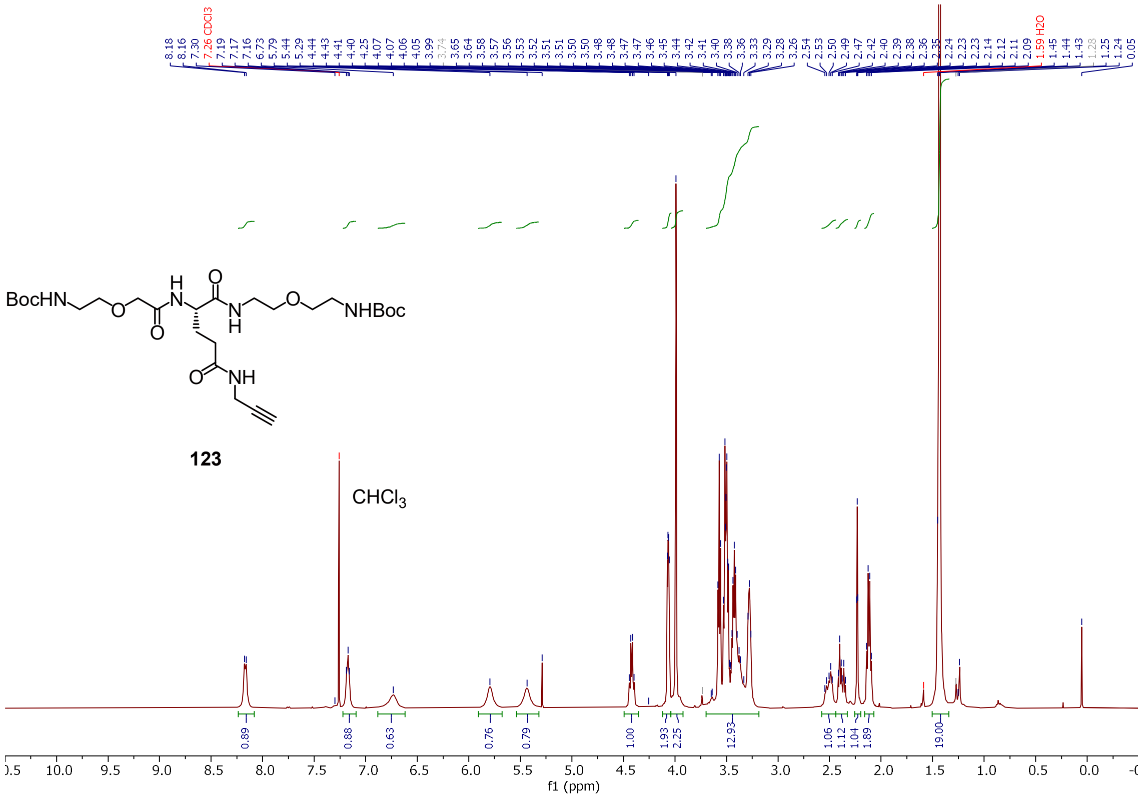

Figure S160. ^{1}H NMR (400 MHz, $CDCl_3$) spectrum of **123**.

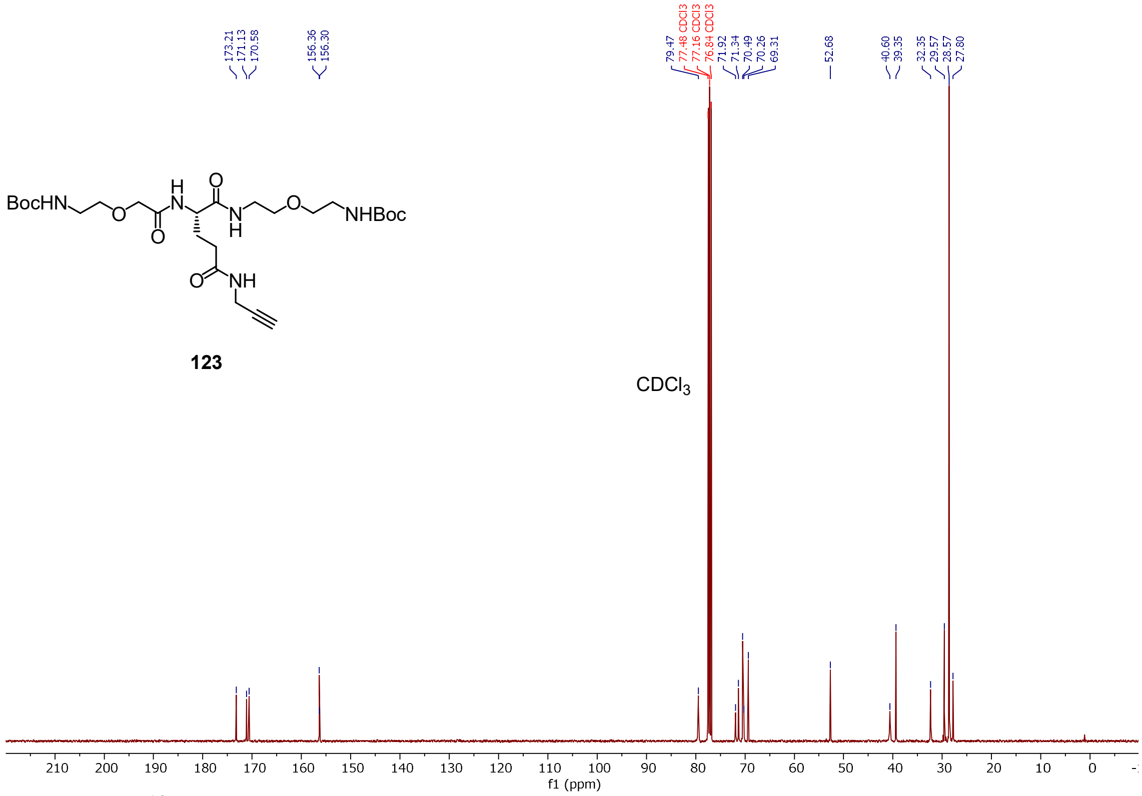

Figure S161. ^{13}C NMR (101 MHz, $CDCl_3$) spectrum of **123**.

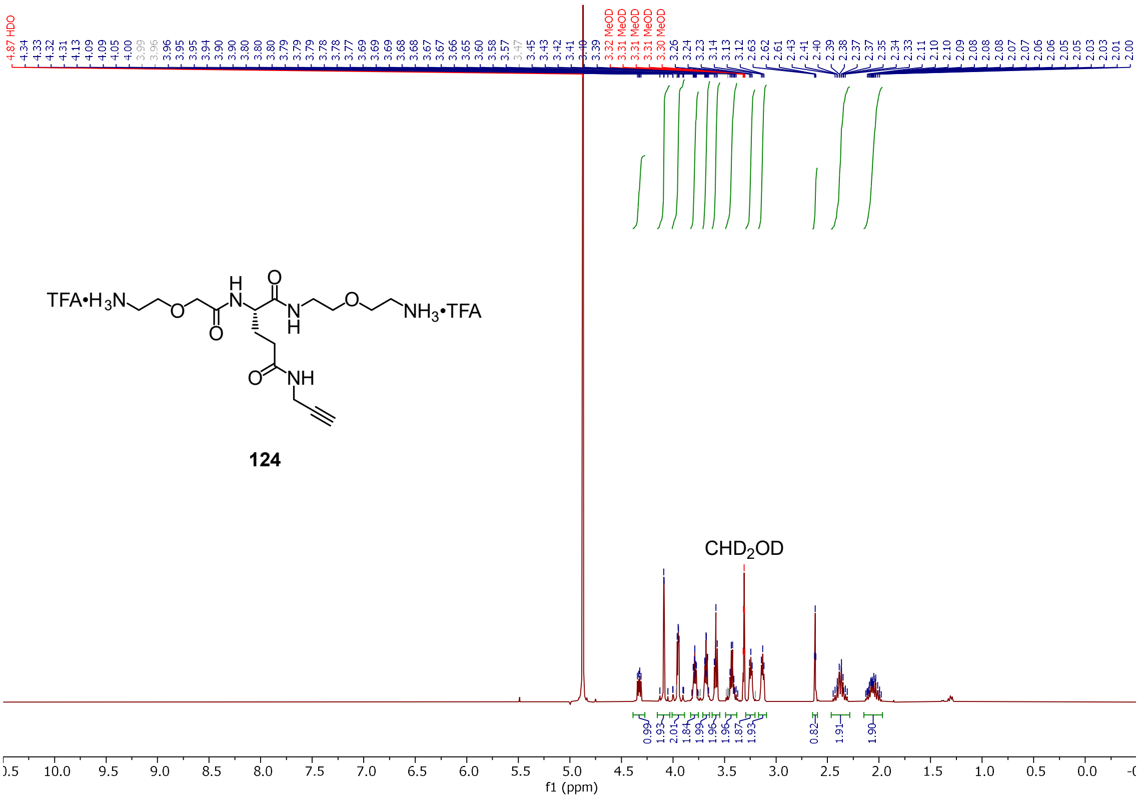

Figure S162. ^{1}H NMR (400 MHz, CD_3OD) spectrum of **124**.

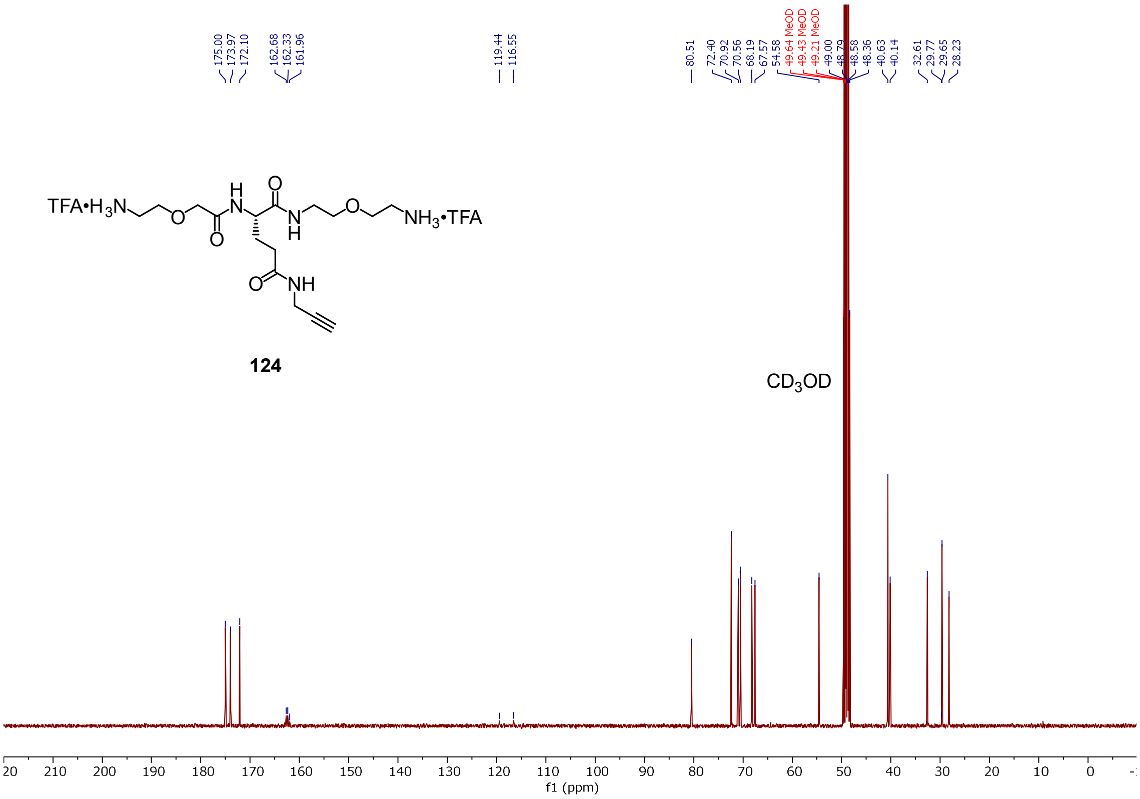

Figure S163. ^{13}C NMR (101 MHz, CD_3OD) spectrum of **124**.

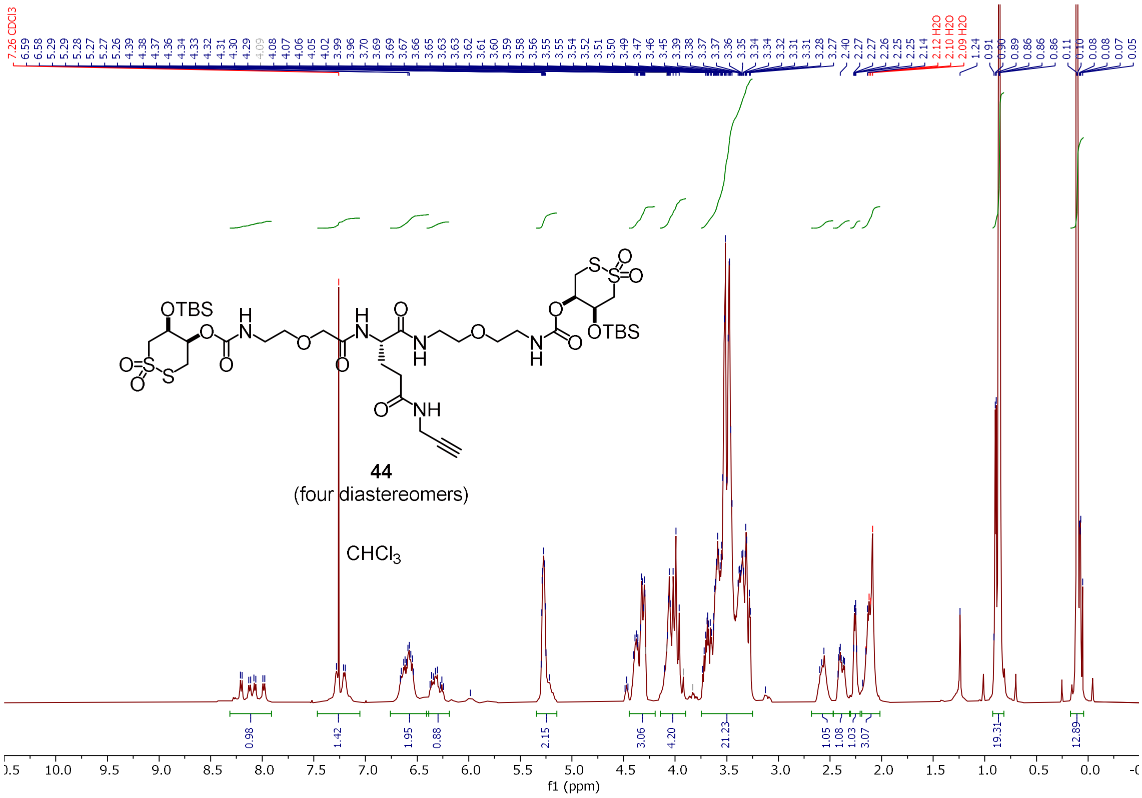

Figure S164. ^{1}H NMR (400 MHz, $CDCl_3$) spectrum of **44**.

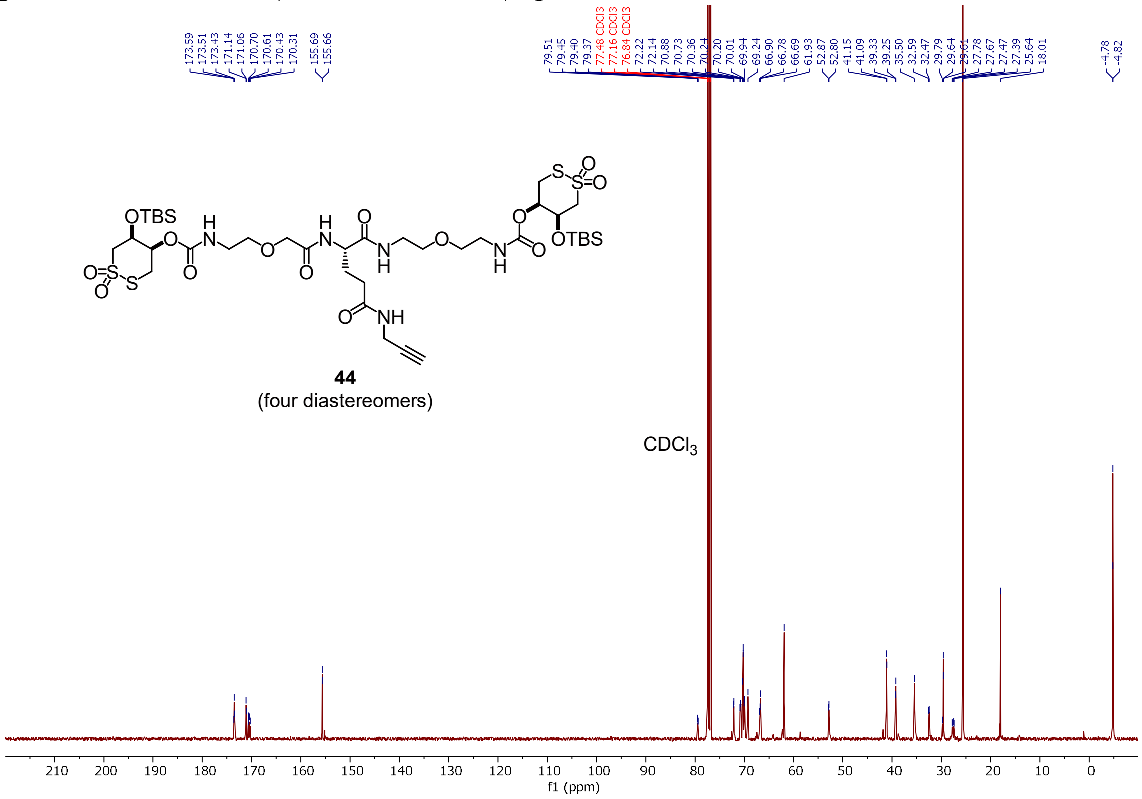

Figure S165. ^{13}C NMR (101 MHz, $CDCl_3$) spectrum of **44**.

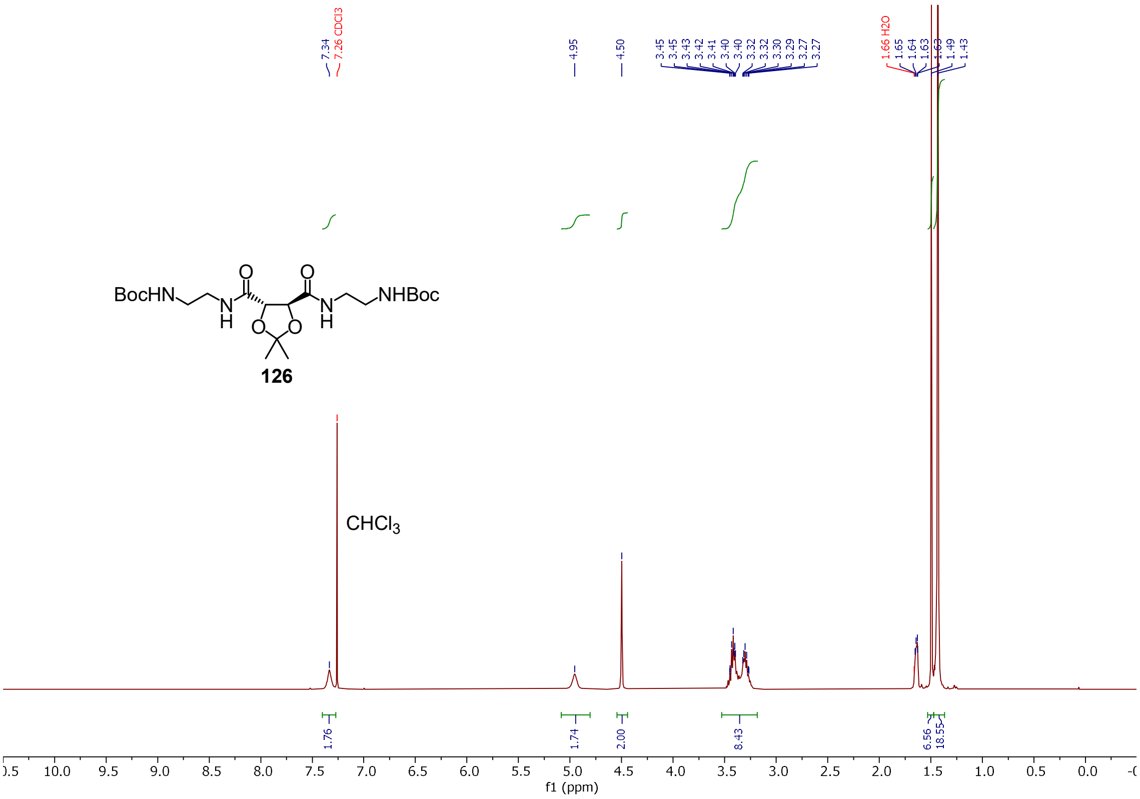

Figure S166. ^{1}H NMR (400 MHz, $CDCl_3$) spectrum of **126**.

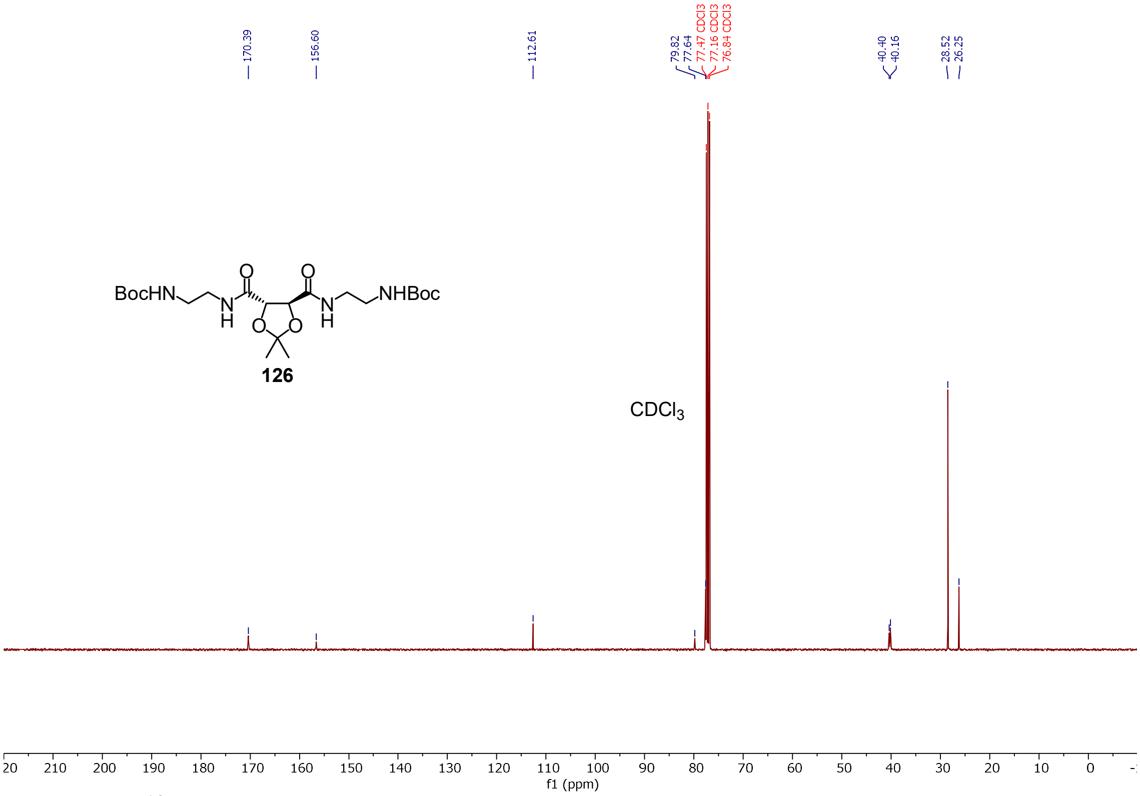

Figure S167. ^{13}C NMR (101 MHz, $CDCl_3$) spectrum of **126**.

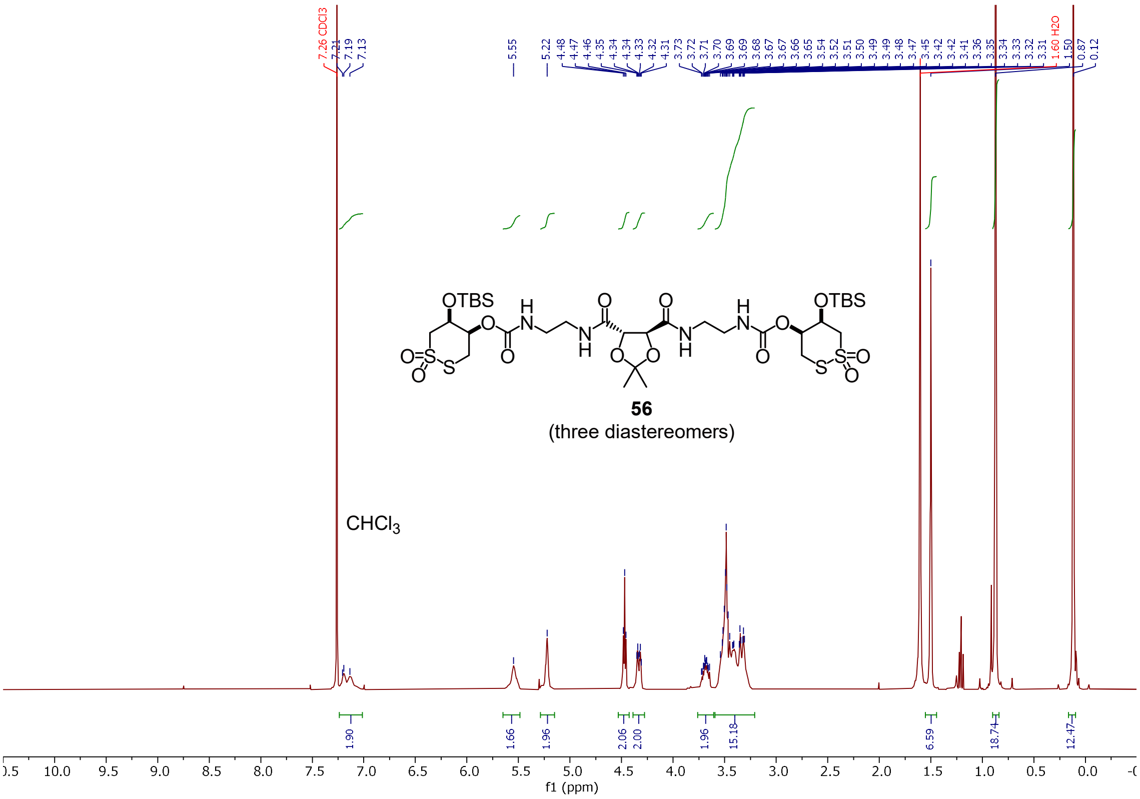

Figure S168. ^{1}H NMR (400 MHz, $CDCl_3$) spectrum of **56**.

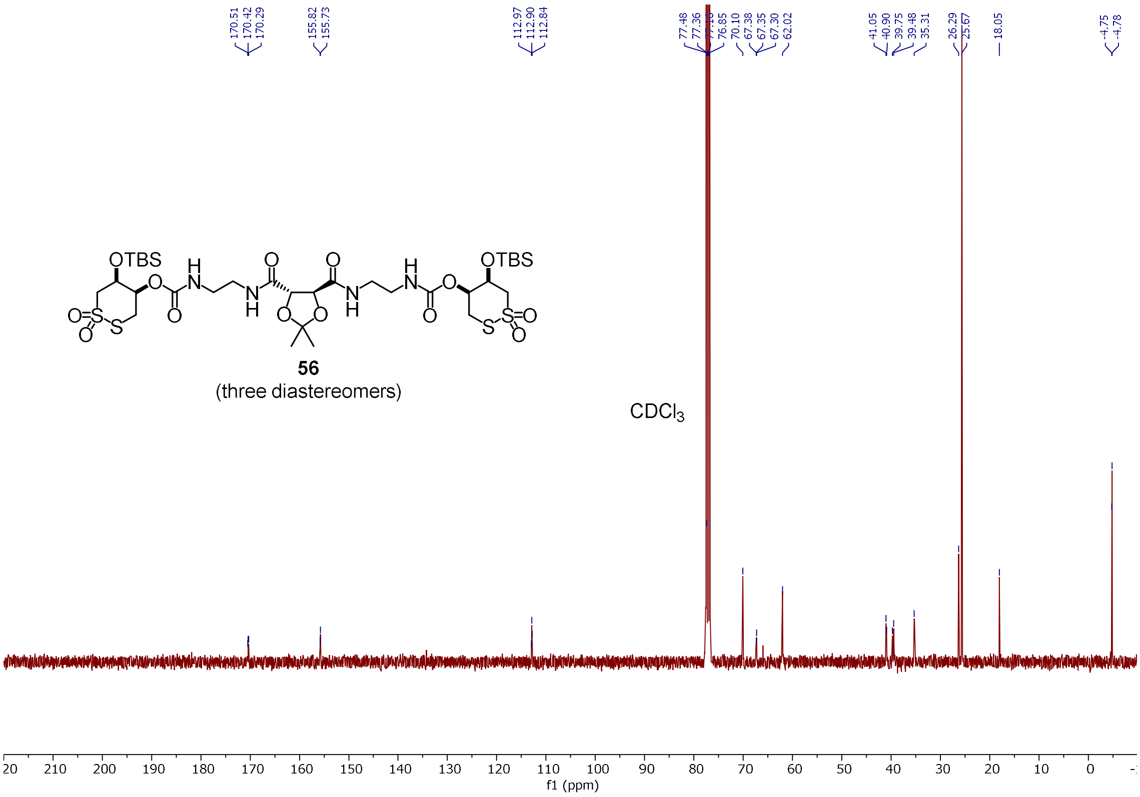

Figure S169. ^{13}C NMR (101 MHz, $CDCl_3$) spectrum of **56**.

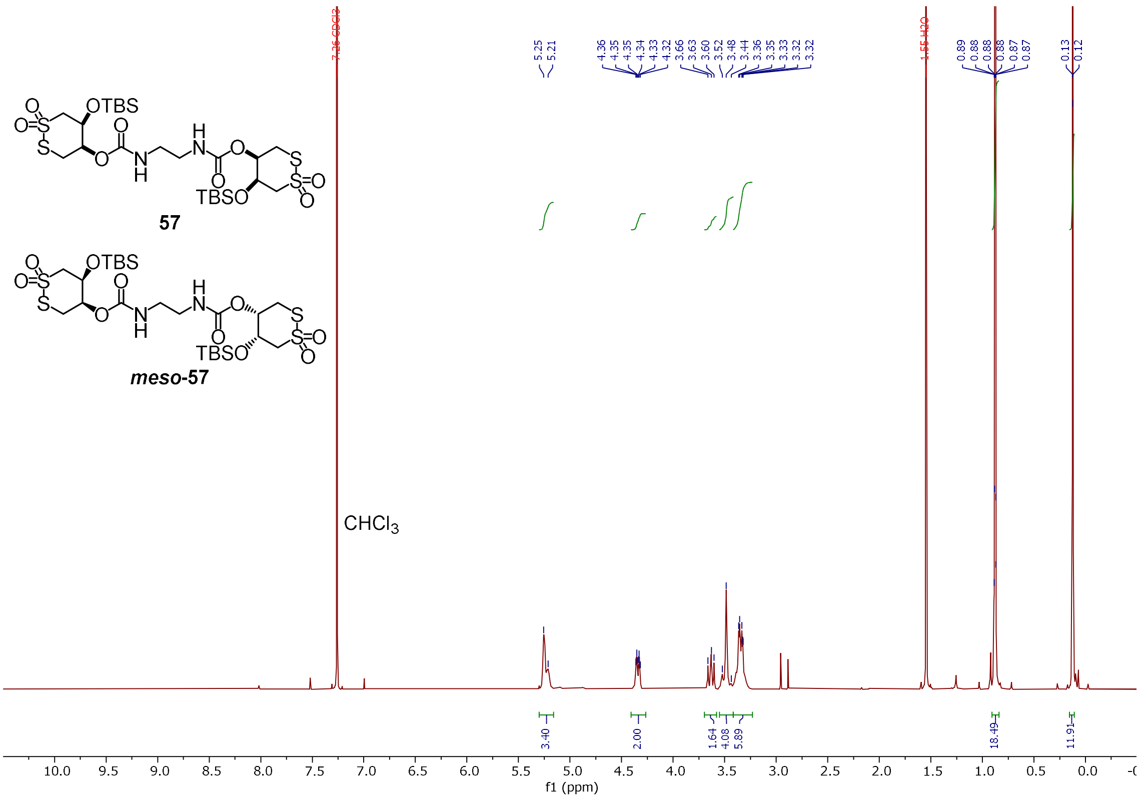

Figure S170. ^{1}H NMR (400 MHz, $CDCl_3$) spectrum of **57** and ***meso*-57**.

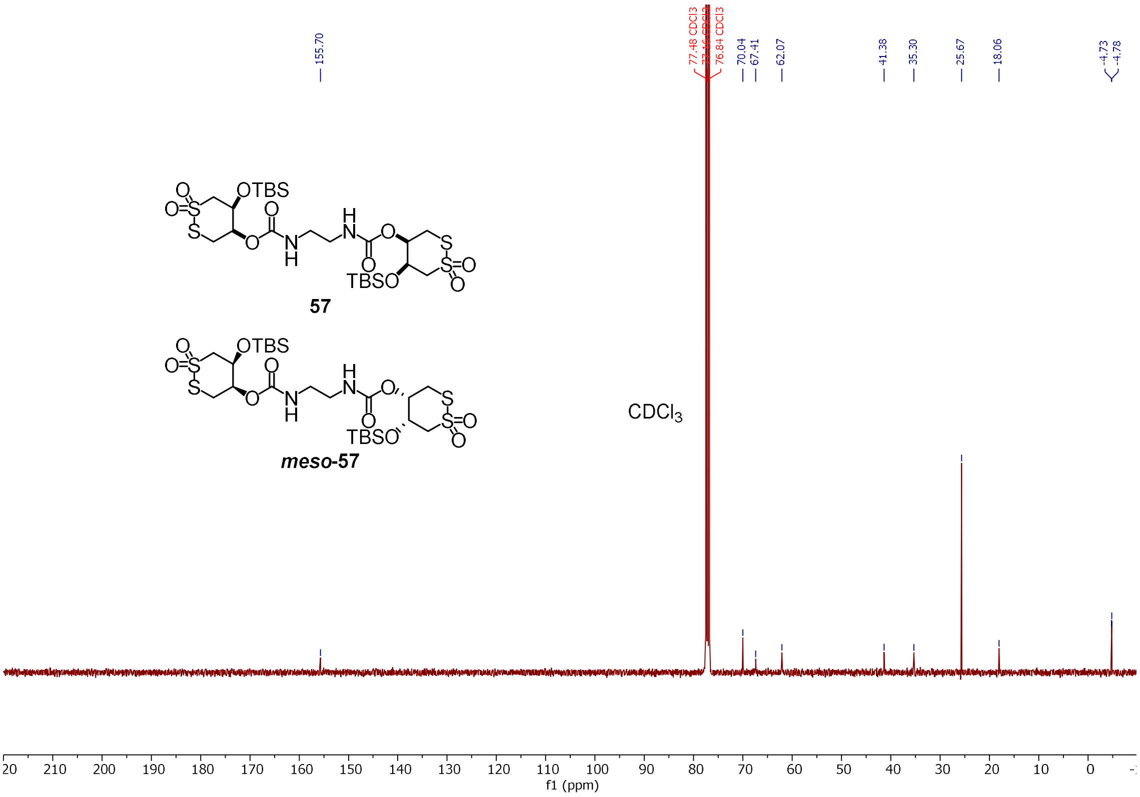

Figure S171. ^{13}C NMR (101 MHz, $CDCl_3$) spectrum of **57** and ***meso*-57**.

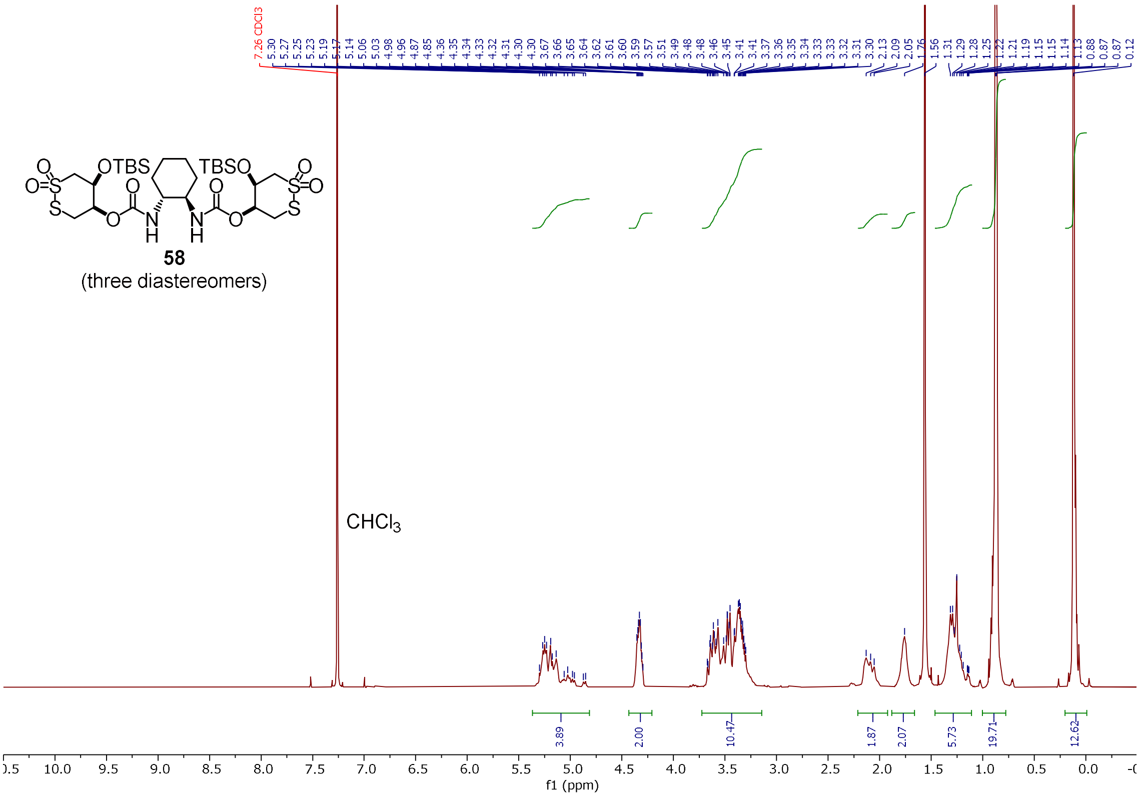

Figure S172. ^{1}H NMR (400 MHz, $CDCl_3$) spectrum of **58**.

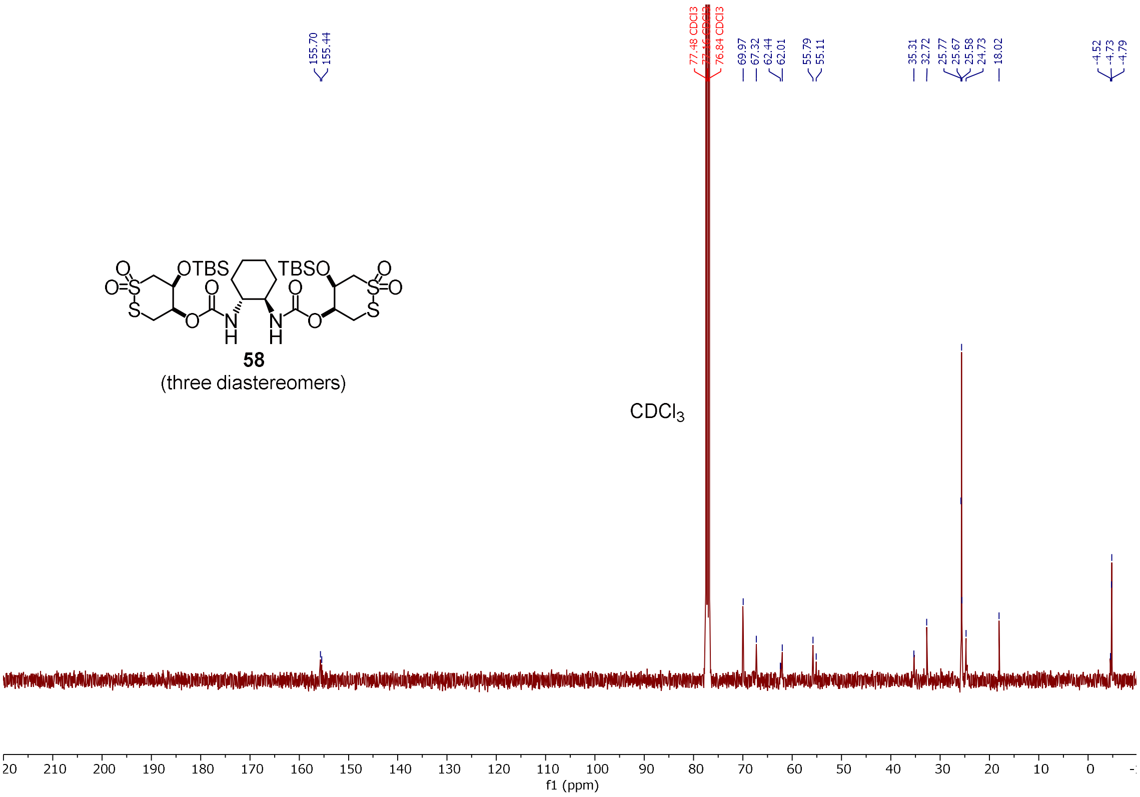

Figure S173. ^{13}C NMR (101 MHz, $CDCl_3$) spectrum of **58**.

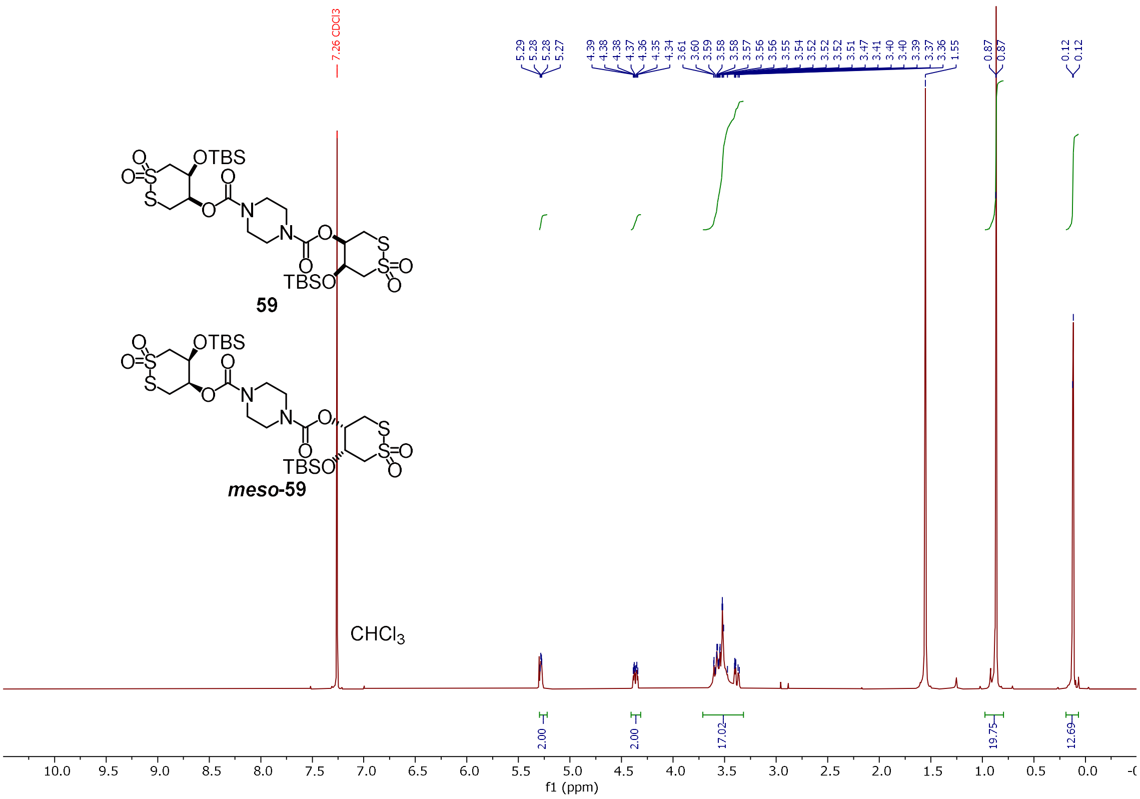

Figure S174. ^{1}H NMR (400 MHz, $CDCl_3$) spectrum of **59** and ***meso*-59**.

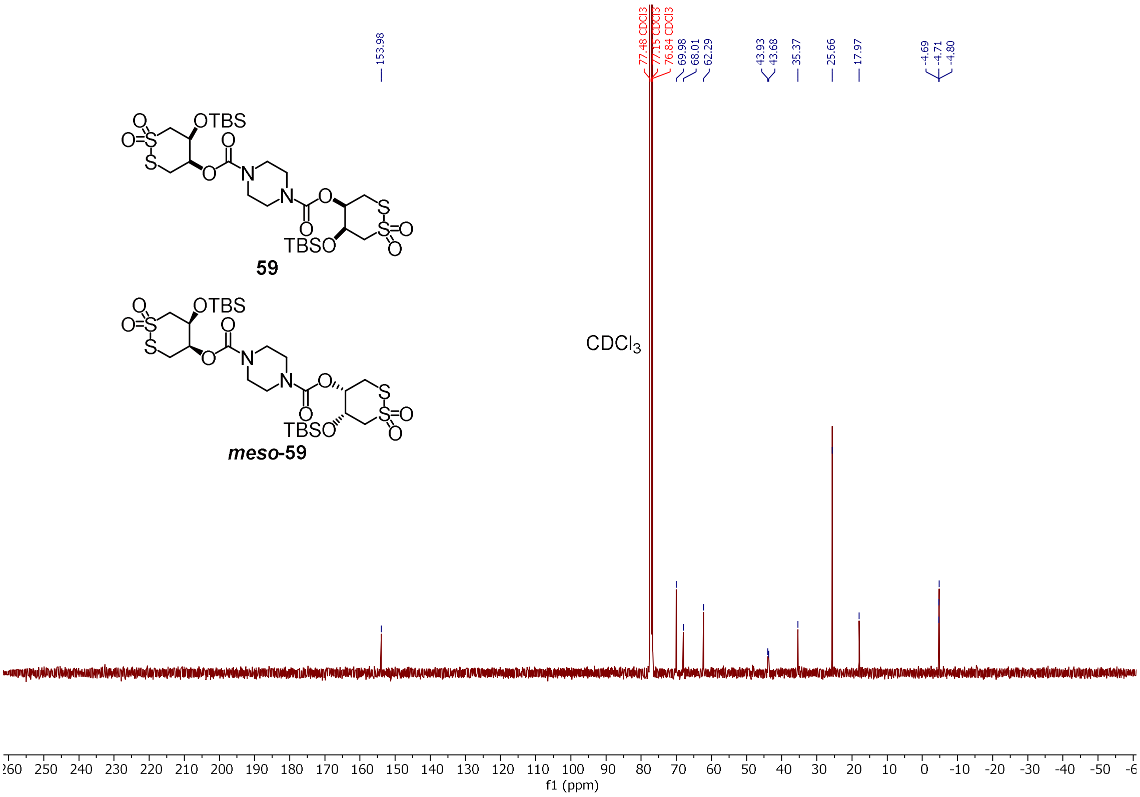

Figure S175. ^{13}C NMR (101 MHz, $CDCl_3$) spectrum of **59** and ***meso*-59**.

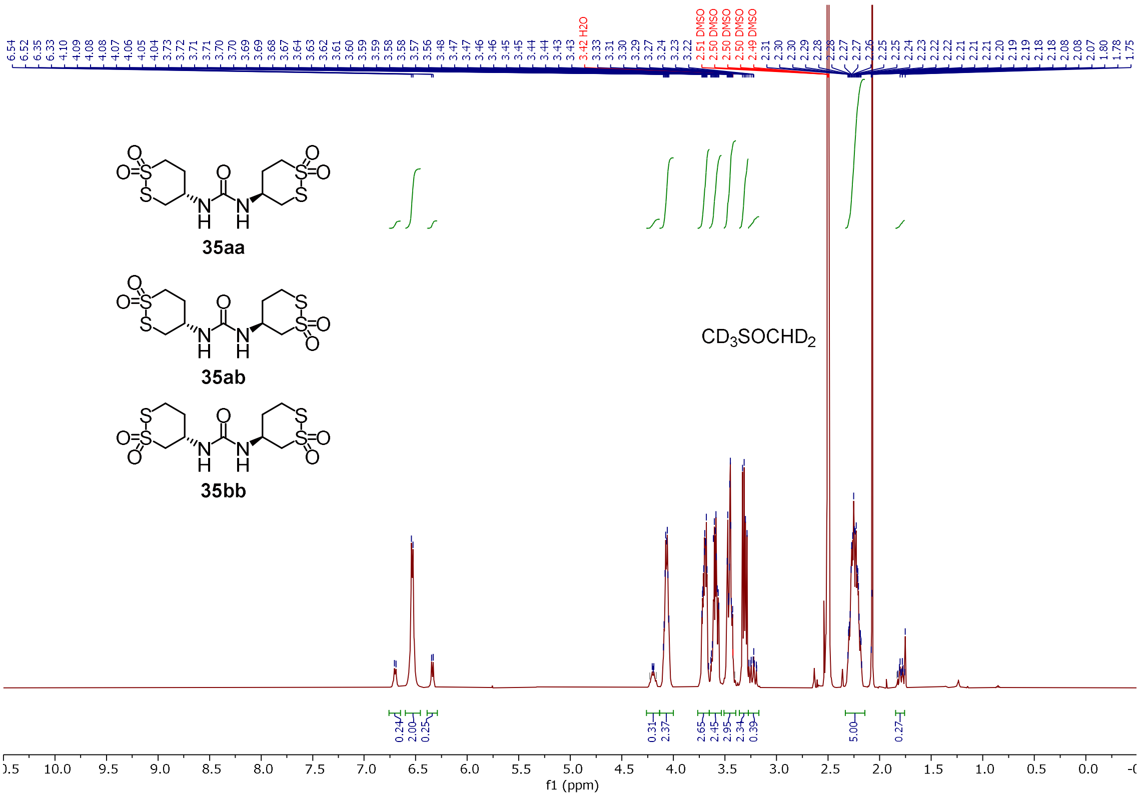

Figure S176. ^{1}H NMR (500 MHz, DMSO-d_6) spectrum of **35**.

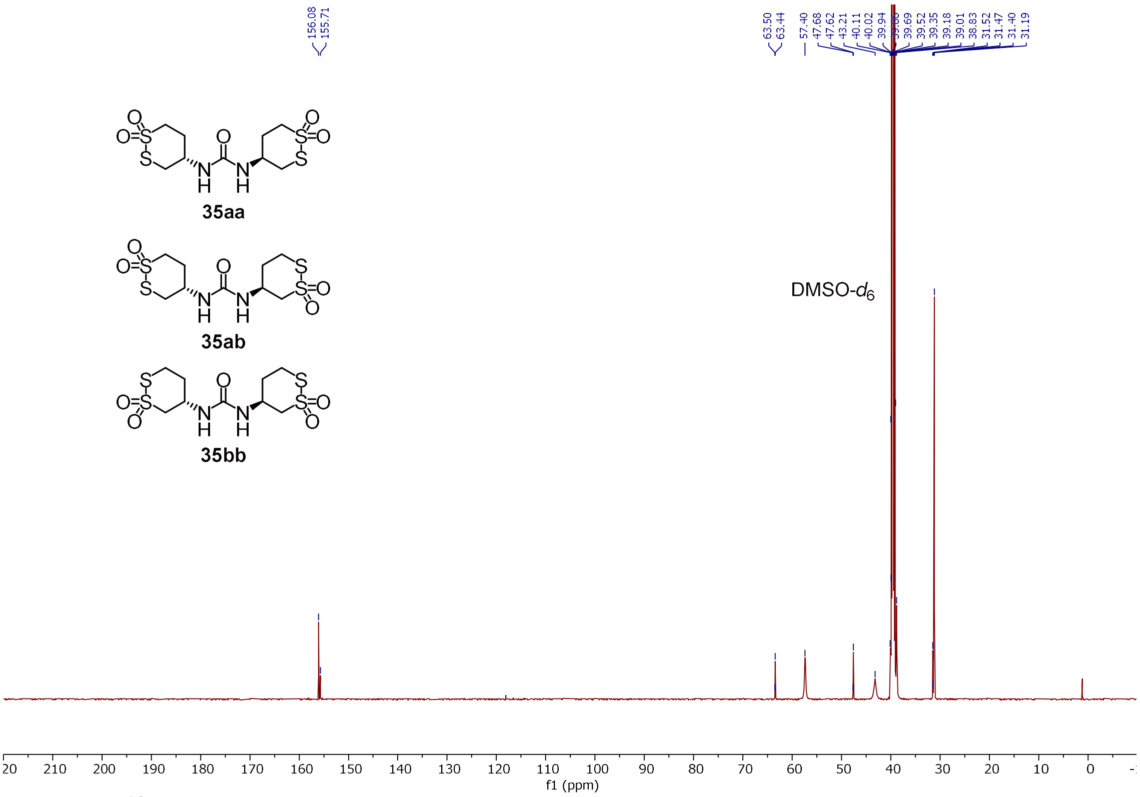

Figure S177. ^{13}C NMR (126 MHz, DMSO-d_6) spectrum of **35**.

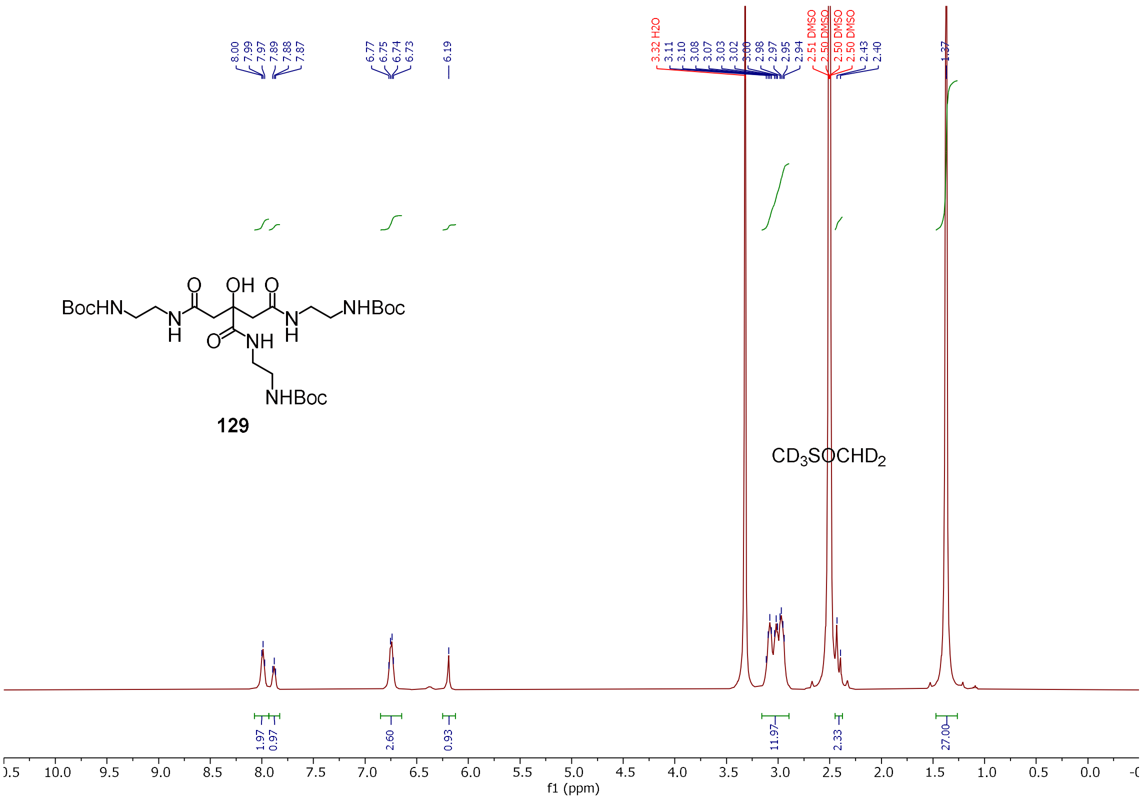

Figure S178. ^{1}H NMR (400 MHz, DMSO-d_6) spectrum of **129**.

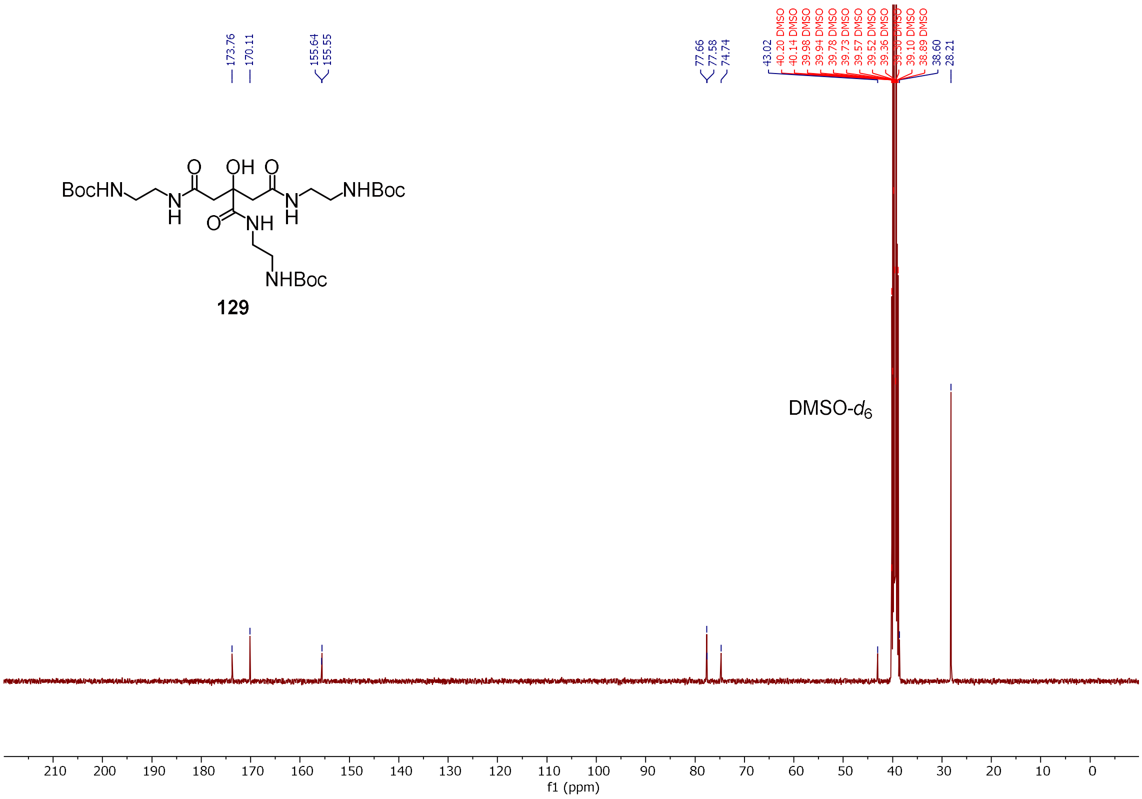

Figure S179. ^{13}C NMR (101 MHz, DMSO-d_6) spectrum of **129**.

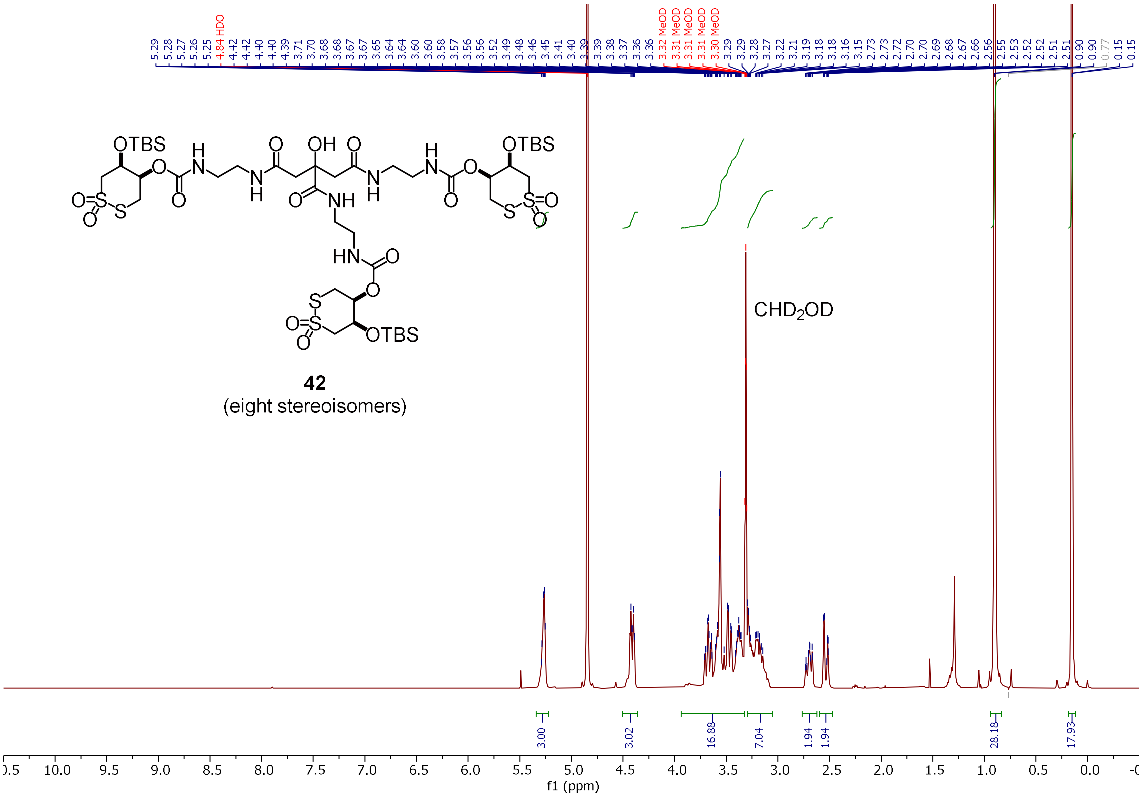

Figure S180. ^{1}H NMR (400 MHz, CD_3OD) spectrum of **42**.

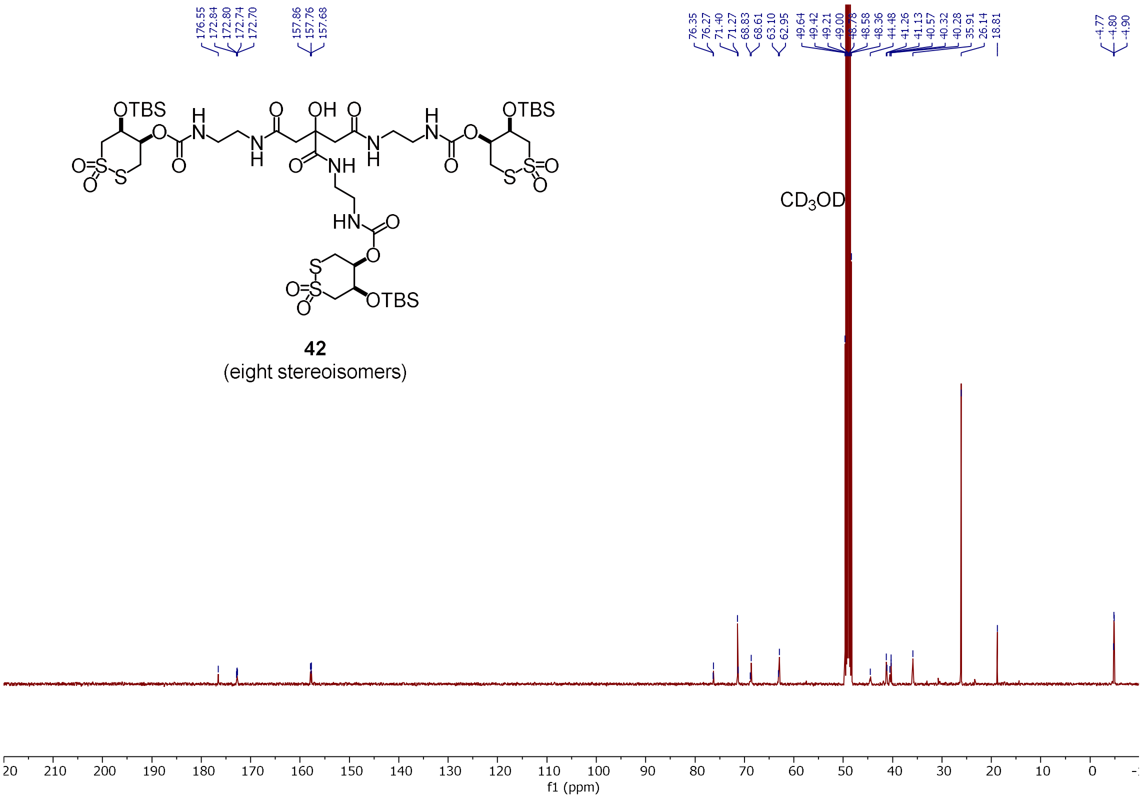

Figure S181. ^{13}C NMR (101 MHz, CD_3OD) spectrum of **42**.

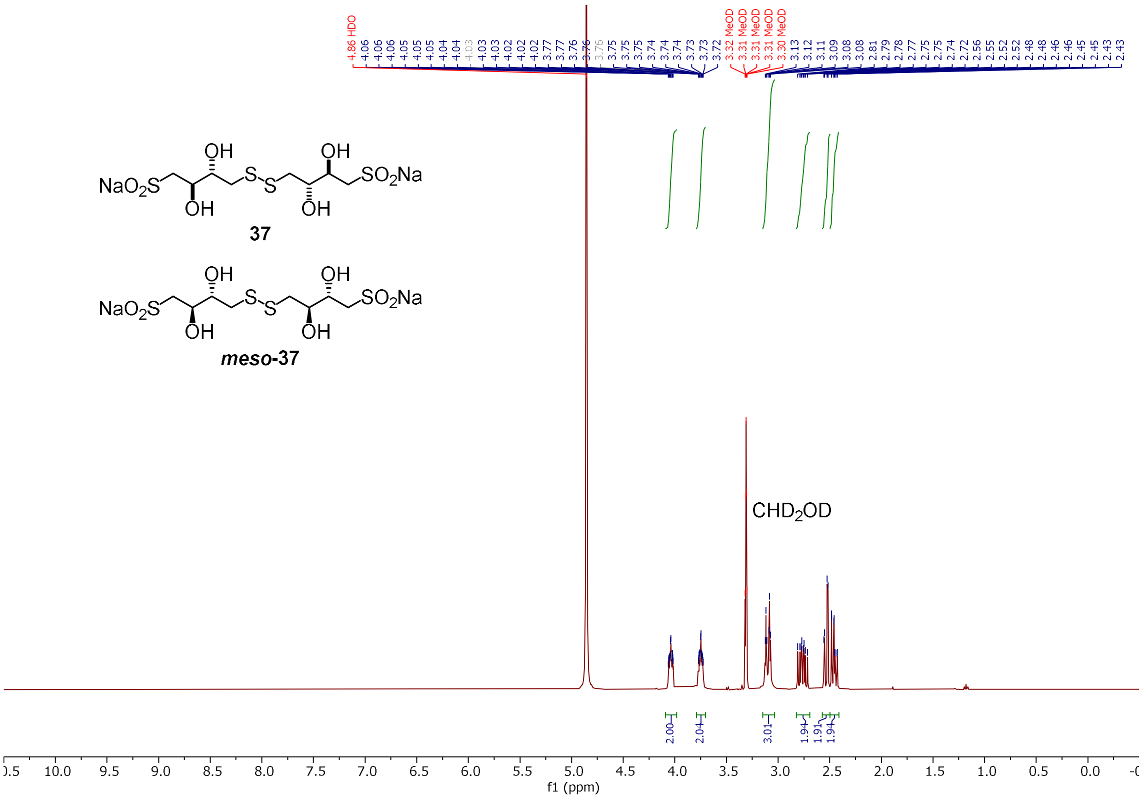

Figure S182. ^{1}H NMR (400 MHz, CD_3OD) spectrum of **37** and ***meso*-37**.

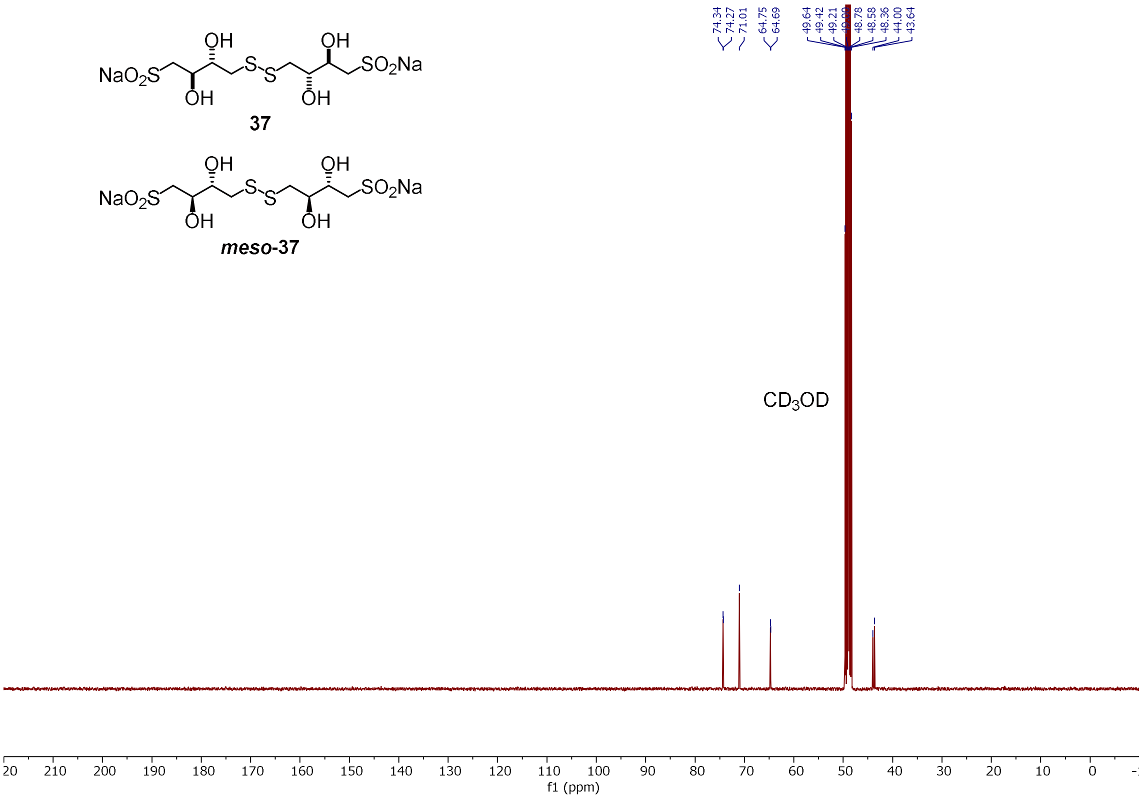

Figure S183. ^{13}C NMR (101 MHz, CD_3OD) spectrum of **37** and ***meso*-37**.

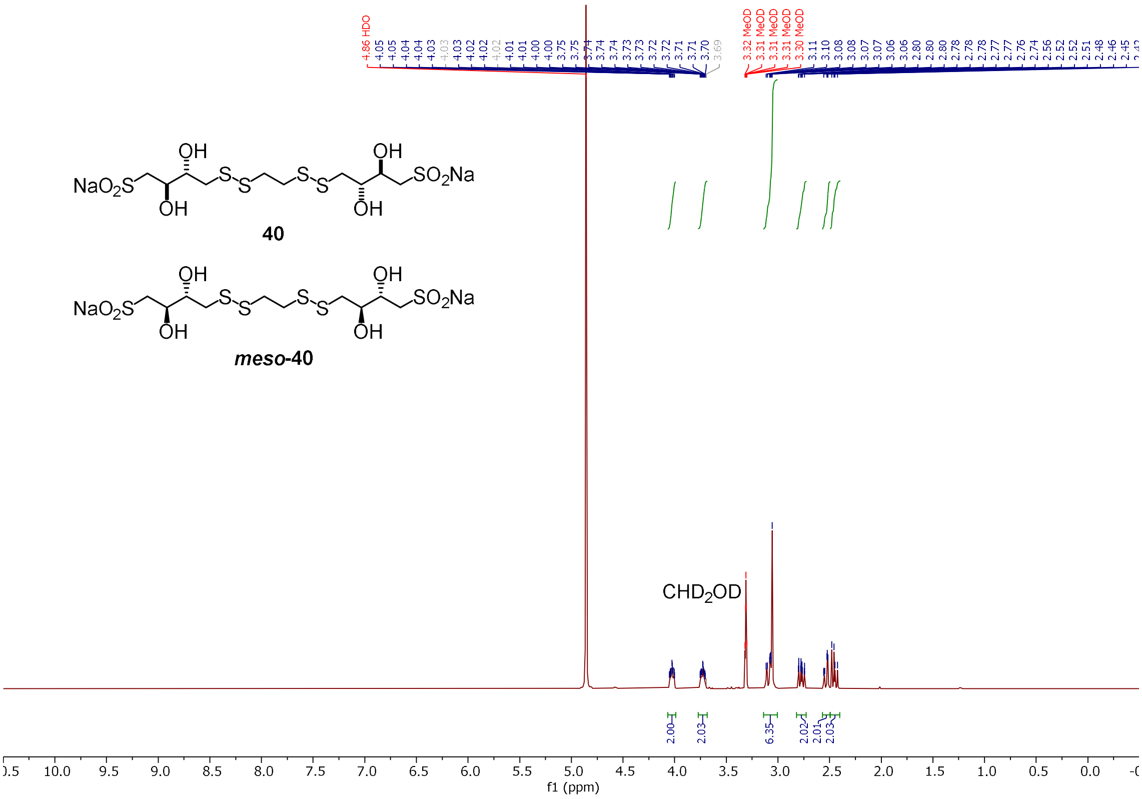

Figure S184. ^{1}H NMR (400 MHz, CD_3OD) spectrum of **40** and ***meso*-40**.

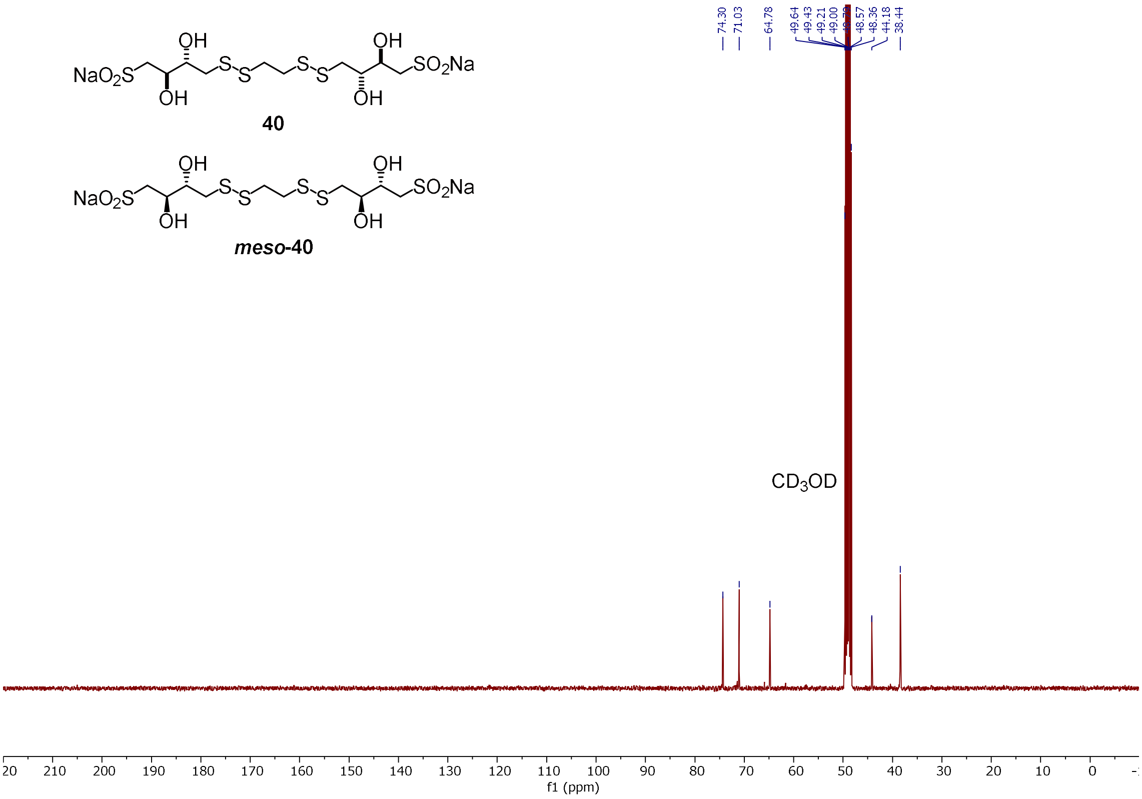

Figure S185. ^{13}C NMR (101 MHz, CD_3OD) spectrum of **40** and ***meso*-40**.

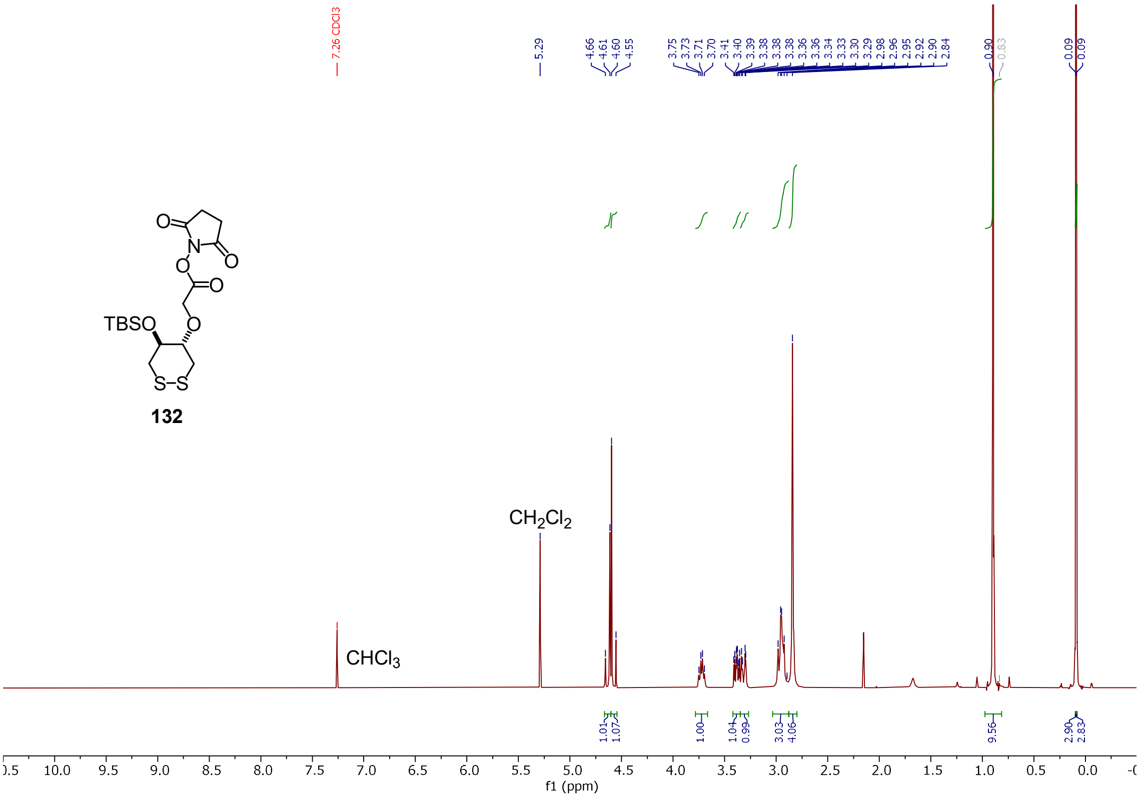

Figure S186. ^{1}H NMR (400 MHz, $CDCl_3$) spectrum of **132**.

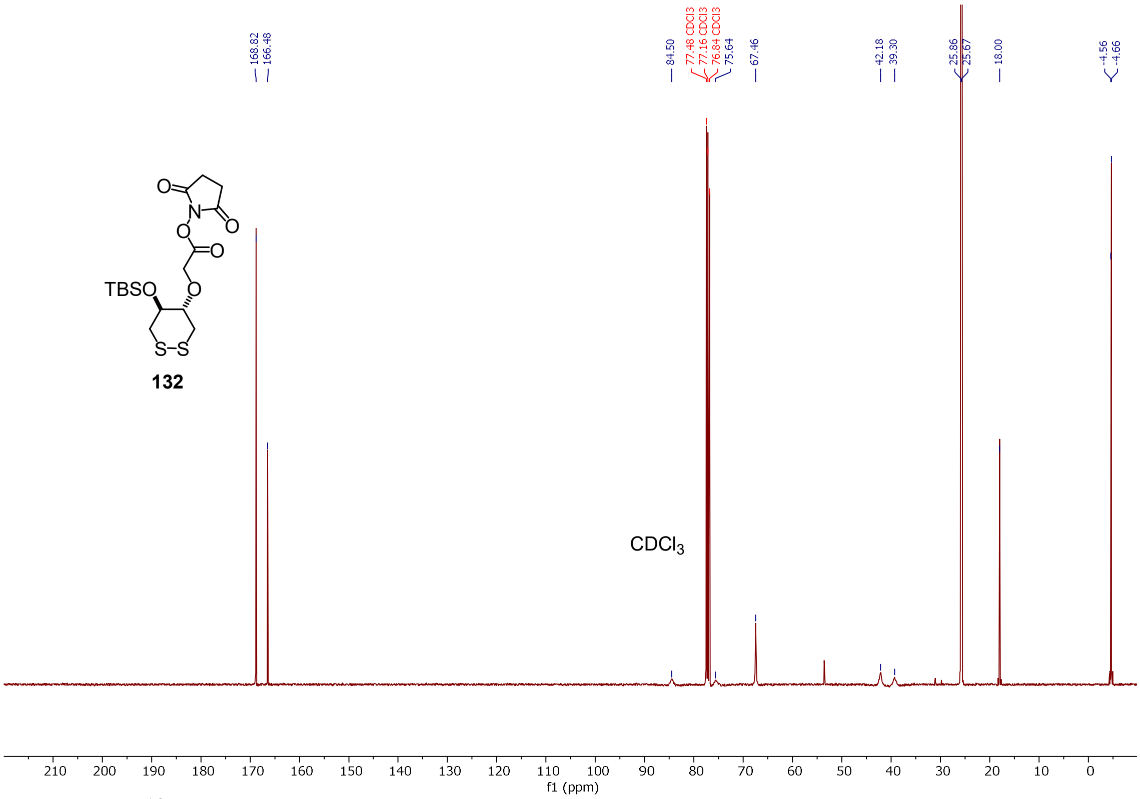

Figure S187. ^{13}C NMR (101 MHz, $CDCl_3$) spectrum of **132**.

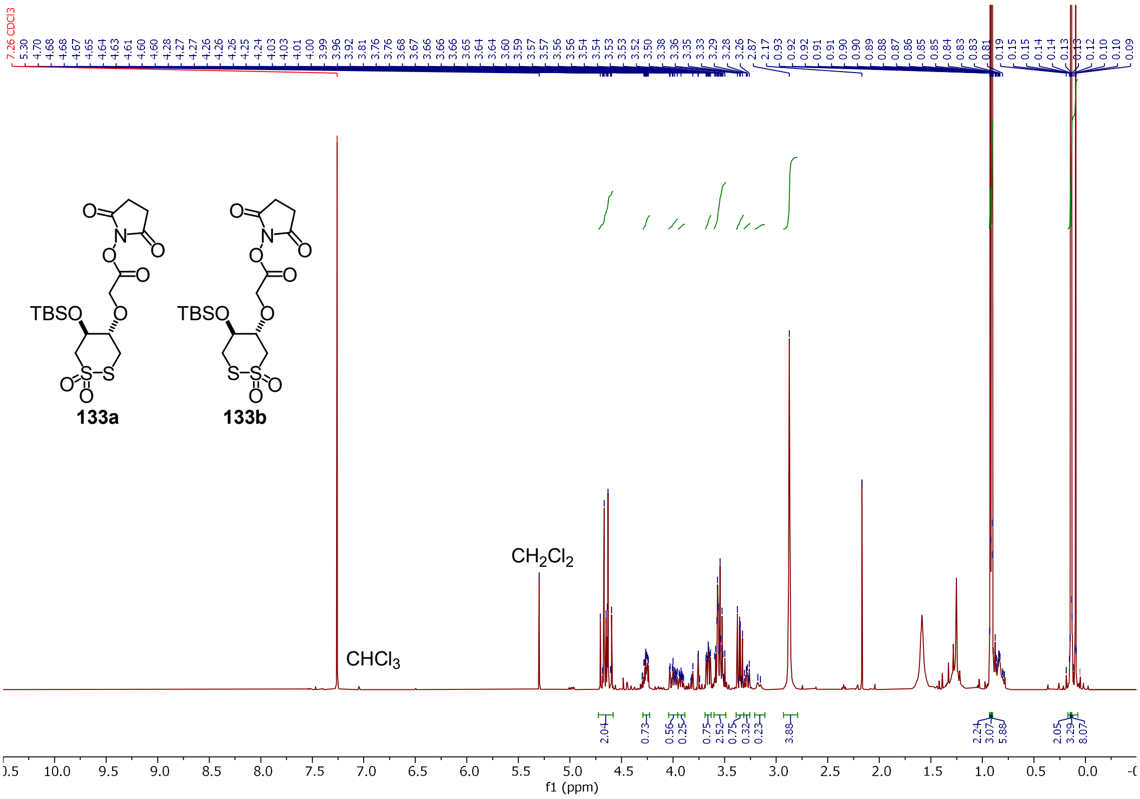

Figure S188. ^{1}H NMR (500 MHz, $CDCl_3$) spectrum of **133**.

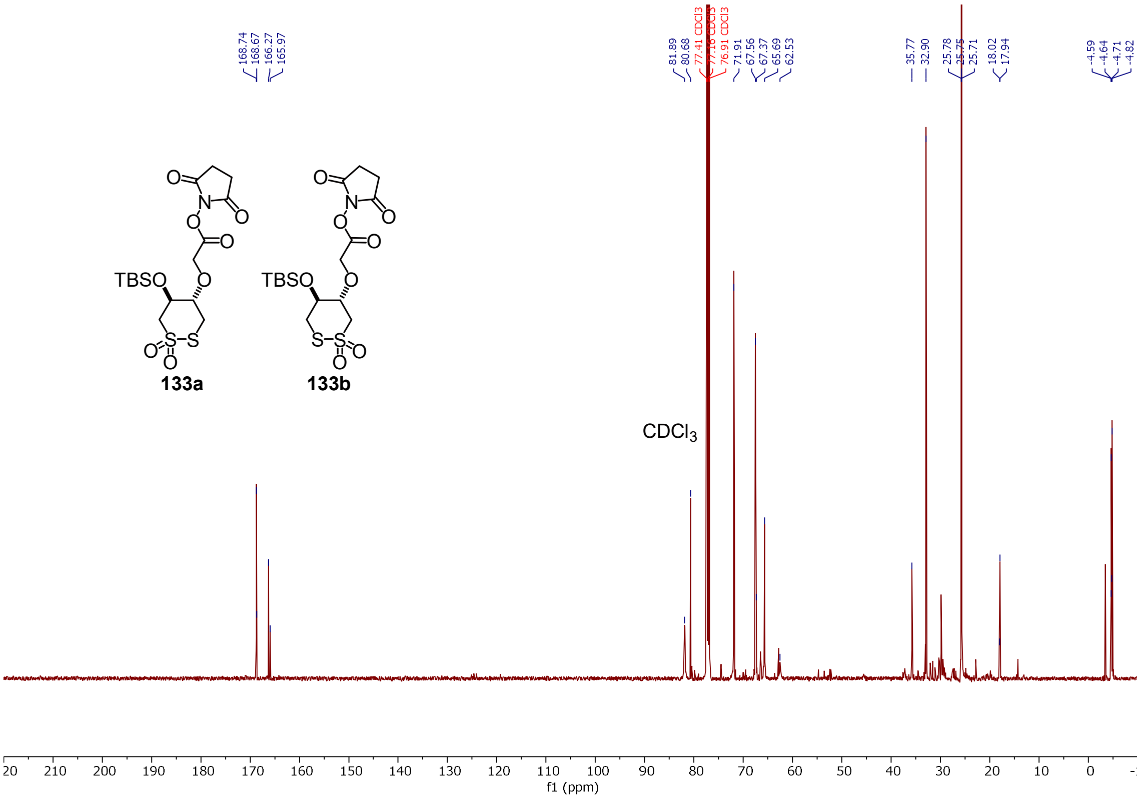

Figure S189. ^{13}C NMR (126 MHz, $CDCl_3$) spectrum of **133**.

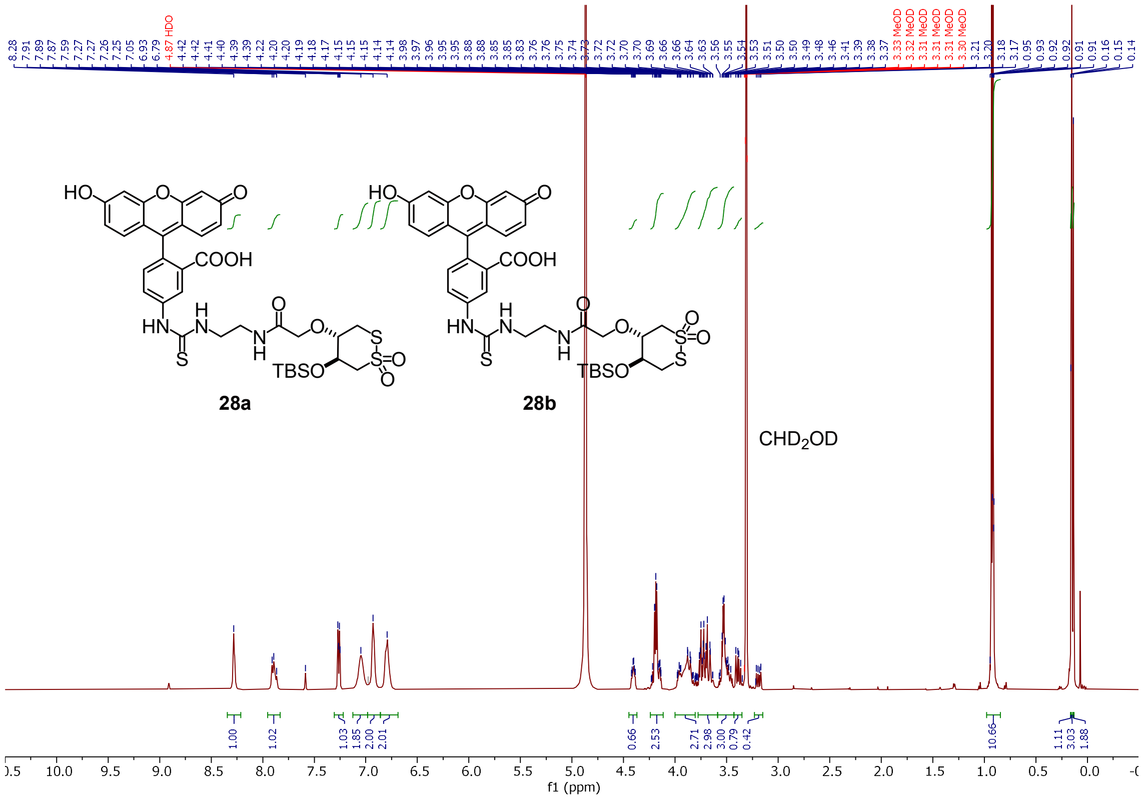

Figure S190. ^{1}H NMR (500 MHz, CD$_3$OD) spectrum of **28**.

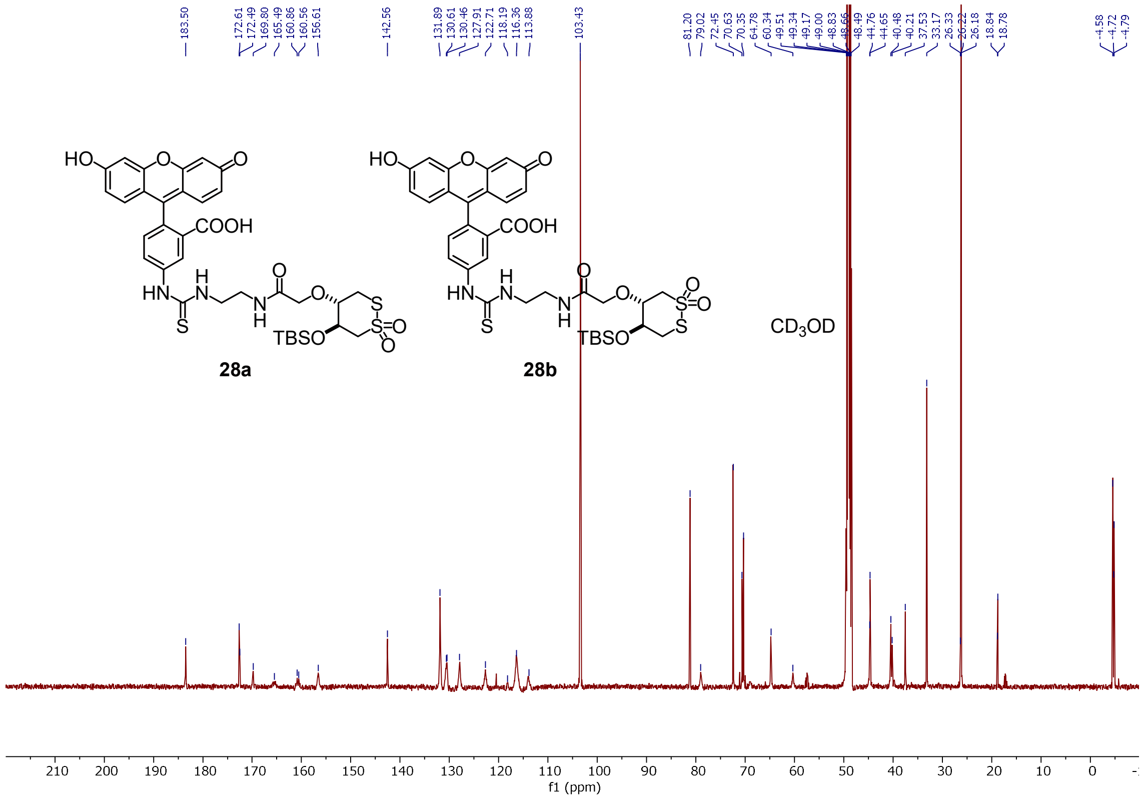

Figure S191. ^{13}C NMR (126 MHz, CD$_3$OD) spectrum of **28**.

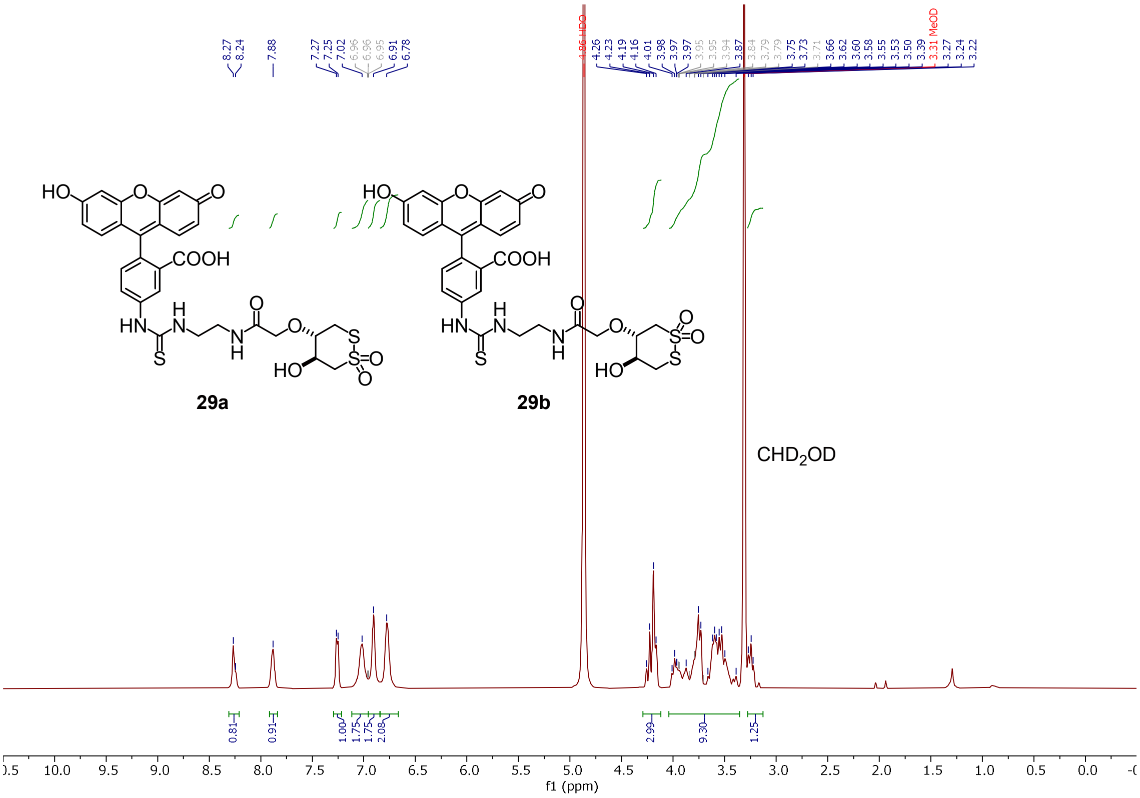

Figure S192. ^{1}H NMR (500 MHz, CD_3OD) spectrum of **29**.

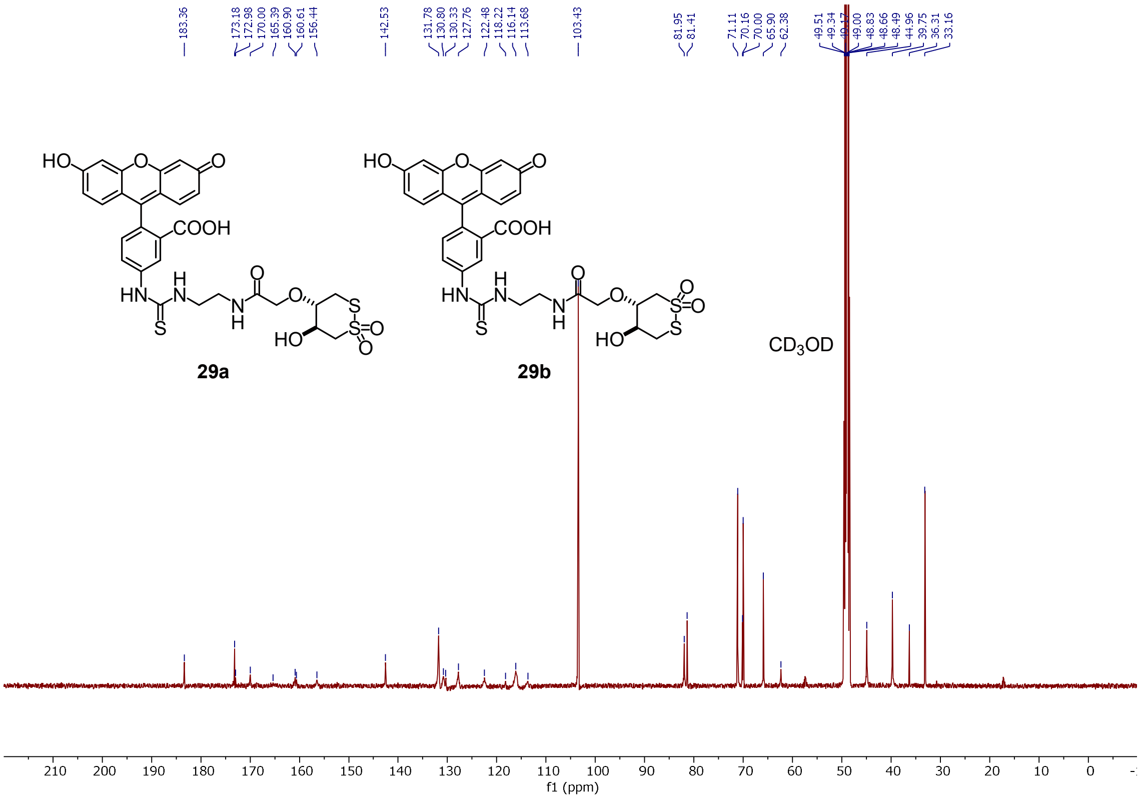

Figure S193. ^{13}C NMR (126 MHz, CD_3OD) spectrum of **29**.
